# Isolation and Genomic Characterization of a Heat-Labile Enterotoxin 1-Producing Escherichia fergusonii Strain from a Human

**DOI:** 10.1128/spectrum.00491-23

**Published:** 2023-07-11

**Authors:** Miki Okuno, Nami Tsuru, Shuji Yoshino, Yasuhiro Gotoh, Takeshi Yamamoto, Tetsuya Hayashi, Yoshitoshi Ogura

**Affiliations:** a Division of Microbiology, Department of Infectious Medicine, Kurume University School of Medicine, Kurume, Japan; b Miyazaki Prefectural Institute for Public Health and Environment, Miyazaki, Japan; c Department of Bacteriology, Faculty of Medical Sciences, Kyushu University, Fukuoka, Japan; Cinvestav-IPN

**Keywords:** *Escherichia fergusonii*, heat-labile enterotoxin, genome, plasmid, antimicrobial resistance

## Abstract

Escherichia fergusonii strains have been isolated from patients with diarrhea, but their virulence determinant has not been well elucidated. Here, we report the first isolation of a heat-labile enterotoxin 1 (LT1)-producing E. fergusonii strain (strain 30038) from a patient in Japan. The complete genome sequence of strain 30038 was determined and subjected to comparative genomics and phylogenetic analyses with 195 publicly available genomes of *E. fergusonii*. In addition to strain 30038, the *elt1* gene was also identified in an *E. fergusonii* strain that is phylogenetically distinct and which was isolated from poultry in the United Kingdom. Fine genomic comparison revealed that these two strains share comparable *elt1*-bearing plasmids. However, an intriguing distinction arises in strain 30038, wherein the plasmid has integrated into the chromosome via a recombination process mediated by an insertion sequence. The production of active LT1 toxin by strain 30038 was verified through an *in vitro* assay using cultured cells. A large plasmid carrying 11 antimicrobial resistance genes was also identified in strain 30038. Our results indicate that extensive surveillance of *elt1*-positive *E. fergusonii* strains as diarrheagenic pathogens is needed.

**IMPORTANCE** Escherichia fergusonii, a species closely related to Escherichia coli, is known to cause sporadic conditions in humans, including diarrhea. However, the critical virulence factors in *E. fergusonii* clinical isolates remain to be identified. This study shows the first isolation of an *E. fergusonii* strain carrying the *elt1* gene, which encodes heat-labile enterotoxin 1, from a patient with diarrhea. Our analysis of public databases also revealed the presence of *elt1*-positive *E. fergusonii* strains isolated from poultry in the United Kingdom. Interestingly, while the *elt1* gene in the poultry isolate was present on a large plasmid, in the human isolate it was integrated into the chromosome, which may confer stability on the *elt1*-carrying genetic element. Our findings highlight the need for extensive surveillance of *elt1*-positive *E. fergusonii* strains in livestock animals.

## OBSERVATION

Escherichia fergusonii is not a common inhabitant of human intestinal tracts, unlike its close relative, Escherichia coli. Although its virulence to humans has not been well elucidated, *E. fergusonii* strains have been isolated from various human infections, including those causing diarrhea (1, 2). *E. fergusonii* strains carrying genes for heat-labile enterotoxin (LT), which are hallmark virulence factors of enterotoxigenic E. coli (ETEC), a pathogen enteric for humans and animals, were also isolated from healthy chickens and a sick pig (3, 4). While LT is antigenically divided into subtypes LT1 and LT2, most human ETEC isolates produce LT1 (5). As the *elt* genes of the LT-positive *E. fergusonii* chicken isolates encode LT1, these isolates are thought to have the potential to cause human diseases (4). However, the isolation of LT-producing *E. fergusonii* strains from humans has not yet been reported. Moreover, there is no information on the virulence factors in the above-mentioned *E. fergusonii* strains from human patients (1). Here, we report the isolation of an LT1-positive *E. fergusonii* strain from an adult patient and describe its genomic features revealed by the determination of its complete genome sequence and genomic comparison with publicly available *E. fergusonii* genomes. The LT1-mediated cytotoxicity of the strain on Chinese hamster ovary (CHO) cells is also shown.

A bacterial strain was isolated using DHL agar (Eiken Kagaku, Japan) from a stool specimen from a 38-year-old male patient in a hospital in Miyazaki, Japan, in 2014. Through routine microbiological tests, in this strain (named strain 30038), the *elt1* gene was identified by PCR using *elt1* primers (6) and LT1 production was confirmed by a latex agglutination test (VET-RPLA; Denka Seiken, Tokyo, Japan). Strain 30038 was initially thought to be ETEC but was ultimately identified as *E. fergusonii* based on its biochemical features (see Table S1 in the supplemental material). Although the detailed clinical records of the patient were not available, the strain was most likely a causative agent of the patient’s intestinal infection.

For genomic characterization, we determined the complete genome sequence of strain 30038 by hybrid assembly of Illumina short-read sequences and Oxford Nanopore Technologies (ONT) long-read sequences using Unicycler (7) (see the methods in the supplemental material for more details). The genome comprised a 4,687,599-bp chromosome and four plasmids (234,318 bp, 109,686 bp, 3,240 bp, and 1,506 bp in length) (Fig. S1 and S2). Unexpectedly, although known *elt1* genes are exclusively carried on large plasmids (5), the *elt1* gene was found on the chromosome in strain 30038.

**FIG 1:**
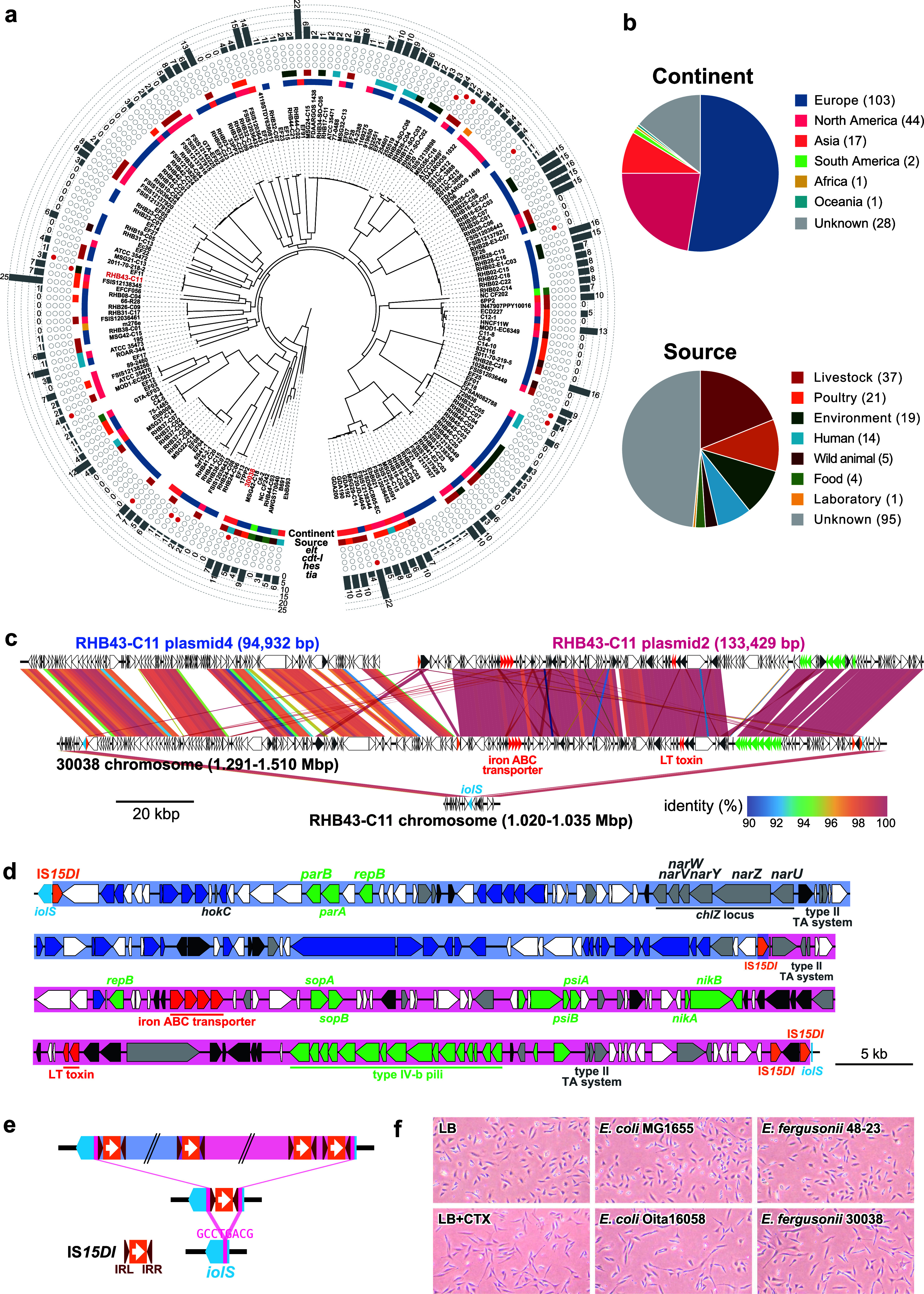
Phylogenetic position of strain 30038 in *E. fergusonii* and the LT1-encoding element and CHO cell elongation activity of strain 30038. (a) Core gene-based phylogenetic tree of 196 *E. fergusonii* strains (strain 300838 sequenced in this study and 195 strains whose genome sequences were publicly available). Two *elt1*-positive strains (30038 and RHB43-C11) are indicated in red. Colored rectangles around the tree indicate the geographic information (continents) and isolation sources of each strain. The same colors as those in the pie charts (b) are used. The distribution of four virulence genes (*elt*, *cdt-IA*, *hes*, and *tia*) in the 196 *E. fergusonii* strains (red circles) and the number of AMR genes possessed by each strain are also shown. (b) Pie charts summarizing the geographic information (continents) and isolation sources of 196 *E. fergusonii* strains. (c) Genomic comparison of the LT1-encoding elements of strains 30038 and RHB43-C11. Genes involved in virulence are indicated in red. Homologous regions are indicated by shading, and the sequence identities (BLASTN search) are indicated by different colors. The insertion site (the *iolS* gene) of the LT1-encoding element of strain 30038 is indicated in light blue. (d) Enlarged view of the genetic structure of the LT1-encoding region in strain 30038. Genes for virulence, phage production, plasmid maintenance, transposases (except for IS*15DI*), other functions, and unknown functions are indicated in red, blue, light green, gray, dark gray, and white, respectively. IS*15DI* and the *iolS* gene are indicated in orange and light blue, respectively. The RHB43-C11 plasmid 4-like region and the plasmid 2-like region are highlighted in blue and red, respectively. (e) Possible process of the insertion of a fused plasmid via IS*15DI*-mediated recombination into the *iolS* gene in strain 30038. Direct repeats (8 bp) generated upon IS insertion are indicated in pink. The *iolS* gene and its fragments are indicated in light blue. (f) Results of the CHO cell elongation assay. CHO cells (2 × 10^5^ cells/well/500 μL) in a 24-well plate were treated with 100-fold-diluted bacterial cell lysates for 48 h at 37°C and observed by light microscopy (×100). Cell lysates were prepared by sonicating bacterial cultures grown in lysogeny broth overnight at 37°C. The cell lysate of an ETEC O6:HNT strain (*elt1* positive) and the purified cholera toxin were used as positive controls, and two *elt*-negative strains (E. coli K-12 MG1655 and *E. fergusonii* 48-23) were used as negative controls. LB, CHO cells treated with lysogeny broth.

To analyze the phylogenetic position of strain 30038 in *E. fergusonii*, we collected 195 *E. fergusonii* genomes available in the NCBI and EnteroBase (8) databases and constructed a core gene-based phylogenomic tree with strain 30038 (Fig. 1a and Table S2; see also methods in the supplemental material for more details). Among the 196 strains analyzed, 37 (18.8%) were isolated from livestock, 21 (10.7%) were from poultry, and 14 (7.1%, including strain 30038) were from humans; most were isolated in Europe (52.6% [*n* = 103]) and North America (20.9% [*n* = 41]) (Fig. 1b). No apparent bias in geographic distribution or isolation source was observed (Fig. 1a).

A search of the 196 genomes for virulence genes using an in-house virulence gene database (supplemental material), which included genes for the major E. coli virulence factors and known ETEC colonization factors (CFs) (see the methods in the supplemental material for more details), revealed that among the genes analyzed, strain 30038 contained only *elt1* (Fig. 1a). In the other strains, *elt1* was detected only in a strain distantly related to strain 30038 (strain RHB43-C11), which was isolated from a pooled fecal sample from a poultry farm in the United Kingdom (9). Although various CFs have been identified in human and animal ETEC strains (10), no known CFs were detected in either *elt1*-positive strain. However, the two *elt1*-positive *E. fergusonii* strains may possess previously unidentified CFs because 20% to 40% of ETEC clinical isolates contain no detectable CFs, and the presence of previously unidentified CFs in these isolates has been suspected (10). Although several *E. fergusonii* strains contained *cdt*-IA (cytolethal distending toxin), *hes* (hemagglutinin), or *tia* (invasion) from livestock (*n* = 3) or unknown samples (*n* = 8), the contribution of these potential virulence factors to human diseases remains unclear (11–13).

A search for acquired antimicrobial resistance (AMR) genes using the ARG-ANNOT database (14) revealed that strain 30038 contained 11 genes that confer resistance to six drug classes. Thus, this strain is genetically regarded as a multidrug-resistant (MDR) strain. These genes were all carried on plasmid 1 (Fig. 1a and Fig. S2), which also contained the tellurium-resistant and mercury-resistant operons. Many other *E. fergusonii* strains (>70%) also carried at least one AMR gene, and 17 strains carried more than 10 genes (up to 25 genes) (Fig. 1a). In previous studies, *E. fergusonii* has been observed to be prevalent among livestock animals with a substantial degree of resistance to various antimicrobials, including tigecycline, colistin, and carbapenem, within China (15–19). Consequently, this bacterium could be regarded as a significant reservoir of AMR genes.

As both Illumina read and ONT long-read data were available for strain RHB43-C11, we determined the complete genome sequence of strain RHB43-C11 by hybrid assembly to compare its *elt1*-bearing genomic region with that of strain 30038. The genome of RHB43-C11 comprised a 4,534,336-bp chromosome and six plasmids (227,114 bp, 133,429 bp, 95,369 bp, 94,932 bp, 45,971 bp, and 4,593 bp in length), and *elt1* was found on a plasmid (plasmid 2) (Fig. 1c), as seen in most ETEC strains (5). However, this plasmid did not show homology to any genomes of ETEC virulence plasmids available in the NCBI database. Instead, it showed high sequence similarity to the LT1-bearing chromosomal region of strain 30038, with several small structural variations (Fig. 1c and d). Moreover, the chromosome region of strain 30038 adjacent to the LT1-encoding region showed a high similarity to plasmid 4 of strain RHB43-C11 (Fig. 1c and d). These results and the comparison of this chromosome region of strain 30038 with the analogous region of strain RHB43-C11 indicate that the LT1-encoding region of strain 30038 is a composite plasmid that was generated by the fusion of plasmids very similar to plasmids 2 and 4 of RHB43-C11 and inserted into the *iolS* gene of strain 30038. The presence of IS*15DI* in the plasmid-plasmid and chromosome-plasmid junctions suggested that insertion sequence (IS)-mediated recombination involved both genetic events (Fig. 1c to e). The integrations of these two plasmid-like sequences were confirmed by PCR using primers located on the flanking regions of the integration sites (Fig. S3). The predominant observed state was the integrated form, as opposed to the circular form. Integrations of virulence plasmids into the chromosome by homologous recombination have also previously been reported for enteroinvasive E. coli and Shigella flexneri, and the plasmid integrations into chromosome have been proposed to be one of the strategies to ensure plasmid maintenance (20). It is hypothesized that the integration of the *elt1*-carrying plasmid into the chromosome in strain 30038 also confers stability. Accordingly, the two strains share not only *elt1* but also genes for an iron utilization system and type IV-b pili homologous to those on plasmid R64. Type IV-b pili may be involved in the horizontal transfer of LT1-encoding elements in these *E. fergusonii* strains.

We finally analyzed the production of LT1 toxin by strain 30038 using the CHO cell elongation assay (21). CHO cells (CHO-K1 cell line from Riken Cell Bank) treated with the culture supernatant of strain 30038 showed clear elongation, comparable to the cells treated with purified cholera toxin and the culture supernatant of a typical ETEC strain (Oita16058), confirming that strain 30038 produces active LT1 toxin (Fig. 1f).

In conclusion, we isolated an LT1-producing *E. fergusonii* strain named strain 30038 from the stool of an adult patient in Japan. In strain 30038, the *elt1* gene was determined to be located on a plasmid that has been integrated into the chromosome. Additionally, a multidrug-resistant plasmid carrying 11 AMR genes was also identified in strain 30038. Given our findings, it is crucial to closely monitor the emergence and dissemination of multidrug-resistant LT1-producing strains of *E. fergusonii* within both human and animal populations. Such strains have the potential to contribute to the prevalence of diarrheal diseases. Moreover, further analyses of virulence mechanisms and the clinical impact of LT1-producing *E. fergusonii* may provide insights into their potential threat to public health.

### Data availability.

All sequence data generated in this study are available in the DDBJ/EMBL/GenBank BioProject under accession number PRJDB13909.
