# Supplementary material for: Isolation and Genomic Characterization of a Heat-Labile Enterotoxin 1-Producing Escherichia fergusonii Strain from a Human
Source: Microbiol Spectr. 2023 Jul 11;11(4):e00491-23. doi: 10.1128/spectrum.00491-23 (PMC10434266; doi:10.1128/spectrum.00491-23)
Supplement: Supplemental file 1 — Supplemental material. Download spectrum.00491-23-s0001.pdf, PDF file, 3.2 MB [file spectrum.00491-23-s0001.pdf]

Supplemental materials

Supplemental methods

Genome sequencing and assembly of strain 30038

Genomic DNA was purified using the Genomic- tip 100/G and Genomic DNA buffer set (Qiagen) according to the manufacturer's instructions. For short-read sequencing, libraries were prepared using the NEBNext Ultra II FS DNA Library Preparation Kit (New England Biolabs, MA, USA) and sequenced on the Illumina MiSeq platform to generate paired-end sequence reads (301 bp x 2). Low-quality and adaptor sequences in the reads were trimmed using Platanus_trim v1.0.7 (http://platanus.bio.titech.ac.jp/pltanus_trim) with the default parameters. For long-read sequencing, libraries were prepared using the Rapid Barcoding Kit (SQK-RBK004) and sequenced on the Oxford Nanopore Technologies (ONT) MinION platform using the R9.4.1 flow cell. Base calling was performed using Guppy GPU ver. 3.4.5 (ONT).

Using the Nanopore and Illumina data, *de novo* assembly was performed by Unicycler 0.5.0 with the default parameters (1). Circular maps were visualized using Circos 0.69-8 (2).

Collection and assembly of public sequence data

We first obtained 54 *E. fergusonii* genomes with the assembly level of the complete genome or chromosome in the NCBI RefSeq database. In addition, Illumina reads of 174 strains that were registered as *E. fergusonii* in the NCBI BioSample or identified as *E. fergusonii* by EnteroBase (3) were also obtained from the NCBI SRA database. For Illumina reads, after the trimming of low-quality and adaptor sequences using Platanus_trim v1.0.7 (http://platanus.bio.titech.ac.jp/pltanus_trim) with the default parameters, *de novo* assemblies were conducted using Platanus_B v1.3.2 with the default parameters (4). Assembled genomes judged to be low-quality by checkM (5) (cutoff values of <95% completeness or >10% contamination) were excluded. We further excluded genomes that were not assigned to be *E. fergusonii* by fastANI (6) with a cutoff value of < 95% average nucleotide identity to the *E. fergusonii* type strain (ATCC 35469^{T}). Finally, 196 strains, including 30038, were used for further analyses. Gene annotation for each genome was performed using prokka v1.14.5 (7).

Phylogenetic analysis

Pangenome analysis was conducted using Roary v. 3.13.0 with the following parameters: -i 90 -e -n (8). Based on the sequences of the core genes identified by Roary, phylogenetic analysis was performed using RaxML-NG ver. 1.0.1 with the following parameters: --all, --bs-trees 100, --model GTR+G4 (9). The tree was rooted using *E. coli* MG1655 and visualized using iTol (10).

Identification of virulence and antimicrobial resistance (AMR) genes

To identify virulence genes, a blastn search (v2.12.0) (11) was conducted against the draft genome sequences using an in-house virulence gene database including genes for the major *E. coli* virulence factors and all the known ETEC colonization factors as query sequences with the following parameters: -perc_identity 90 -qcov_hsp_perc 80. To identify AMR genes, we conducted the ABRicate pipeline (https://github.com/tseemann/abricate) with the '--minid 95' option against the ARG-ANNOT database (12).

CHO cell elongation assay

CHO cells (8×10^4 cells/2 mL in DMEM containing 1% FCS) in a 6-well plate were treated with 100-fold diluted bacterial cell lysate at 37°C for 28 hours and observed under an optical microscope (×100). The cell lysates were prepared by sonication (30 sec on/30 sec off, low setting, in ice water) of overnight bacterial culture in Lysogeny broth (LB) for 4 min with Bioruptor II (BMBio, Tokyo, Japan) and passing through a 0.22 µm syringe filter. A 100 ng/ml cholera toxin solution (Fujifilm Wako, Osaka, Japan) was used as a positive control.

References

1. Wick RR, Judd LM, Gorrie CL, Holt KE. Unicycler: Resolving bacterial genome assemblies from short and long sequencing reads. PLoS Comput Biol. 2017 Jun 8;13(6):e1005595-.
2. Krzywinski M, Schein J, Birol I, Connors J, Gascoyne R, Horsman D, et al. Circos: An information aesthetic for comparative genomics. Genome Res. 2009 Sep;19(9):1639–45.
3. Zhou Z, Alikhan NF, Mohamed K, Fan Y, Achtman M. The EnteroBase user's guide, with case studies on *Salmonella* transmissions, *Yersinia pestis* phylogeny, and *Escherichia* core genomic diversity. Genome Res. 2020;30(1):138–52.
4. Kajitani R, Yoshimura D, Ogura Y, Gotoh Y, Hayashi T, Itoh T. Platanus_B: an accurate *de novo* assembler for bacterial genomes using an iterative error-removal process. DNA Research. 2020 Jun 1;27(3):dsaa014.
5. Parks DH, Imelfort M, Skennerton CT, Hugenholtz P, Tyson GW. CheckM: Assessing the quality of microbial genomes recovered from isolates, single cells, and metagenomes. Genome Res. 2015 Jul 1;25(7):1043–55.
6. Jain C, Rodriguez-R LM, Phillippy AM, Konstantinidis KT, Aluru S. High throughput ANI analysis of 90K prokaryotic genomes reveals clear species boundaries. Nat Commun. 2018;9(1):5114.

7. Seemann T. Prokka: rapid prokaryotic genome annotation. Bioinformatics. 2014 Jul 15;30(14):2068–9.
8. Page AJ, Cummins CA, Hunt M, Wong VK, Reuter S, Holden MTG, et al. Roary: rapid large-scale prokaryote pan genome analysis. Bioinformatics. 2015 Nov 15;31(22):3691–3.
9. Kozlov AM, Darriba D, Flouri T, Morel B, Stamatakis A. RAxML-NG: a fast, scalable and user-friendly tool for maximum likelihood phylogenetic inference. Bioinformatics. 2019 Nov 1;35(21):4453–5.
10. Letunic I, Bork P. Interactive Tree Of Life (iTOL) v5: an online tool for phylogenetic tree display and annotation. Nucleic Acids Res. 2021 Jul 2;49(W1):W293–6.
11. Altschul SF, Gish W, Miller W, Myers EW, Lipman DJ. Basic local alignment search tool. J Mol Biol. 1990;215(3):403–10.
12. Kumar GS, Roshan PB, M DS, Rafael LR, Marie K, Luce L, et al. ARG-ANNOT, a New Bioinformatic Tool To Discover Antibiotic Resistance Genes in Bacterial Genomes. Antimicrob Agents Chemother. 2014 Jan 1;58(1):212–20.

Supplemental Figures

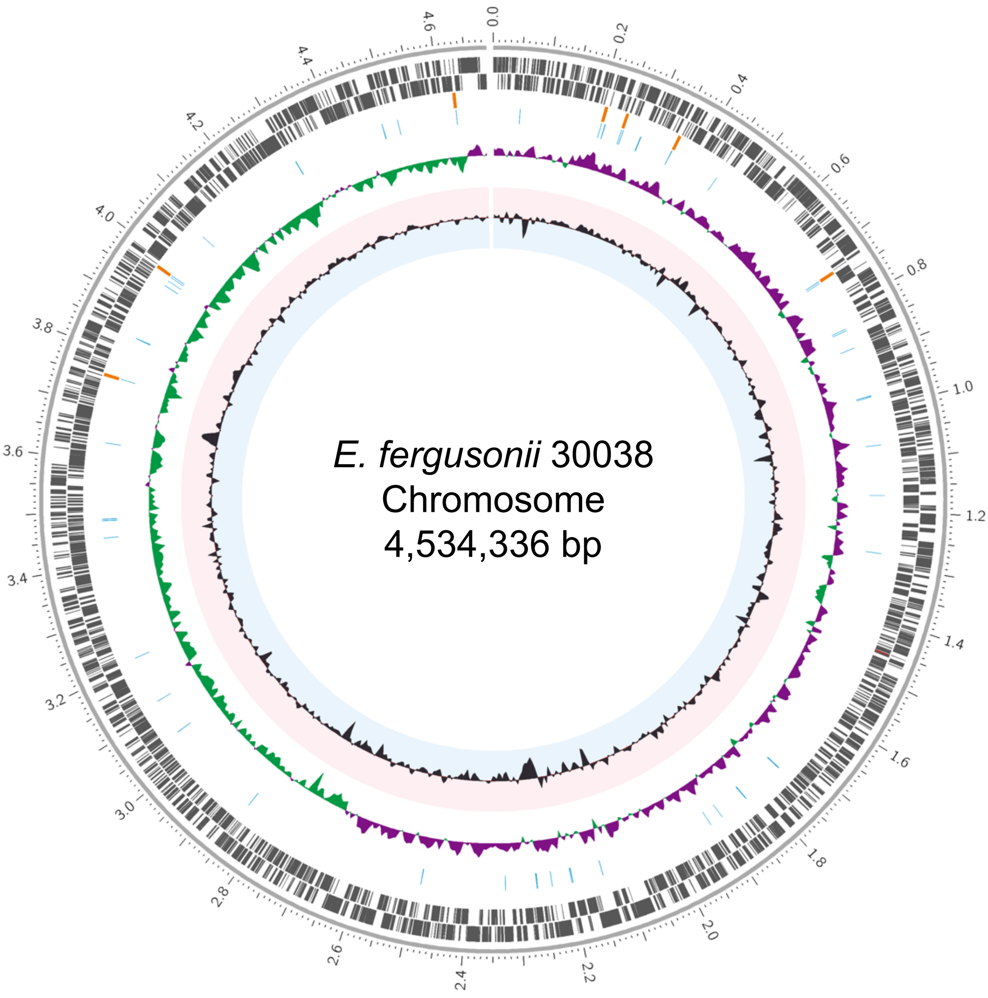

Figure S1. circular map of chromosome in the strain 30038

From the outside to the center: CDSs on forward strand, CDSs on reverse strand, rRNA genes, tRNA genes, the GC skew value, and the GC content.

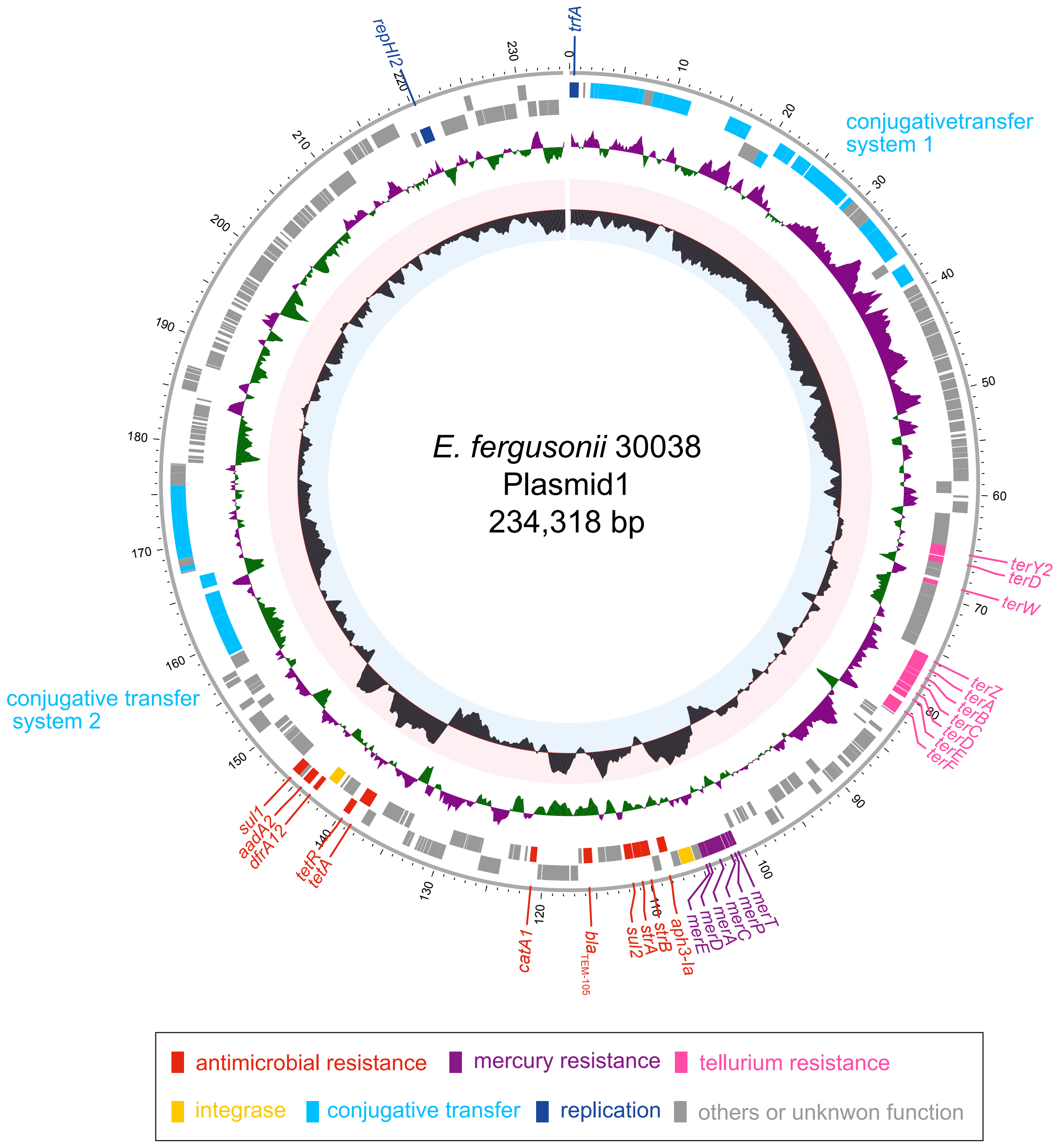

Figure S2. circular map of plasmid1 in the strain 30038
From the outside to the center: CDSs on forward strand, CDSs on reverse strand, the GC skew value, and the GC content.

a

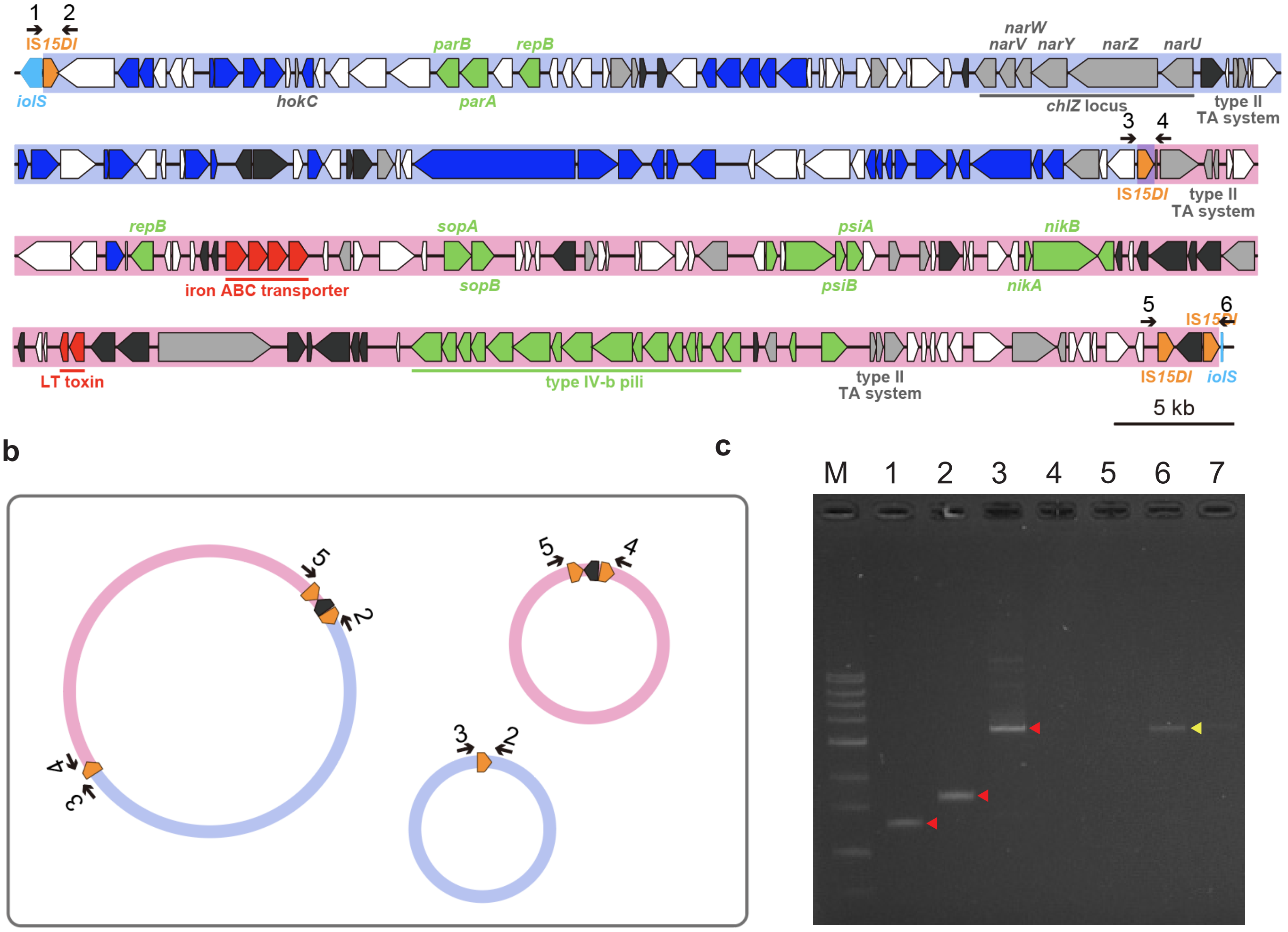

d

	forward primer	primer sequence	reverse primer	primer sequence	estimated size
lane1	primer 1	TAAGGCTTCACCGACCACTTC	primer 2	AAGCTCCGGTTAATAGCCACC	1266
lane2	primer 3	GCTGATACCCTCTGACACGAG	primer 4	ACTTGGCGAATTTGTGCTTCC	1586
lane3	primer 5	TCCGTCGTTCTGCTGTATGTC	primer 6	ATGGTCTGTTCCAGCATCCTG	3287
lane4	primer 2	AAGCTCCGGTTAATAGCCACC	primer 3	GCTGATACCCTCTGACACGAG	1545
lane5	primer 4	ACTTGGCGAATTTGTGCTTCC	primer 5	TCCGTCGTTCTGCTGTATGTC	3249
lane6	primer 2	AAGCTCCGGTTAATAGCCACC	primer 5	TCCGTCGTTCTGCTGTATGTC	3208
lane7	primer 1	TAAGGCTTCACCGACCACTTC	primer 6	ATGGTCTGTTCCAGCATCCTG	1337

Figure S3. PCR confirmation of the chromosomal integrtion of the *elt1*-encoding plasmid. **(a)** The genetic structure of the LT1-encoding region in *E. fergusonii* strain 30038. The primer locations are indicated by arrows. **(b)** Anticipated circular conformations of the LT1-encoding region in strain 30038. The primer locations are indicated by arrows. **(c)** PCR products amplified using seven distinct combinations of the primers (refer to panel d). Lane M: Size marker, lane 1 to 7: PCR amplicon. The bands corresponding to the integrated and circular forms of the *elt1*-encoding region are identified by red and yellow arrowheads, respectively. **(d)** Primer sequences and combinations employed for the PCR analysis.

Table S1. Biochemical features of *E. fergusonii* strain 30038

	**30038**	***E. fergusonii****	***E. coli****
Indole	+	98	98
Motility	+	93	95
Urease	-	0	1
Citric acid (Simmons)	-	17	1
Acetic acid	+	96	90
Malonic acid	+	35	0
Voges-Proskauer	-	0	0
Lysine decarboxylase	+	95	90
Ornithine decarboxylase	+	100	65
Arginine dihydrolase	-	5	17
Lactose	-	0	95
Saccharose	-	0	50
Adonitol	+	98	5
Arabinose	+	98	99
Cellobiose	+	96	2
Inositol	-	0	1
Maltose	+	96	95
Mannitol	+	98	98
Rhamnose	+	92	80
Salicin	-	65	40
Sorbitol	-	0	94
Trehalose	+	96	98
Xylose	+	96	95

*Percentage of positive reactions in *E. fergusonii* and *E. coli* as listed by Patrick et al. [Patrick R. Murray, Ellen Jo Baron, James H. Jorgensen, Marie Louise Landry, and Michael A. Pfaller:Manual of Clinical Microbiology, 9th Edition(2007)]

Table S2. Characteristics of strains used in this study

accession	strain name	description in NCBI BioSample	source	source niche	country	continent	assembly stats.		checkM		ANI	
							num. of scaffold	total length (bp)	Completeness	Contamination	*E. coli* ATCC 11775T	*E. fergusonii* ATCC35469 T
GCF_013928365.1	RHB18-C08	Escherichia fergusonii			United Kingdom		3	4,546,923	96.8	0.3	90.38	98.48
GCF_013713585.1	RHB03-C23	Escherichia fergusonii			United Kingdom		12	4,864,188	99.6	0.2	90.31	98.57
GCF_013714825.1	RHB02-C15	Escherichia fergusonii			United Kingdom		12	4,950,688	99.6	0.2	90.44	98.61
GCF_013714535.1	RHB02-C18	Escherichia fergusonii			United Kingdom		10	4,950,713	99.6	0.2	90.44	98.61
GCF_013715025.1	RHB02-C14	Escherichia fergusonii			United Kingdom		11	4,950,337	99.6	0.2	90.38	98.60
GCF_013710195.1	RHB18-C03	Escherichia fergusonii			United Kingdom		6	4,690,828	99.6	0.3	90.27	98.50
GCF_000191665.1	ECD227	Escherichia fergusonii ECD227	Gallus gallus domesticus		Canada		6	4,866,106	99.6	0.3	90.41	98.55
GCF_013892435.1	RHB19-C05	Escherichia fergusonii			United Kingdom		3	4,784,444	99.6	0.7	90.26	98.58
GCF_020097475.1	FDAARGOS_1499	Escherichia fergusonii	missing		Germany	Europe	2	4,645,928	99.7	0.2	90.26	99.99
GCF_000026225.1	ERS610163	Escherichia fergusonii ATCC 35469					2	4,643,861	99.6	0.2	90.14	100.00
GCF_020002305.1	5zf15-2-1	Escherichia fergusonii	pig		China		5	5,143,724	99.6	1.5	90.42	98.15
GCF_013812875.1	RHB38-C01	Escherichia fergusonii			United Kingdom		5	4,849,536	99.6	0.4	90.35	98.56
GCF_013813205.1	RHB38-C07	Escherichia fergusonii			United Kingdom		6	4,856,194	99.7	0.4	90.39	98.05
GCF_013819325.1	RHB33-C04	Escherichia fergusonii			United Kingdom		5	5,004,593	99.4	0.3	90.44	98.65
GCF_013819085.1	RHB33-C07	Escherichia fergusonii			United Kingdom		5	5,004,548	99.4	0.3	90.40	98.63
GCF_013923185.1	RHB02-C22	Escherichia fergusonii			United Kingdom		6	4,950,899	99.6	0.2	90.52	98.62
GCF_020002285.1	6S41-1	Escherichia fergusonii			China		7	4,977,581	99.4	0.2	90.42	98.17
GCF_013596835.1	RHB28-C13	Escherichia fergusonii			United Kingdom		6	5,152,230	99.7	0.4	90.59	98.62
GCF_013820845.1	RHB32-C05	Escherichia fergusonii			United Kingdom		4	5,053,207	99.6	0.3	90.26	98.58
GCF_013801425.1	RHB41-C20	Escherichia fergusonii			United Kingdom		6	4,909,259	99.7	0.4	90.47	98.10
GCF_013801845.1	RHB41-C15	Escherichia fergusonii			United Kingdom		6	4,909,259	99.7	0.4	90.52	98.09
GCF_008064875.1	ATCC_35473	Escherichia fergusonii	Homo sapiens		USA		1	4,657,720	99.3	0.2	90.24	98.48
GCF_013829265.1	RHB28-C21	Escherichia fergusonii			United Kingdom		7	5,058,232	99.7	0.5	90.43	98.51
GCF_008064895.1	ATCC_35471	Escherichia fergusonii	Homo sapiens		missing		3	4,755,297	99.6	0.2	90.18	98.68
GCF_013834525.1	RHB26-C03	Escherichia fergusonii			United Kingdom		2	4,730,448	99.6	0.3	90.29	98.54
GCF_013820225.1	RHB32-C16	Escherichia fergusonii			United Kingdom		5	4,802,523	99.6	0.2	90.27	98.50
GCF_013820465.1	RHB32-C09	Escherichia fergusonii			United Kingdom		5	4,802,520	99.6	0.2	90.27	98.50
GCF_013813425.1	RHB38-C04	Escherichia fergusonii			United Kingdom		3	4,622,715	99.6	0.2	90.27	98.49
GCF_013798785.1	RHB42-C24	Escherichia fergusonii			United Kingdom		4	4,719,140	99.6	0.2	90.20	98.60
GCF_013800985.1	RHB42-C01	Escherichia fergusonii			United Kingdom		4	4,719,138	99.6	0.2	90.17	98.60
GCF_013899845.1	RHB10-C04	Escherichia fergusonii			United Kingdom		4	4,718,187	99.6	0.2	90.29	98.60
GCF_013899685.1	RHB10-C23	Escherichia fergusonii			United Kingdom		4	4,718,001	99.6	0.2	90.45	98.59
GCF_013825725.1	RHB30-C07	Escherichia fergusonii			United Kingdom		2	4,876,117	99.7	0.4	90.42	98.63
GCF_016128235.1	FDAARGOS_1032	Escherichia fergusonii	missing	Laboratory	USA	North America	2	4,647,315	99.6	0.2	90.24	99.99
GCF_013171325.1	HNCF11W	Escherichia fergusonii	Gallus gallus		China		3	4,772,542	99.7	0.2	90.31	98.51
GCF_013819565.1	RHB32-C22	Escherichia fergusonii			United Kingdom		3	4,732,121	99.7	0.2	90.30	98.55
GCF_013822105.1	RHB31-C13	Escherichia fergusonii			United Kingdom		1	4,582,934	99.6	0.2	90.24	98.55
GCF_003944565.2	EFCF056	Escherichia fergusonii	chicken	Poultry	China	Asia	7	5,179,664	98.3	0.4	90.42	98.29
GCF_013836325.1	RHB25-C10	Escherichia fergusonii			United Kingdom		2	4,573,840	99.7	0.2	90.25	98.58
GCF_013893835.1	RHB18-C04	Escherichia fergusonii			United Kingdom		3	4,690,913	99.6	0.3	90.27	98.50
GCF_013591455.1	RHB41-C23	Escherichia fergusonii			United Kingdom		9	4,888,243	99.7	0.2	90.19	98.54
GCF_020883415.1	EF20JDJ4045	Escherichia fergusonii			China		6	5,125,452	99.5	0.5	90.37	98.48
GCF_013894595.1	RHB17-C11	Escherichia fergusonii			United Kingdom		1	4,549,644	99.6	0.2	90.09	98.69
GCF_013834165.1	RHB26-C09	Escherichia fergusonii			United Kingdom		1	4,549,178	99.6	0.3	90.37	98.57
GCF_013886195.1	RHB23-C02	Escherichia fergusonii			United Kingdom		4	4,814,303	99.6	0.2	90.24	98.54
GCF_013886335.1	RHB23-C01	Escherichia fergusonii			United Kingdom		5	4,855,788	99.6	0.2	90.24	98.54
GCF_019047545.1	FDAARGOS_1438	Escherichia fergusonii	missing		Germany	Europe	1	4,543,156	99.6	0.2	90.23	98.70
GCF_013799825.1	RHB42-C13	Escherichia fergusonii			United Kingdom		4	4,661,185	99.6	0.2	90.20	98.61
GCF_008064915.1	ATCC_35470	Escherichia fergusonii	missing		USA	North America	1	4,524,922	99.3	0.2	90.50	98.25
GCF_020105775.1	EF31	Escherichia fergusonii			China		3	4,788,203	99.3	0.4	90.33	98.58
GCF_013821535.1	RHB31-C17	Escherichia fergusonii			United Kingdom		2	4,573,194	99.6	0.3	90.41	98.64
GCF_013838265.1	RHB24-C08	Escherichia fergusonii			United Kingdom		1	4,457,616	99.7	0.2	90.32	98.14
GCF_013838465.1	RHB24-C06	Escherichia fergusonii			United Kingdom		1	4,457,616	99.7	0.2	90.32	98.14
GCF_020023425.1	EF91	Escherichia fergusonii			China		3	4,792,961	99.4	0.5	90.25	98.66
SRR17241369	FSIS12142225	Escherichia fergusonii		Livestock	USA	North America	157	4,726,602	99.5	0.2	90.31	98.49
SRR6413641	MOD1-EC5837	Escherichia fergusonii	Sus scrofa scrofa	Livestock	USA	North America	140	4,537,766	98.8	0.2	90.40	98.33
ERR7420978	MSG56-C16	Escherichia fergusonii	porcine	Livestock	United Kingdom	Europe	157	5,011,933	99.6	0.5	90.30	98.95
ERR7420865	MSG42-C18	Escherichia fergusonii	porcine	Livestock	United Kingdom	Europe	232	5,000,137	99.5	0.3	90.20	98.30
ERR7420863	MSG42-C15	Escherichia fergusonii	porcine	Livestock	United Kingdom	Europe	87	4,824,594	99.6	0.4	90.12	98.51
ERR7420817	MSG37-C14	Escherichia fergusonii	porcine	Livestock	United Kingdom	Europe	74	4,733,297	99.6	0.2	90.35	98.13
ERR7420767	MSG32-C13	Escherichia fergusonii	porcine	Livestock	United Kingdom	Europe	59	4,628,951	99.6	0.2	90.18	98.61
ERR7420743	MSG29-C14	Escherichia fergusonii	porcine	Livestock	United Kingdom	Europe	79	4,847,654	99.6	0.3	90.33	98.49
ERR7420721	MSG26-C15	Escherichia fergusonii	porcine	Livestock	United Kingdom	Europe	42	4,790,042	99.6	0.2	90.31	98.49
ERR7420690	MSG24-C15	Escherichia fergusonii	porcine	Livestock	United Kingdom	Europe	84	4,704,572	99.7	0.2	90.11	98.61
ERR7420671	MSG22-C14	Escherichia fergusonii	porcine	Livestock	United Kingdom	Europe	110	4,841,915	99.6	0.2	90.44	98.34
ERR7420662	MSG21-C13	Escherichia fergusonii	porcine	Livestock	United Kingdom	Europe	125	4,974,508	99.5	0.3	90.06	98.42
SRR15598555	C14-10	Escherichia fergusonii	Gallus gallus	Poultry	United Kingdom	Europe	54	4,403,018	99.7	0.2	90.34	98.63
SRR15598577	C12-1	Escherichia fergusonii	Gallus gallus	Poultry	United Kingdom	Europe	85	4,636,057	99.7	0.2	90.43	98.61
SRR15598599	C8-6	Escherichia fergusonii	Gallus gallus	Poultry	United Kingdom	Europe	105	4,421,375	99.7	0.2	90.24	98.64
SRR15598601	C8-4	Escherichia fergusonii	Gallus gallus	Poultry	United Kingdom	Europe	45	4,614,640	99.6	0.2	90.44	98.24
SRR15598614	C6-2	Escherichia fergusonii	Gallus gallus	Poultry	United Kingdom	Europe	79	4,692,125	99.6	0.5	90.19	98.79
SRR15598625	C4-5	Escherichia fergusonii	Gallus gallus	Poultry	United Kingdom	Europe	52	4,661,224	99.6	0.5	90.49	98.19
SRR15598580	C11-8	Escherichia fergusonii	Gallus gallus	Poultry	United Kingdom	Europe	66	4,405,379	99.7	0.2	90.29	98.63
ERR4451645	m276e	Escherichia fergusonii			Ethiopia	Africa	96	4,599,403	99.3	0.8	90.10	98.59
ERR4254571	B691	Escherichia fergusonii	Gymnorhina tibicen	Wild Animal	Australia	Oceania	166	4,925,643	99.7	0.6	90.24	98.57
ERR4250973	ROAR-344	Escherichia fergusonii	Homo sapiens	Human	France	Europe	99	4,680,637	99.6	0.2	90.26	98.58
ERR4019919	EbB093	Escherichia fergusonii	Homo sapiens	Human	Cambodia	Asia	428	4,578,735	98.9	0.5	90.17	98.68
ERR4019856	EbB021	Escherichia fergusonii	Homo sapiens	Human	Cambodia	Asia	391	4,459,230	99.5	0.6	90.32	98.78
ERR4019946	EbB008	Escherichia fergusonii	Homo sapiens	Human	Cambodia	Asia	112	4,649,176	99.2	0.2	90.41	98.35
ERR3437195	AWGS170040	Escherichia fergusonii		Food	Netherlands	Europe	130	4,968,054	99.7	0.4	90.23	98.55
ERR769194	2011-70-219-5	Escherichia fergusonii	Sus scrofa domesticus		Denmark		88	4,694,682	99.5	0.3	90.34	98.50

ERR769191	2011-70-218-2	Escherichia fergusonii	Sus scrofa domesticus		Denmark		131	4,982,268	99.5	0.7	90.20	98.41
ERR273809	EF28	Escherichia fergusonii					70	4,616,853	99.6	0.2	90.19	98.69
ERR273808	EF27	Escherichia fergusonii					57	4,630,100	99.6	0.3	90.05	98.59
ERR273807	EF26	Escherichia fergusonii					39	4,563,694	99.4	0.2	90.33	98.67
ERR273806	EF25	Escherichia fergusonii					71	4,705,232	99.5	0.3	90.21	98.55
ERR273805	EF24	Escherichia fergusonii					58	4,692,609	99.7	0.2	90.10	98.74
ERR273804	EF23	Escherichia fergusonii					76	4,716,573	99.6	0.2	90.21	98.66
ERR273803	EF22	Escherichia fergusonii					53	4,612,533	99.7	0.2	90.19	98.58
ERR273802	EF21	Escherichia fergusonii					59	4,556,709	99.6	0.3	90.30	98.60
ERR273801	EF20	Escherichia fergusonii					72	4,887,364	99.5	0.2	90.33	99.02
ERR273800	EF19	Escherichia fergusonii					109	4,929,872	99.7	0.2	90.03	98.56
ERR273799	EF18	Escherichia fergusonii					55	4,405,228	99.6	0.2	90.13	98.53
ERR273798	EF17	Escherichia fergusonii					53	4,710,870	99.3	0.2	90.14	98.58
ERR273797	EF15	Escherichia fergusonii					58	4,746,008	99.7	0.2	90.34	98.58
ERR273796	EF14	Escherichia fergusonii					65	4,634,668	99.6	0.3	90.13	98.54
ERR273795	EF13	Escherichia fergusonii					51	4,541,219	99.7	0.2	90.14	98.14
ERR273794	EF12	Escherichia fergusonii					115	4,959,423	99.7	0.5	90.56	98.21
ERR273793	EF11	Escherichia fergusonii					118	4,983,131	99.5	0.4	90.31	98.48
ERR273792	EF08	Escherichia fergusonii					122	5,138,129	99.6	0.5	90.41	98.29
ERR273791	EF07	Escherichia fergusonii					127	4,872,406	99.3	0.2	90.19	98.63
ERR273790	EF06	Escherichia fergusonii					50	4,671,128	99.7	0.2	90.30	98.62
ERR273789	EF05	Escherichia fergusonii					155	5,163,840	99.7	0.4	90.33	98.45
ERR273788	EF04	Escherichia fergusonii					107	4,989,763	99.7	0.5	90.33	98.36
ERR273787	EF03	Escherichia fergusonii					76	4,532,132	99.6	0.4	90.42	98.29
ERR273786	EF01	Escherichia fergusonii					76	4,612,684	99.6	0.4	90.13	98.54
SRR15361753	832116	Escherichia fergusonii	NA	Wild Animal	United Kingdom	Europe	100	4,485,837	99.5	0.3	90.25	98.64
SRR15243798	FSIS12140591	Escherichia fergusonii		Poultry	USA	North America	292	5,031,782	99.6	0.6	90.45	98.53
SRR15089912	FSIS12139898	Escherichia fergusonii		Livestock	USA	North America	199	4,912,769	99.6	0.2	90.28	99.00
SRR12667821	RHB41-E2-C03	Escherichia fergusonii		Environment	United Kingdom	Europe	74	4,722,188	99.7	0.2	90.28	98.54
SRR12667841	RHB28-E3-C07	Escherichia fergusonii		Environment	United Kingdom	Europe	47	4,675,822	99.6	0.6	90.34	98.63
SRR12667846	RHB28-C16	Escherichia fergusonii		Environment	United Kingdom	Europe	99	5,134,067	99.7	0.4	90.32	98.62
SRR13165320	66-R28	Escherichia fergusonii	Gallus gallus	Poultry	Croatia	Europe	75	4,591,305	99.6	0.3	90.23	98.65
SRR14407954	1169275	Escherichia fergusonii	Homo sapiens	Human	United Kingdom	Europe	96	4,633,941	99.6	0.3	90.26	98.68
SRR13858488	FSIS12036432	Escherichia fergusonii		Poultry	USA	North America	230	4,947,228	99.7	0.8	90.07	98.61
SRR13858486	FSIS12036431	Escherichia fergusonii		Poultry	USA	North America	207	4,941,829	99.7	0.8	90.16	98.58
SRR13858310	FSIS12036433	Escherichia fergusonii		Livestock	USA	North America	98	4,560,272	99.7	0.8	90.39	98.21
SRR13858265	FSIS12036425	Escherichia fergusonii		Livestock	USA	North America	217	4,961,866	99.6	0.2	90.16	98.44
SRR13700261	FSIS12036461	Escherichia fergusonii		Livestock	USA	North America	154	4,544,581	99.6	0.5	90.23	98.62
SRR14038777	FSIS12036449	Escherichia fergusonii		Livestock	USA	North America	210	4,464,185	99.7	0.4	90.36	98.60
SRR14038774	FSIS12036452	Escherichia fergusonii		Poultry	USA	North America	342	4,841,972	99.5	0.5	90.23	98.59
SRR14038769	FSIS12036443	Escherichia fergusonii		Livestock	USA	North America	208	4,636,476	99.6	0.3	90.40	98.62
SRR14004334	FSIS12138348	Escherichia fergusonii		Livestock	USA	North America	163	4,565,953	99.7	0.2	90.26	98.63
SRR14004335	FSIS12138349	Escherichia fergusonii		Livestock	USA	North America	115	4,653,588	99.6	0.2	90.25	98.57
SRR14004330	FSIS12138344	Escherichia fergusonii		Poultry	USA	North America	122	4,871,301	99.5	0.3	90.11	98.54
SRR14004328	FSIS12138345	Escherichia fergusonii		Poultry	USA	North America	244	5,164,264	99.3	0.6	90.17	98.39
SRR14004275	FSIS12138266	Escherichia fergusonii		Poultry	USA	North America	213	4,965,555	99.4	0.3	90.28	98.58
SRR14004228	FSIS12137927	Escherichia fergusonii		Livestock	USA	North America	155	4,751,285	99.7	0.3	90.25	98.56
SRR14004261	FSIS12137928	Escherichia fergusonii		Livestock	USA	North America	157	4,745,722	99.7	0.4	90.42	98.55
SRR14004262	FSIS12138262	Escherichia fergusonii		Poultry	USA	North America	144	4,783,239	99.6	0.2	90.17	98.50
SRR14004211	FSIS12137919	Escherichia fergusonii		Livestock	USA	North America	259	4,962,675	99.6	0.5	90.43	98.40
SRR14004208	FSIS12137920	Escherichia fergusonii		Livestock	USA	North America	205	4,966,978	99.7	0.2	90.52	98.37
SRR14004209	FSIS12137921	Escherichia fergusonii		Livestock	USA	North America	123	4,595,253	99.6	0.6	90.33	98.64
SRR14004107	FSIS12137915	Escherichia fergusonii		Livestock	USA	North America	210	4,898,002	99.7	0.2	90.25	98.51
SRR14004109	FSIS12137916	Escherichia fergusonii		Livestock	USA	North America	192	4,892,515	99.7	0.2	90.34	98.51
SRR11772695	919650	Escherichia fergusonii	Homo sapiens	Human	United Kingdom	Europe	110	4,718,318	99.7	0.2	90.22	98.74
SRR11583701	932551	Escherichia fergusonii	Homo sapiens	Human	United Kingdom	Europe	123	4,717,122	99.7	0.2	90.19	98.64
SRR8878461	720636	Escherichia fergusonii	Homo sapiens	Human	United Kingdom	Europe	203	4,641,442	99.6	0.3	90.15	98.66
SRR6066431	GDA200	Escherichia fergusonii	swine	Livestock	China	Asia	139	4,962,574	99.4	1.3	90.20	98.45
SRR6066376	GDA192	Escherichia fergusonii	swine	Livestock	China	Asia	122	4,959,773	99.4	0.3	90.21	98.47
SRR6066179	GDA190	Escherichia fergusonii		Poultry	China	Asia	124	4,959,429	99.4	0.3	90.24	98.37
SRR6413564	MOD1-EC6349	Escherichia fergusonii	parrot	Wild Animal	USA	North America	225	4,426,390	99.7	0.2	90.35	98.71
SRR13092463	1028457	Escherichia fergusonii	NA	Wild Animal	United Kingdom	Europe	69	4,384,347	99.7	0.2	90.17	98.56
SRR11949359	RHB37-C09	Escherichia fergusonii			United Kingdom		82	4,648,866	99.4	0.2	90.14	98.36
SRR11949360	RHB37-C07	Escherichia fergusonii			United Kingdom		92	4,651,650	99.5	0.2	90.09	98.35
SRR11949361	RHB37-C04	Escherichia fergusonii			United Kingdom		82	4,648,892	99.5	0.2	90.11	98.36
SRR11949362	RHB37-C02	Escherichia fergusonii			United Kingdom		92	4,649,254	99.6	0.2	90.28	98.42
SRR11949363	RHB37-C01	Escherichia fergusonii			United Kingdom		93	4,652,582	99.5	0.2	90.18	98.39
SRR11948753	RHB32-C07	Escherichia fergusonii			United Kingdom		74	4,777,129	99.6	0.2	90.26	98.67
SRR11949031	RHB30-C06	Escherichia fergusonii			United Kingdom		70	4,855,775	99.7	0.4	90.20	98.60
SRR11949037	RHB30-C01	Escherichia fergusonii			United Kingdom		62	4,855,230	99.7	0.4	90.32	98.61

SRR11949409	RHB26-C04	Escherichia fergusonii			United Kingdom		55	4,532,893	99.6	0.2	90.45	99.08
SRR11949420	RHB25-C08	Escherichia fergusonii			United Kingdom		45	4,553,587	99.7	0.2	90.29	98.60
SRR11949436	RHB08-C04	Escherichia fergusonii			United Kingdom		78	4,877,152	99.6	0.2	90.20	98.46
SRR11949053	RHB03-C11	Escherichia fergusonii			United Kingdom		106	4,882,229	99.6	0.2	90.12	98.55
SRR6373706	2011C-4212	Escherichia fergusonii	missing		USA	North America	92	4,643,554	99.6	0.2	90.18	99.99
SRR6373719	2011C-4215	Escherichia fergusonii	missing		USA	North America	81	4,640,827	99.6	0.2	90.16	99.99
SRR5590335	89-2460	Escherichia fergusonii	missing		USA	North America	209	4,760,366	99.6	0.5	90.28	98.54
SRR1916804	GTA-EF02	Escherichia fergusonii			Canada		81	4,740,260	99.7	0.2	90.15	98.54
SRR1916749	GTA-EF03	Escherichia fergusonii		Food	Canada	North America	71	4,523,975	99.6	0.3	90.53	98.26
SRR2040173	77-0488	Escherichia fergusonii	missing		USA	North America	239	4,777,423	99.6	0.6	90.12	98.67
SRR2040166	75-1485	Escherichia fergusonii	missing		USA	North America	195	4,837,065	99.6	0.2	90.52	98.24
SRR2040145	2010C-3896	Escherichia fergusonii	missing		USA	North America	182	4,670,057	99.7	1.1	90.15	99.99
SRR2040144	2010C-3888	Escherichia fergusonii	missing		USA	North America	121	4,634,752	99.6	0.2	90.20	99.99
SRR2040142	ATCC_35472	Escherichia fergusonii	missing		USA	North America	101	4,450,164	99.7	0.2	90.17	98.60
ERR1622487	4119STDY6380815	Escherichia coli					70	4,651,107	99.3	0.3	90.39	98.50
ERR3531499	195	Escherichia coli		Livestock			109	4,867,726	98.2	0.4	90.19	98.55
ERR4220944	17PP2	Escherichia coli	Sus scrofa	Livestock	Thailand	Asia	71	4,572,044	99.6	0.5	90.17	98.19
ERR4220983	33PP2	Escherichia coli	Sus scrofa	Livestock	Thailand	Asia	118	4,808,804	99.7	0.4	90.16	98.53
ERR4221096	6PP2	Escherichia coli	Sus scrofa	Livestock	Thailand	Asia	117	4,867,812	99.4	0.4	90.17	98.61
SRR11653192	IN47907PPY10016	Escherichia coli	cattle	Livestock	USA	North America	198	4,575,270	99.7	0.8	90.13	98.71
SRR12183885	RHB17-SO-C03	Escherichia coli		Environment	United Kingdom	Europe	78	4,939,162	99.6	0.2	90.33	99.10
SRR12183886	RHB17-SO-C02	Escherichia coli		Environment	United Kingdom	Europe	108	4,943,348	99.6	0.2	90.13	99.11
SRR12183892	RHB16-E2-C07	Escherichia coli		Environment	United Kingdom	Europe	59	4,855,271	99.7	0.4	90.33	98.59
SRR12183894	RHB16-E2-C03	Escherichia coli		Environment	United Kingdom	Europe	59	4,855,365	99.7	0.4	90.33	98.59
SRR12183962	RHB46-C20	Escherichia coli		Environment	United Kingdom	Europe	106	4,589,065	99.5	0.2	90.29	98.58
SRR12183967	RHB46-C12	Escherichia coli		Environment	United Kingdom	Europe	108	4,589,261	99.5	0.2	90.39	98.64
SRR12183972	RHB46-C03	Escherichia coli		Environment	United Kingdom	Europe	103	4,585,755	99.5	0.2	90.30	98.63
SRR12183998	RHB45-C03	Escherichia coli		Environment	United Kingdom	Europe	111	4,587,799	99.5	0.2	90.23	98.58
SRR12183999	RHB45-C02	Escherichia coli		Environment	United Kingdom	Europe	105	4,588,662	99.5	0.2	90.21	98.58
SRR12184000	RHB44-SO-C08	Escherichia coli		Environment	United Kingdom	Europe	69	4,672,521	99.6	0.2	90.30	99.10
SRR12184002	RHB02-E1-C03	Escherichia coli		Environment	United Kingdom	Europe	91	4,925,925	99.6	0.2	90.37	98.62
SRR12184017	RHB44-C21	Escherichia coli		Environment	United Kingdom	Europe	86	4,799,679	99.7	0.8	89.99	98.60
SRR12184023	RHB44-C03	Escherichia coli		Environment	United Kingdom	Europe	113	4,834,722	99.6	0.3	90.53	98.57
SRR12184025	RHB44-C02	Escherichia coli		Environment	United Kingdom	Europe	75	4,770,881	99.7	0.5	90.03	98.56
SRR12184047	RHB43-C11	Escherichia coli		Environment	United Kingdom	Europe	139	5,129,101	99.5	0.6	90.30	98.44
SRR12184075	RHB34-SO-C05	Escherichia coli		Environment	United Kingdom	Europe	66	4,653,682	99.6	0.2	90.30	98.67
SRR13823704	Ec36	Escherichia coli	Larus michahellis	Wild Animal	France	Europe	124	4,975,570	99.7	1.1	90.33	98.61
SRR14766731	21SD04CB05-EC	Escherichia coli		Poultry	USA	North America	80	4,905,609	99.7	0.4	90.14	98.58
SRR4897566	254601	Shigella sp. 254601	Homo sapiens	Human	United Kingdom	Europe	71	4,618,357	99.6	0.7	90.15	98.69
SRR5024274	255519	Shigella sp. 255519	Homo sapiens	Human	United Kingdom	Europe	70	4,614,012	99.6	0.5	90.12	98.72
SRR6186591	74-2388	Escherichia coli	missing		USA	North America	60	4,594,239	99.6	0.2	90.08	98.68
SRR7297675	NC_CF202	Escherichia coli		Food	Brazil	South America	200	5,027,456	99.2	0.4	90.19	98.64
SRR7299272	NC_CF242	Escherichia coli		Food	Brazil	South America	231	4,894,853	99.7	0.4	90.32	98.63
SRR7799232	CFSAN082788	Escherichia coli O157:H7	Homo sapiens	Human	USA	North America	192	4,823,932	99.6	0.8	90.30	98.38

Supplemental text

Major virulence genes
>eaeA_alpha1_AF022236
ATGATTACTCATGGTTTTTATGCCCGGACCCGGCACAAGCATAAGCTAAAAAAAACATTT
ATTATGCTTAGTGCTGGTTTAGGATTGTTTTTTATGTTAATCAGAATTCATTTGCAAAT
GGTGAAAATTATTTTAAATTGGGTTCGGATTCAAAACTGTTAACTCATAATAGCTATCAG
AATCGCCTTTTTTATACGTTGAAAACAGGTGAAACTGTTGCCGATCTTTCTAAATCGCAA
GATATTAATTTATCGACGATTTGGTCGTTGAATAAGCATTTATACAGTTCTGAAAGCGAA
ATGATGAAGGCCGAGCCTGGTCAGCAGATCATTTTGCCACTCAAAAAACTTCCCTTTGAA
TACAGTGCCTTACCACTTTTAGGTTCGGCACCTCTTGTTGCTGCAGGTGGTGTCGCTGGT
CATACAAATAAACTGACTAAAATGTCCCCGGACGTGACCAAAAGCAACATGACCGATGAC
AAGGCATTAAATTATGCGGCACAACAGGCGGCGAGTCTCGGTAGCCAGCTTCAGTCGCGA
TCTCTGAACGGCGATTACGCGAAAGATACCGCTCTTGGTATCGCTGGTAACCAGGCTTCG
TCACAGTTGCAGGCCTGGTTACAACATTATGGAACGGCAGAGGTTAATCTGCAGAGTGGT
AATAACTTTGACGGTAGTTCACTGGACTTCTTATTACCGTTCTATGATTCCGAAAAAATG
CTGGCATTTGGTCAGGTCGGAGCGCGTTACATTGACTCCCGCTTTACGGCAAATTTAGGT
GCGGGTCAGCGTTTTTTCCTTCCTGAAAATATGTTGGGCTATAACGTCTTCATTGATCAG
GATTTTTCTGGTGATAATACCCGTTTAGGTATTGGTGGCGAATACTGGCGAGACTATTTC
AAAAGTAGTGTTAACGGCTATTTCCGCATGAGCGGCTGGCATGAGTCATACAATAAGAAA
GACTATGATGAGCGCCCAGCAAATGGCTTCGATATCCGTTTTAATGGCTATCTGCCATCA
TACCCGGCATTAGGTGCCAAGCTGATGTATGAGCAGTATTATGGTGATAATGTTGCTTTG
TTTAATTCTGATAAGCTGCAGTCGAATCCTGGTGCGGCGACCGTTGGTGTAAACTATACT
CCGATTCCTCTGGTGACGATGGGGATCGATTACCGTCATGGTACGGGTAATGAAAATGAT
CTCCTTTACTCAATGCAGTTCCGTTATCAGTTTGATAAACCGTGGTCTCAGCAAATTGAG
CCACAATATGTTAACGAGTTAAGAACATTATCAGGCAGCCGTTACGATCTGGTTCAGCGT
AATAACAATATTATTCTGGAGTACAAAAAGCAGGATATTCTTTCTCTGAATATTCCGCAT
GATATTAATGGTACTGAACGCAGTACGCAGAAGATTCAATTGATCGTTAAGAGCAAATAC
GGTCTGGATCGTATCGTCTGGGATGATAGTGCATTACGTAGCCAGGGCGGCCAGATTCAG
CATAGCGGAAGCCAAAGCGCACAAGATTACCAGGCTATTTTGCCTGCTTATGTGCAAGGT
GGTAGCAATGTTTATAAAGTGACGGCTCGCGCCTATGACCGTAATGGCAATAGCTCTAAC
AATGTACTGCTTACTATTACCGTTCTGTCGAATGGTCAGGTGGTCGACCAGGTTGGGGTA
ACGGACTTTACGGCTGATAAGACTTCGGCTAAAGCGGATGGCACCGAAGCAATTACTTAT
ACTGCGACGGTGAAAAAGAATGGGGTAGCTCAGGCTAATGTCCCTGTTTCATTTAATATT
GTTTCAGGAACTGCAGTTTTAAGTGCCAATAGTGCCAATACCAATGGTAGCGGTAAGGCG
ACTGTAACCCTGAAATCGGATAAACCAGGCCAGGTCGTCGTGTCTGCTAAAACAGCAGAG
ATGACTTCAGCGCTTAATGCCAATGCAGTTATATTTGTTGATCAAACCAAGGCCAGCATT
ACTGAGATTAAGGCTGATAAAACAACGGCAGTAGCAAATGGTCAGGATGCTATTACATAC
ACTGTTAAAGTGATGAAGGGGGATAAGCCTGTATCTAATCAGGAAGTGACCTTTACGACG
ACCTTAGGTAAGTTAAGTAATTCCACTGAAAAAACGGATACGAATGGCTATGCCAAAGTA
ACATTAACATCGACAACTCCAGGAAAATCACTCGTTAGTGCCCGTGTTAGCGATGTCGCC
GTTGATGTCAAAGCACCTGAAGTTGAATTTTTTACAACGCTTACAATTGATGACGGTAAT
ATTGAAATTGTTGGAACCGGAGTTAAAGGGAAGTTACCCACTGTATGGTTGCAATATGGT
CAAGTTAATCTGAAAGCCAGCGGAGGTAACGGAAAATATACATGGCGCTCAGCAAATCCA
GCAATTGCTTCGGTGGATGCTTCTTCTGGTCAGGTCACCTTAAAAGAGAAGGGAACTACA
ACTATTTCCGTTATCTCAAGTGATAATCAAACTGCAACTTATACTATTGCAACACCTAAT
AGTCTGATTGTTCCTAATATGAGCAAGCGTGTGACCTATAATGATGCTGTGAATACATGT
AAGAATTTTGGAGGAAAGTTGCCGTCTTCTCAGAATGAACTGGAAAATGTCTTTAAAGCA
TGGGGGGCTGCAAATAAATATGAATATTATAAGTCTAGTCAGACTATAATTTCATGGGTA
CAACAAACAGCTCAAGATGCGAAGAGTGGTGTTGCAAGTACATACGATTTAGTTAAACAA
AACCCTCTGAATAATATTAAGGCTAGTGAATCTAATGCTTATGCCACTTGTGTAAAATAA
>eaeA_alpha2_DQ523600
ATGATTACTCATGGTTGTTATACCCGGACCCGGCACAAGCATAAGCTAAAAAAAACATTG
ATTATGCTTAGTGCTGGTTTAGGATTGTTTTTTATGTTAATCAGAATTCATTTGCAAAT
GGTGAAAATTATTTTAAATTGGGTTCGGATTCAAAACTGTTAACTCATGATAGCTATCAG
AATCGCCTTTTTTATACGTTGAAAACAGGTGAAACTGTTGCCGATCTTTCTAAATCACAA
GATATTAATTTATCGACGATTTGGTCGTTGAATAAGCATTTATACAGTTCTGAAAGCGAA

ATGATGAAGGCCGCGCCTGGTCAGCAGATCATTTTGCCACTCAAAAAACTTCCCTTTGAA
TACAGTGCACTACCACTTTTAGGTTCGGCACCTCTTGTTGCTGCAGGTGGTGTTGCTGGT
CACACGAATAAACTGACTAAAATGTCCCCGGACGTGACCAAAAGCAACATGACCGATGAC
AAGGCATTAAATTATGCGGCACAACAGGCGGCGAGTCTCGGTAGCCAGCTTCAGTCGCGA
TCTCTGAACGGCGATTACGCGAAAGATACCGCTCTTGGTATCGCTGGTAACCAGGCTTCG
TCACAGTTGCAGGCCTGGTTACAACATTATGGAACGGCAGAGGTTAATCTGCAGAGTGGT
AATAACTTTGACGGTAGTTCACTGGACTTCTTATTACCGTTCTATGATTCCGAAAAAATG
CTGGCATTTGGTCAGGTCGGAGCGCGTTACATTGACTCCCGCTTTACGGCAAATTTAGGT
GCGGGTCAGCGTTTTTTCCTTCCTGAAAATATGTTGGGCTATAACGTCTTCATTGATCAG
GATTTTTCTGGTGATAATACCCGTTTAGGTATTGGTGGCGAATACTGGCGAGACTATTTC
AAAAGTAGTGTTAACGGCTATTTCCGCATGAGCGGCTGGCATGAGTCATACAATAAGAAA
GACTATGATGAGCGCCCAGCAAATGGCTTCGATATCCGTTTTAATGGCTATCTGCCATCA
TACCCGGCATTAGGTGCCAGGCTGATGTATGAGCAGTATTATGGTGATAATGTTGCTTTG
TTTAATTCTGATAAGCTGCAGTCGAATCCTGGTGCGGCGACCGTTGGTGTAAACTATACT
CCGATTCCTCTGGTGACGATGGGGATCGATTACCGTCATGGTACGGGTAATGAAAATGAT
CTCCTTTACTCAATGCAGTTCCGTTATCAGTTTGATAAACCGTGGTCTCAGCAAATTGAG
CCACAGTATGTTAACGAGTTAAGAACATTATCAGGCAGCCGTTACGATCTGGTTCAGCGT
AATAACAATATTATTCTGGAGTACAAAAAGCAGGATATTCTTTCTCTGAATATTCCGCAT
GATATTAATGGTACTGAACGCAGTACGCAGAAGATTCAATTGATCGTTAAGAGCAAATAC
GGTCTGGATCGTATCGTCTGGGATGATAGTTCATTACGTAGCCAGGGCGGTCAGATTCAG
CATAGCGGAAGCCAAAGCGCACAAGATTACCAGGCTATTTTGCCTGCTTATGTGCAAGGT
GGCAGCAATATTTATAAAGTGACGGCTCGCGCCTATGACCGTAATGGCAATAGCTCTAAC
AATGTACAGCTTACTATTACCGTTCTGTCGAATGGTCAAGTTGTCGACCAGGTTGGGGTA
ACGGACTTTACGGCTGATAAGACATCGGCTAAAGCGGACGGCAACGAAGCGATTACTTAT
ACCGCGACGGTCAAAAAGAATGGGGTAGCTCAGGCTAATGTCCCTGTTTCATTTAACATT
GTTTCAGGAACTGCAGTTTTAAGTGCCAATAGTGCCAATACCAATGGTAGCGGTAAGGCG
ACTGTAACGCTGAAATCGGATAAACCAGGCCAGGTCGTCGTGTCTGCTAAAACAGCGGAG
ATGACTTCAGCGCTTAATGCCAATGCGGTCATATTTGTTGATCAAAGTAAGGCTAGTATT
ACCGAGATTAAGGCTGATAAAACAACTGCAGTAGCAAATGGTAAGGATGCTATTACATAC
ACTGTTAAAGTGATGAAAAATGGTCAGCCGGTATCCGGCGAAGAAGTGACCTTTACGAAG
ACCTTAGGTACATTAAGTAAGCCCACTGAAAAAACGGATGCGAATGGCTATGCTAAAGTA
ACATTAACATCGGCGACTCAAGGGAAATCCCTCGTTAGTGCCCGTGTTAGCGATGTCGCC
GTTGACGTCAAAGCCCCTGAAGTTGAATTTTTTACACCACTTACAGTTGATGACGGTAAT
ATTGAAATTGTTGGAACCGGAGTTAAAGGGAAGTTACCTACTGTATGGTTGCAATATGGA
CAAGTTAAGCTGAAAGCTAGCGGAGGTAACGGAAAATATACATGGCGCTCAGCAAATACA
GCGATTGCTTCGGTGGATGCCTCTTCTGGTCAGGTCACCTTAAAAGATAAGGGAACTACA
ACTATTACCGTTGTCTCAAGTGATAATCAAACCGCAACTTATACTATTGCAACACCTAAT
AGCCTGATTGTTCCTAATATGAGCAAGCGTGTGACTTATAATGATGCTGTGAATACATGT
AAGATTTTGGGAGGAAGATTACCCTCTTCTCAGGATGAACTGAAAAATGTCTTTAACACA
TGGGGAGCAGCAAATAAATATGAATATTACAAGTCTCTTCTGACTATAAGCTCTTGGGTA
CAACAAACAACAGTGGATATGAACAGTGGAGTTGCAAGTACATACGATCTAGTTAGACAA
AACCCTCTTAATGGCGTTGAAACTAATACTACTAATGCTTATGCCACTTGTGTAAAATAA
>eaeA_beta1_AJ277443
ATGATTACTCATGGTTTTTATGCCCGGACCCGGCACAAGCATAAGCTAAAAAAAACATTT
ATTATGCTTAGTGCTGGTTTAGGATTGTTTTTTTATGTTAACCAGAATTCATTTGCAAAT
GGTGAAAATTATTTTAAATTGAGTTCAGATTCAAAACTGTTAACTCAAAATGCCGCTCAG
GATCGCCTTTTTTATACGTTAAAAACAGGTGAAACTGTTGCCAATATTTCTAAATCACAG
GGTATCAGTTTATCGGTAATTTGGTCACTGAATAAACATTTATACAGTTCCGAAAGCGAA
ATGATGAAGGCTGGACCTGGTCAGCAGATCATTTTGCCACTCAAAAAACTGTCTGTTGAA
TATAGTGCCTTACCTGTCTTAGGTTCGGCACCTGTTGTTGCTGCAGGTGGTGTCGCTGGT
CATACGAATAAAATGACTAAAATGTCCCCGGACGCGACTAAAAGCAACACGACCGATGAC
AAGGCTCTAAATTATGCGGCACAACAGGCCGCGAGCCTTGGTAGCCAGCTCCAGTCGCGC
TCACTGAACGGCGATTACGCGAAAGATACCGCTCTTGGTATGGCCAGCAGCCAGGCTTCG
TCACAGTTGCAGGCCTGGTTACAACATTATGGAACGGCAGAGGTTAATCTGCAGAGTGGT
AATAACTTTGACGGTAGTTCACTGGACTTCTTATTACCGTTCTATGATTCCGAAAACATG
CTGGCATTTGGTCAGGTCGGTGCGCGTTACATTGACTCCCGCTTTACGGCAAATTTAGGT
GCTGGCCAGCGTTTTTTCCTTCCTGAAAATATGTTGGGCTATAACGTCTTCATTGATCAG

GATTTTTCTGGTGATAATACCCGTTTAGGTATTGGTGGCGAATACTGGCGAGACTATTTC
AAAAGTAGCGTTAACGGCTATTTCCGCATGAGCGGCTGGCATGAGTCATACAATAAGAAA
GACTATGATGAGCGCCCGGCAAATGGTTTTGATATCCGCTTTAATGGCTATTTACCATCA
TATCCGGCATTAGGCGCCAAACTGATGTACGAACAGTATTATGGTGATAATGTTGCTTTG
TTTAATTCCGATAAGTTGCAGTCGAATCCTGGCGCGGCGACCGTTGGTGTAAACTACACT
CCGATTCCTCTGGTGACGATGGGGATCGATTACCGTCATGGTACGGGTAATGAAAATGAT
CTCCTTTACTCAATGCAGTTCCGTTATCAGTTTGATAAACCGTGGTCTCAGCAAATCGAG
CCACAGTATGTTAACGAGTTAAGAACATTATCGGGCAGCCGTTACGATCTGGTTCAGCGT
AATAACAATATTATTCTGGAGTACAAAAAGCAGGATATTCTTTCTCTGAATATTCCGCAT
GATATTAATGGTACTGAACACAGTACGCAGAAGATTCAATTGATCGTTAAGAGCAAATAC
GGTCTGGATCGTATCGTCTGGGATGATAGCGCATTACGCAGTCAGGGCGGTCAGATTCAG
CATGGCGGAAGCCAAAGCGCACAAGACTACCAGGCTATTTTGCCTGCTTATGTGCAAGGC
GGCAGCAATATTTATAAAGTGACCGCTCGCGCCTATGACCGAAATGGTAATAGTTCTAAT
AATGTACAGCTCACTATTACCGTTTTACCGAATGGGCAGGTTGTGGACCAGGTTGGGGTA
ACGGACTTTACGGCTGATAAAACATCGGCTAAAGCGGATGGCATAGAAGCTATTACCTAT
ACCGCGACGGTTAAAAAGAATGGTGTAGCTCAGGCTAATGTCCCTGTAACATTTAGTATT
GTATCCGGGACTGCAACTCTTGGGGCAAATAGTGCCAGAACGGATGGTAACGGTAAGGCG
ACCGTAACGCTGAAGTCGGGTACGCCAGGACAGGTCGTCGTGTCTGCTAAAACCGCGGA
G
ATGACTTCGCCACTTAATGCCAGCGCGGTTATATTTGTTGATCAAACCAAGGCCAGTATT
ACTGAGATTAAGGCTGATAAAACAACAGCGAAGGCAGATGGTTCTGATGCGATTACCTAT
ACTGTCAGAGTGATGAAGGAGGGGGCACCCGTAGTAGATCAGAAAGTGACCTTTTCTAAG
GATTTTGGGACCCTGAATAAGACTGAAGCAACAACCGATCAGAATGGTTATGCTACTGTA
AAATTATCATCCAATACTCCTGGCAAGGCCATTGTTAGTGCAAAAGTGAGTGGAGTAGGT
ACAGAAGTTAAGGCTACTACCGTTGAGTTTTTTGCCCCGTTGAGTATTGATGGTGATAAA
GTGACCGTAATTGGTACTGGTATCACGGGGGCTCTGCCAAAGAACTGGTTACAGTATGGT
CAGGTTAAGCTACAGGCAACAGGGGGCAATGGAAAATACACATGGAAATCCAGTAATACT
AAAATTGCTTCTGTTGATAACTCGGGAGTGATAACCTTAAATGAAAAAGGGAGTGCCACA
ATTACTGTAGTATCTGGTGATAATCAGAGTGCGACATACACAATTAATGCACCGGGTAGT
ATTGTAATTGCTGTGGATAAAAATACTCGAGTTACGTATTTTGATGCCGAAAACAAATGT
AAGACAAATAGCGCAAATTTAGCACAGTCAAAAGAACTATTGGCCAATATCTATTCAACA
TGGGGTGCTGCAAATAAATATCCTTACTATTCTGGTTCTAAATCATTGACTGCTTGGATT
AAACAATCCTCTTCTGAACAGTCATCAGGTGTATCAAGCACATATGATTTGGTTACGAAG
AACCAGTTGATCAATGTTGGAGTAAACAATAAGAATGCTTTTTCTGTTTGTGTAAAATAA
>eaeA_beta2_DQ523605
ATGATTACTCATGGTTGTTATTCCCGGACCCGGCACAAGCATAAGCTAAAAAAAACATTT
ATTATGCTTAGTGCTGGTTTAGGATTGTTTTTTATGTTAATCAGAACTCATTTGCAAAT
GGTGAAAATTATTTTAAATTGGGTTCGGATTCAAAACTGTTAACTCATAATAGCTATCAG
AATCGCCTTTTTTATACGTTGAAAACAGGTGAAACTGTTGCCGATCTTTCTAAATCGCAA
GATATTAATTTATCGACGATTTGGTCGTTGAATAAGCATTTATACAGTTCTGAAAGCGAA
ATGATGAAGGCCGCGCCTGGTCAGCAGATCATTTTGCCACTCAAAAAACTTCCCTTTGAA
TACAGTGCCTTACCACTTTTAGGTTCGGCACCTCTTGTTGCTGCAGGTGGTGTCGCTGGT
CATACAAATAAACTGACTAAAATGTCCCCGGACGTGACCAAAAGCAACATGACCGATGAC
AAGGCATTAAATTATGCGGCACAACAGGCGGCGAGTCTCGGTAGCCAGCTTCAGTCGCGA
TCTCTGAACGGCGATTACGCGAAAGATACCGCTCTTGGTATCGCTGGTAACCAGGCTTCG
TCACAGTTGCAGGCCTGGTTACAACATTATGGAACGGCAGAGGTTAATCTGCAGAGTGGT
AATAACTTTGACGGTAGTTCACTGGACTTCTTATTACCGTTCTATGATTCCGAAAAAATG
CTGGCATTTGGTCAGGTCGGAGCGCGTTACATTGACTCCCGCTTTACGGCAAATTTAGGT
GCGGGTCAGCGTTTTTTCCTTCCTGAAAACATGTTGGGCTATAACGTCTTCATTGATCAG
GATTTTTCTGGTGATAATACCCGTTTAGGTATTGGTGGCGAATACTGGCGAGACTATTTC
AAAAGTAGTGTTAACGGCTATTTCCGCATGAGCGGCTGGCATGAGTCATACAATAAGAAA
GACTATGATGAGCGCCCAGCAAATGGCTTCGATATCCGTTTTAATGGCTATCTGCCATCA
TACCCGGCATTAGGCGCCAGGCTGATGTATGAGCAGTATTATGGTGATAATGTTGCTTTG
TTTAATTCTGATAAGCTGCAGTCGAATCCTGGTGCGGCGACCGTTGGTGTAAACTATACT
CCGATTCCTCTGGTGACGATGGGGATCGATTACCGTCATGGTACGGGTAATGAAAATGAT
CTCCTTTACTCAATGCAGTTCCGTTATCAGTTTGATAAACCGTGGTCTCAGCAAATTGAG
CCACAGTATGTTAACGAGTTAAGAACATTATCAGGCAGCCGTTACGATCTGGTTCAGCGT

AATAACAATATTATTCTGGAGTACAAAAAGCAGGATATTCTTTCTCTGAATATTCCGCAT
GATATTAATGGTACTGAACGCAGTACGCAGAAGATTCAATTGATCGTTAAGAGCAAATAC
GGTCTGGATCGTATCGTCTGGGATGATAGTTCATTACGTAGCCAGGGCGGCCAGATTCAG
CATAGCGGAAGCCAAAGCGCACAAGATTACCAGGCTATTTTGCCTGCTTATGTGCAAGGT
GGTAGCAACATTTATAAAGTGACGGCTCGTGCCTATGACCGTAATGGCAATAGCTCTAAC
AATGTACAGCTTACTATTACCGTTCTGTCGAATGGTCAGGTGGTCGACCAGGTTGGGGTA
ACGGACTTTACGGCGGATAAGACTTCGGCTAAAGCGGATGGCACCGAAGCAATTACTTAT
ACTGCGACGGTGAAAAAGAATGGGGTAGCTCAGGTTAATGTCCCGGTTTCATTTAATATT
GTTTCAGGAACTGCAGTTTTAAGTGCCAATAGTGCCAATACCAATGGTAGCGGTAAGGCG
ACTGTAACCCTGAAATCGGATAAACCAGGCCAGGTCGTCGTGTCTGCTAAAACAGCAGAG
ATGACTTCAGCGCTTAATGCCAATGCAGTTATATTTGTTGATCAAACCAAGGCCAGCATT
ACTGAGATTAAGGCTGATAAAACAACTGCGAAGGCAAATGGTTCCGATGCTGTTACATAC
ACTGTTAAAGTGATGAAGGGGGGAACCCCTGTGTCAGGTCAAAAAGTGACCTTCTCTAAG
GATTTTGGGACACTGGATAAGACTGAAGCAACAACTGATCAGAATGGTTATGCTACTGTA
AAACTGTCATCTAGCACTCCAGGCAAAGCTATTGTTAGCGCAAAAGTGAGTGATGTAGAT
ACAGAAGTTAAGGCTACTACTGTTGAGTTTTTTACCCCACTAAGTATCGATGGTAATAAA
GTGACTGTAATTGGTACTGGTGTCACTGGTTCTCTGCCAAATAACTGGCTGCAGTATGGG
AAGGTTAAGCTACAGGCTGCGGGAGGCAATGGGAAATATACATGGAAATCCAGTGATACT
AAAATCGCCTCTGTTGATAGTACGGGTGTGATAACCTTAAATGAGAAAGGGAGTGCCACA
ATTACTGTAGTATCTGGAGATAATCAGAGTGCGACATATACAATTAATGCGCCAAGCAGT
ATCGTAATTGCTGTGGATAAAAATACTCGAGTTACGTATTCTGAGGCTGAAAATAAATGC
CAGACAAATGGAGCAGCTTTAGCACAGTCAAAAGAAATATTGGCAAATATTTATTCTTCG
TGGGGGGCTGCGAATAAATATCCTTATTATTCTAGCTCTAAATCATTGACTACTTGGATT
AAACAATCCTCTTCTGAACTCTCATCAGGTGTATCAAGTACATATGATTTAGTTACGAAG
AACCAATTGACTAATGTGGGAGTAAAAAATAAAAATGCTTTTGCAGTTTGTGTAAAATAA
>eaeA_beta3_AJ876654
ATGATTACTCATGGTTTTTATGCCCGGACCCGGCACAAGCATAAGCTAAAAAAAACATTT
ATTATGCTTAGTGCTGGTTTAGGATTGTTTTTTATGTTAACCAGAATTCATTTGCAAAC
GGTGAAAATTATTTTAAATTGAGTTCAGATTCAAAACTGTTAACTCAAAATGCCGCTCAG
GATCGCCTTTTTTATACGTTAAAACCAGGTGAAACTGTTGCCAATATTTCTAAATCACAG
GGTATCAGTTTATCGGTAATTTGGTCACTGAATAAACATTTATACAGTTCCGAAAGCGAA
ATGATGAAGGCTGCGCCTGGTCAGCAGATCATTTTGCCACTCAAAAAACTGTCTGTTGAA
TATAGTGCCTTACCTGTCTTAGGTTCGGCACCTGTTGTTGCTGCAGGTGGTGTCGCTGGT
CATACGAATAAAATGACTAAAATGTCCCCGGACGCGACTAAAAGCAACACGACCGATGAC
AAGGCTCTAAATTATGCGGCACAACAGGCCGCGAGCCTTGGTAGCCAGCTCCAGTCGCGC
TCACTGAACGGCGATTACGCGAAAGATACCGCTCTTGGTATGGCCAGCAGCCAGGCTTCG
TCACAGTTGCAGGCCTGGTTACAACATTATGGAACGGCAGAGGTTAATCTGCAGAGTGGT
AATAACTTTGACGGTAGTTCACTGGACTTCTTATTACCGTTCTATGATTCCGAAAACATG
CTGGCATTTGGTCAGGTCGGGGCGCGTTACATTGACTCCCGCTTTACGGCAAATTTAGGT
GCTGGCCAGCGTTTTTTCCTTCCTGAAAATATGTTGGGCTATAACGTCTTCATTGATCAG
GATTTTTCTGGTGATAATACCCGTTTAGGTATTGGTGGCGAATACTGGCGAGACTATTTC
AAAAGTAGCGTTAACGGCTATTTCCGCATGAGCGGCTGGCATGAGTCATACAATAAGAAA
GACTATGATGAGCGCCCGGCAAATGGTTTTGATATCCGCTTTAATGGCTATTTACCATCA
TATCCGGCATTAGGCGCCAAACTGATGTACGAACAGTATTATGGTGATAATGTTGCTTTG
TTTAATTCCGATAAGTTGCAGTCGAATCCTGGCGCGGCGACCGTTGGTGTAAACTACACT
CCGATTCCTCTGGTGACGATGGGGATCGATTACCGTCATGGTACGGGTAATGAAAATGAT
CTCCTTTACTCAATGCAGTTCCGTTATCAGTTTGATAAACCGTGGTCTCAGCAAATCGAG
CCACAGTATGTTAACGAGTTAAGAACATTATCGGGCAGCCGTTACGATCTGGTTCAGCGT
AATAACAATATTATTCTGGAGTACAAAAAGCAGGATATTCTTTCTCTGAATATTCCGCAT
GATATTAATGGTACTGAACACAGTACGCAGAAGATTCAATTGATCGTTAAGAGCAAATAC
GGTCTGGATCGTATCGTCTGGGATGATAGTGCATTACGCAGTCAGGGCGGTCAGATTCAG
CATAGCGGAAGCCAAAGCGCACAAGACTACCAGGCTATTTTGCCTGCTTATGTGCAAGGT
GGCAGCAATATTTATAAAGTGACCGCTCGCGCCTATGACCGAAATGGTAATAGTTCTAAT
AATGTACAGCTCACTATTACCGTTTTACCGAATGGGCAGGTTGTGGACCAGGTTGGGGTA
ACGGACTTTACGGCTGATAAGACATCGGCTAAAGCGGATAACGCTGATACCATTACTTAT
ACCGCGACGGTTAAAAAGAATGGTGTAGCTCAGGCTAATGTCCCTGTAACATTTAGTATT
GTATCCGGGACTGCAACTCTTGGGGCAAATAGTGCCAAAACGGATGGTAACGGTAAGGCA

ACCGTAACGTTGAAGTCGGTTACGCCAGGACAGGTCGTCGTGTCTGCTAAAACCGCGGAG
ATGACTTCGCCACTTAATGCCAATGCGGTTATATTTGTTGATCAAACCCAGGCCAGTATT
ACTGAGATTAAGGCTGATAAAACAACAGCGAAGGCAGATGGTTCTGATGCGATTACCTAT
ACTGTCAGAGTGATGAAGGAGGGGGCACCCGTAGTAGATCAGAAAGTGACCTTTTCTAAG
GATTTTGGGACCCTGAATAAGACTGAAGCAACAACCGATCAGAATGGTTATGCTACTGTA
AAATTATCATCCAATACTCCTGGCAAGGCCATTGTTAGTGCAAAAGTGAGTGGAGTAGGT
ACAGAAGTTAAGGCTACTACCGTTGAGTTTTTTGTCCCGTTGAGTATTGATGGTGATAAA
GTGACCGTAATTGGTACTGGTGTCACGGGGGCTCTGCCAAAGAATTGGTTACAGTATGGT
CAGGTTAAGCTACAGGCAACAGGGGGCAATGGAAAATATACATGGAAATCCAGTAATACT
AAAATTGCTTCTGTTGATAACTCGGGAACGATAACCTTAAATGAAAAAGGGAGTGCCACA
ATTACTGTAGTATCTGGTGATAATCAGAGTGCGACATATACAATTAATGCACCAAGCAGT
ATCGTAATCGCTGTGGATAAAGTTAATCGGATGACATATTCTGCTGCCGAAAACAAGTGT
CGAGCAATTGGTGCAAATTTAGCACAGTCAAAGAGTATATTGACAGACACATATTCTAAA
TGGGGAGCTGCTAATAAATATTCTTACTATTCTGGTTCTAATTCATTGACAGCTTGGATT
ACACAATCCTCTTCTGAGTTACAATCGGGCGTATCAAGTACATATGATTTAATTACAACA
AACTCTTTGATAAATGTTAAGACAACAGATAACAATGCCTTTGCAGTTTGTGTAAAATAA
>eaeA_Crodentium_AF311901
ATGATTATTCATGGTTTTTGCACCGGGACCCGGCACAAGCATAAGTTAAGAAAAACATTT
ATTATGCTTGGTGCTGGTTTAGGATTGTTTTTTTCTGTTAACCAGAACTCATTTGCAAAT
GGTGAAAATTACTTTAAGTTAAGAGCCGATTCTAAACTAATAAATAATAATATTGCTCAG
GATCGTCTTTTTTATACCTTGAAAACGGGTGAAAGCGTTGCTCAGCTTTCTAAATCACAA
GGGATCAGCGTGCCGGTTATTTGGTCGCTGAATAAGCATTTATATAGCTCTGAAAGTGAA
ATGATGAAGGCTAGTCCTGGTCAGCAGATCATTTTACCGCTCAAAAAACTTTCTGCTGAA
TATAGTACCTTGCCTATTTTAGGTACAGCACCTGTCGTTGCTGCAGCTGATGTTGCTGGT
CACACGAAAAAAATGTCCCAGGATACGACCAAAAGCAATACAAGCGATGACAAGGCCTT
A
AACTATGCAGCACAACAGGCCGCGAGCCTCGGTAGCCAACTTCAGTCTCGCTCACTGAAC
GGAGACTATGCGAAAGATACAGCCCTTAGTATGGCTGGTAACCAAGCCTCATCCCAGATG
CAGGCTTGGTTACAGCATTATGGAACGGCAGAGGTTAATCTGCAGAGCGGTAATAACTTT
GATGGCAGTTCACTGGACTTCTTATTACCGTTCTATGATACTGAAAATATGCTGGCATTT
GGTCAGGTTGGAGCGCGTTATATTGACTCTCGCTTTACGGCAAACTTAGGGGCTGGTCAG
CGTTTTTTCCTTCCTGAAAATATGTTGGGCTATAACGTCTTCATTGATCAGGATTTTTCT
GGTAACAATACCCGCTTAGGAATCGGCGGTGAATATTGGCGAGACTATTTTAAAAGTAGC
GTTAACGGCTATTTCCGCATGAGCGGTTGGCATGAGTCATACAATAAGAAAGACTATGAT
GAGCGCCCGGCAAATGGTTTCGATATTCGCTTTAACGGCTACTTACCATCATATCCTGCA
TTAGGTGGCAAGCTGATGTATGAGCAGTATTATGGTGATAATGTTGCTTTGTTTAATGCC
GATAAGCTGTATTCGAATCCTGGTGCGGTGACAGTTGGTGTCAACTACACTCCGATTCCT
TTGGTGACGATGGGGATTGATTATCGTCATGGTACAGGTAATGAAAATGATCTCCTGTAC
TCAATGCAGTTCCATTATCAATTTGATAAACCATGGTCTCAACAAATTGAGCCACAGTAT
GTTAACGAGTTAAGAACATTATCAGGCAGCCGTTACGATCTGGTACAGCGTAATAACAAC
ATTATTCTGGATTACAAAAAGCAGGATATTCTTTCTATGAATATTCCGCATAATATTAAT
GGTACTGAACACAGTACGCATAAGATCCAATTGATCGTTAAGAGTAAGTACGGTCTTGAG
CGTATCGTATGGGATGACAGTACATTACGCACTCAGGGTGGTCAGATTCAGCATAGCGGA
AGCCAAAGCGCACAAGATTATCAGGCTATTTTGCCTGCTTATGTGCAAGGCGGTAGCAAT
GTTTATAAAGTGACCGCTCGCGCCTATGACCGTAATGGAAATAGCTCTAACAATGTACAG
CTTACTATTACCGTTCTGTCGAATGGTCAAGTAGTCGACAAGGTTGGGATAACGAACTTT
ACGGCGGATAAGACATCGGCTAAAGCGGATAATGCGGATACCATTACTTATACCGCGACT
GTGAAAAAGAATGGGGTAGCTCAGGCTAATGTCCCTGTTTCATTTAATATTGTTTCAGGA
ACTGCAACTTTAAGTGCCAAGAGTGCCAATACTAATAGTAGCGGTAAGGCGACTGTAACG
CTGAAATCAGACAAGCCGGGCCAGGTAGTTGTGTCAGCTAAAACAGCGGAGATGACTTCA
GCACTTAATGCCAATGCGGTTATATTTGTTGATCAAACCAAAGCCAGCATTACTGAGATT
AAGGTTGATAAGACAATAGCGACGGCAGACAATAAGGATACTATTGAATACACAGTTAAA
GTGATGAAGGGTGGGAATCCTATTTCTGGTCAAAAAGTGACCTTCTCTAAGGATTTTGGG
ACCCTAAATAAGACTGAAGCAACAACCGATCAGAATGGTTATGCTACTGTAAAATTGTCA
TCCGGAACTCCGGGTAAGGCTATAGTTAGCGCAAAAGTGAGTGAGGTAAATACAGAAGTT
AAGGCCGCCACTGTTGAATTTTTTGCCCCACTGAGTATCGATGGCAATAAAGTGACTGTA
ATTGGTACTGGTGTCACTGGTTCTTTGCCAAAGAACTGGTTACAGTATGGTCAGGTTAAG

CTACAGGCAACAGGAGGCAACGGGAAATATACATGGAAATCCAGTAATACTAAAATTGCT
TCTGTTGATAATTCGGGAGTGATAACTTTAAATGAGAAAGGCAGTGCTACAATTACTGTA
GTATCGGGTGATAATCAGAGTGCGACATATACAATTAATACACCAGACAATATTATAATA
GCGGTGGATAAAATTAATCGGATGGCGTATTCTGAGGCAGAAAGCAGGTGTCAAGCAATT
AGTTCAAATTTAGCACAGTCAAAAAGCGTATTGGAAAATATATATTCTAAATGGGGAGCC
GCGAATAAATATCCTTATTATTCGAGTTCTAATTCATTGACAGCTTGGATTAAGCAATCT
ACTTCTGATTCTGCATCAGGTGTATCAAATACATATGATTTAGTTACAACAAACTCTTTG
ACAAATGTTAAAGCAACAGATAAAAATGCTTTTGCAGTTTGTGTAAAATAG
>eaeA_epsilon_CB10113
ATGATTACTCATGATTTTTATGCCCGGACCCGGCACAAGCATAAGCTAAAAAAAACATTT
ATTATGCTTAGTGCTGGTTTAGGATTGTTTTTTATGTTAACCAGAATTCATTTGCAAAC
GGTGAAAATTATTTAAATTGAGTTCAGATTCAAAACTGTTAACTCAAAATGCCGCTCAG
GATCGCCTTTTTTATACGTTGAAAACAGGTGAAACTGTTGCCAATATTTCTAAATCACAG
GGTATCAGTTTATCGGTAATTTGGTCACTGAATAAACATTTATACAGTTCCGAAAGCGAA
ATGATGAAGGCTGGGCCTGGTCAGCAGATCATTTTGCCACTCAAAAAACTGTCTGTTGAA
TATAGTGCCTTACCTGTCTTAGGTTCGGCACCTGTTGTTGCTGCAGGTGGTGTCGCTGGT
CATACGAATAAAATGACTAAAATGTCCCCGGACGCGACTAAAAGCAACACGACCGGTGAC
AAGGCTCTAAATTATGCGGCACAACAGGCCGCGAGCCTTGGTAGCCAGCTTCAGTCGCGC
TCACTGAACGGCGATTACGCGAAAGATACCGCTCTTGGTATGGCCAGCAGCCAGGCTTCG
TCACAGTTGCAGGCCTGGTTACAACATTATGGAACGGCAGAGGTTAATCTGCAGAGTGGT
AATAACTTTGACGGTAGTTCACTGGACTTCTTATTACCGTTCTATGATTCCGAAAACATG
CTGGCATTTGGTCAGGTCGGTGCGCGTTACATTGACTCCCGCTTTACGGCAAATTTAGGT
GCTGGCCAGCGTTTTTTCCTTCCTGAAAATATGTTGGGCTATAACGTCTTCATTGATCAG
GATTTTTCTGGTGATAATACCCGTTTAGGTATTGGTGGCGAATACTGGCGAGACTATTTC
AAAAGTAGCGTTAACGGCTATTTCCGCATGAGCGGCTGGCATGAGTCATACAATAAGAAA
GACTATGATGAGCGCCCGGCAAATGGTTTTGATATCCGCTTTAATGGCTATTTACCATCA
TATCCGGCATTAGGCGCCAAACTGATGTACGAACAGTATTATGGTGATAATGTTGCTTTG
TTTAATTCCGATAAGTTGCAGTCGAATCCTGGCGCGGCGACCGTTGGTGTAAACTACACT
CCGATTCCTCTGGTGACGATGGGGATCGATTACCGTCATGGTACGGGTAATGAAAATGAT
CTCCTTTACTCAATGCAGTTCCGTTATCAGTTTGATAAACCGTGGTCTCAGCAAATCGAG
CCACAGTATGTTAACGAGTTAAGAACATTATCGGGCAGCCGTTACGATCTGGTTCAGCGT
AATAACAATATTATTCTGGAGTACAAAAAGCAGGATATTCTTTCTCTGAATATTCCGCAT
GATATTAATGGTACTGAACACAGTACGCAGAAGATTCAATTGATCGTTAAGAGCAAATAT
GGTCTGGATCGTATCGTCTGGGATGATAGTGCATTACGCAGTCAGGGCGGTCAGATTCAG
CATAGCGGAAGCCAAAGCGCACAAGACTACCAGGCTATTTTGCCTGCTTATGTGCAAGGT
GGCAGCAATATTTATAAAGTGACCGCTCGCGCCTATGACCGAAATGGTAATAGTTCTAAT
AATGTACAGCTCACTATTACCGTTTTACCGAATGGGCAGGTTGAGGACCAGGTTGGGGTA
ACGGACTTTACGGCTGATAAAACATCGGCTAAAGCGGATGGCATAGAAGCTATTACCTAT
ACCGCGACGGTTAAAAAGAATGGGGTAGCTCAGGCTAATGTCCCTGTAACATTTAGTATT
GTATCCGGAACTGCAACTCTTGGGGCAAATAGTGCCAAAACGGATGGTAACGGTAAGGCA
ACCGTAACGTTGAAGTCGGCTACGCCAGGACAGGTCGTAGTGTCTGCTAAAACCGCGGAG
ATGACTTCAGCTATTAATGCTGGCCCGGTTATATTCATCGATCAAACCAAGGCCAGTATT
ACTGAAATTACCAATGATAAGTCAACGGCAATAGCAAATGATAAGGACGCCATTACATAT
ACTGTTAAGGTAATGAAAAATGATCAGCCAGTACCGAATCATTTAGTCACATTCACGACG
ACTTTTGGTAAATTCAATGGTAAACAGAGCTCAGAAACCGTAACAACAGGGAACGACGG
A
CGCGCTATAGTGACACTGACATCAGGTTTAGCCGGAAAAGCAATTGTCAGTGCAAAAGTT
AATGAAGTAAATACCGAAGTAAAAGCAAAGACTGTTGAGTTTTTCTCAAAACTAGTAGTC
GATAGCAATATAGATATTATTGGTACATCCGTGACAGGAGCTTTGCCTGATGTCTGGTTG
CAATATGGCCAGTTTAAATTGACTGCTAAAGGTGGAAATGGGAAATATACATGGTATTCT
GAAAATCCAAATATTGCATCCGTGGAATCAACTACAGGACTAGTAACCTTAAATAATAAA
GGGAGTGTTAAAATATACGTTTTATCAGATGATAAGCAAACAGCAAGTTATACAATATCT
ACGCCCAAAGATATGATAACAGCTGATATAACAAAACCAGCTATCTATAGTATTGCATTT
AAGCAATGCCAAACAAAAAATGGAAGACTACCATCTTCACAGCGTGAATTAGAAAACGTC
TTTAATCTCTGGGGGGCTGCAAATAACTATAGATATTATTCTGCCAAAAAATCTATAACA
GCATGGGTTCAACAATCTGGTTCAGATTCTAGCGGTGGAGTGACTACAACATATGATCTG
ATAAAGAAGAATCCTCGACAGAATGTTGATGTGAACAGTCCAAATGTCTATTCTGTGTGC

GTTGAATAA
>eaeA_epsilon1_DQ523606
ATGATTACTCATGGTTTTTATACCCGGACCCGGCACAAGCATAAGCTAAAAAAAACATTT
ATTATGCTTAGTGCTGGTTTAGGATTGTTTTTTTATGTTAACCAGAATTCATTTGCAAAT
GGTGAAAATTATTTTAAATTGAGTTCAGATTCAAAACTGTTAACTCAAAATGCCGCTCAG
GATCGCCTTTTTTATACGTTAAAAACAGGTGAAACTGTTGCCAATATTTCTAAATCACAG
GGGATCAGTTTATCGGTAATTTGGTCACTGAATAAACATTTATACAGCTCCGAAAGCGAA
ATGATGAAGGCTGGGCCTGGTCAGCAGATCATTTTGCCACTCAAAAAACTGTCTGTTGAA
TATAGTGCCTTACCTGTCTTAGGTTCGGCACCTGTTGTTGCTGCAGGTGGTGTCGCTGGT
CATACGAATAAAATGACTAAAATGTCCCCGGACGCGACTAAAAGCAACACGACCGATGAC
AAGGCTCTAAATTATGCGGCACAACAGGCCGCGAGCCTTGGTAGCCAGCTCCAGTCGCGC
TCACTGAACGGCGATTACGCGAAAGATACCGCTCTTGGTATGGCCAGCAGCCAGGCTTCG
TCACAGTTGCAGGCCTGGTTACAACATTATGGAACGGCAGAGGTTAATCTGCAGAGTGGT
AATAACTTTGACGGTAGTTCACTGGACTTCTTATTACCGTTCTATGATTCCGAAAACATG
CTGGCATTTGGTCAGGTCGGGGCGCGTTACATTGACTCCCGCTTTACGGCAAATTTAGGT
GCTGGCCAGCGTTTTTTCCTTCCTGAAAATATGTTGGGCTATAACGTCTTCATTGATCAG
GATTTTTCTGGTGATAATACCCGTTTAGGTATTGGTGGCGAATACTGGCGAGACTATTTC
AAAAGTAGCGTTAACGGCTATTTCCGCATGAGCGGCTGGCATGAGTCATACAATAAGAAA
GACTATGATGAGCGCCCGGCAAATGGTTTTGATATCCGCTTTAATGGCTATTTACCATCA
TATCCGGCATTAGGCGCCAAACTGATGTACGAACAGTATTATGGTGATAATGTTGCTTTG
TTTAATTCCGATAAGTTGCAGTCGAATCCTGGCGCGGCGACCGTTGGTGTAAACTACACT
CCGATTCCTCTGGTGACGATGGGGATCGATTACCGTCATGGTACGGGTAATGAAAATGAT
CTCCTTTACTCAATGCAGTTCCGTTATCAGTTTGATAAACCGTGGTCTCAGCAAATCGAG
CCACAGTATGTTAACGAGTTAAGAACATTATCGGGCAGCCGTTACGATCTGGTTCAGCGT
AATAACAATATTATTCTGGAGTACAAAAAGCAGGATATTCTTTCTCTGAATATTCCGCAT
GATATTAATGGTACTGAACACAGTACGCAGAAGATTCAATTGATCGTTAAGAGCAAATAT
GGTCTGGATCGTATCGTCTGGGATGATAGTGCATTACGCAGTCAGGGCGGTCAGATTCAG
CATAGCGGAAGCCAAAGCGCACAAGACTACCAGGCTATTTTGCCTGCTTATGTGCAAGGT
GGCAGCAATATTTATAAAGTGACCGCTCGCGCCTATGACCGAAATGGTAATAGTTCTAAT
AATGTACAGCTCACTATTACCGTTTTACCGAATGGGCAGGTTGTGGACCAGGTTGGGGTA
ACGGACTTTACGGCTGATAAGACATCGGCTAAAGCGGATAACGTTGATACCATTACTTAT
ACCGCGACGGTTAAAAAGAATGGTGTAGCTCAGGCTAATGCCCCTGTAACATTTAGTATT
GTATCCGGGACTGCAACTCTCGGGGCAAATAGTGCCAAAACGGATGGTAACGGTAAGGCA
ACCGTAACGTTGAAGTCGGGTACGCCAGGGCAGGTCGTCGTGTCTGCTAAAACCGCGGA
G
ATGACTTCGCCACTTAATGCCAGTGCGGTTATATTTGTTGATCAAACCAAGGCCAGCATT
ACTGAGATTAAGGCTGATAAAACAACAGCGAAGGCAAATGGTTCTGATGCGATTACCTAT
ATTGTTAAAGTAATGAAGAATAACCAACCAGAAGCAAACCATTCTGTTACATTCTCAACG
AACTTTGGTAATCTGGGGGGAAATTCTAATACCCAAATTGTGAAAACGGATAAAGATGGT
AGGGCTACGGTAAAACTGACATCTGGCGTTGCAGGTAATGCTGTTGTTAGTGCAAAAGTC
AGCGAAGTTAATACAGAGGTTAAGGCTCCTGAGGTAAAATTCTTCTCAGTTCTGAGCATT
GATAGTAATGTGAGTATTATTGGAACCTCCGCTAATGGCGCTTTACCTAATATTTGGTTG
CGATATGGTCAGTTTAAGCTGACAGCCAAAGGTGGCGATGGGAAATATCAATGGCGCTCT
CAAGATCCAAGTGTTGCATCAGTTGATGCTTTAACTGGTCGAGTTACTTTGCTGAAGAAA
GGAACAACAACAATTGAAGTTGTATCGGGTGATAACCAAACAGCAATGTATACAATTAAT
ACACCTACAAAATTTATATCTGTGGAGACACAAAATAAAGTAGTCTATAGTGATGCTGAG
GCAACATGTAGAATGAATAATGCACGCTTGCCGTCATCTACGAGTGAGCTAAAGGATGTG
TATAATAAATGGGGCGCCGCCAATAGTTATGAAGGCTATAAAGGTAAAAAAACAATAACA
GCATGGACACAGCAAACTGAGGATGATAAACAAAAAGGTTGGACTAGTACATTTGACATA
GTTACAAAAAATGAAATCCCTAGTAATGGCAGTAATAGTAAAGTCCACGTGAATAAAGCT
AACGCTTTTGCCGTCTGTGTAAGATGA
>eaeA_epsilon1_DQ523612
ATGATTACTCATGGTTTTTATACCCGGACCCGGTACAAGCATAAGCTAAAAAAAACATTT
ATTATGCTTAGTGCTGGTTTAGGATTGTTTTTTTATGTTAATCAGAATTCATTTGCAAAT
GGTGAAAATTATTTTAAATTGAGTTCAGATTCAAAACTGTTAACTCAAAATGCCGCTCAG
GATCGCCTTTTTTATACGTTAAAAACAGGTGAAACTGTTGCCAATATTTCTAAATCACAG
GGGATCAGTTTATCGGTAATTTGGTCACTGAATAAACATTTATACAACTCCGAAAGCGAA

ATGATGAAGGCTGGGCTTGGTCAGCAGATCATTTTGCCACTCAAAAAACTGTCTGTTGAA
TATAGTGCCTTACCTGTCTTAGGTTCGGCACCTGTTGTTGCTGCAGGTGGTGTCGCTGGT
CATACGAATAAAATGACTAAAATGTCCCCGGACGCGACTAAAAGCAACACGACCGATGAC
AAGGCTCTAAATTATGCGGCACAACAGGCCGCGAGCCTTGGTAGCCAGCTCCAGTCGCGC
TCACTGAACGGCGATTACGCGAAAGATACCGCTCTTGGTATGGCCAGCAGCCAGGCTTCG
TCACAGTTGCAGGCCTGGTTACAACATTATGGAACGGCAGAGGTTAATCTGCAGAGTGGT
AATAACTTTGACGGTAGTTCACTGGACTTCTTATTACCGTTCTATGATTCCGAAAACATG
CTGGCATTTGGTCAGGTCGGGGCGCGTTACATTGACTCCCGCTTTACGGCAAATTTAGGT
GCTGGCCAGCGTTTTTTCCTTCCTGAAAATATGTTGGGCTATAACGTCTTCATTGATCAG
GATTTTTCTGGTGATAATACCCGTTTAGGTATTGGTGGCGAATACTGGCGAGACTATTTC
AAAAGTAGCGTTAACGGCTATTTCCGCATGAGCGGCTGGCATGAGTCATACAATAAGAAA
GACTATGATGAGCGCCCGGCAAATGGTTTTGATATCCGCTTTAATGGCTATTTACCATCA
TATCCGGCATTAGGCGCCAAACTGATGTACGAACAGTATTATGGTGATAATGTTGCTTTG
TTTAATTCCGATAAGTTGCAGTCGAATCCTGGCGCGGCGACCGTTGGTGTAAACTACACT
CCGATTCCTCTGGTGACGATGGGGATCGATTACCGTCATGGTACGGGTAATGAAAATGAT
CTCCTTTACTCAATGCAGTTCCGTTATCAGTTTGATAAACCGTGGTCTCAGCAAATCGAG
CCACAGTATGTTAACGAGTTAAGAACATTATCGGGCAGCCGTTACGATCTGGTTCAGCGT
AATAACAATATTATTCTGGAGTACAAAAAGCAGGATATTCTTTCTCTGAATATTCCGCAT
GATATTAATGGTACTGAACACAGTACGCAGAAGATTCAATTGATCGTTAAGAGCAAATAT
GGTCTGGATCGTATCGTCTGGGATGATAGTGCATTACGCAGTCAGGGCGGTCAGATTCAG
CATAGCGGAAGCCAAAGCGCACAAGACTACCAGGCTATTTTGCCTGCTTATGTGCAAGGT
GGCAGCAATATTTATAAAGTGACCGCTCGCGCCTATGACCGAAATGGTAATAGTTCTAAT
AATGTACAGCTCACTATTACCGTTTTACCGAATGGGCAGGTTGTGGACCAGGTTGCGGTA
ACGGACTTTACGGCTGATAAGACATCGGCTAAAGCGGATAACGTTGATACCATTACTTAT
ACCGCGACGGTTAAAAAGAATGGTGTAGCTCAGGCTAATGCCCCTGTAACATTTAGTATT
GTATCCGGGACTGCAACTCTCGGGGCAAATAGTGCCAAAACGGATGGTAACGGTAAGGCA
ACCGTAACGTTGAAGTCGGGTACGCCAGGGCAGGTCGTCGTGTCTGCTAAAACCGCGGA
G
ATGACTTCGCCACTTAATGCCAGTGCGGTTATATTTGTTGATCAAACCAAGGCCAGCATT
ACTGAGATTAAGGCTGATAAAACAACAGCGAAGGCAAATGGTTCTGATGCGATTACCTAT
ATTGTTAAAGTAATGAAGAATAACCAACCAGAAGCAAACCATTCTGTTACATTCTCAACG
AACTTTGGTAATCTGGGGGGAAATTCTAATACCCAAATTGTGAAAACGGATAAAGATGGT
AGGGCTACGGTAAAACTGACATCTGGCGTTGCAGGTAATGCTGTTGTTAGTGCAAAAGTC
AGCGAAGTTAATACAGAGGTTAAGGCTCCTGAGGTAAAATTCTTCTCAGTTCTGAGCATT
GATAGTAATGTGAGTATTATTGGAACCTCCGCTAATGGCGCTTTACCTAATATTTGGTTG
CGATATGGTCAGTTTAAGCTGACAGCCAAAGGTGGCGATGGGAAATATCAATGGCGCTCT
CAAGATCCAAGTGTTGCATCAGTTGATGCTTTAACTGGTCGAGTTACTTTGCTGAAGAAA
GGAACAACAACAATTGAAGTTGTATCGGGTGATAACCAAACAGCAATGTATACAATTAAT
ACACCTACAAAATTTATATCTGTGGAGAAACAAAATAAAGTAGTCTATAGTGATGCTGAG
GCAACATGTAGAATGAATAATGCACGCTTGCCGTCATCTACGAGTGAGCTAAAGGATGTG
TATAATAAATGGGGCGCCGCCAATAGTTATGAAGGCTATAAAGGTAAAAAAACAATAACA
GCATGGACACAGCAAACTGCGGATGATAAACAAAAAGGTTGGACTAGTACATTTGACATA
GTTACAAAAAATGAAATCCCTAGTAATGGCAGTAATAGTAAAGTCCACGTGAATAACGCT
AACGCTTTTGCCGTCTGTGTAAGATGA
>eaeA_epsilon2_DQ523614
ATGATTACTCATGGTTTTTATGCCCGGACCCGGCACAAGCATAAGCTAAAAAAAACATTT
ATTATGCTTAGTGCTGGTTTAGGATTGTTTTTTATGTTAATCAGAATTCATTTGCAAAT
GGTGAAAATTATTTTAAATTGGGTTCGGATTCAAAACTGTTAACTCATGATAGCTATCAG
AATCGCCTTTTTTATACGTTGAAAACAGGTGAAACTGTTGCCGATCTTTCTAAATCGCAA
GATATTAATTTATCGACGATTTGGTCGTTGAATAAGCATTTATACAGTTCTGAAAGCGAA
ATGATGAAGGCCGCGCCTGGTCAGCAGATCATTTTGCCACTCAAAAAACTTCCCTTTGAA
TACAGTGCACTACCACTTTTAGGTTCGGCACCTCTTGTTGCTGCAGGTGGTGTTGCTGGT
CACACGAATAAACTGACTAAAATGTCCCCGGACGTGACCAAAAGCAACATGACCGATGAC
AAGGCATTAAATTATGCGGCACAACAGGCGGCGAGTCTCGGTAGCCAGCTTCAGTCGCGC
TCTCTGAACGGCGATTACGCGAAAGATACCGCTCTTGGTATCGCTGGTAACCAGGCTTCG
TCACAGTTGCAGGCCTGGTTACAACATTATGGAACGGCAGAGGTTAATCTGCAGAGTGGT
AATAACTTTGACGGTAGTTCACTGGACTTCTTATTACCGTTCTATGATTCCGAAAAAATG

CTGGCATTTGGTCAGGTCGGAGCGCGTTACATTGACTCCCGCTTTACTGCAAATTTAGGT
GCGGGTCAGCGTTTTTTCCTTCCTGAAAACATGTTGGGCTATAACGTCTTCATTGATCAG
GATTTTTCTGGTGATAATACCCGTTTAGGTATTGGTGGCGAATACTGGCGAGACTATTTC
AAAAGTAGTGTTAACGGCTATTTCCGCATGAGCGGCTGGCATGAGTCATACAATAAGAAA
GACTATGATGAGCGCCCAGCAAATGGCTTCGATATCCGTTTTAATGGCTACCTACCATCA
TACCCGGCATTAGGCGCCAGGCTGATGTATGAGCAGTATTATGGTGATAATGTTGCTTTG
TTTAATTCCGATAAGCTGCAGTCGAACCCTGGTGCGGCGACCGTTGGTGTAAACTATACT
CCGATTCCTCTGGTGACGATGGGGATCGATTACCGTCATGGTACGGGTAATGAAAATGAT
CTCCTTTACTCAATGCAGTTCCGTTATCAGTTTGATAAACCGTGGTCTCAGCAAATTGAG
CCACAGTATGTTAACGAGTTAAGAACATTATCAGGCAGCCGTTACGATCTGGTTCAGCGT
AATAACAATATTATTCTGGAGTACAAAAAGCAGGATATTCTTTCTCTGAATATTCCGCAT
GATATTAATGGTACTGAACGCAGTACGCAGAAGATTCAATTGATCGTTAAGAGCAAATAC
GGTCTGGATCGTATCGTCTGGGATGATAGTTCATTACGTAGCCAGGGCGGCCAGATTCAG
CATAGCGGAAGCCAAAGCGCACAAGATTACCAGGCTATTTTGCCTGCTTATGTGCAAGGT
GGTAGCAATGTTTATAAAGTGACGGCTCGCGCCTATGACCGTAATGGCAATAGCTCTAAC
AATGTACAGCTTACTATTACCGTTCTGTCGAATGGTCAAGTTGTCGACCAGGTTGGGGTA
ACGGACTTTACGGCGGATAAGACTTCGGCTAAAGCAGATAACACTGATACCATTACCTAT
ACCGCGATGGTTAAAAAGAATGGGGTAACTCAGGCTAATGTCCCTGTTTCATTTAATATT
GTTTCAGGAACTGCAACTCTTGGGGCAAATAGTGCCAAAACGGATGCTAACGGTAAGGCA
ACCGTAACGTTGAAGTCGAGTACACCAGGACAGGTCGTCGTGTCTGCTAAAACCGCGGA
G
ATGACTTCAGCACTTAATGCCAGTGCGGTTATATTTGTTGATCAAACCAAGGCCAGTATT
ACTGAGATTAAGGCTGATAAAACAACAGCGAAGGCAAATGGCTCTGATGCGATTACCTAT
ACTGTTAAAGTAATGAAGAATAACCAACCAGAAGTAAACCATTCTGTTACATTCTCAACA
AACTTTGGTAATCTGGGGGGGAATTCTCAGACCCAAATTGTGCAAACGGATAAAGATGGT
AAGGCTACGGTAAAACTGACATCTGGCTCGGAGGGTAGTGCTGTCGTTAGTGCAAAAGTC
AGTGAGGTTAATACAGAAGTTAAGGCATCTGAGGTAAAATTCTTCTCAGTTCTGAGTATT
GGTAATAATGTGAATATTATTGGAACCTCCGCTGATGGTGCTTTACCTAATATTTGGTTG
CAATATGGTCAGTTTAAGTTGACAGCCAAAGGCGGTGATGGAAAATATAAATGGCATTCT
AAAGATACAAGTGTTGCATCAGTTGATGCTTCTACTGGTCAGGTTACTTTGCTGAAGAAA
GGAACGACAACAATTGAAGTAGTGTCGGGCGATAACCAAACCGCGACATATACAATTAAT
CAACCTGAGAACATTATAACTGTGGAAACACAAGATAAAGTTCTCTATAACGTTGCTAAA
ACAAAATGTGAAATGAATAGTGGTCGTTTACCGTCATCTACAAGTGAGTTAAAGGATGTG
TATAACCAATGGGGACCGGCCAATAGTTATGATGGTTATAAAGGTAAAAACACAATAACA
GCATGGACACAGCAAACAGCAGATGATATACCTAAAGGTTGGACTAGTACATTTGACATA
GTTACAAAAAATGAAATTCCTAATAATGGAATTAAGGTTAAGGTCAACGTGGATGCAGCT
AACGCTTTTGCCGTTTGTGTAAAATAA
>eaeA_epsilon3_AJ876649
ATGATTACTCATGGTTTTTATGCCCGGACCCGGCACAAGCATAAGCTAAAAAAAACATTT
ATTATGCTTAGTGCTGGTTTAGGATTGTTTTTTTATGTTAACCAGAATTCATTTGCAAAC
GGTGAAAATTATTTTAAATTGAGTTCAGATTCAAAACTGTTAACTCAAAATGCCGCTCAG
GATCGCCTTTTTTATACGTTAAAAACAGGTGAAACTGTTGCCAATATTTCTAAATCACAG
GGGATCAGTTTATCGGTAATTTGGTCACTGAATAAACATTTATACAGTTCCGAAAGCGAA
ATGATGAAGGCTGGACCTGGTCAGCAGATCATTTTGCCACTCAAAAAACTGTCTGTTGAA
TATAGTGCCTTACCTGTCTTAGGTTCGGCACCTGTTGTTGCTGCAGGTGGTGTCACTGGT
CATACGAATAAAATGACTAAAATGTCCCCGGACGTGACTAAAAGCAACACGACCGATGAC
AAGGCTCTAAATTATGCGGCACAACAGGCCGCGAGCCTTGGTAGCCAGCTTCAGTCGCGC
TCATTGAACGGCGATTACGCGAAAGATACCGCTCTTGGTATGGCCAGCAGCCAGGCTTCA
TCACAGTTGCAGGCCTGGTTACAACATTATGGAACGGCAGAGGTTAATCTGCAGAGCGGT
AATAACTTTGACGGTAGTTCACTGGACTTCTTATTACCGTTCTATGATTCCGAAAATATG
CTGGCGTTTGGTCAGGTCGGGGCGCGTTACATTGACTCCCGCTTTACGGCAAATTTAGGT
GCTGGCCAGCGTTTTTTCCTTCCTGAAAATATGTTGGGCTATAACGTCTTCATTGATCAG
GATTTTTCTGGTGATAATACCCGTTTAGGTATTGGTGGCGAATACTGGCGAGACTATTTT
AAAAGTAGCGTTAACGGCTATTTCCGCATGAGCGGCTGGCATGAGTCATACAATAAGAAA
GACTATGATGAGCGCCCGGCAAATGGTTTCGATATCCGCTTTAATGGCTATCTACCATCA
TATCCGGCATTAGGCGCCAGGCTGATGTATGAGCAGTATTATGGTGATAATGTTGCTTTG
TTTAATTCCGATAAGTTGCAGTCGAATCCTGGTGCGGCGACCGTTGGTGTAAACTACACT

CCGATTCCTCTGGTGACGATGGGGATCGATTACCGTCATGGTACGGGTAATGAAAATGAT
CTCCTTTACTCAATGCAGTTCCGTTATCAGTTTGATAAACCGTGGTCTCAGCAAATCGAG
CCACAGTATGTTAACGAGTTAAGAACATTATCGGGCAGCCGTTACGATCTGGTTCAGCGT
AATAACAATATTATTCTGGAGTACAAAAAGCAGGATATTCTTTCTCTGAATATTCCGCAT
GATATTAATGGCACTGAACACAGTACGCAGAAGATTCAATTGATCGTTAAGAGCAAATAT
GGTCTGGATCGTATTGTCTGGGATGATAGTGCATTACGCAGTCAGGGCGGTCAGATTCAG
CATAGCGGAAGCCAAAGCGTACAAGACTACCAGGCTATTTTGCCTGCTTATGTGCAAGGT
GGCAGCAATATTTATAAAGTGACCGCTCGCGCCTATGACCGAAATGGTAATAGTTCTAAT
AATGTACAGCTCACTATTACCGTTTTACCGAATGGGCAGGTTGTGGACCAGGTTGGGGTA
ACGGACTTTACGGCTGATAAGACATCGGCTAAAGCGGATAACGTTGATACCATTACTTAT
ACCGCGACGGTTAAAAAGAATGGTGTAGCTCAGGCTAATGCCCCTGTAACATTTAGTATT
GTATCCGGGACTGCAACTCTTGGGGCAAATAGTGCCAGAACGGATGGTAACGGTAAGGCG
ACCGTAACGCTGAAGTCGGCTACGCCAGGACAGGTCGTCGTGTCTGCTAAAACCGCGGA
G
ATGACTTCGCCACTTAATGCCAGTGCGGTTATATTTGTTGATCAAACCAAGGCCAGCATT
ACTGAGATTAAGGCTGATAAAACAACAGCGAAGGCAAATGGTTCTGATGCGATTACCTAT
ACTGTTAAAGTAATGAAGAATAACCAACCAGAAGCAAACCATTCTGTTACATTCTCAACG
AACTTTGGTAATCTGGGGGGAAATTCTAATACCCAAATTGTGAAAACGGATAAAGATGGT
AGGGCTACGGTAAAACTGACATCTGGCGTTGCAGGTAATGCTATTGTTAGTGCAAAAGTC
AGCGAAGTTAATACAGAGGTTAAGGCTCCTGAGGTAAAATTCTTCTCAGTTCTGAGCATT
GATAGTAATGTTAATATTATTGGAACCTCTGCTACTGGCGCTTTACCTAATATTTGGTTG
CAATATGGTCAGTTCAAATTGACTGCTAAAGGTGGTGATGGGAAATATCAATGGCGCTCT
CAAGATCCAAAAGTTGCATCAGTTGATGCTTTAACTGGTCGAGTTACTTTGCTGAAGAAA
GGAACAACAAAAATTGAAGTTGTGTCTGGCGATAACCAAACCGCGACATATACAATTAAT
ACACCTGAGAAAATTATAACTGTAGAAACACAAAATAAAGTAATCTATAGCGTTGCTGAA
GCAACATGTAGCACGAATAGTGGTCGCTTACCGTCATCTACAAGTGAGCTAAAGGACGTG
TATAATAAATGGGGGGCAGCTAATCGTTATGAGGGCTATAAAGGTAAAACCACAATAACA
GCATGGACCCAACAAACAGGGGATGATAAAATTAAAGGTTGGACTAGTACATTTGATTTA
GTTACCAAAAATGAAATCCCTAGTAATGGAAGTAATAGTAAGGTCGACGTGAAAAAAGCT
AACGCTTTTGCCGTCTGTGTAAGATGA
>eaeA_epsilon4_AJ876651
ATGATTACTCATGGTTTTTATGCCCGGACCCGGCACAAGCATAAGCTAAAAAAAACATTT
ATTATGCTTAGTGCTGGTTTAGGATTGTTTTTTATGTTAACCAGAATTCATTTGCAAAC
GGTGAAAATTATTTTAAATTGAGTTCAGATTCAAAACTGTTAACTCAAAATGCCGCTCAG
GATCGCCTTTTTTATACGTTGAAAACAGGTGAAACTGTTGCCAATATTTCTAAATCACAG
GGTATCAGTTTATCGGTAATTTGGTCACTGAATAAACATTTATACAGTTCCGAAAGCGAA
ATGATGAAGGCTGGGCCTGGTCAGCAGATCGTTTTGCCACTCAAAAAACTGTCTGTTGAA
TATAGTGCCTTACCTGTCTTAGGTTCGGCACCTGTTGTTGCTGCAGGTGGTGTCGCTGGT
CATACGAATAAAATGACTAAAATGTCCCCGGACGCGACTAAAAGCAACACGACCGATGAC
AAGGCTCTAAATTATGCGGCACAACAGGCCGCGAGCCTTGGTAGCCAGCTCCAGTCGCGC
TCACTGAACGGCGATTACGCGAAAGATACCGCTCTTGGTATGGCTAGCAGCCAGGCTTCG
TCACAGTTGCAGGCCTGGTTACAACATTATGGAACGGCAGAGGTTAATCTGCAGAGTGGT
AATAACTTTGACGGTAGTTCACTGGACTTCTTATTACCTTTCTATGATTCCGAAAACATG
CTGGCATTTGGTCAGGTCGGGGCGCGTTACATTGACTCCCGCTTTACGGCAAATTTAGGT
GCTGGCCAGCGTTTTTTCCTTCCTGAAAATATGTTGGGCTATAACGTCTTCATTGATCAG
GATTTTTCTGGTGATAATACCCGTTTAGGTATTGGTGGCGAATACTGGCGAGACTATTTC
AAAAGTAGCGTTAACGGCTATTTCCGCATGAGCGGCTGGCATGAGTCATACAATAAGAAA
GACTATGATGAGCGCCCGGCAAATGGTTTTGATATCCGCTTTAATGGCTATTTACCATCA
TATCCGGCATTAGGCGCCAAACTGATGTACGAACAGTATTATGGTGATAATGTTGCTTTG
TTTAATTCCGATAAGTTGCAGTCGAATCCTGGCGCGGCGACCGTTGGTGTAAACTACACT
CCGATTCCTCTGGTGACGATGGGGATCGATTACCGTCATGGTACGGGTAATGAAAATGAT
CTCCTTTACTCAATGCAGTTCCGTTATCAGTTTGATAAACCGTGGTCTCAGCAAATCGAG
CCACAGTATGTTAACGAGTTAAGAACATTATCGGGCAGCCGTTACGATCTGGTTCAGCGT
AATAACAATATTATTCTGGAGTACAAAAAGCAGGATATTCTTTCTCTGAATATTCCGCAT
GATATTAATGGTACTGAACACAGTACGCAGAAGATTCAATTGATCGTTAAGAGCAAATAC
GGTCTGGATCGTATCGTCTGGGATGATAGCGCATTACGCAGTCAGGGCGGTCAGATTCAG
CATGGCGGAAGCCAAAGCGCACAAGACTACCAGGCTATTTTGCCTGCTTATGTGCAAGGC

GGCAGCAATATTTATAAAGTGACCGCTCGCGCCTATGACCGAAATGGTAATAGTTCTAAT
AATGTACAGCTCACTATTACCGTTTTACCGAATGGGCAGGTTGTGGACCAGGTTGGGGTA
ACGGACTTTACGGCTGATAAGACATCGGCTAAAGCGGATGGCATAGAAGCTATTACCTAT
ACCGCGACGGTTAAAAAGAATGGGGTAGCTCAGGCTAATGTCCCTGTAACATTTAGTATT
GTATCCGGGACTGCAACTCTTGGGGCAAATAGTGCCAGAACGGATGGTAACGGTAAGGCA
ACCGTAACGTTGAAGTCGGTTACGCCAGGACAGGTCGTCGTGTCTGCTAAAACCGCGGAG
ATGACTTCGCCACTTAATGCCAGTGCGGTTATATTTGTTGATCAAACCAAGGCCAGTATT
ACTGAGATTAAGGCTGATAAATCAACAGCGAAGGCAGATGGTTCTGATGCGATTACCTAT
ACTGTTAAAGTAATGAAGAATAACCAACCAGAAGCAAACCATTCTGTTACATTCTCAACG
AACTTTGGTAATCTGGGGGGAAATTCTAATACCCAAATTGTGAAAACGGATAAAGATGGT
AGGGCTACGGTAAAACTGACATCTGGCGTTGCAGGTAATGCTATTGTTAGTGCAAAAGTC
AGCGAAGTTAATACAGAGGTTAAGGCTCCTGAGGTAAAATTCTTCTCAGTTCTGAGCATT
GATAATAATGTGAATATTATTGGAACCTCCGCTAATGGTACTTTACCTAATATTTGGTTG
CGATATGGTCAGTTTAAGCTGACAGCCAAAGGTGGCGATGGGAAATATCAATGGCGCTCT
CAAGATCCAAGTGTTGCATCAGTTGATGCTTTAACTGGTCGAGTTACTTTGCTGAAGAAA
GGAACAACAAAAATTGAAGTTGTGTCTGGCGATAAGCAAACCGCGACATATACAATTAGT
ACACCTGAGAAAATTATAACTGTAGAAACACAAAATAAAGTAATCTATAGCGTTGCTGAA
GCAACATGTAGCACGAATAGTGGTCGCTTACCGTCATCTACGAGTGAGCTAAAGGATGTG
TATAATAAGTGGGGCGCTGCCAATAGTTATGAACGTTATAAAGGTAAAAATACAATAACA
GCATGGACTCAGCAAACTGAGGCTGATAAAAAATCTGGTTGGACCAGTACATTTGACATA
GTTACAAAAAATGAAATTCCTAGTAATGGAAGTAATAGTAAGGTCGATGTGAACACAGCT
AACGCTTTTGCCGTCTGTGTAAGATGA
>eaeA_eta_DQ523604
ATGATTACTCATGGTTTTTATGCCCGGACCCGGCACAAGCATAAGCTAAAAAAAACATTT
ATTATGCTTAGTGCTGGTTTAGGATTGTTTTTTTATGTTAATCAGAATTCATTTGCAAAT
GGTGAAAATTATTTTAAATTGGGTTCGGATTCAAAACTGTTAACTCATAATAGCTATCAG
AATCGCCTTTTTTATACGTTGAAAACAGGTGAAACTGTTGCCGATCTTTCTAAATCGCAA
GATATTAATTTATCGACGATTTGGTCGTTGAATAAGCATTTATACAGTTCTGAAAGCGAA
ATGATTAAGGCCGCGCCTGGTCAGCAGATCATTTTGCCACTCAAAAAACTTCCCTTTGAA
TACAGTGCCTTACCACTTTTAGGTTCGGCACCTCTTGTTGCTGCAGGTGGTGTCGCTGGT
CATACAAATAAAATGACTAAAATGTCCCCGGACGTGGCCAAAAGCAACATGACCGATGAC
AAGGCATTAAATTATGCGGCACAACAGGCGGCGAGTCTCGGTAGCCAGCTTCAGTCGCGA
TCTCTGAACGGCGATTACGCGAAAGATACCGCTCTTGGTATCGCTGGTAACCAGGCTTCG
TCACAGTTGCAGGCCTGGTTACAACATTATGGAACGGCAGAGGTTAATCTGCAGAGTGGT
AATAACTTTGACGGTAGTTCACTGGACTTCTTATTACCGTTCTATGATTCCGAAAAAATG
CTGGCATTTGGTCAGGTCGGAGCGCGTTACATTGACTCCCGCTTTACGGCAAATTTAGGT
GCGGGTCAGCGTTTTTTCCTTCCTGAAAATATGTTGGGCTATAACGTCTTCATTGATCAG
GATTTTTCTGGTGATAATACCCGTTTAGGTATTGGTGGCGAATACTGGCGAGACTATTTC
AAAAGTAGTGTTAACGGCTATTTCCGCATGAGCGGCTGGCATGAGTCATACAATAAGAAA
GACTATGATGAGCGCCCAGCAAATGGCTTCGATATCCGTTTTAATGGCTATCTGCCATCA
TACCCGGCATTAGGCGCCAGGCTGATGTATGAGCAGTATTATGGTGATAATGTCGCTTTG
TTTAATGCCGATAAACTGCAGTCGAATCCTGGCGCGGCGACCGTTGGTGTAAACTACACT
CCGGTTCCTCTGGTGACGATGGGGGTTGATTACCGTCATGGGACAGGTAATGAAAATGAT
CTCCTTTACTCAATGCAGTTCCGTTATCAATTTGACAAACCGTGGTCTCAGCAAATTGAG
CCGCAGTATGTTAACGAGTTAAGAACATTATCAGGCAGCCGTTACGATCTGGTACAACGT
AATAACAATATTATTCTGGAATACAAAAAGCAGGATATTCTTTCTTTGAGTATTCCACAT
GATATTAATGGTACTGAACACAGTACGCAGAAGATTCAATTGAACGTTAAGAGTAAGTAC
GGCCTGGATCGTATCGTCTGGGATGACAGTGCATTACGCAGTCAGGGCGGTCAGATTCAG
CATAGCGGAAGCCAAAGCGTACAAGATTACCAGGCTATTTTGCCTGCTTATGTGCAAGGT
GGTAGCAATATTTATAAAGTGACCGCTCGCGCCTATGACCGTAATGGCAATAGCTCTAAC
AATGTGCAGCTCACTATTACCGTTCTGTCGAATGGTCAGGTTGTCGGCCAGGTTGGGGTA
ACGGACTTTACGGCTGATAAGACATCGGCTAAAGCGGATGGCACCGAAGCTATTACTTAT
ACCGCGACGGTTAAAAAGAATGGGGTTGCCCAGGCTAATGTTCCTGTTTCATTTGATATT
GTTTCAGGTGATGCAACTTTAAGTGCCAGGAGTGCCACTACTAATAGCAGCGGTAAGGCG
ACCGTAACGCTGAAATCAAGTAAGCCGGGCCAGGTTGTTGTGTCAGCTAAAACAGCGGA
G
ATGACTTCAGCGCTTAATGCCAATGCGGTTATATTTGTTGATCAAACCAAGGCCAGCATT

ACTGAGATTAAGGCTGATAAGACAACAGCGAAGGCAGATGGTTCTGATGCGATTACCTAT
ACTGTTAAAGTAATGAAGAATAACCAACCAGAAGCAAACCATTCTGTTACATTCTCAACG
AACTTTGGTGATCTGGGGGGAAATTCTAATACCCAAATTGTGAAAACGGATAAAGATGGT
AGGGCTACGGTAAAACTGACATCTGGCGTTGCAGGTAATGCTATTGTTAGTGCAAAAGTC
AGCGAAGTTAATACAGAGGTTAAGGCTCCTGAGGCAAAATTCTTCTCAGTTCTGAGCATT
GATAATAATGTGAATATTATTGGAACCTCCGCTAATGGTGCTTTACCTAATATTTGGTTG
CGATATGGTCAGTTTAAGCTGACAGCCAAAGGTGGCGATGGGAAATATCAATGGCGCTCT
CAAGATCCAAGTGTTGCATCAGTTGATGCTTTAACTGGTCGAGTTACTTTGCTGAAGAAA
GGAACAACAAAAATTGAAGTTGTGTCTGGCGATAAGCAAACCGCGACATATACAATTAAT
ACACCTGAGAAAATTATAACTGTAGAAACACAAAATAAAGTAATCTATAGCGTTGCTGAA
GCAACATGCAGCACGAATAGTGGTCGCTTACCGTCATCTACGAGTGAGCTAAAGGATGTG
TATAATAAGTGGGGCGCTGCCAATAGTTATGAAGGTTATAAAGGTAAAAATACAATAACA
GCATGGACTCAGCAAACTGCGGCTGATAAACAATCTGGTTGGACTAGTACATTTGACATA
GTTACAAAAAATGAAATTCCTAATAATGGAAGTAATAGTAAGGTCAATGTGAACACAGCT
AACGCTTTTGCCGTCTGTGTAAGATGA
>eaeA_eta2_AJ876652
ATGATTACTCATGGTTTTTATGCCCGGACCCGGCACAAGCATAAGCTAAAAAAAACATTT
ATTATGCTTAGTGCTGGTTTAGGATTGTTTTTTTATGTTAATCAGAATTCATTTGCAAAT
GGTGAAAATTATTTTAAATTGGGTTCGGATTCAAAACTGTTAACTCATAATAGCTATCAG
AATCGCCTTTTTTATACGTTGAAAACAGGTGAAACTGTTGCCGATCTTTCTAAATCGCAA
GATATTAATTTATCGACGATTTGGTCGTTGAATAAGCATTTATACAGTTCTGAAAGCGAA
ATGATGAAGGCCGCGCCAGGTCAGCAGATCATTTTGCCACTCAAAAAACTTCCCTTTGAA
TACAGTGCCTTACCACTTTTAGGTTCGGCACCTCTTGTTGCTGCAGGTGGTGTCGCTGGT
CATACAAATAAAATGACTAAAATGTCCCCGGACGTGGCCAAAAGCAACATGACCGATGAC
AAGGCATTAAATTATGCGGCACAACAGGCGGCGAGTCTCGGTAGCCAGCTTCAGTCGCGA
TCTCTGAACGGCGATTACGCGAAAGATACCGCTCTTGGTATCGCTGGTAACCAGGCTTCG
TCACAGTTGCAGGCCTGGTTACAACATTATGGAACGGCAGAGGTTAATCTGCAGAGTGGT
AATAACTTTGACGGTAGTTCACTGGACTTCTTATTACCGTTCTATGATTCCGAAAAAATG
CTGGCATTTGGTCAGGTCGGAGCGCGTTACATTGACTCCCGCTTTACGGCAAATTTAGGT
GCGGGTCAGCGTTTTTTCCTTCCTGAAAATATGTTGGGCTATAACGTCTTCATTGATCAG
GATTTTTCTGGTGATAATACCCGTTTAGGTATTGGTGGCGAATACTGGCGAGACTATTTC
AAAAGTAGTGTTAACGGCTATTTCCGCATGAGCGGCTGGCATGAGTCATACAATAAGAAA
GACTATGATGAGCGCCCAGCAAATGGCTTCGATATCCGTTTTAATGGCTATCTGCCATCA
TACCCGGCATTAGGTGCCAAGCTGATGTATGAGCAGTATTATGGTGATAATGTTGCTTTG
TTTAATTCTGATAAGCTGCAGTCGAATCCTGGTGCGGCGACCGTTGGTGTAAACTATACT
CCGATTCCTCTGGTGACGATGGGGATCGATTACCGTCATGGTACGGGTAATGAAAATGAT
CTCCTTTACTCAATGCAGTTCCGTTATCAGTTTGATAAACCGTGGTCTCAGCAAATTGAG
CCACAATATGTTAACGAGTTAAGAACATTATCAGGCAGCCGTTACGATCTGGTTCAGCGT
AATAACAATATTATTCTGGAGTACAAAAAGCAGGATATTCTTTCTCTGAATATTCCGCAT
GATATTAATGGTACTGAACGCAGTACGCAGAAGATTCAATTGATCGTTAAGAGCAAATAC
GGTCTGGATCGTATCGTCTGGGATGATAGTTCATTACGTAGCCAGGGCGGCCAGATTCAG
CATAGCGGAAGCCAAAGCGCACAAGATTACCAGGCTATTTTGCCTGCTTATGTGCAAGGT
GGTAGCAATGTTTATAAAGTGACGGCTCGCGCCTATGACCGTAATGGCAATAGCTCTAAC
AATGTACAGCTTACTATTACCGTTCTGTCGAATGGTCAGGTGGTCGACCAGGTTGGGGTA
ACGGACTTTACGGCTGATAAGACATCGGCTAAAGCGGATGGCACCGAAGCTATTACTTAT
ACCGCGACGGTTAAAAAGAATGGGGTTGCCCAGGCTAATGTTCCTGTTTCATTTGATATT
GTTTCAGGTGATGCAACTTTAAGTGCCAGGAGTGCCACTACTAATAGCAGCGGTAAGGCG
ACCGTAACGCTGAAATCAATTAAGCCGGGCCAGGTTGTTGTGTCAGCTAAAACAGCGGAG
ATGACTTCAGCGCTTAATGCCAATGCGGTTATATTTGTTGATCAAACCAAGGCCAGCATT
ACTGAGATTAAGGCTGATAAGACAACAGCGAAGGCAGATGGTTCTGATGCGATTACCTAT
ACTGTTAAAGTAATGAAGAATAACCAACCAGAAGCAAACCATTCTGTTACATTCTCAACG
AACTTTGGTGATCTGGGGGGAAATTCTAATACCCAAATTGTGAAAACGGATAAAGATGGT
AGGGCTACGGTAAAACTGACATCTGGCGTTGCAGGTAATGCTATTGTTAGTGCAAAAGTC
AGCGAAGTTAATACAGAGGTTAAGGCTCCTGAGGCAAAATTCTTCTCAGTTCTGAGCATT
GATAATAATGTGAATATTATTGGAACCTCCGCTAATGGTGCTTTACCTAATATTTGGTTG
CGATATGGTCAGTTTAAGCTGACAGCCAAAGGTGGCGATGGGAAATATCAATGGCGCTCT
CAAGATCCAAGTGTTGCATCAGTTGATGCTTTAACTGGTCGAGTTACTTTGCTGAAGAAA

GGAACAACAAAAATTGAAGTTGTGTCTGGCGATAAGCAAACCGCGACATATACAATTAAT
ACACCTGAGAAAATTATAACTGTAGAAACACAAAATAAAGTAATCTATAGCGTTGCTGAA
GCAACATGCAGCACGAATAGTGGTCGCTTACCGTCATCTACGAGTGAGCTAAAGGATGTG
TATAATAAGTGGGGCGCTGCCAATAGTTATGAAGGTTATAAAGGTAAAAATACAATAACA
GCATGGACTCAGCAAACTGCGGCTGATAAACAATCTGGTTGGACTAGTACATTTGACATA
GTTACAAAAAATGAAATTCCTAATAATGGAAGTAATAGTAAGGTCAATGTGAACACAGCT
AACGCTTTTGCCGTCTGTGTAAGATGA
>eaeA_gamma1_NC002695
ATGATTACTCATGGTTGTTATACCCGGACCCGGCACAAGCATAAGCTAAAAAAAACATTG
ATTATGCTTAGTGCTGGTTTAGGATTGTTTTTTATGTTAATCAGAATTCATTTGCAAAT
GGTGAAAATTATTTAAATTGGGTTCGGATTCAAAACTGTTAACTCATGATAGCTATCAG
AATCGCCTTTTTTATACGTTGAAAACTGGTGAAACTGTTGCCGATCTTTCTAAATCGCAA
GATATTAATTTATCGACGATTTGGTCGTTGAATAAGCATTTATACAGTTCTGAAAGCGAA
ATGATGAAGGCCGCGCCTGGTCAGCAGATCATTTTGCCACTCAAAAAACTTCCCTTTGAA
TACAGTGCACTACCACTTTTAGGTTCGGCACCTCTTGTTGCTGCAGGTGGTGTTGCTGGT
CACACGAATAAACTGACTAAAATGTCCCCGGACGTGACCAAAAGCAACATGACCGATGAC
AAGGCATTAAATTATGCGGCACAACAGGCGGCGAGTCTCGGTAGCCAGCTTCAGTCGCGA
TCTCTGAACGGCGATTACGCGAAAGATACCGCTCTTGGTATCGCTGGTAACCAGGCTTCG
TCACAGTTGCAGGCCTGGTTACAACATTATGGAACGGCAGAGGTTAATCTGCAGAGTGGT
AATAACTTTGACGGTAGTTCACTGGACTTCTTATTACCGTTCTATGATTCCGAAAAAATG
CTGGCATTTGGTCAGGTCGGAGCGCGTTACATTGACTCCCGCTTTACGGCAAATTTAGGT
GCGGGTCAGCGTTTTTTCCTTCCTGCAAACATGTTGGGCTATAACGTCTTCATTGATCAG
GATTTTTCTGGTGATAATACCCGTTTAGGTATTGGTGGCGAATACTGGCGAGACTATTTC
AAAAGTAGCGTTAACGGCTATTTCCGCATGAGCGGCTGGCATGAGTCATACAATAAGAAA
GACTATGATGAGCGCCCAGCAAATGGCTTCGATATCCGTTTTAATGGCTATCTACCGTCA
TATCCGGCATTAGGCGCCAAGCTGATATATGAGCAGTATTATGGTGATAATGTTGCTTTG
TTTAATTCTGATAAGCTGCAGTCGAATCCTGGTGCGGCGACCGTTGGTGTAAACTATACT
CCGATTCCTCTGGTGACGATGGGGATCGATTACCGTCATGGTACGGGTAATGAAAATGAT
CTCCTTTACTCAATGCAGTTCCGTTATCAGTTTGATAAATCGTGGTCTCAGCAAATTGAA
CCACAGTATGTTAACGAGTTAAGAACATTATCAGGCAGCCGTTACGATCTGGTTCAGCGT
AATAACAATATTATTCTGGAGTACAAGAAGCAGGATATTCTTTCTCTGAATATTCCGCAT
GATATTAATGGTACTGAACACAGTACGCAGAAGATTCAGTTGATCGTTAAGAGCAAATAC
GGTCTGGATCGTATCGTCTGGGATGATAGTGCATTACGCAGTCAGGGCGGTCAGATTCAG
CATAGCGGAAGCCAAAGCGCACAAGACTACCAGGCTATTTTGCCTGCTTATGTGCAAGGT
GGCAGCAATATTTATAAAGTGACGGCTCGCGCCTATGACCGTAATGGCAATAGCTCTAAC
AATGTACAGCTTACTATTACCGTTCTGTCGAATGGTCAAGTTGTCGACCAGGTTGGGGTA
ACGGACTTTACGGCGGATAAGACTTCGGCTAAAGCGGATAACGCCGATACCATTACTTAT
ACCGCGACGGTGAAAAAGAATGGGGTAGCTCAGGCTAATGTCCCTGTTTCATTTAATATT
GTTTCAGGAACTGCAACTCTTGGGGCAAATAGTGCCAAAACGGATGCTAACGGTAAGGCA
ACCGTAACGTTGAAGTCGAGTACGCCAGGACAGGTCGTCGTGTCTGCTAAAACCGCGGA
G
ATGACTTCAGCACTTAATGCCAGTGCGGTTATATTTTTTGATCAAACCAAGGCCAGCATT
ACTGAGATTAAGGCTGATAAGACAACTGCAGTAGCAAATGGTAAGGATGCTATTAAATAT
ACTGTAAAAGTTATGAAAAACGGTCAGCCAGTTAATAATCAATCCGTTACATTCTCAACA
AACTTTGGGATGTTCAACGGTAAGTCTCAAACGCAAGCAACCACGGGAAATGATGGTCGT
GCGACGATAACACTAACTTCCAGTTCCGCCGGTAAAGCGACTGTTAGTGCGACAGTCAGT
GATGGGGCTGAGGTTAAAGCGACTGAGGTCACTTTTTTTGATGAACTGAAAATTGACAAC
AAGGTTGATATTATTGGTAACAATGTCAGAGGCGAGTTGCCTAATATTTGGCTGCAATAT
GGTCAGTTTAAACTGAAAGCAAGCGGTGGTGATGGTACATATTCATGGTATTCAGAAAAT
ACCAGTATCGCGACTGTCGATGCATCAGGGAAAGTCACTTTGAATGGTAAAGGCAGTGTC
GTAATTAAAGCCACATCTGGTGATAAGCAAACAGTAAGTTACACTATAAAAGCACCGTCG
TATATGATAAAAGTGGATAAGCAAGCCTATTATGCTGATGCTATGTCCATTTGCAAAAAT
TTATTACCATCCACACAGACGGTATTGTCAGATATTTATGACTCATGGGGGGCTGCAAAT
AAATATAGCCATTATAGTTCTATGAACTCAATAACTGCTTGGATTAAACAGACATCTAGT
GAGCAGCGTTCTGGAGTATCAAGCACTTATAACCTAATAACACAAAACCCTCTTCCTGGG
GTTAATGTTAATACTCCAAATGTCTATGCGGTTTGTGTAGAATAA
>eaeA_gamma2_AF025311

ATGATTACTCATGGTTTTTATGCCCGGACCCGGCACAAGCATAAGCTAAAAAAAACATTT
ATTATGCTTAGCGCTGGTTTAGGATTGTTTTTTATGTTAACCAGAACTCATTTGCAAAC
GGTGAAAATTATTTTAAATTGAGTTCAGATTCAAAACTGTTAACTCAAAATGTTGCTCAG
GATCGCCTTTTTTATACGTTGAAAACAGGTGAAACTGTTTCCAGTATTTCTAAATCACAA
GGTATCAGTTTATCCGTAATTTGGTCACTGAATAAACATTTATACAGTTCCGAAAGCGAA
ATGCTGAAGGCTGCGCCTGGCCAGCAGATCATTTTGCCACTCAAAAAACTGTCTGTTGAA
TATGGTGCCTTACCTGTCTTAGGTTCGGCACCTGTTGTTGCTGCAGGTGGTGTCGCTGGG
CATACAAATAAAATGACTAAAATGTCCCCGGACGCGACTCAAAGCAACATGACTGATGAC
AGGGCTCTAAATTATACGGCACAACAGGCCGCGAGCCTTGGTAGCCAGCTTCAGTCGCGC
TCTCTGCACGGCGATTACGCGAAAGATACCGCTCTTGGTATCGCGGGTAACCAGGCTTCG
TCACAGTTGCAGGCCTGGTTACAACATTATGGAACGGCAGAGGTTAATCTGCAGAGTGGT
AATAACTTTGACGGTAGTTCACTGGATTTCTTATTACCGTTCTATGATTCCGAAAAAATG
CTGGCATTTGGTCAGGTCGGAGCGCGTTACATTGACTCCCGCTTTACGGCAAATTTAGGT
GCGGGTCAGCGTTTTTTCCTTCCTGAAAACATGTTGGGCTATAACGTCTTCATTGATCAG
GATTTTTCTGGTGATAATACCCGTTTAGGTATTGGTGGCGAATACTGGCGAGACTATTTC
AAAAGTAGCGTTAACGGCTATTTCCGCATGAGCGGCTGGCATGAGTCATACAATAAGAAA
GACTATGATGAGCGCCCAGCAAATGGCTTCGATATCCGCTTTAATGGCTATCTACCATCA
TACCCGGCATTAGGCGCCAAGCTGATGTATGAGCAGTATTATGGTGATAATGTTGCTTTG
TTTAATTCCGATAAGCTGCAGTCGAATCCTGGTGCGGCGACCGTTGGTGTTAACTATACT
CCGATTCCTCTGGTGACGATGGGGATCGATTACCGTCATGGTACGGGTTATGAAAATGAT
CTTCTTTACTCAATGCAGTTCCGTTATCAGTTTGATAAACCGTGGTCTCCGCAAATTGAA
CCACAGTATGTTAACGAGTTAAGAACATTATCAGGCAGCCGTTACGATCTGGTTCAGCGT
AATAACAATATTATTCTGGAGTACAAGAAGCAGGATATTCTTTCTCTGAATATTCCGCAT
GATATTAATGGTACTGAACACAGTACGCAGAAGATTCAATTGATCGTTAAGAGCAAATAC
GGTCTGGATCGTATCGTCTGGGATGATAGTGCATTACGTAGCCAGGGCGGTCAGATTCAG
CATAGCGGAAGCCAAAGCGCACAAGATTACCAGGCTATTTTGCCGGCTTATGTGCAAGGT
GGTAGCAATATTTATAAAGTGACGGCTCGTGCCTATGACCGTAATGGCAATAGCTCTAAC
AATGTACAGCTCACTATTACCGTTCTGTCGAATGGTCAGGTGGTCGACCAGGTTGGGGTA
ACGGACTTTACGGCTGATAAGACTTCGGCTAAAGCGGATGGCACCGAGGCGATTACTTAT
ACCGCGACGGTGAAAAAGAATGGGGTAACTCAGGCTAATGTCCCTGTTTCATTTAATATT
GTTTCAGGAACTGCAACTCTTGGGGCAAATAGTGCCACAACGGATGCTAACGGTAAGGCA
ACTGTAACGTTGAAGTCGAGTACGCCAGGGCAGGTAGTCGTGTCTGCTAAAACCGCGGAG
ATGACTTCAGCACTTAATGCCAGTGCGGTTATATTTGTTGAGCAAACCAAGGCCAGTATT
ACTGAGATTAAGGCTGATAAGACAACTGCAGTAGCAAATGGTAATGATGCTGTTACATAC
ACTGTTAAAGTGATGAAAGAGGGTCAGCCAGTGCATGGACACTCCGTTGCATTCACAACA
AACTTTGGGATGTTCAACGGTAAGTCTCAGACGCAAAATGCGACCACGGGAAGTGATGGT
CGTGCGACGATAACACTGACTTCCAGTTCCGCAGGTAAAGCGACTGTTAGTGCGACTGTT
AGTGGTGGGAATGATGTTAAAGCACCTGAGGTTACATTTTTTGATGGACTGAAAATTGAC
AACAAGGTTGATATTCTTGGTAAGAACGTTACTGGTGACTTACCTAATATCTGGTTGCAA
TATGGTCAGTTTAAACTGAAGGTAAGCGGTGGTAATGGTACATATTCATGGCATTCAGAG
AATACCAATATTGCGACTGTTGATGAATCAGGGAAAGTAACCTTGAAAGGAAAAGGTACT
GCAGTAATTAATGTTACATCTGGTGATAAGCAAACAGTAAGCTACACTATTAAAGCTCCG
AATTATATGATAAGAGTGGGTAATAAAGCCAGTTATGCAAATGCTATGTCCTTTTGTGGA
AATTTATTACCATCCTCACAGACGGTATTATCAAACGTTTATAATTCATGGGGGCCTGCA
AACGGATATGACCATTATCGTTCTATGCAGTCAATAACAGCTTGGATTACACAAACTGAA
GCTGATAAAATATCAGGAGTATCAACTACTTATGACTTAATAACACAAAACCCTCATAAG
GATGTTTCGCTAAACGCTCCAAATGTCTATGCAGTTTGTGTAGAATAA
>eaeA_iota1_DQ523601
ATGATTACTCATGGTTTTTATGCCCGGACCCGGCACAAGCATAAGCTAAAAAAAACATTT
ATTATGCTTAGTGCTGGTTTAGGATTGTTTTTTATGTTAATCAGAATTCATTTGCAAAT
GGTGAAAATTATTTTAAATTGGGTTCGGATTCAAAACTGTTAACTCATAATAGCTATCAG
AATCGCCTTTTTTATACGTTGAAAACAGGTGAAACTGTTGCCGATCTTTCTAAATCGCAA
GATATTAATTTATCGACGATTTGGTCGTTGAATAAGCATTTATACAGTTCTGAAAGCGAA
ATGATGAAGGCCGCGCCTGGTCAGCAGATCATTTTGCCACTCAAAAAACTTCCCTTTGAA
TACAGTGCCTTACCACTTTTAGGTTCGGCACCTCTTGTTGCTGCAGGTGGTGTCGCTGGT
CATACAAATAAACTGACTAAAATGTCCCCGGACGTGACCAAAAGCAACATGACCGATGAC
AAGGCATTAAATTATGCGGCACAACAGGCGGCGAGTCTCGGTAGCCAGCTTCAGTCGCGA

TCTCTGAACGGCGATTACGCGAAAGATACCGCTCTTGGTATCGCTGGTAACCAGGCTTCG
TCACAGTTGCAGGCCTGGTTACAACATTATGGAACGGCAGAGGTTAATCTGCAGAGTGGT
AATAACTTTGACGGTAGTTCACTGGACTTCTTATTACCGTTCTATGATTCCGAAAAAATG
CTGGCATTTGGTCAGGTCGGAGCGCGTTACATTGACTCCCGCTTTACGGCAAATTTAGGT
GCGGGTCAGCGTTTTTTCCTTCCTGAAAATATGTTGGGCTATAACGTCTTCATTGATCAG
GATTTTTCTGGTGATAATACCCGTTTAGGTATTGGTGGCGAATACTGGCGAGACTATTTC
AAAAGTAGTGTTAACGGCTATTTCCGCATGAGCGGCTGGCATGAGTCATACAATAAGAAA
GACTATGATGAGCGCCCAGCAAATGGCTTCGATATCCGTTTTAATGGCTATCTGCCATCA
TACCCGGCATTAGGTGCCAGGCTGATGTATGAGCAGTATTATGGTGATAATGTTGCTTTG
TTTAATTCTGATAAGCTGCAGTCGAATCCTGGTGCGGCGACCGTTGGTGTAAACTATACT
CCGATTCCTCTGGTGACGATGGGGATCGATTACCGTCATGGTACGGGTAATGAAAATGAT
CTCCTTTACTCAATGCAGTTCCGTTATCAGTTTGATAAACCGTGGTCTCAGCAAATTGAG
CCACAGTATGTTAACGAGTTAAGAACATTATCAGGCAGCCGTTACGATCTGGTTCAGCGT
AATAACAATATTATTCTGGAGTACAAAAAGCAGGATATTCTTTCTCTGAATATTCCGCAT
GATATTAATGGTACTGAACGCAGTACGCAGAAGATTCAATTGATCGTTAAGAGCAAATAC
GGTCTGGATCGTATCGTCTGGGATGATAGTTCATTACGTAGCCAGGGCGGCCAGATTCAG
CATAGCGGAAGCCAAAGCGCACAAGATTACCAGGCTATTTTGCCTGCTTATGTGCAAGGT
GGTAGCAATGTTTATAAAGTGACGGCTCGCGCCTATGACCGTAATGGCAATAGCTCTAAC
AATGTACAGCTTACTATTACCGTTCTGTCGAATGGTCAGGTGGTCGACCAGGTTGGGGTA
ACGGACTTTACGGCTGATAAGACTTCGGCTAAAGCGGATGGCACCGAAGCCATTACTTAT
ACTGCGACGGTGAAAAAGAATGGGGTAGCTCAGGCTAATGTCCCTGTTTCATTTAATATT
GTTTCAGGAACTGCAACTCTTGGGGCAAATAGTGCCAAAACGGATGCTAACGGTAAGGCA
ACCGTAACGTTGAAGTCGAGTACGCCAGGACAGGTAGTCGTGTCTGCTAAAACCGCAGAG
ATGACTTCAGCACTTAATGCCAGTGCGGTTATATTTGTTGATCAAACCAAGGCCAGCATT
ACTGAGATTAAGGCTGATAAGACAACTGCAGTAGCAAATGGTAAGGATGCTGTCACATAC
ACTGTTAAAGTGATGAAAAATGGGCTGCCGGAAAAGGGACACGTAGTTACATTCTCAACT
GATTTAGGTAAATTGAATCTACAGACTGTAGCAACGGATAAAGATGGTTTCGCCTCGGTA
ACATTGACTTCTGATTCTGTCGGTAAAGCAGTTGTTAGTGCAAAAGTTAGTGAAGCTGGC
TCAGTGGTTAATGCAGATGCAGTTAACTTCTTCGCTACACTTAGTATTGATAATAATGTC
GAGATCGTTGGTACAAAAGTTCGTGGTGAATTACCAAATATCTGGCTACGATATGGCCAA
GTTAAGTTGAATGCAAATGGCGGTAATGGTGGATACTCATGGAGTTCCGATAATCCAGAT
ATTGCATCGATAGATGCTAATACAGGTATTATTACCTTAAATAAAAAAGGAACTACAGTT
ATTAAAGTCATATCTGGTGATAAACAGATAGCAACGTATACGATTAAGACACCTCAAGAA
ATAGTATCTTTAGATAATAGTGTAAAAGTTAAATATGACGAGGCTAGTGGTATATGTTCA
AATAATGCTGCATATTTATCTGTCTCAGTAGACAGTTTGAAAAAATTATATAGTCAATGG
GGGCCAGCAAATAAATATAGCCATTATACTCAAGGAACCATAAATGCTTGGATACAGCAA
ACGGAGCAGGATAAAAAAGACAGCGTCGCAACGACATATGATATCGTCACTGATAATACG
GTAATAAATGTTGATTCAACAGTGGCTAACGCTTATGCAGTTTGCATAAAATAA
>eaeA_iota2_AY696842
ATGATTACTCATGGTTGTTATACCCGGACCCGGCACAAGCATAAGCTAAAAAAAACATTT
GTTATGCTTAGCGCTGGTTTAGGATTGTTTTTTTATGTTAACCAGAACTCATTTGCAAAC
GGTGAAAACTATTTTAAACTGAGTTCAGATTCTAAACTATTAACTCAAAATGCCGCTCAG
AATCGCCTTTTTTATACGTTGAAAACAGGTGAAACTGTTGCCGATCTTTCTAAATCGCAA
AATATTAATTTATCGACGATTTGGTCGCTGAATAAGCATTTATACAGTTCCGAAAGCGAA
ATGATGAAGGCTGCACCTGGTCAGCAGATCATTTTGCCACTCAAAAAACTTCCTGTTGAA
TATAGCGCCTTACCACACTTAGGTTCGGCGCCTGTCGTTGCAGCAGGTGGTGTTGCTGGT
CATACGAATAAAATGGCTAAAATGTCCCCGGACATGACCAAAAGCAACATGACCGATGAC
AAGGCGCTAAATTATGCGGCACAACAGGCTGCGAGTCTCGGTAGCCAACTTCAGTCTCGC
TCACTGAACGGCGATTACGCAAAAGATGCTGCCCTTGGTATGGCCGGCAACCAGGCTTCG
TCACAGTTGCAGGCCTGGTTACAACATTATGGAACGGCAGAGGTTAATCTGCAGAGCGGT
AATAACTTTGATGGCAGTTCGCTGGACTTCTTATTACCGTTCTATGATACCGAAGAGATG
CTGGCATTTGGTCAGGTCGGAGCGCGTTACATTGACTCCCGCTTTACGGCAAATTTAGGT
GCTGGTCAGCGCTTCTTCCTTTCTGAAAATATGCTGGGCTATAACGTCTTCATTGATCAG
GATTTTTCTGGTGATAATACCCGTTTAGGTATTGGTGGCGAATACTGGCGAGACTATTTC
AAAAGTAGCGTGAACGGCTATTTCCGCATGAGCGGTTGGCATGAGTCATACAACAAGAAA
GACTATGACGAGCGCCCGGTAAATGGTTTCGATATCCGCTTTAATGGGTATTTACCATCA
TATCCGGCATTAGGCGCCAAACTGATGTACGAACAGTATTATGGTGATAATGTCGCTTTG

TTTAATGCCGATAAACTGCAATCGAATCCTGGCGCGGCGACCGTTGGTGTAAACTACACT
CCGGTTCCTCTGGTGACGATGGGGGTTGATTACCGTCATGGGACAGGTAATGAAAATGAT
CTCCTTTACTCAATGCAGTTCCGTTATCAATTTGACAAACCGTGGTCTCAGCAAATTGAG
CCGCAGTATGTTAACGAGTTAAGAACATTATCAGGCAGCCGTTACGATCTGGTACAACGT
AATAACAATATTATTCTGGAATACAAAAAGCAGGATATTCTTTCTTTGAGTATTCCACAT
GATATTAATGGTACTGAACACAGTACGCAGAAGATTCAATTGAACGTTAAGAGTAAGTAC
GGCCTGGATCGTATCGTCTGGGATGACAGTGCATTACGCAGTCAGGGCGGTCAGATTCAG
CATAGCGGAAGCCAAAGCGCACAAGATTACCAGGCTATTTTGCCTGCTTATGTGCAAGGT
GGTAGCAATATTTATAAAGTGACCGCTCGCGCCTATGACCGTAATGGCAATAGCTCTAAC
AATGTGCAGCTCACTATTACCGTTCTGTCGAATGGTCAGGTTGTCGGCCAGGTTGGGGTA
ACGGACTTTACGGCTGATAAGACATCGGCTAAAGCGGATGGCACCGAAGCTATTACTTAT
ACCGCGACGGTTAAAAAGAATGGGGTTGCCCAGGCTAATGTTCCTGTTTCATTTGATATT
GTTTCAGGTGTGGGAAAACTTGGCTCTGAAAGAGTTAATACAGATAATACTGGTAAAGCG
ATAGTCAAACTTACTTCGACTATTCCGGGTCAGGTGGTTATATCGGCTAAAACAGCTGAA
ATGGCTTCCGCGAAAAATGCTAGTGCAGTTATATTTATCGACCAAAGTCAAGCTAGTATT
ACTGAGATTAAAGCTAGTAAGACCACCGCAAAGGCGGATGGTGTTGATGCTATTTTATAC
ACAGTTAAAGTAATGAAAAATGGAGTCCCAGAAAAGGGACAGGTAGTTGCATTCTCGACT
AATTTAGGTAAGTTGAATTTACAGACGGTAGAAACGAATAAAGATGGTCTTGCCTCGGTA
ACTTTAACTTCTGTTTCCGTCGGTAAAGCAGTCGTTAGTGCAAAAGTCAGTGAAGCGGGC
TCTGTAATTAATGCTGATGCAGTTAACTTCTTCGCTACACTTAATATTGATAAGAATGTA
GAGATTGTTGGTACAAAAGTTAGCGGTGAATTACCAAATATCTGGCTACAATATGGTCAG
GTTAAGTTAAATGCAAATGGCGGTAATGGAGGATATACATGGAGTTCCGATAATCCAAAT
ATTGCTTCGATAGATTCTAATACAGGCATTATTACCTTGAATAAAAAAGGAACTGCAGTT
ATTAAAGTCGTATCTGGTGATAAACAGATAGCAACGTATACGATTAAGACACCTCAGGAA
ATAGTATCTTTAGATAAGAATGGAAAAGTTACATATGATGAGGCCAATAGTATATGTTTA
GGAAATAGTGCTCATTTATCTGCCTCAGTTGACACTTTGAAAAAAGTATATAGTCAATGG
GGACCTGCAAGTAAATATGAACATTATACTCAAAGGACTATAAACGCTTGGATACAGCAA
ACTGATAAGGATAAAAGAGAAGGCGTCGCAACTACATATGATATCGTCACTAATAATATG
GTGCCAAATGTTTCTTCAAAAACGCCTAACGCTTATGCAGTTTGTGTAAAATAA
>eaeA_kappa_DQ523611
ATGATTACTCATGGTTTTTATGCCCGGACCCGGCACAAGCATAAGCTAAAAAAAACATTT
ATTATGCTTAGTGCTGGTTTAGGATTGTTTTTTATGTTAATCAGAATTCATTTGCAAAT
GGTGAAAATTATTTTAAATTGGGTTCGGATTCAAAACTGTTAACTCATAATAGCTATCAG
AATCGCCTTTTTTATACGTTGAAAACAGGTGAAACTGTTGCCGATCTTTCTAAATTGCAA
GATATTAATTTATCGACGATTTGGTCGTTGAATAAGCATTTATACAGTTCTGAAAGCGAA
ATGATGAAGGCCGCGCCTGGTCAGCAGATCATTTTGCCACTCAAAAAACTTCCCTTTGAA
TACAGTGCCTTACCACTTTTAGGTTCGGCACCTCTTGTTGCTGCAGGTGGTGTCGCTGGT
CATACAAATAAACTGACTAAAATGTCCCCGGACGTGACCAAAAGCAACATGACCGATGAC
AAGGCATTAAATTATGCGGCACAACAGGCGGCGAGTCTCGGTAGCCAGCTTCAGTCACGA
TCTCTGAACGGCGATTACGCGAAAGATACCGCTCTTGGTATCGCTGGTAACCAGGCTTCG
TCACAGTTGCAGGCCTGGTTACAACATTATGGAACGGCAGAGGTTAATCTGCAGAGTGGT
AATAACTTTGACGGTAGTTCACTGGACTTCTTATTACCGTTCTATGATTCCGAAAAAATG
CTGGCATTTGGTCAGGTCGGAGCGCGTTACATTGACTCCCGCTTTACGGCAAATTTAGGT
GCGGGTCAGCGTTTTTTCCTTCCTGAAAATATGTTGGGCTATAACGTCTTCATTGATCAG
GATTTTTCTGGTGATAATACCCGTTTAGGTATTGGTGGCGAATACTGGCGAGACTATTTC
AAAAGTAGTGTTAACGGCTATTTCCGCATGAGCGGCTGGCATGAGTCATACAATAAGAAA
GACTATGATGAGCGCCCAGCAAATGGCTTCGATATCCGTTTTAATGGCTATCTGCCATCA
TACCCGGCATTAGGTGCCAAGCTGATGTATGAGCAGTATTATGGTGATAATGTTGCTTTG
TTTAATTCTGATAAGCTGCAGTCGAATCCTGGTGCGGCGACCGTTGGTGTAAACTATACT
CCGATTCCTCTGGTGACGATGGGGATCGATTACCGTCATGGTACGGGTAATGAAAATGAT
CTCCTTTACTCAATGCAGTTCCGTTATCAGTTTGATAAACCGTGGTCTCAGCAAATTGAG
CCACAATATGTTAACGAGTTAAGAACATTATCAGGCAGCCGTTACGATCTGGTTCAGCGT
AATAACAATATTATTCTGGAGTACAAAAAGCAGGATATTCTTTCTCTGAATATTCCGCAT
GATATTAATGGTACTGAACGCAGTACGCAGAAGATTCAATTGATCGTTAAGAGCAAATAC
GGTCTGGATCGTATCGTCTGGGATGATAGTTCATTACGTAGCCAGGGCGGCCAGATTCAG
CATAGCGGAAGCCAAAGCGCACAAGATTACCAGGCTATTTTGCCTGCTTATGTGCAAGGT
GGTAGCAATGTTTATAAAGTGACGGCTCGCGCCTATGACCGTAATGGCAATAGCTCTAAC

AATGTACAGCTTACTATTACCGTTCTGTCGAATGGTCAGGTGGTCGACCAGGTTGGGGTA
ACGGACTTTACGGCTGATAAGACTTCGGCTAAAGCGGATGGCACCGAAGCAATTACTTAT
ACTGCGACGGTGAAAAAGAATGGGGTAGCTCAGGCTAATGTCCCTGTTTCATTTAATATT
GTTTCAGGAACTGCAGTTTTAAGTGCCAATAGTGCCAATACTAATAGTAGCGGTAAGGCG
ACTGTAACCCTGACATCAAATAAACCGGGCCAGGTCGTCGTATCAGCTAAAACAGCAGAG
ATGACTTCAGCGCTTAATGCCAATGCTGTTATATTTGTTGATCAAACCAAGGCCAGCATT
ACTGAGATTAAGGCTGATAAAACAACTGCAGTAGCAAATGGTCAGGATGCTATTACATAC
ACTGTTAAAGTGATGAAAAATGGTCAGCCATTATCCGGTGAAGAAGTGACTTTCTTTACG
GATTTTGGGGCATTGGATAAAACTAAAGTAACGACCGATCAGAGTGGTTATGCTACTGTA
AAACTGTCATCCAGTACTTCAGGCAAAGCTATTGTTCGCGCAAAAGTGAGTGATGTTGAT
ACAGAAGTTAAGGCTGCCGCTGTTGAGTTTTTGCCTCCCTGAGTATTGATAGCAATAAA
GTTACTGTAATTGGTACTGGTGTCACTGGTTCTCTGCCAAAGAACTGGTTGCAGTATGGT
CAGGCTAAGTTACAGGCAACGGGAGGTAATGGGAAATATACATGGAAATCCAGTGATACT
AAAATTGCTTCTGTTGATAGTTCGGGAGTGATAACCCTGAATGGGAAAGGGAGTACCATA
ATTACGGTCGTATCTGGTGATAATCAGAGTGCGACATATACAATTAGTACTCCTGACAAG
ATTGTAATAGCTGTGGATAAAATTAATCGGATGACATATTCTGCTGCCGAAAACAAGTGC
CGAACAATTAGTGCAAATTTAGCACCGTCAAAGAGTATATTGGCAGACACATATTCTAAA
TGGGGAGCAGCGAATAAATATTCTTACTATTCTGGTTCTAATTCATTGACAGCTTGGGTT
ACACAATCCTCTTCTGAGTTACCATCGGGTGTATCAAGTACATATGATTTAATCACAACA
AACTCTTTGACAAATGTTAAGACAACAGATAACAATGCCTTTGCAGTCTGTGTGAAATAA
>eaeA_lambda_DQ523609
ATGATTACTCATGGTTGTTATACCCGGACCCGGCACAAGCATAAGCTAAAAAAAACATTG
ATTATGCTTAGTGCTGGTTTAGGATTGTTTTTTATGTTAATCAGAATTCATTTGCAAAT
GGTGAAAATTATTTTAAATTGGGTTCGGATTCAAAACTGTTAACTCATGATAGCTATCAG
AATCGCCTTTTTTATACGTTGAAAACAGGTGAAACTGTTGCCGATCTTTCTAAATCGCAA
GATATTAATTTATCGACGATTTGGTCGTTGAATAAGCATTTATACAGTTCTGAAAGTGAA
ATGATGAAGGCCGCGCCTGGTCAGCAGATCATTTTGCCACTCAAAAAACTTCCCTTTGAA
TACAGTGCACTACCACTTTTAGGTTCGGCACCTCTTGTTGCTGCAGGTGGTGTTGCTGGT
CACACGAATAAACTGACTAAAATGTCCCCGGACGTGACCAAAAGCAACATGACCGATGAC
AAGGCATTAAATTATGCGGCACAACAGGCGGCGAGTCTCGGTAGCCAGCTTCAGTCGCGA
TCTCTGAACGGCGATTACGCGAAAGATACCGCTCTTGGTATCGCTGGTAACCAGGCTTCG
TCACAGTTGCAGGCCTGGTTACAACATTATGGAACGGCAGAGGTTAATCTGCAGAGTGGT
AATAACTTTGACGGTAGTTCACTGGACTTCTTATTACCGTTCTATGATTCCGAAAAAATG
CTGGCATTTGGTCAGGTCGGAGCGCGTTACATTGACTCCCGCTTTACGGCAAATTTAGGT
GCGGGTCAGCGTTTTTTCCTTCCTGCAAACATGTTGGGCTATAACGTCTTCATTGATCAG
GATTTTTCTGGTGATAATACCCGTTTAGGTATTGGTGGCGAATACTGGCGAGACTATTTC
AAAAGTAGCGTTAACGGCTATTTCCGCATGAGCGGCTGGCATGAGTCATACAATAAGAAA
GACTATGATGAGCGCCCAGCAAATGGCTTCGATATCCGTTTTAATGGCTATCTACCGTCA
TATCCGGCATTAGGCGCCAAGCTGATATATGAGCAGTATTATGGTGATAATGTTGCTTTG
TTTAATTCTGATAAGCTGCAGTCGAATCCTGGTGCGGCGACCGTTGGTGTAAACTATACT
CCGATTCCTCTGGTGACGATGGGGATCGATTACCGTCATGGTACGGGTAATGAAAATGAT
CTCCTTTACTCAATGCAGTTCCGTTATCAGTTTGATAAATCGTGGTCTCAGCAAATTGAA
CCACAGTATGTTAACGAGTTAAGAACATTATCAGGCAGCCGTTACGATCTGGTTCAGCGT
AATAACAATATTATTCTGGAGTACAAGAAGCAGGATATTCTTTCTCTGAATATTCCGCAT
GATATTAATGGTACTGAACACAGTACGCAGAAGATTCAGTTGATCGTTAAGAGCAAATAC
GGTCTGGATCGTATCGTCTGGGATGATAGTGCATTACGCAGTCAGGGCGGTCAGATTCAG
CATAGCGGAAGCCAAAGCGCACAAGACTACCAGGCTATTTTGCCTGCTTATGTGCAAGGT
GGCAGCAATATTTATAAAGTGACGGCTCGCGCCTATGACCGTAATGGCAATAGCTCTAAC
AATGTACAGCTTACTATTACCGTTCTGTCGAATGGTCAAGTTGTCGACCAGGTTGGGGTA
ACGGACTTTACGGCGGATAAGACTTCGGCTAAAGCGGATAACGCCGATACCATTACTTAT
ACCGCGACGGTGAAAAAGAATGGGGTAGCTCAGGCTAATGTCCCTGTTTCATTTAATATT
GTTTCAGGAACTGCAACTCTTGGGGCAAATAGTGCCAAAACGGATGCTAACGGTAAGGCA
ACCGTAACGTTGAAGTCGAGTACGCCAGGACAGGTCGTCGTGTCTGCTAAAACCGCGGA
G
ATGACTTCAGCACTTAATGCCAGTGCGGTTATATTTGTTGATCAAACTAAGGCCAGCATT
ACTGAGATTAAGGCTGATAAGACAACTGCAGTAGCAAATGGTAAGGATGCTATTAAATAT
ACTGTAAAAGTTATGAAAAACGGTCAGCCTGTGAAGGGCTATGATGTAACGTTTTTAACA

ACTGCAGGTAATCTGAGTAAAACAAAAGAGTTAACAGATAAAGATGGATATGCAACTGTA
AACTTGACCTCTAATGCTGCGGGAAAAGCTGTTGTTAGTGCCAAGGTTAGCGATGTTAAT
TCTGAGGTTAAAGCTTCCGAAGTTGAGTTCTTCACAGAACTGAGTATCAATAAAAATGTA
GAGGTTCTTGGTACAAAGGCTAGTGGTGAGTTACCTGATGTCTGGCTACAATATGGTCAG
ATTAAATTAAATGTTAATGGTGGCAATGATAAATACTCATGGAGCTCCAGTAATCCTAAT
ATTGCGTCGATAGATGCCTCTTCCGGTATTATTACCTTAAAGGAAAAGGGGGAGGCTGTT
ATTAAGGTTGTATCTGGGGATAAGCAAACGGCGACATACACTATTTCTACACCAAAGAAA
ATAGTGAGTGTTAATTCGGGTTCTAGGGTTAATTATAATTCGGCCAGTTCTATTTGCGGA
AAAATTAATGGCTCGTTACCATCTTCAATTGCAGAGCTGGAAACACTGTATAATAAATGG
GGAGCAGCCAATAACTATCAACACTATACTCAATTATCTATAACAGCATGGACGTTGCAA
ACTAGTGATGATGTGAAGAAAGGGGTCACCAGTACTTACGATTTGGTAAGGAAAAACCCA
CAATTAAATAAAGTTAATATAAACGACAATAATGCTTATGCTGTCTGTGTGAAATAA
>eaeA_mu_DQ523607
ATGATTACTCATGGTTTTTATGCCCGGACCCGGCACAAGCATAAGCTAAAAAAAACATTT
ATTATGCTTAGTGCTGGTTTAGGATTGTTTTTTTATGTTAATCAGAATTCATTTGCAAAT
GGTGAAAATTATTTTAAATTGGGTTCGGATTCAAAACTGTTAACTCATAATAGCTATCAG
AATCGCCTTTTTTATACGTTGAAAACAGGTGAAACTGTTGCCGATCTTTCTAAATCGCAA
GATATTAATTTATCGACGATTTGGTCGTTGAATAAGCATTTATACAGTTCTGAAAGCGAA
ATGATGAAGGCCGCGCCAGGTCAGCAGATCATTTTGCCACTCAAAAAACTTCCCTTTGAA
TACAGTGCCTTACCACTTTTAGGTTCGGCACCTCTTGTTGCTGCAGGTGGTGTCGCTGGT
CATACAAATAAAATGACTAAAATGTCCCCGGACGTGGCCAAAAGCAACATGACCGATGAC
AAGGCATTAAATTATGCGGCACAACAGGCGGCGAGTCTCGGTAGCCAGCTTCAGTCGCGA
TCTCTGAACGGCGATTACGCGAAAGATACCGCTCTTGGTATCGCTGGTAACCAGGCTTCG
TCACAGTTGCAGGCCTGGTTACAACATTATGGAACGGCAGAGGTTAATCTGCAGAGTGGT
AATAACTTTGACGGTAGTTCACTGGACTTCTTATTACCGTTCTATGATTCCGAAAAAATG
CTGGCATTTGGTCAGGTCGGAGCGCGTTACATTGACTCCCGCTTTACGGCAAATTTAGGT
GCGGGTCAGCGTTTTTTCCTTCCTGAAAATATGTTGGGCTATAACGTCTTCATTGATCAG
GATTTTTCTGGTGATAATACCCGTTTAGGTATTGGTGGCGAATACTGGCGAGACTATTTC
AAAAGTAGTGTTAACGGCTATTTCCGCATGAGCGGCTGGCATGAGTCATACAATAAGAAA
GACTATGATGAGCGCCCAGCAAATGGCTTCGATATCCGTTTTAATGGCTATCTGCCATCA
TACCCGGCATTAGGTGCCAAGCTGATGTATGAGCAGTATTATGGTGATAATGTTGCTTTG
TTTAATTCTGATAAGCTGCAGTCGAATCCTGGTGCGGCGACCGTTGGTGTAAACTATACT
CCGATTCCTCTGGTGACGATGGGGATCGATTACCGTCATGGTACGGGTAATGAAAATGAT
CTCCTTTACTCAATGCAGTTCCGTTATCAGTTTGATAAACCGTGGTCTCAGCAAATTGAG
CCACAATATGTTAACGAGTTAAGAACATTATCAGGCAGCCGTTACGATCTGGTTCAGCGT
AATAACAATATTATTCTGGAGTACAAAAAGCAGGATATTCTTTCTCTGAATATTCCGCAT
GATATTAATGGTACTGAACGCAGTACGCAGAAGATTCAATTGATCGTTAAGAGCAAATAC
GGTCTGGATCGTATCGTCTGGGATGATAGTTCATTACGTAGCCAGGGCGGCCAGATTCAG
CATAGCGGAAGCCAAAGCGCACAAGATTACCAGGCTATTTTGCCTGCTTATGTGCAAGGT
GGTAGCAATGTTTATAAAGTGACGGCTCGCGCCTATGACCGTAATGGCAATAGCTCTAAC
AATGTACAGCTTACTATTACCGTTCTGTCGAATGGTCAGGTGGTCGACCAGGTTGGGGTA
ACGGACTTTACGGCTGATAAGACTTCGGCTAAAGCGGATGGCACCGAAGCAATTACTTAT
ACTGCGACGGTGAAAAAGAATGGGGTAGCTCAGGCTAATGTCCCTGTGTCATTTGATATT
GTTTCAGGTGATGCAACTTTAAGTGCCAAGAGTGCCAATACTAATAGTAGCGGTAAGGCG
ACTGTAACCCTGAAATCGGATAAACCAGGCCAGGTCGTCGTGTCTGCTAAAACAGCAGAG
ATGACTTCAGCGCTTAATGCCAATGCAGTTATATTTGTTGATCAAACCAAGGCCAGCATT
ACTGAGATTAAGGCTGATAAAACAACGGCAGTAGCAAATGGTCAGGATGCTATTACATAC
ACTGTTAAAGTGATGAAAGATGGTCAGCCAGTGCAGGGACACTCCGTTACATTCTCAACA
AACTTTGGGATATTCAACGGTAAGTCTCAGACACAAAATGCAACCACGGGAAATGATGGT
CGTGCGACGATAACGCTGACTTCCAGTTCCGCAGGTAAAGCGACTGTTAGTGCGACAGTT
AGTGGTGGAACTGATGTTAAAGCGACTGAGGTTACTTTTTTTGATGAACTGAAAATTGAC
AACAAGGTTGACATTATTGGTAACAATGTCAGTGGCGAATTGCCTGATATCTGGTTGCAA
TATGGTCAGTTTAAATTGAAGGCAAGTGGTGGTAACGGTACATATTCATGGTATTCAGAA
AATACCAGTATCGCGACTGTTGATGCATCGGGGAAAGTCACCTTGAATGGTAAAGGCAGT
GTCGTAATTAAAGCCACTTCTGGTGATAAGCAAACAGTAAGCTACACTGTTAAAACTCCA
AAATATATGATAAAGGTAGGCCAAAAAGCTTATTATGATGAGTCCATGACTATTTGTAAA
GGCTCTCTGCCATCCTCACAGACTGTATTATCTGACATCTTTAACGCATGGGGGGCAGCA

AATAAATATAGCCATTATAACACTATGAGCTCAATAACGGCTTGGATTAAACAAACTGAA
AAAGATAAAGCATCTGGAGTATCAAGTACTTATAACTTAATAACACAAAACCCTCTTCCC
GGGGTTAACGTAAAAACTCCAAATGTCTATGCGGTTTGTGTAGAATAA
>eaeA_nu_DQ523615
ATGATTACTCATGGTTGTTATACCCGGACCCGGCACAAGCATAAGCTAAAAAAAACATTT
GTTATGCTTAGCGCTGGTTTAGGATTGTTTTTTTATGTTAACCAGAACTCATTTGCAAAC
GGTGAAAACTATTTTAAACTGAGTTCAGATTCTAAACTATTAACTCAAAATGCCGCTCAG
AATCGCCTTTTTTATACGTTGAAAACAGGTGAAACTGTTGCCGATCTTTCTAAATCGCAA
AATATTAATTTATCGACGATTTGGTCGCTGAATAAGCATTTATACAGTTCCGAAAGCGAA
ATGATGAAGGCTGCACCTGGTCAGCAGATCATTTTGCCACTCAAAAAACTTCCTGTTGAA
TATAGCGCCTTACCACACTTAGGTTCGGCGCCTGTCGTTGCAGCAGGTGGTGTTGCTGGT
CATACGAATAAAATGGCTAAAATGTCCCCGGACATGACCAAAAGCAACATGACCGATGAC
AAGGCGCTAAATTATGCGGCACAACAGGCTGCGAGTCTCGGTAGCCAACTTCAGTCTCGC
TCACTGAACGGCGATTACGCAAAAGATGCTGCCCTTGGTATGGCCGGCAACCAGGCTTCG
TCACAGTTGCAGGCCTGGTTACAACATTATGGAACGGCAGAGGTTAATCTGCAGAGCGGT
AATAACTTTGATGGCAGTTCGCTGGACTTCTTATTACCGTTCTATGATACCGAAGAGATG
CTGGCATTTGGTCAGGTCGGAGCGCGTTACATTGACTCCCGCTTTACGGCAAATTTAGGT
GCTGGTCAGCGCTTCTTCCTTTCTGAAAATATGCTGGGCTATAACGTCTTCATTGATCAG
GATTTTTCTGGTGATAATACCCGTTTAGGTATTGGTGGCGAATACTGGCGAGACTATTTC
AAAAGTAGCGTGAACGGCTATTTCCGCATGAGCGGTTGGCATGAGTCATACAACAAGAAA
GACTATGACGAGCGCCCGGTAAATGGTTTCGATATCCGCTTTAATGGGTATTTACCATCA
TATCCGGCATTAGGCGCCAAACTGATGTACGAACAGTATTATGGTGATAATGTCGCTTTG
TTTAATGCCGATAAACTGCAATCGAATCCTGGCGCGGCGACCGTTGGTGTAAACTACACT
CCGGTTCCTCTGGTGACGATGGGGGTTGATTACCGTCATGGGACAGGTAATGAAAATGAT
CTCCTTTACTCAATGCAGTTCCGTTATCAATTTGACAAACCGTGGTCTCAGCAAATTGAG
CCGCAGTATGTTAACGAGTTAAGAACATTATCAGGCAGCCGTTACGATCTGGTACAACGT
AATAACAATATTATTCTGGAATACAAAAAGCAGGATATTCTTTCTTTGAGTATCCCACAT
GATATTAATGGTACTGAACACAGTACGCAGAAGATTCAATTGAACGTTAAGAGTAAGTAC
GGCCTGGATCGTATCGTCTGGGATGACAGTGCATTACGCAGTCAGGGCGGTCAGATTCAG
CATAGCGGAAGCCAAAGCGCACAAGATTACCAGGCTATTTTGCCTGCTTATGTGCAAGGT
GGTAGCAATATTTATAAAGTGACCGCTCGCGCCTATGACCGTAATGGCAATAGCTCTAAC
AATGTACAGCTCACTATTACCGTTCTGTCGAATGGTCAGGTTGTCGGCCAGGTTGGGGTA
ACGGACTTTACGGCTGATAAGACATCGGCTAAAGCGGATGGCACCGAAGTTATTACTTAT
ACCGCGACGGTTAAAAAGAATGGGGTTGCCCAGGCTAATGTTCCTGTTTCATTTGATATT
GTTTCAGGTGATGCAACTTTAAGTGCCAGGAGTGTCACTACTAATAGCAGCGGTAAGGCG
ACCGTAACGCTGAAATCAAGTAAGCCGGGCCAGGTAGTCGTGTCAGCTAAAACAGCGGA
G
ATGACTTCAGCACTTAATGCCAGTGCGGTTATATTTGTTGATCAAACTAAGGCCAGTATT
ACTGAGATTAAGGCTGATAAGACAACTGCAGTAGCAAATGGTAAGGATGCTATTACATAT
ACTGTTAAAGTGATGAAAAATGGTCAGCCGTTATCCGGTGAAGAAGTGACCTTTACGAAG
ACCTTAGGTACGTTAAGTAAGTCCATTGAAAAAACGGATGCGAATGGCTATGCTAAAGTA
ACATTAACATCTGTGACTCAAGGGAAATCCCTCGTTAGTGCCCGTGTTAGCGATGTTGCC
GTTGACGTCAAAGCACCTGAAGTTGAATTTTTTACGCAGCTTACAATTGATAACAGTAAT
ATCGAAATTGTTGGTACAGGAGTTAAAGGGACGCTGCCTACTGTATGGTTGCAATATGGT
CAAGTTAAGTTGAAAGCTAGCGGAGGTAACGGAAGATATACATGGCGTTCAGCAAATACA
GCAATTGCTTCGGTGGATGCTTCTTCAGGTCAAATCACCTTAAAAGATAAGGGGACTACA
ACTATTACCGTTGTATCAGGTGATAATCAAACCGCAACTTATACTATTGCAACACCTAGC
AGTATGATTGTCTCTAATATAAACAGTCGTATGACTTATAGTGAGGCAATGAGTTCATGC
CAGAGCTTGAGAGGAAGGTTACCATCTTCTCAAAGTGAACTGGCTGATGTATTCGATACA
TGGGGGGCGGCAAATAAATATGAATATTATAAGACTAGCACAAGCATAATATCGTGGATA
AAACAAACCGATGATGATGTGAAGAAGGGCGTTGCAAGCACATACGATCTGGTTAAGAGA
AATCCTCTTACTAGTTCAGTAAAAACAGACGCTCGTAATGCTTATGCAACTTGCGTAAAA
TAA
>eaeA_omicron_AY696838
ATGATTACTCATGGTTGTTATACCCGGACCCGGCACAAGCATAAGCTAAAAAAAACATTT
GTTATGCTTAGCGCTGGTTTAGGATTGTTTTTTTATGTTAACCAGAACTCATTTGCAAAT
GGTGAAAACTATTTTAAACTGAGTTCAGATTCTAAACTATTAACTCAAAATGCCGCTCAG

AATCGCCTTTTTTATACGTTGAAAACAGGTGAAACTGTTGCCGATCTTTCTAAATCGCAA
AATATTAATTTATCGACGATTTGGTCGCTGAATAAACATTTATACAGTTCCGAAAGCGAA
ATGATGAAGGCTGCACCTGGTCAGCAGATCATTTTGCCACTCAAAAAACTTCCTGTTGAA
TATAGCGCCTTACCACACTTAGGTTCGGCGCCTGTCGTTGCAGCAGGTGGTGTTGCCGGT
CATACGAATAAAATGGCTAAAATGTCCCCGGACATGACCAAAAGCAACATGACCGATGAC
AAGGCACTAAATTATGCGGCACAACAGGCTGCGAGTCTCGGTAGCCAACTTCAGTCTCGC
TCACTGAACGGCGATTACGCAAAAGATGCTGCCCTTGGTATGGCCGGCAACCAGGCCTCG
TCACAGTTGCAGGCCTGGTTACAACATTATGGAACGGCAGAGGTTAATCTGCAGAGCGGT
AATAACTTTGATGGCAGTTCGCTGGACTTCTTATTACCGTTCTATGATACCGAAGAGATG
CTGGCATTTGGTCAGGTCGGAGCGCGTTACATTGACTCCCGCTTTACGGCAAATTTAGGT
GCTGGTCAGCGCTTCTTCCTTCCTGAAAATATGCTGGGCTATAACGTCTTCATTGATCAG
GATTTTTCTGGTGATAATACCCGCTTAGGTATTGGTGGGAATACTGGCGAGACTATTTC
AAAAGTAGTGTGAACGGCTATTTCCGCATGAGCGGTTGGCATGAGTCATACAACAAGAAA
GACTATGACGAGCGCCCGGTAAATGGTTTCGATATCCGCTTTAATGGGTATTTACCATCA
TATCCGGCATTAGGCGCCAAACTGATGTACGAACAGTATTATGGTGATAATGTCGCTTTG
TTTAATGCCGATAAACTGCAGTCGAATCCTGGCGCGGCGACCGTTGGTGTAAACTACACT
CCGGTTCCTCTGGTGACGATGGGGGTTGATTACCGTCATGGGACAGGTAATGAAAATGAT
CTCCTTTACTCAATGCAGTTCCGTTATCAATTTGACAAACCGTGGTCTCAGCAAATTGAG
CCGCAGTATGTTAACGAGTTAAGAACGTTATCAGGCAGCCGTTACGATCTGGTACAACGT
AATAACAATATTATTCTGGAATACAAAAAGCAGGATATTCTTTCTTTGAGTATTCCACAT
GATATTAATGGTACTGAACACAGTACGCAGAAGATTCAATTGAGCGTTAAGAGTAAGTAC
GGCCTGGATCGTATCGTCTGGGATGACAGTGCATTACGCAGTCAGGGCGGTCAGATTCAG
CATAGCGGAAGCCAAAGCGCACAAGATTACCAGGCTATTTTGCCTGCTTATGTGCAAGGT
GGTAGCAATATTTATAAAGTGACCGCTCGCGCCTATGACCGTAATGGCAATAGCTCTAAC
AATGTACAGCTCACTATTACCGTTCTGTCGAATGGTCAGGTTGTCGACCAGGTTGGGGTA
ACGGACTTTACGGCTGATAAAACATCGGCTAAAGCGGATGGCATCGAGGCTATTACTTAT
ACCGCGATTGTTAAAAAAAATGGGGTTATTCAGGCTAATGTTCCTGTTTCATTCGATATT
GTTTCAGGTGATGCAACTTTAAGTGCCAGGAGTGCCACTACTAATAGCAGCGGTAAGGCG
ACCGTAACGCTGAAATCAAATAAGCCGGGCCAGGTAGTCGTGTCAGCTAAAACTGCGGAG
ATGACTTCAGCACTTAATGCCAATGCGGTTATATTTGTTGATCAAACTAAGGCTAGCATT
ACTGAAATTAAAGCTGATAAGACAACTGCAGTAGCAAATGGTAAGGATGCTGTCACATAC
ACTGTTAAAGTAATGAAAGATGGTAAGCCGTTATCCGGTGAAGAAGTGACCTTTACGACG
ACCTTAGGGACGTTAAGTAAGTCCACTGAAAAAACGAATACGAATGGCTATGCTAAAGTA
ACGTTAACATCGACGAATCAAGGAAAATCACTCGTTAGTGCCAGCGTTAGCAATGCCGCA
GTTGATGTTAAAGCTCCTGAAGTTGAATTTTTTACACAGCTTACAATTGATAACGGTAAT
GTTGAAATTGTTGGAACCGGAGCTAAGGGGAAACTACCTAATGTATGGTTGCAATATGGC
CAAGTTAATCTGAAAGCTAACGGAGGTAACGGGAAATATACATGGTACTCAGCAAATCCG
GCAATTGCTTCGGTGGATCCCTCTTCTGGTCAAGTCACATTAAAAGATAAGGGGGAAACA
ACAATTACCGTTGTATCTGGGGATAAACAAACGGCCATCTATACAATTGCTATGCCTAAT
AGCATAGTGAGCGTTAACTCAAGTGGTAGGGTTGATTATAATACAGCCAATAACATTTGC
AAAAATATTAAAGGTTCGTTACCTTCTTCAATTAAAGAGCTGAAGGATTTATATGATGAT
TGGGGGGCTGCAAATAAATATCAACATTATTCCCAGGAATCTATAACTGCCTGGACATTA
CAAACTAGTGAGAATAAAGTGCAAGGGGTTGCAAGTACTTACGATTTGGTAAGGAAAAAT
CCACTGATAGATAAAGTTGATATAGCAGGTAATTATGCCTATGCAGTCTGTGTGAGATAA
>eaeA_pi_AJ705052
ATGATTACTCATGGTTTTTATGCCCGGACCCGGCACAAGCATAAGCTAAAAAAAACATTT
ATTATGCTTAGTGCTGGTTTAGGATTGTTTTTTTATGTTAATCAGAATTCATTTGCAAAT
GGTGAAAATTATTTTAAATTGGGTTCGGATTCAAAACTGTTAACTCATAATAGCTATCAG
AATCGCCTTTTTTATACGTTGAAAACAGGTGAAACTGTTTCCGATCTTTCTAAATCGCAA
GATATTAATTTATCGACGATTTGGTCGTTGAATAAGCATTTATACAGTTCTGAAAGCGAA
ATGATGAAGGCCGCGCCAGGTCAGCAGATCATTTTGCCACTCAAAAAACTTCCCTTTGAA
TACAGTGCCTTACCACTTTTAGGTTCGGCACCTCTTGTTGCTGCAGGTGGTGTCGCTGGT
CATACAAATAAAATGACTAAAATGTCCCCGGACGTGGCCAAAAGCAACATGACCGATGAC
AAGGCATTAAATTATGCGGCACAACAGGCGGCGAGTCTCGGTAGCCAGCTTCAGTCGCGA
TCTCTGAACGGCGATTACGCGAAAGATACCGCTCTTGGTATCGCTGGTAACCAGGCTTCG
TCACAGTTGCAGGCCTGGTTACAACATTATGGAACGGCAGAGGTTAATCTGCAGAGTGGT
AATAACTTTGACGGTAGTTCACTGGACTTCTTATTACCGTTCTATGATTCCGAAAAAATG

CTGGCATTTGGTCAGGTCGGAGCGCGTTACATTGACTCCCGCTTTACGGCAAATTTAGGT
GCGGGTCAGCGTTTTTTCCTTCCTGAAAATATGTTGGGCTATAACGTCTTCATTGATCAG
GATTTTTCTGGTGATAATACCCGTTTAGGTATTGGTGGCGAATACTGGCGAGACTATTTC
AAAAGTAGTGTTAACGGCTATTTCCGCATGAGCGGCTGGCATGAGTCATACAATAAGAAA
GACTATGATGAGCGCCCAGCAAATGGCTTCGATATCCGTTTTAATGGCTATCTGCCATCA
TACCCGGCATTAGGTGCCAAGCTGATGTATGAGCAGTATTATGGTGATAATGTTGCTTTG
TTTAATTCTGATAAGCTGCAGTCGAATCCTGGTGCGGCGACCGTTGGTGTAAACTATACT
CCGATTCCTCTGGTGACGATGGGGATCGATTACCGTCATGGTACGGGTAATGAAAATGAT
CTCCTTTACTCAATGCAGTTCCGTTATCAGTTTGATAAACCGTGGTCTCAGCAAATTGAG
CCACAGTATGTTAACGAGTTAAGAACATTATCAGGCAGCCGTTACGATCTGGTTCAGCGT
AATAACAATATTATTCTGGAGTACAAAAAGCAGGATATTCTTTCTCTGAATATTCCGCAT
GATATTAATGGTACTGAGCGCAGTACGCAGAAGATTCAATTGATCGTTAAGAGCAAATAC
GGTCTGGATCGTATCGTCTGGGATGATAGTTCATTACGTAGCCAGGGCGGCCAGATTCAG
CATAGCGGAAGCCAAAGCGCACAAGATTACCAGGCTATTTTGCCTGCTTATGTGCAAGGT
GGTAGCAATGTTTATAAAGTGACGGCTCGCGCCTATGACCGTAATGGCAATAGCTCTAAC
AATGTACAGCTTACTATTACCGTTCTGTCGAATGGTCAAGTTGTCGACCAGGTTGGGGTA
ACGGACTTTACGGCGGATAAGACTTCGGCTAAAGCGGATAACGCCGATACCATTACTTAT
ACCGCGACGGTGAAAAAGAATGGTGTAGCTCAGGCTAATGCCCCTGTAACATTTAGTATT
GTATCCGGGACTGCAACTCTTGGGGCAAATAGTGCCAAAACGGATAGTAACGGCAAGGCA
ACCGTAACGTTGAAGTCGAGTACGCCAGGACAGGTCGTCGTGTCTGCTAAAACCGCAGA
G
ATGACTTCAGCACTTAATGCCAGTGCGGTTATATTTGTTGAGCAAACCAAGGCCAGTATT
ACTGAGATTAAGGCTGATAAAACAACAGCGAAGGCAAATGGCTCTGATGCGATTACCTAT
ACTGTTAAAGTAATGAAGAATAACCAACCCGAAGCAAACCATTCTGTTACATTCTCAACG
AACTTTGGTAAGTTGGATGGTAATTCTAATACCCAAACTGTGAAAACAGATGAAAATGGT
AAAGCTACGGTAAAACTGACATCTGGCGCTGAGGGGAGTGCTGTCGTTAGTGCAAAAGTC
AGCGAGATTAATACAGAAGTTAAGGCTCCTGAGGTAAAATTCTTCTCAGTTCTGAGTATT
GATAATAATGTGAATATTATTGGAACCTCCGCTAATGCTGCTTTACCTAATATTTGGTTG
CAATATGGTCAGTTTAAGCTGACAGCCAAAGGCGGTGATGGAAAATATAAATGGCATTCT
AAAGATACAAGTGTTGCATCAGTTGATGCTTCTACTGGTCAGGTTACTTTGCTGAAGAAA
GGAACGACAACAATTGAAGTAGTGTCTGGCGATAACCAAACCGCGGCATATACAATTAAT
ACACCTGATAAGATAATATCTGTAGAATCACAAAATACAGAAATCTATAGCTCTGCTGAA
AAAACGTGTAGTATTAATAGTGGTCGTTTACCGTTATCTACGAATGAGTTAAAGGATGTG
TATAGTAAATGGGGAGCAGCCAATAGTTATGAGAGGTATAAAGATAAAAATACAATAGCA
GCATGGACACAGCAAACTGAGGAAGATAAAAAAGCAGGTTGGACTAGTACATTTGACATA
GTTACGCAAAATGAAATTCCTAGTAATGGAAGTAATAGCAAGGTCGACGTGAAAAAAGCT
AACGGTTTTGCCGTCTGTGTAAGATGA
>eaeA_rho_DQ523613
ATGATTACTCATGGTTGTTATACCCGGACCCGGCACAAGCATAAGCTAAAAAAAACATTT
GTTATGCTTAGCGCTGGTTTAGGATTGTTTTTTATGTTAACCAGAACTCATTTGCAAAC
GGTGAAAACTATTTTAAACTGAGTTCGGATTCTAAACTATTAACTCAAAATACCGCTCAG
AATCGCCTTTTTTATACGTTGAAAACAGGTGAAACTGTTGCCGATCTTTCTAAATCGCAA
AATATTAATTTATCGACGATTTGGTCGCTGAATAAGCATTTATACAGTTCCGAAAGCGAA
ATGATGAAGGCTGCACCTGGTCAGCAGATCATTTTGCCACTCAAAAAACTTCCTGTTGAA
TATAGCACCTTACCACACTTAGGTTCGGCGCCTGTCGTTGCAGCAGGTGGTGTTGCTGGT
CATACGAATAAAATGGCTAAAATGTCCCCGGACATGACCAAAAGCAACATGACCGATGAC
AAGGCACTAAATTATGCGGCACAACAGGCTGCGAGTCTCGGTAGCCAACTTCAGTCTCGC
TCACTGAACGGCGATTACGCAAAAGATGCTGCCCTTGGTATGGCCGGCAACCAGGCCTCG
TCACAGTTGCAGGCCTGGTTACAACATTATGGAACGGCAGAGGTTAATCTGCAGAGCGGT
AATAACTTTGATGGCAGTTCGCTGGACTTCTTATTGCCGTTCTATGATACCGAAGAGATG
CTGGCATTTGGTCAGGTCGGAGCGCGTTACATTGACTCCCGCTTTACGGCAAATTTAGGT
GCTGGTCAGCGCTTCTTCCTTTCTGAAAATATGCTGGGCTATAACGTCTTCATTGATCAG
GATTTTTCTGGTGATAATACCCGTTTAGGTATTGGTGGCGAATACTGGCGAGACTATTTC
AAAAGTAGCGTGAACGGCTATTTCCGCATGAGCGGTTGGCATGAGTCATACAACAAGAAA
GACTATGACGAGCGCCCGGTAAATGGTTTCGATATCCGCTTTAATGGGTATTTACCATCA
TATCCGGCATTAGGCGCCAAACTGATGTACGAACAGTATTATGGTGATAATGTCGCTTTG
TTTAATGCCGATAAACTGCAATCGAATCCTGGCGCGGCGACCGTTGGTGTAAACTACACT

CCGGTTCCTCTGGTGACGATGGGGGTTGATTACCGTCATGGGACAGGTAATGAAAATGAT
CTCCTTTACTCAATGCAGTTCCGTTATCAATTTGACAAACCGTGGTCTCAGCAAATTGAG
CCGCAGTATGTTAACGAGTTAAGAACATTATCAGGCAGCCGTTACGATCTGGTACAACGT
AATAACAATATTATTCTGGAATACAAAAAGCAGGATATTCTTTCTTTGAGTATTCCACAT
GATATTAATGGTACTGAACACAGTACGCAGAAGATTCAATTGAGCGTTAAGAGTAAGTAC
GGTCTGGATCGTATCGTCTGGGATGACAGTGCATTACGCAGTCAGGGCGGTCAGATTCAG
CATAGCGGAAGCCAAAGCGCACAAGATTACCAGGCTATTTTGCCTGCTTATGTGCAAGGT
GGTAGCAATATTTATAAAGTGACCGCTCGCGCCTATGACCGTAATGGCAATAGCTCTAAC
AATGTACAGCTCACTATTACCGTTCTGTCGAATGGTCAGGTTGTCGACCAGGTTGGGGTA
ACGGACTTTACGGCTGATAAGACATCGGCTAAAGCGGATAACGCTGATACCATTACTTAT
ACTGCGACTGTGAAAAAGAATGGAGTAGCTCAGGCTAATGCCCCTGTAACATTTAGTATT
GTATCCGGGACTGCGACTCTTGGGGCAAATAGTGCCAAAACTGATGGTAACGGTAAGGCA
ACTGTAACGTTGAAGTCGGGTACGCCAGGGCAGGTCGTCGTGTCTGCTAAAACCGCGGAG
ATGACTTCAGCTATTAATGCTGGCTCGGTTATATTCATCGATCAAACCAAGGCCAGTATT
ACTGAAATTACCAATGATAAGTCAACGGCAATAGCAAATGATAAGGACGCCATTACATAT
ACTGTTAAGGTAATGAAAAATGATCAGCCAGTACCGAATCATTTAGTCACATTCACGACG
ACTTTTGGTAAGTTCAATGGTAAACAGAGCTCAGAAACCGTAACAACAGGGAACGACGG
A
CGCGCTATAGTGACACTGACATCAGGTATAGCCGGAAAAGCAATTGTCAGTGCAAAAGTT
AATGAAGTAAATACCGAAGTACAAGCAAAGACTGTTGAGTTTTTCTCAAAACTAGCAGTC
GATAGCAATATAGATATTATTGGCACATCAGTGACAGGAGCTTTGCCTGATGTCTGGTTG
CAATATGGCCAGTTTAAATTGACTGCTAAAGGTGGAAATGGGAAATATACATGGTATTCT
GAAAATCCAAATATTGCATCCGTGGAGTCAACTACAGGACTAGTAACCTTAAATAATAAA
GGGAGTGTTAAAATATACGTTTTATCAGATGATAAGCAAACAGCAAGTTATACAATATCT
ACACCCAAAGATATGATAACAGCTGATATAACAAAACCAGCTATCTATAGTATTGCATTC
AAGCAGTGCCAAACAAAAAATGGAAGACTACCATCTTCACAGCGTGAATTAGAAAACGTC
TTTAATCTCTGGGGGGCTGCAAATAACTATAGATATTATTCTGCCAAACAATCTATAACA
GCATGGATTCAACAATCTGGTTCAGATTCTAGCGGTGGAGTGACTACAACATATGATCTG
ATAAAGAAGAATCCCCGACAGAATGTTGATGTGAAAAGTCAAAATGTCTATTCGGTTTGC
GTTGAATAA
>eaeA_sigma_AJ781125
ATGATTACTCATGGTTTTTATGCCCGGACCCGGCACAAGCATAAGCTAAAAAAAACATTT
ATTATGCTTAGTGCTGGTTTAGGATTGTTTTTTATGTTAACCAGAATTCATTTGCAAAT
GGTGAAAATTATTTTAAATTGAGTTCAGATTCAAAACTGTTAACTCAAAATGCCGCTCAG
GATCGCCTTTTTTATACGTTAAAAACAGGTGAAACTGTTGCCAATATTTCTAAATCACAG
GGTATCAGTTTATCGGTAATTTGGTCACTGAATAAACATTTATACAGTTCCGAAAGCGAA
ATGATGAAGGCTGGACCTGGTCAGCAGATCATTTTGCCACTCAAAAAACTGTCTGTTGAA
TATAGTGCCTTACCTGTCTTAGGTTCGGCACCTGTTGTTGCTGCAGGTGGTGTCGCTGGT
CATACGAATAAAATGACTAAAATGTCCCCGGACGCGACTAAAAGCAACACGACCGATGAC
AAGGCTTTAAATTATGCGGCACAACAGGCCGCGAGCCTTGGTAGCCAGCTCCAGTCGCGC
TCACTGAACGGCGATTACGCGAAAGATACCGCTCTTGGTATGGCTAGCAGCCAGGCTTCG
TCACAGTTGCAGGCCTGGTTACAACATTATGGAACGGCAGAGGTTAATCTGCAGAGTGGT
AATAACTTTGACGGTAGTTCACTGGACTTCTTATTACCGTTCTATGATTCCGAAAACATG
CTGGCATTTGGTCAGGTCGGGGCGCGTTACATTGACTCCCGCTTTACGGCAAATTTAGGT
GCTGGCCAGCGTTTTTTCCTTCCTGAAAATATGTTGGGCTATAACGTCTTCATTGATCAG
GATTTTTCTGGTGATAATACCCGTTTAGGTATTGGTGGCGAATACTGGCGAGACTATTTC
AAAAGTAGCGTTAACGGCTATTTCCGCATGAGCGGCTGGCATGAGTCATACAATAAGAAA
GACTATGATGAGCGCCCGGCAAATGGTTTTGATATCCGCTTTAATGGCTATTTACCATCA
TATCCGGCATTAGGCGCCAAACTGATGTACGAACAGTATTATGGTGATAATGTTGCTTTG
TTTAATTCCGATAAGTTGCAGTCGAATCCTGGCGCGGCGACCGTTGGTGTAAACTACACT
CCGATTCCTCTGGTGACGATGGGGATCGATTACCGTCATGGTACGGGTAATGAAAATGAT
CTCCTTTACTCAATGCAGTTCCGTTATCAGTTTGATAAACCGTGGTCTCAGCAAATCGAG
CCACAGTATGTTAACGAGTTAAGAACATTATCGGGCAGCCGTTACGATCTGGTTCAGCGT
AATAACAATATTATTCTGGAGTACAAAAAGCAGGATATTCTTTCTCTGAATATTCCGCAT
GATATTAATGGTACTGAACACAGTACGCAGAAGATTCAATTGATCGTTAAGAGCAAATAT
GGTCTGGATCGTATCGTCTGGGATGATAGTGCATTACGCAGTCAGGGCGGTCAGATTCAG
CATAGCGGAAGCCAAAGCGCACAAGACTACCAGGCTATTTTGCCTGCTTATGTGCAAGGT

GGCAGCAATATTTATAAAGTGACCGCTCGCGCCTATGACCGAAATGGTAATAGTTCTAAT
AATGTACAGCTCACTATTACCGTTTTACCGAATGGGCAGGTTGTGGACCAGGTTGGGGTA
ACGGACTTTACGGCTGATAAGACATCGGCTAAAGCGGATAACGTTGATACCATTACTTAT
ACCGCGACGGTTAAAAAGAATGGGGTAGCTCAGGCTAATGCTCCTGTAACATTTAGTATT
GTATCCGGGACTGCAACTCTTGGGGCAAATAGTGCTAAAACGGATAGTAACGGTAAGGCA
ACCGTAACGTTGAAGTCGGCTACGCCAGGGCAGGTCGTCGTGTCTGCTAAAACCGCGGAG
ATGACCTCAGCACTTAATGCCAGTGCGGTTATATTTGTTGATCAAACCAAGGCCAGCATT
ACTGAGATTAAGGCTGATAAGACAACTGCAGTAGCAAATGGTCAGGATGCTGTCACATAC
ACTGTTAAAGTGATGAAAGATGGTAAGCCAGTGCAGGGACACTCTGTTGCATTCTCAACA
AACTTTGGGATGTTCAACGGTAAGGCTCAAGCACAAACTGCAATCACGGGAAGTGATGGT
CGTGCGACGATTACACTAACTTCCAATTCCGCCGGTAAAGCGACTGTTAGTGCAGCAGTT
AGTGGGGGAACTGAGGTTAAAGCGACTGAGGTTACTTTTTTTGATGAACTGAAAATTGAC
AATAAGGTTGATATTATTGGTAACAATGTCAGTGGCGAGTTGCCTAATATCTGGTTGCAA
TATGGTCAGTTCAAACTGAAGGCAAGTGGAGGTAACGGTATATATTCATGGTATTCAGAA
AATACCAGTATCGCGACTGTTGATGCATCGGGGAAAGTCACCTTGAATGGTAAAGGCAGT
GTCGTAATTAAAGCCACATCTGGTGATAAGCAAACAGTAAGTTACACTATTAAAGCACCG
TCTTATATGATAAGAGTGGATAATAAAGCCAATTATGCTAGCGCTACGGCCATTTGTAAA
AATTCTTTACCATCCTCACAGAAGGTATTAGCAGACATATTTAATTCATGGGGAGCGGCA
AACAAATATGGTCATTATGGTTCTATGAACTCAATACCGGCCTGGATTAAACAAACCGAA
AGTGATAAAAATTCTGGAGTATCAACCACTTATGATTTAATAAGACAAAATCCTCGTTCT
GATGTTAATGTAGACACTCTAAATGTCTATGCCGTTTGTGTAGAATAA
>eaeA_tau_AY696839
ATGATTACTCATGGTTGTTATACCCGGACCCGGCACAAGCATAAGCTAAAAAAAACATTT
GTTATGCTTAGCGCTGGTTTAGGATTGTTTTTTATGTTAACCAGAACTCATTCGCAAAC
GGTGAAAACTATTTTAAACTGAGTTCAGATTCTAAACTATTAACTCAAAATGCCGCTCAG
AATCGCCTTTTTTATACTTTGAAAACAGGTGAAACTGTTGCCGATCTTTCTAAATCGCAA
AATATTAATTTATCGACGATTTGGTCACTGAATAAACATTTATACAGTTCCGAAAGCGAA
ATGATGAAGGCTGCGCCTGGTCAGCAGATCATTTTGCCACTCAAAAAACTTCCTGTTGAA
TATAGCGCCTTACCACACTTAGGTTCGGCGCCTGTCGTTGCAGCAGGTGGTGTTGCTGGT
CATACGAATAAAATGGCTAAAATGTCCCCGGACATGACCAAAAGCAACATGACCGATGAC
AAGGCACTAAATTATGCGGCACAACAGGCTGCGAGTCTCGGTAGCCAACTTCAGTCTCGC
TCACTGAACGGCGATTACGCAAAAGATGCTGCCCTTGGTATGGCCGGCAACCAGGCCTCG
TCACAGTTGCAGGCCTGGTTACAACATTATGGAACGGCAGAGGTTAATCTGCAGAGCGGT
AATAACTTTGATGGCAGTTCGCTGGACTTCTTATTACCGTTCTATGATACCGAAGAGATG
CTGGCATTTGGTCAGGTCGGAGCACGTTACATTGACTCCCGCTTTACGGCAAATTTAGGT
GCTGGTCAGCGCTTCTTCCTTTCTGAAAATATGCTGGGCTATAACATCTTCATTGATCAG
GATTTTTCTGGTGATAATACCCGTTTAGGTATTGGTGGGGAATACTGGCGAGACTATTTC
AAAAGTAGCGTGAACGGCTATTTCCGCATGAGCGGTTGGCATGAGTCATACAACAAGAAA
GACTATGACGAGCGCCCGGTAAATGGTTTCGATATTCGCTTTAATGGGTATTTACCATCA
TATCCGGCATTAGGCGCCAAACTGATGTACGAACAGTATTATGGTGATAATGTCGCTTTG
TTTAATGCCGATAAACTGCAGTCGAATCCTGGCGCGGCGACCGTTGGTGTAAACTACACT
CCGGTTCCTCTGGTGACGATGGGGGTTGATTACCGTCATGGGACAGGTAATGAAAATGAT
CTCCTTTACTCAATGCAGTTCCGTTATCAATTTGACAAACCGTGGTCTCAGCAAATTGAG
CCGCAGTATGTTAACGAGTTAAGAACATTATCAGGCAGCCGTTACGATCTGGTACAACGT
AATAACAATATTATTCTGGAATACAAAAAGCAGGATATTCTTTCTTTGAGTATTCCACAT
GATATTAATGGTACTGAACACAGTACGCAGAAGATTCAATTGAGCGTTAAGAGTAAGTAC
GGTCTGGATCGTATCGTCTGGGATGACAGTGCATTACGCAGTCAGGGCGGTCAGATTCAG
CATAGCGGAAGCCAAAGCGCACAAGATTACCAGGCTATTTTGCCTGCTTATGTGCAAGGT
GGTAGCAATATTTATAAAGTAACCGCTCGCGCCTATGACCGTAATGGCAATAGCTCTAAC
AATGTGCAGCTCACTATTACCGTTCTGTCGAATGGTCAGGTTGTCGGCCAGGTTGGGGTA
ACGGACTTTACGGCTGATAAGACATCGGCTAAAGCGGATAATGCAGATACCATTACTTAT
ACCGCGACTGTGAAAAAGAATGGGGTTGCTCAGGCTAATATCCCTGTTTCATTTGATATT
GTTTCAGGTGATGCAACTTTAAGTGCCAGGAGTGCCACTACTAATAGCAGCGGTAAGGCG
ACCGTAACGCTGAAATCAAGTAAGCCGGGCCAGGTTGTTGTGTCAGCTAAAACAGCGGA
G
ATGACTTCAGCGCTTAATGCCAATGCGGTTATATTTGTTGATCAAAACAAGGCCAGCATT
ACTGAGATTAAGGCTAATAAGACAACTGCAGTAGCAAATGGTAAGGATGCTGTCACATAC

ACTGTTAAAGTGATGAAAAATGGTCAGCCAGTATCCAATCAGGAAGTGGCTTTTACGACA
ACTTTCGGTAATTTAAGTAATTCCGCTGGAAAAACAGATACGGATGGCTATGCGAAAGTA
ATATTAACATCGACAACTCCAGGAAAATCGCTTGTTAGTGCCCGAGTTCGCGATGTCGCA
GTTGATGTCAAAGCACCTGAAGTTGAATTTTTTACATCACTTGCTATTGATGACAGTAAT
GTTGAAATCGTAGGAACCGGAATCAAAGGGAAGTTACCTACGGTATGGTTGCAATATGGC
CAAGTTAAGCTGAAAGCCAGTGGAGGTGATGGGAAATATACATGGCGTTCGGCAAATCCA
AAAATTGCTTCGGTGGATTCCACTGGTCAAGTTACCTTAAGGGATAAGGGGACTACAACT
ATTACCGTTGTATCAGGTGATAATCAAACCGCAACTTATATTATTGCGACACCTAATAGT
CTGATTGTTCCTAATATAAAAAATCGTATGACTTATAATGATGCTGTAAGCTCATGCCAG
AGTTTGGGAGGAAGGTTACCATCTTCTCAGAGTGAATTGGCGAATGTCTTTAATACATGG
GGGGCAGCAAATAAATATGAACATTATAATGCTAGCACGACTATAATTTCATGGGTAAAA
CAAACAGAAGAAGATATGAAGAAAGGGGTTGCAGACACATACGATCTGGTTAATCAAAAT
CCTCTTAATGGTATTGTTAACACTAAAAAACCTAATGCTTATGCAACTTGCGTAAAATAA
>eaeA_theta_AF449418
ATGATTACTCATGGTTTTTATGCCCGGACCCGGCACAAGCATAAGCTAAAAAAAACATTT
ATTATGCTTAGCGCTGGTTTAGGATTGTTTTTTATGTTAACCAGAACTCATTTGCAAAC
GGTGAAAATTATTTTAAATTGAGTTCAGATTCAAAACTGTTAACTCAAAATGTTGCTCAG
GATCGCCTTTTTTATACGTTGAAAACAGGTGAAACTGTTTCCAGTATTTCTAAATCACAA
GGTATCAGTTTATCCGTAATTTGGTCACTGAATAAACATTTATACAGTTCCGAAAGCGAA
ATGCTGAAGGCTGCGCCTGGCCAGCAGATCATTTTGCCACTCAAAAAACTGTCTGTTGAA
TATGGTGCCTTACCTGTCTTAGGTTCGGCACCTGTTGTTGCTGCAGGTGGTGTCGCTGGG
CATACAAATAAAATGACTAAAATGTCCCCGGACGCGACTCAAAGCAACATGACTGATGAC
AAGGCTCTAAATTATACGGCACAACAGGCCGCGAGCCTTGGTAGCCAGCTTCAGTCGCGC
TCTCTGCACGGCGATTACGCGAAAGATACCGCTCTTGGTATCGCGGGTAACCAGGCTTCG
TCACAGTTGCAGGCCTGGTTACAACATTATGGAACGGCAGAGGTTAATCTGCAGAGTGGT
AATAACTTTGACGGTAGTTCACTGGATTTCTTATTACCGTTCTATGATTCCGAAAAAATG
CTGGCATTTGGTCAGGTCGGAGCGCGTTACATTGACTCCCGCTTTACGGCAAATTTAGGT
GCGGGTCAGCGTTTTTTCCTTCCTGAAAACATGTTGGGCTATAACGTCTTCATTGATCAG
GATTTTTCTGGTGATAATACCCGTTTAGGTATTGGTGGCGAATACTGGCGAGACTATTTC
AAAAGTAGCGTTAACGGCTATTTCCGCATGAGCGGCTGGCATGAGTCATACAATAAGAAA
GACTATGATGAGCGCCCAGCAAATGGCTTCGATATCCGCTTTAATGGCTATCTACCATCA
TACCCGGCATTAGGCGCCAAGCTGATGTATGAGCAGTATTATGGTGATAATGTTGCTTTG
TTTAATTCCGATAAGCTGCAGTCGAATCCTGGTGCGGCGACCGTTGGTGTAAACTATACT
CCGATTCCTCTGGTGACGATGGGGATCGATTACCGTCATGGTACGGGTAATGAAAATGAT
CTTCTTTACTCAATGCAGCTTCGTTATCAGTTTGATAAACCGTGGTCTCAGCAAATTGAG
CCACAGTATGTTAACGAGTTAAGAACATTATCAGGCAGCCGTTACGATCTGGTTCAGCGT
AATAACAATATTATTCTGGAGTACAAGAAGCAGGATATTCTTTCTCTGAATATTCCGCAT
GATATTAATGGTACTGAACACAGTACGCAGAAGATTCAATTGATCGTTAAGAGCAAATAC
GGTCTGGATCGTATCGTCTGGGATGATAGTGCATTACGTAGCCAGGGCGGTCAGATTCAG
CATAGCGGAAGCCAAAGCGCACAAGATTACCAGGCTATTTTGCCGGCTTATGTGCAAGGC
GGTAGCAATATTTATAAAGTGACGGCTCGTGCCTATGACCGTAATGGCAATAGCTCTAAC
AATGTACAGCTCACTATTACCGTTCTGTCGAATGGTCAGGTGGTCGACCAGGTTGGGGTA
ACGGACTTTACGGCTGATAAGACTTCGGCTAAAGCGGATGGCACCGAGGCGATTACTTAT
ACCGCGACGGTGAAAAAGAATGGGGTAACTCAGGCTAATGTCCCTGTTTCATTTAATATT
GTTTCAGGAACTGCAACTCTTGGGGCAAATAGTGCCACAACGGATGCTAACGGTAAGGCA
ACTGTAACGTTGAAGTCGAGTACGCCAGGGCAGGTAGTCGTGTCTGCTAAAACCGCGGAG
ATGACTTCAGCACTTAATGCCAGTGCGGTTATATTTGTTGAGCAAACCAAGGCCAGTATT
ACTGGGATTAAGGCTGATAAGACAACTGCAGTAGCAAATGGTAATGATGCTGTTACATAC
ACTGTTAAAGTGATGAAAGAGGGTCAGCCAGTGCAGGGACACTCCGTTGCATTCACAACA
AACTTTGGGATGTTCAACGGTAAGTCTCAGACGCAAAATGCGACCACGGGAAGTGATGGT
CGTGCGACGATAACACTGACTTCCAGTTCCGCAGGTAAAGCGACTGTTAGTGCGACTGTT
AGTGGTGGGAATGATGTTAAAGCACCTGAGGTTACATTTTTTGATGGACTGAAAATTGAC
AACAAGGTTGATATTCTTGGTAAGAACGTTACTGGTGACTTACCTAATATCTGGTTGCAA
TATGGTCAGTTTAAACTGAAGGTAAGCGGTGGTAATGGTACATATTCATGGCATTCAGAG
AATACCAATATTGCGACTGTTGATGAATCAGGGAAAGTAACCTTGAAAGGAAAAGGTACT
GCAGTAATTAATGTTACATCTGGTGATAAGCAAACAGTAAGCTACACTATTAAAGCTCCG
AATTATATGATAAGAGTGGGTAATAAAGCCAGTTATGCAAATGCTATGTCCTTTTGTGGA

AATTTATTACCATCCTCACAGACGGTATTATCAAACGTTTATAATTCATGGGGGCCTGCA
AACGGATATGACCATTATCGTTCTATGCAGTCAATAACAGCTTGGATTACACAAACTGAA
GCTGATAAAATATCAGGAGTATCAACTACTTATGACTTAATAACACAAAACCCTCATAAG
GATGTTACGCTAAACGCTCCAAATGTCTATGCAGTTTGTGTAGAATAA
>eaeA_xi_DQ523610
ATGATTACTCATGGTTTTTATGCCCGGACCCGGCACAAGCATAAGCTAAAAAAAACATTT
ATTATGCTTAGTGCTGGTTTAGGATTGTTTTTTATGTTAACCAGAATTCATTTGCAAAC
GGCGAAAATTATTTAAATTGAGTTCAGATTCAAAACTGTTAACTCAAAATGCCGCTCAG
GATCGCCTTTTTTATACGTTAAAAACAGGTGAAACTGTTGCCAATATTTCTAAATCACAG
GGTATCAGTTTATCGGTAATTTGGTCACTGAATAAACATTTATACAGTTCCGAAAGCGAA
ATGATGAAGGCTGGACCTGGTCAGCAGATCATTTTGCCACTCAAAAAACTGTCTGTTGAA
TATAGTGCCTTACCTGTCTTAGGTTCGGCACCTGTTGTTGCTGCAGGTGGTGTCACTGGT
CATACGAATAAAATGACTAAAATGTCCCCGGACGTGACTAAAAGCAACACGACCGATGAC
AAGGCTCTAAATTATGCGGCACAACAGGCCGCGAGCCTTGGTAGCCAGCTTCAGTCGCGC
TCACTGAACGGCGATTACGCGAAAGATACCGCTCTTGGTATGGCCAGCAGCCAGGCTTCA
TCACAGTTGCAGGCCTGGTTACAACATTATGGAACGGCAGAGGTTAATCTGCAGAGCGGT
AATAACTTTGACGGTAGTTCACTGGACTTCTTATTACCGTTCTATGATTCCGAAAATATG
CTGGCATTTGGTCAGGTCGGGGCGCGTTACATTGACTCCCGCTTTACGGCAAATTTAGGT
GCTGGCCAGCGTTTTTTCCTTCCTGAAAATATGTTGGGCTATAACGTCTTCATTGATCAG
GATTTTTCTGGTGATAATACCCGTTTAGGTATTGGGGGCGAATACTGGCGAGACTATTTC
AAAAGTAGCGTTAACGGCTATTTCCGCATGAGCGGCTGGCATGAGTCATACAATAAGAAA
GACTATGATGAGCGCCCGGCAAATGGTTTTGATATCCGCTTTAATGGCTATTTACCATCA
TATCCGGCATTAGGCGCCAAACTGATGTATGAACAGTATTATGGTGATAATGTTGCTTTG
TTTAATTCCGATAAGTTGCAGTCGAATCCTGGCGCGGCGACCGTTGGTGTAAACTACACT
CCGATTCCTCTGGTGACGATGGGGATCGATTACCGTCATGGTACGGGTAATGAAAATGAT
CTCCTTTACTCAATGCAGTTCCGTTATCAGTTTGATAAACCGTGGTCTCAGCAAATCGAG
CCACAGTATGTTAACGAGTTAAGAACATTATCGGGCAGCCGTTACGATCTGGTTCAGCGT
AATAACAATATTATTCTGGAGTACAAAAAGCAGGATATTCTTTCTCTGAATATTCCGCAT
GATATTAATGGTACTGAACACAGTACGCAGAAGATTCAATTGATCGTTAAGAGCAAATAT
GGTCTGGATCGTATCGTCTGGGATGATAGTGCATTACGCAGTCAGGGCGGTCAGATTCAG
CATAGCGGAAGCCAAAGCGCACAAGACTACCAGGCTATTTTGCCTGCTTATGTGCAAGGT
GGCAGCAATATTTATAAAGTGACCGCTCGCGCCTATGACCGAAATGGTAATAGTTCTAAT
AATGTACAGCTCACTATTACCGTTTTACCGAATGGGCAGGTTGTGGACCAGGTTGGGGTA
ACGGACTTTACGGCTGATAAGACATCGGCTAAAGCGGATAACGTTGATACCATTACTTAT
ACCGCGACGGTTAAAAAGAATGGTGTAGCTCAGGCTAATGCCCCTGTAACATTTAGTATT
GTATCCGGGACTGCAACTCTCGGAGCAAATAGTGCCAAAACGGATGGTAACGGTAAGGCA
ACCGTAACGTTGAAGTCGGGTACGCCAGGGCAGGTCGTCGTGTCTGCTAAAACCGCGGA
G
ATGACTTCGCCACTTAATGCCAGTGCGGTTATATTTGTTGATCAAACCAAGGCCAGCATT
ACTGAGATTAAGGCTGATAAAACAACAGCGAAGGCAAATGGTTCTGATGCGATTACCTAT
ACTGTTAAAGTAATGAAGAATAACCAACCAGAAGCAAACCATTCTGTTACATTCTCAACG
AACTTTGGTAATCTGGGGGGGAATTCTAATACCCAAATTGTGAAAACGGATAAAGATGGT
AGGGCTACGGTAAAACTGACATCTGGCGTTGCAGGTAATGCTATTGTTAGTGCAAAAGTC
AGCGAAGTTAATACAGAGGTTAAGGCTCCTGAGGTAAAATTCTTCTCAGTTCTGAGCATT
GATAGTAATGTTAATATTATTGGAACCTCTGCTACTGGCGCTTTGCCTAATATTTGGTTG
CAATATGGTCAGTTCAAATTGACTGCTAAAGGTGGTGATGGGAAATATCAATGGCGCTCT
CAAGATCCAAAAGTTGCATCAGTTGATGCTTTAACTGGTCGAGTTACTTTGTTGAAGAAA
GGAACAACAACAATTGAAGTTGTGTCGGGTGATAACCAAACTGCAACGTATACAATTAAT
ACACCTATAAAAATTATATCTGTGGAGACAAAAAATAAAGTAGTCTATAACGATGCTGAA
GCAATATGTAGAACGAATAATGGCCGTTTACCGCTATCTACGAATGAGTTAAAGGACGTG
TATAATAAATGGGGAGCGGCCAATAGTTATGAAGGCTATAAAGGTAAAAACACAATAACA
GCATGGACTCAGCAAACAGAGGATGATAAAACTAAAGGTTGGACTAGTACATTTGACATA
GTTACTAAAAATGAAATCCCTAGTAATGGAAGTAATAATAAGGTCAATGTGACAGCAGCT
AATGCCTTTGCTGTCTGTGTAAGATGA
>eaeA_zeta_AF449417
ATGATTACTCATGGTTTTTATGCCCGGACCCGGCACAAGCATAAGCTAAAAAAAACATTT
ATTATGCTTAGTGCTGGTTTAGGATTGTTTTTTATGTTAATCAGAATTCATTTGCAAAT

GGTGAAAATTATTTTAAATTGGGTTCGGATTCAAAACTGTTAACTCATAATAGCTATCAG
AATCGCCTTTTTATACGTTGAAAACAGGTGAAACTGTTGCCGATCTTTCTAAATCGCAA
GATATTAATTTATCGACGATTTGGTCGTTGAATAAGCATTTATACAGTTCTGAAAGCGAA
ATGATGAAGGCCGCGCCTGGTCAGCAGATCATTTTGCCACTCAAAAAACTTCCCTTTGAA
TACAGTGCCTTACCACTTTTAGGTTCGGCACCTCTTGTTGCTGCAGGTGGTGTCGCTGGT
CATACAAATAAACTGACTAAAATGTCCCCGGACGTGGCCAAAAGCAACATGACCGATGAC
AAGGCATTAAATTATGCGGCACAACAGGCGGCGAGTCTCGGTAGCCAGCTTCAGTCGCGA
TCTCTGAACGGCGATTACGCGAAAGATACCGCTCTTGGTATCGCTGGTAACCAGGCTTCG
TCACAGTTGCAGGCCTGGTTACAACATTATGGAACGGCAGAGGTTAATCTGCAGAGTGGT
AATAACTTTGACGGTAGTTCACTGGACTTCTTATTACCGTTCTATGATTCCGAAAAAATG
CTGGCATTTGGTCAGGTCGGAGCGCGTTACATTGACTCCCGCTTTACGGCAAATTTAGGT
GCGGGTCAGCGTTTTTTCCTTCCTGAAAATATGTTGGGCTATAACGTCTTCATTGATCAG
GATTTTTCTGGTGATAATACCCGTTTAGGTATTGGTGGCGAATACTGGCGAGACTATTTC
AAAAGTAGTGTTAACGGCTATTTCCGCATGAGCGGCTGGCATGAGTCATACAATAAGAAA
GACTATGATGAGCGCCCAGCAAATGGCTTCGATATCCGTTTTAATGGCTATCTGCCATCA
TACCCGGCATTAGGTGCCAGGCTGATGTATGAGCAGTATTATGGTGATAATGTTGCTTTG
TTTAATTCTGATAAGCTGCAGTCGAATCCTGGTGCGGCGACCGTTGGTGTAAACTATACT
CCGATTCCTCTGGTGACGATGGGGATCGATTACCGTCATGGTACGGGTAATGAAAATGAT
CTCCTTTACTCAATGCAGTTCCGTTATCAGTTTGATAAACCGTGGTCTCAGCAAATTGAG
CCACAGTATGTTAACGAGTTAAGAACATTATCAGGCAGCCGTTACGATCTGGTTCAGCGT
AATAACAATATTATTCTGGAGTACAAAAAGCAGGATATTCTTTCTCTGAATATTCCGCAT
GATATTAATGGTACTGAACGCAGTACGCAGAAGATTCAATTGATCGTTAAGAGCAAATAC
GGTCTGGATCGTATCGTCTGGGATGATAGTTCATTACGTAGCCAGGGCGGCCAGATTCAG
CATAGCGGAAGCCAAAGCGCACAAGATTACCAGGCTATTTTGCCTGCTTATGTGCAAGGT
GGTAGCAATGTTTATAAAGTGACGGCTCGCGCCTATGACCGTAATGGCAATAGCTCTAAC
AATGTACAGCTTACTATTACCGTTCTGTCGAATGGTCAGGTGGTCGACCAGGTTGGGGTA
ACGGACTTTACGGCTGATAAGACTTCGGCTAAAGCGGATGGCAACGATACCATTACCTAT
ACCGCGGTGGTTAAAAAGAATGGGGTGACTCAGGCTAATGTCCCTGTTTCATTTAATATT
GTTTCAGGAACTGCAACTTTAAGTGCCAAGAGTGCCAATACTAATAGTAGTGGTAAGGTG
ACTGTAACGCTGAAATCAGATAAGCCGGGTCAGGTAGTTGTGTCTGCCAAAACCGCGGAG
ATGACTTCAGCACTTAATGCCCATGCGGTTATATTTGTTGATCAAACTCAGGCCAGTATT
ACTGAGATTAATGCTGATAAAAAAACAGCGAAGGCAAATGGATCTGATGCGATTACCTAT
ACTGTTAAAGTGATGAAAGACGGTAAGCCGTTATCTGCCCAGGATGTGACCTTTACGGCA
ACCTTAGGTACGTTAAGTAAGTCCACTGAAAAAACGGATGCGAATGGCTATGCTAAAGTA
ACATTAACATCGAAGACTACAGGAAAATCGCTTGTTAGCGCTAGTATTAGCGGGAGCGCA
ATTGATGTCAAAGCACCTGAAGTTGAATTCTTCACCCCACTTGCTATTGATGACGGTAAT
GTTGAAATTGTAGGAACTGGAATCAAAGGGACGTTACCTACGGTATGGTTGCAATATGGA
CAAGTTAGGTTGAAAGCCAGTGGAGGTGATGGGAAATATACATGGAGTTCGGCAAATACT
GGAATTGCTTCGGTGGATTCCACTGGTCAAGTTACCTTAAGGGATAAGGGGAGTACAACT
ATTACCGTTGTATCAGGTGATAAGCAAACTGCAACTTATATAATTGCAAGACCTAGCAGT
ATGATTGTCTCTATAAACAAGCGTATGACTTATAAGAACGCAATGAGTTCATGCCAGAGC
TTGAGCGGGAGGTTGCCATCTTATCAAAAAGAACTAGCTGATGTATTCGATACATGGGGG
GCAGCAAATAAATATAAACATTATGAGACTCGTAACACCATGATATCGTGGATAAAACAA
ACAGATCAGGATATGAGTCAAGGAGTTGCAAGTACATACGATCTGATTAAGGAAAATCCG
CTTACTAATAAAGTAGATATAAATAATCCTAATGCTTATGCAACTTGCGTAAAATAA
>elt1_eltB_AP010910
ATGAATAAAGTAAAATGTTATGTTTTATTTACGGCGTTACTATCCTCTCTATGTGCATAC
GGAGCTCCCCAGTCTATTACAGAACTATGTTCGGAATATCGCAACACACAAATATATACG
ATAAATGACAAGATACTATCATATACGGAATCGATGGCAGGCAAAAGAGAAATGGTTATC
ATTACATTTAAGAGCGGCGCAACATTTCAGGTCGAAGTCCCGGGCAGTCAACATATAGAC
TCCCAAAAAAAAAGCCATTGAAAGGATGAAGGACACATTAAGAATCACATATCTGACCGAG
ACCAAAATTGATAAATTATGTGTATGGAATAATAAAACCCCCAATTCAATTGCGGCAATC
AGTATGGAAAACTAG
>elt1_eltA_AP010910
ATGAAAAATATAACTTTCATTTTTTTTATTTTATTAGCATCGCCATTATATGCAAATGGC
GACAAATTATACCGTGCTGACTCTAGACCCCCAGATGAAATAAAACGTTCCGGAGGTCTT
ATGCCCAGAGGGCATAATGAGTACTTCGATAGAGGAACTCAAATGAATATTAATCTTTAT

GATCACGCGAGAGGAACACAAACCGGCTTTGTCAGATATGATGACGGATATGTTTCCACT
TCTCTTAGTTTGAGAAGTGCTCACTTAGCAGGACAGTCTATATTATCAGGATATTCCACT
TACTATATATATGTTATAGCGACAGCACCAAATATGTTTAATGTTAATGATGTATTAGGC
GTATACAGCCCTCACCCATATGAACAGGAGGTTTCTGCGTTAGGTGGAATACCATATTCT
CAGATATATGGATGGTATCGTGTTAATTTTGGTGTGATTGATGAACGATTACATCGTAAC
AGGGAATATAGAGACCGGTATTACAGAAATCTGAATATAGCTCCGGCAGAGGATGGTTAC
AGATTAGCAGGTTTCCCACCGGATCACCAAGCTTGGAGAGAAGAACCCTGGATTCATCAT
GCACCACAAGGTTGTGGAAATTCATCAAGAACAATTACAGGTGATACTTGTAATGAGGAG
ACCCAGAATCTGAGCACAATATATCTCAGGAAATATCAATCAAAAGTTAAGAGGCAGATA
TTTTCAGACTATCAGTCAGAGGTTGACATATATAACAGAATTCGGAATGAATTATGA
>elt10_eltA_EU113249
ATGAAAAATATAACTTTCATTTTTTTTATTTTATTAGCATCGCCATTATATGCAAATGGC
GACAAATTATACCGTGCTGACTCTAGACCCCCAGATGAAATAAAACGTTCCGGAGGTCTT
ATGCCCAGAGGGCATAATGAGTACTTCGATAGAGGAACTCAAATGAATATTAATCTTTAT
GATCACGCGAGAGGAACACAAACCGGCTTTGTCAGATATGATGACGGATATGTTTCCACT
TCTCTTAGTTTGAGAAGTGCTCACTTAGCAGGACAGTCTATATTATCAGGATATTCCACT
TACTATATATATGTTATAGCGACAGCACCAAATATGTTTAATGTTAATGATGTATTAGGC
GTATACAGCCCTCACCCATATGAACAGGAGGTTTCTGCGTTAGGTGGAATACCATATTCT
CAGATATATGGATGGTATCGTGTTAATTTTGGTGTAATTGATGAACGATTACATCGTAAC
AGGGAATATAGAGACCGGTATTACAGAAATCTGAATATAGCTCCGGCAGAGGATGGTTAC
AGATTAGCAGGTTTCCCACCGGATCACCAAGCTTGGAGAGAAGAACCCTGGATTCATCAT
GCACCACGAGGTTGTGGAAATTCATCAAGAACAATTACAGGTGATACTTGTAATGAGGAG
ACCCAGAATCTGAGCACAATATATCTCAGGAAATATCAATCAAAAGTTAAGAGGCAGATA
TTTTCAGACTATCAGTCAGAGGTTGATATATATAGCAGAATTCGGGATGAATTATGA
>elt10_eltB_EU113249
ATGAATAAAGTAAAATGTTATGTTTTATTTACGGCGTTACTATCCTCTCTATGTGCATAC
GGAGCTCCCCAGTCTATTACAGAACTATGTTCGGAATATCGCAACACACAAATATATACG
ATAAATGACAAGATACTATCATATACGGAATCGATGGCAGGCAAAAGAGAAATGGTTATC
ATTACATTTAAGAGCGGCGCAACATTTCAGGTCGAAGTCCCGGGCAGTCAACATATAGAC
TCCCAAAAAAAAAGCCATTGAAAGGATGAAGGACACATTAAGAATCACATATCTGACCGAG
ACCAAAATTGATAAATTATGTGTATGGAATAATAAAACCCCCAATTCAATTGCGGCAATC
AGTATGGAAAACTAG
>elt11_eltA_EU113250
ATGAAAAATATAACTTTCATTTTTTTTATTTTATTAGCATCGCCATTATATGCAAATGGC
GACAAATTATACCGTGCTGACTCTAGACCCCCGGATGAAATAAAACGTTCCGGAGGTCTT
ATGCCCAGAGGGCATAATGAGTACTTCGATAGAGGAACTCAAATAAATATTAATCTTTAT
GATCACGCGAGAGGAACACAAACCGGCTTTGTCAGATATGATGACGGATATGTTTCCACC
TCTCTTAGTTTGAGAAGTGCTCACTTAGCAGGACAGTCTATATTATCAGGATATTCCACT
TACTATATATATGTTATAGCAACAGCACCAAATATGTTTAATGTTAATGATGTATTAGGC
GTATACAGCCCTCACCCATATGAACAGGAGGTTTCTGCGTTAGGTGGAATACCATATTCT
CAGATATATGGATGGTATCGTGTTAATTTTGGTGTGATTGATGAACGATTACATCGTAAC
AGGGAATATAGAGACCGGTATTACAGAAATCTGAATATAGCTCCGGCAGAGGATGGTTAC
AGATTAGCAGGTTTCCCACCGGATCACCAAGCTTGGAGAGAAGAACCCTGGATTCATCAT
GCACCACAAGGTTGTGGAAATTCATCAAGAGCAATTACAGGTGATACTTGTAATGAGGAG
ACCCAGAATCTGAGCACAATATATCTCAGGGAATATCAATCAAAAGTTAAGAGGCAGATA
TTTTCAGACTATCAGTCAGAGGTTGACATGTATAACAGAATTCGGGATGAATTATGA
>elt11_eltB_EU113250
ATGAATAAAGTAAAATGTTATGTTTTATTTACGGCGTTACTATCCTCTCTATGTGCACAC
GGAGCTCCCCAGTCTATTACAGAACTATGTTCGGAATATCGCAACACACAAATATATACG
ATAAATGACAAGATACTATCATATACGGAATCGATGGCAGGCAAAAGAGAAATGGTTATC
ATTACATTTAAGAGCGGCGCAACATTTCAGGTCGAAGTCCCTGGCAGTCAACATATAGAC
TCCCAAAAAAAAAGCCATTGAAAGGATGAAGGACACATTAAGAATCACATATCTGACCGAG
ACCAAAATTGATAAATTATGTGTATGGAATAATAAAACCCCCAATTCAATTGCGGCAATC
AGTATGGAAAACTAG
>elt12_eltA_EU113251
ATGAAAAATATAACTTTCATTTTTTTTATTTTATTAGCATCGCCATTATATGCAAATGGC
GACAAATTATACCGTGCTGACTCTAGACCCCCAGATGAAATAAAACATTCCGGAGGTCTT

ATGCCCAGAGGGCATAATGAGTACTTCGATAGAGGAACTCAAATAAATATTAATCTTTAT
GATCACGCGAGAGGAACACAAACCGGCTTTGTCAGATATGATGACGGATATGTTTCCACT
TCTCTTAGTTTGAGAAGTGCTCACTTAGCAGGACAGTCTATATTATCAGGATATTCCACT
TACTATATATATGTTATAGCGACAGCACCAAATATGTTTAATGTTAATGATGTATTAGGC
GTATACAGCCCTCACCCATATGAACAGGAGGTTTCTGCGTTAGGTGGAATACCATATTCT
CAGATATATGGATGGTATCGTGTTAATTTTGGTGTGATTGATGAACGATTACATCGTAAC
AGGGAATATAGAGACCGGTATTACAGAAATCTGAATATAGCTCCGGCAGAGGATGGTTAC
AGATTAGCAGGTTTCCCACCGGATCACCAAGCTTGGAGAGAAGAACCCTGGATTCATCAT
GCACCACAAGGTTGTGGAAATTCATCAAGAACAATTACAGGTGATACTTGTAATGAGGAG
ACCCAGAATCTGAGCACAATATATCTCAGGAAATATCAATCAAAAGTTAAGAGGCAGATA
TTTTCAGACTATCAGTCAGAGGTTGACATATATAACAGAATTCGGGATGAATTATGA
>elt12_eltB_EU113251
ATGAATAAAGTAAAATGTTATGTTTTATTTACGGCGTTACTATCCTCTCTATGTGCATAC
GGAGCTCCCCAGTCTATTACAGAACTATGTTCGGAATATCGCAACACACAAATATATACG
ATAAATGACAAGATACTATCATATACGGAATCGATGGCAGGCAAAAGAGAAATGGTTATC
ATTACATTTAAGAGCGGCGCAACATTTCAGGTCGAAGTCCCGGGCAGTCAACATATAGAC
TCCCAAAAAAAAGCCATTGAAAGGATGAAGGACACATTAAGAATCACATATCTGACCGAG
ACCAAAATTGATAAATTATGTGTATGGAATAATAAAACCCCCAATTCAATTGCGGCAATC
AGTATGGAAAACTAG
>elt13_eltA_EU113252
ATGAAAAATATAACTTTCATTTTTTTTATTTTATTAGCATCGCCATTATATGCAAATGGC
GACAAATTATACCGTGCTGACTCCAGACCCCCAGATGAAATAAAACATTCCGGAGGTCTT
ATACCCAGAGGGCATAATGAGTACTTCGATAGAGGAACTCAAATGAATATTAATCTTTAT
GATCACGCGAGAGGAACACAAACCGGCTTTGTCAGATATGATGACGGATATGTTTCCACT
TCTCTTAGTTTGAGAAGTGCTCACTTAGCAGGACAGTCTATATTATCAGGATATTCCACT
TACTATATATATGTTATAGCGACAGCACCAAATATGTTTAATGTTAATGATGTATTAGGC
GTATACAGCCCTCACCCATATGAACAGGAGGTTTCTGCGTTAGGTGGAATACCATATTCT
CAGATATATGGATGGTATCGTGTTAATTTTGGTGTGATTGATGAACGATTACATCGTAAC
AGGGAATATAGAGACCGGTATTACAGAAATCTGAATATAGCTCCGGCAGAGGATGGTTAC
AGATTAGCAGGTTTCCCACCGGATCACCAAGCTTGGAGAGAAGAACCCTGGATTCATCAT
GCACCACAAGGTTGTGGAAATTCATCAAGAACAATTACAGGTGATACTTGTAATGAGGAG
ACCCAGAATCTGAGCACAATATATCTCAGGAAATATCAATCAAAAGTTAAGAGGCAGATA
TTTTCAGACTATCAGTCAGAGGTTGACATATATAACAGAATTCGGGATGAATTATGA
>elt13_eltB_EU113252
ATGAATAAAGTAAAATGTTATGTTTTATTTACGGCGTTACTATCCTCTCTATGTGCATAC
GGAGCTCCCCAGTCTATTACAGAACTATGTTCGGAATATCGCAACACACAAATATATACG
ATAAATGACAAGATACTATCATATACGGAATCGATGGCAGGCAAAAGAGAAATGGTTATC
ATTACATTTAAGAGCGGCGCAACATTTCAGGTCGAAGTCCCGGGCAGTCAACATATAGAC
TCCCAAAAAAAAGCCATTGAAAGGATGAAGGACACATTAAGAATCACATATCTGACCGAG
ACCAAAATTGATAAATTATGTGTATGGAATAATAAAACCCCCAATTCAATTGCGGCAATC
AGTATGGAAAACTAG
>elt14_eltA_EU113253
ATGAAAAATATAACTTTCATTTTTTTTATTTTATTAGCATCGCCATTATATGCAAATGGC
GACAAATTATACCGTGCTGACTCTAGACCCCCAGATGAAATAAAACGTTCCGGAGGTCTT
ATGCCCAGAGGGCATAATGAGTACTTCGATAGAGGAACTCAAATGAATATTAATCTTTAT
GATCACGCGAGAGGAACACAAACCGGCTTTGTCAGATATGATGACGGATATGTTTCCACT
TCTCTTAGTTTGAGAAGTGCTCACTTAGCAGGACAGTCTATATTATCAGGATATTCCACT
TACTATATATATGTTATAGCGACAGCACCAAATATGTTTAATGTTAATGATGTATTAGGC
GTATACAGCCCTCACCCATATGAACAGGAGGTTTCTGCGTTAGGTGGAATACCATATTCT
CAGATATATGGATGGTATCGTGTTAATTTTGGTGTGATTGATGAACGATTACATCGTAAC
AGGGAATATAGAGACCGGTATTACAGAAATCTGAATATAGCTCCGGCAGAGGGTGGTTAC
AGATTAGCAGGTTTCCCACCGGATCACCAAGCTTGGAGAGAAGAACCCTGGATTCATCAT
GCACCACAAGGTTGTGGAAATTTATCAAGAACAATTACAGATGATACTTGTAATGAGGAG
ACCCAGAATCTGAGCACAATATATCTCAGGGAATATCAATCAAAAGTTAAGAGACAGATA
TTTACAGACTATCAGTCAGAGGTTGACATATATAACAGAATTCGGGATGAATTATGA
>elt14_eltB_EU113253
ATGAATAAAGTAAAATTTTATGTTTTATTTACGGTGTTACTATCCTCTCTATGTGCACAC

GGAGCTCCCCAGTCTATTACAGAACTATGTTCGGAATATCGCAACACACAAATATATACG
ATAAATGACAAAATACTATCATATACGGAATCGATGGCAGGCAAAAGAGAAATGGTTATC
ATTACATTTAAGAGCGGCGCAACATTTCAGGTCGAAGTCCCGGGCAGTCAACATATAGAC
TCCCAAAAAAAAGCCATTGAAAGGATGAAGGACACATTAAGAATCGCATATCTGACCGAG
ACCAAAATTGATAAATTATGTGTATGGAATAATAAAACCCCCAATTCAATTGCGGCAATC
AGTATGGAAAACTAG
>elt15_eltA_EU113254
ATGAAAAATATAACTTTCATTTTTGTTATTTTATTAGCATCGCCATTATATGCAAATGGC
GACAAATTATACCGTGCTGACTCTAGACCCCCTGATGAAATAAAACGTTCCGGAGGTCTT
ATGCCCAGAGGGCATAATGAGTACTTCGATAGAGGAACTCAAATGAATATTAATCTTTAT
GATCACGCGAGAGGAACACAAACCGGCTTTGTCAGATATGATGACGGATATGTTTCCACT
TCTCTTAGTTTGAGAAGTGCTCACTTAGCAGGACAGTCTATATTATCAGGATATTCCACT
TACTATATATATGTTATAGCGACAGCACCAAATATGTTTAATGTTAATGATGTATTAGGC
GTATACAGCCCTCACCCATATGAACAGGAGGTTTCTGCGTTAGGTGGAATACCATATTCT
CAGATATATGGATGGTATCGTGTTAATTTTGGTGTGATTGATGAACGATTACATCGTAAC
AGGGAATATAGAGACCGGTATTACAGAAATCTGAATATAGCTCCGGCAGAGGATGGTTAC
AGATTAGCAGGTTTCCCACCGGATCACCAAGCTTGGAGAGAAGAACCCTGGATTCATCAT
GCACCACAAGGTTGTGGAAATTTATCAAGAACAATTACAGATGATACTTGTAATGAGGAG
ACCCAGAATCTGAGCACAATATATCTCAGGGAATATCAATCAAAAGTTAAGAGACAGATA
TTTACAGACTATCAGTCAGAGGTTGACATATATAACAGAATTCGGGATGAATTATGA
>elt15_eltB_EU113254
ATGAATAAAGTAAAATTTTATGTTTTATTTACGGCGTTACTATCCTCTCTATGTGCACAC
GGAGCTCCCCAGTCTATTACAGAACTATGTTCGGAATATCGCAACACACAAATATATACG
ATAAATGACAAAATACTATCATATACGGAATCGATGGCAGGCAAAAGAGAAATGGTTATC
ATTACATTTAAGAGCGGCGCAACATTTCAGGTCGAAGTCCCGGGCAGTCAACATATAGAC
TCCCAAAAAAAAGCCATTGAAAGGATGAAGGACACATTAAGAATCGCATATCTGACCGAG
ACCAAAATTGATAAATTATGTGTATGGAATAATAAAACCCCCAATTCAATTGCGGCAATC
AGTATGGAAAACTAG
>elt16_eltA_EU113255
ATGAAAAATATAACTTTCATTTTTTTTATTTTATTAGCATCGCCATTATATGCTAATGGC
GACAAATTATGCCGTGCTGACTCTAGACCCCCTGATGAAATAAAACGTTCCGGAGGTCTT
ATGCCCAGAGGGCATAATGAGTACTTCGATAGAGGAACTCAAATGAATATTAATCTTTAT
GATCACGCGAGAGGAACACAAACCGGCTTTGTCAGATATGATGACGGATATGTTTCCACT
TCTCTTAGTTTGAGAAGTGCTCACTTAGCAGGACAGTCTATATTATCAGGATATTCCACT
TACTATATATATGTTATAGCGACAGCACCAAATATGTTTAATGTTAATGATGTATTAGGC
GTATACAGCCCTCACCCATATGAACAGGAGGTTTCTGCGTTAGGTGGAATACCATATTCT
CAGATATATGGATGGTATCGTGTTAATTTTGGTGTGATTGATGAACGATTACATCGTAAC
AGGGAATATAGAGACCGGTATTACAGAAATCTGAATATAGCTCCGGCAGAGGATGGTTAC
AGATTAGCAGGTTTCCCACCGGATCACCAAGCTTGGAGAGAAGAACCCTGGATTCATCAT
GCACCACAAGGTTGTGGAAATTTATCAAGAACAATTACAGATGATACTTGTAATGAGGAG
ACCCAGAATCTGAGCACAATATATCTCAGGGAATATCAATCAAAAGTTAAGAGACAGATA
TTTACAGACTATCAGTCAGAGGTTGACATATATAACAGAATTCGGGATGAATTATGA
>elt16_eltB_EU113255
ATGAATAAAGTAAAATTTTATGTTTTATTTACGGCGTTACTATCCTCTCTATGTGCACAC
GGAGCTCCCCAGTCTATTACAGAACTATGTTCGGAATATCGCAACACACAAATATATACG
ATAAATGACAAAATACTATCATATACGGAATCGATGGCAGGCAAAAGAGAAATGGTTATC
ATTACATTTAAGAGCGGCGCAACATTTCAGGTCGAAGTCCCGGGCAGTCAACATATAGAC
TCCCAAAAAAAAGCCATTGAAAGGATGAAGGACACATTAAGAATCGCATATCTGACCGAG
ACCAAAATTGATAAATTATGTGTATGGAATAATAAAACCCCCAATTCAATTGCGGCAATC
AGTATGGAAAACTAG
>elt2a_eltA_JQ031711
ATGATTAAGCATGTATTGTTGTTTTTTGTTTTTATATCATTTTCTGTTTCAGCAAACGAT
TTCTTTAGAGCAGACTCCAGAACACCTGATGAAATAAGACGTGCGGGAGGGCTTTTACCA
AGAGGGCAGCAGGAAGCTTATGAGCGCGGAACGCCAATTAACATCAATCTGTATGAGCAT
GCTCGCGGAACAGTAACGGGGAACACCAGATATAATGATGGATATGTATCTACAACTGTA
ACGTTGAGACAGGCTCATTTAATAGGGCAGAATATACTTGGCAGTTATAATGAATATTAC
ATATATGTAGTCGCACCAGCACCAAATTTATTTGATGTGAATGGAGTGTTAGGACGGTAT

AGTCCATATCCCAGTGAAAATGAATTTGCTGCATTAGGAGGGATTCCCTTATCACAAATT
ATAGGCTGGTATAGAGTATCTTTTGGTGCGATAGAAGGGGGAATGCAGCGAAACAGGGAT
TATCGAGGAGATTTATTTCGAGGGTTAACGGTTGCACCTAATGAAGATGGCTATCAACTT
GCAGGGTTTCCGAGTAACTTCCCAGCCTGGAGAGAAATGCCATGGAGTACATTTGCTCCT
GAACAGTGTGTGCCGAATAATAAAGAATTTAAAGGAGGGGTGTGCATTTCAGCGACAAAT
GTGCTATCGAAATATGATTTGATGAATTTTAAAAAACTCTTAAAACGCAGGCTGGCGTTA
ACGTTTTTCATGAGCGAAGATGATTTTATTGGTGTGCATGGAGAAAGAGATGAGCTCTAA
>elt2a_eltA_CP042298
ATGATTAAGCATGTATTGTTGTTTTTTGTTTTTATATCATTTTCTGTTTCAGCAAACGAT
TTCTTTAGAGCAGACTCCAGAACACCTGATGAAATAAGACGTGCAGGAGGGCTTTTGCCA
AGGGGGCAGCAGGAGGCTTATGAGCGCGGAACGCCAATTAACATCAATCTGTATGAGCAT
GCTCGCGGAACAGTAACGGGGAACACTAGATATAACGATGGGTATGTATCTACAACTACA
ACTTTGAGACAGGCTCATTTAATCGGGCAGAATATACTTGGCAGTTATAATGAATATTAC
ATATATGTAGTCGCACCAGCACCAAATTTATTTGATGTGAATGGTGTGTTAGGACGGTAT
AGTCCATATCCCAGTGAAAATGAATTTGCTGCATTAGGAGGGATTCCCTTATCACAAATT
ATAGGCTGGTATAGAGTATCTTTTGGTGCGATAGAAGGGGGAATGCAGCGAAACAGGGAT
TATCGAGGAGATTTATTTCGAGGGTTAACGGTTGCACCTAATGAAGATGGCTATCAACTT
GCAGGGTTTCCGAGCAATTTCCCAGCCTGGAGAGAAATGCCATGGAGTACATTTGCTCCT
GAACAGTGTGTGCCGAATAATAAAGAATTTAAAGGAGGGGTGTGCATTTCAGCGACAAAT
GTGCTATCGAAATATGATTTGATGAATTTTAAAAAACTCTTAAAACGCAGGCTGGCGTTA
ACGTTTTTCATGAGCGAAGATGATTTTATTGGTGTGCATGGAGAAAGAGATGAGCTCTAA
>elt2a_eltB_JQ031711
ATGAGCTCTAAGAAAATAATTGGTGCTTTTGTTCTGATGACTGGCATTCTGTCTGGTCAG
GTATATGCTGGTGTAAGTGAACACTTCAGGAATATTTGTAATCAAACCACTGCAGATATT
GTGGCAGGAGTGCAACTGAAGAAATATATTGCTGATGTAAACACAAATACTCGTGGGATC
TATGTGGTAAGTAATACTGGAGGTGTTTGGTATATTCCGGGTGGACGGGATTATCCGGAT
AATTTCTTAAGTGGAGAAATCAGAAAAACTGCAATGGCGGCCATTCTCTCAGATACGAAA
GTTAATTTGTGCGCAAAAACATCTTCAAGTCCAAATCACATTTGGGCTATGGAATTAGAT
AGAGAGTCATGA
>elt2b_eltA_JQ031712
ATGGCAAAAGTAATTTCTTTTTTTATTTCTTTATTTCTAATATCCTTTCCTTTATATGCA
AATGATTATTTCAGGGCTGATTCGAGAACGCCTGATGAAGTCAGACGCTCAGGGGGGTTG
ATTCCAAGAGGTCAGGATGAGGCTTATGAACGTGGCACGCCTATAAACATTAACCTATAT
GACCATGCACGAGGTACTGCAACAGGGAACACTAGATATAATGATGGATATGTATCAACA
ACAACGACTTTGAGACAGGCTCACTTCTTAGGGCAAAATATGCTTGGTGGGTATAATGAG
TACTATATTTATGTTGTTGCTGCAGCACCAAATTTGTTTGATGTAAATGGCGTTTTAGGC
AGGTATAGTCCGTATCCAAGTGAAAATGAATATGCTGCATTAGGTGGTATTCCCCTGTCA
CAAATAATTGGCTGGTATAGAGTATCTTTCGGTGCTATAGAGGGGGGAATGCATCGAAAC
AGGGATTACAGAAGAGATTTATTTAGAGGGTTATCTGCTGCTCCTAATGAGGATGGCTAT
CGAATTGCTGGATTTCCCGACGGATTTCCTGCGTGGGAAGAAGTTCCCTGGAGAGAATTT
GCGCCTAACTCTTGTCTACCAAATAATAAAGCTTCCAGTGATACTACCTGTGCCTCTTTA
ACAAATAAACTATCACAGCATGATTTAGCTGATTTTAAGAAATATATAAAGAGAAAATTT
ACCTTGATGACTTTACTAAGTATTAATAATGATGGTTTTTTTTCAAATAATGGAGGAAAA
GATGAGCTTTAA
>elt2b_eltB_JQ031712
ATGAGCTTTAAGAAAATTATCAAGGCATTTGTTATCATGGCTGCTTTGGTATCTGTTCAG
GCACATGCAGGTGCCAGTCAGTTTTTTAAAGATAACTGTAACAGAACCACAGCATCTCTT
GTGGAAGGTGTTGAGCTAACAAAATACATTTCTGACATTAATAATAACACCGATGGAATG
TACGTTGTAAGTTCTACTGGAGGTGTTTGGCGTATTTCTAGGGCTAAAGACTATCCAGAC
AATGTTATGACTGCTGAAATGAGGAAGATTGCGATGGCGGCTGTGTTGTCTGGTATGAGA
GTAAATATGTGTGCCAGCCCAGCTAGCAGTCCCAATGTTATTTGGGCCATCGAGTTAGAG
GCAGAATAA
>elt2c1_eltA_JQ031705
ATGATTAAGCATGTATTGTTGTTTTTTGTTTTTATATCATTTTCTGTCTCAGCAAACGAT
TTCTTTAGAGCAGACTCCAGAACACCAGATGAAATAAGACGTGCGGGAGGGCTTTTACCA
AGAGGGCAGCAGGAGGCTTATGAGCGCGGAACTCCAATTAACATCAATCTGTATGAGCAT
GCTCGCGGAACAGTAACGGGGAACACTAGATATAACGATGGGTATGTATCTACAACTACA

ACTTTGAGACAGGCTCATTTAATCGGGCAGAATATACTTGGCAGTTATAATGAATATTAC
ATATATGTAGTTGCACCAGCACCCAATTTATTTGATGTGAATGGTGTGTTAGGGCGGTAT
AGTCCATATCCCAGTGAAAATGAATTTGCTGCATTAGGAGGGATTCCCTTATCACAAATT
ATAGGCTGGTATAGAGTATCTTTTGGTGCGATAGAAGGGGGAATGCAGCGAAACAGGCAT
TACAGAGGAGATTTATTTCAAGGCTTATCGGTTGCCCCTAATCATGATGGCTATCATCTC
GCAGGATTTCCAGAGGGTTTTGCTGCATGGCGAGAGCTGCCATGGAGTGCATTTGCTCCG
GAACAGTGCGAGCAAGATTACATGGTTAGAAATTTAGATGCCTGCGATTCTTATACAAAT
ATATTATCTCAAAATGATTTGGTCGCTTTTAAAAGATTTATGCGAATTCGTTCTTCCCTT
ATGATTTTACAAAGTATTGAGGATGATTTACAAAACAATGAAAATAAAGATGAACTTTAA
>elt2c1_eltB_JQ031705
ATGAACTTTAAAAAGTCAATTGCGTTGTTGTTTATTGCCTTAAATATTGCATCACTACCA
ACATATGCTGGCGTAAGTAAAACTTTTAAGGATAAATGCGCTTCGACTACGGCCAAGCTT
GTACAGAGTGTTCAGTTGGTAAATATCTCATCTGATGTAAATAAGGACAGCAAGGGAATT
TATATATCAAGCTCAGCAGGAAAAACATGGTTTATTCCGGGGGGGCAGTATTACCCTGAT
AACTATCTAAGTAATGAAATGAGAAAAATAGCAATGGCTGCAGTTCTTTCTAACGTAAGG
GTAAATCTATGTGCGAGTGAAGCATATACTCCGAATCATGTATGGGCAATTGAATTAGCA
CCATAA
>elt2c2_eltA_JQ031706
ATGATTAAGCATGTATTGTTGTTTTTTGTTTTTATATCATTTTCTGTCTCAGCAAACGAT
TTCTTTAGAGCAGACTCCAGAACACCAGATGAAATAAGACGTGCGGGAGGGCTTTTACCA
AGAGGGCAGCAGGAGGCTTATGAGCGCGGAACACCAATTAACATCAATCTGTATGATCAT
GCTCGCGGAACTGTAACGGGGAACACCAGATATAATGATGGGTATGTATCTACTACTACT
ACGCTGAGACAGGCTCATTTAATAGGGCAGAATCTGCTTGGCAGTTATAATGAATATTAC
ATATATGTAGTCGCACCAGCACCAAATTTATTTGATGTGAATGGTGTGTTAGGACGGTAT
AGTCCATATCCCAGTGAAAACGAATTTGCTGCATTAGGTGGGATTCCCTTATCACAAATT
ATAGGCTGGTATAGAGTATCTTTTGGCGTGATAGAAGGGGGAATGCAGCGAAACAGGCAT
TATAGAAGAGATTTATTTCAAGGCTTATCGGTTGCTCCTAATCATGATGGCTATCATCTC
GCAGGATTTCCAGACGGTTTTGCCGCATGGCGAGAGCTGCCGTGGAGTGCATTTGCTCCG
GAACAGTGTGAGCAAGATTACATGGTTAGAAATTTAGATGCCTGCGATTCTTATACAAAT
ATATTATCTCAAAATGATTTGGTCGCTTTTAAAAGATTTATGCGAATTCGTTCTTCCCTT
ATGATTTTACAAAGTATTGAGGATGATTTACAAGACAATGAAAATAAAGATGAACTTTAA
>elt2c2_eltB_JQ031706
ATGAACTTTAAAAAGTCAATTGCGTTGTTGTTTATTGCCTTAAATATTGCATCACTACCA
ACATATGCTGGCGTAAGTAAAACTTTTAAGGATAAATGCGCTTCTACTACGGCCAAACTT
GTACAGAGTGTTCAGTTGGTAAAACTAGCATCTGATACCAACAAGGACAGTAAGGGTATT
TATATAACCGATTCTACAGGAAAAACCAGATTCATTCCTGGGGGGCAGTACTATCCCGAG
AATTATCTGAGCAATGAGATGAGGAAAATAGCAATGGCTGCGGTGCTTTCTAATGTTAGG
GTAAATATCTGTGCGAGCGAAGCATATACTCCTAATCACGTATGGGCAATTGAATTAGCA
GCGGAATAG
>elt2c3_eltA_JQ031707
ATGATTAAACATTTATTGTTGTTTTTTGTTTTTATATCATTTTCTGTCTCAGCAAATGAT
TTTTTTAGAGCAGACACCAGAACACCTAGTGAAATAAGACAAGCTGGAGGACTTTTGCCT
CGAGGTCAGCAAGAGGCTTATGAGCGCGGAACACCAATTAACATCAATCTGTATGATCAT
GCTCGCGGAACTGTAACGGGGAACACCAGATATAATGATGGGTATGTATCTACTACTACT
ACGCTGAGACAGGCTCATTTAATAGGGCAGAATCTGCTTGGCAGTTATAATGAATATTAC
ATATATGTAGTCGCACCAGCACCAAATTTATTTGATGTGAATGGTGTGTTAGGACGGTAT
AGTCCATATCCCAGTGAAAACGAATTTGCTGCATTAGGTGGGATTCCCTTATCACAAATT
ATAGGCTGGTATAGAGTATCTTTTGGCGTGATAGAAGGGGGAATGCAGCGAAACAGGCAT
TATAGAAGAGATTTATTTCAAGGCTTATCGGTTGCTCCTAATCATGATGGCTATCATCTC
GCAGGATTTCCAGACGGTTTTGCCGCATGGCGAGAGCTGCCGTGGAGTGCATTTGCTCCT
GCTGCATGCGAACATGATTACATGGTTCGAATTTTAGATGCCTGCGATTCTTATACGAAT
AGAATATCTAAAAATGATTTATTTGCTTTTAAAAGATTTATGCGGATTCGCTCTTCTCTG
ATGATCTTACAAAGTATCGAGGATGATTTACAATATAATGAAAATAAAAATGAACTTTAA
>elt2c3_eltB_JQ031707
ATGAACTTTAAAAAGTCAATTGCATTATTGTTTGTTGTCTTAAATATTACATCTCTACCA
ACATATGCTGATGTAAGTAAGAATTTTAAGGATAATTGTGGTTCTACTACGGCCAAAATT
GTACAAAGTGTTCGGTTGGTAAAACTAGCATCTGATACCAACAAGGACAGCAAAGGTATT

TATATAACCGATTCTACAGGAAAAACCAGATTCATTCCTGGGGGGCAGTACTATCCCGAG
AATTATCTGAGCAATGAGATGAGGAAAATAGCAATGGCTGCGGTGCTTTCTAATGTTAGG
GTAAATATCTGTGCGAGCGAAGCATATACTCCTAATCACGTATGGGCAATTGAATTAGCA
GCGGAATAG
>elt2c4_eltA_JQ031708
ATGATTAAGCATGTATTGTTGTTTTTTGTTTTTATATCATTTTCTGTCTCAGCAAACGAT
TTCTTTAGAGCAGACTCCAGAACACCAGATGAAATAAGACGTGCGGGAGGGCTTTTACCA
AGAGGGCAGCAGGAGGCTTATGAGCGCGGAACTCCAATTAACATCAATCTGTATGAGCAT
GCTCGCGGAACAGTAACGGGGAACACTAGATATAACGATGGGTATGTATCTACAACTACA
ACTTTGAGACAGGCTCATTTAATCGGGCAGAATATACTTGGCAGTTATAATGAATATTAC
ATATATGTAGTTGCACCAGCACCCAATTTATTTGATGTGAATGGTGTGTTAGGGCGGTAT
AGTCCATATCCCAGTGAAAATGAATTTGCTGCATTAGGAGGGATTCCCTTATCACAAATT
ATAGGCTGGTATAGAGTATCTTTTGGTGCGATAGAAGGGGGAATGCAGCGAAACAGGCAT
TACAGAGGAGATTTATTTCAAGGCTTATCGGTTGCCCCTAATCATGATGGCTATCATCTC
GCAGGATTTCCAGAGGGTTTTGCTGCATGGCGAGAGCTGCCATGGAGTGCATTTGCTCCG
GAACAATGCGAGCAAGATTACATGGTTAGAAATTTAGATGCCTGCGATTCTTATACAAAT
ATATTATCTCAAAATGATTTGGTCGCTTTTAAAAGATTTATGCGAATTCGTTCTTCCCTT
ATGATTTTACAAAGTATTGAGGATGATTTACAAAACAATGAAAATAAAGATGAACTTTAA
>elt2c4_eltB_JQ031708
ATGAACTTTAAAAAGTCAATTGCGTTGTTGTTTATTGCCTTAAATATTGCATCACTACCA
ACATATGCTGGCGTAAGTAAAACTTTTAAGGATAAATGCGCTTCTACTACGGCCAAACTT
GTACAGAGTGTTCAGTTGGTAAAACTAGCATCTGATACCAACAAGGACAGTAAGGGTATT
TATATAACCGATTCTACAGGAAAAACCAGATTCATTCCTGGGGGGCAGTACTATCCCGAG
AATTATCTGAGCAATGAGATGAGGAAAATAGCAATGGCTGCGGTGCTTTCTAATGTTAGG
GTAAATATCTGTGCGAGCGAAGCATATACTCCTAATCACGTATGGGCAATTGAATTAGCA
GCGGAATAG
>elt2c5_eltA_JQ031709
ATGATTAAACATTTATTGTTGTTTTTTGTTTTTATATCATTTTCTGTCTCAGCAAATGAT
TTTTTTAGAGCAGACACCAGAACACCTAGTGAAATAAGACAAGCTGGAGGACTTTTGCCT
CGAGGTCAGCAAGAGGCTTATGAGCGCGGAACACCAATTAACATCAATCTGTATGATCAT
GCTCGCGGAACTGTAACGGGGAACACCAGATATAATGATGGGTATGTATCTACTACTACT
ACGCTGAGACAGGCTCATTTAATAGGGCAGAATCTGCTTGGCAGTTATAATGAATATTAC
ATATATGTAGTCGCACCAGCACCAAATTTATTTGATGTGAATGGTGTGTTAGGACGGTAT
AGTCCATATCCCAGTGAAAACGAATTTGCTGCATTAGGTGGGATTCCCTTATCACAAATT
ATAGGCTGGTATAGAGTATCTTTTGGCGTGATAGAAGGGGGAATGCAGCGAAACAGGCAT
TATAGAAGAGATTTATTTCAAGGCTTATCGGTTGCTCCTAATCATGATGGCTATCATCTC
GCAGGATTTCCAGACGGTTTTGCCGCATGGCGAGAGCTGCCGTGGAGTGCATTTGCTCCT
GCTGCATGCGAACATGATTACATGGTTCGAATTTTAGATGCCTGCGATTCTTATACGAAT
AGAATATCTAAAAATGATTTGTTTGCTTTTAAAAGATTTATGCGGATTCGCTCTTCTCTG
ATGATCTTACAAAGTATCGAGGATGATTTACAATATAATGAAAATAAAGATGAACTTTAA
>elt2c5_eltB_JQ031709
ATGAACTTTAAAAAGTTAATTGCGTTATTGTTTATTGTCTTAAATATTGCATCACTACCA
ACATATGCTGGTGTAAGTAAAACTTTTAAGGATAACTGCGCTTCTACTACGGCAAAACTT
GTACAGAGTGTTCAGTTGGTAAATATCTCGTCTGATGTAAATAAGGACAGTAAGGGAATT
TATATATCAAGCTCAGCAGGAAAAACATGGTTTATTCCGGGGGGGCAGTATTACCCTGAT
AACTATCTAAGTAATGAAATGAGAAAAATAGCAATGGCTGCAGTTCTTTCTAACGTAAGG
GTAAATCTATGTGCGAGTGAAGCATATACTCCGAATCATGTATGGGCAATTGAATTGGCA
CCATAA
>elt2c6_eltA_JQ031710
ATGATTAAGCATGTATTGTTGTTTTTTGTTTTTATATCATTTTCTGTCTCAGCAAACGAT
TTCTTTAGAGCAGACTCCAGAACACCAGATGAAATAAGACGTGCGGGAGGGCTTTTACCA
AGAGGGCAGCAGGAGGCTTATGAGCGCGGAACACCAATTAACATCAATCTGTATGATCAT
GCTCGCGGAACTGTAACGGGGAACACCAGATATAATGATGGGTATGTATCTACTACTACT
ACGCTGAGACAGGCTCATTTAATAGGGCAGAATCTGCTTGGCAGTTATAATGAATATTAC
ATATATGTAGTCGCACCAGCACCAAATTTATTTGATGTGAATGGTGTGTTAGGACGGTAT
AGTCCATATCCCAGTGAAAACGAATTTGCTGCATTAGGTGGGATTCCCTTATCACAAATT
ATAGGCTGGTATAGAGTATCTTTTGGCGTGATAGAAGGGGGAATGCAGCGAAACAGGCAT

TATAGAAGAGATTTATTTCAAGGCTTATCGGTTGCTCCTAATCATGATGGCTATCATCTC
GCAGGATTTCCAGACGGTTTTGCCGCATGGCGAGAGCTGCCGTGGAGTGCATTTGCTCCT
GCTGCATGCGAACATGATTACATGGTTCGAATTTTAGATGCCTGCGATTCTTATACGAAT
AGAATATCTCAAAATGATTTGGTCGCTTTTAAAAGATTTATGCGAATTCGTTCTTCCCTT
ATGATTTTACAAAGTATTGAGGATGATTTACAAGACAATGAAAATAAAGATGAACTTTAA
>elt2c6_eltB_JQ031710
ATGAACTTTAAAAAGTCAATTGCGTTGTTGTTTATTGCCTTAAATATTGCATCACTACCA
ACATATGCTGGCGTAAGTAAAACTTTTAAGGATAAATGCGCTTCTACTACGGCCAAACTT
GTACAGAGTGTTCAGTTGGTAAAACTAGCATCTGATACCAACAAGGACAGTAAGGGTATT
TATATAACCGATTCTACAGGAAAAACCAGATTCATTCCTGGGGGGCAGTACTATCCCGAG
AATTATCTGAGCAATGAGATGAGGAAAATAGCAATGGCTGCGGTGCTTTCTAATGTTAGG
GTAAATATCTGTGCGAGCGAAGCATATACTCCTAATCACGTATGGGCAATTGAATTAGCA
GCGGAATAG
>elt2d_eltA_AP019856
ATGAAAAAATTAGTGTTGTTATTCTTTTCTATTGCTTTTTTCTCTGTATCTTCCAGTGCA
AATGAATTTTACAGAGCGGATGCCAGAAATCCTGAAGAGATTAGGCGGGCAGGAGGGTTA
TTATCTCGGGGGCAGAATGAAGCATATGACCGTGGAACGCCAGTAAATATAAATTTATAT
GATCATGCCCGTGGCACTGTAACTGGTAATGCCCGTTACGACGACGGATACGTATCTACT
ACAACAACACTTAGACAGGCACACTTGATAGGCCAAAATATCCTGAGCGGCTATAGCGAA
TATTATATATATGTCGTTACTGCCGCTCCAAATATGTTTGATGTAAATGGCGTTTTAGGA
AGATACAGTCCGCACCCTAGCGAAAATGAATTCGCAGCACTGGGAGGAATTCCATTGTCA
CAAATTATTGGGTGGTACAGGGTATCTTTTGGCGTTATACAGGGAGAAATGCAACGTAAC
AGGGCTTACAGAGGGGATTTGTATCGGGGATTAGCTGTTGCTCCGAATGAGGATGGTTAT
CATCTTGCAGGGTTTCCGGCTAATTTTCCTGCATGGCGGGAAGCACCCTGGAATGCATTT
GCTCCGGATCAGTGTGTGCCGAAAAATAAAGAAATTGAAGATTCGCCGTGTGCTTCTGCC
ACAAATTCAATGTCGAAACATGACCTGACAAATTTTAAGAAACTTTTAAAACGCAGGTCG
GCCTTAATGATTTTAATGAACGAAGACGATTCATTGTTGTAA
>elt2d_eltB_AP019856
ATGAACACTAAAAAAATTGATCAGCGCTCTTGTTTTGATGACTGGTATTGCATCTGGTCAG
GTATATGCAGGTGTTAGTGAACAACTCAGAAATATCTGTAATCAAACCACTGCAAATATT
GTAGCAGGGGTGCAACTTAAGAAATATATTTCAGATATAAACACAAATACTGAAGGGGTT
TATGTGGTAGCTGATGTTGAACGTGTTTGGTTTATTCCTTCCGCAAAGAATTATCCTGAT
AGCGTCTTAACTGCCGAACTCAGGAAAACTGCAATGGCAGCCATCCTCTCAGACACGAAA
GTAAATTTGTGTACAAAATATTCTTCTGGCCCACATCATATTTGGGCTATGGAATTAGAT
CGAGAGTCGTGA
>elt3_eltA_EU113242
ATGAAAAATATAACTTTCATTTTTTTTATTTATTAGCATCGCCATTATATGCAAATGGC
GACAAATTATACCGTGCTGACTCTAGACCCCCAGATGAAATAAAACGTTCCGGAGGTCTT
ATGCCCAGAGGGCATAATGAGTACTTCGATAGAGGAACTCAAATGAATATTAATCTTTAT
GATCACGCGAGAGGAACACAAACCGGCTTTGTCAGATATGATGACGGATATGTTTCCACT
TCTCTTAGTTTGAGAAGTGCTCACTTAGCAGGACAGTCTATATTATCAGGATATTCCACT
TACTATATATATGTTATAGCGACAGCACCAAATATGTTTAATGTTAATGATGTATTAGGC
GTATACAGCCCTCACCCATATGAACAGGAGGTTTCTGCGTTAGGTGGAATACCATATTCT
CAGATATATGGATGGTATCGTGTTAATTTTGGTGTGATTGATGAACGATTACATCGTAAC
AGGGAATATAGAGACCGGTATTACAGAAATCTGAATATAGCTCCGGCAGAGGATGGTTAC
AGATTAGCAGGTTTCCCACCGGATCACCAAGCTTGGAGAGAAGAACCCTGGATTCATCAT
GCACCACAAGGTTGTGGAAATTCATCAAGAACAATTACAGGTGATACTTGTAATGAGGAG
ACCCAGAATCTGAGCACAATATATCTCAGGGAATATCAATCAAAAGTTAAGAGGCAGATA
TTTTCAGACTATCAGTCAGAGGTTGACATATATAACGGAATTCGGGATGAATTATGA
>elt3_eltB_EU113242
ATGAATAAAGTAAAATTTTATGTTTTATTTACGGCGTTACTATCCTCTCTATGTGCACAC
GGAGCTCCCCAGTCTATTACAGAACTATGTTCGGAATATCACAACACACAAATATATACG
ATAAATGACAAGATACTATCATATACGGAATCGATGGCAGGCAAAAGAGAAATGGTTATC
ATTACATTTAAGAGCGGCGCAACATTTCAGGTCGAAGTCCCGGGCAGTCAACATATAGAC
TCCCAAAAAAAAAGCCATTGAAAGGATGAAGGACACATTAAGAATCACATATCTGACCGAG
ACCAAAATTGATAAATTATGTGTATGGAATAATAAAACCCCCAATTCAATTGCGGCAATC
AGTATGGAAAACTAG

>elt4_eltA_EU113243
ATGAAAAATATAACTTTCATTTTTTTTATTTTATTAGCATCGCCATTATATGCAAATGGC
GACAGATTATACCGTGCTGACTCTAGACCCCCAGATGAAATAAAACGTTCCGGAGGTCTT
ATGCCCAGAGGGCATAATGAGTACTTCGATAGAGGAACTCAAATGAATATTAATCTTTAT
GATCACGCGAGAGGAACACAAACCGGCTTTGTCAGATATGATGACGGATATGTTTCCACT
TCTCTTAGTTTGAGAAGTGCTCACTTAGCAGGACAGTCTATATTATCAGGATATTCCACT
TACTATATATATGTTATAGCGACAGCACCAAATATGTTTAATGTTAATGATGTATTAGGC
GTATACAGCCCTCACCCATATGAACAGGAGGTTTCTGCGTTAGGTGGAATACCATATTCT
CAGATATATGGATGGTATCGTGTTAATTTTGGTGTGATTGATGAACGATTACATCGTAAC
AGGGAATATAGAGACCGGTATTACAGAAATCTGAATATAGCTCCGGCAGAGGATGGTTAC
AGATTAGCAGGTTTCCCACCGGATCACCAAGCTTGGAGAGAAGAACCCTGGATTCATCAT
GCACCACAAGGTTGTGGAAATTCATCAAGAACAATCACAGGTGATACTTGTAATGAGGAG
ACCCAGAATCTGAGCACAATATATCTCAGGGAATATCAATCAAAAGTTAAGAGGCAGATA
TTTTCAGACTATCAGTCAGAGGTTGACATATATAACAGAATTCGGGATGAATTATGA
>elt4_eltB_EU113243
ATGAATAAAGTAAAATGTTATGTTTTATTTACGGCGTTACTATCCTCTCTATATGCACAC
GGAGCTCCCCAGACTATTACAGAACTATGTTCGGAATATCGCAACACACAAATATATACG
ATAAATGACAAGATACTATCATATACGGAATCGATGGCAGGCAAAAGAGAAATGGTTATC
ATTACATTTAAGAGCGGCGAAACATTTCAGGTCGAAGTCCCGGGCAGTCAACATATAGAC
TCCCAGAAAAAAGCCATTGAAAGGATGAAGGACACATTAAGAATCACATATCTGACCGAG
ACCAAAATTGATAAATTATGTGTATGGAATAATAAAACCCCCAATTCAATTGCGGCAATC
AGTATGAAAAACTAG
>elt5_eltA_EU113244
ATGAAAAATATAACTTTCATTTTTTTTATTTTATTAGCATCGCCATTATATGCAAATGGC
GACAAATTATACCGTGCTGACTCTAGACCCCCAGATGAAATAAAACGTTCCGGAGGTCTT
ATGCCCAGAGGGCATAATGAGTACTTCGATAGAGGAACTCAAATGAATATTAATCTTTAT
GATCACGCGAGAGGAACACAAACCGGCTTTGTCAGATATGATGACGGATATGTTTCCACT
TCTCTTAGTTTGAGAAGTGCTCACTTAGCAGGACAGTCTATATTATCAGGATATTCCACT
TACTATATATATGTTATAGCGACAGCACCAAATATGTTTAATGTTAATGATGTATTAGGC
GTATACAGCCCTCACCCATATGAACAGGAGGTTTCTGCGTTAGGTGGAATACCATATTCT
CAGATATATGGATGGTATCGTGTTAATTTTGGTGTAATTGATGAACGATTACATCGTAAC
AGGGAATATAGAGACCGGTATTACAGAAATCTGAATATAGCTCCGGCAGAGGATGGTTAC
AGATTAGCAGGTTTCCCACCGGATCACCAAGCTTGGAGAGAAGAACCCTGGATTCATCAT
GCACCACAAGGTTGTGGAAATTCATCAAGAACAATTACAGGTGATACTTGTAATGAGGAG
ACCCAGAATCTGAGCACAATATATCTCAGGGAATATCAATCAAAAGTTAAGAGGCAGATA
TTTTCAGACTATCAGTCAGAGGTTGACATATATAACGGAATTCGGGATGAATTATGA
>elt5_eltB_EU113244
ATGAATAAAGTAAAATTTTATGTTTTATTTACGGCGTTACTATCCTCTCTATGTGCATAC
GGAGCTCCCCAGTCTATTACAGAACTATGTTCGGAATATCGCAACACACAAATATATACG
ATAAATGACAAGATACTATCATATACGGAATCGATGGCAGGCAAAAGAGAAATGGTTATC
ATTACATTTAAGAGCGGCGCAACATTTCAGGTCGAAGTCCCGGGCAGTCAACATATAGAC
TCCCAAAAAAAAGCCATTGAAAGGATGAAGGACACATTAAGAATCACATATCTGACCGAG
ACCAAAATTGATAAATTATGTGTATGGAATAATAAAACCCCCAATTCAATTGCGGCAATC
AGTATGGAAAACTAG
>elt6_eltA_EU113245
ATGAAAAATATAACTTTCATTTTTTTTATTTTATTAGCATCGCCATTATATGCAAATGGC
GACAGATTATACCGTGCTGACTCTAGACCCCCAGATGAAATAAAACGTTCCGGAGGTCTT
ATGCCCAGAGGGCATAATGAGTACTTCGATAGAGGAACTCAAATGAATATTAATCTTTAT
GATCACGCGAGAGGAACACAAACCGGCTTTGTCAGATATGATGACGGATATGTTTCCACT
TCTCTTAGTTTGAGAAGTGCTCACTTAGCAGGACAGTCTATATTATCAGGATATTCCACT
TACTATATATATGTTATAGCGACAGCACCAAATATGTTTAATGTTAATGATGTATTAGGC
GTATACAGCCCTCACCCATATGAACAGGAGGTTTCTGCGTTAGGTGGAATACCATATTCT
CAGATATATGGATGGTATCGTGTTAATTTTGGTGTGATTGATGAACGATTACATCGTAAC
AGGGAATATAGAGACCGGTATTACAGAAATCTGAATATAGCTCCGGCAGAGGATGGTTAC
AGATTAGCAGGTTTCCCACCGGATCACCAAGCTTGGAGAGAAGAACCCTGGATTCATCAT
GCACCACAAGGTTGTGGAAATTCATCAAGAACAATCACAGGTGATACTTGTAATGAGGAG
ACCCAGAATCTGAGCACAATATATCTCAGGGAATATCAATCAAAAGTTAAGAGGCAGATA

TTTTCAGACTATCAGTCAGAGGTTGACATATATAACAGAATTCGGGATGAATTATGA
>elt6_eltB_EU113245
ATGAATAAAGTAAAATGTTATGTTTTATTTACGGCGTTACTATCCTCTCTATATGCACAC
GGAGCTCCCCAGACTATTACAGAACTATGTTCGGAATATCGCAACACACAAATATATACG
ATAAATGACAAGATACTATCATATACGGAATCGATGGCAGGCAAAAGAGAAATGGTTATC
ATTACATTTAAGAGCGGCGAAACATTTCAGGTCGAAGTCCCGGGCAGTCAACATATAGAC
TCCCAGAAAAAAGCCATTGAAAGGATGAAGGACACATTAAGAATCACATATCTGACCGAG
ACCAAAATTGATAAATTATGTGTATGGAATAATAAAACCCCCAATTCAATTGCGGCAATC
AGTATGGAAAACTAG
>elt7_eltA_EU113246
ATGAAAAATATAACTTTCATTTTTGTTATTTTATTAGCATCGCCATTATATGCAAATGGC
GACAAATTATACCGTGCTGACTCTAGATCCCCTGATGAAATAAAACGTTCCGGAGGTCTT
ATGCCCAGAGGGCATAATGAGTACTTCGATAGAGGAACTCAAATGAATATTAATCTTTAT
GATCACGCGAGAGGAACACAAACCGGCTTTGTCAGATATGATGACGGATATGTTTCCACT
TCTCTTAGTTTGAGAAGTGCTCACTTAGCAGGACAGTCTATATTATCAGGATATTCCACT
TACTATATATATGTTATAGCGACAGCACCAAATATGTTTAATGTTAATGATGTATTAGGC
GTATACAGCCCTCACCCATATGAACAGGAGGTTTCTGCGTTAGGTGGAATACCATATTCT
CAGATATATGGATGGTATCGTGTTAATTTTGGTGTGATTGATGAACGATTACATCGTAAC
AGGGAATATAGAGACCGGTATTACAGAAATCTGAATATAGCTCCGGCAGAGGATGGTTAC
AGATTAGCAGGTTTCCCACCGGATCACCAAGCTTGGAGAGAAGAACCCTGGATTCATCAT
GCACCACAAGGTTGTGGAAATTTATCAAGAACAATTACAGATGATACTTGTAATGAGGAG
ACCCAGAATCTGAGCACAATATATCTCAGGGAATATCAATCAAAAGTTAAGAGACAGATA
TTTACAGACTATCAGTCAGAGGTTGACATATATAACAGAATTCGGGATGAATTATGA
>elt7_eltB_EU113246
ATGAATAAAGTAAAATTTTATGTTTTATTTACGGCGTTACTATCCTCTCTATGTGCACAC
GGAGCTCCCCAGTCTATTACAGAACTATGTTCGGAATATCGCAACACACAAATATATACG
ATAAATGACAAAATACTATCATATACGGAATCGATGGCAGGCAAAAGAGAAATGGTTATC
ATTACATTTAAGAGCGGCGCAACATTTCAGGTCGAAGTCCCGGGCAGTCAACATATAGAC
TCCCAAAAAAAAGCCATTGAAAGGATGAAGGACACATTAAGAATCGCATATCTGACCGAG
ACCAAAATTGATAAATTATGTGTATGGAATAATAAAACCCCCAATTCAATTGCGGCAATC
AGTATGGAAAACTAG
>elt8_eltA_EU113247
ATGAAAAATATAACTTTCATTTTTTTTATTTTATTAGCATCGCCATTATATGCAAATGGC
GACAAATTATACCGTGCTGACTCTAGACCCCCAGATGAAATAAAACGTTCCGGAGGTCTT
ATGCCCAGAGGGCATAATGAGTACTTCGATAGAGGAACTCAAATGAATATTAATCTTTAT
GATCACGCGAGAGGAACACAAACCGGCTTTGTCAGATATGATGACGGATATGTTTCCACT
TCTCTTAGTTTGAGAAGTGCTCACTTAGCAGGACAGTCTATATTATCAGGATATTCCACT
TACTATATATATGTTATAGCGACAGCACCAAATATGTTTAATGTTAATGATGTATTAGGC
GTATACAGCCCTCACCCATATGAACAGGAGGTTTCTGCGTTAGGTGGAATACCATATTCT
CAGATATATGGATGGTATCGTGTTAATTTTGGTGTGATTGATGAGCGATTACATCGTAAC
AGGGAATATAGAGACCGGTATTACAGAAATCTGAATATAGCTCCGGCAGAGGATGGTTAC
AGATTAGCAGGTTTCCCACCGGATCACCAAGCTTGGAGAGAAGAACCCTGGATTCATCAT
GCACCACAAGGTTGTGGAAATTCATCAAGAACAATTACAGGTGATACTTGTAATGAGGAG
GCCCAGAATCTGAGCACAATATATCTCAGGGAATATCAATCAAAAGTTAAGAGGCAGATA
TTTTCAGACTATCAGTCAGAGGTTGACATATATAACAGAATTCGGGATGAATTATGA
>elt8_eltB_EU113247
ATGAATAAAGTAAAATTTTATGTTTTATTTACGGCGTTACTATCCTCTCTATGTGCACAC
GGAGCTCCTCAGTCTATTACAGAACTATGTTCGGAATATCACAACACACAAATATATACG
ATAAATGACAAGATACTATCATATACGGAATCGATGGCAGGCAAAAGAGAAATGGTTATC
ATTACATTTAAGAGCGGCGCAACATTTCAGGTCGAAGTCCCGGGCAGTCAACATATAGAC
TCCCAAAAAAAAGCCATTGAAAGGATGAAGGACACATTAAGAATCACATATCTGACCGAG
ACCAAAATTGATAAATTATGTGTATGGAATAATAAAACCCCCAATTCAATTGCGGCAATC
AGTATGGAAAACTAG
>elt9_eltA_EU113248
ATGAAAAATATAACTTTCATTTTTTTTATTTTATTAGCATCGCCATTATATGCAAATGGC
GACAAATTATACCGTGCTGACTCTAGACCCCCATATGAAATAAAACGTTCCGGAGGTCTT
ATGCCCAGAGGGCATAATGAGTACTTCGATAGAGGAACTCAAATGAATATTAATCTTTAT

GATCACGCGAGAGGAACACAAACCGGCTTTGTCAGATATGATGACGGATATGTTTCCACT
TCTCTTAGTTTGAGAAGTGCTCACTTAGCAGGACAGTCTATATTATCAGGATATTCCACT
TACTATATATATGTTATAGCGACAGCACCAAATATGTTTAATGTTAATGATGTATTAGGC
GTATACAGCCCTCACCCATATGAACAGGAGGTTTCTGCGTTAGGTGGAATACCATATTCT
CAGATATATGGATGGTATCGTGTTAATTTTGGTGTGATTGATGAACGATTACATCGTAAC
AGGGAATATAGAGACCGGTATTACAGAAATCTGAATATAGCTCCGGCAGAGGATGGTTAC
AGATTAGCAGGTTTCCCACCGGATCACCAAGCTTGGAGAGAAGAACCCTGGATTCATCAT
GCACCACAAGGTTGTGGAAATTCATCAAGAACAATTACAGGTGATACTTGTAATGAGGAG
ACCCAGAATCTGAGCACAATATATCTCAGGAAATATCAATCAAAAGTTAAGAGGCAGATA
TTTTCAGACTATCAGTCAGAGGTTGACATATATAACAGAATTCGGGATGAATTATGA
>elt9_eltB_EU113248
ATGAATAAAGTAAAATGTTATGTTTTATTTACGGCGTTACTATCCTCTCTATGTGCACAC
GGAGCTCCCCAGTCTATTACAGAACTATGTTCGGAATATCGCAACACACAAATATATACG
ATAAATGACAAGATACTATCATATACGGAATCGATGGCAGGCAAAAGAGAAATGGTTATC
ATTACATTTAAGAGCGGCGCAACATTTCAGGTCGAAGTCCCGGGCAGTCAACATATAGAC
TCCCAAAAAAAAGCCATTGAAAGGATGAAGGACACATTAAGAATCACATATCTGACCGAG
ACCAAAATTGATAAATTATGTGTATGGAATAATAAAACCCCCAATTCAATTGCGGCAATC
AGTATGGAAAACTAG
>H10407_STa2
TTAATAGCACCCGGTACAAGCAGGATTACAACACAATTCACAGCAGTAATTGCTACTATT
CATGCTTTCAGGACTACTTTCATTATTTTTTTTACAATGTTACATTTTTTCGATTCTAG
TGTAATTTTTTCTTTTGAAGACCCTGCTGGTTTAGCATCCTGAGCGAAAGGTGAAAAAGA
TAATACAGAAAGAAAAATAAATAATATTGATTTTTTCAT
>ST1a_BCE008_MS-13
ATGAAAAAGCTAATGTTGGCAATTTTTATTTCTGTATTATCTTTCCCCTCTTTTAGTCAG
TCAACTGAATCACTTGACTCTTCAAAAGAGAAAATTACATTAGAGACTAAAAAGTGTGAT
GTTGTAAAAAACAACAGTAGAAAAAAATCAAGAAAATATAGAACAACACATTTTACTGCT
GTGAACTCTGTTGTAATCCTGCCTGTGCTGGATGTTATTAAAAAGCATAGAGGGAATCTT
TATTTTGATTCCCTCTATATTATATGTATTGGTGCTGAGAAAATCATAATAAAAAACAAC
GCTTTTATATTATATGTAAGTGCCTCATCTGAACCGTCTGGTCTGTTTCCTCCGGCTCCA
CAAAAATAA
>STa1_2.3916
ATGTTGGCAATTTTTATTTCTGTATTATCTTTCCCCTCTTTTAGTCAGTCAACTGAATCA
CTTGACTCTTCAAAAGAGAAAATTACATTAGAGACTAAAAAGTGTGATGTTGTAAAAAAC
AACAGTGAAAAAAAAATCAGAAAATATGAACAACACATTTTACTGCTGTGAACTTTGTTGT
AATCCTGCCTGTGCTGGATGTTATTAA
>STa1_pT5031
ATGAAAAAGCTAATGTTGGCAATTTTTATTTCTGTATTATCTTTCCCCTCTTTTAGTCAG
TCAACTGAATCACTTGACTCTTCAAAAGAGAAAATTACATTAGAGACTAAAAAGTGTGAT
GTTGTAAAAAACAACAGTGAAAAAAAAATCAGAAAATATGAACAACACATTTTACTGCTGT
GAACTTTGTTGTAATCCTGCCTGTGCTCCATGTTATTAA
>STa2_TW11681
ATGAAAAAATCAATATTATTTATTTTTCTTTCTGTATTATCTTTTTCACCTTTCGCTCAG
GATGCTAAACCAGCAGGGTCTTCAAAAGAAAAAATTACACTAGAATCGAAAAAATGTAAC
ATTGTAAAAAAAAAATAATGAAAGTAGTCCTGAAAGCATGAATAGTAGCAATTACTGCTGT
GAATTGTGTTGTAATCCTGCTTGTACCGGGTGTTATTAA
>STa3_153837-2
ATGAAGAAATCAATATTATTTATTTTTCTTTCTGTATTGTCTTTTTCACCTTTCCCTCAG
GATGCTAAACCAGTAGAGTCTTCAAAAGAAAAAATCACACTAGAATCAAAAAAATGTAAC
ATTGCAAAAAAAAAGTAATAAAAGTGGTCCTGAAAGCATGAATAGTAGCAATTACTGCTGT
GAATTGTGTTGTAATCCTGCTTGTACCGGGTGCTATTAA
>STa4_1.2741
ATGAAAAAGCTAATGTTGGCAATTTTTATTTCTGTATTATCTTTCCCCTCTTTTAGCCAG
AAAGCTGAATCAGTTGACTCTTCAAAAGAGAAAATTACATTAGACACTAAAAAGTGTAAT
GTTGTAAAAAACAACAGTAAAAAAAAAATCAGAAAATATGAATAACACATTTTACTGCTGT
GAACTTTGTTGTAATCCAGCCTGTGCTGGATGTTATTAA
>STa5_1.2741

ATGAAAAAGTTAATACTGGCAATTTTTATTTCTGTATTATCTTTTTCCTCTTTTAGCCAG
AAAACTGAATTGGTTGACTCTTCAAAAGAGGAAAGTACATCAGACACTAAAAAGTGTACC
GTTGTAAAAAACTATAATAAAAATAAATCAGCAGGCATCAATAACACGTTTTACTGCTGT
GAACTTTGTTGTAATCCTGCCTGTGCTGGATGTTATTAA
>STa5_FE95160
ATGAAAAAGTTAATACTGGCAATTTTTATTTCTGTATTATCTTTTTCCTCTTTTAGCCAG
AAAACTGAATTGGTTGACTCTTCAAAAGAGGAAAATACATCAGACACTAAAAAGTGTACC
GTTGTAAAAAACTATAATAAAAATAAATCAGCAGGCATCAATAACACGTTTTACTGCTGT
GAACTTTGTTGTAATTCTGCCTGTGCTGGATGTTATTAA
>STa6_UMEA_3176-1
ATGAAAAAACTAATGTTGGCAATTTTTATTTCTGTATTATCTTTCTCTTCTTTTAGTCAG
AAAACTGAATCAGTTGACTCTTCAAAAGAGAAAATAACATTAGACACTAAAAAGTGTAAT
GTTGTAAAAGGCAACAGTGAAAAAAAAATCAGAAAATATGAACAACACATTTTACTGCTGT
GAACTTTGTTGTAATCCCGCCTGTGCTGGATGTTATTGA
>STb1_2.3916
ATGAAAAAGAATATCGCATTTCTTCTTGCATCTATGTTCGTTTTTTCTATTGCTACAAAT
GCCTATGCATCTACACAATCAAATAAAAAAGATCTGTGTGAAAATTATAGACTAATAGCC
AAGGAAAGTTGTAAAAAAGGTTTTTTAGGGGTTAGAGATGGTACTGCTGGAGCATGCTTT
GGCGCCCAAATAATGGTTGCAGCAAAAGGATGCTAA
>STb1_EC2173
ATGAAAAAGAATATCGCATTTCTTCTTGCATCTATGTTCGTTTTTTCTATTGCTACAAAT
GCCTATGCATCTACACAATCAAATAAAAAAGATCTGTGTGAAAATTATAGACAAATAGCC
AAGGAAAGTTGTAAAATAGGTTTTTTAGGGGTTAGAGATGGTACTGCTGGAGCATGCTTT
GGCGCCCAAATAATGGTTGCAGCAAAAGGATGCTAA
>stx1a_stx1A_O157EDL933
ATGAAAATAATTATTTTTAGAGTGCTAACTTTTTTCTTTGTTATCTTTTCAGTTAATGTG
GTGGCGAAGGAATTTACCTTAGACTTCTCGACTGCAAAGACGTATGTAGATTCGCTGAAT
GTCATTCGCTCTGCAATAGGTACTCCATTACAGACTATTTCATCAGGAGGTACGTCTTTA
CTGATGATTGATAGTGGCTCAGGGGATAATTTGTTTGCAGTTGATGTCAGAGGGATAGAT
CCAGAGGAAGGGCGGTTTAATAATCTACGGCTTATTGTTGAACGAAATAATTTATATGTG
ACAGGATTTGTTAACAGGACAAATAATGTTTTTTATCGCTTTGCTGATTTTTCACATGTT
ACCTTTCCAGGTACAACAGCGGTTACATTGTCTGGTGACAGTAGCTATACCACGTTACAG
CGTGTTGCAGGGATCAGTCGTACGGGGATGCAGATAAATCGCCATTCGTTGACTACTTCT
TATCTGGATTTAATGTCGCATAGTGGAACCTCACTGACGCAGTCTGTGGCAAGAGCGATG
TTACGGTTTGTTACTGTGACAGCTGAAGCTTTACGTTTTCGGCAAATACAGAGGGGATTT
CGTACAACACTGGATGATCTCAGTGGGCGTTCTTATGTAATGACTGCTGAAGATGTTGAT
CTTACATTGAACTGGGGAAGGTTGAGTAGCGTCCTGCCTGACTATCATGGACAAGACTCT
GTTCGTGTAGGAAGAATTTCTTTTGGAAGCATTAATGCAATTCTGGGAAGCGTGGCATTA
ATACTGAATTGTCATCATCATGCATCGCGAGTTGCCAGAATGGCATCTGATGAGTTTCCT
TCTATGTGTCCGGCAGATGGAAGAGTCCGTGGGATTACGCACAATAAAATATTGTGGGAT
TCATCCACTCTGGGGGCAATTCTGATGCGCAGAACTATTAGCAGTTGA
>stx1a_stx1B_O157EDL933
ATGAAAAAAACATTATTAATAGCTGCATCGCTTTCATTTTTTTCAGCAAGTGCGCTGGCG
ACGCCTGATTGTGTAACTGGAAAGGTGGAGTATACAAAATATAATGATGACGATACCTTT
ACAGTTAAAGTGGGTGATAAAGAATTATTTACCAACAGATGGAATCTTCAGTCTCTTCTT
CTCAGTGCGCAAATTACGGGGATGACTGTAACCATTAAAACTAATGCCTGTCATAATGGA
GGGGGATTCAGCGAAGTTATTTTTCGTTGA
>stx1c_stx1A_O174-DG131-3
ATGAAAATAATAATTTTTAGAGTGCTAACTTTTTTCTTTGTTATCTTTTCTGTTAATGTG
GTTGCGAAGGAATTTACCTTAGATTTCTCGACAGCAAAGACGTATGTAGATTCGCTGAAT
GTCATTCGCTCTGCAATAGGTACTCCATTACAGACTATTTCATCAGGAGGTACGTCTTTA
CTGATGATTGATAGTGGCACAGGGGATAATTTGTTTGCAGTTGATGTCAGAGGGATAGAT
CCAGAGGAAGGGCGGTTTAATAATCTACGGCTTATTGTTGAACGAAATAATTTATATGTG
ACAGGATTTGTTAACAGGACAAATAATGTTTTTTATCGCTTTGCTGATTTTTCACATGTT
ACCTTTCCTGGTACAACTGCGGTTACATTGTCTGGTGACAGTAGCTATACCACGTTACAG
CGTGTTGCGGGGATCAGTCGTACGGGGATGCAGATAAATCGCCATTCGTTGACTACTTCT
TATCTGGATTTAATGTCGCATAGCGGAACCTCACTGACGCAGTCTGTGGCAAGAGCGATG

TTACGGTTTGTTACTGTGACAGCTGAAGCTTTACGTTTTCGGCAAATTCAGAGGGGATTT
CGTACAACACTTGATGATCTCAGTGGGCGTTCTTATGTAATGACTGCTGAAGATGTTGAT
CTTACGTTGAACTGGGGAAGGTTGAGTAGTGTCCTGCCTGACTATCATGGACAAGACTCT
GTTCGTGTTGGAAGAATTTCTTTTGGAAGTGTTAATGCAATTCTGGGTAGCGTGGCATTA
ATACTGAATTGTCATCATCATGCATCGCGAGTTGCCAGAATTGTACCTAATGAGTTTCCT
TCTATGTGCCCGGTAGATGGAAGAGTGCGTGGGATTACGCACAATAAAATATTGTGGGAC
TCATCCACTCTGGGGGCAATTTTGATACGCAGGGCTATTAGCAGTTGA
>stx1c_stx1B_O174-DG131-3
ATGAAAAAAATATTATTAATAGCTGCATCACTTTCATTTTTTTCAGCAAGTGTGCTGGCT
GCGCCAGATTGTGTAACTGGGAAGGTGGAGTATACAAAATATAATGATGACGATACCTTT
ACAGTTAAAGTGGGAGATAAAGAATTATTTACTAACAGATGGAATCTTCAGTCTCTTCTT
CTCAGTGCACAAATTACGGGGATGACGGTAACCATTAAAACTAATGCCTGTCATAATGGA
GGGGGATTCAGCGAGGTTATTTTCCGTTGA
>stx1d_stx1A_O8-MHI813
ATGAAAATAATGATTTTTAGGGCGCTAACATTTTTCTTCGTTATCTTTTCAGTTAATGCG
ATTGCTAAGGAGTTTACCTTAGATTTCTCAACAGCAAAGAAGTATGTTGATTCGCTGAAT
GTCATTCGCTCTGCAATAGGTACGCCATTACAGACTATTTCATCGGGAGGTACATCTTTA
CTGATGATTGATAGTGGCACAGGCGATAATTTATTTGCAGTCGATATCATGGGGTTAGAA
CCAGAGGAAGAGCGGTTTAACAATCTACGACTGATTGTTGAACGAAATAATTTATATGTG
ACAGGATTTGTTAACAGGACAAACAATGTTTTTTATCGCTTTGCTGATTTTTCACACGTT
ACCTTTCCTGGTACAAGAGCGGTTACATTGTCTGGTGACAGTAGCTATACCACGTTACAG
CGTGTTGCGGGGATCAGTCGTACGGGGATGCAGATAAATCGCCATTCGCTGACTACTTCT
TATCTGGATTTGATGTCGTATAGCGGAACCTCACTGACGCAGTCTGTAGCAAGAGCAATG
TTACGGTTTGTTACTGTGACAGCTGAAGCTTTGCGTTTTCGCCAAATCCAGAGGGGATTT
CGTACAACACTTGATGATCTCAGTGGACGTTCTTATGTAATGACTGCTGAAGATGTTGAT
CTTACATTGAATTGGGGAAGACTGAGTAGTATTCTGCCCGACTATCATGGACAAGACTCC
GTTCGTGTTGGAAGAATTTCTTTTGGGAGTATTAATGCAATTCTGGGAAGCGTGGCATTA
ATATTAAATTGCCATCATCATGCATCACGAGTTGCCAGAATGACACCTGATGAGTTTCCT
TCTATGTGCCCGACAGATGGAAGTGGGCGTGGGATTACTCACAATAAAATATTGTGGGAC
TCATCTACACTGGGGGCAATTTTGATTCGCAGAACTATCAGTAGTTGA
>stx1d_stx1B_O8-MHI813
ATGAAAAAAGTATTATTAATAGCTGTTTCACTTTCATTTCTTTCAGCAAGTGTTCTGGCA
GCGCCAGATTGTGTAACCGGAAAGGTGGAGTATACAAAATATAATGATGACGATACTTTT
ACAGTTAAAGTGGCAGATAAAGAATTATTTACTAACAGATGGAATCTTCAGTCTCTTCTT
CTAAGTGCACAAATTACGGGGATGACGGTAACTATTAAGACCACTGCTTGTCATAATGGA
GGGGGATTCAGCGAGGTTATTTTTCGTTGA
>stx2a_stx2A_O157EDL933
ATGAAGTGTATATTATTTAAATGGGTACTGTGCCTGTTACTGGGTTTTTCTTCGGTATCC
TATTCCCGGGAGTTTACGATAGACTTTTCGACCCAACAAAGTTATGTCTCTTCGTTAAAT
AGTATACGGACAGAGATATCGACCCCTCTTGAACATATATCTCAGGGGACCACATCGGTG
TCTGTTATTAACCACACCCCACCGGGCAGTTATTTTGCTGTGGATATACGAGGGCTTGAT
GTCTATCAGGCGCGTTTTGACCATCTTCGTCTGATTATTGAGCAAAATAATTTATATGTG
GCCGGGTTCGTTAATACGGCAACAAATACTTTCTACCGTTTTTCAGATTTTACACATATA
TCAGTGCCCGGTGTGACAACGGTTTCCATGACAACGGACAGCAGTTATACCACTCTGCAA
CGTGTCGCAGCGCTGGAACGTTCCGGAATGCAAATCAGTCGTCACTCACTGGTTTCATCA
TATCTGGCGTTAATGGAGTTCAGTGGTAATACAATGACCAGAGATGCATCCAGAGCAGTT
CTGCGTTTTGTCACTGTCACAGCAGAAGCCTTACGCTTCAGGCAGATACAGAGAGAATTT
CGTCAGGCACTGTCTGAAACTGCTCCTGTGTATACGATGACGCCGGGAGACGTGGACCTC
ACTCTGAACTGGGGGCGAATCAGCAATGTGCTTCCGGAGTATCGGGGAGAGGATGGTGTC
AGAGTGGGGAGAATATCCTTTAATAATATATCAGCGATACTGGGGACTGTGGCCGTTATA
CTGAATTGCCATCATCAGGGGGCGCGTTCTGTTCGCGCCGTGAATGAAGAGAGTCAACCA
GAATGTCAGATAACTGGCGACAGGCCTGTTATAAAAATAAACAATACATTATGGGAAAGT
AATACAGCTGCAGCGTTTCTGAACAGAAAGTCACAGTTTTTATATACAACGGGTAAATAA
>stx2a_stx2B_O157EDL933
ATGAAGAAGATGTTTATGGCGGTTTTATTTGCATTAGCTTCTGTTAATGCAATGGCGGCG
GATTGTGCTAAAGGTAAAATTGAGTTTTCCAAGTATAATGAGGATGACACATTTACAGTG
AAGGTTGACGGGAAAGAATACTGGACCAGTCGCTGGAATCTGCAACCGTTACTGCAAAGT

GCTCAGTTGACAGGAATGACTGTCACAATCAAATCCAGTACCTGTGAATCAGGCTCCGGA
TTTGCTGAAGTGCAGTTTAATAATGACTGA
>stx2b_stx2A_O118-EH250
ATGAAGTGTATATTGTTAAAATGGGTACTGTGCCTGTTACTGGGCTTTTCTTCGGTATCC
TATTCCCGGGAATTTACGATAGACTTTTCGACTCAACAAAGTTATGTCTCTTCGTTAAAT
TGTATACGGACAGAAATATCGACCCCACTTGAACATATATCTCAGGGGACCACATCGGTA
TCTGTTATTAACCACACCCCACCGGGCAGTTATTTTGCTGTGGATATACGAGGGCTTGAT
GTCTATCAGGCGCGTTTTGACCATCTTCGTCTGATTATTGAGCAAAATAATTTATATGTG
GCCGGATTCGTTAATACGGCAACAAATACTTTCTACAGATTTTCAGATTTTGCACATATA
TCAGTGCCCGGTGTGACAACTGTTTCCATGACAACGGACAGCAGTTATACCACTCTGCAA
CGTGTCGCAGCGCTGGAACGTTCCGGAATGCAAATCAGTCGTCACTCACTGGTTTCATCA
TATCTGGCGTTAATGGAGTTTAGTGGAAATGCCATGACCAGAGATGCATCCAGAGCAGTT
CTGCGTTTTGTCACTGTCACAGCAGAAGCCTTACGGTTCAGGCAAATACAGAGAGAATTT
CGTCTGGCACTGTCTGAAACTGCTCCTGTTTATACGATGACACCGGAAGAAGTGGACCTC
ACACTGAACTGGGGGAGAATCAGCAATGTGCTTCCGGAGTTTCGGGGAGAGGGTGGTGT
C
AGAGTGGGGCGAATATCCTTTAATAATATATCAGCGATACTGGGCACAGTGGCGGTTATA
CTGAATTGCCATCATCAGGGGGCACGTTCCGTTCGCTCCGTGAATGAAGAGATACAACCA
GAATGTCAGATAACTGGCGACAGGCCAGTTATAAGGCTAAACAATACTTTATGGGAAAGT
AATACCGCAGCTGCTTTTCTGAATCGCAGGGCTCACTCTTTAAATACATCCGGAGAATAA
>stx2b_stx2A_O174-031
ATGAAGTGTATATTATTTAAATGGGTACTGTGCCTGTTACTGGGTTTTTCTTCGGTATCC
TATTCCCGGGAGTTTATGATAGACTTTTCGACCCAACAAAGTTATGTCTCTTCGTTAAAT
AGTATACGGACAGAGATATCGACCCCTCTTGAACATATATCTCAGGGGACCACATCGGTG
TCTGTTATTAACCACACCCCACCGGGCAGTTATTTTGCTGTGGATATACGAGGGCTTGAT
GTCTATCAGGCGCGTTTTGACCATCTTCGTCTGATTATTGAGCAAAATAATTTATATGTG
GCTGGGTTCGTTAATACGGCAACAAATACTTTCTACCGTTTTTCAGATTTTACACATATA
TCAGTGCCCGGTGTGACAACGGTTTCCATGACAACGGACAGCAGTTATACCACTCTGCAA
CGTGTCGCAGCGCTGGAACGTTCCGGAATGCAAATCAGTCGTCACTCACTGGTTTCATCA
TATCTGGCGTTAATGGAGTTCAGTGGTAATACAATGACCAGAGATGCATCCAGAGCAGTT
CTGCGTTTTGTCACTGTCACAGCAGAAGCCTTACGCTTCAGGCAGATACAGAGAGAATTT
CGTCAGGCACTGTCTGAAACTGCTCCTGTGTATACGATGACACCGGAAGAAGTGGACCTC
ACACTGAACTGGGGGAGAATCAGCAATGTGCTTCCGGAGTTTCGGGGAGAGGGGGGTGT
C
AGAGTGGGGCGAATATCCTTTAATAATATATCAGCGATACTGGGCACAGTGGCGGTTATA
CTGAATTGCCATCATCAGGGGGCGCGTTCCGTTCGCGCCGTGAATGAAGAGATACAACCA
GAATGTCAGATAACTGGCGACAGGCCAGTTATAAGGATAAACAATACTTTATGGGAAAGT
AATACCGCAGCTGCTTTTCTGAATCGCAGGGCCCACTCTTTAAATACATCCGGAGAATAA
>stx2b_stx2B_O118-EH250
ATGAAGAAGATATTTGTAGCGGCTTTATTTGCTTTTGTTTCTGTTAATGCAATGGCAGCT
GATTGTGCAAAAGGTAAAATTGAGTTCTCTAAGTATAATGAGAATGATACATTCACAGTA
AAAGTGGCCGGGAAAGAGTACTGGACTAACCGCTGGAATCTGCAACCGCTACTGCAAAG
C
GCACAGTTAACAGGAATGACGGTAACAATCAAATCAAATACCTGTGCGTCAGGTTCAGGA
TTTGCTGAAGTGCAGTTTAATTAA
>stx2b_stx2B_O174-031
ATGAAGAAGATATTTGTAGCGGCTTTATTTGCTTTTGTTTCTGTTAATGCAATGGCAGCT
GATTGTCCAAAAGGTAAAATTGAGTTCTCTAAGTATAATGAGAATGATACATTCACAGTA
AAAGTGGCCGGGAAAGAGTACTGGACTAACCGCTGGAATCTGCAACCGCTACTGCAAAG
C
GCACAGTTAACAGGAATGACGGTAACAATCAAATCAAATACCTGTGCGTCAGGTTCAGGA
TTTGCTGAAGTGCAGTTTAATTAA
>stx2c_stx2A_O174-031
ATGAAGTGTATATTATTTAAATGGGTACTGTGCCTGTTACTGGGTTTTCCTTCGGTATCC
TATTCCCGGGAGTTTATGATAGACTTTTCGACCCAACAAAGTTATGTCTCTTCGTTAAAT
AGTATACGGACAGAGATATCGACCCCTCTTGAACATATATCTCAGGGGACCACATCGGTG
TCTGTTATTAACCACACCCCACCGGGCAGTTATTTTGCTGTGGATATACGAGGGCTTGAT

GTCTATCAGGCGCGTTTTGACCATCTTCGTCTGATTATTGAGCAAAATAATTTATATGTG
GCTGGGTTCGTTAATACGGCAACAAATACTTTCTACCGTTTTTCAGATTTTACACATATA
TCAGTGCCCGGTGTGACAACGGTTTCCATGACAACGGACAGCAGTTATACCACTCTGCAA
CGTGTCGCAGCGCTGGAACGTTCCGGAATGCAAATCAGTCGTCACTCACTGGTTTCATCA
TATCTGGCGTTAATGGAGTTCAGTGGTAATACAATGACCAGAGATGCATCCAGAGCAGTT
CTGCGTTTTGTCACTGTCACAGCAGAAGCCTTACGCTTCAGGCAGATACAGAGAGAATTT
CGTCAGGCACTGTCTGAAACTGCTCCTGTGTATACGATGACGCCGGGAGACGTGGACCTC
ACTCTGAACTGGGGGCGAATCAGCAATGTGCTTCCGGAGTATCGGGGAGAGGATGGTGTC
AGAGTGGGGAGAATATCCTTTAATAATATATCGGCGATACTGGGCACTGTGGCCGTTATA
CTGAATTGTCATCATCAGGGGGCGCGTTCTGTTCGCGCCGTGAATGAAGAGAGTCAACCA
GAATGTCAGATAACTGGCGACAGGCCCGTTATAAAAATAAACAATACATTATGGGAAAGT
AATACAGCTGCAGCGTTTCTGAACAGAAAGTCACAGTTTTATATACAACGGGTAAATAA
>stx2c-stx2B_O174-031
ATGAAGAAGATGTTTATGGCGGTTTTATTTGCATTAGTTTCTGTTAATGCAATGGCGGCG
GATTGCGCTAAAGGTAAAATTGAGTTTTCCAAGTATAATGAGAATGATACATTCACAGTA
AAAGTGGCCGGAAAAGAGTACTGGACCAGTCGCTGGAATCTGCAACCGTTACTGCAAAG
T
GCTCAGTTGACAGGAATGACTGTCACAATTAAATCCAGTACCTGTGAATCAGGCTCCGGA
TTTGCTGAAGTGCAGTTTAATAATGACTGA
>stx2d_stx2A_O73-C165-02
ATGAAGTGTATATTGTTTAAATGGGTACTGTGCCTGTTACTGGGCTTTTCTTCGGTATCC
TATTCCCGGGAATTTACGATAGACTTTTCGACTCAACAAAGTTATGTATCTTCGTTAAAT
AGTATACGGACAGAGATATCGACCCCTCTTGAACACATATCTCAGGGGACCACATCGGTG
TCTGTTATTAACCACACCCCACCGGGAAGTTATTTTTCTGTGGATATACGAGGGCTTGAT
GTCTATCAGGCGCGTTTTGACCATCTTCGTCTGATTATTGAGCAAAATAATTTATATGTG
GCCGGGTTCGTTAATACGGCAACAAATACTTTCTACAGATTTTCAGATTTTACACATATA
TCAGTGCCCGGTGTGACAACGGTTTCCATGACAACGGACAGCAGTTATACCACTCTGCAA
CGTGTCGCAGCGCTGGAACGTTCCGGAATGCAAATCAGTCGTCACTCACTGGTTTCATCA
TATCTGGCGTTAATGGAGTTCAGTGGTAATACAATGACCAGAGATGCATCCAGAGCAGTT
CTGCGTTTTGTCACTGTCACAGCAGAAGCCTTACGCTTCAGGCAGATACAGAGAGAATTT
CGTCAGGCACTGTCTGAAACTGCTCCTGTGTATACGATGACGCCGGGAGACGTGGACCTC
ACTCTGAACTGGGGGCGAATCAGCAATGTGCTTCCGGAGTATCAGGGAGAGGATGGTGTC
AGAGTGGGGAGAATATCCTTTAATAATATATCGGCGATACTGGGCACTGTGGCCGTTATA
CTGAATTGTCATCATCAGGGGGCGCGTTCTGTTCGCGCCGTGAATGAAGATAGTCAACCA
GAATGTCAGATAACTGGCGACAGGCCAGTTATAAAAATAAACAATACATTATGGGAAAGT
AATACAGCAGCAGCGTTTCTGAACAGAAAGTCACAGTCTTTATATACAACGGGTGAATAA
>stx2d_stx2B_O73-C165-02
ATGAAGAAGATGTTTATGGCGGTTTTATTTGCATTAGTTTCTGTTAATGCAATGGCGGCG
GATTGTGCTAAAGGTAAAATTGAGTTTTCCAAGTATAATGAGAACGATACATTCACAGTA
AAAGTGGCCGGGAAAGAGTACTGGACTAACCGCTGGAATCTGCAACCGCTACTGCAAAG
T
GCACAGTTAACAGGAATGACAGTCACAATCAAGTCCAGTACCTGTGCATCAGGCTCCGGA
TTTGCTGAAGTGCAGTTTAATAATGACTGA
>stx2e_stx2A_O139-S1191
ATGAAGTGTATATTGTTAAAGTGGATACTGTGTCTGTTACTGGGTTTTTCTTCGGTATCC
TATTCCCAGGAGTTTACGATAGACTTTTCGACTCAACAAAGTTATGTATCTTCGTTAAAT
AGTATACGGACAGCGATATCGACCCCTCTTGAACATATATCTCAGGGAGCTACATCGGTA
TCCGTTATTAATCATACACCACCAGGAAGTTATATTTCCGTAGGTATACGAGGGCTTGAT
GTTTATCAGGAGCGTTTTGACCATCTTCGTCTGATTATTGAACGAAATAATTTATATGTG
GCTGGATTTGTTAATACGACAACAAATACTTTCTACAGATTTTCAGATTTTGCACATATA
TCATTGCCCGGTGTGACAACTATTTCCATGACAACGGACAGCAGTTATACCACTCTGCAA
CGTGTCGCAGCGCTGGAACGTTCCGGAATGCAAATCAGTCGTCACTCACTGGTTTCATCA
TATCTGGCGTTAATGGAGTTCAGTGGTAATACAATGACCAGAGATGCATCAAGAGCAGTT
CTGCGTTTTGTCACTGTCACAGCAGAAGCCTTACGGTTCAGGCAAATACAGAGAGAATTT
CGTCTGGCACTGTCTGAAACTGCTCCTGTTTATACGATGACGCCGGAAGACGTGGACCTC
ACTCTGAACTGGGGGAGAATCAGCAATGTGCTTCCGGAGTATCGGGGAGAGGCTGGTGTC
AGAGTGGGGAGAATATCCTTTAATAATATATCAGCGATACTTGGTACTGTGGCCGTTATA

CTGAATTGCCATCATCAGGGCGCGCGTTCTGTTCGCGCCGTGAATGAAGAGAGTCAACCA
GAATGTCAGATAACTGGCGACAGGCCCGTTATAAAAATAAACAATACATTATGGGAAAGT
AATACAGCAGCAGCGTTTCTGAACAGAAAGTCACAGTCTTTATATACAACTGGTGAATGA
>stx2e_stx2B_O139-S1191
ATGAAGAAGATGTTTATAGCGGTTTTATTTGCATTGGTTTCTGTTAATGCAATGGCGGCG
GATTGTGCTAAAGGTAAAATTGAGTTTTCCAAGTATAATGAGGATAATACCTTTACTGTG
AAGGTGTCAGGAAGAGAATACTGGACGAACAGATGGAATTTGCAGCCATTGTTACAAAGT
GCTCAGCTGACAGGGATGACTGTAACAATCATATCTAATACCTGCAGTTCAGGCTCAGGC
TTTGCCCAGGTGAAGTTTAACTGA
>stx2f_stx2A_O128-T4-97
ATGCGACATATATTATTAAAGCTGGTGTTGTTTTTTTGTGTTTGCTTGTCTTCAGCATCT
TATGCAGATGAGTTTACTGTGGATTTCTCTTCGCAAAAGAGCTATGTTGATTCATTGAAT
AGTATAAGGTCGGCAATATCCACTCCACTTGGAAATATATCTCAGGGTGGTGTTTCTGTT
TCAGTAATTAATCATGTTCCAGGCGGAAACTATATATCATTGAATGTTAGAGGCCTTGAT
CCATATAGCGAGAGATTTAACCACCTCCGTTTAATAATGGAACGGAATAACTTATATGTT
GCAGGCTTTATTAATACTGAAACGAATACCTTTTACAGATTCTCCGATTTCTCACATATT
TCAGTGCCTGATGTGATAACTGTTTCCATGACGACGGACAGCAGTTATTCATCATTACAG
CGAATCGCAGATCTGGAACGTACAGGGATGCAGATTGGGCGTCATTCACTGGTTGGTTCA
TATCTGGATTTAATGGAGTTCAGAGGACGTTCCATGACCCGCGCATCATCCAGAGCTATG
CTGCGTTTTGTCACAGTGATAGCAGAAGCTCTGCGATTCAGACAAATACAGCGGGGATTC
CGACCGGCGCTGTCTGAGGCATCTCCGCTTTATACAATGACGGCTCAGGATGTTGACCTT
ACCCTGAACTGGGGAAGAATAAGTAATGTTCTTCCAGAGTACAGAGGAGAGGAAGGGGT
A
AGAATCGGTAGGATATCTTTTAATAGTCTTTCTGCGATTCTCGGAAGTGTTGCGGTCATC
CTTAATTGCCACTCAACCGGAAGTTATTCAGTTCGTTCCGTGAGCCAAAAACAGAAAACA
GAATGCCAGATTGTTGGAGACAGGGCGGCCATTAAAGTAAATAATGTTTTGTGGGAAGCG
AATACAATCGCTGCTTTATTAAATCGCAAGCCTCAGGATCTTACTGAACCAAACCAATAA
>stx2f_stx2B_O128-T4-97
ATGAAGAAGATGATTATTGCAGTTTTATTCGGTCTCTTTTCTGCTAATTCCATGGCGGCG
GATTGTGCTGTAGGAAAAATTGAGTTTTCCAAGTATAATGAGGATGATACCTTTACTGTG
AAGGTGTCAGGAAGAGAATACTGGACGAACAGATGGAATTTGCAGCCATTGTTACAAAGT
GCTCAGCTGACAGGGATGACTGTAACAATCATATCTAATACCTGCAGTTCAGGCTCAGGC
TTTGCCCAGGTGAAGTTTAACTGA
>stx2g_stx2A_AJ966783
ATGAAGTGTATATTATTTAAATGGGTACTGTGCCTGTTACTGGGCTTTTCTTCGGTATCC
TATTCCCGGGAATTTACGATAGACTTTTCGACTCAACAAAGTTATGTATCTTCGTTAAAT
AGTATACGGACAGAAATATCGACCCCTCTTGAGCATATATCTCAGGGGGCTACATCGGTA
TCTGTTATTAATCATACTCCACCGGGTAGTTATATTTCTGTGGATATCCGAGGGCTTGAT
ATCTATGAGGCGCGTTTTGACCATCTTCGTCTGATTATTGAGCAAAATAATTTATATGTG
GCCGGGTTCGTTAATACGGCCACAAATACTTTCTACCGTTTTTCAGATTTTACACATATA
TCAGTGCCCGGTGTGACAACTGTTTCCATGACAACGGACAGCAGTTATACCACGCTGCAA
CGTGTCGCAGCGCTGGAACGTTCCGGAATGCAAATCAGTCGTCACTCACTGGTTTCATCA
TATCTGGCGTTAATGGAGTTCAGTGGTAATACAATGACCAGAGATGCATCCAGAGCAGTT
CTGCGTTTTGTCACTGTTACAGCAGAAGCCTTACGGTTCAGGCAAATACAGAGAGAATTT
CGTCTGGCACTGTCTGAAACTGCTCCTGTTTATACGATGACGCCGGAAGACGTGGACCTC
ACTCTGAACTGGGGGAGAATCAGCAATGTGCTTCCGGAGTATCGGGGAGAGGATAGTGTC
AGAGTGGGGAGAATATCCTTTAATAATATATCAGCAATACTGGGTACTGTAGCGGTTATT
CTGAATTGCCATCATCAGGGGACGCGTTCGGTTCGCTACGTGAATGAAGAGATGCAACCA
GAATGTCAGATAAGTGGCGACAGGCCAGTTATAAAAATAAACAATACATTATGGGAAAGT
AATACAGCAGCAGCCTTTCTGAACAGAAAGTCTCAGTCATTATATACAACGGGTGAATGA
>stx2g_stx2B_AJ966783
ATGAAGAAGATGTTTATGGCGGTTTTATTTGCATTGGTTTCTGTTAATGCAATGGCGGCG
GATTGTGCTAAAGGTAAAATTGAGTTTTCCAAATATAATGGGGATAACACATTTACTGTA
AAGGTTGACGGGAAAGAATACTGGACTAACCGGTGGAATTTGCAGCCGTTGTTACAAAGT
GCACAGTTAACAGGAATGACCGTAACAATCAAATCCAATACCTGTGAATCAGGCTCTGGA
TTTGCTGAAGTGCAGTTTAATAATGACTGA
>stx2h_stx2A_NZ_CP076706

ATGAAGTGTATATTGTTAAAATGGATGCTGTGCCTGTTACTGGGCTTTTCTTCGGTATCC
TATTCCCGGGAGTTTACGATAGACTTTTCGACTCAACAAAGTTATGTCTCTTCGTTAAAT
AGTATACGGACAGCGATATCGACCCCTCTTGAACATATATCTCAGGGAACCACATCGGTA
TCTGTTATTAATCATACGTCACCAGGAAGTTATATTACTGTCGATATACGAGGGCTTGAT
GTCTATCAGGCGCGCTTTGACCATCTTCGCCTGATTGTTGAGCAAAATAATTTATATGTG
GCAGGGTTCGTTAATACGGCAACAAATACTTTCTACAGATTTTCAGATTTTGCACATATA
TCAGTGCCCGGTGTGACAACTGTTTCCATGACAACGGACAGCAGTTATACCACTCTGCAA
CGTGTCGCAGCGCTGGAACGTTCCGGAATGCAAATCAGTCGTCACTCACTGGTTTCATCA
TATCTGGCGTTAATGGAGTTCAGTGGTAATGCCATGACCAGAGAGGCTTCCAGGGCTGTC
CTGCGTTTTGTCACTGTTACAGCCGAAGCCTTACGGTTCAGGCAAATACAGAGAGAATTT
CGTCAGGCGCTATCTGAAGCAGCTCCTGTTTATACGATGACACCGGAAGACGTGGACCTT
ACTCTGAACTGGGGGAGAATCAGCAATGTGCTTCCGGAGTTTCGGGGAGAGGATGGTGTC
AGAGTGGGGAGAATATCCTTTAATAATATATCAGCAATACTGGGTACTGTGGCGGTTATA
TTGAATTGCCATCATCAGGGGGCACGTTCTGTTCGCTCCGTTAATGAAGAGAAACAGCCG
GAATGTCAGATAACTGGCGACAGGCCAGTTATAAAAATAAATAATATTTATGGGAAAGT
AATACAGCAGCAGCTTTCTGAACCGAAGATCTCATTCTTTATATGCAACTGGTGAATGA
>stx2h_stx2B_NZ_CP076706
ATGAAGAAGATGTTTATGGTGGCTTTATTTGCGTTAGTTTCTGTTAATGCAATGGCAGCG
GAATGCGCTAAAGGTAAAATTGAGTTCTCTAAATATAATGGGGATAATACATTCACAGTA
AAAGTGGCCGGGAAAGAATACTGGACTAACCGCTGGAATCTGCAACCGTTACTGCAAAGT
GCACAGTTGACAGGAATGACTGTAACAATCAAATCCATTACCTGTGATTCAGGCTCCGGA
TTTGCTGAAGTGCAGTTTAATTAG
E. coli virulence genes
>aafA__EAEC__aggregative_adherence_fimbriae_AAFs__O44:H18_042
ATGAAAAAAATCAGAATGTTTGCGATTGCTACTTTATTATCAAGTGGAGCCGCTATTAAT
GCAACTGCGGTAGCAAAAACTGCGACCAGTACTATCACTGTAGTGAATTTTTGTGATATA
ACGATAACACCGGCTACAAATCGTGATGTCAACGTTGACAGGAGCGCAAATATCGACCTG
AGTTTTACTATTAGACAACCGCAACGCTGCGCTGATGCTGGTATGCGAATAAAAGCTTGG
GGGGAAGCCAATCACGGTCAATTACTGATAAAACCTCAAGGAGGAAATAAATCAGCAGGA
TTCACTCTGGCCTCTCCTAGGTTTTCTTACATTCCGAATAATCCAGCAAACATTATGAAT
GGATTTGTTCTTACGAATCCTGGTGTTTATCAATTAGGAATGCAGGGCTCAATTACACCG
GCTATCCCGCTACGACCAGGCCTATATGAAGTAGTATTAAATGCTGAGCTTGTGACAAAT
TAA
>aap__EAEC__dispersin__O44:H18_042
ATGAAAAAAATTAAGTTTGTTATCTTTTCTGGCATCTTGGGTATCAGCCTGAATGCTTTT
GCGGGTGGTAGCGGTTGGAACGCAGATAATGTGGACCCGTCCCAATGTATAAAACAGTCT
GGAGTACAGTATACTTATAACAGCGGTGTCTCAGTATGTATGCAAGGCCTTAATGAAGGG
AAAGTAAGGGGGGTGTCTGTCTCTGGGGTATTTTATTATAATGATGGCACAACAAGCAAC
TTCAAAGGGGTTGTTACCCCCTCCACACCTGTAAATACGAACCAAGACATTAACAAGACA
AATAAGGTTGGAGTCCAAAAATATCGTGCTCTAACCGAATGGGTTAAATAA
>aatA__EAEC__dspersin__O44:H18_042
GTGAGACACATATTATACTCATTTCTTGCAATAAATGCTTATCTGTTTTCGACACAGACT
CTGGCAAAAGACTGTATCATTGATAATTTCTTTCAGAAAAGCATCCAGTTTAATTCTTAT
TCTCTTGATATCGAAGAGTTGGATATTAATAAACATAACAATATAAAAACGATGTTACCA
GATATAAATATAGGGTTAGGGCAGTATATAAACAACAATCAGTGGTTCTCATCTATTACA
GACAGCAATTTTTATTTATCATTATCCTATAATCTTCTATCGGCTTATGAAGCAAAAATG
CAGAATGATAAATTGGATATTGCTAATTATTTAAAATATATTGAAATGCTTAGTGAGAGA
AACAACTATATAATTAATTTGTTCTCGGAAATCATTAACTATAAGATAAAAAAATCTCAC
CTGATGTTGATGCTCGAGAGATATAGAAAGCTTAATAAAGAATACGAAATTGCAAAGCGT
AAAATGTCAATTGGATTAATATCTGTTCTTGATGTAGAGATGAGATATAATATATTACAA
AAAATCAGGTTTGATATTGATGTACTTGAGGAGGAGGAAAGTTTACTGTCAGATAAAATC
TCGAGAGAATATCATGTTCCAGAGAGTGCAATCCCAGACATTACATATCATAAATTAAAA
GAGTGTAAAACAGCGGATTTCTATACATTATTAGCTGAAAACAAAAAACTCAAGATTAAG
GCTGCTGATATAGATAATGATATAAGAAAACTATCGGAGATCCCATCTTTTTATTTATCA
TTTGGATTAACACCTAAACAGGGAGGTGCATTGGGTAATATGAGTCTCAGAAAAATGGAT
TATAGTGCTAGTCTGGGTATCAGTTTTCCTTTGATGGGATTATTTAGTTCTTCAGAAAAT
CAAAAAGAAAAGATTATTTCTATATCTCGAACCAGAAATGAATTATTGAAAGAAAATATA

AAACTAGATCTGTTGGAAAAAGAGATTCGCCAGAAAATTGATAAATTAGAGAAAAATCTT
GCGATGATGAAAAATGAACTAGCTCTGAAAAAAAGGAAAATTGAGTATATAAATTATCGC
GTAAAGAATGGACAAGACGACGTTATCACTTATTTGTCTAGTGTAGAGAATTTACATGAA
ACAGAAAATGAATTTCAGAAAATTGGATATGAGATTGAATATTATAGTTTATATCATTAT
TTTCTTCTGCAGCACATTTCCAATACAGGGGAAATGTGA
>aggR__EAEC__regulation__55989
TCATTGGCTTTTAAAATAAGTCAAGAATTGTTTTGGTGTTATGCCAAAATGTTTTACAAA
AAGCCTAATGAAATATGATGTACTGGAAAATCCTATCATATTAGATATCTGTGATATCTG
ATATGAGTTATCAAGCAACAGCAATGCTGCTTTGCTCATTCTTGATTGCATAAGGATCTG
GTTAAAAGTAATATACTCTGACTCAAGCCTTTTCCTTATTGTTATCTCTGATACATTAAA
TTCATCTGCAATAATAGCTAGAGTCCATCTCTTTGATAAGTCCTTCTCGATTGTGTTTCT
GACCTTATCGGAAAAGAAGCTTACAGCCGATATATAAATTGACTCTATAATTTTCCTGTT
GTTTTCGATCTTTGATATAAGATATATCAAATCTGAAGTAATTCTTGAATCATCATTATG
ATCGATACTCTTGAATGTATCTAATACAGAATCGTCAGCATCAGCTACAATTATTCCTTT
TGACCAATTCGGACAACTGCAAGCATCTACTTTTGATATTCCGTATATTATCATCAGGGC
ATCCTTTAGGCGTCTTAATGTATCGCTGCTTAATCTGATTGCGATAAATGGATTTACTGT
TGATTTCTTCTTTTGCAATCTTACTGATATATTTATTCCTCTCTCAAGAAATATGAGTTC
ATTTCTAAGATATGTATTGCTTCCTTCTTTTGTGTATACATCGATTGTACAATTAGATGT
ATATAGTACAGTGTACTGATGTATTCTGATATTGTTAATTTTTATAATCTCTTTTTCGAT
ATTTTGTTTTAATTTCAT
>bfpA__STEC__bundle_forming_pilus__E2348
ATGGTTTCTAAAATCATGAATAAGAAATACGAAAAAGGTCTGTCTTTGATTGAATCTGCA
ATGGTGCTTGCGCTTGCTGCCACCGTTACCGCAGGTGTGATGTTTTACTACCAGTCTGCG
TCTGATTCCAATAAGTCGCAGAATGCTATTTCAGAAGTAATGAGCGCAACGTCTGCAATT
AATGGTCTGTATATTGGGCAGACCAGTTATAGTGGATTGGACTCAACGATTTTACTTAAC
ACATCTGCAATTCCGGATAATTACAAAGATACAACAAACAAAAAAATAACCAACCCATTT
GGGGGGGAATTAAATGTAGGTCCAGCAAACAATAACACCGCATTTGGTTACTATCTGACG
CTTACCAGGTTGGATAAAGCGGCATGTGTTAGTCTTGCAACCTTGAACTTAGGTACTTCA
GCGAAAGGCTACGGTGTTAATATCTCTGGCGAAAATAACATTACATCATTTGGTAATAGC
GCTGATCAGGCTGCTAAATCGACTGCTATTACTCCTGCTGAAGCGGCAACTGCATGTAAA
AATACTGATTCAACCAATAAAGTTACATATTTTATGAAGTAA
>cdt-IA__not_specified__cytolethal_distending_toxin_type_I_subunit_A__E6468/62
GTGGATAAAAAACTAATTGCATTTTTGTGCACACTTATAATTACTGGTTGCTCGAATGGG
ATCGGTGATTCACCTTCACCTCCGGGAAAAAATGTAGAATTGGTTGGAATCCCTGGACAA
GGTATTGCAGTGACTTCAAACGGTGCAACTCCAACACTTGGAGCCAACAACACTGAGTTT
CCTGAAGTTTCAATAATGAGCACTGGTGGGGCGCTGCTTACTATTTGGGCCAGACCTGTT
CGTAACTGGCTTTGGGGGTATACTCCTTTTGATTCAGTAAATTTTGGTGAGAATCGGAAC
TGGAAGGTTGTGGATGGGAAAGATGCCGGCACAGTGAAATTTGTTAATGTTGCCCAGGGG
ACTTGCATGGAGGCCTTTAAAAACGGGGTGATACATAATACCTGTGATGATAACTCGTTA
TCTCAGGAGTTTCAGTTACTGCCTTCTACTAATGGTAATGTGCTTATAAGAAGTAGTGCC
TTGCAGACGTGTATAAGAGCAGACTATTTAAGCAGAACTATATTGTCACCGTTTGCTTTT
ACAATCACCCTTGAGAAATGCCCTGGTGCAAAAGAAGAAACGCAAGAAATGCTATGGGC
A
ATAAGTCCACCTGTCAGAGCGGCAAAACCAAATCTGATTAAGCCAGAGTTAAGACCATTC
AGACCATTGCCAATTCCACCTCATGACAAACCTGATGGAATGGAGGGAGTATGA
>cdt-IIA__not_specified__cytolethal_distending_toxin_type_II_subunit_A__9142-88
ATGGCTAACAAACGTACACCTATTTTTATAGCTGGAATCTTGATCCCCATTTTATTAAAT
GGTTGCTCATCAGGAAAAAATAAAGCTTATCTTGACCCCAAAGTTTTCCCTCCTCAAGTG
GAAGGAGGACCAACCGTTCCTTCCCCCGATGAGCCCGGACTTCCATTGCCCGGGCCAGGA
CCGGCGCTGCCCACAAATGGCGCAATCCCTATCCCTGAACCAGGTACCGCACCCGCAGTA
TCTTTAATGAATATGGATGGCTCAGTTCTAACAATGTGGAGCCGCGGAGCTGGTTCATCG
TTATGGGCGTATTATATCGGCGACTCCAATTCATTTGGGGAACTACGTAATTGGCAGATT
ATGCCCGGAACCAGGCCAAATACGATACAGTTTCGCAATGTAGACGTTGGTACCTGTATG
ACAAGTTTCCCAGGATTTAAAGGGGGAGTACAACTTTCTACAGCACCTTGCAAGTTTGGA
CCGGAACGTTTCGATTTCCAGCCAATGGCAACACGCAATGGTAATTACCAGTTAAAATCT
TTATCTACAGGTTTATGCATCAGAGCGAATTTTTTAGGAAGAACACCATCATCTCCGTAC
GCAACGACATTAACAATGGAGCGTTGCCCATCAAGTGGAGAGAAAAACTTTGAATTCATG

TGGTCCATAAGCGAACCATTAAGGCCTGCTCTGGCCACTATTGCCAAGCCAGAAATACGC
CCATTTCCACCACAGCCAATAGAACCAGATGAGCATTCAACTGGAGGAGAACAATGA
>cdt-IIIA__not_specified__cytolethal_distending_toxin_type_III_subunit_A__S5
ATGACTAATAAATGCACATCTATTTTGATAGTAGGCATTCTTATTCCAATTCTATTAAAT
GGTTGTTCGTCAAGAAAAAATAGAGCTCATCTTGACCCCAAAGTTTTCCCTCCTCAAGTA
GAGGGAGGACCAACAATTCCTTCTCCAGATGAGCCTGGACTTCCATTACCCGGGGCTGGA
CCAGCACTTCCCACAAATGCACCAATCCCTATCCCGGTACCAGGTACAGCACCCGCAGTA
TCTTTGATGAACATGGATGGCTCAGTTCTAACAATGTGGAGCCGCGGAGCTGGTTCATCA
TTATGGGCTTATTATATCAGCGACTCCAATTCATTTGGAGAACTACGCAATTGGCAGATT
ATGCCCGGAACCAGACCAAATACGATACAGTTCCGCAATGTAGACGTTGGTACTTGTATG
ACAAGCTTCCCGGGGTTTAAAGGAGGTGTGCAACTATCTACTGCACCTTGCCAGTTTGGA
CCAGATCGCTTCGACTTCCAGCCGATGGTAACACGCAATGGTAATTACCAGTTAAAATCG
TTGTCTACAGGTTTATGCATCAGAGCTAATTTTTTAGAAAGAACACCATCATCTCCGTAC
GCAACAACATTAACAATGGAGCGTTGCCCATCAAGTGGAGAGAGAAACTTCGAATTTATG
TGGTCCATAAGCGAACCATTAAGGCCAGCTCTAGCCACTATTGTTAAGCCAGAAATACGC
CCATTTCCACCACTACCAATAGAACCAGATAGGCATTCAGCAGGAGGCGAACAATGA
>cdt-IVA__not_specified__cytolethal_distending_toxin_type_IV_subunit_A__APEC_O1
GTGGATAAAAAACTAATTGCATTTTTGTGCACACTTATAATTACTGGTTGCTCGGATGGG
ATCGGTGATTCACCTTCGCCACCGGGAAAGAATGTAGAATTAGTCGGAATTCCAGGGCAA
GGTGTCGCTGTTGCTTCAAATGGCACATCTCCAACATTTGGGAGCAACAGTACTGACTTT
CCTGATGTTTCAATAATGAGCACAGGGGGAGCGATGCTTACTGTTTGGGCCAGACCTGTC
CGTAACTGGCTTTGGGGATATACTCCGTTTGATTCAGTAAGTTTTGGAGAGAATCGGAAC
TGGAAGGTTGTGGATGGTAAAGATGCCGGTACAGTGAAATTTGTTAATGTTGCCCAGGGG
ACTTGCATGGAGGCCTTTAAAAACGGGGTGATACATAATACCTGTGATGATAATTCGTTA
TCTCAGGAGTTTCAGTTACTGCCTTCTACTAATGGTAATGTGCTTATAAGAAGTAGCGCC
TTGCAGACGTGTATAAGAGCAGACTATTTAAGCAGAACTATACTGTCACCGTTTGCTTTT
ACAATCACCCTTGAGAAGTGTCCTGGTGCAAAAGAAGAAACGCAAGAAATGCTATGGGC
A
ATAAGTCCACCTGTCAGAGCGGCAAAACCAAATCTGATTAAACCAGAATTAAGACCATTC
AGACCATTGCCAATTCCACCTCATGACAAACCTGATGGAATGGAGGGAGTATGA
>cdt-VA__not_specified__cytolethal_distending_toxin_type_V_subunit_A__q
ATGGCTAATAAATACACACCTATTTTTATAGCTGGTATCCTGCTCCCCATTTTATTAAAT
GGCTGCTCATCAGGAAAGAACAAAGCTCATCTTGACCCCAAAGTTTTCCCTCCTCAAGTA
GAGGGAGGACCAACAATTCCTTCTCCAGATGAGCCTGGACTTCCATTACCCGGGGCTGGA
CCTGCACTTCCCACAAATGCACCAATCCCTATCCCGGTACCAGGTACAGCACCCGCAGTA
TCTTTGATGAACATGGATGGCTCAGTTCTAACAATGTGGAGCCGCGGAGCTGGTTCATCA
TTATGGGCTTATTATATCAGCGACTCCAATTCATTTGGAGAACTACGCAATTGGCAGATT
ATGCCCGGAACCAGACCAAATACGATACAGTTCCGCAATGTAGACGTTGGTACTTGTATG
ACAAGCTTCCCGGGGTTTAAAGGAGGTGTGCAACTATCTACTGCACCTTGCCAGTTTGGA
CCAGATCGCTTCGACTTCCAGCCGATGGTAACACGCAATGGTAATTACCAGTTAAAATCG
TTGTCTACAGGTTTATGCATCAGAGCTAATTTTTTAGAAAGAACACCATCATCTCCGTAC
GCAACAACATTAACAATGGAGCGTTGCCCATCAAGTGGAGAGAGAAACTTCGAATTTATG
TGGTCCATAAGCGAACCATTAAGGCCAGCTCTAGCCACTATTGTTAAGCCAGAAATACGC
CCATTTCCACCACTACCAATAGAACCAGATAGGCATTCAGCAGGAGGCGAACAATGA
>cnf1__not_specified__toxin__UTI89
TCAAAATTTTTTTGAAAATACCTTCAATACCGATATTTCGGAGGCATCTGCATTCAGAGA
ATAACTCTCAGACAGAGACCTTACGACAACATTGCCGTCCACTCTCACCAGTACAGTCGC
CGATGTACCAAAGCCATGTTCAGGTATATTATCAAGGAAGTAAGGGAAAACAGAAACATT
ATCACGAATAATAGTGATTTGACTATCTGGTTTTTTTTCAGATGATGAGTAAGTTATTAA
TGAATCTTCAAAATTTTCCGACAGATAATCGACTAAGAAATCATTGCTCATTATTCCCTC
CACGCGAGGTATTGGTTCTTTTGTAAGTAGCTCAAGTACTTCAACTGCTTTTTTCACCCC
GGTAGTACTGGTAAATCCAGCCAAAGATTTTGTTGTACCAGTATGTACCTTATAAATATA
TCCTTCTTTTCGGGCAACAATTGTCGTACAACCACTTAGATTTCCGCTTGTAATGATTAC
TGGTTTACCCTCCTGGACTTCCTCAAGTGCTATTTTAATACCATTCGCCCCACGAGCAGA
ATTTGACACACGAATTATGCTTGTTTCATTAGTAAGATTATACTTCTTCCAATAAGTTGA
GCCGAGTGAAGATGAGTCTGATGGAATTTCCCCAGTATAGGTGTTCAATGTTGGAATACC
ATTTAGCCCCACTGTATTATCATAGAAATACTGATCATAGCCATGGTTATCAAAGCGATC

TTTTTTGATGTAGAAAAAGAGAGGTTGTTGCTCATCAGCATTAGCGGCTTCAAAATACGG
ATAGATTGCACGCTGACGTGTCGATGAAATACTTCCCCGTCCTTTAAGCACATCAATATT
ACCGCGACTTAATTTCTGAAAGTTAGATTTGGAGGTGCTTTCGATACTTTCTTTAAAATA
TCGTAGAGATAACAATTTACGTATGGTTGATATATCAGGAGGCGTTGATGGCTCAGGAAA
ATTAAATCTCCTGTCAACCACAGCCAGTACTTCAACCGGGGCTTCATCAGGAATTGCCCA
ATGATTAACATATGAAACATTATTATTGGTGATATATTTTACGTGCACTTTTATAACTAC
CAGAAAGTGTCGTCTGCTTGCTGCATCTGCACTGTTAACATTTGAGGTATATTCAGTAAA
ACCTAAGCTTGACTGTGGGCCCTGACCTAACAAAATTTCGTGTAATTCTTCTGTACTTCC
CCCAGCCGTATGATAAGAGGCAATATTCCATGCTTCTTCCTCAGTAGCTCCTCTCATCAA
AGTTACAAATCTATCCCCAAAGCTTCCAGAACCCTTAACATAATTAACAATATCCATTTC
TGACGTTGGTTGCGGTAATTTTGGGTTTGTATCAGGATATAAAAGTGAGCTGTCATCATT
AATAAATGATAACGGAGATCCGGTCACTGCAGAATAGGGTTCATCTTTAAAAAATACAGG
AATATTGAGACTTTCAAAATCAAATGGCTGCCCCCCTTTTAGACCACCACCTCGAGTCCA
GAGAATTTCATTATTATATTCGGTACGATAAATTCTTCGGGATAAAATCTCATATCCTGT
TTCAATGTCCACTTCATAGTAGATGCCGCTCAGAGAACTCCCTTTTACAGCGACAATTTG
ATTATCTTCATCACTAAGCTTTACAATATTGACATGCTGCCCGGAACTTTTAAGCTTAAC
GTCTAACAAATTAGTTGAGGATATACTATCTTCAGCAATTCCATATTCCTTCAAAAATAT
ATCGATATTTGTTTCATTCAGAGTCCTGCCCTCATTATTTATAACGCTGCTAAGTACCTC
CTGGTTTATTCCTGCCTTACTAATCAAGAGTGGAATTGCAAAAGACAGACCAAGCAATAC
TGCATTTGTCGCCAAACCAGGTATAGCAGAACGACGTTCTTCATAAGTATCACCATTAAT
CAGTTGATCAAAACTGATCCCCATACTGGAAGTAATAATCCCCAGTCCAAGCGGAACAGA
TACCTCCGGGACTACGATATCAAATATAGGGGTCATTGATAAAATCGTCTGGAGTATAGT
CAGAGCATAGTCTCGTTGAGCTTCACTGTTTGATTTTATAACGATATCACCATCACTGAA
ACTGCGTTTCTTAACAGAAATAGCTATAGCATCAAAAACATCTTTATTGCTAAGTGTCTT
ATTGGAATACAGAAAGTAAGACTCATTAAAATTACCATCATTTTCTATTGCATGTAGAAC
AGAATTTACTCCTGCATAGGAAACTCCATCTTGACGACTATATAATGAAAAATGTTGGGA
AAGTAATTGTTTGTTGTCACTATCCTTAATAAGTTCTTTAAGGGTAAGCCGCAAATCAGC
GATATTCTTCTTGAATAAAAAAGGTTTTTTTGCACCAGGAATGAAAAGCATTACCTTATC
ATTATTTTTGGCTACAAAAATATCAGATGCGTAATATCCATAAATATCAAAGAATAATAG
TGATATATTATCACTGCGATTTAAGGCGTTCATGGAGAAATTATATTCACGCTCATCAAG
CTCTCCATTTTTATAAAGATACAAAGCAGAGGAAATATAATAATTTTTTAGTAAAAGCTT
AAATTTATCATAGTATTTCGACCAGTAATCTGACAGAGTTTTTCCATAAACACCTGCAAA
ATCACACTTTTGTAAAAAATGCAGGTATTCCGACGGGAGCATTTTTTCTTCATTTTCTTC
GTTAAACACATCTGCACTCTTTGTTTTATAAATTCCTGTGCTTGAATTTGCATCATCAAA
AACATCAAAACGATAACCAATATTAATAAGAGCGGCTTGAGTGAGTGTCAGTACTTCTGT
TTTATATTTCCCAAGATGATCCCATCCAGTATAAGATCTGCTATTACTCCGGTTTTGAGA
AAAGCAGATGAAATAAGCATTATCAGGATCAATCCGACTAAACCACGGCAAGTCAGTTTT
AAAACAATTCTTAATGTAATCGCTGACAACTCTTTCAGGTGAAGGGAAATTTGATACCAA
CTCATTGTACTCAAGAAGATATTTTTGTTGCCATTGGTTACCCAT
>cnf2__not_specified__toxin__S5
ATGAACGTTCAATGGCAACAAAAATATCTTCTTGAGTACAATGAGTTGGTATCAAATTTC
CCTTCGCCAGAAAGAGTAGTTAGCGATTACATTAGGCGTTGTTTCAAAACAGACTTACCT
TGGTTTAGTCAAGTGGATCCTGATAACACTTATTTTATACGATTTTCTCAAAGTCGGAGT
AATAGTAGATCTTACACCGGATGGGATCATCTTGGAAAATATAAAACAGGAGTACTGACA
CTCACTCAAGCCGCACTTATTAATATTGGCTATCATTTTGATGTTTTTGATGATGCAAAT
GCAAGTGCGGGAATCTATAAAACAAGCAGTGCAGATATGTTTAACGAAAAAAATGAAGAG
AAAATGCTTCCTTCAGAATACCTTTATTTTTTGAAAGGGTGTGATTTTTCAGGTATTTAT
GGTCGATTTCTGTCAGATTATTGGTCGAAATACTATGATAAATTTAAGTTATTACTAAAA
AATTATTATATTTCCTCGGCTTTATATCTTTATAAAAACGGAGAAATTGATGAATATGAA
TATAATTTCTCTATTAGCGCATTGAATCGTAGAGATAATATATCATTATTCTTCTTTGAT
ATCTATGGGTATTACTCCTCTGATATGTTTGTGGCCAAGAACAATGAACGGGTAATGCTT
TTTATTCCTGGCGCAAAAAAACCTTTTTTATTCGAAAAAAATATTGCTGATTTACGAATT
AGTCTTAAAAATCTAATTAAAGAGAACGACAATAAACAGTTACTATCCCAGCATTTTTCG
TTATATAGTCGTCAAGATGGAATTACTTATGCGGGTGTAAATTCAGTACTCAATGCAATA
GAAAATGATGGCGTTTTTAATGAATCTTACTTTCTGTACTCCAATAAAAGGATTAATAAT
AAAGATGTTTTCGATGCCGTAGCATTTTCTGTCAAGAAACGTAGTTTTAGTGATGGTGAT
ATCGTTATAAAATCAAACAGTGAGGCTCAACGAGATTATGCACTGACCATACTTCAGACT

ATTTTATCAATGACTCCTATATTTGATGTCGCAATTCCGGAGGTGTCTGTTACGCTTGGA
CTGGGGATTATTGCTTCCAGTATGGGGATCAGTTTTGATCAGTTAATTAATGGTGATACT
TATGAAGAACGTCGTTCTGCTATCCCTGGTTTAGCCACAAATGCAGCATTGCTTGGGTTA
TCTTTTGCAATTCCGTTTTTGATTAGTAAGGCGGGCACAAACCAGAAGATACTTAGTAGA
TATACAAAGCATGAGATCAGGACTCTGAATGAAACAAATATCGATATGTTTTTGGAGGAA
TATGGAATTAATAAAAATAGTATATCAGAAACTAAAGTATTAGAAGTTGAGCTTAAAGGT
TCTGGGCAGCATGTCAATATTGTAAAGCTTAGTGATGAAGATAATAAAATCGTCGCTGTA
AAAGGGAATTCTCTGAGCGGCATCTACTATGAAGTTGACATTGAAACAGGATATGAAATT
TCATCCAGAAGAATTTATCGTACCGAATACAATGATAAAATTTTCTGGACTCGAGGTGGT
GGGCTTAAGGGGGGGCAATCGTTCGATTTTGAAAGTCTCAAACTCCCTATATTTTTCAAA
GATGAACCATATTCTGCAGTACCGGGGTCTTCGTTATCATTTATTAATGATGATAGCTCT
CTTTTATATCCTAATTCGACCCCAAAACTACCGCAACCAACGCCAGAAATGGAGATTGTT
AATTACGTTAAAAGAGCTGGAAACTTTGGGGAGAGACTTGTAACCTTGATGCGAGGAACA
ACTGAAGAAGAAGCGTGGAATATTGCCCGTTATCATACGGCAGGAGGAAGCACCGAAGA
A
TTGCATGAGATTTTGTTGGGGCAAGGCCCACAGTCGAGTTTAGGTTTTACTGAATATACT
TCAAATATTAACAGTGCAGATGCGGCAAGCAGACGACATTTTCTTGTAGTCATAAAAGTG
CAAGTGAAATATATAAACAATAATAACGTTTCGCATGTTAATCACTGGGCAATTCCTGAT
GAGGCTCCAGTAGAAGTACTGGCTGTGGTTGACAGGAGATTTAATTTCCCTGAGCCATCA
ACTCCACCTAATATATCAATTATACACAAGTTGTTATCTCTGAGATATTTTAAAGAAAAT
ATCGAAAGTACATCAAGGCTTAACTTACAGAAATTAAATCGTGGTAATATTGATATATTT
AAAGGGAGGGGGAGTATTTCATCAACACGTCAGCGTGCGATTTATCCGTATTTTGAATCT
GCTAATGCTGATGAGCAACAACCTGTCTTTTTCTACATCAAAAAAAACCGGTTTGATGAC
TTTGGCTATGATCAATATTTCTATAATAGTACAGTGGGGTTGAATGGTATTCCCACATTG
AACACCTATACTGGAGAAATTCTATCAGACGCATCCTCGCTCGGCTCAACTTATTGGAAA
AAGTATAATCTCACTAATGAAACAAGCATCATTCGTGTATCAAATTCTGCTCGAGGGGCA
AATGGTATAAAAATAGCACTTGAAGAAGTGCAGGAAGGTAAACCGGTAATCATTACAAGC
GGAAATTTGAGCGGTTGTACAACAATTGTTGCTCGAAAAGGAGGATACCTTTATAAGGTA
CATACAGGTACAACAATACCTTTAGCTGGTTTTACAAGTACAACAGGGGTAAAAAAAGCT
GTAGAAGTTTTTGAATTACTTACAAATAATCCAATGCCGCGCGTAGAGGGAGTAATGAAT
AATGATTTTTTGGTAAATTATCTGGCGGAAAGTTTTGATGAGTCTTTAATAACGTACTCA
TCATCTGAACAAAAAATAGGTAGTAAGATTACTATTTCTCGCGACAATGTTTCTACTTTT
CCTTACTTTCTTGATAACATACCAGAAAAAGGCTTTGGTACATCGGTGACTATATTGGTA
AGAGTAGATGGTAATGTTATCGTAAAATCCTTATCTGAGAGTTATTCTTTAAATGTAGAA
AACTCCAATATATCAGTATTGCATGTTTTTTCAAAAGATTTTTGA
>ealA__clade1__pertussis-like_toxin_catalytic_A_subunit__10290
GTGGATTCTCGCCCGCCAGAGGAAATATTTCGTGATGGGTTTAGATCCCATGGTTTTAAC
AGAAACTTACAGCAACATTTAAGAGGGGACTCATGTGCGGCAGGGAGTAGGGATAGTGCA
TTTATTGCTACCACAACCAGTTTAATTGAAACATATAACATAGCCAGACAATATTATTCA
AGTTCTGGATTTCACGGTAGGTTATATCGTTACCGTATCAGAGCAAATAATATTTTTTAT
CCTATTCAACCGTCAGTCAATTATCTAACCCAGCGCGGTATAACCTTCTCTGGATTTGAG
CGGATAATGATGAGGGAGCAGAACGAGATTGTCGCGGTGGAACATATACCTGGTGAGAAT
ATTGTTGAGGCTGTGGAGTTGACTTATGACAGATTCAACAGCCAAGTATCTGATGGACCA
GGAACCACCAATGCCAGGTATGTTCCTGGATCCACGTTTGTAAATCCAGGTGTTATACCT
CAATTAGTTGTACCAACTGTGTCAGTTAGAGAAAGAATCAATGCATTTGGAAGTCTGATC
AGTGCTTGTTTTGCTCTGAAGGGAGTCAGAAGAGATGGTTTAAATAAAAGAGCCACTTAT
TATGAACCCGAGTTTTATGATGCAAGAGGTGTGTTAAAAGAAATAATAAAATAA
>eatA__clade1__serine_protease__10290
TTGCTGACGTCCGTACTTTTCTCTGCAGGAGGCTTTGCGGGAACAGTTAATAATGAACTT
GGCTATCAGTTATTTCGTGATTTTGCTGAGAATAAAGGTATGTTCCGCCCGGGGGCAACG
AATATCGCCATTTATAATAAGCAGGGAGAGCTTGTAGGTACGCTGGATAAAGCGGCTATG
CCGGATTTAAGTTCCGTGAATTCAGGAAGCGGTGTGGCGACACTAATTAACCCGCAGTAT
ATCGCCAGTGTGAAACATAACGGGGGATATACAAACGTCAGCTTTGGTGATAGTGAAAAC
CGTTACAATATAGTAGACCGGAATAATGCGACTTCACTGGATTTCCATGCTCCCAGACTG
GATAAACTGGTGACAGAGGTTGCCCCGACTGCGGTGACGGCGCAGGGGGCAGTGGCTGG
C
GCATATAAGGATAAGGAACGCTATCCTGTTTTTTATCGTCTGGGGTCTGGTACCCAGTAT

ATCAAGAACAGTAATGGACAACTGACACAAGTAGGTGGGGCATATTCCTGGCTGACCGGC
GGGACCGTCGGTAGCTTGTCATCCTACCAGAATGGTGAAATGATTAGTACCAGTTCAGGT
CTGGTTTTTGATTACCAATTAAACGGTGCCATGCCTATTTATGGTGAGGCCGGTGACAGT
GGTTCTCCTTTGTTTGCTTTTGATACTGTTCAGAATAAATGGGTGCTGGTTGGTGTTCTC
ACCGCGGGGAATGGTCCAGGTGGCAGGGGAAATAACTGGGCAGTTATTCCTCTTGATTTT
ATCGAGCAAAAATTTAACGAGGACAATGATGCTCCGGTTACGTTCAGTTCATCTGAGGGG
GGAGTACTGGAGTGGAGCTTTGACAGCAGCACCGGAGTTGGCACCCTGACCCAAGGGAC
C
ATCACATATGACATGCACGGACAGCTGGGAAATGACCTGAATGCCGGTAAGAATCTGGAA
TTTCTGGGGCAGAATGGGCAGATTGAACTGAAGGATTCGGTTTCGCAGGGCGCGGGTTCT
CTGACGTTCCGTGATAATTACACAGTAACAACCTCTAACGGAAGTACCTGGACTGGGGCT
GGTATTATTGTGGATAAAGGAGTGTCCGTAAACTGGCTGGTCAACGGCGAGGCAGGTGAC
AATCTGCATAAAATCGGTGAGGGTACGCTGACGGTACAGGGTACAGGTATCAATGAGGGA
GGTCTGAAGGTCGGGGATGGGAAGGTTGTTCTGAGTCAGCAGGCGGACAGTAAAGGACA
G
GTGCAGGCTTTCAGCAGTGTAAATATTGCCAGTGGCCGACCGACTGTGGTACTAACTGAT
GACCGACAGGTAAACCCGGATACCATTTCTTGGGGATATCGTGGGGGGATTCTGGATGTT
AATGGTAACGATCTGACGTTTCATCAGCTGAAGGCCGCAGATTATGGTGCTGTGCTGGCA
AATAATTCTGATAATCATGCCTCAATTACGCTGGATTTCGATACAGAAGAATATCTGTAT
CATGGGCAGCTAAAAGGAAACGTGGATGTAACTAATGTGGTTGAAGTTGGGCAGAGCGCT
GTACTGGTCATGGATGGCTCCGCTGATATATCCGGTAAATTTACCCAGGAAAACGGGCGT
CTGACGTTGCAGGGGCATCCGGTTATCCATGCATACAATACTCAGTCTGTGGCTGACAAA
CTGGCTGCCAGTGGAGACCATTCGGTTCTGACTCAGCCCACATCATTCAGTCAGGAGGAC
TGGGAGAACCGGAGCTTTACCTTTGACAACCTGTCACTGAAGAACACCGATTTTGGCCTT
GGCCGTAATGCCACGCTGAACACCATGCTTGAAGCAACAGATTCGACGGTCACACTGGGC
GACAGCCGGGTTTTTATCGACAAAAACGACGGTAACGGTACGACCTTTACGCTGGAAGAA
GGCACATCAGAGGCTGTGAAGGATGCAGACCGTAGCGTGTTTAATGGGAGCGCTGTCCTG
AACGGGAAGACGACACTGGATATCATGAACGCAACCTTTAATGGTGATATCAGCGGTCAT
GCCAGCTCTCATGTAGAGTTGTCTAGTCGAAGTCACTGGAATATGACGAAAAGCTCCACG
CTGGATTCGTTCAGAAGCAAAGGCGGGACGCTCTCCCTTGTGACAGACAACTGGTCGCCG
AAAACGCTGACGGTAAACACACTGCATGCCAGCAGTATGAATATCGCTATGGGTGTCAGC
ACTGCCGACAACACGGGCGACCGGATTGATATTCTGAATAAGGCAACTGGCGGACATAAC
ACACTGGACCTGTCCACCCTGTTTGACCAGACTCTCACACTGAAAAATGACCTGACTCTG
GCATCTGCACCGGTCGGAACTTCTCACGGGTACTTCTCCTTTGCCAGCCTGAACAGGGGG
TTTACTGTTTATACCCCTGACACACAGGTGCAGGAGAAAGACGGCAGAGTTTACTGGCAA
CTGAAAAGTCATGCCGGAACAACAGAATCTCAGATATCACCGGGTGTCAGTGATGGTGTG
ACAGATACAACCTCTCCGGTTGCACCGGATACAGGTAATACAGGAAGCACTGAGGCTGAT
GGTATTGTGTCTGAAGGGAATAACAGCAGTGGTGTGACGCCTTCCCCTGATTCTCCGGGA
GAGAATGCGGGCACGACGGTGAATGGCAGTACGCTGTTTAAGGGCGCGGATAACACATCT
CTTCTGAAGAAAGCGCGTGTCATGTTTTCCGCCCGTGAGTTTATTCTCAGCGACAGCGCT
GACCGCTGGACACAGGTGGTGGACAACAGCGATGCAGACGGTGGTGTATGGGCCATGAC
A
GGGTACAGTCATGGTGGATATGACGACTTCAGCCTAAACCAGAGTGGACTGAATGTGGGA
TTTCGCCAGTCTGCTGCCAGTAATACCTGGTGGGGGATGGGGGCAGAGTTTTACCGGGGG
CACAGCAGCACAGATGGCTACCGGGATGATTTCAGCCTGTGGGGCGTGCATGTTCTGGCA
GGTAAATCCTTTGACGGTGGTCTGTTTGTGGATGGTATGATCGGATACCGGGAGCTGTCA
GAAGATTACACTATTCAGGGAGAGCTGAGTGACCTGTCCGGCAGAGCAAAAAGCCATATC
CTGACAGCGGGTATCCGTGGCGGGTGGAAGATGTATGCATCCCCTCTTGATATGTCGATA
ACACCGACTGTTTCACTGAACGGTGCACGGGTGGACGGAAATCGTCTGCAGGGCCGGGA
G
CGGAGCGTGGAGCTTCATGATGGCGATGCCTTGTGGCTGAAAGCCGGGGTTGAGGCAGA
A
AAATTATTTGGAAATATGACGCTGAAGGCAGGTATCTGGCGGAATATCACGTTTAACGAT
ATGCCCGGAATGACCCTACGTGATGACTGGAAAGCCCGTCACTATGATGCAGAGAAAGCT
GACCGCTATACCGTTTCGTTTGGTATCAACGGAAAGCTGACAGATAAACTGTCAGTACAG
GCAAAAGTGAACAGCAGTTTTGATGGTTACTTTAAGACCGATGCTGAAGGGATACTGGGT
ATTCGGTATGATTTCTGA

>ecf1__STEC__EHEC_factor_for_adherene__O157_Sakai
ATGTTGAATGCAAGGCATCTTCCCGTACTGATGTATCATCATGTCAGTCGTTGTCCGGGG
CTTGTGACACTCTCACCTGAAACATTTTGTGAACAGATGAAGTGGCTCGCAGATAATAAC
TGGCGGACGGTAACATCTGCTGAGCTTGAATATTTTTATCAGGGGGGAACGCTTCCACGA
AAGAGTGTCATGCTCACATTCGATGATGGTTATCTTGATAACTGGCTCAGAGCATGGCCG
GTCCTGAAGGAATATAACCTTCATGCACATATTTTTCTCATTACGGGGCTGATTGGTAAG
GGAAACGTACGTAGCAGGCAGGAGCAGGAATATTCTCACCGCGACTGTGAACGGTTGATT
GCCGATAACCGTTCAGATGAGGTCATGCTTCGCTGGTCTGAAGTCCGGGAAATGCGTGAT
TCCGGACTGGTTGAATTTCATGTGCACACCCATTCACATAAACGCTGGGACAGGTTGTCT
GTATCACGTGCTGAACAGTGCCGGCTCATGAAAGAAGATATTCTGGTGGGAAAACAATGT
CTGACGGAAAAGCTGGGGTTCTGTAGTTCCCATCTGTGCTGGCCTGAAGGATATTATAAT
CGGGATTATATTAATCTCGCCGGTAAGCTTGGTTTCTCTTATTTATATACAACCGAAAGA
AGAATGAACTGTCCTGAAAATGGTTCATTACGCATAGGGCGTATCAGCACCAAAGAGCGG
GAACATTCCGGCTGGCTGAAACGACGCCTTTTTATTATACAACGCCACTTTTTTCTTCA
GTACTGGCTCTTCATAAGGGGCCAAGATTACCAGATAATTAA
>ecf1__STEC__EHEC_factor_for_adherene__O26_11368
ATGTATCATCATGTCAGTCGTTGTCCGGGGCTTGTGACACTCTCACCTGAAACATTTTGT
GAACAGATGAAGTGGCTCGCAGATAATAACTGGCGGACGGTAACATCTGCTGAGCTTGAA
TATTTTTATCAGGGGGGAACGCTTCCACGAAAGAGTGTCATGCTCACATTCGATGATGGT
TATCTTGATAACTGGCTCAGAGCATGGCCGGTCCTGAAGGAATATAACCTTCATGCACAT
ATTTTTCTCATTACGGGGCTGATTGGTAAGGGAAACGTACGTAGCAGGCAGGAGCAGGAA
TATTCTCACCGCGACTGTGAACGGTTGATTGCCGATAACCGTTCAGATGAGGTCATGCTT
CGCTGGTCTGAAGTCCGGGAAATGCGTGATTCCGGACTGGTTGAATTTCATGTGCACACC
CATTCACATAAACGCTGGGACAGGTTGTCTGTATCACGTGCTGAACAGTGCCGGCTCATG
AAAGAAGATATTCTGGTGGGAAAACAATGTCTGACGGAAAAGCTGGGGTTCTGTAGTTCC
CATCTGTGCTGGCCTGAAGGATATTATAATCGGGATTATATTAATCTCGCCGGTAAGCTT
GGTTTCTCTTATTTATATACAACCGAAAGAAGAATGAACTGTCCTGAAAATGGTTCATTA
CGCATAGGGCGTATCAGCACCAAAGAGCGGGAACATTCCGGCTGGCTGAAACGACGCCTT
TTTATTATACAACGCCACTTTTTTCTTCAGTACTGGCTCTTCATAAGGGGCCAAGATTA
CCAGATAATTAA
>ecf1__STEC__EHEC_factor_for_adherene__O103_12009
ATGTTGAATGCAAGGCATCTTCCCGTACTGATGTATCATCATGTCAGTCGTTGTCCGGGG
CTTGTGACACTCTCACCTGAAACATTTTGTGAACAGATGAAGTGGCTCGCAGATAATAAC
TGGCGGACGGTAACATCTGCTGAGCTTGAATATTTTTATCAGGGGGGAACGCTTCCACGA
AAGAGTGTCATGCTCACATTCGATGATGGTTATCTTGATAACTGGCTCAGAGCATGGCCG
GTCCTGAAGGAATATAACCTTCATGCACATATTTTTCTCATTACGGGGCTGATTGGTAAG
GGAAACGTACGTAGCAGGCAGGAGCAGGAATATTCTCACCGCGACTGTGAACGGTTGATT
GCCGATAACCGTTCAGATGAGGTCATGCTTCGCTGGTCTGAAGTCCGGGAAATGCGTGAT
TCCGGACTGGTTGAATTTCATGTGCACACCCATTCACATAAACGCTGGGACAGGTTGTCT
GTATCACGTGCTGAACAGTGCCGGCTCATGAAAGAAGATATTCTGGTGGGAAAACAATGT
CTGACGGAAAAGCTGGGGTTCTGTAGTTCCCATCTGTGCTGGCCTGAAGGATATTATAAT
CGGGATTATATTAATCTCGCCGGTAAGCTTGGTTTCTCTTATTTATATACAACCGAAAGA
AGAATGAACTGTCCTGAAAATGGTTCATTACGCATAGGGCGTATCAGCACCAAAGAGCGG
GAACATTCCGGCTGGCTGAAACGACGCCTTTTTATTATACAACGCCACTTTTTTCTTCA
GTACTGGCTCTTCATAAGGGGCCAAGATTACCAGATAATTAA
>ecf1__STEC__EHEC_factor_for_adherene__O111_11128
ATGTTGAATGCAAGGCATCTTCCCGTACTGATGTATCATCATGTCAGTCGTTGTCCGGGG
CTTGTGACACTCTCACCTGAAACATTTTGTGAACAGATGAAGTGGCTCGCAGATAATAAC
TGGCGGACGGTAACATCTGCTGAGCTTGAATATTTTTATCAGGGGGGAACGCTTCCACGA
AAGAGTGTCATGCTCACATTCGATGATGGTTATCTTGATAACTGGCTCAGAGCATGGCCG
GTCCTGAAGGAATATAACCTTCATGCACATATTTTTCTCATTACGGGGCTGATTGGTAAG
GGAAACGTACGTAGCAGGCAGGAGCAGGAATATTCTCACCGCGACTGTGAACGGTTGATT
GCCGATAACCGTTCAGATGAGGTCATGCTTCGCTGGTCTGAAGTCCGGGAAATGCGTGAT
TCCGGACTGGTTGAATTTCATGTGCACACCCATTCACATAAACGCTGGGACAGGTTGTCT
GTATCACGTGCTGAACAGTGCCGGCTCATGAAAGAAGATATTCTGGTGGGAAAACAATGT
CTGACGGAAAAGCTGGGGTTCTGTAGTTCCCATCTGTGCTGGCCTGAAGGATATTATAAT
CGGGATTATATTAATCTCGCCGGTAAGCTTGGTTTCTCTTATTTATATACAACCGAAAGA

AGAATGAACTGTCCTGAAAATGGTTCATTACGCATAGGGCGTATCAGCACCAAAGAGCGG
GAACATTCCGGCTGGCTGAAACGACGCCTTTTTATTATACAACGCCACTTTTTCTTCA
GTACTGGCTCTTCATAAGGGGCCAAGATTACCAGATAATTAA
>efa1__STEC__adhesin__O157_Sakai
ATGATTCATCCTGGCTCTTCTTTAGATAAAGCAATTAACAATACAAGAGTTAAAAATGTT
AGTACTGATGTTAAACATGGCCAAATTCAAGAGAGAAAAAGGAACTTTATCTATAAAAAA
AATGATGATATCTCATCCAGATTTAAACTTTACAGTTCGCTCGTAAAGCAGAAAAATGCG
ACAGAAGATGTTGTTTTAATAGGCAAAATGATTTTAGATGAAGTTAGAAGTTACAGAACT
ATACATAATGATCGAAATATCGTAAGTAACTCAGGAAACTGGAAAACATCTTTTTTATGT
AATCTTGCTAGACTACTATATAGCATATTTAATGGTAGTAACTATTTTTGTTCCCGAGAG
GGTGAAAATAATTCATCCCCCAGTTCTACTCTACTTACTATACATCAGCCTGAAAAGCAG
GAACTATTACAACAAAAGAGTATCAAACATTTACCAACAAGTAATAACATCGACGGATAC
ATTAAAATAAGAAAAACAAGAGGCGCTGAAGATCAAACAACAACTATCACTCAAAGTTTG
ATAATTAATGAGTTGTTAAATGGAGTTGATAGAAATACCATCCCTTTTCAGAAAATAAGT
GAGCTCAATGATATCATACATTCATATGAAAATATGCAAATTAAAAATAGTCGAAAAGGT
ATAGAAATACTTGTTAAGCAGGGAGAGCTGTTATCATCATTAATAAATGTTAATAAAGGA
AATAAACAATTATCAGACAATGCATCTAAAATAATAAACTTATTGGGTATAGAGTATCAG
TCACATAAAGTAGACATAGAGCCATTTATACATGCAGTATGGGTTGCTGGCGCACCTCCT
GATAATACATTTTCATATATAACGGCATTTTTAAATACATATAAAGATTATACCTACCTG
CTCTGGATTGATCCTAATGCCTTTGGTGCGGCCAAGTTTAGTGGGATTTTAAAAAATATA
GCGATGAATTATGCAATTATGCGTTTGAGAAGAACGAATCCACATTTAGCGGAAGAAATG
AATGAAGTGATATTAAAAATACAAAACATACAAAATGAAACTATAGAATTTAAGGAAACA
AGAGAAAGATTGAAGGAACTTGAAAATAGATATAAATCATTAACGAGTGAAACTAAAGAA
AAATTCAATGTTTTTTTTCTTGAGTCAATGATAGGTATGCAAGACAACTATTTTACATAT
TGTATATCAAACGGAATATCAAATACTGATGATATATCCAGACTTGACTTTTTAACTAAC
GTTTTAAAACTTTCACCAGAAGTTCAAAATGACTTTAAATCTACAGTAGAGAAAAACAAA
AGAGATATAGACTTATTAAAAAATACAATATCTCAAAAATTTGGGGATAGATTTCAACTG
AGAGATATTAACACATTGGAGTCATTTAAAAAACCACAAGATTATTTTTTTATCAACAA
GAGATGCTATTAAGATGGAACTACGCCGCTGCATCAGATCAGGTAAGAATTAATATATTG
AAAGAGTATGGAGGGATTTATACCGATACAGATATTCTTCCTGCATATTCAGATAAAGTA
TCACAGATAATAAATGAAAAAAGTGATGATAAGAGGTTTTTTGAAGATTTAAAACTAAGA
AGGATTATATCAGAATCTATTCTGTCTCTGATAAAAGGGGAAAAGTACTCAATTAAACAT
GATGGATTAGATGAAACAACACTAAATCAGTTAAATAACATATTGAGTGAAATAGAAAAA
CTGACGATAGATGATTATTTTAAACCCGTTGAGACAAAAGTTGTCAGAGATACATTTAAA
ATTTTTAAAAGATATCAAAAATGGACAGAAAACACCTGGAATATTAGAGGGAATAATAAT
TTCATGTTAACTCACAAGGGAAGTAAATGTATTGATTTTATTCTATCAGGACAGAAAAAA
CAGTATTTAGAACTCCAACGCATCAGAGATAATATAAGTTATAATAATCTATTTTATACT
ACAGAGGATCTAAAGTCTTTAAATAATGTGGCAATAGGAGGAATACCTGCAAAGAAATAT
TTAGAACACGGTTTGTTTTCTGAATATAGACAGGACGGTACAATTCCTTATGTGGTTAGC
ACTTTAAATATATCTGGCCCAGACATGATTATGCGACAAATGAAAAAATACTATAAATCT
CTTGGGCGCATAGGTGAAGTGCATATTAAAGATAATAAATTGAGTGATGTTAATTTTTTG
GGTGTATATGCAAGTTCAAATAAAGACAATAAAAGTTTTAACTGGCTAAATCCAGTGAGT
GTTGGTATTAATGATATTACACCAGATGATGAAAGCTCATGGGCTGTCAGAAATAATGAT
ATAAATAAAATTCTATTCGAAAAAATAAACTGCCATGTACCCGAAAAACTACCTACCTCC
CTATACTATGAAATCGATTCGCGGTCTTTTTTTCAGGGGTGGGATAATAAAAGCATAAAG
CATGTTACAGAGATTAATAAAGACCTCATCAAAGATATTAATTTATTATTAACATCGTCT
AATATTGATGTTAAACTTTTAATAAAACTGGATCGTGAATTATATGCGATTAGCAGTAAA
ATAGATAATCCGTTGGCCTTGCGCTCTATAAGAACCTTACAGCTACAATTAGCTAATTAC
GTAACTTCGAATACATTTGAACCTGAAAACACAATTAATTTTATTTATGATTTTTATAGA
AAAAAACAAGATGACTTACTCAGCGCAATAAAATTATTTTCGCGTAATGATGCCGATACA
AAAATTATTGTCTGGTACAATTCCGTAATGGAAAAAAATGTGTTTCTCCGAGAGGTTATA
TCATGTGTTCTACGAAGTAAAAAAGTAGATAGTTATATAAATGAAAACAAAAAAAAACTTA
TCTAAGGAGGATGCCGGAGCTTTAAGAGATTATGCAAAATTAAAAATGAAAGAACTATTT
TCGATGTTAGATGATGATGGTTATAAAAAAATCATAACTACAAATGCATATATTAAAGAG
AGGGATAAACTATCAGGCATTATATACAACATCGAAAATTCGATTATATCGGGTCATGAA
TCATTTGATATAATACGAAGCAATCAACATGAGTGGGGGGATTTATCAACGGTAGAGCAA
TTTAAAAAATTTGAGTTTTATGTGAAAAGTGAATTATCTTCGGCTAAGTCAATTTTCGAT

GATATAAAAAACAAATATATAACAGATCCTGAAACAAAACGAAACGTACTCTACCATCAG
TTAGATAGTGATATTAAAGAGCGAATAGCATTTCTCGATATATCCCACTATGCATATCCG
GGTAGTTTATTAGAAAAATTACAATTAAGTGGCTACGTTTTTTCTGATATCAACATAATT
GCAGAATACTTATTGGCTAGTTACGGAGTTTCTGGACATTATTCTCACGGCGTGGTATAT
CCAGCTCCATCTGATAAATTACTTGAGCTATTAAGAAGACATACAAAGAGCAATTCTGAG
TGGATAGAAAAAATAACCCCATATGTTTATGATATATTATCCGATAATGTATCCAACGTC
TTGCGCCCCCCTTTATCAGAAGAACAGAAAAAAATATTGAATGATATAAAATTAGAAATT
AGTAAATCTGTATCAGAACAGTATTTCATGAAGTTGACAGAGCAAAAATCATCAGTTATT
GGTATTAAATATTCTGTTGATTTTGACAGATACAATGAGAATTTGTTTTTGTCTTTACCT
ATAAATCAAAATTTAACGCTGCCTTTTATGTATCGCTACTTCGAGATGCTATATGACATT
CACATTGGAATAATAGAAAACAAGGCAAACAGGGAGTTTATATATAGTAAGTTTTCATCT
TTAAACCTGGATTTTTTGATTAATGACGAGAGAGTTTTGAATCTTGAAGGTTTAATAAAA
AAATATAAATATTTATCGCTGAGTGAAATACATAGAACTTTAACAAATAGTACTTCATTT
GCAGATATTTCTATACCATTATTGCAGACCATATGTCCCAGCATAACAACAATAATAAAA
AAAACAGAATATTATGGGCATCAGTTAACAAATGCTATGACTGTTGCTTCAGTGGTTAAA
CCATATGACTTTAGCAATCTTGGTGCCATAAATAGTATAGATAAATCCGTTTCTGATGTG
CCTGCATTGCATACAATTGTTGAACAAGCAAAATATAATTTACTTTCATGGAATGATTTT
TATAACACTCATGCGTCAATATGGGATACAATAGCAAGACAGCATAAAAGTACAAATATC
GAATTTCACCCACAAAGTCTTCTTTTTGATAGAGATTCGAAAGGAAAGTGCCTTGGTTTA
TCTCTTCTGTATCTTGATACAGGGGGGTACGGAGGCGGATATCAAAAACTGAGGCACAAT
ATTGACACTGCCAGCACATTATATCAAACAAAATACAATGACAATCTAAAATTATCTAAC
CGTGATGATTTCTTTTTAAGAAAAACACAGCGTATCATTACAATGTCCAATGAGTTAGGT
AATAACAGGTTAAAAAATGCACAGTTAGAGGTTCTGGAATTGAAAGACCCTATACTAACT
GAAGGCATATTATACCAGCGTCGTATATCATCTCTTTTAATAACAACAGAATATCATTCA
CTTGCATTACAACAAATATCTTCTTTTTGGAGAGTAACTGATCCTAATTTTGGTCATTGT
GACTTTCATTCCTTAGCACAGGCTTTAACGTTTATAAAAAATATAACGAGCAATCGTAAC
TTTTCATCTTTATATGGAAGTGGCATTGTTAAAATTTACTTCAGTGAATCTCTTAATAAT
TGGAAGTATATTAAGCTCCCCCTTGTTCAGACTGGTTCTTTGTTAAGAGACATATATTTA
ACAACCCCAGAAAAATTATCCACAAGCGGGGGTTCATTAAATATTATGGGGCATCTCGTG
CCTGTCAGTTTTATTTATGATATCGGAGGGGTTATTAATGGTAACAGAATAAGTGAAAGT
ACAGATGTGAAAAATAAAATTAGGAGCTTAAAAATAAATGGAGATATTTTACAACATTAT
ATAAATACGCATTATTTGTCAGAGGAGCAAACTCAGAAGATAAAAGATATTGTGGATTTT
TTAGGTATTCAGGATAACACAATAAAAGTTAAGCTTGAAAGTGATATTAAACCTATAAGT
GAAATCCAACAGCCCCTTCATTCCATTCTTTCACGGCAGAAAGAACACGTAAAGAATTTA
CTATCTGGATTGCTTGATGAGTTTTCAAATAAGTTAAGAAAGCAAGGTCTTTCACTTAAA
ACAAATGTTCTTTCAGTTAATAACTTCAAAGAATCAAAGATAAATAGTGATACAGTGGAG
GTTACAGTAACAGATCTGCAAGGAAGACTTTATCGTGTTGATATAGATACAAGAGTTATT
GGCTTAACTTTTAAAGAGGGGATAAATAGTTTATCCGAAGCACTCGAACACATGAATATC
GATGCGATTATGTCTGTTATTGGCTTGGTTCAATATGCACGAATGATAAAAATGAATGAC
AATATTAGTGCAATAGATCATGCAGGAGCAGTATCAGATATAAAAAATATTGTAGACAAA
TTTTTAGGCGGCATTTTGACTCTTACTAATAATCGCGTATATAATCCTGGCGGTGTATCT
GGTGCCTCGCTTGAAGGATTTACATCTTCTGGGTTGGAGGTGTGTGCTTCAAGAATGGGT
GGCACCGCAGGGCGTTATCTTAGCAATGTGGCAAAAGTAATTAAATTACCTTTGCTAGAT
ATAGGCATAAATATTTGGTCATTATATGATTCTTCATTAAATCATGCTAAAGCCACCACA
CAAATTGAATATATATCAACAGCAATAGATGTATCTTTTTCATCTATAAATACAGCTTTG
TCAATAGGCGCAATCGCATATCCTCCTTTGGCAATAGCTATAGTTCCGATAACTATTTTT
TCTCATGAAGTAAAAAACTATGCTGTTTATGTGAATCAAATTAATGAGCGCCATAAGTTA
TGGCTCGAAGCTGAAAAGTATCTGGACAATGGAAGTGCAAAGGTTCTAAGCATTAACAAA
GCCACAGGCATAATCGATTTATCAAATAATCAGGTGTTAGGTAATATTTATTTAGATATG
AGAGAAAATCCTCCAATATTACACGGTGAGAAATCATATAATAGCGGAAAGAATATTGGT
AGTCATCCTGATATGACTGACAGAGAGATTATGGAGTCTAGAGCATACAATTTTGCATGC
ACAAAACTCTCAGATGCAGGTGAACCCGATATTTTCGGTTGGGGAGATAAGGAAATATGT
AATTCAATGGAATTATCAGAATCACAATTAGCTAATGGGTATTCTAACCGCCAATGGCCT
TCTCAGATTCCAGTTATTCCAGAAGGAATTTATAATACAGTATATCTTGGATATGGAGAA
ACATTGCGAGCTAATACTGAAGTTACATTGAGTAGCACTGGTTACTTTTACGAGATTGCA
AGGGCATATACTGATGATGAACTATCTGAGCCATTATTAACAGTATGTAACCAACATAGT
CATGTTATTGGTGGAAAAGAACCATTAACAATTATTATTCCCGCTATTGAACATGGTATG

CTTGGCTCTAATCTACATATGATTGAACGTTTTAAAAACTATAATTTCTCGATATCAGGA
GGGAAGGGAGGGATAAAATTAATGGTTGGTGGTATTGGTGATTACAACATAGAATGTACC
CCCGGTGTTAGAAATATTATATCGTTTGAACAATTATCACGTGACTTCAATCTGGATTTG
GATTTATCAGATGGGAGGAAACAAAATTTTAATTTTCATCATCCCTCAGGTTTTTATTCA
GGAAAAGTTATGAGCATAACGCAAAAGGGTATAAATGCGGTTGTTGGTACTAAACTGGGA
TATGATAAGATTCGTGGTAATAATCTTGATAATACCTTTTCTCTTGGGAGTGGAGGAGGT
GTTATATATTCTGGAGGAGGGAGCAATACCTATTTCGTTCCCGCCACACTACAGGATAAT
TTACACATATATATATCAGAAAAATCAAATGGCAATCATATTATATTAGGTGATATGCAC
TCTCGCCTATCTATTGAATGCCACTTTAGTAATAATGAAAGAGAGTTTATAAAGATGGGC
TACTATAATGGATGTGATGTGATTTTAGAAAGTGACACTATACAAAAAATAAAATCATTT
GCAAAAAATATAACAATACAGACAGCGGATGGAGTTATGGCAAATTGGGATGATAAACTA
AATACTCTGTCTGTTTATTCAATAGATATGATTGCATGGAGGGATAAAAATAAAACTGCA
AAAGAACCGTTACCAGTTGATGTAATCCAGTTAACTAATTGGAGGATGCATAACACATGC
TCTTTATTTTATGATAAATATCAAGTTGATATTGAAGCCAATAAGTTAACGTATACAGTC
TTATTTCCTGATACGGAGTTACCCGTTCAGCTCAATTATACCAGTATTATTTATGGTAAT
CATGGGGCAAAATACACCTTCCTCAATTCAGGAAGTAAAACTATAGATATTCACATTCTT
GATAAAAATAGTGATTATGATACTTTTGACTTCCGTAATATTATTTTTGAACATTACACC
AATGAGATATTTATAAGTTTTGATAATCAAGGAGGATTTGTTATATCAATATTAAATAAT
GCTACGAGTGAAGCGGCGAACATTAATGTATTCCGAAAAAACATGACTTCTTTAGATAGT
AGTGGTTCATTGATATATTTGCCTAGCGGTGATATTTATCATATTTCAGATATCTATAAA
ATGTCCAGAGGTAGAAAATCGTTTAAATTAAATGTAGAAAAGAAACCTGACATTGATGAT
ATCATCAATGTGGCTATTTTAGAAACTTCTTATCTACAAATAAAAAAAATCCCCAATAAT
GATGATAGTGATTATATTTTATGTTTAGACAACCCTAACCTCTCATCTTACACATTGAAC
TTTAATGACTTGTCTGGATACATATCAAGTTTATGGGATAATATTAGGGGTAGTTTTACC
CCTTTTCATAAAAATACTGTAAACATCGCCCCTAATGAAAAAAAATATATTTCCCTAATC
GGACTTGATAAGTTGTCATTCAATATTGATGTTTTAGACAAGCATTAGAGGTAAAAAAT
AAGAATAGTTACAAGATAAGTAAATTTACATGGGAAACGTATGGTGATATCGTTGTATCA
CCTGAAGATAGAATTTCTCATCTGGAGTTAGATGGGTTTAATTATTTTTCACAACCCGAG
TTAGATACTCCAATATCTGACTCATTTTCATATTTATATGATAATTTTCAGATCGTTGAT
AGTGATGTGCATATTAAGCTTCTTCATCTCAACAGAGAAACAAAGCAAATAACACCACAT
CGTATTATTCTTAAAAGATACTTTATAGACTCATTTGCTAAAACAAGTATCACTGACAGA
GAAAAGAATATATATCCTGTGATTTGTGATTCACCTGATCACTTCACAAGCGACATATAT
AGGCATCCATTTAGAATAGTTCTCGGGAATAAGACATTGTATCCATCAGAAGAACTTGTA
AAATTCATAAGCACCTCAAAAGAATATTTAAGCAATATGGATGTGATTAACAATGTTATT
GTTCCTCAAAAAACAACAAAGAAAAACAAATTATCCATAGTTAGTTTAAATAGCAATATC
AAAAATGATATTGTATTGTCCGGAGTAATGACCGGAACATCAAAGATTTTTCATCTAAAT
AATTCAGGTGATTTATTGTTAACAACCTCAAAAACTCATGGTGGTGGGGTTGTGGTTATT
TTTAAAGATTTTATAAATAACTGGTGGAAATATAATCTTACTTTAATAACAGTTCCTATT
GATAATAAATTGTCAGACAATAGAATAAATATAACCCCAATGGGGATAAAGATACAGGAA
ACAGTCAGTGGAAATGACAGGTTGTTTTTTACCCTACTCCATTAAAAAATGGATGTTTT
ATCTTGCATAATCCACTTTACTCTAACTCATTCCCTTCTTTATATTCACATGAATTATTT
GATTTGATTGAAGCATATAGAAATCCAACGTATTCGTATTTACAGAACAATATTATCAAT
AGATATATACATACTGTTTCTGAGTATGCTGGAAAAGACGGTATTGCCAATATGTCATTA
TCTATATACGCATACTCGACATGTCGTTTCTGGTCAAAAGAAAGTGTGGCTGTAGGAGAT
ACAATAAAATTGTCTAATTATGAGCATATTGAGTTTAGCTTTAATTCATTTAGTAAGGAT
TACTTTCGTGAACAAAATAGTGATATATATAAAATATTTTTTAAAATTGCTTCTTCGAGT
AATTCGGTTGTTGTGGATAAAAATACGCTCACTCAAAAAAACTTCCTAGAAAACAATACA
TTTTTCAGTATTACAGGTATAGATGAGTCTCTCTATGAAAAGCATATAATCTTTATCACT
CTTAAGGTTATTGTCCCCTCAAAAAAGTAG
>efa1__STEC__adhesin__O26_11368
ATGATTCATCCAGGTTCTTTTTTAGATAAGACAATTAACAATACAAGAGGTAAAAATGTT
AATGCTAATATCAAACATAGTCAAATTCAAGAGATAAAAAGGAATTTCACCTATAAAAAA
AATGATGACATCTCATCCAGATTTAAATTTTACAGCTCGCTAGTAAAGCAGAAAAACTCA
ACAGAAGATGTTGTTTTGATAGGAAAAATGATTTCAGATGAAGTTAGAAATTACAGCACC
ATACATAATGATAGAAATATCATAATTAACTCTGGAAATTGGAAAACATCTTTTTTATGT
AATCTTGCCAGACTACTATATAGCATGTTTAATGGTAGCGTCTATTTTCCTTCCCGTGAT
GATGAAAATAGCTCATCCCCCAGCCCAACTCTACCTACTATACATCAGTCTGAAAGGAAG

GAGTTATCACAACAAAAAAGCATCAATCATTTAAAAAAAAAGTGATACCATCGACGAATAC
ATTAAAATAAGAAAAACAAGAGGCATTGAAGATCAAATAACAACTACCTCTCAAAGTTTG
ATAATTAATGAGCTGTTAAATGGAGTTAATAGAAATACCATTCCATTTCAGAAAATAAGT
GAGCTCAATGACATCATACATTCATATGAAAATATGCAAACTAAAAATAGCCGAAAAGGT
ATAGAAATATTAGTTAAGCAGGGAGAACTGTTATCATCATTAATAAATTATGACAAAGAA
AACAAACAATTATCAGACAATATATCTAAAATAATAAATTTATTAGGGATAGAGTATCAG
TCACATAAAGTAGACATAGAGCCATTTATACATGCAGTATGGGTTGCAGGTGCACCTCCT
GAAAATACATCGTCATATATAACAGCTTTTTTAAATACATATAAAAATTATACTTACTTG
CTCTGGATTGATCCCAATGCTTTTGGTGCAGCCAAGTTTAGTGGAATTTTAAAAAATATA
GCCATGAATTATGCAATTATGCGTTTGAGAAGAACCAATCCTCATTTAGCGGAAAAAATG
AATGATGTGATATTAAAAATACAACACATACAAAATGAAACAGTAGATTTTAATGAAACA
AGAGAGAGGGTAAAGGAACTTGAAAATAAATACAAATCATTAACGAGAGAAACCAAAGA
A
AAATTCAATGTTTTTTTTCTTGAGTCAATGATAGGTATGCAAGACAACTATTTCACATAT
TGTATATCAAACGGAATATCAAATACTGATGATATATCCAGACTTAACTTTTTAACTACT
GTTTTAAAACTTTCACCAGACATTCTGGATGACTTTAAATCTACAGTAGAGAAAAACAAA
AAAGATATAGATTTATTAAAAGACATAATATCTCAAAAATTCGGGGATAGATTTCAACTG
AGAGATATTAACACATTGGAATCATTTAAAAAACCACAAGATTATTTTTTTATCAACAA
GAGATGCTATTAAGATGGAACTACGCCGCTGCATCAGATCAGGTAAGAATGAATATATTG
AAAGAGTATGGAGGGATTTATACCGATACTGATATTCTTCCTGCATATTCAGATGAAGTG
TCACAGATAATAAATAAAAAAAAGTGATGGAGATATGTTTTTTGAAGATTTAAAACTAAGA
AGGTTTATATCAGAAGCTATTCTGTCCCTCATAAAAGGGGAAAAGTACTCAATTAAGCAT
GATAGCTTAGATGAAAAAACACGTAATCAGTTAAATGCTATATTGAGTGAAATAGAAAAG
CTGACGATAGATAATTATTTTAAACCTGTTGAGACAACAGTTATTAGAGATAGTTTTAAA
ATTTTTAAAAGATATCAAAAATGGTCAGAAAACAACTGGAATATTAGAGGGAATAATAAT
TTCATGCTAACTCACAAGGGAAGTAAATGTATTGATTTTATTCAATCAGGACAGAAAAAA
CAATATTTAGAACTTCAGCGCATCAGAGATAATATAAGTTATAATAATTTCTTTTATACC
ACAAATGATCTAAAATCTTTAGATAACGTGGAAATAGGAGGAATACCTGCAAAAAAATAT
TTAGAACACGGTTTGTTTTCTGAATATCGACAGGACGGTACAATTCCTTATGTAGTAAGT
ACTCTAAATATATCTGGCCCAGACATGATTATGCGACAAATGAAAAAATACTATAAATCT
CTAGGGCGCATAGGTGAAGTACATATTAAAGACAATAAATTAAGTGATATGAATTTTATG
GGGGTATATGCAAGTTCAGATAAAGAAAATAAAAGTTTTAACTGGCTGAATCCAGTGAGT
GTTGGTGTTAATGATATTACACCGGATGATGAAAGCTCATGGGCTGTTAGAAATAATGAT
ATTAATAAAATTTTATTCGAAAAAATAAACTGTCATGTACCCGAAAAACTACCTACCTCC
CTATACTATGAAATCGACTCGCGAGTTTTTTTTCATGGATGGGATAATAAAAGCATACAA
TATGTTACTGAGATTAATAAAGACCTTATTAAAGATATAAATTTATTGTTAACATCGTCT
AATGTTGATGTTAAACTTTTAATAAAGCTAGATCGTGAATTATATGCAATAAGCAGCAAA
ATAGAGAATCCGTTAGCCTTGCGCTCTATAAGGACTTTACAGCTGCAATTAACTAATTAT
GTAACCTCAAACACATTTGAACCTGAAAATACAATTAATTTTATTTATGATTTTTATAGT
AAAAAACAAAATGATTTACTTAGCGCAATTAAGTTATTTTCGCGTAATGATGTCGAGACA
AAAATCATTGTTTGGTACAATTCAACAATGGAAAAAAATGTATTCCTCCGAGAGGTTATA
TCATGTGTTCTATGGACTAAAAAAGTAGACTCTTATATAAAAGAAAACAAAAAACACTTA
TCTACGGAAGATGCTGAAGCTTTAAGAGATTATGCAAAATTAAAAATAAAAGAACTATTT
TCGATGTTGGATGATGATGGTTATAAAAGAATCATAACAACAAATTCATATATTAAAGAG
AGGGATAAACTATCAGGCATTATACACAACATCGAAAATTCTATTATATCAGGTCATGAA
TCATCTGATATAATACGAAGCCATCAACATGAGTGGGGAGATTTATCAACGGTAGAGCAA
TTTAAAAAATTTGAATTTTATGTTAAAAGTGAGTTATCATTTTCCAAGTCAATTTTTGAT
GATATAAAAACCAAATATATAACAGATCCCGAAACAAAACGAAACGCACTTTACCATCAA
TTAGATAGTGATATCAAAGAGCGAATAGCCTTTCTCGACATATCCCACTACGCATATCCA
GGTAGTTTATTAGAAAAATTACAGTTAAGCGGCTACGTTTTTTCTGACATCAACATAATT
GCAGAATACTTATTGTCTAGTTACGGAATTTCTGGGCATTATTCTCACGGCGTGGTATAC
CCAGCTCCATCTGATAAATTATTTGAGTTATTAAGAAGACATACAAATTCCAATTCTGAT
TGGATAGAAAAAATAATCCCATATGTTTATGATATCTTATCAGGTAATGTGTCCAGCTTC
TTGCACCCTCCCTTATCAGAAGAACAGAAAAAAATATTGAGTGATATAAAATTAGAAATT
AGTGAATCTGTATCAGAACAGTATTTCATGAAGTTGACAGAACAAAAATCATCAGTTATT
GGCATTAAATACTCTGTTGATTTTGATAGATACAATGAGAATTTATTTTTGTCTTTACCT
ATAAATCAAAATTTAACTCTACCTTTCATGTATCGCTACTTCGAGATGTTATATGACATT

CACATTGGGATCTTAGAAAACAAGGCAAATAGAGATTTTATATATAGGAAGTTTTCATCT
TTAAATTTGGATTTTTTAATTAATGACGAGAGAGTTTTTAATCTTGAAGGCTTAATAAAA
AAATATAAATATTTATCATTGAGTGAAATACATAAAACTTTAACAAATAGTAATTCATTT
GCAGATATTTCCATACCATTATTACAGACTATATGTCCCAGTATAACAACAATAATAAAA
AAAACAGAATATCATGGACATCAGTTAACAAATGCTATGACAGTTGCTTCAGTGGTTAAG
CCATATGACTTTAGTAATCTTGGTGCCATAAATAGTATAGATAAGTCCGTTTCTGATATA
CCTGCATTGCATACAATTGTTGAACAAGCGAAGTATAATTTACTTTCATGGAATGATTTT
TATAATACTCATGCGTCAATATGGGATACAATAGCAAGGCAGCATAAAAGTACAAATATC
GAATTTCACCCACAAAGTCTTCTTTTTGATAGAGATTCGAAAGGAAAGTGCCTTGGTTTG
TCTCTCCTGTATCTTGATACAGGAGGTTATGGAAGCAGATATCAAAAACTGAGACACAAT
ATTGAAACTGCCAGCGCATTGTATCAAACAAAATATAATGACAATATTAAATTATCTAAT
AGCGATGATTTCTTTTTAAGAAAAACACAACGTATCATTACAATGTCCAATGAGTTAGGC
AATAATAGATTAAAAAACTCACAGTTAGAAATCCTAGAATTGAAGGACCCTGTACTAACT
GAAGGTATATTATACCAGCGTCGAATATCATCTCTTTTAATAACAACAGAATATCACTCA
CTCGCGTTACAACAAGTATCTTCTTTTTGGAGAGTAACCGATCCTAATTTTGGGCATTGT
GACTTCCATTCCTTAGCACAGGCTTTAACGTTTATAAAAAATATAACGAGCAATGGTAAC
TTTTCATCTTTATATGGAAGTGGTATTATTAAAATTTACTTTAGTGAGTCTCCTAATAAT
TGGAAGTATATAAAACTCCCTCTTATTCAGAATAATTCTTTGTCAAGGGATATATATTTG
ACAACTCCAGAGAAATTATCCGCAACTGGAGATTCATTAAATATTATGGGACATTTAGTG
CCTGTCAGTTTTATTTATGACATTGGAGGAGTCTTTAATGGCAACAGAATAAGTGAAAGC
ACAGATATAAAAAATAAAATCAAGAATCTGAAAATAAATGGGGATATTTTACAACATTAT
ATAAATACGCATTATTTGTCAGAGTCGCAAACTCAGAAGATAAAAGATATTGTGGATTTT
TTAGGCATTCAGGACAACACAAAAAGAGTTAAGCTTGAAAGTGATATTAAACATATAAGT
GAAGTTCAACAGCCCCTTCATTCTATTATCTCACGGCAGAAAGAACATGTAAAAAACTTA
CTATCTGGATTACTTAATGAGTTTTCAAATAAGTTAAGACAGCAAGGCCTTTCACTTAAA
ACAAATGTTCTTTCAGTTAATAACTTCAAAGCATCAAAGATAAATAGTGATACTGTGGAG
GTTACAGTAACTGATCTGCAAGGAAGACTTTATAGTGTTGATATAGATACAAGAGTTATT
GGCTTAACTTTTAAAGAGGGGATAAATAGTTTATCTGAAGCACTCGAACACATGAATATA
GATGCAATTATGTCTGTTATTGGCTTGGTTCAATATACACGAATGATAAAAACAAATGAC
AATATTAGCGCAATAGATCATGCAGGAGCAGTATCAGATATTAAAAATATTGTAGACAAA
TTTTTAGGCGGAATTTTAACTTTTACTAATAATCGCGTATATAACCCTGGTGGTATATCT
GGTGCCTCGCTTGAAGGATTTATATCTTCTGGTTTAGAAGTGTGCGCGTCAAGAATAGGT
GGCACCGCTGGGCGTTATCTTAGCAATGTGGCAAAAGTAATAAAATTACCTTTGCTAGAT
ATAGGCATAAATATTTGGTCATTATATGACTCTTCATTAAATCATGCCAGAGCCACCACA
CAAATTGAATATATATCAACAGCGATAGATGTATCTTTTTCATCTATAAATACTGCTTTA
TCAATAGGCGCAATCGCATATCCACCTTTGGCAATAGCTATAGTTCCGATAACTATCTTT
TCTCATGAAGTAAAAAACTATGCTGTTTATGTGAATCAAATTAATGAGCGACATAAGTTA
TGGCTTGAAGCTGAAAAATATCTGGACAATGGAAGCGCAAAGGTTCTAAGCATTAACAAA
GATACAGGCATAATCGATTTATCAAATAATCAGGTGTTAGGTAATGTTCATTTAGATATG
AGAGAAAACCCTCCGATATTACATGGTGAAAAATCATATAATAGCGGGAAGAATATTGGA
AGTCATCCAGATATGACTGACAGAGAAATTATGGAGTCTGGAGCATACAATTTTGCATGT
ACAAAACTCTCAGATGCAGGTGAACCCGATATTTTCGGTTGGGGAGATAAGGATGTATGT
AATTCAATGGAACTGTCTGAATCTCAATTAGCTAATGGGTATTCTAACCGCCAATGGCCT
TCACAAAGTCCAGTTATTCCTGAAGGGATTTATAATACAGTATATCTTGGGTATGGAGAA
ACATTGCGAGCTAATACAGAAGTTACATTGAGTAGCACTGGTTACTTTTACGAGATTGCA
AGGGCATATACTGATGATGAACTATCTGAGCCATTATTAACAGTATGTAACCAACGTAGT
AGTGTAATTGGTGGAAAAGAACCATTAACAATAATCATTCCAGCTCTTGAACATGGTATG
CTTGGCTCTAATCTACATATGATTGAACGTTTTAAAAACTACAATTTTTCAATATCAGGA
GGGAAGGGCGGGATAAAATTAATGGTTGGTGGTATTGGTGATTACAACATAGAATGTACC
CCCGGTGTTAGAAATATTATTTCTTTTGAACAATTATCACGTGACTTCAATCTGGATTTG
GATTTATCAGATGAGAGGAAACAAAATTTTTACTTTCATCATCCCTCTGGTTTTTATTCA
GGAAAAGTTATGAGTATAACGCAAAAGGGCATAAATTCGGTTGTTGGTACTAAACAGGGA
TATGATAAGATTCGTGGTAATAATTTAGATAATACCTTTTCCCTTGGAAATGGGGGGGGG
GTTATATATTCTGGTGGAGGAAGCAATACATATTTTGTTCCTGCTACACTACAGCATAAT
TTGCATATATATATATCAGAAAAATCAAATGGTAACCATATTATTTAGGTGAGACACAC
TCTCGCTTATCTATTGAATGCCACTTTAGTGATGATAAAAGAGAGTTTATAAAGATGGAC
TTATATAATGGAGGTGATGTAATTTTAGAAAGTGACACTTTACAAAAAATAAATACATTT

TCAAAAAATATAACAATACAGACAGCGGATGGAGTTATGGCAAACTGGGATGATGAATCA
AGTACTCTGTCTGTTTATTCAATAGATATGATTGCCTGGAGGGATAAAAATAAAACTGAA
AAAGAGCCTTTACCACTTGATGTGATTCAGTTAACTAATTGGAGGATACATAACTCATGC
TCTTTATTTTATGATAAATATCAAGTTGACATTGAAGCCAATAAATTAACGTATACAATT
TTATTTCCTGATACGGAGTTACCTGTTCAACTTAATTATGCTAGTGTTATTTATGGTAAT
CATGGGGCAAAATATACATTCCTCGACTCAGGAAGTAAAGCTATAACCATCCACATTACT
GATGAAAGTGGCGATTATGATACTTTTGACTTCCGCAATATTGTTTTTGAACATCACACC
AATGACATGTTTATAAGCTTTGATAATCAAGGAGGATTTGTTATATCAATATCAAATAAT
GCCACAAATGAACTGACAGATATTAATGTATTCCGAAAAAACATGACTTCTTTTGATGGC
TGTCGTTCATTGATATATTTGCCTAGCGGTGATGTTTTTTATATTTCAGATATCTATAAA
ATGTCCAGAGGAAGAAAATCGGTTAAATTAAATGTAGAAAAGAAACCGGACATTAACGAC
ATCATCAATGTGGCTATTTTAGAAACTTCTTATCTGCAAATAAAAAAAATCCCAAATAAT
GATGATGGCAGTTATGTTTTATGTCTAGACAACCCTAACCTCTCATCTTACACATTACAC
TTTAATGACTTATCTGGATATATATCAAGTATATGGGATAATATTAGAGGAAGCTTCACT
CCTTTTCATAATAATACTGTGAATATCGCCCCTAATGAAAAAAAATTTATTTCCCTAACA
GAGCCAGATAAGTTGTCATTCAACATTGATGTTTTTAGAGAAGCATTAGAGGTAAAAAAT
AGTTATAAGTTAAGTAAATTTACATGGGAAACGTATGGTGATATCGTGGTATCACCTGAA
GATAGAATTTCTCATCTGGAATTAGATGGATTTAATTATTTTTCACAACCCGAGTTAGAT
GCTCAAATATCAGACACCTTTTCATATCTATATGATAATTTTCAGATTGCTGATGGTGAT
GTTCATATTAAGCTTCTTCATCTCGATAGAGAAACAAAGCAAATAACGCCACATCGTATT
ATCCTTCAAAAATACTTTATAGACTCATTTGCTAAAACAAGTATCTCTGACAGAGAAAAC
AATATATATCCTGTTATTTGTGATTCACCTGATCACTTCACAAATGATATATATAGGCAT
CCATTTAGGATAGTTCTCGGGAATAAAACATTGTATCCATCAGAAGAACTTATAAAGTTT
ATAAGAACCTCAAAGGAATATTTAAGCAATATGGATATAATTAATAATATTATTGTTCCA
CAAAAAACAATAAAGAAAAATAAATTATCTGTCGTTAGTTTAAATAGCAACCTCAAAAAT
GAGGTTGTTTTGTCTGGAGTAATGACAGGAACATCAAAGGTTTTTCATCTAAATAAATCA
GGCGACTTATTGTTAACCACTTCAAAAACTCATGGGGGCGGGGTTATAGTTATTTTTAAA
GATTTTATAAATAACTGGTGGAAATATAACCTTACATTGATAACAGTTCCTATTGATGAC
AAATTGTCAGATAACAGGATAAATATAACACCAATGGGTATAACGATACATGAAATAGTC
AATGGGGATGATAAGTTGTTTTTTTATCCTACTCCATTAAAAAATGGAGGTTTTATCTTA
CATAATCCACTTTACACTAATTCATTTCCTTCTTCATATTCACATGAATTATTTGATTTG
GTTGAGGCATATAGAAATCCAACGTATTCATATTTACAGAATAGTATTATCAATAGATAT
ATACATACTGTTTCTGAGCATAATGGAAAAGACGGTGTAGCTCAGATGTCATTACCTATA
TATGCATACTCAACATGTCGTTTTTGGCCAAAAGAAAGTGTAGCTGTTGGAGATACAATA
AATTTATCTAATTATGAACATATTGAATTTAGCTTTAATTCTTTTAGTAAAGATGTCTGT
CGTGAACAAAATAGTGATATATATAAAATATTTTTTAAAATTGTGGCTTTGAATAATTCG
ATTGTTGTGAGCGAAAATATGTTCACTCAAAAAAACTTCTTGGAACACAATACATTTTTC
AGTGTTACCGGCATAGATGAGTCTCTCTATGAAAAGCATATAATCTTTATCACTCTTAAG
GCCATTCCTCCCCAGAAGTAG
>ehxA__STEC__enterohemolysin__O157_Sakai
ATGACAGTAAATAAAATAAAGAACATTTTCAATAATGCGACATTGACTACAAAATCAGCA
TTTAATACAGCATCATCAAGCGTACGTTCCGCTGGAAAAAAACTCATATTATTAATACCT
GATAATTATGAAGCTCAGGGCGTGGGTATTAATGAGTTGGTCAAAGCTGCTGATGAGCTT
GGAATAGAAATACACCGTACTGAACGAGATGATACAGCGATTGCAAACCAGTTTTTTGGT
GCAGCAGAAAAAGTTGTAGGATTAACTGAACGTGGTGTTGCAATATTCGCACCACAACTT
GACAAACTTCTGCAGAAGTATCAGAAAGTTGGGAGTAAAATAGGAGGAACCGCTGAAAA
T
GTAGGTAATAATCTGGGAAAAGCCGGAACAGTTCTCTCAGCACTACAGAATTTTACGGGG
ATTGCTTTATCAGGCATGGCTCTTGATGAATTGCTGAGAAAACAACGGGAAGGAGAGGAT
ATAAGTCAGAATGATATTGCCAAAAGTAGTATTGAACTTATTAATCAGCTTGTAGATACA
GTATCAAGTATAAACAGTACCGTTGATTCATTTTCTGAGCAGCTTAACCAGCTTGGCTCA
TTTTTATCCAGTAAACCTCGATTAAGTTCTGTTGGTGGGAAATTACAAAATTTACCAGAC
CTGGGCCCCCTGGGGGATGGGCTGGATGTTGTCTCCGGAATTCTTTCTGCTGTATCAGCA
AGCTTTATTCTGGGAAACAGTGACGCACATACAGGAACAAAAGCTGCAGCGGGTATCGAA
CTGACAACTCAGGTTCTTGGAAATGTTGGTAAAGCTGTTTCGCAATATATTCTGGCTCAG
AGAATGGCACAGGGGTTATCGACAACAGCTGCAAGTGCGGGTCTGATCACATCGGCTGTT
ATGCTGGCTATCAGTCCTCTTTCTTTCCTGGCTGCTGCAGATAAATTTGAGCGAGCTAAG

CAGCTTGAATCATATTCTGAACGATTTAAAAAATTGAATTATGAAGGGGATGCTTTACTC
GCAGCCTTTCATAAAGAAACCGGAGCTATAGATGCAGCCCTGACAACAATAAATACTGTC
CTGAGTTCTGTATCTGCGGGAGTTAGTGCAGCCTCCAGTGCATCCCTCATAGGGGCCCCG
ATAAGCATGCTGGTGAGTGCATTAACCGGTACGATATCTGGCATTCTGGAAGCATCAAAA
CAGGCTATGTTTGAGCACGTTGCAGAGAAATTCGCTGCTCGGATCAATGAATGGGAAAAG
GAGCATGGCAAAAATTATTTTGAGAATGGATATGACGCAAGACATGCTGCGTTTTTAGAA
GACTCTCTGTCTTTGCTTGCTGATTTTTCTCGTCAGCATGCAGTAGAAAGAGCAGTCGCA
ATAACCCAGCAACATTGGGATGAGAAGATCGGTGAACTTGCAGGCATAACCCGTAATGCT
GATCGCAGTCAGAGTGGTAAGGCATATATTAATTATCTGGAAAATGGAGGGCTTTTAGAG
GCTCAACCGAAGGAGTTTACACAACAAGTGTTTGATCCTCAAAAAGGGACCATAGACCTT
TCAACAGGTAATGTATCAAGTGTTTTGACATTTATAACACCAACATTTACCCCAGGAGAA
GAAGTTAGAGAAAGAAAACAGAGTGGTAAATATGAATATATGACATCTCTTATTGTAAAT
GGTAAGGATACATGGTCTGTAAAAGGCATAAAAAATCATAAAGGTGTATATGATTATTCA
AAATTGATTCAGTTTGTTGAAAAGAATAACAAACACTATCAGGCGAGAATAATTTCTGAG
CTCGGAGATAAAGACGATGTGGTTTATTCTGGAGCAGGCTCATCAGAAGTATTTGCTGGT
GAAGGTTATGATACCGTATCTTATAATAAGACGGATGTTGGTAAACTAACAATTGATGCA
ACAGGAGCATCAAAACCTGGTGAGTATATAGTTTCAAAAAATATGTATGGTGACGTGAAG
GTATTGCAGGAAGTCGTTAAGGAACAGGAGGTGTCAGTAGGGAAGCGAACAGAGAAAAT
A
CAATATCGTGATTTTGAATTCAGAACCGGTGGAATTCCTTATGATGTAATAGATAATCTT
CATTCTGTTGAAGAGCTCATTGGCGGAAAACATGATGATGAATTCAAAGGCGGTAAGTTT
AATGATATATTCCATGGCGCAGATGGGAACGATTATATCGAAGGTAATTATGGTAATGAT
CGACTATACGGCGATGATGGGGATGATTATATATCCGGAGGACAGGGAGACGACCAGTTA
TTTGGTGGTAGTGGAAACGATAAATTGAGTGGAGGGGATGGTAATAATTATCTGACAGGA
GGAAGCGGTAATGATGAGCTTCAGGCACACGGAGCTTATAATATTCTGTCAGGTGGTACT
GGTGATGATAAACTTTATGGTGGTGGTGGTATTGATCTTCTGGATGGAGGGGAAGGTAAT
GACTATCTGAATGGTGGTTTTGGTAATGATATTTATGTTTATGGGCAAAACTATGGTCAT
CATACAATTGCAGATGAAGGAGGTAAAGGAGATCGTTTGCACTTATCTGATATTAGCTTT
GATGATATCGCATTTAAGAGAGTTGGAAATGATCTTATCATGAATAAAGCCATTAATGGT
GTACTTTCATTTAATGAGTCAAATGATGTCAATGGGATAACATTTAAAAACTGGTTTGCG
AAAGATGCCTCAGGAGCAGATAATCATCTTGTTGAGGTTATAACAGATAAAGATGGTCGA
GAGATAAAAGTTGATAAGATACCTCATAATAATAATGAACGGTCAGGTTATATAAAAGCC
AGTAATATAGCATCTGAAAAAAACATGGTTAATATCACCAGTGTTGCCAATGATATTAAT
AAGATTATTTCTTCAGTTTCAGGGTTCGATTCAGGTGATGAACGATTAGCATCTTTATAT
AATTTATCCTTACATCAAAACAACACACACTCAACAACTTTAACGACAACTGTCTGA
>ehxA__STEC__enterohemolysin__O26_11368
ATGACAGTAAATAAAATAAAGAACATTTTCAATAATGCGACATTGACTACAAAATCAGCA
TTTAATACAGCATCATCAAGCGTACGTTCCGCTGGAAAAAAACTCATATTATTAATACCT
GATAATTATGAAGCTCAGGGCGTGGGTATTAATGAGTTGGTCAAAGCTGCTGATGAGCTT
GGAATAGAAATACACCGTACTGAACGAGATGATACAGCGATTGCAAACCAGTTTTTTGGT
GCAGCAGAAAAAGTTGTAGGATTAACTGAACGTGGTGTTGCAATATTCGCACCACAACTT
GACAAACTTCTGCAGAAGTATCAGAAAGTTGGGAGTAAAATAGGAGGAACCGCTGAAAA
T
GTAGGTAATAATCTGGGAAAAGCCGGAACAGTTCTCTCAGCACTACAGAATTTTACGGGG
ATTGCTTTATCAGGCATGGCTCTTGATGAATTGCTGAGAAAACAACGGGAAGGAGAGGAT
ATAAGTCAGAATGATATTGCCAAAAGTAGTATTGAACTTATTAATCAGCTTGTAGATACA
GTATCAAGTATAAACAGTACCGTTGATTCATTTTCTGAGCAGCTTAACCAGCTTGGCTCA
TTTTTATCCAGTAAACCTCGATTAAGTTCTGTTGGTGGGAAATTACAAAATTTACCAGAC
CTGGGCCCCCTGGGGGATGGGCTGGATGTTGTCTCCGGAATTCTTTCTGCTGTATCAGCA
AGCTTTATTCTGGGAAACAGTGACGCACATACAGGAACAAAAGCTGCAGCGGGTATCGAA
CTGACAACTCAGGTTCTTGGAAATGTTGGTAAAGCTGTTTCGCAATATATTCTGGCTCAG
AGAATGGCACAGGGGTTATCGACAACAGCTGCAAGTGCGGGTCTGATCACATCGGCTGTT
ATGCTGGCTATCAGTCCTCTTTCTTTCCTGGCTGCTGCAGATAAATTTGAGCGAGCTAAG
CAGCTTGAATCATATTCTGAACGATTTAAAAAATTGAATTATGAAGGGGATGCTTTACTC
GCAGCCTTTCATAAAGAAACCGGAGCTATAGATGCAGCCCTGACAACAATAAATACTGTC
CTGAGTTCTGTATCTGCGGGAGTTAGTGCAGCCTCCAGTGCATCCCTCATAGGGGCCCCG
ATAAGCATGCTGGTGAGTGCATTAACCGGTACGATATCTGGCATTCTGGAAGCATCAAAA

CAGGCTATGTTTGAGCACGTTGCAGAGAAATTCGCTGCTCGGATCAATGAATGGGAAAAG
GAGCATGGCAAAAATTATTTTGAGAATGGATATGACGCAAGACATGCTGCGTTTTTAGAA
GACTCTCTGTCTTTGCTTGCTGATTTTTCTCGTCAGCATGCAGTAGAAAGAGCAGTCGCA
ATAACCCAGCAACATTGGGATGAGAAGATCGGTGAACTTGCAGGCATAACCCGTAATGCT
GATCGCAGTCAGAGTGGTAAGGCATATATTAATTATCTGGAAAATGGAGGGCTTTTAGAG
GCTCAACCGAAGGAGTTTACACAACAAGTGTTTGATCCTCAAAAAGGGACCATAGACCTT
TCAACAGGTAATGTATCAAGTGTTTTGACATTTATAACACCAACATTTACCCCAGGAGAA
GAAGTTAGAGAAAGAAAACAGAGTGGTAAATATGAATATATGACATCTCTTATTGTAAAT
GGTAAGGATACATGGTCTGTAAAAGGCATAAAAAATCATAAAGGTGTATATGATTATTCA
AAATTGATTCAGTTTGTTGAAAAGAATAACAAACACTATCAGGCGAGAATAATTTCTGAG
CTCGGAGATAAAGACGATGTGGTTTATTCTGGAGCAGGCTCATCAGAAGTATTTGCTGGT
GAAGGTTATGATACCGTATCTTATAATAAGACGGATGTTGGTAAACTAACAATTGATGCA
ACAGGAGCATCAAAACCTGGTGAGTATATAGTTTCAAAAAATATGTATGGTGACGTGAAG
GTATTGCAGGAAGTCGTTAAGGAACAGGAGGTGTCAGTAGGGAAGCGAACAGAGAAAAT
A
CAATATCGTGATTTTGAATTCAGAACCGGTGGAATTCCTTATGATGTAATAGATAATCTT
CATTCTGTTGAAGAGCTCATTGGCGGAAAACATGATGATGAATTCAAAGGCGGTAAGTTT
AATGATATATTCCATGGCGCAGATGGGAACGATTATATCGAAGGTAATTATGGTAATGAT
CGACTATACGGCGATGATGGGGATGATTATATATCCGGAGGACAGGGAGACGACCAGTTA
TTTGGTGGTAGTGGAAACGATAAATTGAGTGGAGGGGATGGTAATAATTATCTGACAGGA
GGAAGCGGTAATGATGAGCTTCAGGCACACGGAGCTTATAATATTCTGTCAGGTGGTACT
GGTGATGATAAACTTTATGGTGGTGGTGGTATTGATCTTCTGGATGGAGGGGAAGGTAAT
GACTATCTGAATGGTGGTTTTGGTAATGATATTTATGTTTATGGGCAAAACTATGGTCAT
CATACAATTGCAGATGAAGGAGGTAAAGGAGATCGTTTGCACTTATCTGATATTAGCTTT
GATGATATCGCATTTAAGAGAGTTGGAAATGATCTTATCATGAATAAAGCCATTAATGGT
GTACTTTCATTTAATGAGTCAAATGATGTCAATGGGATAACATTTAAAAACTGGTTTGCG
AAAGATGCCTCAGGAGCAGATAATCATCTTGTTGAGGTTATAACAGATAAAGATGGTCGA
GAGATAAAAGTTGATAAGATACCTCATAATAATAATGAACGGTCAGGTTATATAAAAGCC
AGTAATATAGCATCTGAAAAAAACATGGTTAATATCACCAGTGTTGCCAATGATATTAAT
AAGATTATTTCTTCAGTTTCAGGGTTCGATTCAGGTGATGAACGATTAGCATCTTTATAT
AATTTATCCTTACATCAAAACAACACACACTCAACAACTTTAACGACAACTGTCTGA
>ehxA__STEC__enterohemolysin__O103_12009
ATGACAGTAAATAAAATAAAGAACATTTTCAATAATGCGACATTGACTACAAAATCAGCA
TTTAATACAGCATCATCAAGCGTACGTTCCGCTGGCAAAAAACTCATATTATTAATACCT
GATAATTATGAAGCTCAGGGCGTGGGTATTAATGAGTTGGTCAAAGCTGCTGATGAGCTT
GGAATAGAAATACACCGTACTGAACGAGATGATACAGCGATTGCAAACCAGTTTTTTGGT
GCAGCAGAAAAAGTTGTAGGATTAACTGAACGTGGTGTTGCAATATTCGCACCACAACTT
GACAAACTTCTGCAGAAGTATCAGAAAGTTGGGAGTAAAATAGGAGGAACCGCTGAAAA
T
GTAGGTAATAATCTGGGAAAAGCCGGAACAGTTCTCTCAGCACTACAGAATTTTACGGGG
ATTGCTTTATCAGGCATGGCTCTTGATGAATTGCTGAGAAAACAACGGGAAGGAGAGGAT
ATAAGTCAGAATGATATTGCCAAAAGTAGTATTGAACTTATTAATCAGCTTGTAGATACA
GTATCAAGTATAAACAGTACCGTTGATTCATTTTCTGAGCAGCTTAACCAGCTTGGCTCA
TTTTTATCCAGTAAACCTCGCTTAAGTTCTGTTGGTGGGAAATTACAAAATTTACCAGAC
CTGGGCTCCCTGGGGGGATGGGCTGGATGTTGTCTCCGGAATTCTTTCTGCTGTATCAGCA
AGCTTTATTCTGGGAAACAGTGACGCACATACAGGAACAAAAGCTGCAGCGGGTATAGAA
CTGACAACTCAGGTTCTTGGAAATGTTGGTAAAGCTGTTTCGCAATATATTCTGGCTCAG
AGAATGGCACAGGGGTTATCGACAACAGCTGCAAGTGCGGGTCTGATCACATCGGCTGTT
ATGCTGGCTATCAGTCCTCTTTCTTTCCTGGCTGCTGCAGATAAATTTGAGCGAGCTAAG
CAGCTTGAATCATATTCTGAACGATTTAAAAAATTGAATTATGAAGGGGATGCTTTACTC
GCAGCCTTTCATAAAGAAACCGGAGCTATAGATGCAGCCCTGACAACAATAAATACTGTC
CTGAGTTCTGTATCTGCGGGAGTTAGTGCAGCCTCCAGTGCATCCCTCATAGGGGCCCCG
ATAAGCATGCTGGTGAGTGCATTAACCGGTACGATATCTGGCATTCTGGAAGCATCAAAA
CAGGCTATGTTTGAGCACGTTGCAGAGAAATTCGCTGCTCGGATCAATGAATGGGAAAAG
GAGCATGGCAAAAATTATTTTGAGAATGGCTATGACGCAAGACATGCTGCGTTTTTAGAA
GACTCTCTGTCTTTGCTTGCTGATTTTTCTCGTCAGCATGCAGTAGAAAGAGCAGTCGCA
ATAACCCAGCAACATTGGGATGAGAAGATCGGTGAACTTGCAGGCATAACCCGTAATGCT

GATCGCAGTCAGAGTGGTAAGGCATATATTAATTATCTGGAAAATGGAGGGCTTTTAGAG
GCTCAACCGAAGGAGTTTACACAACAAGTGTTTGATCCTCAAAAAGGGACCATAGACCTT
TCAACAGGTAATGTATCAAGTGTTTTGACATTTATAACACCAACATTTACCCCAGGAGAA
GAAGTTAGAGAAAGAAAACAGAGTGGTAAATATGAATATATGACATCTCTTATTGTAAAT
GGTAAGGATACATGGTCTGTAAAAGGCATAAAAAATCATAAAGGTGTATATGATTATTCA
AAATTGATTCAGTTTGTTGAAAAGAATAACAAACACTATCAGGCGAGAATAATTTCTGAG
CTCGGAGATAAAGACGATGTGGTTTATTCTGGAGCAGGCTCATCAGAAGTATTTGCTGGT
GAAGGTCATGATACCGTATCTTATAATAAGACGGATGTTGGTAAACTAACAATTGATGCA
ACAGGAGCATCAAAACCTGGTGAGTATATAGTTTCAAAAAATATGTATGGTGACGTGAAG
GTATTGCAGGAAGTCGTTAAGGAACAGGAGGTGTCAGTAGGGAAGCGAACAGAGAAAAT
A
CAATATCGTGATTTTGAATTCAGAACCGGTGGAATTCCTTATGATGTAATAGATAATCTT
CATTCTGTTGAAGAGCTCATTGGCGGAAAACATGATGATGAATTCAAAGGCGGTAAGTTT
AATGATATATTCCATGGCGCAGATGGGAACGATTATATCGAAGGTAATTATGGTAATGAT
CGACTATACGGCGATGATGGGGATGATTATATATCCGGAGGACAGGGAGACGACCAGTTA
TTTGGTGGTAGTGGAAACGATAAATTGAGTGGAGGGGATGGTAATAATTATCTGACAGGA
GGAAGCGGTAATGATGAGCTTCAGGCACACGGAGCTTATAATATTCTGTCAGGTGGTACT
GGTGATGATAAACTTTATGGTGGTGGTGGTATTGATCTTCTGGATGGAGGGGAAGGTAAT
GACTATCTGAATGGTGGTTTTGGTAATGATATTTATGTTTATAGGCAAAACTATGGTCAT
CATACAATTGCAGATGAAGGAGGTAAAGGAGATCGTTTGCACTTATCTGATATTAGCTTT
GATGATATCGCATTTAAGAGAGTTGGAAATGATCTTATCATGAATAAAGCCATTAATGGT
GTACTTTCATTTAATGAGTCAAATGATGTCAATGGGATAACATTTAAAAACTGGTTTGCG
AAAGATGCCTCAGGAGCAGATAATCATCTTGTTGAGGTTATAACAGATAAAGATGGTCGA
GAGATAAAAGTTGATAAAATACCTCATAATAATAATGAACGGTCAGGTTATATAAAAGCC
AGTAATATAGCATCTGAAAAAAACATGGTTAATATCACCAGTGTTGCCAATGATATTAAT
AAGATTATTTCTTCAGTTTCAGGATTCGATTCAGGTGATGAACGATTAGCATCTTTATAT
AATTTATCCTTACATCAAAACAACACACACTCAACAACTTTAACGACAACTGTCTGA
>ehxA__STEC__enterohemolysin__O111_11128
ATGACAGTAAATAAAATAAAGAACATTTTCAATAATGCGACATTGACTACAAAATCAGCA
TTTAATACAGCATCATCAAGCGTACGTTCCGCTGGCAAAAAACTCATATTATTAATACCT
GATAATTATGAAGCTCAGGGCGTGGGTATTAATGAGTTGGTCAAAGCTGCTGATGAGCTT
GGAATAGAAATACACCGTACTGAACGAGATGATACAGCGATTGCAAACCAGTTTTTTGGT
GCAGCAGAAAAAGTTGTAGGATTAACTGAACGTGGTGTTGCAATATTCGCACCACAACTT
GACAAACTTCTGCAGAAGTATCAGAAAGTTGGGAGTAAAATAGGAAGAACCGCTGAAAA
T
GTAGGTAATAATCTGGGAAAAGCCGGAACAGTTCTCTCAGCACTACAGAATTTTACGGGG
ATTGCTTTATCAGGCATGGCTCTTGATGAATTGCTGAGAAAACAACGGGAAGGAGAGGAT
ATAAGTCAGAATGATATTGCCAAAAGTAGTATTGAACTTATTAATCAGCTTGTAGATACA
GTATCAAGTATAAACAGTACCGTTGATTCATTTTCTGAGCAGCTTAACCAGCTTGGCTCA
TTTTTATCCAGTAAACCTCGCTTAAGTTCTGTTGGTGGGAAATTACAAAATTTACCAGAC
CTGGGCTCCCTGGGGGATGGGCTGGATGTTGTCTCCGGAATTCTTTCTGCTGTATCAGCA
AGCTTTATTCTGGGAAACAGTGACGCACATACAGGAACAAAAGCTGCAGCGGGTATCGAA
CTGACAACTCAGGTTCTTGGAAATGTTGGTAAAGCTGTTTCGCAATATATTCTGGCTCAG
AGAATGGCACAGGGGTTATCGACAACAGCTGCAAGTGCGGGTCTGATCACATCGGCTGTT
ATGCTGGCTATCAGTCCTCTTTCTTTCCTGGCTGCTGCAGATAAATTTGAGCGAGCTAAG
CAGCTTGAATCATATTCTGAACGATTTAAAAAATTGAATTATGAAGGGGATGCTTTACTC
GCAGCCTTTCATAAAGAAACCGGAGCTATAGATGCAGCCCTGACAACAATAAATACTGTC
CTGAGTTCTGTATCTGCGGGAGTTAGTGCAGCCTCCAGTGCATCCCTCATAGGGGCCCCG
ATAAGCATGCTGGTGAGTGCATTAACCGGTACGATATCTGGCATTCTGGAAGCATCAAAA
CAGGCTATGTTTGAGCACGTTGCAGAGAAATTCGCTGCTCGGATCAATGAATGGGAAAAG
GAGCATGGCAAAAATTATTTTGAGAATGGCTATGACGCAAGACATGCTGCGTTTTTAGAA
GACTCTCTGTCTTTGCTTGCTGATTTTTCTCGTCAGCATGCAGTAGAAAGAGCAGTCGCA
ATAACCCAGCAACATTGGGATGAGAAGATCGGTGAACTTGCAGGCATAACCCGTAATGCT
GATCGCAGTCAGAGTGGTAAGGCATATATTAATTATCTGGAAAATGGAGGGCTTTTAGAG
GCTCAACCGAAGGAGTTTACACAACAAGTGTTTGATCCTCAAAAAGGGACCATAGACCTT
TCAACAGGTAATGTATCAAGTGTTTTGACATTTATAACACCAACATTTACCCCAGGAGAA
GAAGTTAGAGAAAGAAAACAGAGTGGTAAATATGAATATATGACATTTCTTATTGTAAAT

GGTAAGGATACATGGTCTGTAAAAGGCATAAAAAATCATAAAGGTGTATATGATTATTCA
AAATTGATTCAGTTTGTTGAAAAGGATAACAAACACTATCAGGCGAGAATGATTTCTGAA
CTCGGAGATAAAGACGATGTGGTTTATTCTGGAGCAGGCTCATCAGAAGTATTTGCTGGT
GAAGGTCATGATACCGTATCTTATAATAAGACGGATGTTGGTAAACTAACAATTGATGCA
ACAGGAGCATCAAAACCTGGTGAGTATATAGTTTCAAAAAATATGTATGGTGACGTGAAG
GTATTGCAGGAAGTCGTTAAGGAACAGGAGGTGTCAGTAGGGAAGCGAACAGAGAAAAT
A
CAATATCGTGATTTTGAATTCAGAACCGGTGGAATTCCTTATGATGTAATAGATAATCTT
CATTCTGTTGAAGAGCTCATTGGCGGAAAACATGATGATGAATTCAAAGGCGGTAAGTTT
AATGATATATTCCATGGCGCAGATGGGAACGATTATATCGAAGGTAATTATGGTAATGAT
CGACTATACGGCGATGATGGGGATGATTATATATCCGGAGGACAGGGAGACGACCAGTTA
TTTGGTGGTAGTGGAAACGATAAATTGAGTGGAGGGGATGGTAATAATTATCTGACAGGA
GGAAGCGGTAATGATGAGCTTCAGGCACACGGAGCTTATAATATTCTGTCAGGTGGTACT
GGTGATGATAAACTTTATGGTGGTGGTGGTATTGATCTTCTGGATGGAGGGGAAGGTAAT
GACTATCTGAATGGTGGTTTTGGTAATGATATTTATGTTTATAGGCAAAACTATGGTCAT
CATACAATTGCAGATGAAGGAGGTAAAGGAGATCGTTTGCACTTATCTGATATTAGCTTT
GATGATATCGCATTTAAGAGAGTTGGAAATGATCTTATCATGAATAAAGCCATTAATGGT
GTACTTTCATTTAATGAGTCAAATGATGTCAATGGGATAACATTTAAAAACTGGTTTGCG
AAAGATGCCTCAGGAGCAGATAATCATCTTGTTGAGGTTATAACAGATAAAGATGGTCGA
GAGATAAAAGTTGATAAAATACCTCATAATAATAATGAACGGTCAGGTTATATAAAAGCC
AGTAATATAGCATCTGAAAAAAGCATGGTTAATATCACCAGTGTTGCCAATGATATTAAT
AAGATTATTTCTTCAGTTTCAGGGTTCGATTCAGGTGATGAACGATTAGCATCTTTATAT
AATTTATCCTTACATCAAAACAACACACACTCAACAACTTTAACGACAACTGTCTGA
>eibG__STEC__adhesin__393/98
ATGAGTAAAAAGTTTACAATGACACTTCTGTCATCTTCTCTGGGTGGGCTTCTGCTTGGG
ATCAGCTCAGGTGTTTTAGCTCAGGAGTTACCTCCTATCAAAGATTCTGGTTTGCCTTTT
TATTCAGTGCTGAAAGAGGCAGATGCTTCGCATGTTACATACGGTAGTACTTATCGTATT
TACTATAACAAATCCTCTGGTTGGGGAACAATGCGATATGTTCTGAACGGAAAAGATGAG
ACCCTTTTTAATTTCGATCAGGAGGGTAATATTATAGTATTGAGTAAAGATAACTCAATT
GCATATACCGTTCATGAGCCTGTGCTAAAAGACTTTGCCCGTATGGCAGCAGGCCTCAGA
ACGTCTGACAAAGAAAACGGTAAGCATCAGGTTGATGAGGAGGAGGTCCGCCGGATCTT
C
AATAAAGTTAATAATCTATCAAATACTATTATTAATCCTGAAATATTAAAACAATCGGCT
TTCATCGCATCAGGACCACGTCCGGAAGGTGACCGTTTGGTTCAGGCTAGTGCGGCGGGC
GAATTCGCTCTGGCTGTTGGTACAGGGGCCAGAGCGGATAAAAAACTTGCAACATCAGTG
GGATCATGGTCTGCAGCAAGTGGACAGCAAAGTGTGGCTTTAGGTGGGGGAACATATGCT
TATGCAGATGCATCTACAGCCCTGGGAAGCGTTGCATTTGTTGATAACACGGCTACATAT
GGCACGGCAGTAGGTAACCGAGCCAAAGTTGATAAAGATGCAACAGAAGGTACTGCGTTA
GGGGCAAAGGCAACAGTAACAAATAAAAACAGTGTTGCATTAGGAGCTAATTCACGCACA
ACCCGTGACAATGAGGTTTATATCGGTTATGAAGCAGAGCCGGGTAAGGCTTATAAAACC
CGGGTGCTTGGTGGTCTGAGTGACGGTACTCGTCCCTCAGATGCTGCAACCGTTCGTCAG
GTTGACCGTGTAAAAGACAGTGTTGAGCAACTGGCTCAGGATACGAATACCCGCCTTGTG
GTTGAAGCGAAAAAGTCTCGTGAATATACAGACTCCAGAACGACAGTTGGTGTTAACTCC
GACGGAACCCTGACCCGTGCAGAAGGAGCCAGCAAAACCCTCGCTGTTAATGACGGTCT
A
GTTGCTTTGTCCGGCAGAACAGACCGTATTGATGCTGCAGTTGGTAGCGTTGACCGCCGG
GTTACTAAAAATACACAGGCTATTCAGTCCAATACTCGCCAGTTGCAGGAGCATAATGCA
CGCCTGAACAGCCAGCAGCGCCAGATTCGTGAAAACCACGAAGAGATGAAGCGTGCAGC
A
GCACAGAGTGCAGCGCTGGCGGGTCTTTTCCAGCCGTACAGTGTGGGGAAATTCAACGCC
ACGGCAGCCCTGGGTGGTTACAGCGATAAACAGGCGATTGCTGTAGGTGTTGGTTACCGT
TTCAACGAGCAGACCGCAGCGAAAGCGGGTATTGCAGCGAGTGATGGCGATGTGTCCTAC
AACATGGGCGTTAACTTCGAGTTTTAA
>espC__STEC__entero_toxin__E2348
ATGAATAAAATATACGCATTAAAATATTGTCACGCGACAGGGGGGCTGATTGCTGTATCC
GAACTGGCCTCCAGAGTTATGAAGAAAGCCGCTCGCGGCAGCCTTTTAGCATTATTTAAT
CTATCATTGTATGGTGCTTTTTTAAGCGCATCTCAGGCTGCTCAACTAAATATTGATAAT

GTATGGGCTAGAGATTATTTAGACCTCGCACAAAATAAGGGGGTGTTTAAAGCTGGTGCG
ACCAATGTTTCAATTCAACTCAAGAATGGCCAGACGTTTAATTTTCCAAATGTTCCAATT
CCTGATTTCTCGCCGGCCTCAAATAAAGGCGCTACTACATCTATAGGTGGAGCTTATAGT
GTCACAGCAACCCATAACGGAACAACTCATCATGCAATAAGCACCCAAAACTGGGGACAA
AGCTCATATAAATATATAGACCGGATGACGAATGGAGATTTTGCTGTAACACGACTTGAT
AAGTTTGTTGTTGAAACAACAGGGGTAAAAAATTCAGTAGATTTTTCTCTCAATAGTCAT
GATGCTCTTGAACGTTATGGTGTGGAGATCAATGGTGAGAAAAAAATCATTGGTTTCAGG
GTTGGGGCTGGGACGACTTATACCGTTCAAAATGGTAATACATATAGTACAGGACAGGTA
TACAATCCTCTTTTGTTAAGCGCTTCAATGTTTCAGTTAAACTGGGATAACAAAAGACCA
TATAATAACACGACACCTTTTTATAATGAAACTACCGGTGGAGACAGTGGTTCCGGTTTC
TATCTGTATGATAACGTAAAAAAAGAATGGGTTATGCTTGGTACTTTATTTGGAATAGCA
TCCAGTGGTGCAGATGTTTGGTCTATTCTGAATCAGTATGATGAAAATACAGTTAATGGT
TTAAAAAACAAATTTACTCAAAAAGTCCAGTTAAACAATAATACAATGTCGCTTAATAGT
GACAGTTTTACGTTAGCTGGTAATAATACAGCAGTGGAAAAAAATAATAATAACTATAAA
GATCTAAGTTTTAGTGGTGGTGGAAGTATTAATTTCGACAATGACGTAAACATTGGCTCT
GGTGGTCTCATTTTTGATGCAGGGCATCATTATACTGTCACTGGTAATAATAAAACATTC
AAGGGTGCCGGGCTGGATATTGGTGACAATACTACAGTCGACTGGAATGTGAAAGGGGTT
GTCGGTGATAACCTGCATAAAATTGGTGCAGGTACATTGAATGTTAATGTTTCTCAAGGT
AATAATCTTAAAACGGGGGATGGTCTTGTCGTATTAAATAGCGCTAATGCATTTGATAAT
ATTTATATGGCCAGTGGTCATGGTGTTGTAAAAATTAATCATAGTGCAGCGCTTAACCAG
AACAATGACTATAGAGGTATTTTCTTTACTGAAAATGGTGGTACTCTGGATTTAAATGGT
TATGACCAGAGTTTTAATAAAATTGCAGCGACAGATATAGGAGCACTCATAACAAATAGT
GCAGTGCAGAAAGCAGTTCTTTCTGTTAATAATCAGTCAAACTATATGTATCATGGTTCT
GTTTCAGGTAATACAGAGATAAACCACCAGTTTGATACCCAAAAAAATAATAGTCGCCTG
ATTCTGGACGGTAATGTCGATATTACAAATGACATTAACATTAAGAATAGCCAGCTCACC
ATGCAGGGACATGCTACATCTCATGCTGTTTTTAGAGAGGGTGGGGTTACCTGCATGCTG
CCAGGAGTTATTTGTGAAAAGGATTATGTTTCAGGCATACAGCAACAGGAAAACTCAGCC
AATAAAAATAATAATACAGATTATAAGACCAATAATCAGGTATCATCATTTGAGCAACCT
GACTGGGAAAATCGTCTGTTTAAGTTTAAGACATTGAATCTGATAAATTCAGATTTTATC
GTTGGCCGTAATGCTATTGTTGTTGGTGATATTTCTGCCAATAATTCCACTCTGTCTTTA
AGTGGAAAAGATACAAAAGTACATATTGATATGTATGACGGCAAAAACATCACGGGAGAT
GGCTTCGGTTTTCGGCAGGATATTAAAGATGGTGTATCTGTTTCTCCTGAGAGCAGCAGT
TATTTTGGAAATGTTACGCTGAATAATCACTCATTACTGGATATTGGTAATAAATTTACC
GGTGGTATCGAGGCTTATGACAGCTCCGTGAGTGTGACCTCACAGAATGCTGTTTTTGAT
CGTGTTGGCAGCTTTGTCAACAGCAGCCTGACCCTCGAAAAAGGAGCAAAACTAACGGC
T
CAGGGCGGTATTTTCAGCACCGGGGCTGTGGACGTAAAAGAAAATGCCTCCCTGATCCTG
ACGGGGACACCTTCTGCACAGAAACAGGAGTATTACTCCCCTGTGATTTCTACAACGGAA
GGGATTAACCTCGGAGATAAGGCCAGCCTTTCTGTTAAAAACATGGGCTATCTGAGTTCG
GATATTCATGCAGGAACCACGGCGGCAACCATTAATCTGGGAGACGGTGATGCTGAGACG
GATTCTCCGTTATTCAGCTCCCTGATGAAGGGATATAACGCGGTTCTGAGTGGCAACATT
ACGGGTGAGCAGAGTACGGTAAATATGAACAATGCTCTGTGGTACTCTGACGGAAACTCA
ACGATCGGAACGCTGAAGAGTACGGGGGGACGAGTTGAACTGGGGGGCGGAAAGACT
TT
GCCACCCTGCGGGTAAAAGAGCTTAACGCAAATAACGCCACATTCCTGATGCATACCAAC
AACAGTCAGGCTGACCAGCTGAATGTCACGAATAAACTGTTGGGCAGTAATAATACCGTC
CTGGTCGACTTTTTAAACAAGCCAGCCAGTGAAATGAACGTGACGTTAATTACCGCACCG
AAAGGGAGTGACGAGAAAACGTTCACTGCAGGAACGCAGCAGATTGGTTTCAGTAATGT
C
ACGCCGGTAATCAGCACAGAAAAAACGGATGATGCCACAAAATGGATGCTGACAGGGTAT
CAGACCGTCTCTGATGCCGGTGCCTCGAAAACCGCAACGGACTTTATGGCGTCAGGTTAT
AAATCCTTCCTGACAGAGGTCAATAATCTGAACAAGCGTATGGGTGACCTGCGGGATACT
CAGGGGGATGCCGGCGTCTGGGCGCGCATCATGAACGGTACCGGTTCGGCAGATGGTGGT
TACAGCGATAACTACACTCACGTTCAGATTGGTGCCGACAGAAAGCATGAGCTGGACGGT
GTGGATTTGTTCACGGGTGCATTACTGACCTATACAGACAGCAATGCAAGCAGCCACGCC
TTCAGTGGTAAAACCAAATCCGTGGGGGGAGGGTTGTACGCTTCAGCACTCTTTGATTCC
GGGGCTTATTTTGACCTGATTGGTAAATATCTCCATCACGACAATCAGTACACGGCGAGT

TTTGCGTCTCTTGGTACAAAAGACTACAGCTCTCATTCCTGGTATGCCGGTGCAGAGGTC
GGGTATCGTTACCACCTGTCGGAAGAGTCCTGGGTGGAGCCACAGATGGAGCTGGTTTAC
GGTTCTGTGTCAGGAAAATCTTTTAGCTGGGAAGACCGGGGAATGGCCCTGAGCATGAAA
GACAAGGATTATAACCCACTGATTGGCCGTACCGGTGTTGACGTGGGAAGAACCTTCTCC
GGAGACGACTGGAAAATTACCGCGCGAGCCGGGCTGGGTTACCAGTTCGACCTGCTGGC
G
AACGGAGAAACGGTTCTGCGGGATGCATCCGGAGAGAAACGTTTTGAAGGTGAAAAGGA
C
AGCAGAATGCTGATGAATGTGGGGATGAATGCGGAAATTAAGGATAATATGCGTTTTGGC
TTGGAGCTGGAAAAATCGGCGTTCGGGAAATATAACGTGGACAATGCGATAAACGCTAAC
TTCCGTTATTCTTTCTGA
>espP__STEC__serine_protease__O157_Sakai
ATGAATAAAATATACTCTCTTAAATACAGCCATATTACAGGAGGGTTAATCGCTGTTTCT
GAATTATCCGGCAGAGTATCATCAAGAGCAACTGGTAAGAAAAAACACAAACGCATACTT
GCATTATGTTTTTTAGGCTTATTACAATCCTCATATTCTTTTGCGTCACAGATGGATATT
TCAAATTTCTACATCCGTGACTATATGGATTTTGCACAGAACAAGGGCATATTTCAGGCT
GGCGCAACAAATATTGAAATAGTGAAGAAAGATGGCTCCACCCTGAAACTACCGGAAGTA
CCATTTCCTGACTTCTCACCGGTTGCAAACAAAGGGTCAACCACATCTATTGGTGGTGCA
TACAGTATCACAGCCACACACAATACGAAAAACCACCACTCAGTTGCGACGCAAAACTGG
GGGAACAGCACGTACAAACAAACTGACTGGAATACTTCACATCCTGATTTTGCAGTATCC
CGACTTGACAAGTTTGTTGTTGAGACCCGAGGTGCGACTGAAGGCGCAGATATTTCGTTA
TCAAAACAGCAGGCACTTGAACGTTACGGGGTTAATTATAAAGGAGAAAAGAAACTTATC
GCATTCAGAGCCGGCTCTGGTGTGGTATCCGTTAAAAAAAATGGACGCATAACTCCATTT
AATGAGGTTTCTTATAAGCCAGAAATGTTAAATGGCTCTTTCGTTCACATTGATGACTGG
AGTGGATGGCTGATATTAACCAACAACCAGTTTGATGAGTTTAATAACATTGCCTCTCAG
GGTGACAGCGGTTCAGCACTGTTCGTCTATGATAACCAAAAGAAAAAGTGGGTTGTCGCT
GGAACTGTCTGGGGGATTTATAATTACGCCAATGGCAAAAACCACGCAGCATACAGTAAA
TGGAACCAGACAACCATTGACAACCTGAAGAACAAGTATTCTTACAACGTGGATATGTCA
GGGGCTCAGGTTGCAACCATTGAAAATGGAAAACTGACAGGCACTGGCTCAGACACCAC
C
GATATAAAAAATAAGGACTTAATATTTACTGGCGGTGGAGATATCCTCCTGAAATCCTCT
TTTGATAATGGTGCTGGCGGTCTTGTCTTTAATGATAAAAAGACCTATCGAGTAAACGGG
GATGATTTCACCTTTAAAGGTGCCGGTGTTGATACAAGAAACGGCAGCACCGTTGAGTGG
AATATCCGGTATGATAATAAAGACAACCTTCACAAAATTGGTGATGGCACATTAGATGTC
CGAAAAACCCAGAACACCAACCTGAAAACAGGTGAGGGTCTTGTCATTCTTGGAGCTGA
A
AAAACATTCAATAATATCTACATAACCAGTGGTGATGGAACTGTCCGACTGAATGCAGAA
AATGCACTGTCTGGCGGTGAATACAACGGTATTTTCTTTGCGAAAAATGGCGGAACTCTT
GACCTGAACGGATATAATCAGTCTTTCAATAAAATTGCTGCAACTGATTCAGGTGCTGTA
ATAACCAATACGTCAACCAAAAAATCCATTTTATCCCTGAATAATACTGCTGACTATATC
TATCACGGTAACATAAACGGGAATCTGGACGTACTTCAGCATCATGAGACGAAAAAAGAG
AACCGTCGTCTTATTCTTGATGGGGGCGTGGACACAACAAATGATATAAGCCTGCGTAAT
ACACAACTGTCCATGCAGGGACATGCCACTGAACATGCCATTTATCGGGATGGAGCTTTC
TCTTGTTCACTACCAGCTCCTATGCGCTTTTTGTGTGGCAGTGATTATGTTGCAGGAATG
CAAAATACAGAAGCTGATGCTGTAAAACAAAACGGAAATGCCTATAAAACCAACAATGCT
GTCTCTGATTTATCGCAGCCAGACTGGGAAACCGGAACATTCAGATTTGGAACGCTACAT
CTTGAAAATTCCGATTTTTCTGTTGGTCGTAATGCAAATGTAATCGGGGACATTCAGGCC
AGTAAATCAAACATTACTATTGGTGACACTACAGCATATATTGATTTGCATGCTGGTAAA
AATATTACCGGTGATGGTTTTGGCTTCCGCCAGAATATTGTGCGTGGAAACTCACAAGGA
GAAACGCTGTTTACAGGAGGGATCACAGCAGAAGACAGCACTATCGTTATTAAAGATAAA
GCAAAAGCATTATTTTCAAATTATGTATACCTGCTGAACACAAAAGCAACCATAGAGAAC
GGTGCTGATGTGACAACTCAAAGTGGTATGTTCTCCACGAGCGATATCAGCATCTCTGGT
AATCTGTCCATGACAGGCAATCCCGACAAAGACAATAAATTCGAGCCCTCAATATATCTG
AATGATGCTTCTTATCTACTGACTGACGACTCCGCCAGACTCGTTGCCAAAAATAAAGCA
TCTGTGGTGGGAGATATACACTCCACTAAAAGTGCATCCATCATGTTTGGTCATGATGAA
AGCGACCTCTCGCAGTTGTCTGACAGAACCTCAAAAGGGCTTGCACTTGGTCTTTTAGGT
GGCTTTGATGTCTCATATCGCGGTTCAGTCAATGCCCCGTCAGCATCTGCCACTATGAAC

AACACCTGGTGGCAACTAACCGGAGATTCTGCGCTGAAAACACTGAAAAGTACAAACAG
C
ATGGTCTATTTCACTGACAGCGCAAACAATAAGAAATTCCATACGCTGACGGTCGATGAG
CTGGCAACCAGCAACAGCGCCTATGCGATGCGTACAAACCTTTCTGAATCAGACAAACTG
GAGGTCAAAAAACACTTGTCTGGTGAGAACAATATTTACTCGTTGATTTCCTTCAGAAA
CCAACGCCTGAAAAACAACTGAATATTGAACTGGTAAGCGCGCCAAAAGACACCAATGA
A
AATGTCTTTAAAGCCAGTAAACAAACCATTGGTTTCAGTGATGTAACGCCGGTCATTACA
ACCAGGGAAACCGATGACAAAATAACATGGTCACTGACAGGCTATAACACGGTAGCAAAC
AAGGAAGCAACCCGGAATGCCGCCGCCCTGTTCTCTGTTGACTATAAAGCGTTTCTGAAC
GAGGTCAACAACCTGAACAAACGTATGGGTGACCTGCGTGATATCAACGGCGAAGCCGGT
GCATGGGCACGCATCATGAGCGGTACCGGCTCTGCCAGTGGTGGTTTCAGTGACAACTAC
ACGCACGTTCAGGTCGGGGTCGACAAAAAACACGAGCTGGACGGACTGGATTTGTTTAC
C
GGTTTCACTGTCACACACACTGACAGCAGTGCCTCCGCCGATGTTTTCAGTGGTAAAACG
AAGTCTGTGGGGGCTGGCCTGTATGCTTCCGCCATGTTTGATTCCGGTGCCTATATCGAC
CTGATTGGCAAGTATGTTCACCATGATAATGAGTACACTGCAACCTTTGCCGGACTCGGA
ACCCGTGATTACAGCACGCATTCATGGTATGCCGGTGCAGAAGCGGGCTACCGCTATCAT
GTCACTGAGGATGCCTGGATTGAGCCACAGGCTGAGCTGGTTTACGGTTCTGTATCCGGT
AAACAGTTTGCATGGAAGGACCAGGGAATGCATCTGTCCATGAAGGACAAGGACTACAAT
CCGCTGATTGGCCGAACGGGTGTGGATGTGGGTAAATCCTTCTCTGGTAAGGACTGGAAA
GTGACAGCCCGTGCCGGTCTGGGCTACCAGTTCGACCTGCTGGCTAACGGCGAAACCGTA
TTGCGGGATGCATCTGGTGAAAAACGCATCAAAGGTGAAAAGGACAGCCGTATGCTGATG
TCCGTTGGCCTGAATGCAGAAATCAGGGATAACGTCCGCTTTGGACTGGAGTTTGAGAAA
TCCGCCTTTGGTAAGTACAACGTTGATAATGCTGTCAACGCTAATTTCCGTTACTCGTTC
TGA
>espP__clade1__serine_protease__10290
ATGAATAAAATATATTCTCTTAAATACAGCCATATTACAGGAGGGCTAATCGCTGTTTCT
GAATTATCCGGCAGAGTATCATCCAGAGCATCGGGTAAGAAAAACAAGAAAAAAATTATA
CTTCCATTATGTTTTTTAGGTTTATTATCTCCCTCATATTCTTTTGCGTCACAGATGGAT
ATTTCAAATTTTTACATCCGTGATTATATGGACTTTGCGCAGAACAAGGGGATATTTCAG
GCCGGCGCTACAAATATAGAAATAGAGAAGAAAGATGGCAGCAGCCTTAAACTACCTGAA
GTCCCGTTCCCTGACTTTTCACCTGTTGCAAACAAAGGTTCAACAACATCTATAGGTGGG
GCATACAGTATTACAGCAACACATAACACCAAAACCCACCACTCAGTTGCGGAGCAAAAC
TGGGGAAACACAACATACAAACAGGCAGACTGGCATACATCACACCCCGACTTCGCAGTA
TCCCGACTTGACAAATTTGTTGTTGAGACCAGAGGAGTGACTGAAGGCGCAGACACTTCG
TTATCAAAGCAACAGGCACTTGAGCGGTATGGCATTAATTATAAAGGCGAGAAAAAACTT
ATCGCGTTCAGGGCCGGTTCAGGAACAATTGGTATAAAAAAAGATGGAAAAACAACGCCA
TTTGATGAGGTTTTTTACAAACCAGAAATGTTAAATGGTTCTTTTGTTCATATTGATGAC
TGGAGTGGATGGTTAGTATTAACCAACAACCAGTTTGATGAATTTAATAACCTTGCAACT
CAGGGTGACAGTGGTTCAGCCCTCTTTGTATATGACAACGAAAAGAAAAAATGGGTTGTC
GCCGGAACTGCATGGGGCGTTTATTACTATTCCAATGGAAAAACGCATACTGCATATAGC
AAATGGGATCAGAACGCTGTTGATACCATAAAGAAAACATTTAGCCACAATGTTGATATG
ACCAATCAACAGGAAGTAACCATTGAAAACGGTAAACTTGCCGGCATCGGCACTGACACA
TCCGAAATAAAAAACAAAGACTTAATATTCACAGGTGGCGGTAACATCCTCCTGAAATCC
AGTTTTGACAATGGTGCTGGCGGTCTTGTCTTTAATGATAAAAAGACATATCAGATAAAC
GGGAATGATTTCACCTTTAAAGGTGCTGGTATTGACACAAGAAACGGTAGCACCGTTGAA
TGGAATATACGGTATGACAATAAAGACAACCTTCATAAAATTGGTGATGGCACTTTAGAT
GTCAGAAAATCCCAGAACACCAATCTTAAAACAGGTGACGGCCTTGTCATACTTGGTGCA
GAGAAAACCTTCAACAATATCTACATGGCCAGTGGTGATGGCACAGTCCGACTGAATGCA
GAAAAAGCGCTGTCTGACGGTGAGTACAACGGCATTTTCTTCGCGAAAAATGGCGGCACA
CTTGACCTGAATGGACACAATCAGTCTTTCAATAAAATTGCTGCAACTGATTCAGGCGCA
GTAATAACCAATACGTCGCCCCAAAAATCCATTTTATCCCTGAGTAATAATGCTGATTAT
ATCTATCACGGCAACATTAACGGGAATCTGGACGTGCTTCAGCATCATGAGACGAAAAAA
GAGAACCGCCGTCTTATTCTTGATGGTGGAGTGGACACAACAAATGATATAAGCCTGCGT
AATACACAACTGTCCATGCAGGGACATGCCACTGAACATGCTATTTATCGGGATGGTGGT
TTTGCGTGTTCACTACCCGCTCCTATGCGTTTTTTGTGTGGCAGTGATTATGTGGCAGGC

ATGCAAAATACAGAAGCAGATGCTGTAAAACAAAGCGGGTATACTTATAAAACCAGCAAT
GAAGTCTCGGATTTATCTCAGCCTGACTGGGAAAACGGAACCTTCAGATTTGGAACACTA
CATCTTGAAAATTCCGATTTTTCCATCGGTCGTAATGCCAATGTAATGGGAGACATTCAG
GCCAGCAAGTCAGACATTACAATTGGTGACACTACAGCATATATTGATTTACATGCAGGT
AAAAATATTACCGGCGATGGTTTTGGCTTCCGCCAGAATATTGTAAGCGGGCACTCTCAG
GGCGAAACCGGGTTTACTGGTGGTATAACTGCTGAAGACAGCAGCATTGTCATTAAAGAT
AAAGCAAAAGCGTTATTCTCAAATTATGTATACCTGCTTAACACAAAAACAACCATAGAG
AAAGGTGCTGATGTGACAGCTCAGAGTGGTGTGTTCTCCACGAACGATATCAGCGTCTCC
GGCAATCTCACCATGACAGGCTATCCTGACAAAGACAATGAATTCGAACCCTCAATATAT
CTGAATGATGCATCTTATCTACTGACTGACGATTCAGCCAGACTCGTTGCAAAAAATAAA
GCATCAGTGGTGGGGGATATAACTTCCACTAAAAGTGCATCCATCATGTTCGGCCATGAT
GAAAGCACCCCATCGAACCTGTCTGACAAAGCCTCAAAAGAGCTTGCTCTTGGTCTTTTA
GGTGGATTTGATGTCTCATACCGGGGTGCAATTCATGCCCCGCCAGCATCTGCCACTATG
AACAACACCTGGTGGCAACTAACCGGAGATTCTGCGCTGAAAACACTGAAAAGTACAAA
C
AGCATGGTCTATTTCACTGACAGCGCAAACAATAAGAAATTCCATACGCTGACGGTCGAT
GAGCTGGCAACCAGCAACAGCGCCTATGCGATGCGTACAAACCTTTCTGAATCAGACAAA
CTGGAGGTCAAAAAACACCTGTCTGGTGAGAACAATATTTTACTCGTTGATTTCCTTCAG
AAACCAACGCCTGAAAAACAACTGAATATTGAACTGGTAAGCGCACCAAAAGACACCAA
T
AAAAATGTCTTTAAAGCCAGTAAACAAACCATTGGTTTCAGTAATGTAACGCCGGTCATT
ACAACCCAGGAAACCGATGACAAAATAACATGGTCACTGACAGGCTATAACACGGTAGCA
AACAAGGAAGCAACCCGGAATGCCGCCGCCCTGTTCTCTGTTGACTATAAAGCGTTTCTG
AACGAGGTCAACAACCTGAACAAACGTATGGGTGACCTGCGTGACATCAACGGCGAAGC
C
GGTGCATGGGCACGCATCATGAGCGGTACCGGCTCTGCCAGTGGTGGTTTCAGTGACAAC
TACACGCACGTTCAGGTCGGGGTCGACAAAAAACATGAGCTGGACGGACTGGATTTGTTT
ACCGGTTTCACTGTCACACACACTGACAGCAGTGCCTCCGCCGATGTTTTCAGTGGTAAA
ACGAAGTCTGTGGGGGCTGGCCTGTATGCTTCCGCCATGTTTGATTCCGGTGCCTATATC
GACCTGATTGGCAAGTATGTTCACCATGATAATGAGTACACTGCAACCTTTGCCGGACTC
GGAACCCGTGATTACAGCACGCATTCATGGTATGCCGGTGCAGAAGCGGGCTACCGCTAT
CATGTCACTGAGGATGCCTGGATTGAGCCACAGGCTGAGCTGGTTTACGGTTCTGTATCC
GGTAAACAGTTTGCATGGAAGGACCAGGGAATGCATCTGTCCATGAAGGACAAGGACTAC
AATCCGCTGATTGGCCGAACTGGTGTGGATGTGGGTAAATCCTTCTCTGGTAAGGACTGG
AAAGTGACAGCCCGTGCCGGTCTGGGCTACCAGTTCGACCTGCTGGCTAATGGCGAAACC
GTATTGCGGGATGCATCTGGTGAAAAACGCATCAAAGGTGAAAAGGACAGCCGTATGCTG
ATGTCCGTTGGCCTGAATGCAGAAATCAGGGATAACGTCCGCTTTGGACTGGAGTTTGAG
AAATCCGCCTTTGGTAAGTACAACGTTGATAATGCAGTCAACGCTAACTTCCGTTACTCG
TTCTGA
>etpA__ETEC__Two-partner_secreted_adhesi__O78:H11:K80_str._H10407
ATGAACCGTATATATAAACTGAAGTTTGACAAACGCCGCAACGAACTGGTGGTGGTGAGT
GAAATCACCACCGGCGTGGGTAATGCAAAAGCCACGGGCAGCGTGGAGGGCGAAAAGTC
C
CCCCGTCGTGGCGTGCGCGCCATGGCGCTGAGCCTGCTGTCGGGTATGATGATAATGGCC
CATCCGGCGATGTCAGCAAACCTGCCGACCGGTGGCCAGATTGTGGCAGGTTCAGGCAGT
ATCCAGACGCCTTCCGGCAACCAGATGAATATTCATCAGAACAGCCAGAACATGGTGGCC
AACTGGAACAGCTTTGACATTGGTAAAGGAAATACGGTGCAGTTTGACCAGCCCAGCAGC
AGTGCGGTGGCGCTGAACCGTGTTGTGGGTGGCGGTGAATCGCAGATTATGGGTAACCTG
AAGGCGAATGGTCAGGTGTTCCTGGTTAACCCGAACGGCGTGCTGTTTGGTGAGGGGGCC
AGTGTCAGCACGTCAGGTTTTGTGGCATCGACCCGCGACATTAAAAACGACGACTTCATG
AACCGTCGTTACACCTTCAGCGGCGGACAGAAAGCCGGGGCAGCGATTGTGAACCAGGG
G
GAACTGACCACAAATGCCGGTGGCTATATTGTGCTGGCAGCAGACAGGGTCAGCAACAGT
GGCACCATCCGTACGCCGGGCGGCAAGACCGTCCTGGCGGCCAGCGAGCGCATCACGCT
G
CAGCTGGATAATGGTGGCCTGATGTCCGTGCAGGTGACAGGAGATGTGGTTAATGCCCTG
GTGGAAAACCGCGGTCTGGTCAGTGCCCGGGATGGTCAGGTGTACCTGACCGCACTTGGC

CGGGGTATGCTGATGAACACGGTACTGAACGTGAGCGGGGTGGTGGAAGCCAGCGGTATG
CACCGTCAGGACGGTAACATTGTACTGGACGGTGGCGACAGTGGTGTGGTGCACCTGAGT
GGTACCCTGCAGGCGGACAATGCGTCCGGTCAGGGTGGTAAGGTTGTCGTGCAGGGTAAG
AATATTCTGCTGGACAAGGGCAGCAACATCACAGCAACCGGTGGTCAGGGCGGCGGTGA
A
GTGTATGTCGGTGGCGGCTGGCAGGGTAAGGACAGCAACATCCGTAATGCGGACAAGGTG
GTGATGCAGGGCGGCGCCCGCATTGACGTTTCTGCAACGCAGCAGGGTAACGGCGGTACG
GCTGTGCTGTGGTCAGACAGCTACACCAACTTCCATGGTCAGATTAGCGCGAAGGGCGGT
GAGACCGGCGGTAACGGTGGTCGGGTGGAGACCTCTTCGCACGGTAACCTGCAGGCATTT
GGTACGGTCAGTGCATCCGCGAAGAAAGGCAAGGCGGGTAACTGGCTGCTGGACTCGGC
G
GATATCACCATTGTGAATGGTAGCAATGTTAGCAAAACTGAGACGACTCAATCGCCGCCG
CACACGCAATTTGCACCCACCGCTGCGGGCTCTGCGGTCAGCAATACCAGTATCAACAAC
AGGCTGAACAACGGGACCAGTGTCACTATTCTGACCCATCGCACAAGAACAGGCACAGC
T
CAGGGCGGGAATATTACCGTTAATGCGGCAATTAACAAAAGCAACGGAAGTGATGTCAAC
CTGACGCTGCAGGCTGGCGGCAACATCACGGTAAACAACAGCATCACGTCCACCGAGGG
T
AAGCTGAATGTTAATCTGTCGGGCGCCAGGACCAGCAATGGCAGTATCACCATTAGCAAT
AACGCCAATATAACGACCAATGGTGGGGATATAACTGTTGGGACGACAAATACTTCAAAC
CGTGTGAATATATCTATTAATAACACTACCCTGAATGCGTCAAATGGCAACATCCAGTTG
ACCGGGACCGGGACCGATAGCGGGATTCTGTTTGCTGGCAACAACAGGCTGACGGCCAG
T
AACATTGCTCTTACCGGGAACAGTACGAGTGGGAATGCCATCAACCTTACAGGCACTGCC
ACGCTGAATGCCACGAATAACATTACTCTTACCGGGAGCAGTACGAGTGGGAATGCCATC
AACCTTAAAGGCAACAACACGCTGACGGCCAGTAACATTACTCTTACCGGGGAAAGTACG
AGTGGGAATGCCATCAACCTTACAGACACTACAGGCACTACCACGCTGAATGCCACGAAT
AACATCACTATGCAGGGGACCCGTGTTCAGATTAAACACTCCAACATCACCGCGGGCAAC
TTTGCGCTGAATGCGACAGTGGCCGGCTCTGAAATCAGCAATACCACGCTGACGGCCACC
AACAACATCAACCTGGCGGCTAAGACGAACAGTGCGAGCTCTGGTGTTTACCTGAAAGAT
GCAAGAATTACATCCACCAATGGCAGTATCACGGCTAACGGTACTGCCACAGCAAACGGC
AAGGCCACGCATCTGGACGGCAACGTCACCCTGAATGCGTCAAATGGCAGAATCAAGTTG
ACCGGGAACGGGCACGGTAGCGCCTCCGGGATTCTGTTTGCTGGCAACAACAGGCTGAC
G
GCCAGTAACATTGCTCTTACCGGGAACAGTACGAGTGGGAATGCCATCAACCTTACAGGC
ACTGCCACGCTGAATGCCACGAATGACATTACTCTTACCGGGAGCAGTACGAGTGGGAAT
GCCATCAACCTTACAGGCACTGCCACGCTGAATGCCACGAATAACATTACTCTTACCGGG
AGCAGTACGAGTGGGAATGCCATCAACCTTAAAGGCAACAACACGCTGACGGCCAGTAA
C
ATTACTCTTACCGGGGAAAGTACGAGTGGGAATGCCATCAACCTTACAGACACTACAGGC
ACTACCACGCTGAATGCCACGAATAACATCACTATGCAGGGGACCCGTGTTCAGATTAAA
CACTCCAACATCACCGCGGGCAACTTTGCGCTGAATGCGACAGTGGCCGGCTCTGAAATC
AGCAATACCACGCTGACGGCCACCAACAACATCAACCTGGCGGCTAAGACGAACAGTGC
G
AGCTCTGGTGTTTACCTGAAAGATGCAAGAATTACATCCACCAATGGCAGTATCACGGCT
AACGGTACTGCCACAGCAAACGGCAAGGCCACGCATCTGGACGGCAACGTCACCCTGAA
T
GCGTCAAATGGCAGAATCAAGTTGACCGGGAACGGGCACGGTAGCGCCTCCGGGATTCTG
TTTGCTGGCAACAACAGGCTGACGGCCAGTAACATTGCTCTTACCGGGAACAGTACGAGT
GGGAATGCCATCAACCTTACAGGCACTGCCACGCTGAATGCCACGAATGACATTACTCTT
ACCGGGAGCAGTACGAGTGGGAATGCCATCAACCTTACAGGCACTGCCACGCTGAATGCC
ACGAATAACATTACTCTTACCGGGAGCAGTACGAGTGGGAATGCCATCAACCTTAAAGGC
AACAACACGCTGACGGCCAGTAACATTACTCTTACCGGGGAAAGTACGAGTGGGAATGCC
ATCAACCTTACAGACACTACAGGCACTACCACGCTGAATGCCACGAATAACATCACTATG
CAGGGGACCCGTGTTCAGATTAAACACTCCAACATCACCGCGGGCAACTTTGCGCTGAAT
GCGACAGTGGCCGGCTCTGAAATCAGCAATACCACGCTGACGGCCACCAACAACATCAAC
CTGGCGGCTAAGACGAACAGTGCGAGCTCTGGTGTTTACCTGAAAGATGCAAGAATTACA

TCCACCAATGGCAGTATCACGGCTAACGGTACTGCCACAGCAAACGGCAAGGCCACGCAT
CTGGACGGCAACGTCACCCTGAATGCGTCAAATGGCAGAATCAAGTTGACCGGGAACGG
G
CACGGTAGCGCCTCCGGGATTCTGTTTGCTGGCAACAACAGGCTGACGGCCAGTAACATT
GCTCTTACCGGGAACAGTACGAGTGGGAATGCCATCAACCTTACAGGCACTGCCACGCTG
AATGCCACGAATGACATTACTCTTACCGGGAGCAGTACGAGTGGGAATGCCATCAACCTT
ACAGGCACTGCCACGCTGAATGCCACGAATAACATTACTCTTACCGGGAGCAGTACGAGT
GGGAATGCCATCAACCTTAAAGGCAACAACACGCTGACGGCCAGTAACATTACTCTTACC
GGGGAAAGTACGAGTGGGAATGCCATCAACCTTACAGACACTACAGGCACTACCACGCTG
AATGCCACGAATAACATCACTATGCAGGGGACCCGTGTTCAGATTAAACACTCCAACATC
ACCGCGGGCAACTTTGCGCTGAATGCGACAGTGGCCGGCTCTGAAATCAGCAATACCACG
CTGACGGCCACCAACAACATCAACCTGGCGGCTAAGACGAACAGTGCGAGCTCTGGTGT
T
TACCTGAAAGATGCAAGAATTACATCCACCAATGGCAGTATCACGGCTAACGGTACTGCC
CCAGCAAACGACAATGCCACGTATCTGGACGGCAACGTCACCCTGAATGCGTCAAATGGC
AGCATCAAGTTGACCGGGAACGGGAACGGTAGCACCTCCGGGATTCTGTTTGCTGGCAAC
AACACGCTGACGGCCAGTAACATTACTCTTACCGGGAACAGTGAGGTGTACTGGCAATAG
>etpD__STEC__type-II_effector__O157_Sakai
TTGTTCAGGAAATGGTTGAATGGGCGTTTGCCGGTACTTGTGTTCACTACAGTAATTTTG
GGGGCCATTCCAGGGTGGGGGGCTGAATTTTCGGCCAACTTTAAAGATACGGATATTCAG
GAGTTCATAAATACTGTCAGTAAAAATTTACACAAAACGGTAATAATTAATCCTGACGTG
CAGGGAACCATCACTGTACGCAGCTACGATATGCTGAACGAGGAACAATATTATCAGTTC
TTTCTCAGTGTGCTGGACGTTTATGGTTTTGCTGTGGTCGATATGCACAACGGTATACTG
AAAGTAGTGCGCTCAAAAGATGCCAAAACGTCGGCGGTGCCGGTAGCTAGTGATGTCAGT
CCCGGGACTGGTGATGAGGTTGTTACCCGGGTGGTCCCCGTAAGTAACGTGGCAGCCAGA
GATCTGGCGCCTTTGCTGCGTCAGCTCAATGATAATGCTGGCGCAGGAAGCGTGGTGCAT
TATGAACCTTCTAATGTTTTGTTGATGACCGGACGTGCTGCAGTGATGAAACGGTTGATG
GAGATTGTTGAACGTGTGGATAAGGTGGGTAATCGCAGCGTTGCCACGGTCCCGCTCACC
TACGCGTCCGCAACAGACGTAGCCAGACTTGTTACGGAACTGACTAAAGAAACAGATAAG
ACAGCTATACCTGCTTGGATGACGGCGAAACTGGTTGCAGACGAGAGGACAAACTCAGTG
CTCGTCAGCGGAGAGCCAATCTCCCAACAGCGTATCATCTCCATAATTAAGCAACTGGAT
CGTCAGGAGGATGTTCAGGGTAATACTAAGGTGATTTACCTGAAATATGCGAAGGCGAAG
GATTTAGTGGAAGTCCTGACAGGTATCAGCAGCAGTATTGAAAACGACTCTAAAAAGAGT
CCGTCAACGGAAGCCTTGCGCAAAGGAGTGACGATTAAATCCCACGAACAAACCAATGC
C
CTGATCCTGACGGGGGCCCCTGACGTCATCCGCGACCTTGAAAATGTGATTTCGCAGTTG
GATATTCGTCGTCCTCAGGTCCTGGTGGAGGCCATCATTGCTGAAATACAGGATGCTGAC
GGGCTGAACCTTGGGATCCAGTGGGTGAATAAACATGCCGGTGTGGCGCAGTTCACCAGT
ACCGGTTTACCTATTACCACGATGGTTCAGACTCGTCAGAACGAAATCTTAGACAGCGAT
CAGAGCAATGCCCTGAGCATGTTTAACGGAATTGCAGCGGGGTTTTATCAGGGAAACTGG
GCGATGCTGTTGACGGCGCTCTCCACAAGTAGCAAGAATGATATCTTGGCGACCCCCAGT
ATTGTCACGCTGGACAATATGGAGGCCACTTTCAATGTTGGTCAGGAGGTCCCGGTACTT
TCGGGCTCACAGACAACCTCTGGGGACAATATTTTTAACACGGTCGAGCGCAAAACGGTG
GGGATCAAACTCAGGGTAAAACCCCAGATCAACGAGGGTGATTCCGTGTTACTGGAGATA
GAACAGGAGGTGTCCGGTGTGGCGGACACTGCAGTAGCCACCACTACTGACTTGGGAGC
A
ACCTTCAACACCCGAACAGTGACCAATGCCATGCTGGTCGGGAATGGCGAAACGGTGGTG
GTCGGAGGATTACTGGATAAGTCGATCAGGGGGAGTGAGAGTAAAGTGCCACTGCTGGGG
GATATCCCGGTACTGGGGCATCTTTTTCGCGCAAAAAGCGAACAGACAGCTAAGCGTAAT
CTGATGCTGTTCATTCGGCCAACTATTATTCGTGAGCGCGACGGATTTCGTCATGCTTCG
GCCGAAAAATACCAGTCGTTTAATCAGGAACAGGTGCAGTCGCGTGGCAAAGAAACAAC
G
GCGCTGACGCTGAATGAGGAACAGCTCAGGCTGTCCCCCGATCAAGACGATACGGCTTTC
CGGAAGGTGAAAGCGGCGATTGCTGCGTTTTATGCGCAGGAGATGTAA
>etpD__STEC__type-II_effector__O103_12009
TTGTTCAGGAAATGGTTGAACGGGCGCTTGTCGATATTTGTATTCACGACAGTATTTCTG
AGTGCCATTCCGGGATGGGGGGCTGAATTTTCGGCCAGCTTTAAAGATACGGATATTCAG

GAGTTCATTAATACTGTCAGCAAAAATCTGCACAAAACGGTAATAATTAATCCTGATGTG
CAGGGGACCATCACTGTACGCAGCTACGATATGCTGAACGAGGAACAATATTATCAGTTC
TTTCTCAGCGTGCTGGATGTTTATGGTTTTGCCGTGGTCGATATGCACAACGGTATACTG
AAAGTGGTGCGTTCAAAAGATGCTAAAACGTCGGCGGTGCCGGTAGCTAGTGATGCCAGC
CCTGGGACTGGTGATGAGGTTGTTACCCGGGTGGTCCCCGTCAGTAACGTGGCAGCCAGA
GACTTGGCGCCCTTGCTGCGTCAGCTCAATGATAATGCTGGCGCAGGAAGCGTAGTGCAT
TATGAACCCTCTAACGTTCTGCTGATGACTGGTCGTGCTGCCGTGGTGAAACGGCTGATG
GAGATTGTTGAACGGGTGGATAAGGAGGGCAATCGCAGCGTTGCCACGGTCCCACTTACC
TACGCCTCTGCAACGGATGTAGCCAGACTTGTCACGGAACTGACTAAAGAAAGTGATAAG
ACAGCCATGCCAGCCTGGATGACAGCGAAGCTGGTTGCTGACGAGCGGACAAACTCAGT
T
CTCGTCAGCGGAGAGCCAATCTCCCAACAGCGTATCATCTCCATGATTAAGCAACTGGAC
CGTAAGGAGGATGTTCAGGGTAATACGAAGGTGATTTACCTGAAATATGCGAAGGCGAAG
GATTTAGTGGAAGTCCTGACGGGCATCAGCAGCAATATTGAAAACGACGCTAAAAAGAGC
TCGTCAACGGAAGCTCTGCGAAAAGGGGTGACGATTAAATCCCACGAACAAACCAATGC
C
TTGATCCTGACAGGGGCTCCTGACGTCATCCGCGACTTGGAAAATGTAATTTCGCAGTTG
GATATTCGTCGTCCTCAGGTCCTGGTGGAAGCCATCATTGCTGAAGTACAGGATGCTGAC
GGGTTGAATCTGGGGATTCAGTGGGTGAATAAACACTCCGGTGTGACGCAGTTCACCAGT
ACTGGTTTACCCATTACCACGATGGTTCAGACCCGTCAGAGCGATATTTCTGACAGCGAT
CAGAGCAATACCTTGAGCATGTTTAACGGGATTGCAGCAGGGTTTTATCAGGGAAACTGG
GCGATGCTGCTGACGGCGCTCTCCACAAGTAGCAAAAATGATATCTTGGCAACCCCCAGT
ATTGTTACGCTGGACAATATGGAGGCCACGTTCAATGTTGGTCAGGAAGTTCCGGTGCTT
TCGGGTTCACAGACCACCTCTGGGGACAATATTTTTAACACCGTCGAGCGTAAAACGGTG
GGGATCAAACTCAGGGTAAAACCCCAGATCAACGAGGGTGATTCAGTGTTACTGGAGATA
GAGCAGGAGGTGTCCGGTGTGGCAGATACAGCAGTAGCTACCACTACTGACTTGGGGGCA
ACCTTCAATACCCGGACAGTGACCAATGCTATGCTGGTAGGGAATGGCGAAACGGTGGTG
GTCGGCGGATTACTGGATAAGTCGATCAGAGGGAGTGAGAGTAAAGTACCTCTGCTGGGG
GATATCCCAGTACTGGGACATCTTTTTAGCTCAAAAAGTGAACATGCAGCCAAGCGTAAT
CTGATGTTGTTCATTCGGCCAACAATTATTCGTGAACGCGACGGATTTCGTCATGCTTCG
GCCGAAAAATACCTGTCGTTTAATCAGGAGCAGGTGCAGTCGCGTGGTAAAGAAACAATG
GCGCTGACGCTGAATGAGGAACAGCTCAAGCTATCCCGAGATCGGGACGATACGACTTTT
CGGAAAGTGAAAACGGCGATTGCTGCGTTTTATGCGCGGGGGATGCAATGA
>fimB__ExPEC__T1_fimbriae__CFT073
ATGAAGAATAAGGCTGATAACAAAAAAAGGAACTTCCTGACCCATAGTGAAATCGAATCA
CTCCTTAAAGCTGCAAATACCGGGCCTCATGCTGCACGTAATTATTGTCTGACATTACTT
TGTTTTATTCATGGTTTCCGGGCGAGTGAAATTTGCCGATTGAGGATTTCGGATATTGAT
CTTAAGGCAAAGTGTATATATATCCATCGATTAAAAAAAGGCTTTTCAACAACGCACCCG
CTATTGAACAAAGAAATTCAGGCTTTAAAAAACTGGTTGAGTATCCGTACTTCTTATCCG
CATGCTGAGAGCGAGTGGGTATTTTTATCACGTAAGGGGAATCCACTTTCTCGGCAACAG
TTTTACCATATTATCTCGACTTCCGGTGGTAATGCCGGGTTGTCACTGGAGATTCATCCG
CACATGTTACGCCATTCGTGTGGTTTTGCTTTGGCGAATATGGGAATAGATACGCGACTT
ATCCAGGATTATCTTGGACATCGCAATATTCGTCATACTGTCTGGTATACCGCCAGCAAT
GCAGGGCGTTTTTACGGCATCTGGGATAGAGCCAGAGGACGACAGCGTCACGCTGTTTTA
TAG
>hes__STEC__Hemagglutinin_from_Shiga_toxin-producing_E._coli__RM10042
ATGAAAAAGGTTATTGTGGTTTCAGTATTTGCAATGGCGGGTATGTTTTCAGCCCAGGCC
TTGGCTGATGAGAGCAAAACAGGTTTTTATATGACCGGTAAGGCGGGGACTTCCGTTTTA
TCTCTTTCCAACCAGCGTCTTATCGATGGTGAAGGAGAGTGGGCGGACAAATATAAAGGT
GGTGATGACCATGATACGGTATTCAGTGGCGGTATCGCGGCTGGTTATGATTTTTATCCG
CAGTTCAGTATTCCGGTTCGTACGGAACTGGAGTTTTACGCTCGTGGAAAAGCTGATTCG
AAGTATAACGTAGATAAAGACAGTTGGTCAGGTGGTTACTGGCGTGATGACCTGAAGAAT
GAAGTGTCAGTCAACACACTGATGCTGAATACGTACTATGACTTCCGGAATGACAGCGCA
TTCACACCATGGATATCTGCAGGGATTGGCTACGCCAGAGTTCACCAGAAAACAACCGGT
ATCAGTATCTGGGATTATGGGTACGGAAACAGTGGTCGCGAATCGTTGTCACGCTCAGGC
TCTGCTGATAACTTTGCATGGAGCCTTGGCGCAGGTGTCCGCTATGACGTCACCCCGGAT
ATCGCGCTGGACCTCAGCTATCGCTATCTTGATGCAGGTGATGCCAGTGTGAGTTATAAG

GACGAGTGGGGCGATAAATATAAGTCAGAAGTTGATGTTAAAAGTCATGACATCATGCTT
GGTATGACTTATAACTTCTGA
>hlyA__ExPEC__haemolysin_A__UTI89
ATGCCAACAATAACCACTGCACAAATTAAAAGCACACTACAGTCTGCAAAGCAATCCGCT
GCAAATAAATTGCACTCAGCAGGACAAAGCACGAAAGATGCATTAAAAAAAGCAGCAGA
G
CAAACCCGCAATGCGGGAAACAGACTCATTTTACTTATCCCTAAAGATTATAAAGGACAG
GGTTCAAGCCTTAATGACCTTGTCAGGACGGCAGATGAACTGGGAATTGAAGTCCAGTAT
GATGAAAAGAATGGCACGGCGATTACTAAACAGGTATTCGGCACAGCAGAGAAACTCATT
GGCCTCACCGAACGGGGAGTGACTATCTTTGCACCACAATTAGACAAATTACTGCAAAAG
TATCAAAAAGCGGGTAATAAATTAGGCGGCAGTGCTGAAAATATAGGTGATAACTTAGGA
AAGGCAGGCAGTGTACTGTCAACGTTTCAAAATTTTCTGGGTACTGCACTTTCCTCAATG
AAAATAGACGAACTGATAAAGAAACAAAAATCTGGTAGCAATGTCAGTTCTTCTGAACTG
GCAAAAGCGAGTATTGAGCTAATCAACCAACTCGTGGACACAGCTGCCAGCATTAATAAT
AATGTTAACTCATTTTCTCAACAACTCAATAAGCTGGGAAGTGTATTATCCAATACAAAG
CACCTGAACGGTGTTGGTAATAAGTTACAGAATTTACCTAACCTTGATAATATCGGTGCA
GGGTTAGATACTGTATCGGGTATTTTATCTGCGATTTCAGCAAGCTTCATTCTGAGCAAT
GCAGATGCAGATACCGGAACTAAAGCTGCAGCAGGTGTTGAATTAACAACGAAAGTACTG
GGTAATGTTGGAAAAGGTATTTCTCAATATATTATCGCACAGCGCGCTGCACAGGGGTTA
TCTACATCTGCTGCTGCTGCCGGTTTAATTGCTTCTGTAGTGACATTAGCAATTAGTCCC
CTCTCATTCCTGTCCATTGCCGATAAGTTTAAACGTGCAAATAAAATAGAGGAGTATTCA
CAACGATTCAAAAAACTTGGATACGATGGTGACAGTTTACTTGCTGCTTTCCACAAAGAA
ACAGGAGCTATTGATGCATCATTAACAACGATAAGCACTGTACTGGCTTCAGTATCTTCA
GGTATTAGTGCTGCTGCAACGACATCTCTTGTTGGTGCACCGGTAAGCGCACTGGTAGGT
GCTGTTACGGGGATAATTTCAGGTATCCTTGAGGCTTCAAAGCAGGCAATGTTTGAACAT
GTTGCCAGTAAAATGGCTGATGTTATTGCTGAATGGGAGAAAAAACACGGTAAAAATTAC
TTTGAAAATGGATATGATGCCCGCCATGCTGCATTTTTAGAAGATAACTTTAAAATATTA
TCTCAGTATAATAAAGAGTATTCTGTTGAAAGATCAGTCCTCATTACTCAACAACATTGG
GATATGCTGATAGGTGAGTTAGCTAGTGTCACCAGAAATGGAGACAAGACACTCAGTGGT
AAAAGTTATATTGACTATTATGAAGAGGGAAAGCGGCTGGAAAGAAGGCCAAAAGAGTTC
CAGCAACAAATCTTTGATCCATTAAAAGGAAATATTGACCTTTCTGACAGCAAATCTTCT
ACGTTATTGAAATTTGTTACGCCATTGTTAACTCCCGGTGAGGAAATTCGTGAAAGGAGG
CAGTCCGGAAAATATGAATATATTACCGAGTTATTAGTCAAGGGTGTTGATAAATGGACG
GTGAAGGGGGTTCAGGACAAGGGGTCTGTATATGATTACTCTAACCTGATTCAGCATGCA
TCAGTCGGTAATAACCAGTATCGGGAAATTCGTATTGAGTCACACCTGGGAGACGGGGAT
GATAAGGTCTTTTTATCTGCCGGCTCAGCCAATATCTACGCAGGTAAAGGACATGATGTT
GTTTATTATGATAAAACAGACACCGGTTATCTGACCATTGATGGCACAAAAGCAACCGAA
GCGGGTAATTACACGGTAACACGTGTACTTGGTGGTGATGTTAAGGTTTTACAGGAAGTT
GTGAAGGAGCAGGAGGTTTCAGTCGGAAAAAGAACTGAAAAAACGCAATATCGGAGTTA
T
GAATTCACTCATATCAATGGTAAAAATTTAACAGAGACAGATAACTTATATTCCGTGGAA
GAACTTATTGGGACCACGCGTGCCGACAAGTTTTTTGGCAGTAAATTTACTGATATCTTC
CATGGCGCGGATGGTGATGACCATATAGAAGGAAATGATGGGAATGACCGCTTATATGGT
GATAAAGGTAATGATACGCTGAGGGGCGGAAACGGGGATGACCAGCTCTATGGCGGTGAT
GGCAATGATAAGTTAATTGGGGGGACAGGTAATAATTACCTTAACGGCGGTGACGGAGAT
GATGAGCTTCAGGTTCAGGGGAATTCTCTTGCTAAAAATGTATTATCCGGTGGAAAAGGT
AATGACAAGTTGTACGGCAGTGAGGGAGCAGACCTGCTTGATGGCGGAGAAGGGAATGA
T
CTTCTGAAAGGTGGATATGGTAATGATATTTATCGTTATCTTTCAGGATATGGCCATCAT
ATTATTGACGATGAAGGGGGGAAAGACGATAAACTCAGTTTAGCTGATATAGATTTCCGG
GACGTTGCCTTTAAGCGAGAAGGGAATGACCTCATTATGTATAAAGCTGAAGGTAATGTT
CTTTCTATTGGCCACAAAAATGGTATTACATTTAAAAACTGGTTTGAAAAAGAGTCAGAT
GATCTCTCTAATCATCAGATAGAGCAGATTTTTGATAAAGACGGCAGGGTAATCACACCA
GATTCTCTTAAAAAAGCATTTGAATATCAGCAGAGTAATAACAAGGTAAGTTATGTGTAT
GGACATGATGCATCAACTTATGGGAGCCAGGACAATCTTAATCCATTAATTAATGAAATC
AGCAAAATCATTTCAGCTGCAGGTAACTTCGATGTTAAGGAGGAAAGATCTGCCGCTTCT
TTATTGCAGTTGTCCGGTAATGCCAGTGATTTTTCATATGGACGGAACTCAATAACTTTG

ACAGCATCAGCATAA
>hlyA__ExPEC__hemolysin__CFT073
ATGCCAACAATAACCACTGCACAAATTAAAAGCACACTACAGTCTGCAAAGCAATCCTCT
GCAAATAAATTGCACTCAGCAGGACAAAGCACGAAAGATGCATTAAAAAAAGCAGCAGA
G
CAAACCCGCAATGCGGGAAACAGACTCATTTTACTTATCCCTAAAGATTATAAAGGACAG
GGTTCAAGCCTTAATGACCTTGTCAGGACGGCAGATGAACTGGGAATTGAAGTCCAGTAT
GATGAAAAGAATGGCACGGCGATTACTAAACAGGTATTCGGCACAGCAGAGAAACTCATT
GGCCTCACCGAACGGGGAGTGACTATCTTTGCACCACAATTAGACAAATTACTGCAAAAG
TATCAAAAAGCGGGTAATAAATTAGGCGGCAGTGCTGAAAATATAGGTGATAACTTAGGA
AAGGCAGGCAGTGTACTGTCAACGTTTCAAAATTTTCTGGGTACTGCACTTTCCTCAATG
AAAATAGACGAACTGATAAAGAGACAAAAATCTGGTAGCAATGTCAGTTCTTCTGAACTG
GCAAAAGCGAGTATTGAGCTAATCAACCAACTCGTGGACACAGCTGCCAGCATTAATAAT
AATGTTAACTCATTTTCTCAACAACTCAATAAGCTGGGAAGTGTATTATCCAATACAAAG
CACCTGACCGGTGTTGGTAATAAGTTACAGAATTTACCTAACCTTGATAATATCGGTGCA
GGGTTAGATACTGTATCGGGTATTTTATCTGCGATTTCAGCAAGCTTCATTCTGAGCAAT
GCAGATGCAGATACCGGAACTAAAGCTGCAGCAGGTGTTGAATTAACAACGAAAGTACTG
GGTAATGTTGGAAAAGGTATTTCTCAATATATTATCGCACAGCGCGCTGCACAGGGATTA
TCTACATCTGCTGCTGCTGCCGGTTTAATTGCTTCTGTAGTGACATTAGCAATTAGTCCC
CTCTCATTCCTGTCCATTGCCGATAAGTTTAAACGTGCCAATAAAATAGAGGAGTATTCA
CAACGATTCAAAAAACTTGGATACGATGGTGACAGTTTACTTGCTGCTTTTCACAAAGAA
ACAGGAGCTATTGATGCATCGTTAACAACGATAAGCACTGTTCTGGCTTCAGTATCTTCA
GGTATTAGTGCTGCTGCAACGACATCTCTGGTTGGTGCACCGGTAAGCGCGCTGGTAGGG
GCTGTTACGGGGATAATTTCAGGCATCCTTGAGGCTTCAAAACAGGCAATGTTTGAACAT
GTCGCCAGTAAAATGGCCGATGTTATTGCTGAATGGGAGAAAAAACACGGCAAAAATTAC
TTTGAAAATGGATATGATGCCCGCCATGCTGCATTTTTAGAAGATAACTTTAAAATATTA
TCTCAGTATAATAAAGAGTATTCTGTTGAAAGATCAGTCCTCATTACCCAGCAACATTGG
GATACGCTGATAGGTGAGTTAGCGGGTGTCACCAGAAATGGAGACAAAACACTCAGTGGT
AAAAGTTATATTGACTATTATGAAGAAGGAAAACGTCTGGAGAAAAAACCGGATGAATTC
CAGAAGCAAGTCTTTGACCCATTGAAAGGAAATATTGACCTTTCTGACAGCAAATCTTCT
ACGTTATTGAAATTTGTTACGCCATTGTTAACTCCCGGTGAGGAAATTCGTGAAAGGAGG
CAGTCCGGAAAATATGAATATATTACCGAGTTATTAGTCAAGGGTGTTGATAAATGGACG
GTGAAGGGGGTTCAGGACAAGGGGTCTGTATATGATTACTCTAACCTGATTCAGCATGCA
TCAGTCGGTAATAACCAGTATCGGGAAATTCGTATTGAGTCACACCTGGGAGACGGGGAT
GATAAGGTCTTTTTATCTGCCGGCTCAGCCAATATCTACGCAGGTAAAGGACATGATGTT
GTTTATTATGATAAAACAGACACCGGTTATCTGACCATTGATGGCACAAAAGCAACCGAA
GCGGGTAATTACACGGTAACACGTGTACTTGGTGGTGATGTTAAGATTTTACAGGAAGTT
GTGAAGGAGCAGGAGGTTTCAGTTGGAAAAAGAACTGAAAAAACGCAATATCGGAGTTA
T
GAATTCACTCATATCAATGGTAAAAATTTAACAGAGACTGATAACTTATATTCCGTGGAA
GAACTTATTGGGACCACGCGTGCCGACAAGTTTTTTGGCAGTAAATTTACTGATATCTTC
CATGGCGCGGATGGTGATGACCATATAGAAGGAAATGATGGGAATGACCGCTTATATGGT
GATAAAGGTAATGATACGCTGAGGGGCGGAAACGGGGATGACCAGCTCTATGGCGGTGAT
GGCAATGATAAGTTAATTGGGGGGACAGGTAATAATTACCTTAACGGCGGTGACGGAGAT
GATGAGCTTCAGGTTCAGGGGAATTCTCTTGCTAAAAATGTATTATCCGGTGGAAAAGGT
AATGACAAGTTGTACGGCAGTGAGGGAGCAGATCTGCTTGATGGCGGAGAAGGGAATGAT
CTTCTGAAAGGTGGATATGGTAATGATATTTATCGTTATCTTTCAGGATATGGCCATCAT
ATTATTGACGATGATGGGGGGAAAGACGATAAACTCAGTTTGGCTGATATTGATTTCCGG
GATGTGGCCTTCAGGCGAGAAGGTAATGACCTCATCATGTATAAAGCTGAAGGTAATGTT
CTTTCCATTGGTCATAAAAATGGTATTACATTCAGGAACTGGTTTGAAAAAGAGTCAGGT
GATATCTCTAATCACCAGATAGAGCAGATTTTTGATAAAGACGGCAGGGTAATCACACCA
GATTCCCTTAAAAAGGCACTTGAGTATCAACAGAGTAATAATAAGGCAAGTTATGTGTAT
GGGAATGATGCATTAGCCTATGGAAGTCAGGATAATCTTAATCCATTAATTAATGAAATC
AGCAAAAATCATTTCAGCTGCAGGTAATTTTGATGTTAAAGAGGAAAGAGCTGCAGCTTCT
TTATTGCAGTTGTCCGGTAATGCCAGTGATTTTTCATATGGACGGAACTCAATAACTTTG
ACAGCATCAGCATAA
>ihaA__STEC__Iron_regulated_adhesin__O157_Sakai

TCAGAACTGATAGTTCAGCGACATCCAGTAATTTCGCTCAGGTATCACATATCCTGTTGT
TGATGATCCCGTCTGGAAGTAATCACCGGCATACAGCGTACTCTTACCGGCACTGTACAG
GCTCACGTCACTGTAATCCTTGTTGAGCAGGTTATTCACCGCAGCATTCAGCGTCAGGGC
ATCCGTCATCTTCCACGACAGACCTGCATCCACCACCGTCCAGGCTTTCAGGTATTCTCC
TTTCTCATCATACACTTTCTTCTGTACAGCGCTCAGTGACGAATAATTCTGGGTGAAACG
TGGTGTTTTCCCGCGATAACGGGCACCCAGCCATGATGCCACCTCTTCGGTGATCTGCCA
GTTCAGTTTCGCATTCACCATGTGTTCAGGGGTATAACTCAGCGGCGCACCTTTGTTATC
ACCATCACGTTGTTCACTTCGGGTCCAGGTGTAATTCAGTGACAGCGTGACATCCTCTGA
CCACAGCGGCAATGTGCCGGCAAATTCCACACCGTGCAACCGGGCCTTTCCGCTGTTTAC
ATAGCTATTGGTGTTATCATTTATGGAATAAGAGACAATCTTGTTGGAGAAGTCAGTCAT
AAAACCTGTGACATTGGCATTTAGACCGGCGGGGTTATCGTAATACACCCCAGCCTCATA
ACTGACGCTCTCTTCCGGCTTCAGGTCGGGGTTACCAAGTAGATTTGTTTTTCCCTGCCC
GGACACACCACTAATCCCTTTATGTAGCTGCCCCATTCTGGGTGCCTTATATCCCGTGGT
CACACCGCCTTTCAGCGTCCAGGCATCTGCCACATCCCAGACCAGATATGCACGCGGACT
GAAGTGTCCCCCGAATTGCTCATGATGTTCATAGCGGCTGCCCGCAGTCAGCGCAAGTGC
ATCCGTGAGATGCCACTCATCCTCAGCAAATACCGACCAGCTTTTCTGCCGGAAAGTTTC
ACCTGTGCTGGCAAGGACAACTCCGTCTTTCATGGACGAGCTCTGAAACTCGCCCCCCAC
CGTAACCAGATGAGATTCTCCCAGAGGGGTAAGCAGTAATGAATTCAGGATAAGGTTCGA
TTCCTTAAGCTCCCGCGGCTGACCGGCAAGCCCCCATTTGTCGCGCTTCAGTACACTGCG
TACAAGCTCACGACCTTTATTTTCTGTCTCGTTCCAGTTCAGATACGATTTCCATGTTCC
GAAGGTGAAAGTATGATCATAGCCAGCTGAAATTTTGTTTCGCTCATAGCGCAGGGTCCG
GTCATATCCCCCCGTCAGACTCCCCAGTTGCCCATCCCGGTTATCATAACGCTGCCGGGT
GGTATCCATATCAAACCAGAGCACATCCTGCTCCGACGCCTTCCAGTCAAGACGTGCACC
AAGATTATAATTCTGTGACTCCGTGGGATAAGGAATACGCGTGGCTGCTGTATCGCTCAG
TGATGTGACCGATGAACCCTGACGCTGTTGTGTGCTACCGCGTACCTGCAGGCTGACAGA
ATCATCCACAAGGGGACCACTGCTCCAGAAATTAAACTGGCTGCTGTTACCCCATTTGTT
GCTTTCCTGCAGATTCAGCCCTGCATTGACGGAAGAGAGCCATTTGTCTGCATTCTTTCT
GGTAATGATATTCACCACACCGCCCATCGCATCAGAGCCATACAGTGTGGACATCGGCCC
CCTGATAACCTCAATACGCTCAATGGCGGCCAGAGGGGGCATGAACCCGGTATTCATGGC
AGAAAAACCGTTGGGAGTCACGTCACTGCTTCCGCCCTGACGAACACCATCAATCAGTAT
CAGCGTGTAACTGGCTGGCATTCCTCGGATGCTGATTTCCAGCCCTCCGGTTTTACCCGT
ACCACTTTCAACATCCACACCCTCTACTGATCTCAGAGCCTCCGCCAGATCGTGGTACTG
GCTGGACTGCAATTCCTCCTGGCTAATCACAGAAACACTGGCGGCTGCGTTAGTCAGCTT
TTTCTCATAGCCGGATGCCGAGACAATCATCACATCCTCTGCAGCAGCTATGCTGCTGGC
TGAAAATCCGAGACAGGGAATGACTACGGAAGCCAGAGTGGTTATTCGCAT
>ihaA__STEC__Iron_regulated_adhesin__O26_11368
ATGCGAATAACCACTCTGGCTTCCGTAGTCATTCCCTGTCTCGGATTTTCAGCCAGCAGC
ATAGCTGCTGCAGAGGATGTGATGATTGTCTCGGCATCCGGCTATGAGAAAAGCTGACT
AACGCAGCCGCCAGTGTTTCTGTGATTAGCCAGGAGGAATTGCAGTCCAGCCAGTACCAC
GATCTGGCGGAGGCTCTGAGATCAGTAGAGGGTGTGGATGTTGAAAGTGGTACGGGTAAA
ACCGGAGGGCTGGAAATCAGCATCCGAGGAATGCCAGCCAGTTACACGCTGATACTGATT
GATGGTGTTCGTCAGGGCGGAAGCAGTGACGTGACTCCCAACGGTTTTTCTGCCATGAAT
ACCGGGTTCATGCCCCCTCTGGCCGCCATTGAGCGTATTGAGGTTATCAGGGGGCCGATG
TCCACACTGTATGGCTCTGATGCGATGGGCGGTGTGGTGAATATCATTACCAGAAAGAAT
GCAGACAAATGGCTCTCTTCCGTCAATGCAGGGCTGAATCTGCAGGAAAGCAACAAATGG
GGTAACAGCAGCCAGTTTAATTTCTGGAGCAGTGGTCCCCTTGTGGATGATTCTGTCAGC
CTGCAGGTACGCGGTAGCACACAACAGCGTCAGGGTTCATCGGTCACATCACTGAGCGAT
ACAGCAGCCACGCGTATTCCTTATCCCACGGAGTCACAGAATTATAATCTTGGTGCACGT
CTTGACTGGAAGGCGTCGGAGCAGGATGTGCTCTGGTTTGATATGGATACCACCCGGCAG
CGTTATGATAACCGGGATGGGCAACTGGGGAGTCTGACGGGGGGATATGACCGGACCCTG
CGCTATGAGCGAAACAAAATTTCAGCTGGCTATGATCATACTTTCACCTTCGGAACATGG
AAATCGTATCTGAACTGGAACGAGACAGAAAATAAAGGTCGTGAGCTTGTACGCAGTGTA
CTGAAGCGCGACAAATGGGGGCTTGCCGGTCAGCCGCGGGAGCTTAAGGAATCGAACCT
T
ATCCTGAATTCATTACTGCTTACCCCTCTGGGAGAATCTCATCTGGTTACGGTGGGGGGC
GAGTTTCAGAGCTCGTCCATGAAAGACGGAGTTGTCCTTGCCAGCACAGGTGAAACTTTC
CGGCAGAAAAGCTGGTCGGTATTTGCTGAGGATGAGTGGCATCTCACGGATGCACTTGCG

CTGACTGCGGGCAGCCGCTATGAACATCATGAGCAATTCGGGGGACACTTCAGTCCGCGT
GCATATCTGGTCTGGGATGTGGCAGATGCCTGGACGCTGAAAGGCGGTGTGACCACGGGA
TATAAGGCACCCAGAATGGGGCAGCTACATAAAGGGATTAGTGGTGTGTCCGGGCAGGGA
AAAACAAATCTACTTGGTAACCCCGACCTGAAGCCGGAAGAGAGCGTCAGTTATGAGGCT
GGGGTGTATTACGATAACCCCGCCGGTCTGAATGCCAATGTCACAGGTTTTATGACTGAC
TTCTCCAACAAGATTGTCTCTTATTCCATAAATGATAACACCAATAGCTATGTAAACAGC
GGAAAGGCCCGGTTGCACGGTGTGGAATTTGCCGGCACATTGCCGCTGTGGTCAGAGGAT
GTCACGCTGTCACTGAATTACACCTGGACCCGAAGTGAACAACGTGATGGTGATAACAAA
GGTGCGCCGCTGAGTTATACCCCTGAACACATGGTGAATGCGAAACTGAACTGGCAGATC
ACCGAAGAGGTGGCATCATGGCTGGGTGCCCGTTATCGCGGGAAAACACCACGTTTCACC
CAGAATTATTCGTCACTGAGCGCTGTACAGAAGAAAGTGTATGATGAGAAAGGAGAATAC
CTGAAAGCCTGGACGGTGGTGGATGCAGGTCTGTCGTGGAAGATGACGGATGCCCTGACG
CTGAATGCTGCGGTGAATAACCTGCTCAACAAGGATTACAGTGACGTGAGCCTGTACAGT
GCCGGTAAGAGTACGCTGTATGCCGGTGATTACTTCCAGACGGGATCATCAACAACAGGA
TATGTGATACCTGAGCGAAATTACTGGATGTCGCTGAACTATCAGTTCTGA
>ihaA__STEC__Iron_regulated_adhesin__O111_11128
ATGCGAATAACCACTCTGGCTTCCGTAGTCATTCCCTGTCTCGGATTTTCAGCCAGCAGC
ATAGCTGCTGCAGAGGATGTGATGATTGTCTCGGCATCCGGCTATGAGAAAAAGCTGACT
AACGCAGCCGCCAGTGTTTCTGTGATTAGCCAGGAGGAATTGCAGTCCAGCCAGTACCAC
GATCTGGCGGAGGCTCTGAGATCAGTAGAGGGTGTGGATGTTGAAAGTGGTACGGGTAAA
ACCGGAGGGCTGGAAATCAGCATCCGAGGAATGCCAGCCAGTTACACGCTGATACTGATT
GATGGTGTTCGTCAGGGCGGAAGCAGTGACGTGACTCCCAACGGTTTTTCTGCCATGAAT
ACCGGGTTCATGCCCCCTCTGGCCGCCATTGAGCGTATTGAGGTTATCAGGGGGCCGATG
TCCACACTGTATGGCTCTGATGCGATGGGCGGTGTGGTGAATATCATTACCAGAAAGAAT
GCAGACAAATGGCTCTCTTCCGTCAATGCAGGGCTGAATCTGCAGGAAAGCAACAAATGG
GGTAACAGCAGCCAGTTTAATTTCTGGAGCAGTGGTCCCCTTGTGGATGATTCTGTCAGC
CTGCAGGTACGCGGTAGCACACAACAGCGTCAGGGTTCATCGGTCACATCACTGAGCGAT
ACAGCAGCCACGCGTATTCCTTATCCCACGGAGTCACAGAATTATAATCTTGGTGCACGT
CTTGACTGGAAGGCGTCGGAGCAGGATGTGCTCTGGTTTGATATGGATACCACCCGGCAG
CGTTATGATAACCGGGATGGGCAACTGGGGAGTCTGACGGGGGGATATGACCGGACCCTG
CGCTATGAGCGAAACAAAATTTCAGCTGGCTATGATCATACTTTCACCTTCGGAACATGG
AAATCGTATCTGAACTGGAACGAGACAGAAAATAAAGGTCGTGAGCTTGTACGCAGTGTA
CTGAAGCGCGACAAATGGGGGCTTGCCGGTCAGCCGCGGGAGCTTAAGGAATCGAACCT
T
ATCCTGAATTCATTACTGCTTACCCCTCTGGGAGAATCTCATCTGGTTACGGTGGGGGGC
GAGTTTCAGAGCTCGTCCATGAAAGACGGAGTTGTCCTTGCCAGCACAGGTGAAACTTTC
CGGCAGAAAAGCTGGTCGGTATTTGCTGAGGATGAGTGGCATCTCACGGATGCACTTGCG
CTGACTGCGGGCAGCCGCTATGAACATCATGAGCAATTCGGGGGACACTTCAGTCCGCGT
GCATATCTGGTCTGGGATGTGGCAGATGCCTGGACGCTGAAAGGCGGTGTGACCACGGGA
TATAAGGCACCCAGAATGGGGCAGCTACATAAAGGGATTAGTGGTGTGTCCGGGCAGGGA
AAAACAAATCTACTTGGTAACCCCGACCTGAAGCCGGAAGAGAGCGTCAGTTATGAGGCT
GGGGTGTATTACGATAACCCCGCCGGTCTGAATGCCAATGTCACAGGTTTTATGACTGAC
TTCTCCAACAAGATTGTCTCTTATTCCATAAATGATAACACCAATAGCTATGTAAACAGC
GGAAAGGCCCGGTTGCACGGTGTGGAATTTGCCGGCACATTGCCGCTGTGGTCAGAGGAT
GTCACGCTGTCACTGAATTACACCTGGACCCGAAGTGAACAACGTGATGGTGATAACAAA
GGTGCGCCGCTGAGTTATACCCCTGAACACATGGTGAATGCGAAACTGAACTGGCAGATC
ACCGAAGAGGTGGCATCATGGCTGGGTGCCCGTTATCGCGGGAAAACACCACGTTTCACC
CAGAATTATTCGTCACTGAGCGCTGTACAGAAGAAAGTGTATGATGAGAAAGGAGAATAC
CTGAAAGCCTGGACGGTGGTGGATGCAGGTCTGTCGTGGAAGATGACGGATGCCCTGACG
CTGAATGCTGCGGTGAATAACCTGCTCAACAAGGATTACAGTGACGTGAGCCTGTACAGT
GCCGGTAAGAGTACGCTGTATGCCGGTGATTACTTCCAGACGGGATCATCAACAACAGGA
TATGTGATACCTGAGCGAAATTACTGGATGTCGCTGAACTATCAGTTCTGA
>invE__EIEC__regulator__Shigella_flexneri_5a_str._M90T
TTATGAAGACGATAGATGGCGAGAAATTATATCCCGAATAGCTTCATCTATTTCTCTCTG
AACTGTTTTGTTTATTTTAGATAATGTAAATATTAGAGTTCTGTTTGTTTTATTCTCTTT
TCTTTTTATGTAAGTCCGTTTGTCTTTAGAAATAAATAACGAGTCAACTTTTAGCGAAGG
GTTTTGCTTTCTGTTTTTGCTTTTCTTTATGATGTTTAAAATTTCTTTTTTATATATGTC

CGGGGGCAGATTTGTATCAAGGTCTTTTATTTCTTCCTTTAATATTGGTAGTGTAGAACT
AAGAGATTCATTAGCCTTTTCAAGTCCTTTATAATAATTGAATAATATCTTGTAGTCATT
AAAGTTAAGTTCTGACGCGATTGGAAATAGAGATATTATTTCCTGTGGAACGCTTGCTGC
CTGAAAGGCACGAGTGACTTTCGCGCGAGACAGATTCTCTTTTTTGGCTATGTCTTTATA
GGACATCCCTGATACTTTCAGAAAATTAAGACCAATACCAAGTTCTCGGATGCTATGCTC
TTTTGCTGTTTGTATATCGTTTGCTAGTTTTCTGGCATCAAGAGTAGATATATATTCTTT
TGAATATAGAACTTCAAGATCTGCTCCTGCATATATTGCAGATGCTCTTCTACGAGTGCC
ATCCAGAATTTCAATTCTACCATCAATCTCCCTTCCTATTACAGGGAAGAATTGTTGTAG
CTTTATAGTTTTTATGATATCGGCTAGTGATTCTTCTGTTAGGGATTCTTGATCGCGTAC
ATTCGTTTTTTGGTTGACGAAGGTTAAATCTTGGATCCTTTTATGAGGAATCTTGGCTTT
GATAAAGGAAACTTTATTACCAGAATGGAGTGTAAACTCTTTCTTTTGGCCTTCCTTTAT
ACTTAACAAGTCGTTGCACAAATCCACCAT
>ipaH__EIEC__incasive_plasmid_antigen__FORC_011
CATGGCTGGAAAAACTCAGTGCCTCTGCGGAGCTTCGACAGCAGTCTTTCGCTGTTGCTG
CTGATGCCACTGAGAGCTGTGAGGACCGTGTCGCGCTCACATGGAACAATCTCCGGAAAA
CCCTCCTGGTCCATCAGGCATCAGAAGGCCTTTTCGATAATGATACCGGCGCTCTGCTCT
CCCTGGGCAGGGAAATGTTCCGCCTCGAAATTCTGGAGGACATTGCCCGGGATAAAGTCA
GAACTCTCCATTTTGTGGATGAGATAGAAGTCTACCTGGCCTTCCAGACCATGCTCGCAG
AGAAACTTCAGCTCTCCACTGCCGTGAAGGAAATGCGTTTCTATGGCGTGTCGGGAGTGA
CAGCAAATGACCTCCGCACTGCCGAAGCCATGGTCAGAAGCCGTGAAGAGAATGAATTTA
CGGACTGGTTCTCCCTCTGGGGACCATGGCATGCTGTACTGAAGCGTACGGAAGCTGACC
GCTGGGCGCT
>katP__STEC__katalase_peroxidase__O157_Sakai
ATGATAAAAAAAACTCTTCCTGTTCTGATTCTTCTGGCGCTATCGGGGAGCTTTTCTACC
GCTGTAGCCGCTGATAAAAAAGAGACTCAAAATTTCTACTATCCAGAAACACTGGATTTA
ACTCCTCTGAGATTACACAGCCCTGAATCAAATCCCTGGGGGGCTGATTTTGATTATGCC
ACCAGATTTCAACAGCTGGATATGGAGGCTCTGAAAAAAGATATCAAAGATTTGCTGACA
ACTTCCCAGGATTGGTGGCCTGCGGATTATGGTCATTATGGTCCTTTCTTTATTCGTATG
GCTTGGCACGGTGCCGGAACATACAGGACATATGATGGCCGGGGAGGCGCCAGTGGTGGT
CAGCAACGTTTTGAACCGCTGAACAGCTGGCCGGATAACGTTAATCTGGATAAAGCCCGT
CGATTGCTGTGGCCAGTCAAGAAAAAATACGGCTCCAGTATTTCCTGGGGAGACCTGATG
GTCCTGACTGGTAATGTTGCCCTTGAATCCATGGGATTTAAAACGCTGGGATTTGCTGGC
GGAAGAGAAGATGACTGGGAGTCGGACCTGGTATACTGGGGGCCTGACAACAAGCCTCT
T
GCAGATAACCGGGATAAAAACGGGAAACTTCAGAAACCTCTTGCCGCCACGCAGATGGG
A
CTTATTTATGTCAATCCTGAAGGCCCCGGTGGAAAACCAGATCCTCTGGCTTCCGCGAAA
GATATCAGGGAAGCTTTTTCACGTATGGCCATGGATGATGAGGAGACTGTGGCCCTGATC
GCGGGAGGGCATACATTTGGTAAAGCACATGGTGCAGCGTCTCCTGAAAAATGTATTGGC
GCAGGGCCTGATGGTGCACCTGTGGAGGAGCAGGGACTGGGATGGAAAAATAAATGTGG
T
ACAGGAAACGGCAAATATACCATCACCAGTGGCCTGGAAGGAGCCTGGTCGACATCGCCA
ACCCAGTTCACAATGCAGTATCTGAAGAATTTATATAAATATGAATGGGAGCTGCACAAG
AGTCCTGCCGGTGCTTATCAGTGGAAGCCTAAAAAAGCGGCAAATATAGTTCAGGACGCG
CATGATCCGTCTGTCCTGCATCCGTTGATGATGTTTACGACGGATATTGCTCTTAAAGTT
GATCCTGAATATAAGAAAATAACCACCCGTTTCCTGAATGATCCAAAAGCTTTTGAGCAG
GCATTCGCAAGAGCATGGTTTAAACTGACCCACCGGGATATGGGACCGGCAGCCCGATAT
CTTGGTAATGAAGTTCCTGCAGAATCATTTATCTGGCAGGATCCTCTTCCTGCGGCGGAT
TATACAATGATTGATGGTAAAGACATTAAGTCGCTGAAAGAGCAGGTTATGGATTTGGGT
ATCCCTGCATCTGAGCTGATAAAGACAGCCTGGGCTTCAGCTTCCACATTTCGTGTGACT
GATTATCGTGGGGGAAATAATGGTGCCCGCATCAGGTTACAGCCCGAAATTAACTGGGAA
GTTAATGAGCCTGAAAAACTGAAGAAAGTACTGGCATCCCTGACCTCATTACAGCGTGAA
TTTAACAAAAAACAGTCTGACGGAAAGAAAGTGTCGTTGGCTGATTTAATTGTTCTTTCG
GGTAATGCTGCAATCGAAGATGCGGCCAGAAAAGCCGGGGTGGAACTTGAGATTCCCTTT
ACTCCGGGAAGAACTGACGCCTCTCAGGAGCAGACGGATGTTGCCTCATTCAGTGTACTG
GAGCCGACAGCAGATGGATTCAGAAATTATTACTCAAAAAGCAGAAGTCATATATCGCCG
GTTGAAAGCCTCATTGATAAAGCCAGTCAGCTGGATCTCACCGTTCCTGAAATGACGGCA

TTACTGGGTGGTCTGCGGGTAATGGATATTAATACAAATAATTCTTCGTTGGGAGTGTTT
ACCGATACCCCTGGTGTTCTGGATAACAAGTTTTTTGTTAATCTGCTGGATATGTCAACA
CGATGGAGTAAAGCAGATAAAGAAGATACATACAATGGATTCGATCGTAAAACGGGAGCA
TTAAAATGGAAAGCATCCTCTGTTGATTTAATCTTCAGTTCAAATCCTGAATTACGTGCG
GTGGCAGAAGTATATGCCTCGGATGATGCGAGAAATAAGTTTATTCATGATTTTGTTAAA
TCGTGGAATAAAGTTATGAATAGCGATCGGTTTGATTTAAACAATAAATGA
>katP__STEC__katalase_peroxidase__O26_11368
ATGATAAAAAAAACTCTTCCTGTTCTGATTCTTCTGGCGCTATCGGGGAGCTTTTCTACC
GCTGTAGCCGCTGATAAAAAAGAGACTCAAAATTTCTACTATCCAGAAACACTGGATTTA
ACTCCTCTGAGATTACACAGCCCTGAATCAAATCCCTGGGGGGCTGATTTTGATTATGCC
ACCAGATTTCAACAGCTGGATATGGAGGCTCTGAAAAAAGATATCAAAGATTTGCTGACA
ACTTCCCAGGATTGGTGGCCTGCGGATTATGGTCATTATGGTCCTTTCTTTATTCGTATG
GCTTGGCACGGTGCCGGAACATACAGGACATATGATGGCCGGGGAGGCGCCAGTGGTGGT
CAGCAACGTTTTGAACCGCTGAACAGCTGGCCGGATAACGTTAATCTGGATAAAGCCCGT
CGATTGCTGTGGCCAGTCAAGAAAAAATACGGCTCCAGTATTTCCTGGGGAGACCTGATG
GTCCTGACTGGTAATGTTGCCCTTGAATCCATGGGATTTAAAACGCTGGGATTTGCTGGC
GGAAGAGAAGATGACTGGGAGTCGGACCTGGTATACTGGGGGCCTGACAACAAGCCTCT
T
GCAGATAACCGGGATAAAAACGGGAAACTTCAGAAACCTCTTGCCGCCACGCAGATGGG
A
CTTATTTATGTCAATCCTGAAGGCCCCGGTGGAAAACCAGATCCTCTGGCTTCCGCGAAA
GATATCAGGGAAGCTTTTTCACGTATGGCCATGGATGATGAGGAGACTGTGGCCCTGATC
GCGGGAGGGCATACATTTGGTAAAGCACATGGTGCAGCGTCTCCTGAAAAATGTATTGGC
GCAGGGCCTGATGGTGCACCTGTGGAGGAGCAGGGACTGGGATGGAAAAATAAATGTGG
T
ACAGGAAACGGCAAATATACCATCACCAGTGGCCTGGAAGGAGCCTGGTCGACATCGCCA
ACCCAGTTCACAATGCAGTATCTGAAGAATTTATATAAATATGAATGGGAGCTGCACAAG
AGTCCTGCCGGTGCTTATCAGTGGAAGCCTAAAAAAGCGGCAAATATAGTTCAGGACGCG
CATGATCCGTCTGTCCTGCATCCGTTGATGATGTTTACGACGGATATTGCTCTTAAAGTT
GATCCTGAATATAAGAAAATAACCACCCGTTTCCTGAATGATCCAAAAGCTTTTGAGCAG
GCATTCGCAAGAGCATGGTTTAAACTGACCCACCGGGATATGGGACCGGCAGCCCGATAT
CTTGGTAATGAAGTTCCTGCAGAATCATTTATCTGGCAGGATCCTCTTCCTGCGGCGGAT
TATACAATGATTGATGGTAAAGACATTAAGTCGCTGAAAGAGCAGGTTATGGATTTGGGT
ATCCCTGCATCTGAGCTGATAAAGACAGCCTGGGCTTCAGCTTCCACATTTCGTGTGACT
GATTATCGTGGGGGAAATAATGGTGCCCGCATCAGGTTACAGCCCGAAATTAACTGGGAA
GTTAATGAGCCTGAAAAACTGAAGAAAGTACTGGCATCCCTGACCTCATTACAGCGTGAA
TTTAACAAAAAACAGTCTGACGGAAAGAAAGTGTCGTTGGCTGATTTAATTGTTCTTTCG
GGTAATGCTGCAATCGAAGATGCGGCCAGAAAAGCCGGGGTGGAACTTGAGATTCCCTTT
ACTCCGGGAAGAACTGACGCCTCTCAGGAGCAGACGGATGTTGCCTCATTCAGTGTACTG
GAGCCGACAGCAGATGGATTCAGAAATTATTACTCAAAAAGCAGAAGTCATATATCGCCG
GTTGAAAGCCTCATTGATAAAGCCAGTCAGCTGGATCTCACCGTTCCTGAAATGACGGCA
TTACTGGGTGGTCTGCGGGTAATGGATATTAATACAAATAATTCTTCGTTGGGAGTGTTT
ACCGATACCCCTGGTGTTCTGGATAACAAGTTTTTTGTTAATCTGCTGGATATGTCAACA
CGATGGAGTAAAGCAGATAAAGAAGATACATACAATGGATTCGATCGTAAAACGGGAGCA
TTAAAATGGAAAGCATCCTCTGTTGATTTAATCTTCAGTTCAAATCCTGAATTACGTGCG
GTGGCAGAAGTATATGCCTCGGATGATGCGAGAAATAAGTTTATTCATGATTTTGTTAAA
TCGTGGAATAAAGTTATGAATAGCGATCGGTTTGATTTAAACAATAAATGA
>katP__clade1__katalase_peroxidase__10290
ATGATAAAAAAAACTCTTCCTGTTCTGATTCTTCTGGCGCTATCGGGGAGTTTTTCTACC
GCTGTAGCCGCTGATAAAAAAGAGACTCAAAATTTCTACTATCCAGAAACACTGGATTTA
ACTCCTCTGAGATTACACAGCCCTGAATCAAATCCCTGGGGGGCTGATTTTGATTATGCC
ACCAGATTTCAACAGCTGGATATGGAGGCTCTGAAAAAAGATATCAAAGATTTGCTGACA
ACTTCCCAGGATTGGTGGCCTGCGGATTATGGTCATTATGGTCCTTTCTTTATTCGTATG
GCTTGGCACGGTGCCGGAACATACAGGACATATGATGGCCGGGGAGGCGCCAGTGGTGGT
CAGCAACGTTTTGAACCGCTGAACAGCTGGCCGGATAACGTTAATCTGGATAAAGCCCGT
CGATTGCTGTGGCCAGTCAAGAAAAAATACGGCTCCAGTATTTCCTGGGGAGACCTGATG
GTCCTGACTGGTAATGTTGCCCTTGAATCCATGGGATTTAAAACGCTGGGATTTGCTGGC

GGAAGAGAAGATGACTGGGAGTCGGACCTGGTATACTGGGGGCCTGACAACAAGCCTCT
T
GCAGATAACCGGGATAAAAACGGGAAACTTCAGAAACCTCTTGCCGCCACGCAGATGGG
A
CTTATTTATGTCAATCCTGAAGGCCCCGGTGGAAAACCAGATCCTCTGGCTTCCGCGAAA
GATATCAGGGAAGCTTTTTCACGTATGGCCATGGATGATGAGGAGACTGTGGCCCTGATC
GCGGGAGGGCATACATTTGGTAAAGCACATGGTGCAGCGTCTCCTGAAAAATGTATTGGC
GCAGGGCCTGATGGTGCACCTGTGGAGGAGCAGGGACTGGGATGGAAAAATAAATGTGG
T
ACAGGAAACGGCAAATATACCATCACCAGTGGCCTGGAAGGAGCCTGGTCAACATCGCCA
ACCCAGTTCACAATGCAGTATCTGAAGAATTTATATAAATATGAATGGGAGCTGCACAAG
AGTCCTGCCGGTGCTTATCAGTGGAAGCCTAAAAAAGCGTCAAATATAGTTCAGGACGCG
CATGATCCGTCTGTCCTGCATCCGTTGATGATGTTTACGACGGATATTGCTCTTAAAGTT
GATCCTGAATATAAGAAAATAACCACCCGTTTCCTGAATGATCCAAAAGCTTTTGAGCAG
GCATTCGCAAGAGCATGGTTTAAACTGACCCACCGGGATATGGGACCGGCAGCCCGATAT
CTTGGTAATGAAGTTCCTGCAGAATCATTTATCTGGCAGGATCCTCTTCCTGCGGCGGAT
TATACAATGATTGATGGTAAAGACATTAAGTCGCTGAAAGAGCAGGTTATGGATTTGGGT
ATCCCTGCATCTGAGCTGATAAAGACAGCCTGGGCTTCAGCTTCCACATTTCGTGTGACT
GATTATCGTGGGGGAAATAATGGTGCCCGCATCAGGTTACAGCCCGAAATTAACTGGGAA
GTTAATGAGCCTGAAAAACTGAAGAAAGTACTGGCATCCCTGACCTCATTACAGCGTGAA
TTTAACAAAAAACAGTCTGACGGAAAGAAAGTGTCGTTGGCTGATTTAATTGTTCTTTCG
GGTAATGCTGCAATCGAAGATGCGGCCAGAAAAGCCGGGGTGGAACTTGAGATTCCCTTT
ACTCCGGGAAGAACTGACGCCTCTCAGGAGCAGACGGATGTTGCCTCATTCAGTGTACTG
GAGCCGACAGCAGATGGATTCAGAAATTATTACTCAAAAAGCAGAAGTCATATATCGCCG
GTTGAAAGCCTCATTGATAAAGCCAGTCAGCTGGATCTCACCGTTCCTGAAATGACGGCA
TTACTGGGTGGTCTGCGGGTAATGGATATTAATACAAATAATTCTTCGTTGGGAGTATTT
ACCGATACCCCTGGTGTTCTGGATAACAAGTTTTTTGTTAATCTGCTGGATATGTCAACA
CGATGGAGTAAAGCAGATAAAGAAGATACATACAATGGATTCGATCGTAAAACGGGAGCA
TTAAAATGGAAAGCATCCTCTGTTGATTTAATATTCAGTTCAAATCCTGAATTACGTGCG
GTGGCAGAAGTATATGCCTCGGATGATGCGAGAAATAAGTTTATTCATGATTTTGTTAAA
TCGTGGAATAAAGTTATGAATAGCGATCGGTTTGATTTAAACAATAAATGA
>lpxR__STEC__lipid_A_3'-O-deacylase__O157_Sakai
ATGAAAAAAAGTGTCATCGCTGGCGTCTTTATTGCTCTGTCATTTACCACGTGTTCAGCT
ATCGCGAACAGCCTTGCATTATCATTAGCAAATGATGATGCAGGGAAGTTTCAACCAATA
CTTAATGATATTTATGGCAATAAACATGAAAACAGAGATGATTACTCACAAGGCTTATTT
CTGGGATATAGCCACGATATCTCAGACTCGAGCCAATTATCTCTCCATATTGCGCAAGAT
ATTTACTCTCCATCAGGCAGTAATAAAAGACACAACACAGCTGTAACTGGAGACAGAGCT
TTTAGTGCATACACTCACACTGGTATTGAATGGAACTCCCTTGCGAATGACTGGATTCGC
TATCGATTAGGTACTGACATAGGTGTTGTTGGCCCCGACGCAGGCGGTCAGAAAGTACAA
AATAAAGCTCATGAGATTATTGGGGCAGAAAAATATCATGCATGGGATGATCAAATAGAG
AATCGCTACGGTTATACTGTAAAAGGGATGCTATCCATGACACCAAGTATGGATATTTTA
GGTGCTAATGTTGGATTATACCCTGAAGTTTCTGCTGTTACTGGAAACTTATTTCAATAT
GTAGCATATGGCGCAACCATTGCCATTGGTAATGATAAAACCTTCAATTCGGATAATGGC
TTTGGTCTGCTGGCTCCCCGTGGTTTAATGCATATGTCCGATACAAGCGGATTCAAATAC
AAGATTTTTGCAGGTATGGAAAGACGAGATGTCAATCGCAACTATACTCTCGAAGGAAAA
ACAATACAGACGAAACAAACAACAGTATCGCTAAACAAAACTGTTGATGAATATCAAGTT
GGCGCAACAATTGGGTATGCACCTGTAGCCTTCACACTAGCATTTAATAAAGTAACATCA
GAATTCAAGACAGGGGATGACTATTCATTTATAAATGGAGCAATCACCTTCTTTTTTTAA
>paa__STEC__adhesin__O157_Sakai
ATGAGGAACATAATGGCAGGTTTTTTAATATTCCTGTCTTCTGCTGCTTATGCTGATATC
AATCTGTATGGTCCTGGTGGCCCGCATACAGCCTTGCTTGATGCAGCCAAACTTTATGCC
GAAAAAACAGGTATTATAGTGAACGTTCATTACGGCCCACAGAACAAATGGAATGAAGAT
GCCAAAAAAAATGCAGATATCTTGTTTGGCGCATCAGAACAATCTGCTCTGGCTATCATT
CGGGACCATAAAGACAGCTTCAGTGAAAAAGATATTCAGCCTCTTTATCTGCGAAAAAGT
ATTTTACTGGTAAAGAAAGGTAATCCTAAAAATATCCGGAGTATTGACGACCTGACCAGA
CCTGGGATTGGCGTAATTGTTAATGATGGTGGTGGTACCAGTAATACATCAGGCACTGGC
GTCTGGGAAGATATTGCCGGACGTAAAGGGAATATAGAAACTGTCGCCGCAATCCGAAAA

AATATTATTTTATATGCGCCCAATAGCGGAACTGCACGTAAGGCTCTTGAGAATCAGCCT
GGAGCAGATGTCTGGATAACCTGGGCTGACTGGGCAGCCAGTAATCCAGAAATTGGTGAT
GTCGTGGAAATAGCGCCAGACTACGTGATATGGCGTGATATGAACATTACAGTACGTCAG
GATGCAAATGATGAAACCCGTCGATTTGCAGAATGGCTACAAACCGATGAAGCGGCGCCT
GCATTCAAAAAATATGGCTGGACCAGGAAAGGCACTTGA
>paa__STEC__adhesin__O26_11368
ATGGCAGGTTTTTTAATATTCCTGTCTTCTGCTGCTTATGCTGATATCAATCTGTATGGT
CCTGGTGGCCCGCATACAGCCTTGCTTGATGCAGCCAAACTTTATGCCGAAAAAACAGGT
ATTATAGTGAACGTTCATTACGGCCCACAGAACAAATGGAATGAAGATGCCAAAAAAAAT
GCAGATATCTTGTTTGGCGCATCAGAACAATCTGCTCTGGCTATCATTCGGGACCATAAA
GACAGCTTCAGTGAAAAAGATATTCAGCCTCTTTATCTGCGAAAAAGTATTTTACTGGTA
AAGAAAGGTAATCCTAAAAATATCCGGAGTATTGACGACCTGACCAGACCTGGGATTGGC
GTAATTGTTAATGATGGTGGTGGTACCAGTAATACATCAGGCACTGGCGTCTGGGAAGAT
ATTGCCGGACGTAAAGGGAATATAGAAACTGTCGCCGCAATCCGAAAAAATATTATTTTA
TATGCGCCCAATAGCGGAACTGCACGTAAGGCTCTTGAGAATCAGCCTGGAGCAGATGTC
TGGATAACCTGGGCTGACTGGGCAGCCAGTAATCCAGAAATTGGTGATGTCGTGGAAATA
GCGCCAGACTACGTGATATGGCGTGATATGAACATTACAGTACGTCAGGATGCAAATGAT
GAAACCCGTCGATTTGCAGAATGGCTACAAACCGATGAAGCGGCGCCTGCATTCAAAAAA
TATGGCTGGACCAGGAAAGGCACTTGA
>paa__STEC__adhesin__O103_12009
ATGGCAGGTTTTTTAATATTCCTGTCTTCTGCTGCTTATGCTGATATCAATCTGTATGGT
CCTGGTGGCCCGCATACAGCCTTGCTTGATGCAGCCAAACTTTATGCCGAAAAAACAGGT
ATTATAGTGAACGTTCATTACGGCCCACAGAACAAATGGAATGAAGATGCCAAAAAAAAT
GCAGATATCTTGTTTGGCGCATCAGAACAATCTGCTCTGGCTATCATTCGGGACCATAAA
GACAGCTTCAGTGAAAAAGATATTCAGCCTCTTTATCTGCGAAAAAGTATTTTACTGGTA
AAGAAAGGTAATCCTAAAAATATCCGGAGTATTGACGACCTGACCAGACCCGGGATTGGC
GTAATTGTTAATGATGGTGGTGGTACCAGTAATACATCAGGCACTGGCGTCTGGGAAGAT
ATTGCCGGACGTAAAGGGAATATAGAAACTGTCGCCGCAATCCGAAAAAATATTATTTTA
TATGCGCCCAATAGCGGAACTGCACGTAAGGCTCTTGAGAATCAGCCTGAAGCAGATGTC
TGGATAACCTGGGCTGACTGGGCAGCCAGTAATCCAGAAATTGGTGATGTCGTGGAAATA
GCGCCAGACTACGTGATATGGCGTGATATGAACATTACAGTACGTCAGGATGCAAATGAT
GAAACCCGTCGATTTGCAGAATGGCTACAAACCGATGAAGCGGCGCCTGCATTCAAAAAA
TATGGCTGGACCAGGAAAGGCACTTGA
>paa__STEC__adhesin__O111_11128
ATGGCAGGTTTTTTAATATTCCTGTCTTCTGCTGCTTATGCTGATATCAATCTGTATGGT
CCTGGTGGCCCGCATACAGCCTTGCTTGATGCAGCCAAACTTTATGCCGAAAAAACAGGT
ATTATAGTGAACGTTCATTACGGCCCACAGAACAAATGGAATGAAGATGCCAAAAAAAAT
GCAGATATCTTGTTTGGCGCATCAGAACAATCTGCTCTGGCTATCATTCGGGACCATAAA
GACAGCTTCAGTGAAAAAGATATTCAGCCTCTTTATCTGCGAAAAAGTATTTTACTGGTA
AAGAAAGGTAATCCTAAAAATATCCGGAGTATTGACGACCTGACCAGACCTGGGATTGGC
GTAATTGTTAATGATGGTGGTGGTACCAGTAATACATCAGGCACTGGCGTCTGGGAAGAT
ATTGCCGGACGTAAAGGGAATATAGAAACTGTCGCCGCAATCCGAAAAAATATTATTTTA
TATGCGCCCAATAGCGGAACTGCACGTAAGGCTCTTGAGAATCAGCCTGGAGCAGATGTC
TGGATAACCTGGGCTGACTGGGCAGCCAGTAATCCAGAAATTGGTGATGTCGTGGAAATA
GCGCCAGACTACGTGATATGGCGTGATATGAACATTACAGTACGTCAGGATGCAAATGAT
GAAACCCGTCGATTTGCAGAATGGCTACAAACCGATGAAGCGGCGCCTGCATTCAAAAAA
TATGGCTGGACCAGGAAAGGCACTTGA
>paa__clade1__adhesin__10290
ATGAGGAACATAATGGCAGGTTTTTTAATATTCCTGTCTTCTGCTGCTTATGCTGATATC
AATCTGTATGGTCCTGGTGGCCCGCATACAGCCTTGCTTGATGCAGCCAAACTTTATGCC
GAAAAAACAGGTATTATAGTGAACGTTCATTACGGCCCACAGAACAAATGGAATGAAGAT
GCCAAAAAAAATGCAGATATCTTGTTTGGCGCATCAGAACAATCTGCTCTGGCTATCATT
CGGGACCATAAAGACAGCTTCAGTGAAAAAGATATTCAGCCTCTTTATCTGCGAAAAAGT
ATTTTATTGGTAAAGAAAGGTAATCCTAAAAATATCCGGAGTATTGACGACCTGACCAGA
CCCGGGATTGGCGTAATTGTTAATGATGGTGGTGGTACCAGTAATACATCAGGCACTGGC
GTCTGGGAAGATATTGCCGGACGTAAAGGGAATATAGAAACTGTCGCCGCAATCCGAAAA
AATATTATTTTATATGCGCCCAACAGCGGAACTGCACGTAAGGCTCTTGAGAATCAGCCT

GAAGCAGATGTCTGGATAACCTGGGCTGACTGGGCAGCCAGTAATCCAGAAATTGGTGAT
GTCGTGGAAATAGCGCCAGACTACGTGATATGGCGTGATATGAACATTACAGTACGTCAG
GATGCAAATGATGAAACCCGTCGATTTGCAGAATGGCTACAAACCGATGAAGCGGCGCCT
GCATTCAAAAAATATGGCTGGACCAGGAAAGGCACTTGA
>papA__ExPEC__P_fimbriae__CFT073
TTACTGATAAGTCAGGTTGAAATTCGCAACTGCTGAGAAAGCACCTTCTGTTACAGGGTT
GCCACTACCATCTTTTCTGACGGCAGCAGTAAAATGAATTGTGTTATCACCATCAACTAA
ATAGGAAACACCTGTTGCTGTCGCACCATCAAATTTTACGCGTTTTCCATGTGGATCGGT
GACAACAATAGCTGTATTTGTTGCACCGACGGTCTGTAACATGTCACTCTGCGGACCACT
TGGGACACCCGAAAAAGTCAGAGATACTGTGCCAGTCTTCGCCCCACCACCGCCAGCAG
C
TTTTTTAAAGTTTGTAATATCACAATTTATAAGTTTAATATCAAAAGATTTGGGTTGACT
TTCCCCATCATTCTCCAGAAATAATTTTGATACTTGTCCAAAATCAATAGATTGATCAGC
AGACTGAGCATCAATACCACATGGTGCATCTACTACAGTTCCGTTAAAAGTTACTTTACC
CTGCCCCTGAGGAATAGTTGGAGCAGCATATGCACCAAAAGACACCACTGCCATAGCTAC
CGCACCGGCAATAACCGACTTAATCATAAATAACAACCTCTTTTTCATTACTGAACACAC
>papA__ExPEC__P_fimbriae__CFT073
ATGATTAAGTCGGTTATTGCCGGTGCGGTAGCTATGGCAGTGGTGTCTTTTGGTGCATAT
GCTGCTCCAACTATTCCTCAGGGGCAGGGTAAAGTAACTTTTAACGGAACTGTAGTAGAT
GCACCATGTGGTATTGATGCTCAGTCTGCTGATCAATCTATTGATTTTGGACAAGTATCA
AAATTATTTCTGGAGAATGATGGGGAAAGTCAACCCAAATCTTTTGATATTAAACTTATA
AATTGTGATATTACAAACTTTAAAAAAGCTGCTGGCGGTGGTGGGGCGAAGACTGGCACA
GTATCTCTGACTTTTTCGGGTGTCCCAAGTGGTCCGCAGAGTGACATGTTACAGACCGTC
GGTGCAACAAATACAGCTATTGTTGTCACCGATCCACATGGAAAACGCGTAAAATTTGAT
GGTGCGACAGCAACAGGTGTTTCCTATTTAGTTGATGGTGATAACACAATTCATTTTACT
GCTGCCGTCAGAAAAGATGGTAGTGGCAACCCTGTAACAGAAGGTGCTTTCTCAGCAGTT
GCGAATTTCAACCTGACTTATCAGTAA
>pchA__STEC__regulater__O157_O157_Sakai
ATGCTACATGATCACGTGGCAGAATGTCTGGAGAAAAAAGGACTGTACCGGAGAGCAGCT
GAACGATGGGCAAAAGTGATGGTACAGCTAAGTGATGACCAGAAAAGAAAAGTGGCGGC
A
CAGAAACGAGCAGAGTGTTTGCGTAAGGCGCGCCGGACTCCGGTTTCACCGGTGAACCT
G
ACCGAAATAAAACAAGCGGTCAACAGACTACATTCTGAGTTGGGAATGGGATTTGAAGAG
CGGCGGGTATTCCGACGATATAAAGGGACAGGAGAACAGAATACGTCCGGAAACGCGCG
G
TCAAAAAAATGCTAA
>pchA__STEC__regulater__O26_11368
ATGCTACATGATCACGTGGCAGAATGTCTGGAGAAAAAAGGACTGTACCGGAGAGCAGCT
GAACGATGGGCAAAAGTGATGGTACAGCTAAGTGATGACCAGAAAAGAAAAGTGGCGGC
A
CAGAAACGAGCAGAGTGTTTGCGTAAGGCGCGCCGGACTCCGGTTTCACCGGTGAACCT
G
ACCGAAATAAAACAAGCGGTCAACAGACTACATTCTGAGTTGGGAATGGGATTTGAAGAG
CGGCGGGTATTCCGACGATATAAAGGGACAGGAGAACAGAATACGTCCGGAAACGCGCG
G
TCAAAAAAATGCTAA
>pchA__STEC__regulater__O103_12009
ATGCTACATGATCACGTGGCAGAATGTCTGGAGAAAAAAGGACTGTACCGGAGAGCAGCT
GAACGATGGGCAAAAGTGATGGTACAGCTAAGTGATGACCAGAAAAGAAAAGTGGCGGC
A
CAGAAACGAGCAGAGTGTTTGCGTAAGGCGCGCCGGACTCCGGTTTCACCGGTGAACCT
G
ACCGAAATAAAACAAGCGGTCAACAGACTACATTCTGAGTTGGGAATGGGATTTGAAGAG
CGGCGGGTATTCCGACGATATAAAGGGACAGGAGAACAGAATACGTCCGGAAACGCGCG
G
TCAAAAAAATGCTAA

>pchA__STEC__regulater__O111_11128
ATGCTACATGATCACGTGGCAGAATGTCTGGAGAAAAAAGGACTGTACCGGAGAGCAGCT
GAACGATGGGCAAAAGTGATGGTACAGCTAAGTGATGACCAGAAAAGAAAAGTGGCGGC
A
CAGAAACGAGCAGAGTGTTTGCGTAAGGCGCGCCGGACTCCGGTTTCACCGGTGAACCT
G
ACCGAAATAAAACAAGCGGTCAACAGACTACATTCTGAGTTGGGAATGGGATTTGAAGAG
CGGCGGGTATTCCGACGATATAAAGGGACAGGAGAACAGAATACGTCCGGAAACGCGCG
G
TCAAAAAAATGCTAA
>pet__EAEC__serine_protease__O44:H18_042
ATGAATAAAATATACTCCATTAAATATAGTGCTGCCACTGGCGGACTCATTGCTGTTTCT
GAATTAGCGAAAAAAGTCATATGTAAAACAAACCGAAAAATTTCTGCTGCATTATTATCT
CTGGCAGTTATTAGTTATACTAATATAATATATGCCGCCAATATGGATATATCTAAAGCA
TGGGCCCGGGATTATCTCGATCTGGCACAGAATAAAGGGGTGTTTCAACCAGGTTCAACA
CATGTAAAAATAAAACTGAAAGACGGGACTGATTTTTCATTTCCAGCACTTCCTGTTCCT
GACTTTTCATCTGCAACCGCAAATGGAGCTGCAACAAGTATTGGTGGTGCCTATGCCGTA
ACCGTTGCACACAATGCAAAAAATAAGTCATCAGCTAATTATCAAACATACGGTTCTACG
CAATATACTCAAATAAACAGAATGACAACTGGAAACGATTTTTCCATTCAGCGATTAAAC
AAGTATGTCGTGGAAACAAGAGGGGCTGATACATCATTTAATTATAATGAGAACAACCAA
AATATTATTGACAGATATGGCGTAGACGTTGGAAATGGAAAAAAAGAAATCATTGGTTTT
CGTGTTGGTTCAGGAAACACCACTTTTTCCGGAATAAAAACATCCCAAACATATCAGGCT
GACCTGTTAAGTGCATCACTATTCCATATAACAAATTTACGAGCAAATACTGTCGGAGGT
AACAAAGTGGAATATGAAAATGACTCATATTTCACTAACTTAACCACTAATGGTGACAGT
GGATCAGGCGTGTATGTATTTGATAACAAAGAAGATAAATGGGTTCTACTTGGAACAACC
CATGGAATAATAGGGAACGGAAAAACGCAAAAAACATATGTAACACCATTTGACTCCAAA
ACCACCAATGAATTAAAGCAACTATTTATTCAAAATGTTAATATTGATAACAATACTGCT
ACCATTGGTGGTGGTAAGATAACTATTGGCAATACAACTCAAGATATCGAGAAAAATAAA
AATAACCAGAATAAAGACCTAGTGTTCTCTGGTGGTGGTAAAATCTCATTAAAAGAGAAT
CTTGATCTTGGATATGGTGGGTTTATTTTTGATGAAAATAAAAAATATACTGTTAGCGCT
GAAGGGAATAATAATGTCACCTTTAAAGGTGCAGGCATTGATATAGGTAAAGGCAGTACT
GTTGACTGGAACATCAAATATGCCTCAAATGATGCACTGCATAAAATTGGTGAAGGGAGC
CTTAATGTCATACAGGCACAGAATACGAATCTGAAAACCGGGAACGGGACCGTCATTCTT
GGCGCACAGAAAACGTTCAACAATATCTATGTCGCCGGTGGCCCGGGCACAGTACAACTC
AATGCAGAGAACGCCCTGGGTGAGGGTGATTATGCTGGTATTTTTTTCACTGAAAACGGC
GGAAAACTCGACCTGAATGGTCATAACCAGACCTTCAAAAAAATTGCTGCAACAGATTCC
GGAACCACCATCACTAACAGTAACACCACTAAAGAGAGTGTACTGTCGGTCAATAACCAG
AATAACTATATCTATCATGGTAATGTGGACGGCAATGTACGCCTTGAACATCACCTCGAC
ACTAAGCAGGATAATGCCCGCCTGATACTGGATGGTGATATTCAGGCAAACAGTATCAGT
ATCAAAAATGCCCCTCTGGTAATGCAGGGCCATGCGACTGATCACGCCATTTTCAGAACA
ACAAAAACAAATAATTGTCCTGAGTTCCTCTGTGGTGTTGACTGGGTCACCAGAATCAAA
AATGCTGAGAATTCAGTAAATCAGAAGAATAAAACCACCTATAAATCGAATAACCAGGTT
TCCGATTTGTCCCAGCCGGACTGGGAAACCAGAAAATTTAGATTCGACAATCTGAATATT
GAAGATTCATCATTATCCATTGCCAGAAATGCAGATGTTGAAGGTAACATCCAGGCTAAA
AACTCTGTGATAAATATCGGGGACAAAACGGCATATATTGATCTGTACTCAGGAAAAAAT
ATTACCGGTGCCGGATTCACCTTTCGTCAGGACATAAAAAGCGGTGACTCCATCGGTGAA
AGTAAATTTACCGGGGGCATTATGGCAACAGATGGCTCCATCAGCATAGGGGATAAAGCC
ATTGTCACGCTGAACACGGTCTCGTCTCTGGACAGAACAGCGCTGACTATCCACAAGGGG
GCGAATGTTACGGCCAGCAGTTCCCTTTTCACCACCAGTAACATCAAATCCGGAGGCGAC
CTGACCCTGACTGGCGCAACAGAATCGACCGGGGAAATCACTCCGTCGATGTTCTATGCT
GCAGGAGGATATGAACTGACGGAAGACGGGGCTAACTTTACCGCCAAAAATCAGGCCTCT
GTAACCGGTGATATTAAATCCGAAAAAGCAGCAAAACTTTCATTTGGCTCCGCTGACAAG
GATAATTCTGCCACAAGATATTCGCAGTTTGCTCTCGCGATGCTGGATGGCTTTGATACG
TCCTATCAGGGCAGCATTAAGGCTGCACAATCCAGCCTTGCAATGAATAATGCGCTCTGG
AAAGTGACCGGCAATTCCGAGTTGAAAAAACTGAACTCCACCGGCAGTATGGTGCTCTTC
AACGGAGGGAAAAACATCTTCAATACACTGACTGTCGATGAACTGACAACCAGTAACAGT
GCCTTTGTGATGCGAACCAATACACAACAGGCAGACCAGTTAATTGTTAAAAACAAACTG

GAAGGTGCAAACAACCTGCTGTTAGTCGATTTATTGAGAAAAAAGGAAACGACAAAAA
C
GGTCTGAACATCGATCTGGTTAAGGCTCCTGAGAATACCAGTAAGGATGTCTTCAAAACT
GAAACACAGACCATTGGTTTCAGTGATGTAACCCCTGAAATTAAACAGCAGGAAAAAGAT
GGCAAATCTGTCTGGACGCTGACCGGGTATAAAACGGTGGCAAATGCTGATGCTGCGAAA
AAGGCAACATCACTGATGTCAGGCGGCTATAAAGCCTTCCTTGCAGAGGTCAACAACCTC
AACAAACGTATGGGTGATCTGCGTGACATTAACGGTGAGGCCGGTGCATGGGCCCGTATC
ATGAGTGGAACCGGGTCTGCCGGCGGTGGATTCAGTGACAACTACACCCACGTTCAGGTC
GGTGCGGATAACAAACATGAACTCGATGGCCTTGACCTCTTCACCGGGGTGACCATGACC
TATACCGACAGCCATGCAGGCAGTGATGCCTTCAGTGGTGAAACGAAGTCTGTGGGTGCC
GGTCTCTATGCCTCTGCCATGTTTGAGTCCGGAGCATATATCGACCTCATCGGTAAGTAC
GTTCACCATGACAACGAGTATACCGCAACTTTCGCCGGCCTTGGCACCAGAGACTACAGC
TCCCACTCCTGGTATGCCGGTGCGGAAGTCGGTTACCGTTACCATGTAACTGACTCTGCA
TGGATTGAGCCGCAGGCGGAACTTGTTTACGGTGCTGTATCCGGGAAACAGTTCTCCTGG
AAGGACCAGGGAATGAACCTCACCATGAAGGATAAGGACTTTAATCCGCTGATTGGGCGT
ACCGGTGTTGATGTGGGTAAATCCTTCTCCGGTAAGGACTGGAAAGTCACAGCCCGCGCC
GGCCTTGGCTACCAGTTTGACCTGTTTGCCAACGGTGAAACTGTACTGCGTGATGCGTCC
GGTGAAAAACGTATCAAAGGTGAAAAAGACGGCCGTATGCTCATGAATGTTGGTCTGAAT
GCTGAGATTCGTGACAACGTACGCTTTGGTCTTGAGTTTGAGAAATCGGCATTTGGTAAG
TACAACGTGGATAACGCCATCAACGCCAACTTCCGTTACTCCTTCTGA
>pic__EAEC__serine_protease__O44:H18_042
GTGAATAAAGTTTATTCTCTTAAATATTGCCCCGTCACCGGGGGGCTTATTGCTGTCTCT
GAACTTGCCCGCAGGGTAATAAAAAAGACATGCCGAAGATTAACGCATATTCTTCTGGCT
GGCATTCCAGCAATCTGTCTGTGTTACTCTCAGATATCCCAGGCGGGTATTGTCCGTTCC
GATATTGCCTATCAGATTTATCGTGATTTCGCCGAAAACAAAGGGCTTTTTGTACCTGGT
GCCAATGATATTCCGGTATATGATAAGGACGGAAAACTTGTGGGAAGACTGGGTAAAGCC
CCAATGGCCGATTTCAGCAGTGTGAGCTCAAATGGCGTTGCTACGCTTGTATCACCTCAG
TATATCGTCAGCGTAAAGCATAACGGAGGATATCGGAGTGTGAGCTTTGGTAATGGGAAA
AATACATATTCCCTTGTTGACCGTAATAACCACCCTTCTATTGACTTCCATGCTCCACGT
CTGAATAAACTGGTTACAGAAGTTATTCCCTCAGCGGTAACATCAGAAGGAACCAAAGCC
AATGCTTATAAATACACTGAACGTTACACCGCTTTTTATCGGGTGGGTAGTGGTACGCAG
TACACTAAGGACAAGGACGGAAATTTAGTTAAGGTTGCCGGTGGATATGCTTTTAAAACA
GGAGGAACCACAGGAGTTCCTCTGATATCTGATGCAACAATAGTCTCTAATCCCGGGCAA
ACTTATAATCCTGTAAACGGCCCTTTACCTGACTATGGAGCCCCTGGGGATAGTGGTTCT
CCTTTGTTTGCTTATGATAAACAACAAAAAAAATGGGTTATTGTTGCTGTATTAAGAGCA
TATGCAGGTATTAATGGTGCTACGAACTGGTGGAATGTCATACCAACAGATTATCTGAAC
CAGGTTATGCAGGACGATTTCGATGCCCCCGTAGACTTTGTTTCCGGACTGGGCCCCCTG
AACTGGACATACGACAAAACATCAGGCACAGGTACCCTGAGCCAGGGCAGTAAAAACTG
G
ACCATGCACGGGCAGAAAGACAATGACCTCAATGCCGGTAAAAATCTGGTATTCAGCGGG
CAGAATGGTGCAATTATCCTGAAAGACAGTGTGACTCAGGGTGCCGGTTATCTCGAATTT
AAAGACAGTTACACCGTATCTGCTGAATCCGGAAAAACATGGACGGGTGCCGGCATTATT
ACTGACAAGGGGACGAATGTAACCTGGAAGGTCAACGGCGTTGCCGGTGACAACTTGCA
T
AAGCTGGGGGAAGGAACCCTGACCATAAACGGAACAGGTGTAAACCCGGGAGGACTGAA
A
ACGGGAGACGGTATCGTTGTACTTAACCAGCAGGCAGACACTGCAGGTAATATCCAGGCC
TTCAGTTCAGTGAACCTCGCCAGCGGACGTCCGACCGTGGTGCTCGGGGATGCCCGTCAG
GTCAATCCGGATAACATTTCATGGGGATACCGGGGAGGTAAGCTTGACCTTAATGGTAAT
GCCGTTACCTTCACCCGACTGCAGGCTGCTGATTACGGGGCGGTGATTACAAATAATGCA
CAGCAAAAATCCCAGCTTTTACTGGATCTTAAGGCTCAGGATACAAATGTCAGTGAACCG
ACGATTGGAAATATATCCCCCTTTGGTGGTACCGGCACTCCAGGAAACCTGTACAGCATG
ATACTCAACAGCCAGACCCGCTTCTATATTCTGAAATCTGCCAGCTATGGTAACACTCTG
TGGGGGAACAGCCTGAATGATCCGGCTCAGTGGGAGTTTGTTGGCATGGACAAAAACAA
A
GCAGTTCAGACAGTAAAAGATAGGATCCTGGCCGGGCGGGCAAAACAACCCGTTATCTTT
CATGGTCAGCTGACCGGGAATATGGATGTCGCCATTCCACAGGTGCCGGGGGGAAGAAAG

GTCATCTTTGATGGTAGCGTGAACCTGCCGGAAGGTACCCTGAGTCAGGACAGTGGCACC
CTGATATTCCAGGGACATCCGGTTATCCATGCCTCCATCAGTGGCAGTGCACCGGTCAGC
CTGAACCAGAAAGACTGGGAAAACCGTCAGTTTACAATGAAAACACTGTCGCTGAAAGA
C
GCTGACTTCCATCTTTCACGTAACGCCTCGCTGAACAGTGACATTAAGTCGGATAACAGC
CATATCACACTGGGAAGTGACAGGGCATTTGTGGATAAAAATGACGGAACAGGAAATTAT
GTCATTCCGGAGGAAGGTACCTCTGTCCCGGACACCGTGAATGACAGGAGCCAGTATGAA
GGGAATATTACGCTGAACCATAACTCAGCCCTGGATATCGGCAGCAGGTTCACCGGGGGG
ATTGACGCTTATGACAGTGCCGTCAGCATCACCTCTCCGGACGTCCTGTTGACAGCCCCG
GGTGCTTTTGCCGGCAGTTCACTGACAGTGCATGATGGCGGTCATCTTACAGCACTGAAC
GGTCTTTTCAGCGACGGGCATATTCAGGCCGGTAAGAACGGCAAAATCACCCTGAGCGGT
ACACCGGTTAAAGATACGGCTAATCAGTATGCCCCTGCTGTATATCTGACGGACGGATAT
GACCTGACCGGCGATAACGCAGCACTGGAAATTACCCGTGGAGCACATGCTTCCGGTGAT
ATTCATGCCTCTGCGGCATCAACAGTTACCATCGGGTCTGACACGCCGGCAGAACTGGCT
TCTGCGGAAACGGCTGCATCGGCGTTTGCCGGCAGTCTTCTTGAGGGCTATAACGCAGCA
TTCAATGGTGCCATAACCGGTGGCAGGGCTGATGTCAGTATGCATAATGCACTGTGGACT
CTGGGTGGGGACTCTGCCATCCACAGTCTTACCGTCAGAAACAGCCGTATTAGTTCTGAA
GGAGACCGTACATTCCGTACCCTGACGGTGAATAAACTGGATGCAACAGGCAGTGATTTT
GTTTTGCGTACGGACCTGAAAAATGCCGATAAAATTAATGTGACTGAAAAAGCCACTGGT
TCAGATAACAGCCTGAACGTCAGCTTTATGAATAATCCTGCTCAGGGACAGGCCCTGAAT
ATTCCTCTGGTCACGGCACCGGCGGGAACTTCAGCAGAGATGTTTAAGGCCGGCACCCGG
GTGACAGGTTTCAGTCGGGTGACCCCAACCCTGCATGTTGATACCAGTGGTGGCAATACG
AAGTGGATACTGGATGGTTTTAAAGCGGAGGCTGATAAAGCCGCTGCCGCGAAGGCTGAC
AGTTTCATGAATGCCGGGTATAAAAACTTCATGACGGAAGTTAACAATCTGAACAAACGT
ATGGGTGACCTGCGTGACACAAACGGTGATGCCGGTGCCTGGGCGCGCATCATGAGTGGT
GCCGGTTCTGCAGACGGTGGTTACAGTGATAATTACACCCATGTTCAGGTCGGCTTTGAC
AAAAAACATGAACTGGACGGTGTGGACCTGTTTACCGGTGTCACGATGACCTATACCGAC
AGCAGTGCAGACAGCCATGCATTCAGCGGAAAGACGAAATCGGTGGGGGGCGGTCTGTA
T
GCTTCAGCATTGTTTGAGTCCGGTGCCTATATCGATTTGATTGGTAAATATATTCACCAT
GACAATGATTACACAGGTAACTTTGCTAGCCTGGGAACGAAACACTACAACACCCATTCC
TGGTATGCCGGTGCTGAAACGGGTTACCGCTATCACCTGACAGAGGACACGTTCATTGAG
CCGCAGGCTGAACTGGTTTACGGCGCCGTGTCCGGGAAAACATTCCGCTGGAAAGACGG
T
GATATGGACCTGAGCATGAAGAACAGGGACTTCAGTCCGCTGGTTGGAAGAACAGGGGT
T
GAACTGGGCAAGACCTTCAGTGGTAAGGACTGGAGTGTGACGGCCCGTGCCGGAACCAG
C
TGGCAGTTTGACCTGCTGAATAATGGAGAGACCGTACTGCGTGATGCGTCCGGGGAGAAA
CGGATAAAAGGAGAGAAGGACAGCCGGATGCTGTTTAATGTTGGTATGAATGCGCAGATA
AAGGACAATATGCGCTTTGGTCTGGAGTTTGAGAAGTCAGCCTTTGGTAAATATAACGTG
GATAATGCGGTAAACGCGAATTTCCGGTATATGTTCTGA
>saa__STEC__adhesin__98NK2
ATGCTCAAAAGAAATTGTGTGCATTCGATATTAGCTCTTACCTTCATGGCAACGAGTACT
CATGCAGCCCCCCATAATGGAGTGACTTATGAAAGCCTTGAAAAAGGGAAGTTTAGTGAT
TTAAGTAGTTTTTTGAAAACGATCAATCTTTATGATAAAGATAATAATTTAATTGATAAT
GGGAAGCAACTTGACATAAGTAAAGCTGAAAACAAATATCTTTTTTACACATTAAAGACA
AACAAACAAGCAGGTCTCTCTCCTGAAGGTTTTGGCACGACATTGAGTTCAATCGCAACT
TATTATGATTATGCAAAAGAACAAGCTTACTTTGTAGATAAAGAGTCTAAAAAAATCCTA
TTATCATCCGATGGTTTAGGGAATGTAATGGTCATTGGACCTGATGGATATGGTTATACA
AAACATATACCTGAAATTAGTGAATACTTGTACAAGCTCAAGATAAATGGACCTCTTGGA
AATGCTGTTGACATGGAACTATCCGGAGAGGATATCAAAGCAATTTACTCGGAGATGTCA
TATCTTGGTAGCGGTAAAACGGAGGCAGGGGGAAGAGCATCTGCTACAGGAGTTGATTCG
ACCTCATTAGGATATAATAGTATCGCTAATCACTCTAATAGCATAGCCCTTGGCGCGAAC
TCAGTCACAACACGCTCAAATGAAGTCAGTATAGGCACAAAAAATGGAGAAACTCGCATA
ATTGGTGGTGTTTCTGATGGGAAATTAACAACAGATGCTGCTACAGTTGGCCAACTGTAT
CACGAATATTCGATTGAAAAAGTAGCACGTATTGAAGGTGATGCACAAACATTAAAATCA

GCAAACACTTATACAAATAAGCAAGTGAATGCGCTGGAGCAAAATACCAATCAACAACTT
CAACATGAAGCAACTGCCCGTATCGAAGGTGATGCACAAACACTAAAATCAGCAAACACT
TATACAAATAAGCAAGTGAATGCGCTGGAGCAAAATACCAATCAACAACTTCAACATGAA
GCAACTGCCCGTATCGAAGGTGATGCACAAACACTAAAATCAGCAAACACTTATACAAAT
AAGCAAGTGAATGCGCTGGAGCAAAATACCAATCAACAACTTCAACATGAAGCAACTGCC
CGTATCGAAGGTGATGCACAAACACTAAAATCAGCAAACACTTATACAAATAAGCAAGTG
AATGCGCTGGAGCAAAATACCAATCAACAACTTCAACATGAAGCAACTGCCCGTATCGAA
GGTGATGCACAAACACTAAAATCAGCAAACACTTATACAAATAAGCAAGTGAATGCGCTG
GAGCAAAATACCAATCAACAGTTTCGTCAATTACGAGATCAGATTATTAAAAATAGAAAA
CGTTCAGATGCCGGCATTGCAGGTGCAATGGCCATGACAGCAATACCAATGATTGATGGT
AAACAGTATTCTTTCGGCATGGCTGCAAGTAATTATCGTGATGAACAGGCTATTGCCGCT
GGTATAATTTTTCGCACCAGCGAAAACACCGTCGTCAGACTAAACACCTCATGGGATACA
CAACATGGAACAGGCGTTGCCACAGGCATGTCCATTGGATGGTAA
>sab__STEC__STEC_autotransporter_mediating_biofilm_formation__EH41
TTACCACTGCCAGCCCACACCGGCACCGACGGAGAAATTACCCCGGGTGTTCTGACCTGC
ATTCAGTTTCATTATCACCTTACCGTTATCGGAGATACGGGACACACCTATTGCAATGGC
GCTTTCACCACCATATCCACCGACACCCAGCCCCATCAGACTTTTACCCGGCAGATAGGC
CTGAGGTATCCCACTCATGGCCGCAGCCGATGCACCAACACCACGGATATCCCGGTTAAG
CTCTTCCACCCGGTAGCTCAGCTGGCTGAATTTATTCCCGAGAGTGTTTATCGCCTGTGT
ATTTCCGGCAACCTGACGGTTAGTTGCATGCAACTGACTGCCATTGACGGCATCCGTTGA
GTTCGCAGACAACACACCTGGTGCCACTGACTGCAGTTGACGCTCCTGTCCCGGCTGACC
AAAAGAAACCACCGCAGTATCACTTCCGGTACGTCCCGCAACAGCCTGACCGTTCAGGGC
ATAAGTCCCGGTATTCGCGCGGCCAGCACGGGCATGGCTGCCAATTGCAACAGAGCCGGC
AGCAGCCTGACGCGTGACTTCAGCACCATTACCCATGGCAATGCTGTTTTGTCCCCGGGA
AACGGCCCCCTGTCCGACAGCAATACTGTCACGTCCTTCCGCTTTTGCTGCCTGACCGGA
AGAGGCAGCTTTAAAATACTTCGTGCCTTCACGGTTCAGCATATTCACTGCATCAGCCAC
ATTATCCGGTGTTTTATGTACACCACCATTCACCGTTGTCTTACCGCCACCTGCAGTTGT
CAGCTCACTGAAGGTGTCAGGAGCCAGCGCTGCTTTTCCGTCTTTCCAGACAACATTATC
ACCGCCAGCAATTCTGGCTGCAGATGCCAGCTGGCTCCCGTTAACCGCATCCGTGGATAC
CGGTGACACATTTCCGGCCGCCACATTTGTCAGAACAAGAGGCTGCTCACTGCTTATTCT
GACACGGGCTGGTTCAGCCGGCGTCATTGTCAGTTTCCCGGTCGCAGCGTCTTCGGTCTG
TGTGTAAAAACGACCTCCAATATTGACAAGCGGGTTCCCCTGGCTGTCGGTGTACTCCAC
CGGAATCCCCGTCACATCAATCTCATAGCTGTAATTTCCCTTATCATCCTTACTGATGGC
TGAAACCCGGGTATTCTGTCCGTGACGGACTTCAACCGTCTGGGAGGAAACAGCAACAG
C
CTCATCACCGGATTCAGTCTTCAGTGCCCAAGTCGTTGCTGCAGTTGCCTGACTGATTTC
ACGGTTAAGTTGTGAAACGTTAACGGCATCCTGGTCATTCACCCCGTCTTTCACGCCGGA
AATAACCCGACCAGCCGCATCAATACCGTTCACAGTTACTGACGGGCCATCCTTGATGGT
CAGCCCATTTTTATCCATCCGGGTTCCACCGACAGAAACACTGCCGTCATCTGCCAGACG
AATGTTATCAGCGAGTCTGACCGTCAGGTTCCCGTTCTGAACAGTGGTGCTGATATTCTG
CCCATCACCACCAACCGTGATATGCTTCCCGAGAGACGTGTTCACCTTGCTTCCGTCATC
CGCCTGCAGACCGAAGCCAGTATTCACCAGTGCCGCACTGTTATCACTGAGAGCATTTTT
CAGGTCACCGATATTTGCCGCATTATTCAGAACATCTGCGCCGGCGTCAGCAAGCGCAGT
GCCGTTCAGACCACTGGCCACATTGGTTATCTGATTTCCACCGTTATTCAGGCCGGCCCC
GGTAAGGGAAACTGTTTTCCCCTGACCACCGCCCGCAGCAGGTGTTGTGATTGTGATGCC
TTTATCATTTACTGTTGTTATCTGCTCACCTGTACCAAAAACCGCGCTAGCTAAATCACG
GAGATTTCTGGTCATCCGTACGAGAAGACCATCTCCCTTTTCATTTGCATCAATACGAAT
GTTACTGTCTGTCACATCCTCATTATCACCAGCTCCACCTTTGATGGTCAGTTTTTCATT
CAGTTTTTTATGAATAACCTTTCCATCATCACCCACAAAAGAAAGACCATCATTCATGGT
CGCCAGCTCTTCTGCCGGTGATTTTTCATCAGGGGTATAAGTCAGACGTGTCTTCGTGGT
ACCGGCAACATCTGTTAATCCACCTTTACCTGCATTGACAGCAATGGTTGCTGATGCAGA
CTTTCCGTTCGCAGCAGACGTGGATGTCAGTTTAATCGTGCCATCTTTACCATTCAAACT
CACACTCTCCTCACCATCAGCCCCATTAACTTTGATGCTGCCAGGAGTATTATTACTCCC
CACACTCAGGTCATTATTCAGGGATATTTTTAACCCCTTATCTCCATTGGTGAGGGCTGT
GGTGTTGATATTGCTATCCCCGGATACTGTAATCGCCTGCCCAAGAGACTGTTTCACTTC
CTGTCCCTTAGAGTCTTTCAGTGCGAAAACAGCTCCCTGCATACTGGTGGATACACCATC
AATTGCAGTTTTCAGGTCTGCGATACTAGCGGCATTAAACTTTTCATTATCAGTGATAGT

ATTAAGAGTTTTACCATTCAACCCACTAGCTACTCCACTAATAACAACACCGCTGTTTTG
CCCAGCACCATTACCGCCTGACAATGTCAACTTATTACCTTCAGATGACAACGTTACACT
CCCGTTATTTCCTTTTCCATTCAGTTGAACACTACTGAATGACGGAGTATCAACAACAGA
GAACGTAAAATCACCGTTACTTTGTTTCACGTTAAGATTTTACCGGCTTTCAACGTAAC
AGTATCACCGGGATTAATTAATTCCTCCCTATCACCCTCAACCTTTCCACCATCATCACT
GCCTGCTTTAACTTTCCAACCAGACTGATTTATAGCATCAGTAATATTTTTGCAGTAAC
AAGCTTATTCTCATCCACCTTGTTTACAGTCATCTGCCCGTTACCACCATTTACAGGCAA
TACGGCTGTATCAGCTGAAATTTCCAGTTTATTTCCGTTAACACTGGTTTTTACTGGACC
TGAACCAGTGATTTCTATTTCATCTCCAAGATTTTTCGTTATCGTATTATTATCTCCACC
CTTAATCTTTATGGCATCATTTCTGATTGCCCGTTCCAGAGGTGCTCCATCAATTGTCAT
TCCCTTTTCATCCACCATAACATCAATATTGCCTTCGCCTTTGACTGTTATAGTATTGTT
ATTGGCCGTCTTTCCTGTATTAATCGTTACCGAACTTCCGTCTTTATCCTGAAGTATCCA
CCTAGGATTAATGACATCAAGGTCATTACCCAGTTTCTCAGCTACCGCATATAATTGGCT
GCCGTTAATTGCATCAGTACTTGTCTCAGATATAGAACCAGCAGCAACATTGACTATCTG
ACGTTCATTTCCATTCTTTCCAATGGAAAAAACACTTTGGGGATTTGAGGCTGCAAACCC
AGTATAAATCCTATCATTAATTTTTGCCTCAGTTACTTCTGTAGCCAACTTATCAACTGA
GCCAGAACCAATTGCTACACTCTTATCAAAATTTACAGTGGCATCTTGTCCCAGAGCTAC
CGCAGAGGGCTTATTTACGATCGCCGAGTCACCTATAGCTACAGAATTGACAGAATTTAT
ACCCTTAACATGGCTGTACGCCCCTAAAGCAATACTGTTTTCAGCCTCTGCTTTGCTATT
ATATCCTATCGCATTTGACTGATTTCCTCCAGCAACGGCTGTAGCACCCAGCGCGTTAGC
CCCCATACCAGTTGCTTTACTGACCGCACCAATTGCAAGAGATAAAGTTTCTGTTGCTTT
ACTTTGAGGGCCGACAACGATACTGGCGTCTCCTGTAGCCTCTGATGCTTCATAATTATC
AGGGATCAATATTCCCCCATACGAACGTCCTTTTAATGGCGTAAGGTCATCACCACCAAT
AACAATTGCTCCCCATCCACTTGCTTTAGTATCACCACCTATCGCGATTGACTGTTCTTT
AGTCGCTTGTGCTTTGGCGCCGATTGCAATAGATTCTCCTGTCTTAAACCCTTCTGGAGT
TTGTATCTCACCACCTGCTATCGCACTTGTACCAATAGCAATAGAATTACGCCCACTAGC
AATTGGAGACGTATGTTTGAAATAAATACCCTTATCTGGATCTTCTGGAACTTCACTAGT
AGTCCCTATTTCGATGCTCAGCCAGTCATTACCTGCTGTATCCACCGGAGCTTCATCAAC
CTCAATATGAGACGTATTCTTTTCTGCAAAGGTCATAGCAACCGGAGCTATTGCTAGCGC
AATAGAAACACAAAGGAGTGTTTTTTTATATTTCAT
>sat__ExPEC__autotransporter_toxin__CFT073
ATGAATAAAATATACTCCCTTAAATATAGTGCTGCCACTGGCGGACTCATTGCTGTTTCT
GAATTAGCGAAAAGAGTTTCTGGTAAAACAAACCGAAAACTTGTAGCAACAATGTTGTCT
CTGGCTGTTGCCGGTACAGTAAATGCAGCAAATATTGATATATCAAATGTATGGGCGAGA
GACTATCTTGATCTTGCACAAAATAAAGGTATTTTCCAGCCCGGAGCAACAGACGTAACA
ATCACTTTAAAAAACGGAGATAAATTCTCTTTCCATAATCTCTCAATTCCGGATTTTTCT
GGTGCAGCAGCGAGTGGCGCAGCTACCGCAATAGGAGGTTCTTATAGTGTTACTGTTGCA
CATAACAAAAAGAACCCTCAGGCCGCAGAAACCCAGGTTTACGCTCAGTCTTCTTACAGG
GTTGTTGACAGAAGAAATTCCAATGATTTTGAGATTCAGAGGTTAAATAAATTTGTTGTG
GAAACAGTAGGTGCCACCCCGGCAGAGACCAACCCTACAACATATTCTGATGCATTAGAA
CGCTACGGTATAGTCACTTCTGACGGTTCAAAAAAAATCATAGGTTTTCGTGCTGGCTCT
GGAGGAACATCATTTATTAATGGTGAATCCAAAATCTCAACAAATTCAGCATATAGCCAT
GATCTGTTAAGTGCTAGTCTATTTGAGGTCACCCAATGGGACTCATACGGCATGATGATT
TATAAAAATGATAAAACATTTCGTAATCTTGAAATATTCGGAGACAGCGGCTCTGGAGCA
TACTTATATGATAACAAACTAGAAAAATGGGTATTAGTCGGAACAACCCATGGTATTGCC
AGCGTTAATGGTGACCAACTGACATGGATAACAAAATACAATGATAAACTGGTTAGTGAG
TTAAAAGATACCTATAGTCATAAAATAAATCTGAATGGCAATAATGTAACCATTAAAAAC
ACAGATATAACATTACACCAAAACAATGCAGATACCACTGGTACTCAAGAAAAAATAACT
AAAGACAAAGATATTGTGTTCACAAATGGGGGAGATGTCCTGTTTAAGGATAATTTGGAT
TTTGGTAGCGGTGGTATTATCTTTGACGAAGGCCATGAATATAACATAAACGGTCAGGGA
TTTACATTTAAAGGAGCAGGAATTGATATCGGAAAAGAAAGCATTGTAAACTGGAATGCA
TTGTATTCCAGTGATGATGTTTTACACAAAATAGGCCCCGGTACTCTGAATGTTCAAAAA
AAACAGGGGGCAAATATAAAGATAGGTGAAGGAAATGTTATTCTTAATGAAGAAGGAACA
TTTAACAATATATACCTTGCAAGCGGAAATGGTAAGGTAATACTAAATAAAGATAATTCC
CTTGGCAATGATCAATATGCGGGGATATTTTTTACTAAACGTGGTGGTACGCTAGATTTA
AATGGACACAATCAGACTTTTACTAGAATTGCCGCCACTGACGATGGAACAACAATAACT
AACTCAGATACAACGAAAGAAGCCGTTCTGGCAATCAATAACGAAGACTCCTACATATAT

CATGGGAACATAAATGGCAATATAAAACTAACGCACAATATTAATTCTCAGGATAAGAAA
ACTAATGCAAAATTAATTCTGGATGGTAGTGTCAACACAAAAAATGATGTTGAAGTCAGT
AATGCCAGTCTTACCATGCAAGGCCATGCAACAGAGCATGCAATATTCAGAAGCTCAGCG
AATCATTGCTCCCTGGTATTTCTTTGTGGAACGGACTGGGTCACCGTTTTGAAAGAAACA
GAGAGTTCATATAATAAAAAATTCAATTCTGATTACAAAAGTAATAATCAGCAGACCTCA
TTTGATCAGCCTGACTGGAAAACCGGGGTGTTTAAATTTGATACATTACACCTGAACAAT
GCTGACTTTTCAATATCACGCAATGCCAATGTTGAAGGAAATATATCAGCAAATAAATCA
GCTATCACAATCGGCGATAAAAATGTTTACATTGATAATCTTGCAGGGAAAAATATTACT
AATAATGGTTTTGACTTCAAACAAACTATCAGTACTAATCTATCCATAGGAGAAACTAAA
TTTACAGGTGGCATCACTGCACATAACAGCCAAATAGCCATAGGTGATCAAGCTGTAGTT
ACACTTAATGGTGCAACCTTTCTGGATAATACTCCTATAAGTATAGATAAAGGAGCAAAA
GTTATAGCACAAAATTCCATGTTCACAACAAAAGGTATTGATATCTCCGGTGAACTGACT
ATGATGGGAATCCCTGAACAGAATAGTAAAACTGTAACGCCGGGTCTCCACTACGCTGCT
GATGGATTCAGGCTGAGTGGTGGAAATGCAAATTTCATTGCCAGAAATATGGCATCTGTC
ACCGGAAATATTTATGCTGATGATGCAGCAACCATTACTCTGGGACAGCCTGAAACTGAA
ACACCGACTATATCGTCTGCTTATCAGGCATGGGCAGAGACTCTTTTGTATGGCTTTGAT
ACCGCTTATCGAGGCGCAATAACAGCCCCCAAAGCTACAGTTAGCATGAATAATGCGATC
TGGCATCTAAATAGCCAGTCATCAATTAATCGTCTAGAAACAAAAGACAGTATGGTGCGT
TTTACTGGTGATAATGGGAAGTTTACAACCCTTACAGTGAACAACCTTACTATAGATGAC
AGTGCATTTGTGCTGCGTGCAAATCTGGCCCAAGCAGATCAGCTTGTTGTCAATAAATCG
TTGTCTGGTAAAAACAACCTTCTGTTAGTCGACTTCATTGAGAAAAATGGAAACAGCAAC
GGACTGAATATCGATCTGGTCAGCGCACCAAAAGGAACTGCAGTAGATGTCTTTAAAGCT
ACGACTCGGAGTATTGGCTTCAGTGATGTAACACCGGTTATCGAGCAAAAGAACGATACA
GACAAAGCAACATGGACTCTGATCGGCTATAAATCTGTGGCCAACGCCGATGCGGCTAAA
AAGGCAACATTACTGATGTCAGGCGGCTATAAAGCCTTCCTTGCTGAGGTCAACAACCTT
AACAAACGTATGGGTGATCTGCGTGACATTAACGGTGAGTCCGGTGCATGGGCCCGAATC
ATTAGCGGAACCGGGTCTGCCGGCGGTGGATTCAGTGACAACTACACCCACGTTCAGGTC
GGTGCGGATAACAAACATGAACTCGATGGCCTTGACCTCTTCACCGGGGTGACCATGACC
TATACCGACAGCCATGCAGGCAGTGATGCCTTCAGTGGTGAAACGAAGTCTGTGGGTGCC
GGTCTCTATGCCTCTGCCATGTTTGAGTCCGGAGCATATATCGACCTCATCGGTAAGTAC
GTTCACCATGACAACGAGTATACCGCAACTTTCGCCGGCCTTGGCACCAGAGACTACAGC
TCCCACTCCTGGTATGCCGGTGCGGAAGTCGGTTACCGTTACCATGTAACTGACTCTGCA
TGGATTGAGCCGCAGGCGGAACTTGTTTACGGTGCTGTATCCGGGAAACAGTTCTCCTGG
AAGGACCAGGGAATGAACCTCACCATGAAGGATAAGGACTTTAATCCGCTGATTGGGCGT
ACCGGTGTTGATGTGGGTAAATCCTTCTCCGGTAAGGACTGGAAAGTCACAGCCCGCGCC
GGCCTTGGCTACCAGTTTGACCTGTTTGCCAACGGTGAAACCGTACTGCGTGATGCGTCC
GGTGAGAAACGTATCAAAGGTGAAAAAGACGGTCGTATGCTCATGAATGTTGGTCTCAAC
GCCGAAATTCGCGATAATCTTCGCTTCGGTCTTGAGTTTGAGAAATCGGCATTTGGTAAA
TACAACGTGGATAACGCGATCAACGCCAACTTCCGTTACTCTTTCTGA
>sepA__clade1__serine_protease__10290
ATGTATCACATCACTCATAAAGTGGATATTATCACGAAACTATCTATTAAACAGCAGATT
AGCAGATACAACATGAATAAAATTTACTCACTAAAATATAGTAGCCTTACTGGTGGGCTT
ATAGCTGTATCAGAGTTAAGTAAAAAAGTTAAAGGAAAAACAGGCAGAAAATTAATGACT
GCTTCCGTTGCTTTATCTGTGAGTCTTTCAGCCTTACCCGCAGAGGCATCAACGGTCAGC
GCTGAAATACCATATCAGACTTTCCGTGATTTTGCTGAAAATAAAGGCGTATTTACACCT
GGTGCAACCGGAATTGAAATAAAAGACAAAAAATGGAAATGCAGTTGGTACGCTTGATGTG
CCAATGATTGACTTCAGCAGCGTGTCTCGGCGGGGCTCACTGACCCTGTTGTCGCAGGGA
TATGGTGTATCAGCAAAACACGGAGGCCTTGGTGATGTAAACAATGCCAGTTTTGGCTAT
GATAAAAATAATTATACTGTAGTAAAGAACAACAAACATTCGGGCCTTGATTTTTCTCTG
CATCGGTTCAGCAAACTCATCACAGAGGCTGCACCTGCTGATATAAATATATCCGGACAA
CTTAGTGATTCGTCACAGTATACTGCTTTTTACAGAGCAGGCGCTGGCACACAGTATATT
AAAGAACGCTCAGGTAAACAGACTCATATCCCGGGAACATTTTTAACTGGTGGAACAGTA
GGCACCCCATGGTATTCAGGAAATAATTTAATATCAAGCTCTCCAGGTGACACCTATAAT
AAATCACAGGGACCATTAGCCAGTTATGGGCAGATGGGTGACAGCGGGTCGCCATTATTT
GCTTATGATTCTCTCAGTGAAAAATGGTCTCTGGCTGGAGTCACCCTGCATAATAATGGT
GTTAATGGCCAAAAAAACAACTGGTTGTTATTACCTGAAGATTATATTAAAAATATTATT
ACAGCCGATTTCGATCCCATTATTTCATTTAATAAAAACAGTAAAGAACATATGTCATGG

ACATATGATGCAGCCAAAGGCGTGGGCCGTATTCAGCAGGATGACCAACAGTTTGTGATG
CATGGTAACCTGAATGGTAATCTGAATGCAGGTAAAAATCTTTATTTACAGGCGAAAAT
GGCATTATTGATTTAAAAGATAATGTTAATCAGGGGGCCGGATATCTTCAGTTTGCTGAT
GATTATACAGTAACAACGAGCAATGACAGTTCATGGTCCGGAGGAGGCATTATTGTCAAT
TATGGTACAACTGTAAAATGGGGTATAAACGGGGTTTCCGGAGATGACCTTCATAAAGTT
GGGGATGGAACGCTTATTATTAATGGCACAGGGAAAAATGAAGGGGGACTGAAGATTGGG
GCTGGTACTGTAATCCTTGAACAAAAAGCGAAAAACAACGATTCAACGGCTTTTTCTTCC
ATTAACATCAGTGGGGGAAACTCGCGTGTTAAACTCTCAGGAGATAATCAGATTATACCT
GATAATGTTTCCTGGGGATTCCGGGGTGGATATTTAGATATAAACGGAAAAAACACAGAG
TTCTCCCGCTTACAGGCTGTTGATTATGGGGCTGCAATTATTAACAGCAGCACAGACAAG
TCATTATTGACTTTAAATCTGTCACCATTGAAAAAAGATGAAATAGCTGTAAGCGTGAAG
GCGTTAGATATGAACGCCATCTTTCAGGGGGGACACGGGACAGCCGGAGATTTGTATAAA
ACAACTTTTTACGGTCCGACACAGTATTATCTGCTGAAAAAGCCCAAATTTGGCAGTGTG
CTGATGGGGTCACTGAAAAACACCAGCGAATGGCAATTTGCAGGAACAGACCTGAATCA
G
GCCGTTGATATGGCAAAGAATAATAAACTGACATCAAGTGCACAGGCTTCATATCTTTAT
CACGGTAAGCTCCTGGGGAATATGGATATTGTCATTCCGGAGCTTACAGGCAATGATATT
TTAACGCTTGATGGTTCTGTCAGTATATCAGGAGATATGTCAAAACAGGACGGTGCTCTT
ATCTTCCAGGGACACCCGGTTATTCATGCAGGGCAAACTGTTTCTGCATCGCAGAGTGAC
TGGGAGAACAGGGAGTTCTCACTCAACAATCTGAATCTTAATAATGCGGACTTCAGTCTG
TCCCGTAATGCATTTATGAACGGGAATATCAGGGCCGTTAACCAGAGCACTGTTATTATC
GGCGGAGATACAGTCTTTACTGATAAAAATGACGGAACAGGTAATGATGTCATCAGTGTT
GAAGGGAAATCTGCTGCCGCAGGAACATCCTCCTATACAGGGCATATCACTCTGGAGCAA
AAATCAGCACTGGATATCCGCGATAATTTTCGTGGCGGGGTTACGTCTGAAGACAGTCAT
ATCAATGTTTCTTCATCTTCAGTCCTGTTCTCAGATGCATCGTCATTTATAAACAGCTCC
CTGAATATTCATAAAGGAGGAGCGCTGACCGCTCAGGGAGGGCTGTTTACAAGTGGAAGC
ATTGATATTGGTGACGCTTCCCTTCTGCTTACCGGTACACCAGTGAATTCAGATGATGCT
GCTTTTTTACCGACCATCAATATGGCTGATGGCGGATTTAACCTGATGTCTGATTCATCA
GTACTGAAAGCCAGAGACCAGGCATCTGTTGTTGGTGATATTATTTCTGATAAACAGGCC
ACAATCAGCTTCGGAACTGAATCAGGTAAAGAGGGCATATTGTCTGAGAAGGCATCCCGG
GGACTGGCGGTCGGATTACTGAGTGGTTTTAATACGGCATACCGCGGTGCAATTCATGCC
CCGTCTGCATCTGCCACTGTGAACAATACCTGGTGGCAACTGACAGGAGACTCCTCACTT
CGCTCGTTAAAAAATACCGGAAGCATGACATATTTTACAGGAAGTGCAGCGAATAAAGCA
TTCCATACACTGACGGTTGATGAGCTGACGACGAATGGCACTGCGTATGCCATGCGTACG
GACCTGAAAAATGCGGATAAGCTGGTAGTAAACAAAAAGCTGTCAGGTAAGGACAATATT
CTGTTGGTTGATTTTCTGAACAAACCCTCCGGAGAAAAACTGGATATTGAACTGGTGAGT
GCACCGGGGAACAGCAGTAAAGATGTCTTTAAAGGAAGTGAACAGGCGATAGGTTTCAG
T
AATGTAACGCCGGTCATTACAACCCGGGAAACCGATGACAAAATAACATGGTCACTGACA
GGCTATAACACGGTTGCAAACAAGGAAGCAACCCGGAATGCCGCCGCCCTGTTCTCTGTT
GACTATAAAGCGTTTCTGAACGAGGTCAACAACCTGAACAAACGTATGGGGGACCTGCGT
GACATCAACGGCGAAGCCGGTGCATGGGCACGCATCATGAGCGGTACCGGCTCTGCCAGT
GGTGGTTTCAGTGACAACTACACGCACGTTCAGGTCGGGGTCGACAAAAAACATGAGCT
G
GACGGACTGGATTTGTTTACCGGTTTCACTGTCACACACACTGACAGCAGTGCCTCCGCC
GATGTTTTCAGTGGTAAAACGAAGTCTGTGGGGGCTGGCCTGTATGCTTCCGCCATGTTT
GATTCCGGTGCCTATATCGACCTGATTGGCAAGTATGTTCACCATGATAATGAGTACACT
GCAACCTTTGCCGGACTCGGAACCCGTGATTACAGCACGCATTCATGGTATGCCGGTGCA
GAAGCGGGCTACCGCTATCATGTCACTGAGGATGCCTGGATTGAGCCACAGGCTGAGCTG
GTTTACGGTTCTGTATCCGGTAAACAGTTTGCATGGAAGGACCAGGGAATGCATCTGTCC
ATGAAGGACAAGGACTACAATCCGCTGATTGGCCGAACTGGTGTGGATGTGGGTAAATCC
TTCTCTGGTAAGGACTGGAAAGTGACAGCCCGTGCCGGTCTGGGCTACCAGTTCGACCTG
CTGGCTAACGGCGAAACCGTATTGCGGGATGCATCTGGTGAAAAACGCATCAAAGGTGAA
AAGGACAGCCGTATGCTGATGTCCGTTGGCCTGAATGCAGAAATCAGGGATAACGTCCGC
TTTGGACTGGAGTTTGAGAAATCCGCCTTTGGTAAGTACAACGTTGATAATGCAGTCAAC
GCTAACTTCCGTTACTCGTTCTGA
>set1A__EAEC__serine_protease__O44:H18_042

ATGGAAGTCAGCGTCTTTCAGCGACAGTGTTTTCATTGTAAACTGACGGTTTTCCCAGTC
TTTCTGGTTCAGGCTGACCGGTGCACTGCCACTGATGGAGGCATGGATAACCGGATGTCC
CTGGAATATCAGGGTGCCACTGTCCTGACTCAGGGTACCTTCCGGCAGGTTCACGCTACC
ATCAAAGATGACCTTTCTTCCCCCCGGCACCTGTGGAATGGCGACATCCATATTCCCGGT
CAGCTGACCATGAAAGATAACGGGTTGTTTTGCCCGCCCGGCCAGGATCCTATCTTTTAC
TGTCTGAACTGCTTTGTTTTTGTCCATGCCAACAAACTCCCACTGAGCCGGATCATTCAG
GCTGTTCCCCCACAGAGTGTTACCATAGCTGGCAGATTTCAGAATATAGAAGCGGGTCTG
GCTGTTGAGTATCATGCTGTACAGGTTTCCTGGAGTGCCGGTACCACCAAAGGGGGATAT
ATTTCCAATCGTCGGTTCACTGACATTTGTATCCTGAGCCTTAAGATCCAGTAA
>set1A__shigella__exotoxin__Shigella_flexneri_2a_str._301
ATGGAAGTCAGCGTCTTTCAGCGACAGTGTTTTCATTGTAAACTGACGGTTTTCCCAGTC
TTTCTGGTTCAGGCTGACCGGTGCACTGCCACTGATGGAGGCATGGATAACCGGATGTCC
CTGGAATATCAGGGTGCCACTGTCCTGACTCAGGGTACCTTCCGGCAGGTTCACGCTACC
ATCAAAGATTACCTTTCTTCCCCCCGGCACCTGTGGAATGGCGACATCCATATTCCCGGT
CAGCTGACCATGAAAGATAACGGGTTGTTTTGCCCGCCCGGCCAGGATCCTATCTTTTAC
TGTCTGAACTGCTTTGTTTTTGTTCATGCCAACAAACTCCCACTGAGCCGGATCATTCAG
GCTGTTCCCCCACAGAGTGTTACCATAGCTGGCAGATTTCAGAATATAGAAGCGGGTCTG
GCTGTTGAGTATCATGCTGTACAGGTTTCCTGGAGTGCCGGTACCACCAAAGGGGGATAT
ATTTCCAATCGTCGGTTCACTGACATTTGTATCCTGAGCCTTAAGATCCAGTAA
>sfaA__ExPEC__S_fimbriae__UTI89
ATGAAGTTAAAATTCATCTCCATGGCTGTATTTTCAGCCCTGACCCTGGGTGTTGCGACA
AGTGCGTCTGCCGCACCTGCTACTGTCAATGGTGGCACTGTTCATTTTAAGGGGGAAGTG
GTTAATGCTGCATGTGCTGTAAATATGAACTCAGTAGATCAGACAGTTTTGCTCGGTCAA
GTTCGTACGAAAAAGTTAGCTAATCTTGATGATGTTAGTGGACCGGTTGGATTTAATATT
CAATTAGATGAGTGTGATTCCACAACATCTGGATCTGTGAAGATTCTTTTTTCTGGCACA
CCTGTTGCTGGAAAAAATAACGCCTTGGCTATACAAAGCTCGGCATCTGGAGCTGCAACA
AATGTTGGGATTCAGATACTTGACTCCTCAGGGAATCCTGTGACATTAAATTCGGATCAA
AGCGCAGTATATACATTGACAGACGGAACTAATAATATTCCGTTCCAGGCTCGCTATATT
GCTACTGGTCAATCAACAGCTGGTACAGCCAACGCCGACGCCACCTTTAAAGTTCAGTAC
CAGTAA
>stcE__STEC__zinc_metalloprotease__O157_Sakai
ATGAAATTAAAGTATCTGTCATGTACGATCCTTGCCCCTCTGGCGATTGGGGTATTTTCT
GCAACAGCTGCTGATAATAATTCAGCCATTTATTTCAATACCTCCCAGCCTATAAATGAT
CTGCAGGGTTCGTTGGCCGCAGAGGTGAAATTTGCACAAAGCCAGATTTTACCCGCCCAT
CCTAAAGAAGGGGATAGTCAACCACATCTGACCAGCCTGCGGAAAAGTCTGCTGCTTGTC
CGTCCGGTGAAAGCTGATGATAAAACACCTGTTCAGGTGGAAGCCCGCGATGATAATAAT
AAAATTCTCGGTACGTTAACCCTTTATCCTCCTTCATCACTACCGGATACAATCTACCAT
CTGGATGGTGTTCCGGAAGGTGGTATCGATTTCACACCTCATAATGGAACGAAAAAGATC
ATTAATACGGTGGCTGAAGTAAACAAACTCAGTGATGCCAGCGGGAGTTCTATTCATAGC
CATCTAACAAATAATGCACTGGTGGAGATCCATACTGCAAATGGTCGTTGGGTAAGAGAC
ATTTATCTGCCGCAGGGACCCGACCTTGAAGGTAAGATGGTTCGCTTTGTTTCGTCTGCA
GGCTATAGTTCAACGGTTTTTTATGGTGATCGAAAAGTCACACTCTCGGTGGGTAACACT
CTTCTGTTCAAATATGTAAATGGTCAGTGGTTCCGCTCCGGTGAACTGGAGAATAATCGA
ATCACTTATGCTCAGCATATTTGGAGTGCTGAACTGCCTGCGCACTGGATCGTGCCTGGT
TTAAACTTGGTGATTAAACAGGGCAATCTGAGCGGTCGCCTAAATGATATCAAGATTGGA
GCACCGGGTGAGCTGTTGTTGCATACAATTGATATCGGGATGTTGACCACTCCCCGGGAT
CGCTTTGATTTTGCCAAAGACAAAGAAGCACATAGGGAATATTTCCAGACCATTCCTGTA
AGTCGTATGATTGTTAATAATTATGCGCCTCTACACCTAAAGGAAGTTATGTTACCAACC
GGAGAGTTATTGACAGATATGGATCCAGGAAATGGTGGGTGGCATAGTGGTACAATGCGT
CAAAGAATAGGTAAAGAATTGGTTTCGCATGGCATTGATAATGCTAACTATGGTTTAAAT
AGTACCGCAGGCTTAGGGGAGAATAGTCATCCATATGTAGTTGCGCAATTAGCGGCACAT
AATAGCCGCGGTAATTATGCTAATGGCATCCAGGTTCATGGTGGCTCCGGAGGTGGGGGA
ATTGTTACTTTAGATTCCACATTGGGGAATGAGTTCAGTCATGAAGTTGGTCATAATTAT
GGTCTTGGTCATTATGTAGATGGTTTCAAGGGTTCTGTACATCGTAGTGCAGAAAATAAC
AACTCAACTTGGGGATGGGATGGTGATAAAAAACGGTTTATTCCTAACTTTTATCCGTCT
CAAACAAATGAAAAGAGTTGTCTGAATAATCAGTGTCAAGAACCGTTTGATGGACACAAA
TTTGGTTTTGACGCCATGGCGGGAGGCAGCCCTTTCTCTGCTGCAAACCGTTTCACAATG

TATACTCCGAATTCATCGGCTATCATCCAGCGTTTTTTGAAAATAAAGCTGTGTTCGAT
AGCCGTTCCTCCACCGGCTTCAGCAAGTGGAATGCAGATACGCAGGAAATGGAACCGTAT
GAACACACCATTGACCGTGCGGAGCAGATTACGGCTTCAGTCAATGAGCTAAGTGAAAGC
AAAATGGCTGAGCTGATGGCAGAGTACGCTGTCGTCAAAGTGCATATGTGGAACGGTAAC
TGGACAAGAAACATCTATATCCCTACAGCCTCCGCAGATAATAGAGGCAGTATCCTGACC
ATCAACCATGAGGCCGGTTATAATAGTTATCTGTTTATAAATGGTGACGAAAAGGTCGTT
TCCCAGGGGTATAAAAAGAGCTTTGTTTCCGATGGTCAGTTCTGGAAAGAACGTGATGTG
GTTGATACTCGTGAAGCGCGTAAGCCAGAGCAGTTTGGTGTTCCTGTGACGACTCTGGTG
GGGTATTACGATCCGGAAGGCACGCTGTCAAGCTACATCTATCCTGCGATGTATGGTGCC
TATGGCTTCACTTATTCCGATGATAGTCAGAATCTATCCGATAACGACTGCCAGCTGCAG
GTGGATACGAAAGAAGGGCAGTTGCGATTCAGACTGGCTAATCACCGGGCTAACAACACT
GTAATGAATAAGTTCCATATTAACGTGCCAACAGAAAGTCAGCCCACACAGGCCACATTG
GTTTGCAATAACAAGATACTGGATACCAAATCGCTCACACCTGCGCCAGAAGGACTTACC
TATACTGTAAATGGGCAGGCACTTCCAGCAAAAGAAAACGAGGGATGCATCGTGTCCGTG
AATTCAGGTAAACGTTACTGTTTGCCGGTTGGTCAACGGTCAGGATATAGCCTTCCTGAC
TGGATTGTTGGGCAGGAAGTCTATGTCGACAGCGGGGCTAAAGCGAAAGTGCTGCTTTCT
GACTGGGATAACCTGTCCTATAACAGGATTGGTGAGTTTGTAGGTAATGTGAACCCAGCT
GATATGAAAAAAGTTAAAGCCTGGAACGGACAGTATTTGGACTTCAGTAAACCTAGGTCA
ATGAGGGTTGTATATAAATAA
>stcE__STEC__zinc_metalloprotease__O103_12009
ATGAAATTAAAGTATCTGTCATGCATGATCCTTGCCTCTCTGGCGATGGGAGCATTTGCT
GCAACTGCTGCTGATAATAATTCAGCCATTTATTTCAATACCACCCAGCCCGTAAATGAT
CTGCAGGGGGGACTGGCCGCTGAGGTGAAATTTGCACAGAGCCAGATTTTATCTGCCCAT
CCTAAAGAAGGGGAAAGTCAACAACATCTGACCAGCCTGCGGAAAAGTTTGCTGTTGGT
C
CGTCTGGTGAAGGCTGATGATAAAACACCTGTTCAGGTGGAAGCCCGAGATGCTAATGAT
AAAATTCTCGGTACGTTAACCCTTTCGCCTCCCTCCTCACTACCGGATACCGTCTACCAC
CTAGATGGTGTTCCTGCTGATGGTATCGATTTACTCCTCAGAATGGAACGAAAAAGATC
ATTAATACGGTTGCTGAAGTAAACAAACTCAGTGATGCCAGCGGGAGTTCTATTAAAAGC
TATCTGGCAAATAATGCACTGGTGGAGATCCAGACAGCAAATGGTCGTTGGATAAGAGAC
ATGTATTTGCCGCAGGGAGCCGAGCTTGAAGGTAAGATGGTTCGCTTTGTTTCGTATGCC
GGCTATAATTCAACGGTTTTTTATGGAGATCGAAAGGTCACGCTCTCGGTGGGTAACACC
CTGCTGTTCAAATATGTAAATGGTCAGTGGTTCCGCTCCGGTGAGCTGGAGAATAACCGA
ATCGCTTATGCTCAGCATACCTGGAGTGCTGAACTGCCTGCGCACTGGATCGTGCCCGGG
TTAAACTTGGTGATTAAACAAGGTAACCTGAGTGGTAGCCTAAATGACATCAATGTTGGA
GCACCAGGGGAGCTGTTGTTGCATACCATCGATATCGGGATGCTGACCACTCCCCGGGGG
CGCTTTGATTTTGCCAAGGACAAAGAGGCGCATCGGGAATATTTCCAGACGATCCCGGTC
AGTCGTATGATCGTCAACAATTATGCTCCACTTCATCTGAAGGAAGTCATGCTACCTACC
GGTACATTGCTGACAGATGCAGATCCCGGTGAGGGTGGATGGCATAGCGGCACGATGCGA
CAGAGTATCGGTAAGGAGCTGATCTCTCATGGTATTGATAATGCCAACTATGGTATTAAC
AGCACTGCTGGTTCAGGAGAAAGCAGCCATCCTTATGTGGCTGCGCAACTGACCGCACAT
ACAAACCGCGGTAACTACTCCAATGGAGTACAGGTGCACGGTGGTTCCGGTGGAGGGGG
A
ATTGTTACCCTAGACTCTACCTTGGGTAATGAATTCAGCCATGAAGTCGGGCATAACTTC
GGTCTTGGTCATTATGTGGGTGGCTTCAGGGGGTCCGTGCATCGCAGCGCTGACCAGATC
AACTCGACTTGGGGCTGGGACAGCGATAAAAAACGTTTCATACCTAATTTTTATCCAGCC
CAGACAAACCGGAAAAGTTGTCTTGACGAGCAGTGCCAGGAGCCGTTCGACGGTCACTC
G
TTTGGCTTTGACGCCATGGCGGGAGGCAGTCCTTTCTCCGACGCAAACCGTTTCACAATG
TATACGCCAAATTCATCGGCTATCATCCAGCGCTTTTTTGAAAATAAAGCTGTGTTCGAT
AGCCTTTCCTCCACTGGATTCAGCAAGTGGAATGCCGATGAGCATAAAATGGAACCGTAT
GAACACACCATTGACAGGTTTGAGCAGATTACTGCTTCTGTCAGGGATCTCAGTGAAAAC
AAAATGGCTGAGATGATGGCAGAATATCCTATTGTCAAAGTACATATGTCGGACGGCAAC
TGGACTAGAGATATTCATGCCCCTGCAGCTTCCGCGGAGAATAAAGGCCGTATTCTGACA
CTCGATCATGAAGCTGGTTATAACAGCCATCTGTTTATCAACGGTGGTGAAAAGATAATT
TCCAAGGGATATAAGAAGAGCTTTGTTTCCGATGGGCAGACCTGGAAAGAAAGTGACGTG
GTGAATACCCGCGAAGCGCGTAAGCCAGAGAAGTTTGGCGTTCCGGTGACGACACTGGT

G
GGTTATTACGATCCCAAAGGCACTTTGTCTAGCTTCATCTATCCTGCAATGTACGGTGCG
TATGGTTTCACTTATTCTGATGACAGTGAGAACCTGTCCAGTAATGACTGCCAGCTGCAG
GTGGATACTAGAGAAGGGCAGCTACGATTCAAACTGGCTAATAATCGTGTGAACAGTTCA
GTGATGAACAAGTTCCATATCAACGTGCCGACTGAAAGTCAGCCCACTCAGGCGATTTTA
GTCTGTAATAACAAAGTATTGGATACCCGAACACTCACACCAGCACCAGAAGGTCTGACT
TATACTGTAAATGGTCGGGCACTTCCGGCAAAAGAAAACGAGGGATGTATCGTTTCTGTG
AAGACTGCCCAGCGTTACTGTCTCCCCGTTGGCCAGCGGTCAGGATCCAGTCTGCCAGAC
TGGATCGCAGGCCAGGAAGTCTATGTCGACAGCGGGGCCAAAGCGAAAGTGCTGCTCTCT
GACTGGGATAACCTGTCCTATAACAGGATTGGTGAGTTTGTGGGTAATGTGAATCCAGCT
GATATGAAAAAAGTTAAAGCTTGGAACGGGCAGTACCTGGACTTCAGCAGACCGAGGTC
G
ATGAGGGTTGTAAATAAATAA
>subA__STEC__Subtilase_cytotoxin__EC40
ATGCTTAAGATTTTATGGACGTATATTCTATTCCTGCTTTTTATATCTGCGTCAGCTAGG
GCGGAAAAACCCTGGTATTTTGATGCAATTGGTCTGACAGAAACAACAATGTCTCTTACA
GACAAAAATACCCCAGTGGTCGTATCTGTTGTTGATTCCGGAGTGGCATTTATTGGAGGT
CTCAGCGATAGTGAATTTGCAAAATTTAGTTTTACTCAGGATGGTTCACCGTTCCCGGTA
AAAAAGTCTGAAGCTTTATATATTCATGGTACTGCTATGGCTTCCCTCATTGCCTCACGT
TATGGGATTTATGGTGTTTATCCTCATGCTCTGATATCCAGTAGAAGAGTTATTCCTGAC
GGTGTACAGGACTCATGGATTAGAGCAATTGAAAGCATTATGTCGAATGTTTTTCTTGCT
CCAGGAGAAGAGAAAATCATTAATATATCGGGAGGCCAGAAGGGAGTGGCTTCCGCATCG
GTCTGGACAGAACTGCTTTCCCGTATGGGCAGAAATAATGATCGATTAATTGTTGCGGCA
GTGGGTAATGATGGCGCTGATATACGCAAACTGAGTGCTCAGCAGAGAATATGGCCAGCG
GCTTATCATCCTGTCAGCAGTGTGAATAAAAAGCAAGATCCTGTGATAAGAGTCGCTGCC
CTGGCACAGTACCGGAAAGGAGAAACACCGGTATTGCATGGTGGAGGAATTACCGGAAG
T
CGGTTCGGGAACAATTGGGTTGATATTGCTGCACCAGGGCAGAATATTACATTCCTCAGA
CCTGATGGCAAAACGGGGACTGGTAGCGGAACGTCAGAAGCAACAGCTATAGTTTCCGGC
GTACTGGCAGCAATGACCTCATGTAATCCCCGGGCAACAGCGACAGAACTGAAGCGAACG
CTGCTGGAGTCTGCAGATAAATACCCTTCACTTGTAGATAAAGTGACAGAAGGGAGGGTT
TTGAATGCAGAAAAAGCGATTAGTATGTTTTGCAAGAAAAATTATATTCCTGTCCGTCAG
GGAAGGATGAGTGAAGAACTGTAA
>t6ss_orf2__clade1__T6SS__10290
ATGACAATTACATCAACATTAGGGCAGAATGAAACACAGTATGCTACAGATGATTGTCTA
GAAGAAATTATAAATAATACCAGAGCTGTAAGACAAGATTCTGAAAAAACTAGATTTAAA
TTACAGGTTAATAATTTCCTTGCTGAAGTTGCCAGTGGTTCTTTAGTCATTAACAGTGAT
TTAATCGGTAGTATTGAAAAGCGAATCACTGATATTGATAAGTTGATGTCTGAGCAGCTT
AGTTTGATTATGCATGCAAGTGAGTTTCAAAAAATAGAGTCAGCGTGGACAGGGCTTTAT
AAGCTGGTTCATACCAGCGTAACTGAAAATGTAAAATATACAGTGTTACACTGTACTAAA
AAAGAGTTACTCAAAGATTTTAAATCATCCTCAGATTTCGACCAGTCTGTATTATTTAAA
AATATTTATGAAAGCGAGTATGGAACCTTCGGGGGAACACCATATTCTGCTTTTGTGGGG
GACTTCTATTTCGATAATACACCTCAGGATATTGATTTGCTGGAGCATATTTCCCATGTG
GCTGCCAGCGCTCATGCTCCGTTTTTGAGTTCTATTGCTCCTGGGATGCTATCCATGAAT
TCTTTTAGCGAACTACCCTACCCGCGTGATCTGGCAAAATTATTTGAAACAACAGATTAT
GCCCGGTGGCGTAGTTTCAGGCAGACAGAAGACAGTCGTTATGTGGGACTAACTTTGCCA
CAGTCCTTGGGACGTATCCCATATGGCATGAAGACTGTACCTGCTGAGACGTTTAATTTT
GAAGAGCACATTAGTGAAGACAACTCAGGAAAAGACTATCTGTGGGTAAATACAGCTTTT
GAACTTGCCTGTCGGATAGTTGATGCTTTTGAGGAGTATGGCTGGTGTGCAGCAATTCGT
GGTGTGGAAGGTGGTGGTTTGGTGAAATCATTACCGGCCTATAATTATGTATCCCATACG
GGTGAGAGATTATTACAATGTCCGACTGAGGTTGCTATCTCAGACCGAAGGGAAAAAGAA
CTGTCAGATCTCGGATTCATTCCACTGGTTTACTGTAAAGGCACTGATTTTGCAGCATTT
TTTGCCGTTCAATCGGTGAACAAACCACGGCTATACAATACTGATCAGGCTAATGCAAAT
GCAAAATTATCCAGCCAACTTCAGTATATATTAGCAACGTCACGATTCGCACATTATCTG
AAAGTTATTGTTCGTGATAAAGTTGGAAGTTTTATGTCAAAGACAGAATGTCAGACATAT
CTGCAAAACTGGATCATGCAATATGTTGTGGCATCAGATAACGCCGGACAGGAAACAAAG
GCACGTTATCCATTACGTGAAGCATCCATAGATGTTGTTGAAATTCCTGGCAGTCCTGGT

TCATATCGTGCAATTGCATGGATTAAACCTCATTTTCAGCTTGAGGGATTAAGTATGTCA
TTACGTCTTGTTGCTGACTTGCCATCAAGTGTAAGTTAA
>tia__ETEC__toxigenic_invasion_loci_A__H10407
ATGATTGAAATGAAAAAGGTTATTGCGGTTTCAGCGCTTGCAATGGCAGGTATGTTTTCG
GCCCAGGCTCTGGCTGATGAGAGCAAAACAGGCTTTTATGTGACCGGTAAAGCCGGTGCT
TCAGTTGTGATGCAGACTGACCAGCGCTTCCGTCAGGACTTTGGGGATGATGTTTATAAG
TATAAGGGCGGTGATAAAAACGATACTGTATTTGGTGCCGGCCTTGCAGTGGGCTATGAT
TTTTATCAACATTACAATGTTCCAGTACGCACGGAAGTGGAATTCTATGGCCGTGGAGCT
GCAGACTCCCGTTATACACTGGATACATGGCGTTCTCCGATGGGGGATGGTGGTCGGGAA
GACACACAAAATAGGCTCAGTGTGAATACCCTGATGGTGAACACGTATTATGATTTCAGA
AACAGCAGTGCATTTACTCCATGGGTATCTGTTGGCCTGGGTTATGCACGGGTACATCAT
AAGGCGACATATATTGATACCTCCTGGAATGAATCTGGCGAGATAAGTGATATTTCTGCG
TTACATTACTCGGGATATGATAACAACTTCGCATGGAGCATTGGGGCCGGTGTTCGCTAT
GACGTAACCCCGGATATCGCTCTTGACCTCAGCTATCGCTATCTGGATGCTGGTAAATCC
TCCCTGTCTTACAAGGATACAGAAGGGGATAAATATAAATCAGAGGCTGACGTTAAAAGT
CATGACATTATGCTTGGGGTAACTTATCATTTCTGA
>ureC__STEC__urease-associated_protein__O157_Sakai
ATGAGTAATATTTCACGCCAGGCCTATGCTGACATGTTCGGCCCTACCACCGGTGATAAA
ATTCGTCTGGCAGACACTGAGCTGTGGATCGAGGTCGAAGATGATTTAACTACCTACGGC
GAAGAGGTCAAATTCGGCGGCGGTAAAGTAATCCGCGACGGTATGGGACAGGGGCAAAT
G
CTCTCCGCCGGCTGCGCTGATCTGGTGCTGACCAATGCCCTGATCATCGATTACTGGGGG
ATCGTTAAAGCCGATATCGGCGTCAAAGATGGAAGGATATTTGCTATCGGCAAAGCCGGT
AATCCTGATATACAACCCAACGTCACTATCCCAATCGGCGTATCCACGGAAATTATTGCC
GCAGAAGGCAGGATCGTTACCGCAGGTGGCGTCGATACGCATATTCACTGGATCTGCCCA
CAGCAGGCTGAAGAAGCGCTGACATCCGGCATTACCACCATGATCGGTGGCGGTACTGGC
CCGACAGCGGGTTCTAACGCCACAACCTGTACCCCAGGACCATGGTACATTTATCAAATG
CTGCAGGCTGCAGACAGCCTGCCGGTCAATATCGGGTTGCTGGGTAAAGGCAATTGCTCC
AATCCGGATGCGCTTCGTGAGCAGGTCGCGGCCGGGGTTATCGGCCTCAAAATTCACGAA
GACTGGGGAGCTACACCTGCGGTAATCAACTGCGCACTGACTGTAGCCGACGAAATGGAC
GTTCAGGTTGCGCTACACAGTGATACGCTTAACGAATCAGGATTCGTTGAGGATACTCTG
ACTGCCATCGGCGGGCGCACTATCCATACCTTCCATACAGAAGGTGCAGGTGGTGGTCAT
GCTCCGGATATTATCACCGCCTGCGCGCACCCCAATATTCTGCCTTCCTCAACCAATCCG
ACGCTACCCTATACCGTCAACACTATTGATGAGCATCTGGACATGCTGATGGTTTGCCAT
CATCTTGACCCGGATATCGCCGAGGACGTAGCCTTTGCCGAATCGCGCATTCGCCAGGAA
ACCATTGCCGCGGAAGACGTCCTGCACGACCTTGGCGCGTTCTCCCTCACCTCGTCCGAT
TCGCAGGCCATGGGACGCGTCGGAGAAGTAGTGTTACGAACCTGGCAGGTGGCACACCG
G
ATGAAAGTTCAGCGCGGCCCGTTACCGGAAGAAAGTGGTGATAACGACAACGTCCGCGT
G
AAGCGCTATATCGCTAAATACACCATTAATCCGGCATTAACCCACGGTATTGCTCATGAA
GTCGGCTCGATTGAAGTGGGAAAACTGGCGGATCTGGTGCTCTGGTCCCCGGCGTTCTTT
GGCGTAAAACCGGCGACTATCGTCAAAGGCGGAATGATAGCCATGGCGCCGATGGGTGAT
ATCAACGGCTCTATCCCCACACCGCAGCCGGTGCACTATCGCCCAATGTTCGCTGCATTG
GGCAGTGCCCGTCACCGCTGTCGTGTGACTTTCCTGTCGCAGGCAGCAGCAGCAAATGGC
GTCGCTGAACAGCTTAACCTGCACAGCACAACTGCTGTGGTAAAAGGCTGCCGCACAGTA
CAAAAAGCCGATATGCGCCACAACAGCCTGTTGCCTGATATAACCGTGGATTCACAAACC
TACGAAGTGCGTATCAACGGCGAACTGATAACCAGTGAACCGGCGGACATTCTGCCAATG
GCGCAACGTTATTTCCTGTTTTAA
>ureC__STEC__urease-associated_protein__O26_11368
ATGTTCGGCCCTACCACCGGTGATAAAATTCGTCTGGCAGACACTGAGCTGTGGATCGAG
GTCGAAGATGATTTAACTACCTACGGCGAAGAGGTCAAATTCGGCGGCGGTAAAGTAATC
CGCGACGGTATGGGACAGGGGCAAATGCTCTCCGCCGGCTGCGCTGATCTGGTGCTGACC
AATGCCCTGATCATCGATTACTGGGGGATCGTTAAAGCCGATATCGGCGTCAAAGATGGA
AGGATATTTGCTATCGGCAAAGCCGGTAATCCTGATATACAACCCAACGTCACTATCCCA
ATCGGCGTATCCACGGAAATTATTGCCGCAGAAGGCAGGATCGTTACCGCAGGTGGCGTC
GATACGCATATTCACTGGATCTGCCCACAGCAGGCTGAAGAAGCGCTGACATCCGGCATT

ACCACCATGATCGGTGGCGGTACTGGCCCGACAGCGGGTTCTAACGCCACAACCTGTACC
CCAGGACCATGGTACATTTATCAAATGCTGCAGGCTGCAGACAGCCTGCCGGTCAATATC
GGGTTGCTGGGTAAAGGCAATTGCTCCAATCCGGATGCGCTTCGTGAGCAGGTCGCGGCC
GGGGTTATCGGCCTCAAAATTCACGAAGACTGGGGAGCTACACCTGCGGTAATCAACTGC
GCACTGACTGTAGCCGACGAAATGGACGTTCAGGTTGCGCTACACAGTGATACGCTTAAC
GAATCAGGATTCGTTGAGGATACTCTGACTGCCATCGGCGGGCGCACTATCCATACCTTC
CATACAGAAGGTGCAGGTGGTGGTCATGCTCCGGATATTATCACCGCCTGCGCGCACCCC
AATATTCTGCCTTCCTCAACCAATCCGACGCTACCCTATACCGTCAACACTATTGATGAG
CATCTGGACATGCTGATGGTTTGCCATCATCTTGACCCGGATATCGCCGAGGACGTAGCC
TTTGCCGAATCGCGCATTCGCCAGGAAACCATTGCCGCGGAAGACGTCCTGCACGACCTT
GGCGCGTTCTCCCTCACCTCGTCCGATTCGCAGGCCATGGGACGCGTCGGAGAAGTAGTG
TTACGAACCTGGCAGGTGGCACACCGGATGAAAGTTCAGCGCGGCCCGTTACCGGAAGA
A
AGTGGTGATAACGACAACGTCCGCGTGAAGCGCTATATCGCTAAATACACCATTAATCCG
GCATTAACCCACGGTATTGCTCATGAAGTCGGCTCGATTGAAGTGGGAAAACTGGCGGAT
CTGGTGCTCTGGTCCCCGGCGTTCTTTGGCGTAAAACCGGCGACTATCGTCAAAGGCGGA
ATGATAGCCATGGCGCCGATGGGTGATATCAACGGCTCTATCCCCACACCGCAGCCGGTG
CACTATCGCCCAATGTTCGCTGCATTGGGCAGTGCCCGTCACCGCTGTCGTGTGACTTTC
CTGTCGCAGGCAGCAGCAGCAAATGGCGTCGCTGAACAGCTTAACCTGCACAGCACAAC
T
GCTGTGGTAAAAGGCTGCCGCACAGTACAAAAAGCCGATATGCGCCACAACAGCCTGTTG
CCTGATATAACCGTGGATTCACAAACCTACGAAGTGCGTATCAACGGCGAACTGATAACC
AGTGAACCGGCGGACATTCTGCCAATGGCGCAACGTTATTTCCTGTTTTAA
>ureC__STEC__urease-associated_protein__O103_12009
ATGATGAGTAATATTTCACGCCAGGCCTATGCTGACATGTTCGGCCCTACCACCGGTGAT
AAAATTCGTCTGGCAGACACTGAGCTGTGGATCGAGGTCGAAGATGATTTAACTACCTAC
GGCGAAGAGGTCAAATTCGGCGGCGGTAAAGTAATCCGCGACGGTATGGGACAGGGGCA
A
ATGCTCTCCGCCGGCTGCGCTGATCTGGTGCTGACCAATGCCCTGATCATCGATTACTGG
GGGATCGTTAAAGCCGATATCGGCGTCAAAGATGGAAGGATATTTGCTATCGGCAAAGCC
GGTAATCCTGATATACAACCCAACGTCACTATCCCAATCGGCGTATCCACGGAAATTATT
GCCGCAGAAGGCAGGATCGTTACCGCAGGTGGCGTCGATACGCATATTCACTGGATCTGC
CCACAGCAGGCTGAAGAAGCGCTGACATCCGGCATTACCACCATGATCGGTGGCGGTACT
GGCCCGACAGCGGGTTCTAACGCCACAACCTGTACCCCAGGACCATGGTACATTTATCAA
ATGCTGCAGGCTGCAGACAGCCTGCCGGTCAATATCGGGTTGCTGGGTAAAGGCAATGGC
TCCAATCCGGATGCGCTTCGTGAGCAGGTCGCGGCCGGGGTTATCGGCCTCAAAATTCAC
GAAGACTGGGGAGCTACACCTGCGGTAATCAACTGCGCACTGACTGTAGCCGACGAAATG
GACGTTCAGGTTGCGCTACACAGTGATACGCTTAACGAATCAGGATTCGTTGAGGATACT
CTGACTGCCATCGGCGGGCGCACTATCCATACCTTCCATACAGAAGGTGCAGGTGGTGGT
CATGCTCCGGATATTATCACCGCCTGCGCGCACCCCAATATTCTGCCTTCCTCAACCAAT
CCGACGCTACCCTATACCGTCAACACTATTGATGAGCATCTGGACATGCTGATGGTTTGC
CATCATCTTGACCCGGATATCGCCGAGGACGTAGCCTTTGCCGAATCGCGCATTCGCCAG
GAAACCATTGCCGCGGAAGACGTCCTGCACGACCTTGGCGCGTTCTCCCTCACCTCGTCC
GATTCGCAGGCCATGGGACGCGTCGGAGAAGTAGTGTTACGAACCTGGCAGGTGGCACA
C
CGGATGAAAGTTCAGCGCGGCCCGTTACCGGAAGAAAGTGGTGATAACGACAACGTCCG
C
GTGAAGCGCTATATCGCTAAATACACCATTAATCCGGCATTAACCCACGGTATTGCTCAT
GAAGTCGGCTCGATTGAAGTGGGAAAACTGGCGGATCTGGTGCTCTGGTCCCCGGCGTTC
TTTAGCGTAAAACCGGCGACTATCGTCAAAGGCGGAATGATAGCCATGGCGCCGATGGGT
GATATCAACGGCTCTATCCCCACACCGCAGCCGGTGCACTATCGCCCAATGTTCGCTGCA
TTGGGCAGTGCCCGTCACCGCTGTCGTGTGACTTTCCTGTCGCAGGCAGCAGCAGCAAAT
GGCGTCGCTGAACAGCTTAACCTGCACAGCACAACTGCTGTGGTAAAAGGCTGCCGCAC
A
GTACAAAAAGCCGATATGCGCCACAACAGCCTGTTGCCTGATATAACCGTGGATTCACAA
ACCTACGAAGTGCGTATCAACGGCGAACTGATAACCAGTGAACCGGCGGACATTCAGCCA
ATGGCGCAACGTTATTTCCTGTTTTAA

>ureC__STEC__urease-associated_protein__O111_11128
ATGAGTAATATTTCACGCCAGGCCTATGCTGACATGTTCGGCCCTACCACCGGTGATAAA
ATTCGTCTGGCAGACACTGAGCTGTGGATCGAGGTCGAAGATGATTTAACTACCTACGGC
GAAGAGGTCAAATTCGGCGGCGGTAAAGTAATCCGCGACGGTATGGGACAGGGGCAAAT
G
CTCTCCGCCGGCTGCGCTGATCTGGTGCTGACCAATGCCCTGATCATCGATTACTGGGGG
ATCGTTAAAGCCGATATCGGCGTCAAAGATGGAAGGATATTTGCTATCGGCAAAGCCGGT
AATCCTGATATACAACCCAACGTCACTATCCCAATCGGCGTATCCACGGAAATTATTGCC
GCAGAAGGCAGGATCGTTACCGCAGGTGGCGTCGATACGCATATTCACTGGATCTGCCCA
CAGCAGGCTGAAGAAGCGCTGACATCCGGCATTACCACCATGATCGGTGGCGGTACTGGC
CCGACAGCGGGTTCTAACGCCACAACCTGTACCCCAGGACCATGGTACATTTATCAAATG
CTGCAGGCTGCAGACAGCCTGCCGGTCAATATCGGGTTGCTGGGTAAAGGCAATTGCTCC
AATCCGGATGCGCTTCGTGAGCAGGTCGCGGCCGGGGTTATCGGCCTCAAAATTCACGAA
GACTGGGGAGCTACACCTGCGGTAATCAACTGCGCACTGACTGTAGCCGACGAAATGGAC
GTTCAGGTTGCGCTACACAGTGATACGCTTAACGAATCAGGATTCGTTGAGGATACTCTG
ACTGCCATCGGCGGGCGCACTATCCATACCTTCCATACAGAAGGTGCAGGTGGTGGTCAT
GCTCCGGATATTATCACCGCCTGCGCGCACCCCAATATTCTGCCTTCCTCAACCAATCCG
ACGCTACCCTATACCGTCAACACTATTGATGAGCATCTGGACATGCTGATGGTTTGCCAT
CATCTTGACCCGGATATCGCCGAGGACGTAGCCTTTGCCGAATCGCGCATTCGCCAGGAA
ACCATTGCCGCGGAAGACGTCCTGCACGACCTTGGCGCGTTCTCCCTCACCTCGTCCGAT
TCGCAGGCCATGGGACGCGTCGGAGAAGTAGTGTTACGAACCTGGCAGGTGGCACACCG
G
ATGAAAGTTCAGCGCGGCCCGTTACCGGAAGAAAGTGGTGATAACGACAACGTCCGCGT
G
AAGCGCTATATCGCTAAATACACCATTAATCCGGCATTAACCCACGGTATTGCTCATGAA
GTCGGCTCGATTGAAGTGGGAAAACTGGCGGATCTGGTGCTCTGGTCCCCGGCGTTCTTT
GGCGTAAAACCGGCGACTATCGTCAAAGGCGGAATGATAGCCATGGCGCCGATGGGTGAT
ATCAACGGCTCTATCCCCACACCGCAGCCGGTGCACTATCGCCCAATGTTCGCTGCATTG
GGCAGTGCCCGTCACCGCTGTCGTGTGACTTTCCTGTCGCAGGCAGCAGCAGCAAATGGC
GTCGCTGAACAGCTTAACCTGCACAGCACAACTGCTGTGGTAAAAGGCTGCCGCACAGTA
CAAAAAGCCGATATGCGCCACAACAGCCTGTTGCCTGATATAACCGTGGATTCACAAACC
TACGAAGTGCGTATCAACGGCGAACTGATAACCAGTGAACCGGCGGACATTCTGCCAATG
GCGCAACGTTATTTCCTGTTTTAA
>virA__EIEC__invasion_and_intercellular__Shigella_sonnei_Ss046
TTAAACATCAGGAGATATGATGGCAAATGATACTTGACGACTTTCTGGAGGGATAGCATT
GCGAAGCCCTTCACTGCTTGGAAGTGTTCCAGCATGAACGAGATAGCTTGTTTCTATTTT
ATTAATACCAACACCATTACATGTTATGTGCGATGGTAATATTGATGAGCTAACTTCGTA
AGCCCTCCCCCCAGAGGCAGAGATTGCAGTGTCGTTTTTAGGGACAACTGCGTTGATTTT
TATAATATGTCCACAATTGTTAATAACTCGTTGTTCAGGCATGAAATTAAGCCTGTTTTC
TATGTTTTCGGACATAATTTGGGCATATATGTGTTCCACAGAGGAAGACGTGATGTGAAG
AGATTGCCAGAATGCAGATGTTAGTGCAGATATAAATGCAGGGTCTTGAAAGAGTGAAGT
AAACAAAAGTTTTTCTGTTTGAAAACATAGGTCTTGTTGGGGGAAGTTTGAATTGATTAT
TTTATTTACTTGGTTGTAACTTAGCATTTGAAGCTCTTTTTTGCTATCAATGAATTCATT
AATTCCTAACTCATTAATTAGATCTGCAATTAATTTTAGTGGTATTTCAATATTGTTGAA
ATATATTTTTGTACTATTGCTGTTAGATGCTCTAATATCTCTGTAAATATCATGCCAAAC
ACTATTAAAATCAGTGTCTTTAACGGGGAAGTAATCATCGTTTGGTATTATCGATGTACA
GCAATTAATGGCTGTTTCAATAACTTCTGGTGCAGGCTTGTGAGTTTTAAAATTCCCTGA
TACCGGAGCTGTAATAGTATGTACTTTTCCTAAGTTGAATTGTTTGGGGGGGAGTGAACT
TAACAAGTTATTATATTCTTTTGATGAAAGTTGAGTGGATTGTATTTTATCTCCATCAAG
ATAAAAAAACCGAACATATGCCTTTGTTAAGGCATAGCCGTATTGAAATGCCATCTATGTT
TGCGTGGGCAAACTCTCCGTCCATTTTATCTTGTGATAGTGATGGTGAGAAACTATCCAT
TAGTTTTTTTTCGCTATATTTTTCGGCTAGAGTTTCGTGTGGAGAATATATGCCGAAAGT
TATTATTTTCAGTAAAACGTCACAAAAACTCAATTTATTCCAACTTACTTCTGTAGTTGA
TTTGACAGTTGACATCCATGATGAGTCATTTCTTTCATGGTTAGTTATGTTTGATGTCTG
CAT
>CS1_cooB LR883053 ~~~cooB~~~CS1 CooB chaperone~~~
ATGCGAAAATTATTTTTAAGTTTGCTTATGATTCCCTTTGTTGCGAAGGCGAACTTTATG

ATCTATCCAATATCAAAAGAAATCAAAGGAGGCAGTAGTGAACTTATTCGTATATATTCT
AAATCAAAAGATACACAATATATAAAAGTGTATACTAAAAAAGTTTAAATCCGGGGACA
AAGGAAGAATACGAGGTAGACACCCCAAATTGGGAGGGAGGGTTGGTTACTACGCCGTCC
AAAGTAATCTTACCGGGTGGGGGCAGTAAGTCCGTTCGGCTAAGTCAGTTAAAGGACATC
AGTAGCGAAGATGTCTACAGAGTGTATTTTGAATCAATTAAACCAGAAAAACAGGATGGT
TTATCGAAAAATAAATCGCTGAAAACAGACCTATCTGTCAACATTATATATGCGGCATTA
ATAAGAGTGCTCCCCAAGGATGGGAAAAGTGATATGAGAGCATCATTATCACCCAAGAGC
AGTCTTCTTATAAAGAACACAGGAAATGTGCGGGTCGGAATAAAAGATGCTTTTTTTTGT
AAAAAAACAAGCATTAACAATGATGACTGCATAAAGAAAACATACAACAAGAATATCTAT
CCCGGTTCATCATTTGATACAGGGGTTATACAAAATGGATTCTCGCATATTTTTATCGAT
AGTGTTGATGGAAGCGCGGGCAAGCAAGGAAAGCGAATGCTAATAAGCATTCATTGA
>CS1_cooA LR883053 ~~~cooA~~~CS1 CooA major subunit~~~
ATGAAACTAAAGAAAACAATTGGCGCAATGGCTCTGGCGACATTATTTGCAACTATGGGA
GCATCTGCGGTCGAGAAGACCATTAGCGTTACGGCGAGTGTTGACCCGACTGTTGACCTT
CTGCAATCTGATGGCTCTGCGCTGCCGAACTCTGTCGCATTAACCTATTCTCCGGCTGTA
AATAATTTTGAAGCTCACACCATCAACACCGTTGTTCATACAAATGACTCAGATAAAGGT
GTTGTTGTGAAGCTGTCAGCAGATCCAGTCCTGTCCAATGTTCTGAATCCAACCCTGCAA
ATTCCTGTTTCTGTGAATTTCGCAGGAAAACCACTGAGCACAACAGGCATTACCATCGAC
TCCAATGATCTGAACTTTGCTTCGAGTGGTGTTAATAAAGTTTCTTCTACGCAGAAACTT
TCAATCCATGCAGATGCTACTCGGGTAACTGGCGGCGCACTAACAGCTGGTCAATATCAG
GGACTCGTATCAATTATCCTGACTAAGTCAACGTAA
>CS1_cooC LR883053 ~~~cooC~~~CS1 CooC Outer membrane usher~~~
ATGATTGGTGGAAAGTCGAGCAAGGTGGTCATTGTGTTATCCGTTCTTATTGGATCTTCT
TCCGGATTTGCCAGCAAATACAACCTTGTTGATATTCCGGAGTCTTTTCGTGATTTATGG
GGAGAGCAGGACGAATTACTCGAAGTCAGACTTTATGGGCAATCTCTTGGCGTTCATCGT
ATTAAGTCCACTCCTACTACTGTGGCATTTGAGTCTCCGGATAATTTATTAGATAAAATT
GAGATTAATAAAGGAAAGGAAGCTGACTTAAGAGTACTTATGCGAGGTTCATTCCAACGG
AATGGAAATATGAGTTGCCAGGGATATACGGGACAGAACAACTGCAATTACATTAAAACA
AACACAGTTGCGGTTATCGTAGATGATGTTGAAAATGTACTTAATCTTTTTATAGGGAAT
GAGTTTCTTGCTTCCGGAGAGAATGACAGTGATTATTATCAGCCATCCAAGAACACAAAA
AAAGCATTCATACATAGCCAGACAATTAATTTATCTGATACCGGTAATTATGAAAACTTG
TCCATTGTCGGGACGGGTTCGCTTGGGATAACAGATAACAGTTATGCTATTTTGGGTTGG
GCAGCAAATTATAATCGGTATAAATCTTACAATTACAATGAACAGTCGATTAACAGCCTG
TATTTCAGACATGATTTTGAAAAAAATTTTTACTATCAGTTGGGACGGATAGACAGATCC
GACTTATCGCAAAGTAGTGGCGGAAACTTTAACTTCGATCTACTTCCTGTACCTGATATT
TATGGTATGAGAGCCGGAACGACTCAGTCATATATCAAGAATACGGGAAAGTCAGTTGCA
TCTCCGGTCACAATTATGCTGACCCACTTTTCCCGTGTAGAAGCATATCGAAATGGGCAA
TTACTGGGAGTTTGGTATTTAGATGCAGGTATCAGTGAGTTAGATACGGAGCGTTTACCT
GACGGAAATTACGATCTGAAATTAAAAATATTTGAGCAGGAGCAACTTGTCCGTGAAGAA
ATTGTACCTTTTAACAAATCAGGTTCTTCAATTGGTGACACGCATTGGGATGTTTTCGTG
CAGGCAGGCGATATTATAAATGATAATGGCCGATATGTTGAAAAACAGAAAAACCATAAA
TCAGCCATTAATAGTGGATTACGTTTACCGTTAACAAGGAATCTTGCAGTACAGCTGGGG
GGGGTGTTATTGATAATAAAAATTATTACGAGACTGGGATTCTGTGGAACTCAGGACTCC
TTGATGGTTCTCTCAATAGCAAATTCACTTTCCTTTTTGGTGACGACACACATGGAAACT
ATCAGAATGTTTCCTATACTGATGGTTTCAGCTTATCGTTCTATCATAATGATAAGCGAG
TTGATGATTGTGGTAAAGATTACAATATGGGCTGGAGTGGATGTTATGAGTCTTATTCAG
CGTCTTTAAAGTATCCCTGTGAAAGGGTGGAATAGCACTCTTGCATACAGTAATACGTAC
AGTACGTCTGTATACAGATATGATGCTGTTTCTGAATATGTTCCTTATTACTATTATAAA
GGAAGAACTAAAAGATGGCAACTTACTGCTTCTACGGTGGTAAGATGGGGGGACTATAAC
ATTATGCCAACAATAGGAGTATATAATAGTGAACAGAAACAATGGGCTGATAAAGGAGGC
TATTTATCCTTAACGCTTACTCGAGTTGATGGTGGCAAGTCCTTGAATGCTGGTTATTCC
TATAACTACTCCCGGGGTAATTATACTTCTAATGATGCATTTGTTGAGGGGCATCTGGTT
TCAGATACAAATGTCAGTTATCGTGAACTAAGTGCACGCGTCAGTGGTAATAGATATTAC
ACTGAGGGAGGAGTTTCAGGGCGCATTAACAATAGATTTGGTGATCTGAATGGGACACTT
AATGTTAATAAAAACAGAAAATCACATGATACAACTCACTCTCTGACAGCTGGTTACAGT
TCGTCATTTGCTCTTACGACCGATGGCATCTACTGGGGAGGAAGCGCATCCGGGCTGACG
AATTTATCCGGAGGAATTGTAAGAGTAAAATCAAATGAGGATGAGAGTGAACTATTGAAT

GTGAAAGGCTCATCATATGGTCATTATTCCCTGGGCAGCAATGATAGTTTATTTATACCT
GTACCTGCCCTGATGCAAGCCAGCCTTACTATTGAAGAGAATACAAATAAATCTAAAAAT
ATTGATGTGCTCGCACCAACAAAAAACACTTTTTTATGTTACCTGGAAGTGTTTATCCT
ATTGATGTTTCAGCCAATGTTAGTTTTACTTACGTTGGACGTGGAGTTGATGTTAAGGGA
CGACCTTTATCTGGTGCATATATTTTGAATGCGCAAAATATTGTGTTGGATGAAAATGGT
GGATTTTCTTTTGAGAGTTCAGAGAATGAGAAGGAACTCTTTTTATTAAAAGATAAAACA
ATTTATTCCTGTTCATTAGACAGAAGCGAAATGCGCAATGGTATTGCTTTCGTTGGTGAG
GTTGCATGCAATTCTACCATCAAAGAACTTCTTCCTGAAAAATTGGTTACAAATTCTCGC
ATTCATGATTTATTAGCTTACAATCAGGATACTGAATGA
>CS1_cooD LR883053 ~~~cooD~~~CS1 CooD minor adhesin~~~
ATGAAAAAGATATTTATTTTTTTGTCTATCATATTTTCTGCGGTGGTCAGTGCCGGGCGA
TACCCGGAAACTACAGTAGGTAATCTGACGAAGAGTTTTCAAGCCCCTCGTCTGGATAGA
AGCGTACAATCACCAATATATAACATCTTTACGAATCATGTGGCTGGATATAGTTTGAGT
CATAGCTTATATGACAGGATTGTTTTTTTATGTACATCCTCGTCGAATCCGGTTAATGGT
GCTTGCCCAACCATTGGAACATCTGGAGTTCAATACGGTACTACAACCATAACCTTGCAG
TTTACAGAAAAAAGAAGTCTGATAAAAAGAAATATTAATCTTGCAGGTAATAAGAAACCA
ATATGGGAGAATCAGAGTTGCGACTTTAGCAATCTAATGGTGTTGAATTCGAAGTCTTGG
AGCTGTGGGGCTTACGGAAATGCTAACGGAACACTTCTAAATCTGTATATCCCTGCAGGA
GAAATCAACAAATTGCCTTTTGGAGGGATATGGGAGGCAACTCTGATCTTACGCTTATCA
AGATATGGCGAAGTCAGTAGCACCCATTACGGCAATTATACCGTAAATATTACGGTTGAT
TTAACTGATAAAGGTAATATTCAGGTATGGCTTCCAGGGTTTCACAGCAACCCGCGTGTA
GACCTGAATCTGCGCCCTATCGGTAATTATAAATATAGTGGTAGTAATTCACTCGACATG
TGTTTCTATGATGGATATAGTACAAACAGTGATAGCATGGTAATAAAGTTCCAGGATGAT
AATCCTACCAATTCATCTGAATATAATCTTTATAAGATAGGGGGCACTGAAAAATTACCA
TATGCTGTTTCACTGCTTATGGGAGAAAAAATATTTTATCCAGTGAATGGTCAATCATTT
ACTATCAATGACAGTAGTGTACTCGAAACAAACTGGAATCGAGTAACCGCAGTTGCTATG
CCGGAAGTTAATGTTCCAGTATTATGCTGGCCAGCAAGATTGCTATTAAATGCTGATGTA
AATGCTCCCGATGCAGGACAGTATTCAGGACAGATATATATAACATTTACACCCAGTGTC
GAAAATTTATGA
>CS2_cotB E1649_chr ~~~cotB~~~CS2 CotB chaperone~~~
ATGAAGATATTGTTATTTGTTATTCTGTTTTTTAATGTTTTTGCTGCCAGTGCAAATTTT
ATGGTATATCCGATCTCAAAGGATATACAGAGTGGTGGCAGCGAAACTATAAAAGTTTTT
TCAAAATCTAAAGATGTTCAGTATATAAAGATATATACGAAAAGGGTTATTAATCCAGGA
ACAAAAGAAGAGCAAGAGGTTGATATAAAAAATTGGGATGGAGGTCTGATTGTAACTCCG
GCAAAAGTTGTTTTGCCAGCTGGAGCAAGTAAGTCAATACGACTTACTGAGATAAATAAA
AAAGAGCAGGAGGAAGTCTATCGTGTGTATTTTGAATCTGTAAAACCGGGACAGCAAGAT
GATATAGAGGAAAAAAATGGGCGTGTAAATACTGATTTATCAGTAAACATAATCTATGCC
GCTCTCATAAGAACCAGCCCTGAGAACCCACAGAGGAAACTTGATGTATCCATAGAATCA
AACAATGTATGGATTAAGAACACTGGAAATATTAGGCTGGGAATTAAGGATGTATTCTTG
TGTGATACAACCAGCATAAATGATAAATGTGCAAAGTTTTCTTATAATAGAAATCTATAT
CCAGATATGTCGGTAGATACTAAATTAGGAAAAAAAAGGATTTTCTTATGCTGTCATTGAT
ACAAAGGATGACAGAAATGAAAATAGCGGAGAGTTAATTAACATAAAGCTCCCGTAA
>CS2_cotA E1649_chr ~~~cotA~~~CS2 CotA major subunit~~~
ATGAAACTCAATAAGATTATTGGAGCATTAGTTCTTTCATCTACATTTGTTAGCATGGGG
GCTTCTGCTGCCGAGAAAAATATCACTGTAACTGCTAGCGTTGATCCAACTATCGATCTG
ATGCAATCTGATGGCACAGCGTTACCAAGTGCAGTTAATATTGCATATCTTCCAGGAGAG
AAAAGATTTGAATCTGCTCGTATCAATACCCAAGTTCATACCAATAATAAAACTAAGGGT
ATTCAGATAAAGCTTACTAATGATAATGTGGTAATGACTAACTTATCTGATCCAAGCAAG
ACTATTCCTTTAGAGGTTTCATTCGCTGGCACTAAGCTGAGCACAGCTGCAACATCTATT
ACTGCCGATCAATTAAATTTTGGCGCAGCTGGTGTAGAGACAGTTTCTGCAACTAAGGAA
CTCGTTATTAATGCAGGAAGCACCCAGCAAACTAATATTGTAGCTGGTAACTATCAAGGA
TTGGTGTCAATTGTGCTTACTCAAGAACCTTAA
>CS2_cotC E1649_chr ~~~cotC~~~CS2 CotC outer membrane usher~~~
ATGCGAGCTTTCAATAAAATAACTGTTTTCATTTTGTTTATTCCTGGTTTATGTTTTGGA
ACGAATGGTTTAGAGAGTAAAAAAAATATTCCTGAAGAATTTATAGACTTATGGATGGAA
CAGGATGAATTACTTGAAGTTAATTTATATGGGCGTTCTCTAGGTGTTCATCGTGTATTG
ACAACGCCTACTACTGTGAAATTTTCATCTGTAGAGGAAATTCTAGAAAAGATTAATGTG

AAACAAGAGAAAAAAGAAGACCTGAGAAGTCTTCTTCTTCAATCATATTCCCGCAACGGG
AATATGAGTTGTAATGGGTTTGATGAAAAGGAATATAGCTGCAATTACATTAGAACTGAT
ACGGTTAATGTTATTGTAGATGAAGAAAATAATGAGCTAAATCTTTTTATAGGTGCGAGT
TTTCTTTCTGTTCAAGCTCAGGATAATATTTATTATCAAAAAAATATAAACTCAGAAAAA
GCATTCATTCACAGTCAGACAATTAACTTTTCTGAATCTGAAGGGTATAAAAGTTTATCT
TTGAAAGGGGTTGGTGCACAGGGGTTAACTGAAAATAGTTATCTTGTTTTTGGTTGGGAT
GCCATATATAATAGTTCTAGGAAATACACATATAAAAATCAGTCAATCAATAATATATAT
TACAGATATGATTTTGATAAAAAAATATTATTATCAGTTGGGGCGAATGGATCGTTCAGAT
TTATCAAGTGCCTCTAGTGGTAATTTTAATTTCAATATGCTTCCTTTGCCTGATATTGAT
GGATTTCAGATAGGTACGACCCAATCCTATATTAAAAATATCGAAAAATCAATATCATCG
CCAGTAACCGTTATGTTAACCCGATTTTCTAGGGTTGAAGCCTTTCGTAATGAAGAGTTA
CTGGGAGTATGGTATTTGAATTCAGGAATCAATGATCTCGATACAAGTCGTTTGCCTGAC
GGCAGTTATGATTTAACGTTGAAGATATTTGAGCAGGACATTCTTGTTCGTGAAGAGAAG
GTCCCTTTTAACAAGGGAGGAGCCTCTTTTGGGGATATGCAATGGGATGTGTTTGCTCAG
GCTGGTAATATTGTCAATAATAACGATAGTTATATTGAGAAGCAAACTAATAAAAAAACG
GGAATAAATGCTGGTATACGTACGCCTGTAACCAGAAATTTATCGTTCTTACAGGGCGGT
GCTATAATTGATAATGATAAATATTATGAGGCTGGTGTTAACTGGCGTTCAGGGTTTCTT
GATGGGGTACTAAGTGGAAACTTCAGTTTCCTGTATGGTGATGGTGCAAGAGGAAATTAT
CAAAATATTTCGTATACCGATGGTTTTAATCTCTCTTTTTATCGTAATGATAAAAGCGTT
GATAATTGTAGTCACAATTACAGTGCGGGATGGAGTGGGTGCTATGAGTCTTATTCCTTT
TCACTAAGTGTTCCTGTATCTGGCTGGACTACTACTCTTGGCTATAACCATACAAATAAT
GAGGCTGTACATAAATATGATTACACCCCGGAATATTTTTTTAGTAAAAAATATAAAGGT
GTCAGTAAAAGATGGCAATTGACATCTTCTTCGTCCTATAAATGGATGGATTATCATGTG
ATTCCGACGATAGGTGTATATCGTAGTGATCAGAGTCGATGGAGTGAGCAGGGAGGGTAT
TTTTCTTTGAGTTTTACCCGAGTAAAGGAAAATAGTGCCATTAATGCAGGATATTCTTAT
AATTATGTAAAGCATAAAAATGCCACACATGAGGCTTTTTTAGATGGTCGTATAACGACA
AATACTTTTGGCTATAGTGAATTAGGCTCTCGTATAAATACGAACAAAAATAACACAGAA
GCAGGTGTTACCGGACGTGTAAAAAACAGGTTTGGAGATCTGAATGGTTCATTAAATGTT
AATAAAAGTAAAACATCCGGTAAGATGACTCACTCAATGAGTGCAAACTATAACTCCTCA
TTTGCAATTACTGGTGATTCTGTCTATTGGGGGGGAGATGCCTCTGGTTTAACGAAGCTA
TCTGGGGGTGTGGTGAATGTAAGATCAGATGATAAATCAAAAGAGCTAATAAAAATATCA
GGTTCTTCATATGGTAATTATATCCTCGGCAGTAATGACCGTTCATTTATCCCTGTAAGT
GCATTAATGCCAAGTAACCTAACTATAGAAGAGATTCAGTCAAACGACAAGAATATTACT
GTTCAGGCGTTATCAAAAAATGACTTTTTTATTCTGCCTGGTAATGTTTTCCCTATTGAT
GTAACTGCTAATGTGACAGTTTCTTATATAGGGAGAGCTCTTGATGATAAAGGAAATCCA
TTATCAAATGCCCATATACTTGATGTTCACGGGGTTAGGCTGGATGAGGATGGTGGTTTT
TCTTTCGAAACTTCAGCTCAAAAGAAATCTCTTTTCCTGTTAAAAGATAAAGATATTTAT
TCATGTGATGTTAAGAAATATGATTTACGTAGTGGTGTTTTATTTACTGGTGACCTTATA
TGTGAACACAGTGGTATAGAACGTCTTGGAAAAGATTTGGTTAACAATCCAAGAGTTAAG
CAACTGCTTGCTTATAAATAA
>CS2_cotD E1649_chr ~~~cotD~~~CS2 CotD minor subunit~~~
TTGAAAAAAGTGATTTTTGTTTTATCCATGTTTCTATGTTCTCAGGTTTACGGGCAATCA
TGGCATACGAACGTAGAGGCTGGTTCAATAAATAAAACAGAGTCGATAGGCCCCATAGAC
CGAAGTGCTGCTGCATCGTATCCTGCTCATTATATATTTCATGAACATGTTGCTGGTTAC
AATAAAGATCACTCTCTTTTTGACAGGATGACGTTTTTATGTATGTCATCAACAGATGCA
TCTAAAGGTGCATGTCCGACAGGAGAAAACTCCAAATCCTCTCAAGGGGAGACTAATATT
AAGCTAATATTTACTGAAAAGAAAAGTCTGGCCAGAAAAACATTAAACTTAAAAGGATAT
AAGAGATTTTTATATGAATCAGATAGATGCATTCATTATGTCGATAAAATGAATCTCAAT
TCTCATACTGTTAAATGTGTAGGTTCATTCACAAGAGGAGTAGATTTCACTTTATATATC
CCACAAGGTGAAATTGATGGGCTTCTAACTGGAGGTATATGGGAGGCAACACTAGAGTTA
CGAGTCAAAAGGCATTACGACTATAATCATGGTACTTACAAAGTTAATATCACAGTTGAT
TTGACAGACAAAGGAAATATTCAGGTCTGGACACCAAAGTTTCATAGCGATCCTAGAATT
GATCTGAATTTACGTCCTGAAGGTAATGGTAAATATTCTGGTAGTAACGTGCTTGAGATG
TGTCTCTATGATGGCTATAGTACACATAGTCAAAGTATAGAAATGAGGTTTCAGGATGAC
TCACAAACAGGAAATAATGAATATAATCTTATAAAAACTGGAGAGCCATTAAAAAAATTG
CCATATAAACTTTCTCTTCTTTTAGGAGGACGAGAGTTTTATCCAAATAATGGAGAGGCT
TTTACTATTAATGATACTTCGTCATTGTTTATAAACTGGAATCGTATTAAGTCTGTATCC

TTACCACAGATTAGTATTCCAGTACTATGCTGGCCAGCAAACTTGACATTTATGTCAGAG
CTAAATAATCCAGAAGCGGGTGAGTATTCAGGAATACTTAACGTAACATTTACTCCTAGT
AGTTCAAGTCTGTAA
>CS3_cstA LR882976 ~~~cstA~~~CS3 CstA chaperone~~~
ATGACACCTATTAAGCTAATTTTTGCAGCTCTGTCTTTATTTCCATGCAGTAACATTTAT
GCAAACAATATAACCACTCAGAAATTCGAAGCTATATTGGGTGCAACAAGAGTAATTTAC
CACCTAGATGGTAATGGTGAAAGTCTAAGAGTTAAAAATCCGCAGGATTATCCAATTCTA
ATTCAATCTAAAGTAATGGACGAGGGTAGTAAAGATAATGCGGATTTTATTGTTACCCCC
CCTCTTTTTAGACTAGATGCAAAAAGAGAAACTGACATTCGTATAGTTATGGTGAATGGC
TTATACCCAAAAGACAGGGAATCTCTAAAGACCCTCTGTGTGCGAGGAATTCCACCAAAA
CAAGGAGATTTATGGGCTAACAATGAAAAAGAATTTGTTGGAATGAAACTTAACGTTTCA
ATTAACACATGTATTAAATTAATATTAAGACCACATAATCTTCCTAAACTTGATATTAAT
TCCGAAGGGCAGATAGAATGGGGGATAAGGGATGGTAATTTAGTAGCAAAGAATAAAACA
CCTTACTATTTTACTATAGTAAATGCATCGTTTAATGGAAAGGCACTCAAAACACCGGGG
ACGCTAGGGCCGTATGAGCAAAAACTTTACACGCTACCTAGTAAAATTTCTGTATCTGGA
CTGGTAAAGTGGGAAATTATTGGTGATCTAGGTGAGAGCAGTGAAACAAAGAAATTCAAT
ATTTGA
>CS3_cstB LR882976 ~~~cstB~~~CS3 CstB outer membrane usher~~~
ATGTACTTTGACGCTGGAGAAAGTGAGGATTTTTGTATTCAGTACTCTGTACTACAGGAT
ATAGGTGTAACTGTGAGTGGGAATCAGGATGAATGTGCAAATCTTGATGATGAATTAAAC
TTAAGAACCAGGTTTGATTTTTACTCGAAAAGAATGGATATTTTTGTATCACCAAAGTTT
GTTCCACGAAAAAAAAACGGTCTTGCGCCAATTAAACTTTGGGATGAGGGTGAAAATGCG
CTATTCACAAGTTACAACTTTAGTGAGGATTATTACCATTTTAAAGGTGACGCAAGAGAT
AGTTATTCACAATACGCTAACATTCAACCACGCTTAAATATAGGACCATGGAGAATAAGA
ACTCAAGCCATATGGAATAAAAATAATAACACAAAAGGGGAGTGGAGTAATAATTACCTG
TATGCCGAAAGAGGCTTAGGAAATATAAAGAGTAGACTATACATTGGGGATGGATATTTT
CCATTAAAAAACTTTAATTCGTTCAAATTTAAAGGAGGGGTGCTAAAAACTGATGAGAAT
ATGTATCCCTATTCAGAAAAAACTTATTCACCAATAGTTAAAGGCTCGGCAAAAACTCAA
GCAAAAGTTGAATTTTTTCAGGATGGTGTAAAAATTTATAGCTCAATCGTCCCTCCAGGG
GATTTTTCTATCTCAGATTATATTTTATCAGGCTCAAATAGTGATCTTTATGTCAAAGTT
ATAGAGGAAAATGGCTCAATTCAGGAATTTATCGTTCCATTTACCTATCCTGCAGTTGCG
GTCCGGGAAGGATTTACCTATTATGAAATCGCTATGGGAGAGACTCAGCAGTCGAATGAT
TATTTTACACAGTTATCATTTACTCGTGGGCTTCCATATGACTTTACCGTACTTACATCT
TTAGAATATTCTGGCTTCTACAGATCTCTTGAAATTGGGTTAGGGAAAATGCTTGGGAAT
TTGGGCGCATTATCGTTAATCTATGGACAGTCAAACTTTAGTAAAAGTGATAATAGTAAA
AATAAAAAATGGGATATCAGATATAATAAAAATATTCCGGACCTAAATACATATTTGAGT
TTTTCTGCTGTTAGCCAAACTAGAGGGGGGTATTCTTCACTCAGGGATGCTTTGGACTAT
GAGATCGGAGAATATACTTTTAACTCAAAAAACTCCTATACAGCCTCAATAAACCACTCA
TTAGGAGAGCTTGGTAGTTTAAACTTTAGTGGAACATGGCGAAACTACTGGGAGAATAAG
AACCAAACCAGATCTTACAATTTATCATATTCTACACAAATCTTTAATGGAAAGGCCTAC
TTGTCAGGAAGTTTGATTAGAAGTGAACTTATGAATTTTAATAATAAGATAAGTGATACT
ATTTTAAATATCGGTGTTAATATTCCCTTTGGCCTTTCTCGTGGCATTCAATCTGTAAGT
TATAACACCAGTTCAGTGAAAGGGGGGAGGAGTACTCATCAGTTAGGGATAAGTGGTTCT
GAATTTGACAATAAATTGTACTGGCATGTAAATCAGGGTTACTCAGATAATTACAGTAAT
ACCTCTATGTATGGTTATTATAAAGCTAAGTATGCTCAGGTTAATGCCGGATACTCAGTT
TCTGAGAGATACAATCATGCTTATGGAGGTATAGAGGGAGGAATTCTGGTATATGACGGT
GGAATTATTTTAGGTCGCAATCTTGGTGATACAATGTCAATTATTGAAGCTCCAGGTGCG
GAAAATACAAAGATTAGAGGATGGGGATCGATTGAAACTGATTGGAGGGGGAGGGCTTTT
ATTGGTTATCTTTCACCTTACCAAAATAATGATATATCCCTTGACCCATCATCATTACCA
TTAGACTCCTCTTTAGATATCACAACAAATTCGGTTATTCCAACAACTGGTGCAATTGTT
AAAACGACATATAATGTTAAAAAAGGAAAAAAAGTAATGCTTACTTTAAAAAAGTCAAAT
GGTGATGCAGTTCCATTTGGAGCAATTGTGACAGTTATGGATGGCGATCAAAATACAAGC
ATTGTGGGCGATAATGGGCAATTGTATTTAGGTTCCTCAATGGATACAGGAAGGCTAAAA
GTTATATGGGGAAATGGCGAAGATAAAAAATGTGTTGTTGACTACATAGTAGGTGACAAT
AAAAATATAGCGGGTATTTATATAGGCAGTGCCGGAACATGTATTTAGCTCAATGCTCCT
TTATGACAAAAAAATATCTTTTTTATCCGCTTCTGTTTGGTAG
>CS3_cstG LR882976 ~~~cstG~~~CS3 CstG minor subunit/adhesin/pilin~~~

GTGAAAAAAATGATTTTAGCATTGACTTTGATGTCGGTGTGGGGAGGTGCGTTTGCCGCA
GTGGGCCCAACGAAAGATATGAGTTTAGGTGCAAATTTAACTTCAGAGCCTACATTAGCT
ATTGATTTTACGCCTATTGAAAATATTTATGTAGGTGCCAATTATGGTAAAGATATTGGA
ACCCTTGTTTTCACAACAAATGATTTAACAGATATTACATTGATGTCATCTCGCAGCGTT
GTTGATGGTCGCCAGACTGGTTTTTTACCTTCATGGACTCATCAGCCACTTACAAAATT
AGTACAAAACTGGGATCATCGAATGATGTAAACATTCAAGAAATTACTCAAGGAGCTAAA
ATTACTCCTGTTAGTGGAGGGAAAACTTTGCCTAAAAAATTCACTCTTAAGCTACATGCA
CACAGGAGTAGCAGTACAGTTCCAGGTACGTATACTGTTGGTCTTAACGTAACCAGTAAT
GTTATTTAA
>CS3_cstH LR882976 ~~~cstH~~~CS3 CstH major subunit~~~
ATGTTAAAAATAAAATACTTATTAATAGGTCTTTCACTGTCAGCTATGAGTTCATACTCA
CTAGCTGCAGCGGGGCCCACTCTAACCAAAGAACTGGCATTAAATGTGCTTTCTCCTGCA
GCTCTGGATGCAACTTGGGCTCCTCAGGATAATTTAACATTATCCAATACTGGCGTTTCT
AATACTTTGGTGGGTGTTTTGACTCTTTCAAATACCAGTATTGATACAGTTAGCATTGCG
AGTACAAATGTTTCTGATACATCTAAGAATGGTACAGTAACTTTTGCACATGAGACAAAT
AACTCTGCTAGCTTTGCCACCACCATTTCAACAGATAATGCCAACATTACGTTGGATAAA
AATGCTGGAAATACGATTGTTAAAACTACAAATGGGAGTCAGTTGCCAACTAATTTACCA
CTTAAGTTTATTACCACTGAAGGTAACGAACATTTAGTTTCAGGTAATTACCGTGCAAAT
ATAACAATTACTTCGACAATTAAATAA
>CS4_csaA AF296132.1 ~~~csaA~~~CS4 CsaA chaperone~~~
ATGCATAAATTATTTTGTTTACTAAGTTTACTCATAACTCCATTTGTTGCAAATGCAAAC
TTTATGATATATCCAATATCAAAAGATTTAAAGAATGGAAATAGCGAGTTAATTCGTGTT
TATTCAAAATCAAAAGAGATACAATATATAAAAATATATACAAAAAAGATTATTAATCCC
GGCACAACTGAAGAACATGAAGTTGATATGCCCAATTGGGATGGTGGGTTTGTAGTTACT
CCTCAAAAAGTTATTCTTCCTGCAGGAGGGAGTAAATCAATACGTTTAACTCAATTTAGA
ATACCAAAAAAAGAGGAAATTTATAGAGTATATTTTGAGGCGGTAAAACCAGATAGCAAA
GAAAATGTAATTGATAATAAAAAACTAACAACAGAGCTATCTGTTAATATAATTTATGCG
GCTCTAATCAGATCTTTACCAAGTGAACAAAACATATCACTAAACATTTCTAGAAATGCA
AGAAAAAATATAATTATTTATAATAATGGGAATGTTAGAGCAGGTGTTAAAGATATTTAT
TTTTGTAAGTCATCTAATATCGATGATAGCTGTGTAAAAAAAAACGCATAACAAGAATATA
TATCCAGAAAAGTCATTTGATACGCTGGTTAATAACAATTTTTCTTATGTTTTCATTAAA
TTAAACCATGAAGACATAGAAAAAGAGCAAGGACTAATACAATTAAAAGTTCCTTGA
>CS4_csaB AF296132.1 ~~~csaB~~~CS4 CsaB major subunit~~~
ATGAAATTAAAAAAAACTATTGGTGCAATGGCACTGACCACAATGTTTGTAGCTATGAGT
GCTTCTGCAGTAGAGAAAAATATCACTGTAACAGCTAGTGTTGATCCTACAATTGATATT
TTGCAAGCTGATGGTAGTAGTTTACCTACTGCTGTAGAATTAACCTATTCACCTGCGGCA
AGTCGTTTTGAAAATTATAAAATCGCAACTAAAGTTCATACAAATGTTATAAATAAAAAT
GTACTAGTTAAGCTTGTAAATGATCCAAAACTTACAAATGTTTTGGATTCTACAAAACAA
CTCCCCATTACTGTATCATATGGAGGAAAGACTCTATCAACCGCAGATGTGACTTTTGAA
CCTGCAGAATTAAATTTTGGAACGTCAGGTGTAACTGGTGTATCTTCTTCCCAAGATTTA
GTGATTGGTGCGACTACAGCACAAGCACCAACGGCGGGAAATTATAGTGGGGTCGTTTCT
ATCTTAATGACCTTAGCATCATAA
>CS4_csaC AF296132.1 ~~~csaC~~~CS4 CsaC outer membrane usher~~~
ATGACAAAAAAAAATACATTATATATAACGATCATCGCAATGCTAACTCCATATTCAGTT
TTTTCCGGAGATATACCCAACTCTTTCCGTGATTTATGGGGAGAACAAGATGAATTTTAT
GAAGTAAAACTATATGGACAAACTCTAGGAATACATCGAATTAAAACAACCCCAACACAT
ATTAAGTTTTATTCACCCGAAAGCATTTTAGATAAAATAAATGTAAAAAAAGAAAAGGAA
AAGAAATTGAGTGTTTTGTTCACTAATTCTTTTTCAAGAAATGGCAATATGAGTTGTCAG
GGGAATGCTACTATACAGTATAACTGCAATTACATTAAAACAAAATCAGTAGATGTCATC
GTTGATGATGTTGATAATGTTGTTAACCTTTTTATAGGTAATGAATTTCTGGATTCTGAA
GCACACAATGATGAATATCATCAATTATCACGAAATGTAAAAAAAGCTTTTATACAAAGC
CAGACAATTAATGTCTCAGATTCTGGGAAGTATAAAAGTTTGTCTGTTTCAGGGAATAGC
GCGCTGGGTATTACAGATACAAGTTATGCTGTCTTAAATTGGTGGATGAATTACAATAAA
TTTAATGGTTACAGCAACAACGAAAGAACAATCAATAGTTTGTACTTTAGACATGATTTA
GATAAGAGATATTATTATCAATTTGGACGAATGGATCGTACAGATTTGTCACAAAGTATT
AGCGGGAACTTTAATTTTAACTTACTTCCTTTACCCGATATTGATGGTATAAGGACAGGA
ACCACACAATCTTATATCAAAAATACAGATAAGTTTATCGCATCCCCTGTAACTGTTATG

TTAACTAATTTTTCCAGAGTGGAAGCTTTTCGCAATAATCAATTATTGGGCGTATGGTAT
TTAGATTCTGGAGTAAATGAATTAGATACAGCTCGTTTACCTTATGGTAGTTACGATCTT
AAATTAAAAATTTTTGAAAATACTCAGTTAGTTCGTGAAGAAATAATTCCTTTTAATAAA
GGGAGAAGTTCTATTGGTGATATGCAATGGGACGTTTTCATTCAGGGAGGGAATATTATT
AATGACAAGGATCGTTACATAGAAAAACAAAATAATCATAAGTCATCAGTTAATGCTGGG
CTACGTTTACCAATTACGAAAAATATCTCTGTTCAACAAGGAGCATCTGTTATAGATAAT
AAAAATTATTATGAAGGGAGTCTCAAATGGAATTCCGGCATTCTGTCTGGCTCACTAAAT
AGTGAGTTCAGTTTTCTTTGGGGAGATAATGCAAAAGGTAATTATCAAAGTATCTCGTAT
ACCGATGGATTTAGTTTATCATTTTATCATAATGATAAGCGGGTCGATAATTGTGGAAGA
AATTACAATGCTGGTTGGAGTGGATGCTACGAATCATATTCGGCATCTTTAAGTATTCCT
TTATTGGGATGGACAAGTACTCTGGGATATAGTGACACTTATAGTGAATCAGTTTATAAA
AACCATATTCTTTCTGAATATGGTTTTTATAATCAAAACATATATAAAGGGAGAACCCAA
AGATGGCAACTGACTTCGTCCACCTCTTTAAAATGGATGGATTATAATTTTATGCCAGCA
ATTGGAATATATAACAGTGAGCAAAGACAACTGACTGATAAAGGCGGATATATATCTGTA
ACTCTCACCCGAGCCAGCAGAGAAAATTCATTAAACGCAGGGTATTCTTACAACTATTCC
AGAGGAAAGTATTCTTCTAACGAATTATTTGTTGATGGATATATGACATCAACAAATAAT
GGTGACTATCATGAGGTAAGAATGCGTTTTAATAAAAATAGACATAATGCAGAAGGTAGA
CTTTCAGGTCGTATAAACAATCGATTTGGAGATTTAAATGGTTCATTCAGCATGAATAAA
AACAGAAACACCAACAGTAGCAATCATTCTCTCACTGGTGGTTATAATTCCTCATTTGCT
CTTACAAGTGATGGATTTTACTGGGGAGGAAGTGCATCTGGTTTGACAAAACTAGCTGGC
GGTATTATCAAGGTTAAATCAAACGATACTAAAAAAAATCTGGTAAAAGTGACTGGGGCA
TTGTACGGTGATTATTCGCTAGGGAGCAACGATAATGCTTTTATTCCTGTACCAGCATTA
ACTCCAGCCAGTTTAATTATTGAAGATAATAATTATGGTGACAAGAATATTTCTGTACTT
GCACCAACGAACAACGATATGTTTATATTGCCGGGTAATGTTTATCCTGTTGAAATTGAA
ACCAAAGTAAGTGTTTCTTATATTGGTAGAGGTTTTGACAAAAACGGCACGCCACTTTCT
GGCGCACATGTTTTGAATGAACCACATGTTATCCTGGATGAGGACGGTGGATTTTCGTTT
GAATATACAGGTAATGAGAAAACACTTTTTTTATTAAAGGGCAGAACTATTTATACATGT
CAACTGGGGAAAAATAAAGTTCACAAAGGCATTGTTTTCGTCGGAGATGTTATATGTGAT
GTTAATAGCACAAGTTCCTTACCAGATGAATTTGTAAAGAACCCACGTGTGCAGGATTTG
CTGGCAAAGAATGATAAAGGATAA
>CS4_csaE AF296132.1 ~~~csaE~~~CS4 CsaE minor subunit/adhesin/pilin~~~
ATGAATAAGATTTTATTTATTTTTACATTGTTTTTCTCTTCAGTACTTTTTACATTTGCT
GTATCGGCAGATAAAATTCCCGGAGATGAAAGCATAACTAATATTTTTGGCCCGCGTGAC
AGGAACGAATCTTCCCCCAAACATAATATATTAAATAACCATATTACAGCATACAGTGAA
AGTCATACTCTGTATGATAGGATGACTTTTTTATGTTTGTCTTCTCACAATACACTTAAT
GGAGCATGTCCAACCAGTGAGAATCCTAGCAGTTCATCGGTCAGCGGTGAAACAAATATA
ACATTACAATTTACGGAAAAAAGAAGTTTAATAAAAAGAGAGCTACAAATTAAAGGCTAT
AAACAATTATTGTTCAAAAGTGTTAACTGCCCATCCGGCCTAACACTTAACTCAGCTCAT
TTTAACTGTAATAAAAACGCGGCTTCAGGTGCAAGTTTATATTTATATATTCCTGCTGGC
GAACTAAAAAATTTGCCTTTTGGTGGTATCTGGGATGCTACTCTGAAGTTAAGAGTAAAA
AGACGATATAGTGAGACCTATGGAACTTACACTATAAATATCACTATTAAATTAACTGAT
AAGGGAAATATTCAGATATGGTTACCTCAGTTCAAAAGTGACGCTCGCGTCGATCTTAAC
TTGCGTCCAACTGGTGGGGGCACATATATTGGAAGAAATTCTGTTGATATGTGCTTTTAT
GATGGATATAGTACTAACAGCAGCTCTTTGGAGATAAGATTTCAGGATAACAATCCTAAA
TCTGATGGGAAATTTTATCTAAGGAAAATAAATGATGACACCAAAGAAATTGCATATACT
TTGTCACTTCTCTTGGCGGGTAAAAGTTTAACTCCAACAAATGGAACGTCATTAAATATT
GCTGACGCAGCTTCTCTGGAAACAAACTGGAATAGAATTACAGCTGTCACCATGCCAGAA
ATCAGTGTTCCGGTGTTGTGTTGGCCTGGACGTTTGCAATTGGATGCAAAAGTGGAAAAT
CCCGAGGCTGGACAATATATGGGTAATATTAATGTTACTTTCACACCAAGTAGTCAAACA
CTCTAG
>CS4_csaD_truncated AF296132.1 ~~~csaD truncated~~~CS4 CsaD regulator~~~
ATCAGTAAGTTGGCAGCATCACCTGTATTTCTTGAAAGAGGGGTGAATATATCTGTAAGA
ATACAGAAGCAAATTTTATCAGAAAAACCATATGTTGCATTCAGATTGAACGGAGACATA
CTAAGACATTTAAAGGATGCATTGATGATAATATATGGTATGTCAAAAATAGATACCAAT
GATTGTAGAAATATGTCAAGGAAAATAATGAAAACAGAAGTGGATAAAACCTTACTGGAT
GTATTAAAAAATATAAATAGCTATGATGACTCAGCTTTTATATCTAATTTGATATATTTA
ATTTCAAAGATCGAGAATAATAAAAAATAA

>CS5_csfA LR883008 ~~~csfA~~~CS5 CsfA major subunit~~~
ATGAAGAAAAATTTACTGATAACTTCAGTGTTGGCAATGGCAACCGTATCAGGTTCTGTT
TTGGCTGCTGTTACAAATGGCCAACTCACATTTAATTGGCAGGGAGTGGTTCCTTCCGCT
CCCGTTACTCAGAGCAGCTGGGCTTTTGTGAACGGATTGGATATACCGTTTACTCCTGGT
ACTGAACAGTTGAATATCACCCTTGATTCAAATAAAGATATCACGGCCCGTTCGGTTAAG
CCTTATGATTTTTTCATTGTTCCAGTTTCTGGAAACGTAACTCCTGGAGCGCCGGTTACG
CGTGACACGTCAGCTAATATAAACAGTGTGAACGCTTTTCTATCAAGTGTACCCGTTTCT
AATGGTTTTGTTGGCAACAAGCAGTTAACCCTGAGTACCGCAGTAGAAGCAGCTAAGGGG
GAAGTCGCAATCACTTTAAATGGTCAAGCGCTTAAAGTGGGGAGCGCTAGTCCAACAGTT
GTTACTGTGGCTAGTAATAAAAAAGAGTCTCATATTTCTATTGATATGAATGCCAAGGCA
GCTGCTGCGGATGTGGCAGAGGGGGCAGCTATTAACTTTGTAGCTCCGGTAACATTTGCT
GTTGATATTTAA
>CS5_csfB LR883008 ~~~csfB~~~CS5 CsfB chaperone for CsfA~~~
ATGAAGATTCTGTATTCTTTTTTGTTGTTACCTTTTTTTTCTTGCGCCTTCAGTGTTGAT
TCAATGATAAAGTTTTCAGGCGAAGATGACTTTTTTCTTGTAAATGGAAATAGCAAGGAA
AGAGAGTATATCTATGTAACGCTTTCTGAACTAATTAGCGAGAAAAACAATAGGCGCGAT
GAAATATTTTACAACGCAGACAATGTGCCTCTATGGCCTATATCTGCAGAACCTACAGAT
ATTATTATTTCATCTGGCGAACAAGTCAAAATAAAAATCAACAAAAATTATACTCCTGTC
GGAGGAGATCGAATTTTTGGTATTAATTTCAGCCCAGATACACTGAATGATAATGATAGA
AATCAGTATAACATACCGTTTGGTTATAAAGCATGGCTGATTGTTCCCGGAACAGAATCT
GAATCTGGTACAGTAGATGTTAGCAAAGTTTCGGAAAAAAACAAATATATCATTAAAAAC
AACACAAATAAAGTAATGGATGTTTGGGCAGATTATTGTGGAAGTTATAATAATAATAAA
TGCAGAGTACAGCTTATTACTCGACCGTATTCAGAAAAAAAGATAGAGATAGATAGTAAC
AATAATCCAATTGAATTTACTTTTTCTATTTATATCGGACGCGAACGAAAACTGATAAAA
AGAAAGATTTTATGA
>CS5_csfC LR883008 ~~~csfC~~~CS5 CsfC outer membrane usher~~~
ATGACTCTAAAAGACACCTTATTTTTTTTATCTATCAGTATTTATTGCAGTCAATCTCTA
GCTGATAAAAGTGAGCTGGCTATACTCAGTCCTAATAAATCAACCACGGATTTGGTTCTG
GCAGGAGGAATTAACCTGAACGTTTCCCGTTATGCAGGAATAATTACGCCTGAATCTGGT
ACAGTCAAGGTGCTTTTTGATGGAGTAACTGAGAGCACTCTGAATGCCAAAATATCCTTG
GATACAGTGCAGTTTCAAGACAATGTACAGTTTGAATCATTTCTGAAAAATGTCGGTATT
CGTGAGAAATATATTGAAAAAATCTTGAACCAGAATACCCGAGTCGGTTTCGTCCATTCC
CAAGGGTGTCAGGGACCTCGAAGTGAATGTATTGTAGTTAGTAAAGGAATTGACTTTGTC
GTGGATTACTATAACCAAACAATTCGACTGTTTGTTGCGCCAGAATTGTTAGGGAAGAGC
GTTGGAGAGAATTCATATCTCACGCTTAATGGCGAACTTGGGATTATTAATAACCTATCA
GGTTACTATTACGAAACTTTTGGTCGCTATGACCCGACATACTATATTCGTGATCAAGGG
GTGGTTGGGGCAGGTGCGGGATTTATTCGCTATAATATTTACCGTTCAGATTATCAGAAT
AATGTGGATGAACTCTATTACAGCCGTGCCTTGATAGCCGATAATAAAATACTGGTTGGC
AGGACGCAAAGTAACGGTAACTTTAATCCATCCAGCGCTCAGTCTATTTTTTCTGATATT
TCAGTCACTGGGATACGATTTGGAACAGCCGAGGAGTTGGTTGATCGCAGTTATGGAAAG
AAAACATTTAGTTATTACAGCCCGTCAACTGGAATAGTGGAAGTAAGTAAAGATAATATT
CTGGTGTACGCTATTGCTACGCAGGCAGGATATGGCGAAATCAATTTAGCAAATTTGCCT
TATGGGCAGTATAATGCTTTGGTTCAGGTAAAATCGTCTTCCGGAATAGTTGTCTCATCG
CAGAATGTGCTGATTAACAATACAGGTTCATTCAACAGTGACTTCTCTTGGCATCTCTTT
GTAGGGAATAGTGGTTCTTCTGACAATGAATTTGTCAGAAAAAATACCGAAGTTATTGAG
AGCGGTGTTCAACTTCCTGTAAATACACTTACTGCTCTTTACGTAGGAGGTGCTAAGGTT
GATAAAAATACGATTTACAGTACTGGTTTAATGTTCCAGAAAGAACCGATTTCTGTTTCA
CTAAAAATGGGGGGGGGCAGGGATTTAGACATTACGAAATGAAAAGTTATCTTGAAAGA
CTGTCACTCTCATGGAAAAAGACAAGTACAGGTAAAAACTGGAATGGCCTAAAATCCAGC
ACAGATAATACTACACTATCAGCCGGCTATAACTTCAATGTGATGTCGAATGTGTCTGCT
AATGTTGGATATATATATTCCTCGAGCATGAGGCCAGATTACTTTTACGCTAATACTGAC
CATTTGGGTATGGAGTCAGAGTTCAGATATAAAAAAAACAACTATTCAAACAAGAATCTT
TATGCCAATATGTATTACAATTTTCCTGGAGGCAATAGTTTATATCTAAACACCTACAAG
GAATTAAGAGGAAATGATTACAGCGTTTCTTTGGGGATGAATATATCTTTGGGTAAAAAC
TCACGTTTTAACAGCTCATTCTATAAAAACGGAGCAGATATAACAAATAGTAGCACTGTG
GATTATGCAAAAAGGCTTTCTGATAACTGGTCACACTCAGTATCAGTAGGAAGATATTTT
TCTAATGATAGTTATAATTCTGCCACATACAGTCTCTCCCATAATAGCAATGAAGTGAGG

GGGGCAGGTTATTACTATGCTACTGATAATGGACAGAGTCAACTTACGTTGACAGCAGAT
AGCACTCAGATTATTAACAGTAATGGGATATATTTACTTCGTCTTCATGGAAGGATAAT
GCTTTTATTATTCGAGGAAAGGACGCCAAATATGATATTTCCGTCAGGAATATGACTGAT
AATACCACGCGTTATTTTGATTCCGACACAAATATTATCAGTGTGCCTGTATATAATAAA
GTAATGGTGAACAGTGACACATCTGGGTCAAACTTGATTTTTGAAAATTATCAGACCAAG
AAAAGTCGTAGTTTTGCGCTTGTACCAGGGTCAACGGTAATGGTTTCAGACAAAACTATC
AGCGCGAACTCCGTCATTGTCACACTGAAAAACAGTAATAATCAGTATGCCCGAACGGCA
TTTTGCAACGGAGACAGTTGTATAGCAGTTTCTCGCCTGAATCAGGGAGTATTTCGAGTG
AAATATACGGGGGATTCACTCACGCTGCGTTCGGAGGGGGAGCAGTGTTCGACATCTGAA
ATTAATAAAAGGAAATATGTAAGCATTACCTGTCAAAAAATATAA
>CS5_csfE LR883008 ~~~csfE~~~CS5 CsfE second minor subunit~~~
ATGAAAATAAAAAAATTCAAACTTGTCTTCTATATGGTAATTTTTTATGTAATCAGTCTG
CAAGAAGTGCTATCAGCCAGCACGTCTGTGATTACTAATAACGGACAAACGATAACTCTT
ACGCTTCCTGTGAGGGCAACCATTACAGCAGATAGTATTCTACGTGACACTATATTGGTT
AAGCCCTTGTCATCCCTTTATGATGTTGTTACATGGGATTCGGAGAATAACAGATTTAAA
AATCATGAATTCCTTGTTAGGGTGATTAAAGAAACAGCTGTACCTATTTCATTTGAGGTT
ATTAATGATCAATATACCTGTAGTTATAATAACCCTGACAGGATGTCTCCCCTGCCGACA
GATATTGCCATCGCGAACTCTGATTATAAATACAGTGTTTCATGGTCAGGTGGATATGTT
GATATGGGAAAGGGACGCGCAGCTACTGTAAATGACAGTCACTCGTGGCTATCTTCTGTC
AATGGTGTTGACAGATATTTAGACTTAACGCTAAATATAAACTTTCCTGATATGACTCCA
TATACTCAGTTGCTAAATCGTGGAGGACTATGTAGGGGGAGTATAACTATGCTGCTAAGT
AATAAATTATAA
>CS5_csfF LR883008 ~~~csfF~~~CS5 CsfF chaperone for CsfE~~~
ATGTTTTTTTTTAGAGCGATTTTAATCGTTTTTTTTATCGGATCAGATGTTCAAGCTTTT
CAGATAGATACGTTAACCAAGGTGATAGATAAAGACACCCAATACATTGAAATAACGGGG
GAATACGAACGGGAATATATTTATACACAATTAACACAACTTCTTACTGATAAAAAACAT
GGACTCCGTGAGATTCCTTTTAATCCGGAGGATATATCTTCATGGCCTATTATTGTAGAA
CCTGGAGAAATTGTGCTTGATAAGGCGGATAAAATCAGAGTTAAAATCATCCGAAACGGT
CCGCAACAGGATGAGGATCGCGTGTCAGGTCTAGCATTTATACCCGAAAAGGTGCGCAGG
AAGAAAATCCAAGATTCAGGCCTTCAAATATCGGTAGGATATAAGGTATGGCTGTTTATA
CCCGGCAAATCTCCTTTAAAGGGACAGATAAAAGCCTCCAAAAAAAGTGGAAATATAACA
ATTGAAAATATGACCAATAAAATATTAAGAATTGTTCCTGATGATTGTTCAGGAAAAAAT
AAATCTGAATGTGCCGGAGCGGTAATTCTATTACCATATACTAGTAAGCAGATTGATGAC
TCAGAGCATGTACAGACGCTTAGTATTTATCTAATTAATGATTTACATAAAAAAATAAAG
GTAATCACATTATGA
>CS5_csfD LR883008 ~~~csfD~~~CS5 CsfD second minor subunit~~~
ATGAAAAAACAACCTATTGTATTAACTCTAGGCTTTTTTTCCTTTATGGTTCAGGCTGCT
ACAACAGTTACTTCAGAATTTGAAATTACTAATAAAACTATCGAAAAATATACAATATCA
AGTACAGATAGTACTATGACATATACTGATGTATCAGGGAGTGGTTTATATAAAATATCA
GACCAGTATTCAGATGCCAATGTCAATATTAGAAATTACGGCAATCATCAGTTTGGATTG
CTCAGAAATAACAGCACTGTTAATATTATCATGAAGGGCGTAAACTTAGGCCACACTTTT
ACTGTACAAGGAAAATATGCCAATTCAGCCGTGTCAGTTCCCAATCCTCAAAAATCTTTT
ACCGTTAGAAGTAATAATGGATGCTCAAGTGTATCTTCTGCATATCTAGGTAATGCGAGT
TATACGCTATACGAAATAAGATCTAGTAATGATGTTACACGGAACTGTTCCGGACAAACG
GATCAGTACACTCATATGCCAAATAATAGTGGTCAGGTAAATGTTACAGGAATTTACAGA
GATTTCTACTTGGATATTGGTCGACTGCAATCAGACGCTGAGTATAGGAAAGCACCTCCT
GATACCTATATAGGAACAGGGACATTCGCTGGAGAGGTTTTAAAGAATCGAGTAGGTTCT
GGTTATACTCCGACTTATACAAACAAAATAACAATTACAAAAAAACCATATTTTGAAAGT
GTGACATTGCCCACGGTAGATAATATCTTCGATACTCGTACTATCGGCAGACAGATTCAG
GGGAATCTTGTAATTCCATTTGTGATTAATGGGCATTTCACACCATACAATACTATTTCG
TTGCAGGTCATTTCACTAAATGGGTTTAAGTTACAAAGTGAGAATGTTGGTTCCTCAGCA
ACCATTCCTTATTCGCTAAATATGACGATAGGTAGTGAACGACGTTATTCCTTGGCCACA
AATGGGAATGGTTTGGGAAATGTTACAATAAATAACCTCGAATCTGATGGCTATTCCATT
CAAGGACGCTTCAATGCAGATTTTTTGATCGATAAAAATACAGCTGTGACAGGAGATTAT
GCCGATACATTGACAGCAATATTTCAGATTTCGCTACTATAA
>CS6_cssA2 LR883014 ~~~cssA2~~~CS6 CssA2 major subunit A subtype A2~~~
ATGAAGAAAACAATTGGTTTAATTCTAATTCTTGCTTCATTCGGCAGCCATGCCAGAACA

GAAATAGCGACTAAAAACTTCCCAGTATCAACGACTATTTCAAAAAGTTTTTTGCGCCT
GAACCACAAATCCAGCCTTCTTTTGGTAAAAATGTTGGAAAGGAAGGAGGTTTATTATTT
AGTGTGAGCTTAATTGTTCCTGAAAATGTATCCCAGGTAACGGTCTACCCTGTTTATGAT
GAAGATTATGGATTAGGACGACTCGTAAATACCGCTGATGATTCCCAATCAATAATCTAC
CAGATTGTTGATGATAAAGGGAAAAAAATGTTAAAAGATCATGGTACAGAGGTTACGCCT
AATCAACAAATAACTTTTAAAGCGCTGAATTATACTAGCGGAGATAAAGAAATACCTCCT
GGGATATATAACGATCAGGTTATGGTTGGTTACTATGTAAACTAA
>CS6_cssA2 LR883014 ~~~cssA2~~~CS6 CssB2 major subunit B subtype B2~~~
ATGTTGAAAAAAATTATTCCGGCTATTGCATTAATTGCAGGAACTTCCGGAGTGGTAAAT
GCAGGAAACTGGCAATATAAATCTCTGGATGTAAATGTAAATATTGAGCAAAATTTTATT
CCAGATATTGATTCCGCTGTTCGTATAATACCTGTTAATTACGATTCGGATCCGAAACTG
AATTCACAGTTATATACGGTTGAGATGACGATCCCTGCAGGTGTAAGCGCAGTTAAAATC
GTACCAACAGATAGTCTGACATCTTCTGGACAGCAGATCGGAAAGCTGGTTAATGTAAAC
AATCCAGATCAAAATATGAATTATTATATCAGAAAGGATTCTGGCGCTGGTAAGTTTATG
GCAGGGCAAAAAGGATCCTTTTCTGTCAAAGAGAATACGTCATACACATTCTCAGCAATT
TATACTGGTGGCGAATACCCTAATAGCGGATATTCGTCTGGTACTTATGCAGGACATTTG
ACTGTATCATTTTACAGCAATTAA
>CS6_cssC LR883014 ~~~cssC~~~CS6 CssC chaperone~~~
ATGAAATCAAAGTTAATTATATTATTGATGTTTGTGCCATTTTCATCTTTTTCAACAGGA
AATAATTTTGAAATAAATAAGACACGAGTAATTTACTCTGACAGCACACCATCAGTTCAA
ATATCAAATAATAAAGCATATCCTTTAATTATCCAAAGCAATATATGGGATGAAAACAAT
AATAAAAATCATGACTTTATAGCAACACCACCGATTTTTAAAATGGAAAGTGAAAGCAGG
AACATAATAAAAATAATCAAAACAAATATTAAATTGCCGGACTCTCAGGAAAGTATGAGA
TGGTTATGTATTGAATCAATGCCACCAACAGAAAAAAGTACTAAAATAAACAGAAAAGAA
GGAAGGACAGACAGTATTAATATCAGCATTCGGGGATGCATTAAACTGATATATCGACCT
GCCAGTGTTCCGTCTCCTGTTTTTAATAATATAGTGGAAAAATTAAAATGGCATAAAAAT
GGAAAGTATCTTGTATTAAAAAATAATACACCCTATTACATTAGCTTTTCTGAGGTTTTT
TTTGATTCAGATAAAGTAAACAATGCAAAAGATATTTTATATGTAAAACCATACTCAGAG
AAGAAAATAGATATCAGCAACAGAATAATAAAAAAAATCAAATGGGCAATGATTGATGAT
GCTGGCGCAAAAACAAAACTTTATGAATCAATTTTATAA
>CS6_cssD LR883014 ~~~cssD~~~CS6 CssD outer membrane usher~~~
ATGATGCTGGCGCAAAAACAAAACTTTATGAATCAATTTTATAAAAAATCTCATTATAGT
ATACAAAAACATCAGATTACAGACTTGCTTTTTTTTCTATTTCTATATCCTTTTTCAACC
TCATATGGAAATGAACAATTTAGTTTTGACTCACGATTCCTACCATCAGGTTATAATTAC
TCTTTAAATAGTAACTTACCTCCTGAAGGTGAGTATCTGGTTGATATTTATATTAACAAA
ATAAAAAAGGAGTCCGCGATTATTCCTTTTTATATAAAAGGAAATAAACTTGTACCATGT
TTATCAAAAGAAAAACTTTCATCTTTGGGTATCAACATTAATAATAACGACAACGCAGAG
TGTGCAGAAACAAGTAAGGCAGGTATTAGTAATATCAGCTTTGAGTTTAGCTCCCTTCGT
TTGTTTATTGCTGTACCGAAAAATCTTCTGTCTGAGATTGATAAAATATCATCAAAGGAT
ATAGATAACGGGATCCATGCTTTATTTTTTAATTATCAAGTAAATACAAGGCTAGTTAAT
AATAAAAATCGTTATGATTACATTTCTGTTTCACCAAATATAAATTATTTTTCATGGCGG
TTGCGTAATCGTTTTGAATTTAACCAAAACAATGATAAAAAAAACATGGGAAAGAAACTAC
ACTTATCTAGAAAAAAGTTTTTATGATAAAAAGCTAAACTTAATCGTTGGTGAAAGTTAT
ACGAGTTCAAATGTTTATAATAACTACTCTTTTACTGGTATTTCAGTTTCTACAGATACA
GATATGTATACGCCAAGTGAAATCGATTATACACCAGAAATTCATGGAGTGGCTGATTCA
GACTCTCAGATTATTGTTAGGCAAGGCAACACCATTATCATTAATGAAAGTGTTCCAGCC
GGACCGTTCTCATTTCCAATAACCAATCTCATGTATACTGGGGGGCAACTTAATGTGGAG
ATAACAGATATTTATGGAAATAAAAAACAATATACTGTCAGTAATTCCTCTCTTCCTGTT
ATGAGAAAAGCGGGACTAATGGTATATAATTTTATATCTGGAAAATTAACAAAAAAAAAT
AGTGAGGATGGTGATTTTTTTGCCCAAGGTGATATTAACTACGGTACTCACTATAACAGC
ACACTATTCGGTGGGTATCAGTTTAGTAAAAATTATTTTAACTTATCTACTGGTATAGGC
ACTGATCTGGGATTTTCTGGAGCATGGCTACTAAACGTTAGCAGAAGTAATTTTAAGGAT
AAAAATGGATATAATATTAATCTACAACAAAACACTCAGTTAAGACCATTCAATGCCGGG
GTTAATTTCGATTACATATACAGAAAAAAAGGGTATGTGGAACTTTCCGGCATTGGCTGG
CATGGTAATTTATATAATCAACTTAAAAATAGTTTTTCTTTATCTTTGTCAAAATCATTG
GATAAATACGGAAATTTCTCACTTGATTATAACAAAATAAAATACTGGGATAATGCGTAT
GATAGTAACTCAATGTCGATTCGTTATTTTTTTAAATTCATGCGAGCAATGATTACAACA

AATTATTCTTTGAATAAATATCAATCTTATGAAAAAAAAGATAAAAGATTTAGTATTAAT
ATATCATTGCCTTTAACCAAAGATTACGGGCACATATCTTCAAACTATTCATTTTCCAAT
GCAAATACAGGAACGGCAACCAGTTCTGTAGGTGTAAACGGTAGTTTTTTAATGACGCA
AGATTAAACTGGAACATTCAGCAGAACAGAACGACCCGTAACAATGGATATACTGATAAT
ACCAGTTACATAGCAACCAGCTATGCCTCTCCCTATGGCGTTTTTACTGGTTCATATTCA
GGATCGAACAAGTATTCAAGCCAGTTCTATTCCGCATTGGGAGGTATTGTTTTGCATAGC
GATGGCGTAGCTTTTACTCAAAAAGCCGGAGATACCTCTGCTCTTGTCCGTATTGATAAT
ATTTCTGATATAAAAATTGGTAACACTCCTGGTGTTTATACTGGGTATAATGGTTTTGCT
TTAATTCCTCATCTTCAGCCGTTCAAAAAAAACACCATTTTAATTAATGATAAAGGAATT
CCAGACGATATTGCTCTTGCTAATATAAAAAAACAAGTTATCCCATCACGAGGAGCTATT
GTTAAAGTAAAATTTGATGCTAAAAAAGGCAATAACATTTTGTTTAAGCTTACAACTAAA
GATGGAAAAACGCCCCCATTAGGAGCTATAGCCCATGAAAAAAATGGAAAACAGATTAAT
ACGGGTATCGTTGACGATGATGGTATGCTTTATATGTCTGGATTATCAGGGACAGGGATT
ATTAATGTAACATGGAATGGAAAAGTCTGTTCATTTCCTTTTTCAGAAAAAGATATATCT
AGCAAACAATTATCTGTTGTAAATAAACAATGTAACCGCCCCGGAAATTCTGGAGACTAA
>CS6_cssA3 LR883008 ~~~cssA3~~~CS6 CssA2 major subunit A subtype A3~~~
ATGAAGAAAACAATTGGTTTAATTCTAATTCTTGCTTCATTCGGCAGCCATGCCAGAACA
GAAATAGCGACTAAAAACTTCCCAGTATCAACGACTATTTCAAAAAGTTTTTTGCGCCT
GAACCACAAATCCAGCCTTCTTTTGGTAAAAATGTTGGAAAGGAAGGAGGTTTATTATTT
AGTGTGAGCTTAACTGTTCCTGAAAATGTATCCCAGGTAACGGTCTACCCTGTTTATGAT
GAAGATTATGGGTTAGGACGACTCGTAAATACCGCTGATGATTCCCAATCAATAATCTAC
CAGATTGTTGATGATAAAGGGAGAAAAATGTTAAAAGATCATGGTGCAGAGGTTACGCCT
AATCAACAAATAACTTTTAGAGCGCTGAATTATACTAGCGGAGATAAAGAAATACCTCCT
GGGATATATAACGATCAGGTTATGGTTGGTTACTATGTAAACTAA
>CS6_cssB1 LR883008 ~~~cssB1~~~CS6 CssB1 major subunit B subtype B1~~~
ATGTTGAAAAAAATTATTCCGGCTATTGTATTAATTGCAGGAACTTCCGGAGTGGTAAAT
GCAGGAAACTGGCAATATAAATCTCTGGATGTAAATGTAAATATTGAGCAAAATTTTATT
CCAGATATTGATTCCGCTGTTCGTATAATACCTGTTAATTACGATTCGGATCCGAAACTG
AATTCACAGTTATATACGGTTGAGATGACGATCCCTGCAGGTGTAAGCGCAGTTAAAATC
GTACCAACAGATAGTCTGACATCTTCTGGACAGCAGATCGGAAAGCTGGTTAATGTAAAC
AATCCAGATCAAAATATGAATTATTATATCAGAAAGGATTCTGGCGCTGGTAAGTTTATG
GCAGGGCAAAAAGGCTCCTTTTCTGTCAAAGAGAATACGTCATACACATTCTCAGCAATT
TATACTGGTGGCGAATACCCTAATAGCGGATATTCGTCTGGTACTTATGCAGGACATTTG
ACTGTATCATTTTACAGCAATTAA
>CS6_cssA7 LR882991 ~~~cssA7~~~CS6 CssA7 major subunit A subtype A7~~~
ATGAAGAAAACAATTGGTTTAATTCTAATTCTTGCTTCATTCGGCAGCCATGCCAGAACA
GAAATAGCGACTAAAAACTTCCCAGTATCAACGACTATTTCAAAAAGTTTTTTGCACCT
GAACCACGAATACAGCCTTCTTTTGGTGAAAATGTTGGAAAGGAAGGAGCTTTATTATTT
AGTGTGGACTTAACTGTTCCTGAAAATGTATCCCAGGTAACGGTCTACCCTGTTTATGAT
GAAGATTATGGGTTAGGACGACTAGTAAATACCGCTGATGCTTCCCAAGCAATAATCTAC
CAGATTGTTGATGAGAAAGGGAGAAAAATGTTAAAAGATCATGGTACAGAGGTTACACCT
AATCAACAAATAACTTTTAAAGCGCTGAATTATACTAGCGGGGAAAAAAAAGTATCTCCT
GGAATATATAACGATCAGGTTATGGTTGGTTACTATGTAAACTAA
>CS6_cssB3 LR882991 ~~~cssB3~~~CS6 CssB3 major subunit B subtype B3~~~
ATGTTGAAAAAAATTATTTCGGCTATTGCATTAATTGCAGGAACTTCCGGAGTGGTAAAT
GCAGGAAACTGGCAATATAAATCTCTGGATGTAAATGTAAAAATTGAGCAAAATTTTATT
CCAGATATTGATTCCGCTGTTCGTATAATACCTGTTAATTACGATTCGGACCCGAAACTG
GATTCACAGTTATATACGGTTGAGATGACGATCCCTGCAGGTGTAAGCGCAGTTAAAATC
GCACCAACAGATAGTCTGACATCTTCTGGACAGCAGATCGGAAAGCTGGTTAATGTAAAC
AATCCAGATCAAAATATGAATTATTATATCAGAAAGGATTCTGGCGCTGGTAACTTTATG
GCAGGACAAAAAGGATCCTTTCCTGTCAAAGAGAATACGTCATACACATTCTCAGCAATT
TATACTGGTGGCGAATACCCTAATAGCGGATATTCGTCTGGTACTTATGCAGGAAATTTG
ACTGTATCATTTTACAGCAATTAA
>CS7_csvA LR882979 ~~~csvA~~~CS7 CsvA major subunit~~~
ATGAAGAAAAAAATTACTGATAGCTTCAGTATTAGCAATGGCAACCGTATCAGGTTCTGTT
TTGGCTGCTGTTACAAATGGCCAGCTCACATTTAATTGGCAGGGAGTGGTTCCTTCTGCT
CCCGTTACTAAAAATACGTGGGCTTTTGTGAACGGATTGGATATACCGTTTACTCCTGGT

ACTGAACAGTTGAATATCACCCTTGGTGCAGATAAAGGTATCACAGCCCGTTCGGTTAAG
CCTTATGATTTTTCATTGTTCCAGTTACTGGAACAGTAACTGCTGGATCGCCGGTTACA
CGTAGTGATACGACATCTATGAATAGTGTGAAAGCTTTTCTATCAAGTGAACCCGTTTCT
AATGGTTTTGTTGGCAACAAGCAGTTAACCCTGAGCACCACAGCAGAAGCAGTTACGGGG
CAAGTCGCAATCACTTTAAATGGTCAGCCGCTTAAAGTGGGGAGTGCTAATGCAACAACT
GTTGCTATGGATACCAATAAAAAAGAGTCTCATATTTCTATTGATATGAATGCCAAGGCA
AGTGCTTCGGATGTGGCGGAGGGTTCAGCTATTAACTTTGTAGCTCCGGTAACATTTGCT
GTTGATATTTAA
>CS7_csvB LR882979 ~~~csvB~~~CS7 CsvB chaperone for CsvA~~~
ATGAAAATACTGTATTCTTTCTTGTTGTTACCTTTTTTTTCTTGCGCCTTTAGTGTTGAT
TCAATGATAAAATTTTCAGGCAAAGATGACTTTTTTCTTGTAAATGGAAACAGCAAGGAA
AGAGAGTATATTTATATAACACTTTCTGAACTAATTAGCGAGAAAAATAATAGGCACGGT
GAAATATTTTACAACGCAGACAATGTTCCTCTATGGCCGATATCTGCAGAACCTTCAGAT
ATTATCATTTCATCCGGTGAGCAAATCAAAATAAAAATCAACAAAAATTATACTCCTGTC
GGTGGGGATCGCATTTTTGGTATTAACTTCAGCCCAGACACACTGAATGATACTGATAGA
AATCAGTATAGCATACCGTTTGGTTATAAAGCATGGCTGATCGTTCCCGGAACAGAGCCT
GAATCCGGTACGGTAGATGTTAGCAAAGTTTCGGAAAAAAATAAATATATCATTAAAAAT
AACACAAATAAAGTAATGGATGTTTGGGCAGATTATTGTGGAAGTAATAATAATAAATGC
AGAGTGCAACTTATTACTCGACCTTATTCAGAGAAAAAAATAGAGATAGATAGCAACAAT
AATCCTATTGAATTTACTTTTTCTGTTTATATCGGACATGAACGAAAACTGATAAAAAGA
AAGATTTTATGA
>CS7_csvC LR882979 ~~~csvC~~~CS7 CsvC outer membrane usher~~~
ATGACACTAAAACACACCTTATTTTTTTATCTATCAGTATTTATTGCAGTCAATCTTTA
GCTGATAAAAGTGAGCTGGCTATACTCAGTCCTAATAAATTAACCACGGATTTGGTTTTG
GCAGGAGGAATTAGCCTGAACGTTTCCCGTTATGCAGGAATAATTACGCCTGAACCTGGT
GCAGTCAAGGTGCTTTTTGATGGAGTAACTGAGAGTACGCTAAATGCCAAAATATCCCTA
GATACAGTGCAGTTTCAAGACAATGCACAGTTTGAATCATTTTTGAAAAATGCCGGTATT
CGAGAGAAATATATTGAAAAAATCTTGAACCAGAATACCCGAGTCGGTTTCGTCCATTCC
CAAGGATGTCAAGGACCTCGAAGTGAATGCATTGTAGTGAGTAAAGGGATTGACTTTGTC
GTGGATTACTATAACCAAACAATTCGGCTGTTTGTTGCGCCAGAATTGTTAGGGAAGAGC
GTTGGAGAGAATTCATACCTCACGCTTAATGGCGAGCTTGGGATTATTAATAACCTATCA
GGTTACTATTACGAAACTTTTGGTCGCTATGACCCGACATACTATATTCGTGATCAAGGG
GTGGCTGGGGCAGGTGCTGGATTTATTCGCTATAATATTTACCGTTCAGATTATCAGAAT
AATGTGGATGAACTCTATTACAGCCGTGCCTTGATAGCCGGTAATAAAATACTGGCTGGC
AGGACGCAGAGTAACGGCAACTTTAATCCATCCAGCGCTCAGTCTCTTTTCTCTGATATT
TCAGTCAATGGGATACGATTTGGAACAGCCGAGGAGTTGGTTGATCGCAGTTATGGAAAG
AAAACATTTAGTTATTATAGCCCGTCTGCTGGAATAGTGGAAGTAAGTAAAGATAATATT
CTGGTGTACGCTATTGCTACGCAGGCAGGATATGGAGAAATCAATTTAGCAAATTTGCCT
TATGGGCAGTATAATGCTTTGGTTCAGGTAAAATCGTCTTCCGGAATGCTTGTCTCATCG
CAGAATGTGCTGATTAACAATACAGGCTCATTCAACAGTGACTTTTCTTGGCATCTCTTT
GTAGGAAACAGTGGCTCTTCTGACAATGAATTTGTCAGAAAAAAATACTGAAGTTATTGAG
AGCGGTGTTCAACTTCCTGTAAATACACTTACTGCTCTTTACGTAGGAGGTGCTAAGGTT
GATAAAAATACGATTTACAGTACTGGCTTAATGTTCCAGAAAGAACCGATTTCTGTTTCC
CTAAAAATGGGGGGGGGGGATCAGGGATTTAGCCATTACGAAATGAAAAGTTACCTTGAG
AGACTGTCACTCTCATGGAAAAAGACAAGTACAGGTAAAAACTGGAATGAGCTAAAATCC
GGCACAGATAATACTACACTATCAGCAGGATATAACTTCAATGTGATGTCGAATGTGTCT
GCTAATGTTGGATATATATATTCATCAAGCATGAGACCAGATTACTTTTACGCTAATACT
GACCATTTTGGTATGGAGTCAGAGTTCAGATATAAAAAAAACAACTATTCAAATAAGAGT
CTTTATGCCAATATTTATTACAATTTTCTTGGGGGCAATAGCTTATATCTGAACACCTAC
AAGGAATTAAGTGGAAATGATTACAGCGTTTCTTTGGGGATGAATATATCTTTGGGTAAA
AACTCACGCTTTAACAGTTCATTCTATAAAAACGGAACGGCTATAACAAATAGTAGCACA
GTGGATTATGCAAAAAAGCTTTCTGATAACTGGTCACACTCAGTATCAGTAGGAAGATAT
TTTTCTAATGATAGTTATAACTCTGCCACATACAGTCTTTCCCATAATAGCAATGAAGTG
AGGGGTTCAGGTTATTACTATGCTACTGATAATGGACAGAATCAACTTACGTTGACAGCA
GATAGTACTCAGATTATTAACAGTGGTGGAATATATTTACTCCGTCTTCATGGAAGGAT
AATGCTTTTATTATTCGAGGAAAGGACGCCAAATATGATATTTCCGTCAGAAATATGACT
GATAATACCACGCGTTATCTTGATTTCGACACGAATATTATCAGTGTGCCTGTATATAAT

AAAGTAATGGTGAACAGTGACACTTCTGGGTCAAACTTGATTTTTGAAAATTATCAGACC
AAGAAAAGTCGTAGTTTTGCGCTTGTACCAGGGGCAACGGTAATGGTTTCAGACAAAACC
ATCAGCACCAACTCCGTCATTGTCACACTGAAAAACAGTAATAATCAGTATGCCCGAACG
GCATTTTGCAACGGAGACAGTTGCATAGCCGTTTCACGCCTGAATCAGGGAGTATTTCGA
GTGAAATATACGGGGAATTCACTCACGCTGCGTTCGGAGGGGGAACAGTGCTCGGCATCT
GAAATTAATAAAAGGAAATATGTAAGCATTACCTGTCAAAAAATATAA
>CS7_csvE LR882979 ~~~csvE~~~CS7 CsvE minor subunit~~~
ATGAAAATAAAAAAAATTAAATTTGCCTTCTGTATGACAATTTTTTCGGTAATCAGCCCA
CAGGAAGTGCTATCAGTCGGCACGTCTGTGATAACTAATAATGGACAAACGATAACTCTT
ACACTTCCTGTGAAGGCAACCATCACGGCAGATAGTATTCTACGTGACGCTATATTGGTT
AAGCCGTTGTCATCCCTTTATGATATTGTTACATGGGATTCGGAGAATAACAGATTTAAA
AATCATGAATTCCTTGTTAGGGTGATTAAAGAAACAGCTGTGCCTATTTCATTTGAGGTT
ATTAATGATCAATATACTTGTGGTTATAATAACCCTGATAGGATGTCTCCCCTGCCGATA
GATATTGCTATTGCGAACTCTGATTATAAATACAGTGTATCATGGTCAGGTGGATATGTT
AATATGGAGAAGGGACGTGCTGCTATTGTAAATGACAGTCACTCGTGGCGATCTTCAGTT
AATGGAGTTGACAGATATTTAGACTTAACGTTAAACATAAACTTTCCAGATATGACTCCA
TATACTCAGTTGCTAAATCGTGGAGGGATATGTAGGGGGAGTATAACCATGCTGCTAAGT
AATAAATTATAA
>CS7_csvF LR882979 ~~~csvF~~~CS7 CsvF chaperone for CsvE~~~
ATGTTTTTTTTTAGAAACGTGATTTTAATTGTTTTTTTTATCGGATCAGATGTGCAAGCT
TTTCAGATAGATTCGTTAACTAAGGTGATAGATAAAGACACTCAATATATTGAAATAACA
GGGGAATACGAACGAGAATATATTTATACACAATTAACACAACTTCTTACTGATAAAAAA
CATGGACTCCGTGAGATTCCTTTTAATCCGGAGGATATATCCTCATGGCCTGTTATTGTA
GAACCTGGAGAAATTGTGCTTGATAAGGCGGATAAAATCAGAGTTAGAATCATCCGAAAC
GGCCCGCAACAGGATGAGGATCGCGTGTCAGGTCTAGCATTTATACCCGAAAAGGTGCAC
GGAAAGAAAATCCAAGATTCAGGCCTTCAAATATCTGTAGGATATAAGGTATGGCTGTTT
ATACCCGGCAAATCCCCTTTAAAGGGACAGATAAAAGCCTCCAAAAAAAGTGGAAATATA
ACAATTGAAAATATGACCAATAAAATATTAAGAATTGTTCCCGATGATTGTTTAGAAAAA
AATAAATCTGAATGTGTCGGGGCGATAATTCTATTACCACAGGCTAGTAAGCAGATCGAT
AACTCAGAGCATGTACATACGCTTAGTATTTATCTAATTAATGATTTACATAAAAAAATA
AAGGTAATCACATTATGA
>CS7_csvD LR882979 ~~~csvD~~~CS7 CsvD second minor subunit~~~
ATGAAAAAACAACCTATTGTATTAGCCATAGGCTTTTTCCCCTTTATGGTTCAGGCTGCT
ACAACAGTTACTTCAGAATTTGAAATTACTAATAAAACTATCGAAAAATATACAATATCA
AGTACAGATAGTACTATGACATATACTGATGTATCAGGGAGTGGTTTATATAAAATATCA
GACAGGTATTCAGATGCCAATGTCAATATTAGAAATTACGGCAATCATCAGTTTGGATTG
CTCAGAAATAACAGCACTGTTAATATTATCATGAAGGGCGTAAACTTAGGTCACACTTTT
ACTGTACAAGGAAAATATGCCAATTCAGCCGTGTCAGTTCCCAATCCTCAAAAATCTTTT
ACCGTTAGACGTAATAATGGATGCTCAAGTGTATCTTCTCAATATCTAGGTAATGCGAGT
TATACGCTATACGAAATAATATCTAGTAATGATGTTACACGGAACTGTTCCGGACAAACG
GATCAGTACACTCATATGCCAAATAATAGTGGTCAGGTAAATGTTACAGGAATTTACAGA
GATTTCTACTTGGATATTGGTCGACTGCAATCAGACGCTGAGTATAGGAAAGCACCTCCT
GATACCTATATAGGAACAGGGACATTCGCTGGAGAGGTTTTAAAGAATCGAGTAGGTTCT
GGTTATACTCCGACTTATACAAACAAAATAACAATTATAAAAAAACCATATTTTGAAAGT
GTGACATTGCCCACGGTAGATAATATCTTCGATACTCGTACTATCGGCAGACAGATTCAG
GGGAATCTTGTAATTCCATTTGTGATTAATGGGCATTTCACACCATACAATACTATTTCG
TTGCAGGTCATTTCACTAAATGGGTTTAAGTTACAAAGTGAGAATGTTGGTTCCTCAGCA
ACCATTCCTTATTCGCTAAATATGACGATAGGTAGTGAACGACGTTATTCCTTGGCCACA
AATGGGAATGGTTTGGGAAATGTTACAATAAATAACCTCGAATCTGATGGCTATTCCATT
CAAGGACGCTTCAATGCAGATTTTTTGATCGATAAAAATACAGCTGTGACAGGAGATTAC
GCCGATACATTGACAGCAATATTTCAGATTTCGCTATGA
>CS8_cofR AB049751 ~~~cofR~~~CS8 CofR regulator~~~
ATGAAGCTATATAATGAGCAGAAAACGTCTAAAAATACCCGTGTAGATTTTTTTATTAAA
AAACGAGGAATATTGCCAGGAACTATAGATGAAGAGCATTTCTGGTTGCTCATGGAGATA
TTACCTATCCATAGTGAAAAAGTTATCTGTGCTATGAGAGATCATCTTGTTTCGGGGGTA
TCCCGTAAGGATGTATGCCAAAAATATAGTGTTAATAGTGGTTATCTAAGTATCAGTATA
TCTAGGTTGAATTATGCACATCAGATAGTAATGAATATGGTTCATTACTATACCAAAACG

TAA
>CS8_cofS AB049751 ~~~cofS~~~CS8 CofS unknown function~~~
ATGAATAATTATTTAAATAATCACAATCTTCCACGTTTGTGTGAAGGGGGTATCTTAGAG
TGCGTCAATGTATCCAATGTTTATGTAGCATCATTCACGCTCGTCTATCTGCGGCATGGG
TGTGTTACAGTTACTGATGAGCGGGGGCGTGTATCACATTGTGTCGCACCAGGTATAATG
GTTTTTGAAAGAGACCAGTCTATAAACATTGTTATGCAGGAAGTTGATGGACACCTTTCC
TTTGATGTGGTCGAGGTTCCTCATCACTTGTTAACTGTTGCACATGAAGTACTAATTGAA
AAAATCCCCCAGACAGAATATCCGACGATAGAACATCCATTGCGGATAGTTTATACGGGA
GACTTTCCAGCCGGGGAAGAAGTCTTTGAGTTACTGAAAAATATGTTGTCAGAAAACCGG
ATGTCCATTAAAAATGAAGGAGAGATAAACATTGCAACATTCTCTTTGTTGTTGTTGCTT
TCAGGTTTTATTCATGAACCTGCATTTCCAGGGATCATCAGGAGGTCAGTAAAACTTAAC
GTAAAATATAAAGTTTATAATTTGCTTTATACAGAACCAGACAGACAGTGGAAACTGAAT
GACGTATCGTCTGCCATTTTTATGAGTGCTTCCACGTTGAAAAGAAAACTGGCTTCTGAG
GGTACGACATTCAGTGAATTATATATCACTGCAAGAATGAATCTTGCAAGCAAGCTTTTG
CGTACAGGTAAATACAGCGTGAGTAACGTGGCTGTGTTGTGTGGTTATGATAGTGTTTCT
TACTTCATTGCTTGTTTTAAGCGGCAGTTTAGTATCACGCCGTTTGCATTTATGAAATCA
GTGAATCACTGA
>CS8_cofT AB049751 ~~~cofT~~~CS8 CofT unknown function~~~
ATGCGTAAAAGAATCATTCTTCCGTTGTTCTGTTTACTGGTATGTAAAACGGCTTCCGCT
GATTGTTTTGAGCAGGCAGGGTATGACAGCAATATCGATCCTGATTTGTTAAGGGCAATT
GCCAAGGTGGAGTCCAACGACAATCATCTGGCGATAGGTAAAAATCCAGTAAATGGGTTT
GGTGTTGGACTAATGCAGATCGACTCTCAGAACTTTGAGCATCTGCAGAGGTTTGATATT
GCGCCAGAAATGCTGCTGGATGCCTGCATCAATGTGTATGCAGGTGCATATTTTCTCCGC
CTGGCGGTAAACCGGATGGGTCATAACTGGGACGCGATAGGAGCGTACAATGCTGGTTTT
TCAAAGACACCACGGCAGAAAAAACGACGTTATCAATATGCATCAAAAGTACGGCAACAG
TATCAAAGTATAAAGAAGAAGTGA
>CS8_cofA AB049751 ~~~cofA~~~CS8 CofA major subunit~~~
ATGCTTTCGGTTTATAACAGAACGCAAAAAATGAAAGAAGAGGCAAGAAAAAAACTGGC
C
AAGTATCATGAATTACGTAAACAGCGAGGTATGAGCCTTCTGGAAGTCATCATCGTTCTG
GGGATTATCGGAACAATTGCTGCGGGTGTGGTGATTCTGGCTCAACGAGCATTTGACTCA
CGTACTGTTTCTGAATTGGTCACTAATACGAATACTATTCGTGTTGCGATGAAAGATGCT
TATCAGCGTGACGGTAAGTATCCGGATTATCAAGCTCCATTAAGTCTTACTGCTGATTCA
ATTAAAACAGATTCAACAGGTATAGCGGTTGCGCAGTTAGTCCAATTAGGGAAACTAACC
CCTGATGAAGCCCGAAATGGTATTTCTGGGGACTATATTGGTATTGGTGGTGCAATAACA
TCTTCAGGTTCTACAATCAACAAGGGATTTGCAATGGAACTGAACGGACTTAGCCAAGAG
CAATGTCGTTCAATTCTTGGACAAGTTGGTGATAACTGGGAGTATGTGGCAGTTGGTACT
AGTCCTTCTGGTTCTTATGATGCTCTGTCTGCAGGCGCAGTAAACATGCTGGCTGCTACT
GATAATACTACAATATTACGTAGCCTGGCGGCTAATGGTCAAGTATCACTGACAGCTGAG
AAAATTTTAAAAACCTGCACAGCCACAGTTAACTCTATTACTTTGGCGAGCCGTTAA
>CS8_cofB AB049751 ~~~cofB~~~CS8 CofB unknown function~~~
ATGAATATGAGGGGTTTCACGCTTCTGGAAATGATTGTTACTCTGGCTGTTATGGGAGTT
GCAATGTTATCTGTCATTAAATATAAAGAGAAAGAAGCAGATGAAGCCAGACGACAAATT
GTATCTAATGCTCTGATTTCAGAAATCGCCGGCATTGTGGATTTTGTCGCAGAGGAACAA
ATAACCGTTATAGAACAGGGAATAGAAAAAGAAATTACGAATCCACTTTATGAGCAGAGC
TCTGGGATTCCATATATAAATCGAACTACAAATAAAGATTTAAACTCAACTATGTCAACA
AATGCCTCTGAGTTTATTAATTGGGGGGCTGGTACGTCGACGAGGATTTTTTTACGCGC
AAGTATTGCATAAGTACGGGAACTCAGGGGAACTACGAGTTTAGTAAGGATTATATACCG
TGTGAAGAACCAGCTATTTTATCAAATAGTGATTTAAAGATAGATCGTATTGATTTTGTT
GCTACAGATAACACAGTAGGGAGTGCAATAGAACGAGTAGATTTTATTCTTACTTTTGAT
AAAAGTAATGCTAATGAGAGTTTTTATTTCTCGAATTATGTAAGTTCTCTTGAAAAAGCT
GCTGAACAACACTCTATATCATTTAAAGATATTTATGTTGTTGAGCGTAATTCTTCAGGT
GCGGCAGGATGGAGGTTAACAACAATATCCGGAAAACCTTTAACATTCTCAGGTTTATCA
AAGAATATTGGTTCACTTGATAAAACTAAAAATTATGGACTACGATTAAGTATCGATCCT
AATCTTGGAAAATTTTTGCGTGCTGATGGTCGTGTCGGTGCTGATAAGCTTTGCTGGAAT
ATAGACAATAAAATGTCAGGACCATGTCTGGCTGCCGATGATAGCGGTAATAATCTTGTT
TTAACGAAAGGTAAGGGAGCTAAAAGTAACGAGCCGGGATTGTGTTGGGATCTTAATACG

GGTACAAGCAAGTTATGCCTTACACAAATAGAAGGTAAAGATAATAATGATAAAGATGCA
TCCTTGATAAAATTAAAAGATGATAATGGTAATCCAGCAACAATGCTAGCAAATATTTTG
GTAGAGGAAAAATCCATGACGGATAGCACAAAAAAAGAGCTTCGTACGATACCAAATACC
ATATATGCAGCTTTTAGTAACAGCAATGCAAGTGACCTAGTGATAACAAACCCCGGTAAT
TATATCGGTAATGTTACATCAGAAAAAGGACGTATTGAACTTAATGTGCAGGATTGTCCT
GTATCTCCTGATGGAAATAAACTTCATCCGAGGTTAAGTGCATCAATTGCTTCTATTGTC
GCAGATACGAAGGACAGTAATGGAAAATATCAAGCTGATTTAGTAGCCTTGCTGGAAAT
AGGAATAGTGGTGGGCAATTAGGATATTTATCTGGTACAGCCATTCAGGTTAATCAGTCT
GGATCAAAGTGGTATATCACCGCTACGATGGGGGTGTTTGACCCATTGACAAATACAACG
TATGTATATCTGAACCCAAAATTTCTTTCTGTAAACATTACAACATGGTGCAGTACAGAA
CCACAAACCTAA
>CS8_cofC AB049751 ~~~cofC~~~CS8 CofC unknown function~~~
ATGAGAACAAAATGGAGTATGATTCTTTTTTTGAGTGCCATTTCAGTCAATGTGATGGCA
TCTCCGGTTGTAAGCAATGATAAACCCAGAAGCCCATTCAGTATAGATCGTCAATCAGAG
TCAAATACTGTAAAAAATCGAGCCGATATTAATAATAGCATAGACAGTTATTCTCTTATT
CTAAAAGAAAACTCACTTCTCAGTCAGGAAGTTAAAAATTGGGCGCAGGAACAAGGCTAT
AAACTATTATGGCGAAGTGATAAAGATTATATTATCTATAAAACAGTAAGATTTAATGGT
CAGTCCCGTGAAGATATCCTAAAATCTTTAGGTGATCTATTTTCTTCAGAGCAATATGGG
CTGGTTGTAAAATTATATGCCGGAAATAATGTGCTGGTGATTGATTCTCAATAA
>CS8_cofD AB049751 ~~~cofD~~~CS8 CofD outer membrane usher~~~
ATGAAGTTAAAAGTTTCCATGATGTTAGTTTTGTTAGCATTACAGGGATGTTCATCATTT
GATGAGAAATTTTCAATTGAGAAAAAACTTTCACCAGAAGTTGGTCTTCTTGAAGCGGAA
GATGTAAAGGCATCACCATTTATTGTATATAAAACCTCTTTCTTGGGTGAAAAGGTTGCT
TACAGTGAGGAAAAACAACGCTTACTTGAGCAGAATATTTCTATTACCAGTTATGATGTT
ATGACGCTTGATGATGTGATGAAAACAATCGCATTACAGACGGGAATTTCATATAGGATC
AAAGAAACCTATCCTGGGCAGAATAAAGATGAGGTTGTTGAAGAGTTTCATAATGTAAAT
TTTCGTGGAACGGTAAGCGAATTTATTCGTTATATCTCTGCATTATATGATGTCAGTGTA
AAACTTGATGAGAATAACATTCTGAATATCGCTTATTATGATAATTATGCGATTAAACTT
GATTATTATGGTGAAAATAATAAGTTTGAAACCAGTATTGATCTTTCTGGTAACGAAGCA
ACCACTTCAGGAGGCGTTAAGGGTAAATCTGAACTAAAGTTCGAATCAAGCTTCTGGGAA
GATGTTGAAAAACTTGCAGAACAGTATGTAAGCAGTAAAAACTATAATATTATAAAAGAC
GTATCTGTTCTTGCATTTATTGGACGTCCGTCAGAATATCGTATGCTTGATAATGTACTT
AAAAAGTATCGGGAAGATAATAACCGTCAGTTTGTTTTAACATACAAAATTTTTACTATT
GACAAGAAAAAAGTTCGTAAACTTGGGGCTGCAGCAAATCTGAACTATCTTAATGGCGGA
ACCAGCATTGGCATTGATACCAGTTTGTTATCTTCTCTGAGCGGAGCTTCTTTTGGTCGC
GATTTTGTAGTTCCAGTTGAACAAATACAGAAATTTAACATTACTGGTAAAATTGAAGCA
ATTTATCAATTAACAGGGAAGAATGTTTTACAAAGTGGTACTTTTATAACCAGAAATAAT
GTGCCTGTACCTGTAAACCTGACACAGACACAAAACTATATTTCGGGAAGAACGCAGACA
ACTAACTCATTGACATCTGTATCCGATACTACGATTGAAACAGATAAGGTTATTACGGGC
TCAAGTTTTATACTGACACCACGGATCCTTTCTGATGGATCAATTGAGGTTACAAGTGCA
TTTACCAAGAAAACTCTGAATGCTATAGATAATTTTGAGTCAGTCCAGTTGCCAAATGTG
AGTACTACAGAAATGTTTAATACATCAGTTCTGAAACCGGGGACTTTGATGATGGTAGCT
AAATATGAAGGCGGCGAAGAAAATGATGATCGTGGTGCGATGATCTTCGGTGGTGAATAT
AATCGCGAAAATAGTGATAACACTATCGCGATGATTGTGGGTATTGATTATTACCGTGCT
CCGGTTATGGCACCATAA
>CS8_cofE AB049751 ~~~cofE~~~CS8 CofE anchoring function~~~
ATGAATCTAGAACAGATAACGGATGAAAAAATTACACTTGAGGTAATATGGCTTTCCTCT
GATAATGTGTCAAAAAAAATCACAGAAACACGGGAAAGAAGCTGGCGCAGGGTTATAGA
G
CGGCATTACAGGAAAGTTGAGTATGTAAATGCTGAGGGGAAAATATGTCTTGGATACAGT
TCAGACACATCATCAATGAACCAACCATTCGCTCTGTATATTCGGAATCTGTTTGGTGAT
GGAGTCTATTATATAAATGAAGAGGAGGGCAGAAATTATCTGTTAGTCATAATTAATGGT
GAGGTTATGCAAGGAACGGATGTATATATCAACAATGCTTTATTTGAACGATATCGGCAA
AAATTGCTGTCCGGGGAATATACATCATTGCAATGGAGTTGCCTGACAATGGCTCATATT
GATGAAGTTATTGAAGCGAACAATCTGTACAAAAGGAAAAATAAAAAGAAGAAAATTACA
TATCTGTTTACAATGCTAGGTATTGGAGTAATTTGTCTTGTGTTATTTGCCGTTGTGCTT
AAGATTTTCTTGGTGAACTGA

>CS8_cofF AB049751 ~~~cofF~~~CS8 CofF unknown function~~~
ATGAAATTATCTAATTTTGACTGGAAAACCTATCTTGCAAAAATACAGAGACAATCATAC
CTTGCTATTGGGGCTCTGATTTTTATCTTCTTCTGTGTTTGTGCTGGAGGGGTGTTGTAT
TATATTCAGCAAAAAGAAGAGGCTAAAGAGGCAGCAAAAAAACTCGCTGAACAGGAATT
G
GCTAATAATGTTAAAAGAATAAACGATTATTATACAAGTATTCTGAGTGGGGCTTCTCCA
CAAAAAACGATAGATATACTTATAGCTATCAGACAGAGTAGTGTACCTCTTTCACTCTCT
GGTTTTAATCTTGATACGTATAATTGCGATGTAACTAACTGCACCTTTAGTTATATAACA
GAGGAAAATAAAATATTTAATACTCAGGAAATTAACTTTTTTGGTAAAGACTATAAAGCT
AATGTGTCAGAGAAAGGTTTGGAATATCAGGTTGAACCATCACCGCTGAATGATGATAGT
TTCTTTAAAAAATATGATTTGCGTGAAAGTATATCTGTAGCGGATTGCAGTGAACTGGTA
AATTATGTGCAATCATTTAATTCACTTAAATCTGACAGCAAAGAAAAGATAGTACTTTCT
GGTTATCCAGATTCAAGTATCAGCGCTATAGAAAACATTCTACCTGAAGTTAAGAGTAAA
TATGGATTTAAAACTGTTCAGTGGAGCACTACATTATCTGATGATATTCTGAGAATCTCG
TCTTTTCTGAATCGTCAGGCATACAGTGAGTCTTTTCGTATAAATAAAATTGAAAAGAAA
AAATCTTCCGAAATAGAGGTTTCAGGAAAATTATTATGCGCAATTTAA
>CS8_cofG AB049751 ~~~cofG~~~CS8 CofG periplasmic protein~~~
ATGCGCAATTTAAATTTATATTCTGTTATTTGTATCGCGATATCAGGAGCTTCAGTTTGT
GGCAATGCCTTAGCTACAGATTTACAAGATAAGGTCGAAACAGTTAGCCCGGCACAGGAA
CAGATTGTGCGTATGCTTGAAGAGGATTTGAATTTCGAATCCCAACGTAAGCAACTGAGT
AATGAACTTGCTCTGGAAAAATTACGTATTGAATTAAATAAGCTCAAAGCAGAGAGTCAA
CCTGTCGTTACATTTTCGCCGTCACAATCTGATGAGAAAACAGGGGAATATTCACCCGTT
AAAACTGGCACGCCTCCGGTTATAGTGCTTGTTTCGGAAGTTGCTGGCTTATCGCGAATT
CTGATAAAGGATGGAGAATCTGTTAAACTTCGTAAACCATCTGAGGCATTTACAGCATCT
AATGGTAATAAATATAGGTTAGTTTCTCAAGGTGGTCAGAGATTCACTCTGAAGGAAGTT
CAATAG
>CS8_cofH AB049751 ~~~cofH~~~CS8 CofH unknown function~~~
ATGGCTGGGCTTGCTCTTGTTGATTCTGAGTTATATTATCAGTTACTTGAGGATATATCA
TTAGAAAATACTAAAAGACGTGATATTAATTCCAGCTCAATTCAGCAAGAGCTCGAAGCG
CTTTTATTAAATGCGGTAAATCGAGGGGCCAGCGATCTCCATATCACAAGGGGGGATGTA
ATGGCCAAGGTGGAGTTAAGAATTAATGGGGTACTTGTTCTTGATCACCAGATGCTTTCA
TCAAAATGCGATGAATTAATCTTTGTTTTATATAATGTTCAGGCTTCGACGCGTGATACC
ACCTGGAACAGAGCTATAGCGCAGAGCGCAAACATTTTATATACATTAAACGAGCGAAGC
TATCGATTTAGATATGAACATTACCCCATTTTTGGGGAGTCGGATGGATGTTATCATTGT
GTTTTACGTATAATTCCCTCAGGTTTATCCACAGTTGTAAATCCAGATCTGGCAAAACTG
GGAGTATCTGATGAGGAGGTAAAAGACATACAAAGGATTTTAAGTAATCCTTATGGTGTT
TATTTTATCGCGGGTACTACGGGCTCAGGAAAATCTACCACTTTAAAAAATATGATGGAG
TGGATGCAGATAAATCGCTATGATGATAAGGGATGTTTTTTAACCGTTGAAGACCCGGTT
GAATATCAGATTTATGGTGCCAAGCAGAGCTCAGTTCTGGATGGAGAAAACGGTGGTTTC
CATTCAGCAATAAAATCATCTTTACGACGTGATCCAGATGTTCTAATGGTTGGTGAAATA
CGTGATCCGGTTTCATCAAATGCCCTTGCCGGAGCTGTAGAAAGTGGTCACTATTGTTTT
ACAACCGTGCATGCTGGAAATATTGTTACGTTACTACAGCGACTTTCAGCGCTAGGTATT
AGTAGTGATAAGTTATCAACACCTGGTTTTATTGCAGGTTTACAGTGTCAGAAATTAATT
CCAGTATTATGTGATAATTGTAAACTTGAGTATGCGGTAAATATTCTGGGGCGCCGGTTT
ACTTTATGCAAAGTAAATGAAAGTGGTTGTGAAAGATGTAAGAATACTGGTGTAAAATCT
CGTCAACTTGTAATGGAGTATTTAATTCCGACTCAACGTGAGTTGGAAGCACTGGCTAAT
CAACACTGGCTCAATGTATATTCGATATGGCGAGAAAAACGGTTTACGTCGTCGGGAATA
TCTGAGGGGTTTTCCATTAAAGAAAAGGTATTTATGCATGTGTTACGCAAGCGTGCGTGT
TATCAGTGGTTCATAATGGAGTTTGGTGAAGTTCCGGATACTGATATGGAGGTGTTACAT
GAAAAAATTCAATAA
>CS8_cofI AB049751 ~~~cofI~~~CS8 CofI unknown function~~~
ATGAAAAAATTCAATAAAAAACAACGAATTTATCTCTATCAGTTTTGTGCCGATATGCTG
CATTCAGGGTTACCGATCTATGATTCTATTGTGAAATTACGCTCTGAAGGTGAAGCATTA
CTGGGTAAGTCGTTTGCAAAACGGCTTGATTATTTAACGGGGCGAATGAAAAATAGTCCA
TCTGTTTCAGCTTCGTTTGAAACACTTATTCCGACAGAGGAATTAAGCGCTATTACCGCT
GCAGAAAATAGTGGTAGTCTTGCGGAAGGTTTTAACAGTATGGTTCTGACTATAAATTAT
CAGGAGAAATTAAAAAGTCAGTTGATTAAATCTGTAACATTTCCGGCTATAATGATGGTC

TTGGCTCTTATTGTTATAGCGGGCTACGCCGTAAAAGTATTCCCCGCTTTTGAAAAAGTT
GTTGCGGTTTCAAGATGGCCTGGGGTAACTCAGAGCCTTTATCATTTTGGCACGGCTTTA
TATAATGGATTATGGATCACCATATTGGTTTCTTTTGTATCAGCAGTTTTTTTTATTCGC
TTTATTATGTTTAATTTCCATGGAGAGCTTAGGGATAAGTTCTTGGACAAAATCGTTCCT
TTTTCCATTTATAAGAAATTAGTAGCTTCAGTGCTCATTAATGATTTGTCTTTAATGATA
AAGAATAGAATACCATTGTCTAACTGCTTGATCATTATAGAAAAAAATGCCAACAAATGG
CTTAAGTCCCATATCAGAAAAATGCAGGATAATATGGCAAAAGGTTTGGGATATGGTGAT
GCATTTAAGACAGGATTACTTGGCGGTGACGAGTTACTTAATATCAGTCTTTATGCGAGC
TTGCCTTCTTTCGATAAAGTTTTAGGCACAGTTTCTGATAAAGCGAAAAGTAAAATTGAG
CAAAACATAAATGCGCTGGCAGGTATGTTGAAATCACTGTCGACTTTAGTCTTGGGTGGA
TGCGTCGTGTGGGTTTTTATTGCGCTATTTGCGCTTTCTGATGAACTGTCAAAAATGACA
GGTTAA
>CS8_cofJ AB049751 ~~~cofJ~~~CS8 CofJ unknown function~~~
ATGAAAACAAAACTCGGGTATAGCATTTTAGCTGCAGCAATATTTTATTCATTGCCGGGG
ATGGCATCGCCATCCTCTTCAGAAGGTGGTGCTTTTACAGTCAATATGCCTAAGACCTCT
ACTGTAGATGATATTAGAGGTTGCCCAACTTTGGAAACACCATTAAAATTGACCTTTACA
GAAGATATTCAACCTCGTAAAGAAAATGGTTCAACATATTTTTACTATGATGGCTGGCGG
GGAGTTGGACAAACAGTTAATCCATGGTCTCCTGTGTTGGATAACCATAAATATGCAGCT
ACAGAACATGAGATTCACATTTATGTAGAGTTTTTCCAAACACCGTCGAATCGTTTTGCT
GATAAAAATGGTGCTTATTCATATATCGATGCTAATGGAGTTATGTATACAAATGGTGAA
TATTCATGGGAGCATGTTCCAGCACTGGGGAAAAATATATACAAAGTTGTTATTAGTGAT
TGGAATAAGGGGCAAACGAAAAGTATCTACTTACCTGGCAGAGATTTTAAAACAGTTGAA
GTTTTTCATTTTCAAAACAATAGACCTCAGTGGGATGACCGAAATAGCTATGAAAATGTT
AAATCACGGATTAACAATAACATCAGCAAAAGTTACTCTAAGGCTAAACTGAATGAACAG
TTAAGTACTTACGTACATGATGATGGTACTGACTCTCTTTTCTTATATCAAAAATTGAGT
CGAGCCAGTTTGAAAGAAAGCCAGATTAATTATTATCAACTACGAGGTAAATTTAATGGC
GTAAATCTTGGTTACTGGGCTCAAGAATATATATTATTTGGAGGTGAAGGTGCAGAGCAA
TTAAAAAATAAAATACCTGACATGAGCAATTATTCCATGGAGGATAACGGTAGTTTTAAA
AATGCATTAAAGATTGAAAGTCTTGACCTTAGACTAATGGATAATAATCGAATGGCGTAT
GGTTCTACTGGTACATATATTGCTAGTTTTAACAGAACCGATTTTTCTATGACACCTGAA
AATTTGAAGGCTTGTGGCCTTGATTAA
>CS8_cofJ AB049751 ~~~cofJ~~~CS8 CofJ unknown function~~~
ATGTATATTGAAATCGGCATTTTTTTTGCGCTCTTAATTGTTGGGTTGTGTATTGGAAGT
TTTTTGAATGTGGTCATTTACCGTATTCCAATTACTCTTCTCAATGCTAATGAAAAAGTG
TTTAACATTGCATGGCCACCTTCGCATTGTACACACTGTGGGAATAATATTCTCAAAAGA
GATAATATTCCAGTAATTAGCTGGTTTATGTTAAGAGGTCGGTGCCGATTTTGTGGTAGC
GTGATTTCCTCACGCTACCCGTTTGTAGAGTTTATTACAGGACTCATTTTTTCCGTTTTA
GGTACATATATGTTTTTTATCGCAGATAAAAACATATATGTTGTATCATTAATTCTTTTT
CTTTTTTCAATACTTCTTTGTCTTGCTGTTATTGATTTTGACCATTTGCTATTGCCAGAC
AGTCTTGTTTATATCATGCTTTGGGGGGGGCTCTTGGCTGCGGCATTTGAGTTATCACCC
ATAACCTTAAAAGATGCTGTCATTAGTATATGTGGAACATGGATAACTCTCAGTACGATT
GTTCAGTTATTTATTTTTATCCGTAAAAAGGAGGGATTGGGAGCAGGTGATATTAAGCTT
ATATGCGCTTTGGCTGCTTGGATAGGCTGGTATAATATTCCTTTTTTACTTGTAATATCT
TCTATCATTGGTGGTGTTATGTTTATCAATATTAAACGAAGATTTGATTGTGGCTACGAT
GGGAATATAAAAAACTGCCACGTGATACCTTTTGGCCCGGCAATTTCCATTTCTGGGTTT
GTTATATATTTATTCATCGCTAATGGGTACGATGTTCTCTAG
>CS8_cofP AB049751 ~~~cofP~~~CS8 CofP prepilin peptidase~~~
ATGTATATTGAAATCGGCATTTTTTTTGCGCTCTTAATTGTTGGGTTGTGTATTGGAAGT
TTTTTGAATGTGGTCATTTACCGTATTCCAATTACTCTTCTCAATGCTAATGAAAAAGTG
TTTAACATTGCATGGCCACCTTCGCATTGTACACACTGTGGGAATAATATTCTCAAAAGA
GATAATATTCCAGTAATTAGCTGGTTTATGTTAAGAGGTCGGTGCCGATTTTGTGGTAGC
GTGATTTCCTCACGCTACCCGTTTGTAGAGTTTATTACAGGACTCATTTTTTCCGTTTTA
GGTACATATATGTTTTTTATCGCAGATAAAAACATATATGTTGTATCATTAATTCTTTTT
CTTTTTTCAATACTTCTTTGTCTTGCTGTTATTGATTTTGACCATTTGCTATTGCCAGAC
AGTCTTGTTTATATCATGCTTTGGGGGGGGCTCTTGGCTGCGGCATTTGAGTTATCACCC
ATAACCTTAAAAGATGCTGTCATTAGTATATGTGGAACATGGATAACTCTCAGTACGATT
GTTCAGTTATTTATTTTTATCCGTAAAAAGGAGGGATTGGGAGCAGGTGATATTAAGCTT

ATATGCGCTTTGGCTGCTTGGATAGGCTGGTATAATATTCCTTTTTTACTTGTAATATCT
TCTATCATTGGTGGTGTTATGTTTATCAATATTAAACGAAGATTTGATTGTGGCTACGAT
GGGAATATAAAAAACTGCCACGTGATACCTTTTGGCCCGGCAATTTCCATTTCTGGGTTT
GTTATATATTTATTCATCGCTAATGGGTACGATGTTCTCTAG
>CS12_cswA AY009096.1 ~~~cswA~~~CS12 CswA major subunit~~~
ATGAGAAAAATTACGTCTCTGATCATGGCTGTTACTCTAATGAATAGCTCAGCCTTCGCT
GCGATCGGTAGTAACGGCCAAGCGAATAACAATGATGCAAGCCAGGCTGAATTGCATTTC
ACTGGTAAGTTGACTTCCAGTCTATGCCAGGTTGCTACATCAGATGTTAAAAAAGAAATT
GATCTAGGGGAACTTAGCAAAGCTGCGTTAATTGCATCAGGTCGAGGGCCATCGCAGTCT
TTTTCAGTTAGTTTGGTAAACTGTGACCCCACAATAAATACAATTAGTTATGCTTTGCAG
GATAAAAACGGCAGTGTAGGTAATTATCTTGTAAACCAGTCAGGTGACACGATGGCTAAG
GGGGTTGGTGTCTATATTGAAAATAATTTAAATAGCCCTTTGAAGGTTGATGGTTCACTT
AATACTGTTGGTGTTCAGAAAGATGGTGCTACGGCTTTACCTGATCAGGTTATTCCGTTG
ACTGCTTATATTGGTAGCACAACTCCGGGAGCTGTGGCCGATTTTGCTACAGTAACTCCT
GGCTTAGTTGATGCAAATGCAGTAATGACTATTCGTGCGAGCGCACCATAA
>CS12_cswB AY009096.1 ~~~cswB~~~CS12 CswB first chaperone~~~
ATGGGAGGAGAGTCTCCTCCCATTCCGGGAGAGGTTATGTGTTATCAGTATTCTATTTTA
TTTGCTTTTTTATTCTCTGGCATTGTGCAGGCTGCTGAGGCTTTCGGTCCACGGGAAAAT
CGGTTGTTTTTTGATGAGAAGAAGGGAAACACCTTTTATCGGATTGACAATTCAGACAAA
AAACTTCCCTGGCTTGTACAGGCATGGATTGAAGATGCCTCTGAAAATAAAACAACTGCA
CTGACATCCATACCAATGGTGTTTCGCGTAGAGCCTTCTTCCGTTTTTACCGTTAGGGTG
GTTAAAACAGGAGTCTTGCCGGAAGATAGGGAAACTCTTTTTTGGGCTGTTTCCAATTCA
TTACCAGGAGTCGTTCCCACAAAACTGGATAATAAAGAGGGGAAAATCACTGCAAAACTC
AGCCTAGCTTATCGCTTTAAGGTGCCGCTTATTTACCGGCCTACAGCGTTAAATAATTTC
AGACAGGAACCGGAGAAACTGGAGTGGACATATAATGGTAATGAAGGATTAAAATTATAT
AACCCTACCCGTTACATAGTTCAGTTATATAACATAACGGTAAATGGTCATGAATTTAAG
GGAGAAGGGGTTTCCTCTATTATTACTCCTATGTCAGGTAAAAATGTAAGTGCCACTGTG
AAAAAAGGTACAAGAATAAAATATAGTGTAATAAATGACAATGGTGCGATAAAAGAATAT
GAGGGTATTGTTAATTAA
>CS12_cswC AY009096.1 ~~~cswC~~~CS12 CswC second chaperone~~~
ATGAGGTTAACAATTAAACAAGGGACGGTGATATATTTTACGGTTATTAACGATACAGAA
CGAGAGTATATTGTTATACCAAGAGTAGTAAGTGATTCAGAGATTGATGATAAAGATAAT
ATATTTGTTTTTAGCCCACCTCTGAAGATTTTGAAAAAAAGGGAGACGTCTGTAATGGGA
ATTGTATATCTAAAAAGAATAGGGCAGGATAAAATGAAATGCTATTTATCTGTCTCATTT
ATTCCAAAAGTGACTAAGGATAATATGAGAATATCTATTCCAGTAGTTCTTGTTCATCAA
ATACCGCTGACATTTGAATAA
>CS12_cswD AY009096.1 ~~~cswD~~~CS12 CswD outer membrane usher~~~
ATGCATAAATATCAAATACTAATGATTTCTTTTTTTGTGTTGTGCGCAGAAACTTTTGCC
GAGGATTATTTTAATCCATCTCTTCTTGCGACAGACATTATTGGTGAAGGTAATATTGAT
CTTTCTGCATTTTCACGTCACGGCGGGGGTATGGAAGGGGAACGAGAAGTTGCTATCTAT
GTTAATAATAATTTTTATTCCCGTAGAACTCTTGTTTTTAAAAATACTTCAGATAAAGGA
CTATTACCTGAATTTACTCCAGGATTTTTTGATAACTTGGTGTCGGGGGATATTTTGGTG
TCCGAAAAAAAATAAAATAATATCATCATCAGACTTCCTGAAAAAGGTCCCCTATAGTGAT
ATAAACTTTGATCAAGGAATGTCTCGCGTCAATGTTAGTATTCCTCAGGCATATCTTGGT
GATGGGGCAAAGCTGATCTCCTCGCCTGATACATGGGAATATGGCGTACCAGCATTTTTG
CTGGATTATAATATTTCCGGAAACCGGAATGCTTCAGGAAATAATGATTCACGTAGTTTG
TATATATCCTCCCAGATGGGAGTAAATCTCATGAAGTGGCACCTGCGTACATCCTCAAGT
TATAGCAATTATAAGACAAATTCTGTATGGGGAGGTGCTCGTTCTGAGCGAAACAGCTTT
TATAATACTTACGCAGAGAGGGATATCAGTTCGTTACGTGCAGTTTTACGACTTGGTGAA
ATTTCGACCAGAGGATTGATACTGGATTCTGTCCCTTTTCGAGGTGTAAAGCTGTCAAGT
AGTGATGACATGCTGGATGCGCGTTTGCGTAATTACACACCCACAGTGCGAGGTATGGCG
CGTAGTCAGGCTGTTGTTACAATAACTCAGAATGGTCGGCAGGTATACCAGACAAATGTA
CCAGCAGGACCGTTTGAATTGAATGATTTTTATCTTTCCGGTTATTCGGGGGACATGCAG
GTAACTGTTCGTGAAGCTGATGGTAGTGAGCACAGTTTCTTGCAACCTTATTCGACCCTT
CCGGAAATGAAGCGAGAAGGAATTTCCGGGTTTGAGATTTCGGTTGGTCGCTATGATAAC
AACGGTGCTGAACACTATTACGATGCTGAGTCATTTGTTTATGGTAACTGGTCTCGTGGT
TTTGCTCGTGGAGTGACATTTTTTGGTGAAACTCTTCAGGCAGAAAAATACCAGAGTCTG

GGGGTAGGGAGTACGTTGTCGCTGGGGAGATTAGGGGCTTCATCTGCAGATATATCCTTA
TCACGTGTGGATAAATACGGTGCCATCCGGGGCGGTCAGTCTTATGGTTTAAATATTCC
AAGAGCCAGATTGAAACGGGCACAACATTAACACTGGCAACATACCGATATTCCACTGAG
CATTTTTATACTTTCCGTGATTTTGTTTCAAAAACGGATACTGCTCGTTATGTCTGGGAA
AATAAACTGAAAAACAGAATGACATTGAGTCTCAGTCAGTCTCTGGGAGAATATGGTTAT
CTGTCAGCCAGTGCCAGTAAGCAGGATTACTGGACCAGCAGAGAAGTGAGTCGAAATTAC
GCGTTGACTCATAGTTTTAACTGGAATGATATTTATTTAAGTACAACATTGTCAATGGAT
GATCAACGAGGACGGGAGGCTGGAGATTTGTCCAATAAACAAGCAGGACTATATGTCACT
GTTCCTTTAAATAAGTTTTTACTAGGAATTGATTCGACAGGTAGTTCACTAACTTGGAGT
ACTTCACATGCTGATCATAACGTACGCAATAGTGTGACACTGAACGGGAAAATGCCAGAA
AGTGATGTACGTTATCGAGTGGGAGGTAGTTGGGGTAATGGTAACACTGAAGGCTCGCGT
ACGGTATCAGTGAGTTGGAGCGGAGATTATGTCAGCACTTCTCTGGGATATGCGCGTACA
GGGAAATATCGAACTCTGGATTACAATATGTCTGGCGCAGCGGTGATGTATCCATGGGGA
ATTGCTATGGGTAACAATAGTGTTACTGGTGATGGCGCTATTGTAGTAGAAACACCTGGA
GCTAAAGGGGTAAGAACAAGCACTGGATACAGAACTTCATGGTTGGGAACGTCTTTAGTT
AGTTCTCCTCGAAAATATACAGAAAATCGTATTGATTTATATCCTGATGGCCTTCCTGAT
GATACAGTCCTTGGTGAAACATCTAGAACCACAGTGCCAGTTAAAGGTGCAGTCGTTGTA
CTGGATTATACAGTTTTTCGTGGTAAGCAGGTAGTGTTCACACTGAGACAGAGTAATGGT
AAGCCATTACCGTTTGGTACAGTAGTTACTCTTGATGGTGTATCTAGAGGAAAAGAAAAT
AGTGGTATTGTGGGTGAGGAGGGCCGAGTGTATATGGCGGGTATGCCGGAAAAAGGAACT
CTCACTGCAACATGGGGGCAGGATAAAAGCTGTAGTGTACCATTTCGTATAAACCAGCAT
AAAACGGAGGCCGTTATCAGTGAGATCCATGGGGTATGCCGTGTATGA
>CS12_cswE AY009096.1 ~~~cswE~~~CS12 CswE third chaperone~~~
ATGTTACGAATCTTCATTACAATTTTATTGCTCCTAACTTCGTTAGTTACGAAAGCTGAA
ATATTGTATCCTTGGCCTAGCGATACTGAAGTTAGACTGAAAATAAGTGGCGAAAAAGGC
CAGCGAAGAGGAACTCTACGAATTACTAATCCTGGTGATGTTCCTTGGCTGGTTCAGACA
TGGGCTGAAGATGAACATTATAGAAGATATAACGTTGTATATCCATCCGTATACAGATTG
GAGCCATTCGGCGCTTATGCTCTGAATGTTTACCCAGACAGTAATGTGATCTCAAAAAAA
CTGAAATGGTTTTTGGTTACTTTCATACCTTCGACAGTGAAGGAAAATAAAAATCAACTA
TTTATGCCTGTGACTTATAGACTGAAAATAATTGATGAAGGGTAG
>CS12_cswF AY009096.1~~~cswF~~~CS12 CswF minor subunit~~~
ATGTTGAAAAGAATATCGTGTATAATTTTTGTTTTTTTTTCAGGGCTGATTTATGCTGCG
GAAATTACAAATCAGATAGAGCTTTCGGTAAAGGTTAATATATCTAAGCCTATGTGTAAA
CTTAATTCTGGAACGCAAACAATAGACTTCGGCGATTTTGATGTACTGGATATTATTACG
GAGAACAGAAAATTAAATGGTCATGCGACCTTTAAATTTACTGAGTGTAGTTCTGTCAAA
AACATGAAGATAAAATTTAAACAGGCAGGACAAAATCCAGCGTTAGATATCGTAAACAAT
TATATCCCTAATAGTAAGGGAGATAGAATGGCAAAGGGGGTAGCGGTAAAGCTTCTGGAT
GATAAAAAGCAAGAAATTCAACTGAACAAGGAAATGAATGTTATTGTGGAGGAGAGTCTG
ACATTTAAAGATTTAACGTTAAATGCTCAGGTTATCTCTATTAATAAAGACGGAGAGGGA
GTTTCACCTGGGCTACTTCAGACCGCAATAGGAATGGAGATATCCTATGAATGA
>CS12_cswG AY009096.1 ~~~cswG~~~CS12 CswG adhesin~~~
ATGAATTATATTAATGAATATCCAATGTGGTTTTCTACCAAATTGAATGTAAAAACAATG
CTTCAAAATTTTTTTATTTTTGCTTCTTTATTTTCTATACAAAAATGTGCAGAAGCGGTT
GTGATTAGCCGAAACCATACAATTAACATCGATAAAACAATTGATGTAACAACAGTAACT
AACGGTTGGTTGGAGTTGGGAAGGTTTACCTTACCAAAGGCTTCGGGTGATAATGAATCA
CATTATCGTTGTAGTACGGGAGCGGGATCTGGTTTCTGCGCTTATTCAGAGTTTCGAGTC
AATGGAGGATTACAAAGTAATCCGGCAGCATACTGGACTGGACGACTTCGAATGGAGCCT
GTGGATGTAGATACAGAAAATGGGAAAAAGCTAACATTTTCGGCATATTTTAAGGGGACT
CCCATTGTTCGATGGGAAGAACATAACCATCAGGGGGGGAGAAGTATGTATCATAAATAC
TTCATTGGAGTTGGATCTGTTGGGACATCTGCAGGAGCTATAACCCAGTGGAGTTCGGGA
GAAAAATCTCCATCCTTAGTATGTGGTTCTATTGCTGGATGTACTATAGGTACGCATACA
TATTTTGATAATACTATGTCTGGTTCATTAGTATTATCGGTTAAATTACCGGCAGATTTC
CAAAAAGGCACTTATATTTTTTCTAATGTTCCTGTTCTTCATTTAGGTCATACATCTCGT
AATGCTTCTGGTACTAATAAAGAAAGTATCGATACGGTAGTTTATATTAGCGGGAAAATT
ACAGTACCTGAAAGATGTTATATTGAGACAGGCTCTAATGCTGAAATAAAATTTAATGAT
GTAAACGCAGGTATGAATAATGGAAAACTTGAAGAACGTAATTTTGAATTAAGGACTACA
TGTAAATATATTAATCTTAAACTTAAGCAATATGTTAAAGTAAGTGGGAGTAATGGTGAT

TCAGAATATGAGATTTTTTCTAAAACTAGCTCAAATGACAAAGCATTGGCTCTAGTAATG
AATATTGTTCGTGGAGATAATGGGGAAAATTATTCAGCAGATTGCAATCCTTCTTCTGGA
CGGGTTAAGTTTGGTCATGAGTATCTCTTGAGAGAAATAAATGGTAATGGATTAAATATT
TATAGTTATAACGATATAATAAAGCTTTCATTATGTAAATATGGTGTGCCAAAAGATTAT
GGTGAGAAAAATATACCTCTTACTATTATTTCAAGATGGAGCGATTTTTGA
>CS13_cshA X71971 ~~~cshA~~~CS13 CshA ~~~
ATGCGTCAGAATGGTCAGAATGCAAAGATAAAAAAGTTTGCTTTTATGTTTGTAGTAATG
ACATTTAATTCTTTTGCATATGCAGGAATGACCCAACGAACGATTTTTGATGTGATGGTT
ATCGCTTCCACTTGTCATGTGGTTGTTGATACAGAAAATTCTGTGGGCAACAGCGGACGC
CTTATTTTTGGTACTTATCGTAAATCGATGCCTAGTTCTGTCCCATCGCAAGATTTTACA
GTGCGTTTGTTTGAGTCCGGATCTACTGTTCAGGGCTGTTCCGCTTTTCGGGCCGGACAG
ATCGCCACCCTGGATTTTGGTAATCCGGGACAGCTGGATGCAGGGGGCGTGGTCACCCGC
GGTGTCGGTGATGGTATTCGCGTGGATGTGCGCGCGGTGGATGTACAGGCAGATTATCGC
GGACGTCTGACACAGAGCAATCATTCGGTGAATTACCCGGTGGAGTTTGCTACCAAGGGG
CAGTTCCGCTTTCGTGCACAACCGATGTTTCCGGCGAACGTGAAAGCGGGGGAATACACG
GGTGCGCTGACCTTTGTGGTCTCGTATCAGTAA
>CS13_cshB X71971 ~~~cshB~~~CS13 CshB outer membrane usher~~~
GTGCTCTTCTGTCGGACTGCACTTGCGTTTGCTGTGTCAGCCGCTTTCGTCAGTGCCACA
GGTGTTGCGAGTGAAAAACTGGATATGTCCTTTATCCAGGGGGGGGGCGGAGTTAATCCG
GATGTCTGGGCAGCCCTGAACGGCAGCTATGCGCCGGGGCGTTATCTGGTTGACCTGTCC
CTGAACGGGAAGGAGGTCGGGAAACAGATACTGGATGTGACACCACGGGAGAGTAATGA
A
CTGTGTCTGACAGAAGCATGGCTGACGAAGGCAGGAATTTACGTCAGTGCAGATTATTTT
CGTGAGGGATATGACGCCACACGACAGTGTTATGTGCTGACAAAAGCGCCGTCAGTGAAG
GTGGATTTTGATGTTTCCACCCAGAGTCTGGCACTGTCTGTTCCCCAGAAGGGGCTGGTG
AAGATGCCGGAGAACGTGGATTGGGATTACGGGACCGGTGCATTTCGCGTGAACTATAAT
GCGAACGCCAACACCGGTCGCAATAACACCTCAGCCTTTGGCTCAGCAGACCTGAAAGC
C
AATATCGGACGCTGGGTGGTGAGTTCTTCTGCCACAGCCAGCGGTGGCGACAGTGGGGGT
AACACTGCCACGATAAATATGTTCACGGCCACCCGGGCCATCCGCGCACTGAGTGCGGAC
CTGGCGGCCGGGAAAACATCCACCGGGGATAGTCTGCTTGGCAGCACAGGAACGTATGGA
GTGTCGCTGAGCCGGAACAACAGCATGAAGCCGGGCAATCTGGGGTATACCCCTGTGTTC
AGCGGCATTGCGAACGGGCCGTCGAGGGTGACGCTGACACAGAACGGGCGGTTGCTGTA
T
TCGGAGATGGTGCCGACAGGTCCGTTCTCCATCACCGATGTGCCGTTGTATACCAGTGGT
GATGTGACGATGAAAATCACTGGTGAAGACGGACGAGAGCAGACCCAAGTCTTCCCGTTA
TCGGTAATGGCCGGGCAGTTAAGCCCGGGGCAACACGAGTTCAGCGTGGCAGCCGGTTTG
CCTGACGATGACAGTGACCTGAAAGGCAGCGTGCTGGCGGCGTCATATGGTTACGGTCTG
GACGGGCTGACGCTGCGCACCGGGGGAGTTTTTAACCAGGACTGGCAGGGTGTCAGCGC
C
GGAGCTGTTCTGGGGCTGAGTTACCTGGGGGCAGTATCTGCTGACGGGGCTTATGCCAAG
GCAAAATACCGTGACGGCAGCCGCAGCGGAAATAAAGTGCAGTTATCCTGGAGTAAACAA
CTGGAGATGACGAACACCGGACTGCGGGTGAGCTGGTCACGGCAGAGTGAGGAATATGA
G
GACATGTCCTCCTTTGACCCGACAGAGCTGTGGTCACAGTCAAATCATGGTCGCCGGACG
AAGGATGAGTGGAATGCTGGTATCAGTCAGCCGGTGGGTGGATTGTTCAGTCTGTCGGTG
TCCGGCTGGCAGCGGAGCTATTACCCCGCATCCATGACCGGGAGTTACCGGTACAGCGAT
GACAACGGTAAAGACACAGGTATTACCGGCACCATGAGCACACAGATTAAGGGCGTCAGT
CTGAATCTGGGCTGGTCCGGTTCACGGAACACTCGGGGGGGAAACAACTGGTCTGCATCG
GCGTCAGTATCGGTACCGTTCACACTGTTTGACCGCCGTTACAGCAGCAGTACGTCGGTG
AGCACCAGCAAAGACGGTGGTACTGGTTTCAGCACTGGCGTATCCGGCTCGCTGAATGAC
CGTTTCAGCTATGGTCTGGGCGGTGGGCGTGACAGCAGCGGTGGTGTGAGCAGTTATCTG
AATGTGTCGTACAGTGGTGACCGGGCTTATCTGAGTGGAGCTCTGAACAACTCGCAGTCC
GGCGGAACCAGCGGTTCTGTATCGGCCAGCGGTTCGGTACTGGCCGTTCCGGCGGCGAAA
GACATTATGTTCAGCCGCACGACCGGGGACACCGTGGCGGTTGTGAACGTGAAGGATACA
CCCGGAGTGAAGGTGACGTCCGGTGACGGACAGACTGACAGCGACGGCAACCTGGTGGT
A

CCGCTGAACAGCTATGACTGGAACACGGTGACGATTGATGCGGGTACACTCCCGCTGAGC
ACCGAACTGACGAATACCAGTCAGAAGGTGGTGCCGACGGACAAAGCGGTGGTCTGGAT
G
CCGTTTGACTCCCTGAAAGTTAAGCGTTACCTGCTGCAGGTGAAGCAGCGTGACGGTGAG
TTTGTGCCAGGGGGAACGTGGGCACGTGACAGTAAGAACACACCGCTGGGCTTTGTGGC
C
AACAACGGTGTGCTGATGATTAACACGGTGGATACACCGGGTGATATTACCTTGGAGCAG
TGCCGGATACCTGCGGCCAGACTGCAGGATACTGAGAAATTACAGGAGATTACGTGTGAG
TAA
>CS13_cshC X71971 ~~~cshC~~~CS13 CshC chaperone~~~
GTGAGTAAGCGTAACGCAGTTACGACGTTTTTCACTAACCGGGTGACAAAAGCACTGGGA
ATAACTCTGGCGCTGATGATGACCTGTCAGAGTGCGGTGGCTTCCCTGGCGGCAGACCAG
ACCCGCTATATCTTTCGCGGAGATAAGGATGCGCTGACCATCACGGTCACCAACAATGAC
AAAGAGCGTACCTTTGGTGGACAGGCCTGGGTGGACAATATTGTGGAGAAGGACACCCG
C
CCGACTTTTGTGGTGACGCCGTCCTTCTTCAAGGTGAAACCGAATGGTCAGCAGACAATG
CGTATCATCATGGCCTCGGACCATCTGCCGGAGGATAAAGAGTCGGTGTACTGGCTGAAC
CTGCAGGATATTCCGCCGGCTCTGGAGGGCAGCGGTATTGCCGTGGCGCTGCGCACGAAA
CTGAAGCTGTTCTACCGCCCGAAGGCGCTGCTTGAAGGCCGCAAGGGGGCAGAAGAAGG
T
CTCAGCCTGCAGAGTCGCCCGGATGGCAGAATCATGCTGGTGAACACCACGCCATACATT
TTTGCGATTGGCAGTCTGCTGGACGGAAACGGAAAGAAAATTGCCACGGATAACGAGAC
G
GCACAGAAACTGCTGATGTTTATGCCGGGTGATGAAGTACAGGTAAAAGGAAATGTGGTG
AAAGTGGACTCCCTGAATGATTACGGTGAACTGCAGACCTGGACAATTAATAAGAAGAAA
CCGACAACACCGGAAGCAGCAAAAACTGAGAAAGCAGACAGCACAGAGCAGAAATAA
>CS13_cshD X71971 ~~~cshD~~~CS13 CshD ~~~
ATGAAGAAAACAATGATGGCCGCCGTCCTGGCTCTGAGTGCACTCAGTATTCAGTTTGCA
CCTGCCGCTGAATACAGCGAAAAAACTCAGTATCTGGGCGTGGTGAATGGTCAGGTGGTG
GGTAACAGCGTGGTGAAAGTAAGCCGTACACCGACAGACCCGGTGCTGTACCGGAGTGG
T
GACACGACGCCTCTTCCCGGCAGTCTGACTATCCGCAACGCAGAGTCCAGGGCGGCATCC
GGTGGCCTGGCGTATATCACGGTGAAGCAGGTGCTGCCGGATAATGGAGAAGCCCGCATC
ACCCTGAAGACAGCGTTGATGGTTGACGGAAAGAAAATGGCTCTCAGTGCCAGACAGCA
G
GGCGAGGATGTGGTGATAACCGTACCGGAGGCACAGAAACAGGTTGAGTTAAGAACGGA
T
ACACCGGTTGAGCTGGAAGTTCCGGTCAACTACCGGGGAAACTTGCAAATATATTTGCAA
GTGGAGGACTGA
>CS13_cshE X71971 ~~~cshE~~~CS13 CshE major subunit~~~
ATGAAAAAAACTCTGATTGCACTTGCTGTGGCTGCTTCTGCAGCAGTATCCGGCTCTGCT
ATGGCGTGGTCTCAGCAGGATAGTGCTTTTAATGGTAACATTGAGCTGGGAGGAACACTT
TCTCCTGAAGTAAAGAAACTACCATGGGAGCTTCAAATTGGTACTGGTAGTAGCCAATTA
AATGGGACTATTGCCAAGGGTAATAGAGAGGCCACCCTCACGATAAATGATGCTATTCCT
GTTTTAGGTATGCGTAACGTGAATGGTGGTTTTAAAGGAGAGGCTGGCCTTACTCCTCAG
GTTAGTTACAACGGAAAGGTTGATGTTGATACTTTCAATGCCGGGACTGCCACAATGAAT
TTGGACGTCACTAATAAGTCCGGTGCTCGTATTGGCTCTTTAAGTGTTGACTTTTCAGCA
GCTGCTTATGGTGCTAATAATACTAATCGAGCGTCTCTGTATGCAGGTTCGGCCGGATAT
GCTTTCTGGGGGGGGGTCGCTAAATCAGCAGGTGGTGCTGTAAATAGTGTTTCTGATGTC
GAGAATTTGGCTGTATCGTTCTTCCCGAATATTCTCGATACTAAAGGTGATATGAGTGGT
TTAAGAAATTTAAATCCTCACCAGTTTAAGTTTGATTCGGCAGAGAGTACATATCGTGCT
ATTTATTCTTCAGGTATTAGTGCTGGGAAAAATATCTCGATTTCTCTTGATGATCCGGCG
ACTACTGATAGAATTGAATGGAGAGCTTCTCTGCCAGTTACAGTTTCTTACCAGTAA
>CS13_cshF X71971 ~~~cshF~~~CS13 CshF~~~
ATGAAAATAACAAATAATTATAATTTCCTTCTCTTGGCTATTATTTTGATGGCCCTGTTT
TATCCGGCAGAGTCATATGCAAAAATAAAACTCGATGGTGGCGAAATTCAATTTAATGGT
TTTGTCACTGACGATGCTCCCAAATGGACGTGGCAGATAAGTTCACCGAACCAGACTTGG

GCTGTGGATACTGCTGATGCGCGCACAGAAAACGGACAGCTTATTTTTGATTTACGAAGG
AAAGGAGCACTCCCGTTTCTGGAAGGTCATTTGCATGAGGTGGCAGAGCGTGGTGGTCCC
GGATTTACTCCCATTATTACTTTCAGCAGTAATGGGCAGTCTTTTAATGTGACGGAAGGA
AGTGGCACTTCAGCGCAGTATTTTCGCGCCTCTGTTCCGGTGTATGACCCGGATACAGGA
AATGCGTCTGGACAGCTTTCCTTTACCCTGAATCAGGGGATAGCAGTCAGTGTTGGTGCA
CAGGAAGAAGTGCGGACAGTGCCGGCAGGGATGTCACTGGTTAGCGGACAAAGCGTGAC
T
GACGTACAGTCAGGAACACTACCGCAGTGGCTTAAAAACCGTCTTTCCACATTACTTCTT
ATGAATCAGGGATTTGGTAATGGTATGAGTGCTACTGATAATGGTCAGGTTATCTCTCAA
GGGGTGCTGGCTGACGGACGTGTGATGAACCTTGCAGCGTCATATGCCTCAGCAGTGTCG
GATTTTGAACTGCGGTTGCAGGCAGAGAATACACCGTTCCGTTGGCAGGCAGGTCTGAAT
GTTACGGTCACAGTACAGTGA
>CS13_cshG X71971 ~~~cshG~~~CS13 CshG~~~~
ATGAAAAGGGTAACGTTATTTCTGCTTGCTGTCAGCCTTCTGCCTTCCTGTTTATTAGCT
TGGAATACACCGGGAGAGGATTTTAATGGAGACCTTACGCTGGAGGGGCATGTAACAAGC
ACTCGTAATCCCTGGGTGTGGAAAGTGGAGTTGACTCCCGTTATCTGGGACTCAACCGCC
CCGGCCGTGCCGGTTGGCGAGGGGGAGGTGATGATCCTGGTTCCTGTGCCAGAATTGTCT
GTATTACTGGGAAAAACAGCATTCACAACGACGGTTGGACGTGAAGGATTGTCACCGCGG
GTTATCTATGGCAAGGGTATGGCGAATTTCAGTCTGTCATGGACGACGCCGGGAACAGCT
GAGGTGGTACTACCTGTGTCAGGTGATGGTAATACAGTATCAGGACGTTTTGTTTTCCGC
CTGCAGGCTGCAGGTGTGTTGCGTCATACGCAGGGCGGGAAGTCAGTGTATACCGGTGTC
TATGACGACCTGAATGCGAATGGTCTGCCGGGAAAGATGCAGATGATGCCGGCAGAGGAG
GTGCCGGGAATACTTCTGTCGATGTTTACAGGAGAGGGGCCTTCATGGTTGCAGGTAATG
AAGGTTACCGGTTCAGATGGTTTGAGTCGTTTCAGTGATGCAGCTCTGCGTCAGGTTGAG
GGTGTGTATGGTGCACAAATAATGGCCGGCTACGGTAAGTTATATCTGAAAGGGCAGGTT
CCGTCGATTTGGCATGGTTCACTGCCGGTGAGTATTGAGTATCAGTAG
>CS14_csuB AY283611 ~~~csuB~~~CS14 CsuB chaperone~~~
ATGCATAAATTATTTTATTTACTAAGTTTACTCATGGCTCCATTTGTTGCAAATGCAAAC
TTTATGATATATCCAATATCAAAAGATTTAAAGAATGGAAATAGTGAGTTAGTTCGTATT
TATTCAAAATCAAAAGAGATACAATATATAAAAATATATACAAAAAAGATTATTAATCCC
GGTACAACTGAAGAACATGAAGTTGATATGCCCAATTGGGATGGTGGGTTTGTGGTCACT
CCTCAGAAAGTTATCCTTCCTGCAGGAGCGAGTAAATCAATACGTTTAACTCAATTTAAA
ATACCAAAAAAAGAGGAAGTTTATAGAGTATATTTTGAGGCGGTAAAACCAGATAGCAAA
GAAAATGTAATTGATAATAAAAAACTAACAACAGAACTATCTGTTAATATAATCTATGCG
GCTCTAATCAGATCTTTACCAAGTGAACAAAATATATCACTAAATATTTCTAGAAATGCA
AAAAAAAATATAATTATTTATAATAACGGGAATGTTAGAGCAGGTGTTAAAGATATTTAT
TTTTGTAAGTCATCTAATATCGATGATAACTGTGTAAAAAAAGCGCATAACAAGAATATA
TATCCAGAAAAGTCATTTGATACGCTGGTTAATAACAATTTTTCTTATGTTTTCATTAAA
TTAAACCATGAAGGCATAGAAAAAGAACAAGGGCTAATACAATTAAAAGTTCCTTGA
>CS14_csuA1 AY283611 ~~~csuA1~~~CS14 CsuA1 major subunit~~~
ATGAAATTAAAAAAAACTATTGGCGCAATGGCTCTGAGCACAATATTTGTAGCGGTGAGT
GCTTCAGCAGTAGAGAAAAATATTACTGTGACAGCCAGTGTTGATCCTACTATTGATATT
CTTCAAGCAAATGGTTCTGCGCTACCGACAGCTGTAGATTTAACTTATCTACCTGGTGCA
AAAACTTTTGAAAATTACAGTGTTCTAACCCAGATTTACACAAATGACCCTTCAAAAGGT
TTAGATGTTCGACTGGTTGATACACCGAAACTTACAAATATTTTGCAACCGACATCTACC
ATTCCTCTTACTGTCTCATGGGCAGGGAGGACATTAAGTACAAGTGCTCAGAAGATCGCA
GTTGGCGATCTGGGTTTTGGTTCCACCGGAACGGCAGGTGTTTCGAATAGTAAAGAATTA
GTAATTGGAGCAACTACATCCGGAACTGCACCAAGTGCAGGTAAGTATCAAGGCGTCGTT
TCCATTGTAATGACTCAATCGACAAACTAA
>CS14_csuA2 AY283611 ~~~csuA2~~~CS14 CsuA2 major subunit~~~
ATGAAATTAAAAAAAACTATTGGCGCAATGGCTCTGAGCACAATGTTTGTAGCGGTGAGT
GCTTCAGCAGTAGAGAAAAATATTACTGTGACAGCCAGTGTTGATCCTACTATTGATATT
CTTCAAGCAAATGGTTCTGCGCTACCGACAGCTGTAGATTTAACTTATCTACCTGGTGCA
AAAACTTTTGAAAATTACAGTGTTCTAACCCAGATTTACACAAATGACCCTTCAAAAGGT
TTAGATGTTCGACTGGTTGATACACCGAAACTTACAAATATTTTGCAACCGACATCTACC
ATTCCTCTTACTGTCTCATGGGCAGGGAAGACATTAAGTACAAGTGCTCAGAAGATTGCA
GTTGGCGATCTGGGTTTTGGTTCCACCGGAACGGCAGGTGTTTCGAATAGTAAAGAATTA

GTAATTGGAGCAACTACATCCGGAACTGCACCAAGTGCAGGTAAGTATCAAGGCGTCGTT
TCCATTGTAATGACTCAATCGACAGACACAGCCGCGCCTGTTCCTTAA
>CS14_csuC AY283611 ~~~csuC~~~CS14 CsuC outer membrane usher~~~
ATGATTTATAAAAAATTCAGAAAAAGAAAAATATCTTTATTTATTTCTATGTTCTTTATA
TCATGCTCAGTTTTTGCAGATGATATACCCGACTCTTTCCGTGATTTATGGGGAGAACAA
GATGAATTTTATGAAGTAAAACTATATGGGCAAACTCTAGGAATACATCGAATTAAAACA
ACCCCAACACATATTAAGTTTTATTCACCCGAAAGCATTTTAGATAAAATAAATTTAAAA
AAAGAAAAGGAAAAGGAATTGAGTGTTCTTTTTACTAATTCTTTTTCAAGAAATGGCAAT
ATGAGTTGTCAGGGTAACGCTGCTATACAGTATAACTGCAATTACATTAAAACAAAATCA
GTAGATGTCATCGTTGATGATGTTGATAATGTTGTTAACCTTTTTATAGGTAATGAATTT
CTGGATTCTGAAGCACACAGTGATGAATACCATCAATTATCACGAAATGTAAAAAAAGCT
TTTATACAAAGCCAGACAATTAATGTCTCAGATTCTGGGAAGTATAAAAATCTGTCTATT
TCAGGGAATAGTGCACTGGGTATTATAGGTACAAGTTATGCTGTCTTAAATTGGTGGATG
AATTACAATAAATCTAATGGTTACAGCAACAACGAAAAAACAATCAATAGTTTATACTTT
AGACATGATTTAGATAAGAGATATTATTATCAATTTGGACGAATGGATCGTACAGATTTG
TCACAAAGTATTAGCGGGAGCTTTAATTTTAACTTACTTCCTTTACCCGATATTGATGGC
ATACGGACAGGAACCACACAATCTTATATCAAAAATACAGATAAGTTTATCGCATCCCCT
GTAACTGTTATGTTAACTAATTTTTCCAGAGTGGAAGCTTTTCGCAATGATCAATTATTG
GGCGTATGGTATTTAGATTCTGGAGTAAATGAATTAGATACAGCTCGTTTACCTTATGGT
AGTTACGATCTTAAATTGAAGATTTTTGAAAAAACTCAGTTAGTTCGTGAAGAAATAATT
CCTTTTAATAAAGGGAGAAGTTCTATTGGTGATATGCAATGGGACGTTTTCGTTCAGGGA
GGGAATATTGTTAATGACAAGGGTCGTTACATAGAAAAACAATATAATCATAAGTCATCA
GTTAATGCTGGGCTACGTTTACCAATTACGAAAAATATCTCTGTTCAACAAGGAGTATCT
GTTATAGATAATAAAAATTATTATGAAGGGAGTCTGAAATGGAATTCCGGCATTCTGTCT
GGTTCACTAAATAGTGAGTTCAGTTTTCTTTGGGGAGATAATGCAAAAGGTAATTATCAA
AGTATCTCGTATACCGATGGATTTAGCTTATCATTTTATCATAATGATAAGCGGGTCGAT
AATTGTGGAAGAAATTACAATGCTGGTTGGAGTGGATGCTACGAATCATATTCGGCATCT
TTAAGCATTCCTTTATTGGGATGGACAAGCACTCTGGGATATAGTGACACTTATAGTGAA
TCAGTTTATAAAAGTCATATTCTTTCTGAATATGGTTTTTATAATCAAAATATATATAAA
GGGAGAACCCAAAAATGGCAACTGGCTTCATCCACATCTTTAAAATGGATGGATTATAAT
TTTATGCCAGCAATTGGAATATATAACAGTGAGCAAAGACAACTGACTGATAAAGGCGGA
TATATATCTGTAACTCTCACCCGAGCCAGCAGAGAAAATTCATTAAACGCAGGGTATTCT
TACAACTATTCCAGAGGAAACTATTCTTCTGACGAATTATTTGTTGGTGGATATATGACA
TCAACAAATAATGGTGACTATCATGAGGTAGGAATGCGTTTTAATAAAAATAGACATAAT
GCAGAAGGTAGACTTTCAGGTCGTATAAACAATCGATTTGGAGATTTAAATGGTTCATTC
AGCATGAATAAAAACAAAAACACCAACAGCACCAATCATTCTCTCACTGGTGGTTATAAT
TCCTCATTTGCTCTTACAAGTGATGGATTTTACTGGGGAGGAAGTGCATCTGGTTTGACA
AAACTGGCTGGCGGTATTATCAAGGTTAAATCAAACGATACTAAAAAAAATCTGGTAAAA
GTGACTGGGGCATTGTACGGTGATTATTCGCTAGGGAGCAACGATAATGCTTTTATTCCT
GTACCAGCATTAACTCCAGCCAGTTTAATTATTGAAGATAATAATTATGGTGACAAGAAT
ATTTCTGTACTTGCACCAACGAACAACGATATGTTTTTATTGCCGGGTAATGTTTATCCT
GTTGAAATTGAAACCAAAGTAAGTGTTTCTTATATTGGTAGAGGTTTTGACAAAAACGGC
ACGCCACTTTCTGGCGCACATGTTTTGAATGAACCACATGTTATCCTGGATGAGGACGGA
GGATTTTCGTTTGAATATACAGGTAATGAGAAAACACTTTTTTTATTAAAGGGCAGAACT
ATTTATACATGTCAACTGGGGAAAAATAAAGTTCATAAAGGCATTATTTTCGTCGGAGAT
GTTATATGTGATGTTAATAGCACACGTTCCTTACCAGATGAATTTGTAAAGAACCCACGT
GTGCAGGATTTGCTGGCAAAGAATGATAAAGGATAA
>CS14_csuD AY283611 ~~~csuD~~~CS14 CsuD minor subunit~~~
ATGAATAAGATTTTATTTATTTTTACATTGTTTTTCTCTTCAGTACTTTTTACATTTGCT
GTATCGGCAGATAAAATTCCCGGAGATGAGAATATAACTAATATTTTTGGCCCGCGTGAC
AGGAACGAATCTTCCCCCAAACATAATATATTAAATGACTATATTACAGCATACAGTGAA
AGTCATACTCTGTATGATAGGATGATTTTTTTATGTTTGTCTTCTCAAAATACACTTAAT
GGAGCATGTCCAACCAGTGAGAATCCTAGCAGTTCATCGGTCAGTGGCGAAACAAATATA
ACATTACAATTTACGGAAAAAAGAAGTTTAATTAAAAGAGAGCTACAAATTAAAGGCTAT
AAACGATTATTGTTCAAAGGTGCTAACTGCCCATCCTACCTAACACTTAACTCAGCTCAT
TATACCTGCAATAGAAACTCGGCTTCAGGTGCAAGTTTATATTTATATATTCCTGCTGGC
GAACTAAAAAATTTACCTTTTGGTGGTATCTGGGATGCTACTCTGAAGTTAAGAGTAAAA

AGACGATATGATCAGACCTATGGAACTTACACTATAAATATCACTGTTAAATTAACTGAT
AAGGGAAATATTCAGATATGGTTACCTCAGTTCAAAAGTGACGCTCGCGTCGATCTTAAC
TTGCGTCCAACTGGTGGGGGCACATATATTGGAAGAAATTCTGTTGATATGTGCTTTTAT
GATGGATATAGTACTAACAGCAGCTCTTTGGAGCTAAGATTTCAGGATAACAATCCTAAA
TCTGATGGGAAATTTTATCTAAGGAAAATAAATGATGACACCAAAGAAATTGCATATACT
TTGTCACTTCTCTTGGCGGGTAAAAGTTTAACTCCAACAAATGGAACGTCATTAAATATT
GCTGACGCAGCTTCTCTGGAAATAAACTGGAATAGAATTACAGCTGTCACCATGCCAGAA
ATCAGTGTTCCGGTGTTGTGTTGGCCTGGACGTTTGCAATTGGATGCAAAAGTGGAAAAT
CCCGAGGCCGGACAATATATGGGTAATATTAATATTACTTTCACACCAAGTAGTCAAACA
CTCTAG
>CS15_nfaA X64623 ~~~nfaA~~~CS15 NfaA major subunit~~~
ATGCGTAGTAAATTATCCATTCTTATTTTTGCTTTGATGACCTGTGGTGCCGCAAATGCC
GCAACTGCAGTAGGTGATGTTGCCACTGTTCGGGCTCCACTAGTATTTAGTGCACAGAAT
ACCATCAACGCAACCTGGACTCAGGATTCCTCTGTATCTGGTTCTTCAGTTACGGCAATG
CAAAAACTGGGTACTCTTAATATTCGATTAACCGGTTCTCATGCAGGGGTCTATGTTTCA
GGAGATGATACGGGGGAAAGTGGCGGCCTCATTACTATCCCATTTAAAAATACTGCAGGT
CAGGTTCTGTTCCGTGGACGTACTAATGCCGAAATTGGACAAGCGATGACGACGCCCATA
GTAGGCCACAGTGGACCGGGTTGGCATTTGCCAGGCACTCAGGATAATTTTAATCTTGAC
ATTCGCGCCTTCCAAAATGCGAATAATATCCCTGCTGGAGAATATACAGCAACATTCTAC
ATTCAACAATATCAGAGCTAA
>CS17_csbB AY515609.1 ~~~csbB~~~CS17 CsbB chaperone~~~
ATGCGAAAATTATTTTTAAGTCTGCTTATGATTCCCTTTGTTACGAAGGCGAACTTTATG
GTTTATCCTATATCAAAAGAAATCAAAGGAGCCAGTAGTGAAATTATTCGTATATATTCT
AAATCAAAAGATACACAATATATAAAAGTGTATGCTAAAAAAGTTGTAGGTCCGGGTACT
AAAGATGAGTATGAAATAGACGCACCTAATTGGGAAGGGGCGTTGGTTGTAACTCCAGCC
AGAGTGATTTTGCCTAGTGGTTCAAATAAATCGGTGCGTTTAACACAGCTAAATGCACCG
ACGACCGAAGAAGTTTATAGAGTGTACTTTGAATCCGTTAAGCCTGAGGCACAGGAAAAT
TCATCTGGAAACAAAACGTTAAAAACAGATTTGTCCGTTAATGTTATATATGCGGCTCTG
ATTAGAATTTTGCCAATAAATATTAAAACGAATGCGAGTGTATCGATATCACCTAGTAAT
AACATTCTTATAAAGAATACTGGTAATGTAAGACTTGGTATTATGAATGTTTTCTTCTGC
AAAAGCTCAAGTATTAATGATGATTGTGTGAAGAAAAGCTATAACAAAAACGTTTATCCA
GGATATTTATTAGACACAATGATTTACAATGATGGTTATTCGCACTTATTTCTTGATACC
AAGGATGAAAGTTCTAATAAAACAGATAATTTGATTCAGGTACCTATTATCTGA
>CS17_csbA AY515609.1 ~~~csbA~~~CS17 CsbA major subunit~~~
ATGAAACTGAAGAAAACAATTGGCGCAATGGCTATGGCGACTCTGTTTGCCACCATGGCT
GCCTCTGCAGTCGAAAAAAATATTACTGTGAGGGCAAGTGTTGACCCTAAACTTGATCTT
CTGCAAGCAGATGGAACTTCACTGCCGGACTCTATCGCATTAACCTATTCTTCGGCTTCA
AATAATTTTGAAGTTTACTCTCTTAATACTGCTATTCATACAAATGACAAAAGCAAGGGA
GTTGTAGTGAAGCTGTCAGCTTCACCAGTTCTGTCCAATATTATGAAGCCAAACTCGCAA
ATTCCGATGAAAGTGACTTTGGGGGGGAAGACGCTGAATACAACTGATACTGAGTTTACT
GTTGATACTCTGAACTTTGGTACATCTGGTGTTGAAAACGTTTCTTCCACTCAACAGCTT
ACGATTCATGCAGACACACAAGGAACTGCGCCTGAGGCAGGCAATTACCAAGGTATTATT
TCTCTTATCATGACTCAAAAAACTTAA
>CS17_csbC AY515609.1 ~~~csbC~~~CS17 CsbC outer membrane usher~~~
ATGGTTAGTAAAAATTTTAGCAAGCTATCTATAGTATTATTCTTTTTTCTCAGCTCTCCT
CTTGGATTTGCTGATAAGAATAAAATTACTAATATACCTGAATCATTTCGAGATTTATGG
GGAGAACAGGATGGATTTCTCGAAGTTAGATTTTATGGACAATCTCTTGGAGTTCATCGT
ATTAAATTAACTCCTACTACTGTGGCATTTGAGTCTCCGGATAGTTTATTAGATAAAATT
GAGATTAATAAAGAAAAGGAAGCTGACTTAAGAGTACTTATGCGAGGTTCATTCCAACGG
AATGGAAATATGAGTTGCCAGGGATATACGGGACAGAACAACTGCAATTACATTAAAACA
AACACAGTTGCGGTTATCGTAGATGATGTTGAAAATGTACTTAATCTTTTTATAGGAAAT
GAGTTTCTTGCTTCCGGAGAGAATGACAGTGATTATTATCAGCCATCCAAGAACACAAAA
AAAGCATTCATACATAGCCAGACAATTAATTTATCTGATACCGGTAATTATGAAAATTTG
TCCATTGTCGGGACGGGTTCGCTTGGGATAACAGATAACAGTTATGCTATTTTGGGTTGG
GCAGCAAATTATAATCGGTATAAATCTTACAATTACAATGAACAGTCGATTAACAGCCTA
TATTTCAGACATGATTTTGAAAAAAATTTTTACTATCAGTTGGGACGGATAGACAGAACC
GACTTATCGCAAAGTAGTGGTGGAAACTTTAACTTCGATCTACTTCCTGTACCTGATATT

TATGGTATGAGAGCCGGAACGACTCAGTCATATATCAAGAATACGGGAAAGTCAGTTGCA
TCTCCGGTCACAATTATGCTGACCCACTTTTCCCGTGTAGAAGCATATCGAAATGGGCAA
TTACTGGGAGTTTGGTATTTAGATGCAGGTATCAGTGAGTTAGATACGGAGCGTTTACCT
GACGGAAATTACGATCTGAAATTAAAAATATTTGAGCAGGAGCAACTTGTCCGTGAAGAA
ATTGTACCTTTTAACAAATCAGGTTCTTCAATTGGTGACACGCATTGGGATGTTTTCGTG
CAGGCAGGCGATATTATAAATGATAATGGCCGATATGTTGAAAAACAGAAAAACCATAAA
TCAGCCATTAATAGTGGATTACGTTTACCGTTAACAAGGAATCTTGCAGTACAGCTGGGG
GGGGCTGTTATTGATAATAAAAATTATTACGAGACTGGGATTCTGTGGAACTCAGGATTC
CTTGATGGTTCTCTCAATAGCAAATTCACTTTCCTTTTTGGTGACGACACACATGGAAAC
TATCAGAATGTTTCCTATACTGATGGTTTCAACTTATCGTTCTATCATAATGATAAGCGA
GTTAATGATTGTGGTAAAGATTACAATATGGGATGGAGTGGATGTTATGAGTCTTATTCA
GCGTCTTTAAGTATCCCTGTGAAAGGGTGGAATAGCACTCTTGCATACAGTAATACGTAC
AGTACGTCTGTATACAGATATGATGCTGTTTCTGAATATGTTCCTTATTCCTATTATAAA
GGAAGAACTAAAAGATGGCAACTTACTGCTTCTACGGTGGTAAGATGGGGGGACTATAAC
ATTATGCCAACAATAGGAGTATATAATAGTGAACAGAAACAATGGGCTGATAAAGGAGGC
TATTTATCCTTAACGCTTACTCGAGTTGATGGTGGCAAGTCCTTGAATGCTGGTTATTCC
TATAACTACTCCCGGGGTAATTATACTTCTAATGATGCATTTGTTGAGGGGCGTCTGGTT
TCAGATACAAATGTCAGTTATCGTGAACTAAGCGCACGCGTCAGTGGTAATAGATATTAC
ACTGAGGGAGGTGTTTCAGGGCGCATTAACAATAGATTTGGTGATCTGAATGGGACACTT
AGTGTTAATAAAAACAGAAAATCACATGATACAACTCACTCTCTGACAGCCGGTTACAGT
TCGTCATTTGCTCTTACGACCGATGGCATCTACTGGGGAGGAAGCGCATCCGGGCTGACG
AATTTATCCGGAGGGATTGTAAGAGTAAAATCAAATGAGGATGAGAGTGAACTATTGAAT
GTGAAAGGCTCATCATATGGTAATTATTCCCTAGGCAGCAATGATAGTTTATTTATACCT
GTACCTGCCCTGATGCAAGCCAGCCTTACTATTAAAGAGAATACAAATAAATCTAAAAAT
ATTGATGTACTAGCACCAACAAAAAACACTTTTTTTATGTTGCCTGGAAGTGTTTATCCT
ATTGATGTTTCAGCCAATGTTAGTTTTACTTACGTTGGACGTGGAGTTGATGTTAAGGGA
CGACCTTTATCTGGTGCATATATTTTGAATGCGCAAAATATTGTGCTGGATGAAAATGGT
GGATTTTCTTTTGAGAGTTCAGAGAATGAGAAGGAACTCTTTTTATTAAAAGATAAAACA
ATTTATTCCTGTTCATTAGACAGAAGCGAAATGCGCAATGGTATTGTTTTCGTTGGTGAG
GTTGCATGCAATTCTACCATCAAAGAACTTCTTCCTGAAAAGTTGGTTACGAATTCTCGC
ATTCATGATTTATTAGCTTACAATCAGGATACTGAATGA
>CS17_csbD AY515609.1 ~~~csbD~~~CS17 CsbD minor subunit~~~
ATGAAAAAGATATTTATTTTTTTGTCTATCATATTTTCTGCGGTGGTCAGTGCCGGGCGA
TACCCGGAAACTACAGTAGGTAATCTGACGAAGAGTTTTCAAGCCCCTCGTCAGGATAGA
AGCGTACAATCACCAATATATAACATCTTTACGAATCATGTGGCTGGATATAGTTTGAGT
CATAACTTATATGACAGGATTGTTTTTTTATGTACATCCTCGTCGAATCCGGTTAATGGT
GCTTGCCCAACCCTTGGAACATCTGGAGTTCAATACGGTACTACAACCATAACCTTGCAG
TTTACAGAAAAAAGAAGTCTGATAAAAAGAAATATTAATCTTGCAGGTAATAAGAAACCA
ATATGGGAGAATCAGAGTTGCGACACTAGCAATCTAATGGTGTTGAATTCGAAGTCTTGG
TCCTGTGGGCATTACGGAAATGCTAACGGAACACTTCTAAATCTGTATATCCCTGCAGGA
GAAATCAACAAATTGCCTTTTGGAGGGATATGGGAGGCAACTCTGATCTTACGCTTATCA
AGATATGGCGAAGTCAGTAGCACCCATTACGGCAATTATACCGTAAATATTACGGTTGAT
TTAACTGATAAAGGTAATATTCAGGTATGGCTTCCAGGGTTTCACAGCAACCCGCGTGTA
GACCTGAATCTGCACCCTATCGGTAATTATAAATATAGTGGTAGTAATTCACTCGACATG
TGTTTCTATGATGGATATAGTACAAACAGTGATAGCATGGTAATAAAGTTCCAGGATGAT
AATCCTACCTATTCATCTGAATATAATCTTTATAAGATAGGGGGCACTGAAAAATTACCA
TATGCTGTTTCACTGCTTATGGGAGAAAAAATATTTTATCCAGTGAATGGTCAATCATTT
ACTATCAATGACAGTAGTGTACTCGAAACAAACTGGAATCGAGTAACCGCAGTTGCTATG
CCGGAAGTTAATGTTCCAGTATTATGCTGGCCAGCAAGATTGCTATTAAATGCTGATGTA
AATGCTCCCGATGCAGGACAGTATTCAGGACAGATATATATAACATTTACACCCAGTGTC
GAAAATTTATGA
>CS18_fotA AF335469.1 ~~~fotA~~~CS18 FotA major subunit~~~
ATGAAAAAGACAATTATGTCTCTGGCTGTGGTTTCAGCTTTAGTAAGCGGTGTTGCATTT
GCTGCACTGCCTGCGAATGATTCAAGTAAGGCAACTCTTGACTTTACTGGCAATGTTACT
GCCAGCCTTTGCCAAGTGAAGACTGATAATTTAAGTCAGACTATTTCCCTTGGTGAGGTT
TCAAAGGCAGCTCTTACAACTAATGGGAAAGGAGAACCTCAGAGCTTCGAAATTAACTTG
GTTAACTGCGACACAACCACAGCCGATATTGCTTATGTGCTTGCGGATGCGAACAACAGC

ACCCCTTCCACTGGAACAGCACCTGATTATTTAGTACCGAAATCAGGTGATACCGCTGCA
GCTGGTGTAGGTGTGTTTGTCCAAACCAGCACCGGTGATAAAGTGACACCAGGGGCTACA
AAAACTCTGAAAGCTGATGCAAATAATGGCTCTGCCTATTCTGAACAGCGAATTCCTCTG
CGCGCATACATTGGTACCTTAAATGGCCAGCCAGGTGTTTTGGGCAATGCGGCCGGTCAG
GTTAAAGCTGGTACTGTTAACGCGACAGGTGTGTTGACTATTAAAGCCTCTGCAACAAAT
GTAAATCCGGTGCCTTAA
>CS18_fotB AF335469.1 ~~~fotB~~~CS18 FotB chaperone~~~
ATGTTTAAAAAATCAATTCTACTATTTTTTATATCAAGTAGCGCTATAGCTGGGGCATTT
GGGCCAAGAGAAAGTAAACTTATTTTTGAAAGTGACAAGGCTTATATTCAATACCGCATT
GACAATACTGGTAAAGACATGCCTTGGCTAGTTCAAGCTTGGGTAGAAGATAGTAAAGAA
AAAAAAACAAAAGAATTTACGCCAACTCCTCTTGTCTTTAGAGTGGAACCTTCATCCGTA
TTCTCTGTGCGAGTAATGAAAACTGGTTCCCCTGATGAACACAAGGAAACTCTCTACTGG
ATTGTATCAAATTCTTTGCCTGGAGGAGATAAAACAGAACTCAAAAGCCACGATGATAAA
ATAACCGCAAAAATGAATTTGGCATATCGATTTAAAGTGCCAATGTTTTATCGCCCAATT
TCATTGAAAAATATACCTCAACAACCTGAAAATCTGGAGTGGTCAGTTGATGGTAAGGGA
AAAGTTAAAGTAAAAAATTCCAGTAGATATATTGTTCAACTGCAAAGTATAAACATCCAT
AATTCTCTCCATCAAGGAAAAGGGGTTTCCTATTTTATTAATCCTATGAGTGATGTTGCT
TTGAATGTTAGTGCGAAATCCGGAACCAAGATACATTATAGTGTCATAAACGACTATGGC
GCGGTAAAAGAGTATGAGGGTGTTATCAAATAA
>CS18_fotC AF335469.1 ~~~fotC~~~CS18 FotC chaperone~~~
ATGAGGGTGTTATCAAATAAATTTTTAAATTTAGCAGGGGTTGTTTTTTTATTTCTTTTT
TCATTTGTCTCCTTTGCTAAATCTCCAGAATTAGGAGTTGCGATCGATCCGCTAAAAATA
AAAGTAAAACCTAATCGGATGGTTTACTTCAGCGTAATCAATGATACAGAAAACGATTAC
ATTGTAACAACAAAAGTAGTAAACGCTTTAACTAAAAAAGATTCTGATGCTGAACCACGT
TTTTTAGTCAACCCACCAATACGGTTACTAAAGAAAAGAGACAAGGCACAAATGGGGGTG
GTATATCTATCAGAACGACAACTGCCTCCACCTGACTCTAAAATTTATCTTTCTGTTTCA
TTTATTCCTAAGGTTCCTGATAAGTCAGCTTTGGTTCATATGCCAGTTATTTTTGTACAG
CAAGTACCATTAGTTTTTGAATAG
>CS18_fotD AF335469.1 ~~~fotD~~~CS18 FotD outer membrane usher
ATGAGTAAACATCATCTTATTCTTGCTCCTCTAATTTTAGGCATTGGCACTAATGTTGAT
GCCCGGGATTATTTTGACCCTTCTCTTTTAGCGTCAGATGTGGCGGGCGGGGGTGATATT
GATTTGTCTGCATTTGCAATCCCTGGTGGCGGTATGGAGGGTGAGCAAGAAGTAGGTATA
TACATCAATAATGATTTTTATTCAAGAGCAACGCTGAACTTCAAGAACACTGATGAGCGG
GGGTTGCTGCCGGATTTTCCTGCAGATTTTTTTGACGACATTCTATATGAAGAATATTTG
GTTTTTGACAAAACCCAGGTCATTCCCTCTTCAGACTTTCTGGCTAAAGTACCGTTTAGT
GAGGTTACCTTTGATCAGGCTTTGTCTCGAGTAAATGTTAGTATTCCACAAGCTTATATA
GATGAGGGAGCAAAACTTGTTTCTTCCCCTGATACATGGGACTACGGCGTCCCCGCACTA
CTGTTTGATTATAATATATCAGGGAACCGTAACAAATATAATGACTCTAATTCAGAGAGT
TTTTATGCATCTTCGTTGGTAGGCGTTAACCTGTGGGGATGGCGTTTACGCACTTCGGCT
AACTACAATCAGTATACGACCGAATCAGTGTGGGGAAAAACACGCTCGGAGACGAACAG
C
TTCTTCAATACATACATTGAACGAGATATTAGCTCTTTGCGTGCCGGATTGCGCATAGGT
GAGGCTTCGACAGGGGGTATTATTCTTGATTCAATACCATTCCGGGGGATAAAGCTTTAC
AGCGACGATGATATGTTGGGATATCGCCTCAGAAACTACTCGCCAACTGTGCGAGGTATT
GCCCGTAGCCAAGCTGTTGTAACGATTAGTCAAAATGGTAGACAAGTATATCAGACAAAC
GTTCCGCCAGGGCCTTTTCAACTTAATGATTTTTATATCTCTGGTTATTCTGGCGATATG
GTCGTGACCGTTCGTGAGGCTGATGGAAGTGAACATAGTTTTGTCCAGCCGTATTCTACA
TTACCCGAAATGAAACGTGAGGGCGTTTCTGGTTTTGAATTGTCCGTGGGCAAGTATGAT
AACTATGGTTCTGATGATTATTATGATCAACCTTCTTTTGTTTATGGCAACTGGTCACGG
GGTTTTTCGTATGGCATCACAACTTTCGGTGAGACACTTCAGGCTGAAAAATATCAAAGC
TTAGGGCTAGGAAGCACTGTTTCTTTGGGAGCGCTTGGTGCGGCATCAGCGGATGTGTCT
GTTTCTCGCGCAGAAAAATATGGTGATGTTAAGACTGGTCAATCTTATGGATTTAAGTAT
TCCAAGAGCCAACTTGAGACTGGTACTACTTTAACACTGGCGACTTATCGTTACTCGACA
AAGAATTTTTATACCTTCAGTGACTTTGCTTCTAACTCAGAAGAAGCTCGTTTTGTATGG
GAAAACAAACTCAAGAACAGAATGACTCTTAGCGTAAGTCAGGCTTTAGGGGCGTATGGC
CATCTGTCACTCAGTGCCAATCAGCAGAGTTACTGGACGAGTAGTGAGGTAACGCGAAAC
TATAGTTTGTCACATAGTTTTAGCTGGGAGGACATCTTCTTTAGTACAACGTTTTCTCTT

GATCAGTCACACAATCGTTATAACGATTACGCTGAGAATAAACAGATTGATTTTTACGTT
AGCGTGCCTTTGAGTAAGTTCCTTGGCGATAAGGATATTACCTCAAGTTCTCTAACATAC
AATGTGACAAACTCAGATCATCGAGTTCGCAACAGTGCGGCCTTAAATGGGAACATTCCA
GAAACTGATTTCCGGTATCGTGTGGGCGGTAGCTGGGGTAATAATAATCTTAATAGTACT
AAAACAGCGTCTCTTAGCTGGACGGGCAGTTATACCAGTGCTTCTTTAGGGTATACCTAC
TCTCATAATAACAATACTTTAGATTACAGTTTATCAGGAGCGGCCGTTGCATATCCTTGG
GGGCTTGCATTGGGTAACAACAATGTAACTAACAGTGGCGCTATTGTTGTCGAAACGTCT
GGAGCTCCGGGGGTCAGAACCAGCGCTGGATATAGTACTTCTTTCTTAGGTACCGCACTA
ATAGGTTCGCCGCAAAAATATACAGAAAACAGAATCGACCTGTATCCAGATGGTCTTCCG
GATGATACGGTTTTGGCTGAAACATCGAAAGTAGCAGTACCTGCTAAGGGAGCTGTTGTT
GTATTAGATTACAAAGTTTTTAAAGGAAGCCAGGTTGTTTTCTCACTAAAACAGGAGAGT
GGTAAACCACTACCTTTCGGCACGATCGTTTCTCTCGATGGAATGCCAAAAGGAAAAGAA
AATACAGGCATTGTGGGTGAAGACGGGCAAGTGTATATGGCAGGGGTCCCCTCTAAAGGC
TCTTTAAAAGCTGTATGGGGGGGTAACACTTGTGGTATTAAGTTCCATTTGGACGATAAA
AAAACAGTGGGCCCAATCAGAGAAACTACTGAGGTTTGCAAAGCATGA
>CS18_fotE AF335469.1 ~~~fotE~~~CS18 FotE chaperone~~~~
ATGAAAGGTTTATTAATACTGTTACTGGTAATTGTGACCTTTAACACCCAAGGGGTATTA
GCTAATGTAGACTATCCTTACCCTGAGGATACTATCCTTAACCTGAATCATGACACAAAG
GATTTATATCTGAAAGGTAGTATTCGTATATCAAATCCAGGAAGTTTGGTTTGGTTGGTC
CAGACATGGACTGAGGACGAGAACAAAAGTCGATTCGCTGATGTTTATCCTGCTCTAATG
AGGCTTGAACCATACTCTAGCAAAGTACTAAAAGTTTACCAGAAAAGCACTCCTGATAGA
AAAGAATTGAAATGGTTGCTAATATCTTTTATTCCATCACAGGATAAAATTGGTCATAAT
CAATTGACTATACCAGTATTTTATAGATTGAAAATAATTGAAAAACACTAA
>CS18_fotF AF335469.1 ~~~fotF~~~CS18 FotF minor subunit~~~
ATGATTTGGTGTAGGGATATGATTAATAAGTTAATTTTTCTTTTAACCTTAGTTTCCTTC
GCAAACTCTTCGTTTGCCGAACAAATCAAACAAGAAGCATCTTTAAGTTTAAATGTTAAC
ATTCTCAACCCTGTTTGTAAGCTAGCGAGCGGGGATCAAACTTTATACTTTGATGATTTT
AATGCTTTGGATGTAGTTACTAACAATAATAAGCTAATTAAGAATGCAGTTCTTAATTTT
ACTGAATGTAGTGGTGTGAAAAAACTTAATATTTCATTTGTTCAGTCAGGACAAACCCCC
CCTATTGATACAGTTAATAACTGGATACCTAACAAAAGCGGAACAGATATGGCAGCTGGT
ATCGCTATTATTTTGCTCAATAACAATAACTCATTGATAAATCTAGGGCAAAAGATGGTC
ATTGACGTAGGACAGTCTGAAAGCTCAAAACAAATTACTCTAAAAGCGCAAGTCGTCCCG
ACAGATAAAGCTGGTAGTGGAATAAAACCCGGAAAACTGGAGACAGCAGTTGGCATTGA
G
ATTTCATATGAGTAA
>CS18_fotG AF335469.1 ~~~fotG~~~CS18 FotG adhesin~~~
ATGATTATGAAAAAAAAACTAAAATCCAAAAAAATTTGGATCTATTTCGCGTTGCTTGCA
GCCCCTAGTCATAGCTATGCTAATGACGGCGCAGGAGAGGCTTTTACCAAAGATATGACA
TCCACTAAGAATATTGAAGTTCCCATTTATAATTTCCCCGGCAGCTGGATTGAGCTTGGT
AACTTGGGAATAAGCAAGTATTCACAGGTTGATGGAAATCATCGTTGCTCAAATCTAACT
TTGTGTGGATATACTGCTGTTAGGGTAAATGGTGGTACAAGTGGTAATAAGGGGGCGTAT
TATCGCATGAAACTCGAAGCCTCCCCAGTTACAGTTTCCTCAAATGGTATAAATTTTGTT
TTTTCCGTTTATTTTAAAGGATCTCCATATTTAACATGGCAAGAAAGAAATATCAATAAT
AACAGAAATTGGTTTCATACTATTTATGTGCCTGTAAATAGTATTGGAAGTAGTGCAGGG
AGTACTACCAACTATTCTCAAAATTTAAATCCGTCAGGTGTTTGTGGTTCTTTAAGTGGA
TGTACATATGGAGCCACGACATATTTTACTTCGGGAGGAGATGCTTATCTAGCATTAAAG
GTACCTGAAAATTTAGCTGCTGGCACTTATCAATTCAGTGATGTTGAAGTTTTAAGCCTG
TGGCAACAGTCAAATAATGCTACATGGTTAAACCGTTATGAGGCCAAAGCTACGGTTAAA
ATTAGTGGAACCATTAAGTTACCTAACAGGTGCTATTTCTCAAGCTCACAAAATAACATC
AACTTTAGTGATGTTAAAATAAATTCAAACAATGGTAGCTTAGAGACTAAAGATTTTAAA
TTGCTTACTACCTGTCGAGGAATTCAGGTGAATGTAAAACAATATTTAACTGTCAGTTCA
GATGTGAACGATTATATAAAAGTATTTTCCTATGATGAAGAGGGGAATAAAGCTTTAGGT
TTTGCCATGCAAATTGCTCAGCAGGGATTATCCTCAAAAGAACCTGATTGTGATGCAAGA
AGTGAATCTTTGAACAAATTTAATAGCGAATATCTTATAAGAACAATACCGGCTTCATCT
TATCAAGCGTTTGAAGATACTGTTAAATTTTCATTATGTAAGTTTAGTGTTCCAGCATCA
AAATATATTGGAGCACATAATATACCGATAAAAATCATTTCCCGATGGGAAAGCTGA
>CS19_csdB AY288101.1 ~~~csdB~~~CS19 CsdB chaperone~~~

ATGCGAAAATTATTTTAAGTCTGCTTATGATTCCCTTTGTTACGAAGGCGAACTTTATG
GTTTATCCTATATCAAAAGAAATCAAAGGAGCCAGTAGTGAAATTATTCGTATATATTCT
AAATCAAAAGATACACAATATATAAAAGTGTATGCTAAAAAAGTTGTAGGTCCGGGTACT
AAAGATGAGTATGAAATAGACGTACCTAATTGGGAAGGGGCGTTGGTTGTAACTCCAGCC
AGAGTGATTTTGCCTAGTGGTTCAAATAAATCGGTGCGTTTAACACAGCTAAATGCACCG
ACGACCGAAGAAGTTTATAGAGTGTACTTTGAATCCGTTAAGCCTGAGGCACAGGAAAAT
TCATCTGGAAACAAAACGTTAAAAACAGATTTGTCCGTTAATGTTATATATGCGGCTCTG
ATTAGAACTTTGCCAATAAATATTAAAACGAATGCGAGTGTATCGATATCACCTAGTAAT
AACATTCTTATAAAGAATACTGGTAATGTAAGACTTGGTATTATGAATGTTTTCTTCTGC
AAAAGCTCAAGTATTAATGATGATTGTGTGAAGAAAAGCTATAACAAAAACGTTTATCCA
GGATATTTATTAGACACAATGATTTACAATGATGGTTATTCGTACTTATTTCTTGATACC
AAGGATGAAAGTTCTAATGAAACAGATAATTTGATTCAGGTACCTATTATCTGA
>CS19_csdA AY288101.1 ~~~csdA~~~CS19 CsdA major subunit~~~
ATGAAACTGAAGAAAACAATTGGCGCAATGGCTATGGCGACTCTGTTTGCCACCATGGCT
GCCTCTGCAGTCGAAAAAAATATTACTGTGAGGGCAAGTGTTGACCCTAAACTTGATCTT
CTGCAAGCAGATGGAACTTCACTGCCGGACTCTATCGCATTAACCTATTCTTCGGCTTCA
AATAATTTTGAAGTTTACTCTCTTAATACTGCTATTCATACAAATGACAAAACCAAGGCA
GTTGTAGTGAAGCTGTCAGCTCCAGCAGTTCTGTCCAATATTATGAAGCCAAGCTCGCAA
ATTCCGATGAAAGTGACTTTGGGGGGGAAGACGCTGAGTACAGCTGATGCTGAGTTTGCT
GCTGATACTCTGAACTTTGGTGCATCTGGTGTTGAAAACGTTTCTTCCGTTCAACAGCTT
ACGATTCATGCAGAAGCTGCTCCGCCTGAGGCAGGTAATTACCAAGGTGTTATTTCTCTT
ATCATGACTCAAAAAACTTAA
>CS19_csdC AY288101.1 ~~~csdC~~~CS19 CsdC outer membrane usher~~~
ATGGTTAGTAGAAATTTTAGCAAGCTATCTATAGTATTATTCTTTTTTCTCAGCTCTCCT
CTTGGATTTGCTGATAAGAATGAAATTACTAATATACCTGAATCATTTCGAGATTTATGG
GGAGAACAGGATGGATTTCTCGAAGTTAGATTTTATGGACAATCTCTTGGAGTTCATCGT
ATTAAATTAACTCCTACTACTGTGGCATTTGAGTCTCCGGATAGTTTATTAGATAAAATT
GAGATTAATAAAGAAAAGGAAGCTGACTTAAGAGTACTTATGCGAGGTTCATTCCAACGG
AATGGAAATATGAGTTGCCAGGGATATACGGGACAGAACAACTGCAATTACATTAAAACA
AACACAGTTGCGGTTATCGTAGATGATGTTGAAAATGTACTTAATCTTTTTATAGGAAAT
GAGTTTCTTGCTTCCGGAGAGAATGACAGTGATTATTATCAGCCATCCAAGAACACAAAA
AAAGCATTCATACATAGCCAGACAATTAATTTATCTGATACCGGTAATTATGAAAATTTG
TCCATTGTCGGGACGGGTTCGCTTGGGATAACAGATAACAGTTATGCTATTTTGGGTTGG
GCAGCAAATTATAATCGGTATAAATCTTACAATTACAATGAACAGTCGATTAACAGCCTA
TATTTCAGACATGATTTTGAAAAAAATTTTTACTATCAGTTGGGACGGATAGACAGAACC
GACTTATCGCAAAGTAGTGGCGGAAACTTTAACTTCGATCTACTTCCTGTACCTGATATT
TATGGTATGAGAGCCGGAACGACCCAGTCATATATCAAGAATACGGGAAAGTCAGTTGCA
TCTCCGGTCACAATTATGCTGACCCACTTTTCCCGTGTAGAAGCATATCGAAATGGGCAA
TTACTGGGAGTTTGGTATTTAGATGCAGGTATCAGTGAGTTAGATACGGAGCGTTTACCT
GACGGAAATTACGATCTGAAATTAAAAATATTTGAGCAGGAGCAACTTGTCCGTGAAGAA
ATTGTACCTTTTAACAAATCAGGTTCTTCAATTGGTGACACGCATTGGGATGTTTTCGTG
CAGGCAGGCGATATTATAAATGATAATGGCCGATATGTTGAAAAACAGAAAAACCATAAA
TCAGCCATTAATAGTGGATTACGTTTACCGTTAACAAGGAATCTTGCAGTACAGCTGGGG
GGGGCTGTTATTGATAATAAAAATTATTACGAGACTGGGATTCTGTGGAACTCAGGATTC
CTTGATGGTTCTCTCAATAGCAAATTCACTTTCCTTTTTGGTGACGACACACATGGAAAC
TATCAGAATGTTTCCTATACTGATGGTTTCAACTTATCGTTCTATCATAATGATAAGCGA
GTTAATGATTGTGGTAAAGATTACAATATGGGCTGGAGTGGATGTTATGAGTCTTATTCA
GCGTCTTTAAGTATCCCTGTGAAAGGGTGGAATAGCACTCTTGCATACAGTAATACGTAC
AGTACGTCTGTATACAGATATGATGCTGTTTCTGAATATGTTCCTTATTACTATTATAAA
GGAAGAACTAAAAGATGGCAACTTACTGCTTCTACGGTGGTAAGATGGGGGGACTATAAC
ATTATGCCAACAATAGGAGTATATAATAGTGAACAGAAACAATGGGCTGATAAAGGAGGC
TATTTATCCTTAACGCTTACTCGAGTTGATGGTGGCAAGTCCTTGAATGCTGGTTATTCC
TATAACTACTCCCGGGGTAATTATACTTCTAATGATGCATTTGTTGAGGGGCGTCTGGTT
TCAGATACAAATGTCAGTTATCGTGAACTAAGCGCACGCGTCAGTGGTAATAGATATTAC
ACTGAGGGAGGAGTTTCAGGGCGCATTAACAATAGATTTGGTGATCTGAATGGGACACTT
AGTGTTAATAAAAACAGAAAATCACATGATACAACTCACTCTCTGACAGCCGGTTACAGT
TCGTCATTTGCTCTTACGACCGATGGCATCTACTGGGGAGGAAGCGCATCCGGGCTGACG

AATTTATCCGGAGGGATTGTAAGAGTAAAATCAAATGAGGATGAGAGTGAACTATTGAAT
GTGAAAGGCTCATCATATGGTAATTATTCCCTAGGCAGCAATGATAGTTTATTTATACCT
GTACCTGCCCTGATGCAAGCCAGCCTTACTATTAAAGAGAATACAAATAAATCTAAAAAT
ATTGATGTACTAGCACCAACAAAGAACACTTTTTTTATGTTGCCTGGAAGTGTTTATCCT
ATTGATGTTTCAGCCAATGTTAGTTTTACTTACGTTGGACGTGGAGTTGATGTTAAGGGA
CGACCTTTATCTGGTGCATATATTTTGAATGCGCAAAACATTGTGCTGGATGAAAATGGT
GGATTTTCTTTTGAGAGTTCAGAGAATGAGAAGGAACTCTTTTTATTAAAAGATAAAACA
ATTTATTCCTGTTCATTAGACAGAAGCGAAATGCGCAATGGTATTGTTTTCGTTGGTGAG
GTTGCATGCAATTCTACCATCAAAGAACTTCTTCCTGAAAAGTTGGTTACGAATTCTCGC
ATTCATGATTTATTAGCTTACAATCAGGATACTGAATGA
>CS19_csdD AY288101.1 ~~~csdD~~~CS19 CsdD minor subunit~~~
ATGAAAAAGATATTTATTTTTTTGTCTATCATATTTTCTGCGGTGGTCAGTGCCGGGCGA
TACCCGGAAACTACAGTAGGTAATCTGACGAAGAGTTTTCAAGCCCCTCGTCTGGATAGA
AGCGTACAATCACCAATATATAACATCTTTACGAATCATGTGGCTGGATATAGTTTGAGT
CATAGATTATATGACAGGATTGTTTTTGTATGTACATCCTCGTCGAATCCGGTTAATGGT
GCTTGCCCAACCATTGGAACATCTAGAGTTGAATACGGTACTACAACCATAACCTTGCAG
TTTACAGAAAAAAGAAGTCTGATAAAAAGAAATATTAATCTTGCAGGTAATAAGAAACCA
ATATGGGAGAATCAGAGTTGCGACACTAGCAATCTAATGGTGTTGAATTCGAAGTCTTGG
TCCTGTGGGGCTCTAGGAAATGCTAACGGAACACTTCTAAATCTGTATATCCCTGCAGGA
GAAATCAACAAATTGCCTTTTGGAGGGATATGGGAGGCAACTCTGATCTTACGCTTATCA
AGATATGGCGAAGTCAGTAGCACCCATTACGGCAATTATACCGTAAATATTACGGTTGAT
TTAACTGATAAAGGTAATATTCAGGTATGGCTTCCAGGGTTTCACAGCAACCCGCGTGTA
GACCTGAATCTGCACCCTATCGGTAATTATAAATATAGTGGTAGTAATTCACTCGACATG
TGTTTCTATGATGGATATAGTACAAACAGTGATAGCATGGTAATAAAGTTCCAGGATGAT
AATCCTACCAATTCATCTGAATATAATCTTTATAAGATAGGGGGCACTGAAAAATTACCA
TATGCTGTTTCACTGCTTATGGGAGGAAAAATATTTTATCCAGTGAATGGTCAATCATTT
ACTATCAATGACAGTAGTGTACTCGAAACAAACTGGAATCGAGTAACCGCAGTTGCTATG
CCGGAAGTTAATGTTCCAGTATTATGCTGGCCAGCAAGATTGCTATTAAATGCTGATGTA
AATGCTCCCGATGCAGGACAGTATTCAGGACAGATATATATAACATTTACACCCAGTGTC
GAAAATTTATGA
>CS20_csnA KJ922517 ~~~csnA~~~CS20 CsnA major subunit~~~
ATGAAAAAAATGATTATGCCTTTAACTATGGTGTCCGTTCTGATGAGCGGCTCTGCTCTG
GCTGCGCCGGCTGCTAACGATTCCAGTCAGGCCACACTGAATTTTAGTGGTCGTGTGACA
TCCAGCCTTTGCCAGGTAAAAACAGATGATCTGGTAAAAAATATTTCTTTGGGTGAGGTA
TCCAAATCCGCACTGGAAGCTACTGGTAAGAGTCCTGCACAGAGCTTTCAGGTTAACCTG
ATTAACTGTGACAGCCTGACTGACGACATTTCCTATGTGCTGGCTGATGCGAACAATAAC
GGTACTACTACTGCTTACCTGGTTCCAAAATCAGGTGATACTGCTGCAACCGGCGTAGGT
GTTTTTGTGGAAACCAGTAAAGGTACTCCTGTGAATATTGGAAGCGATCAAAAACTTGAC
GTTGTAGCAAATAAAGGTAATGCTCTTTCTGAACAAGTAATTCCTCTGCGTGCCTATATT
GGTACTCAGACCCGTGCTGCCGGAGCTATAGGTACTGACGTTACGGCAGGTACTGTTGAC
GCAACTGGTGTTTTGACCATTCGTGCGGCGGATGCAACTCCGTAA
>CS20_csnB KJ922517 ~~~csnB~~~CS20 CsnB chaperone~~~
ATGCGTTATCAGTATTCTGTTTTATTCGTCTTTTTATTCTCTGGCGTTGTTCAGGCGGCG
GAGTCTTTCGGTCCGCGGGAAAGCCGGTTGCTTTTTGAAGAGAAGAAAGGAAACACCTT
T
TACCGGATTGACAATTCAGACAAAAAACTTCCCTGGCTTGTACAGGCATGGGTTGAAGAT
GCGTCTGAAAAGAAAACAACCGCACTGACAGCCACGCCCATGGTGTTTCGCGTTGAGCCT
TCTTCTGTCTTTACTGTCAGGGTGGTTAAAACGGGAGCCCTGCCGGAAGACCGGGAAACA
CTTTTCTGGGCCGTTTCCAATTCATTACCGGGAGGTGTCTCAACAAAGCAGGATAATGAA
GAGGGAAAAATCAGCGCAAAACTCAGCCTGGCTTACCGGTTTAAGGTGCCACTTATTTAC
CGGCCTGCAGCGTTGGATAATTTCAGGCAGGAGCCGGAGAAACTGGAATGGACATATAAT
GGCAAGGACGGTCTGAAATTATATAACCCCACCCGTTATGTGGTCCAGTTACATAACGTA
ACGGCGAACGGACGTGAATTTAAGGGTAAAGGGGTTTCGTTTATTCTTCTTCCGATGTCC
GGTAAAAAATGTGAGTGCCGCTGTGAATAAAGGCACCAGAATAAAATATGGTGTCATAAAT
GACTATGGCGCGGTAAAAGAATATGATGGTGTTGTTAAGTAA
>CS20_csnC KJ922517 ~~~csnC~~~CS20 CsnC chaperone~~~
ATGATGGTGTTGTTAAGTAATGCTCTAAAAAAAGCAGTGCTTTCCCTGTTGGCGTCCTGT

AGCATAGTTGTTTTTGCATCTGAATCAGAACTTGGCGTTGCTGTAGAACCGATGAGGTTA
TCAATTAAGCCTGGAGAGATGACATATTTTACGGTTATCAATGAGACAGAGAGAGAGTAC
ATTGTCACCACCAGGGTAGTAAGTGACTCAGGGATTGATGATAAAGATAAAGTATTTGTC
TTTAGTCCCCCCCTGAAGCATTTAAAAAAAAGGGAACAGTCTGTAATGGGAGTTGTATAT
CTGAAAAATGGGAGAGAGAATAAGATGAAATACTATTTATCTGTCTCATTTGTTCCCAAA
CTTTCTGAGGATAAGGTGAAAATATCTATTCCTGTAGTTCTTGTTCATCAAATACCGCTG
ATGTTTGAATAA
>CS20_csnD KJ922517 ~~~csnD~~~CS20 CsnD outer membrane usher~~~
ATGAATAAATATTATCTACTAATAATTCTTTTTTTTGTGAGGAGCACAAAGGTTTTTGCC
GAAGATTATTTTGACCCATCTCTTCTGGCAACCGATATTATTGGTGAAGGTAATATTGAC
CTTTCTGCATTCTCACGCCCTGGTGGAGGTATGGAGGGGGAACAGGAGGTCGCTATTTAT
GTCAATGATGAGTTTTATTCCCGTAATACTCTGTTTTTTAAAAATACTTTAGATAAAGGA
CTTTTACCTGAATTTACCCCGGGATTTTTTGACGAATTGTTGTCGGGGGATTTTCTTGTG
TCTGAAGAAGATAAAACAATATCATCATCAGACTTCCTGAAAAAGGTTCCTTATAGTGAT
ATAAACTTTAATCAAGGAATGTCTCGTGTCAATATCAGCATTCCTCAGGCATATCTTGGT
GATGGAGCAAAGTTGATCTCCTCGCCTGATACATGGGAATATGGGGGGCCGGCATTTTTG
CTAGATTATAATATTTCAGGCAACCGGAATGATTCAGGAAATTATGATTCCCGTAGTCTG
TATATCTCCTCACAGATGGGGGTAAATCTCATGAAGTGGCGTTTGCGTACATCCTCCAGT
TACAGCAATTATAAGACAAATTCTGTATGGGGAGGTGCTCGTTCAGAGCAAAATAGCTTT
TACAATACTTACGCAGAGAGGGATATTAGCTCGTTACGTGCAATATTACGACTTGGTGAA
GTTTCGACCGCAGGGTTAATACTGGATTCTGTTCCTTTCCGAGGTGTGAAGCTGTCAAGT
AGTGATGACATGCTGGGGATGCGTTTGCGTAATTACACACCCACAGTGCGAGGTATGGCG
AGCAGTCAAGCAGTTGTTACAATAACTCAGAATGGGCGACAGGTATACCAGACAAATGTA
CCAGCAGGGCCATTTGAACTGAATGATTTTTATCTTTCCGGCTATTCGGGGGACATGCTG
GTGACTGTCCGTGAGGCTGATGGTAGTGAGCACAGTTTCTTGCAACCTTATTCGACCCTT
CCGGAAATGAAACGCGAGGGAGTCTCCGGATTTGAGGTTTCGGTTGGTCGCTATGATAAT
AACGGTGCTGAACACTATTACGATGCTGAGTCATTTGTTTATGGTAACTGGTCTCGGGGT
TTTGCTCGTGGAGTGACATTTTTTGCTGAGACTCTTCAGGCAGAAAAATACCAGAGTCTG
GGGGGAGGGAGTACGTTGTCGCTGGGGAGGCTGGGGGCTGCATCTGCAGATATATCCTTA
TCACGAGCAGATAAATACGGTGACATCCGGATTGGTCAGTCTTATGGCTTTAAATACTCC
AAGAGCCAGATTGAAACGGGCACAACGGTAACATTGGCAACATACCGATATTCCACTGAG
AATTTTTATACTTTCCGTGATTTTGTTTCGAAAACGGATACAGCTCGTTATATCTGGGAA
AATAAACTGAAAAGCAGAATGACATTTAGCCTCAGTCAGTCTCTGGGGGAGTATGGCTAT
TTGTCAGCCAATGCCAGTCAGCAGGATTATTGGAATAGCCGAGAGGTGAGTCGAAATTAC
TCGCTGACTCATAGCTTTAGCTGGAATGATATTTATTTCAGTACAACGTTGTCCATGGAT
GATCAACGAGGGCGGGAGACTGGACATTTATCCAATAAGCAAGCAGGGATATATGCCAGT
GTTCCTTTAAGTAAGCTTTTACCTAGAACTGATCCGACGAGTAGTTCGCTAACTTGGAGT
ACTTCGCATGCTGATCATAAGGTACGCAATAGTGTGACTCTGGACGGGAAAGTTCCAGAA
AGTGACGTTCGTTATCGAGTGGGAGGCAGTTGGGGAAATGGAACCACTGAAGGCTCACG
T
ATGGCATCAGTGAGTTGGACTGGTGATCATGCAAGCACCTCGCTGGGATATACCCGTGTA
GGGAAATATCGAACCCTGGATTACAGTATGTCTGGCGCAGCGGTGATGTATCCATGGGGG
ATTGCTGTGGGTAACAGTAGTGTTACTGGTGATGGTGCTATTGTAGTGGAAACACCAGGA
GCTAAAGGAGTAAGAACTAGCACCGGATACAAAACTTCATGGTTGGGAACGGCTTTAATT
AGTTCACCACAAAAATATACAGAGAATCGTATTAATTTATATCCCGACGGTCTTCCTAGT
GACACCGTCCTTGGTGAAACATCTAAAACTGCCGTGCCTGCTAAAGGTGCAGTCGTTGTG
CTGGATTATACTGTCTTTCGTGGTAGTCAGGTGGTGTTCACACTGAGACAGACTGATGGT
AATCCATTACCGTTTGGTACAGTGATTACGCTTGATGGTGTATCCAGAGGCAAGGAAAAC
AGTGGTATTGTAGGTGAGGAGGGCCGAGTGTATATGGCGGGTATACCGGAAAAAGGAACT
CTCACTGCATCATGGGGGCTGAATAAAACTTGTAGCATACCATTCCGTATAAACCAGCAT
AAAGCTGAGGCCGTTATCAGGGAGGTTCAGGGAGTATGCCGTGTATGA
>CS20_csnE KJ922517 ~~~csnE~~~CS20 CsnE chaperone~~~
ATGCCGTGTATGATGTTACAGATCTTCATTACAATTTTACTGTTACTGCCTTTATCAGTT
ACGAAAGCAGATATATTATATCCGTGGCCAAGTGATACTGAAGTCAGGCTGAAAATAAGT
GACGAAAAGGGTCAGAGCAGGGGAGATCTACGAGTTACTAATCCTGGCGATGCACTCTGG
TTGGTTCAGGCATGGGCTGAAGATGAAAAATATAGACGATATAGCGTTATATATCCATCC
GTATACAGATTGGAGCCATTTAGTGCTTATGCGCTGAATATTTACCCTGGTAATAATATT

ATGCCAGAACGGCTGAAATGGTTTTTGATTTCTTTTATACCTTCGATAGTGGAAAAAAAC
AAAAACCAACTAATTATACCAGTTACTTACAGACTCAAAATAATTGATGATGTGGTATGT
AAGGGTATTGAAAAAGTAGCTGGTGAGTGTTAA
>CS20_csnF KJ922517 ~~~csnF~~~CS20 CsnF minor subunit~~~
ATGCTGAAGAAAATACCGTGCATAATATTAATGTCTTTACCTGGACTTATTTCTGCTGCG
GAAATAACAAAGCAGATAGAACTCACGCTAAAAGTTAATGTATTAAAGCCAGTATGTAAA
CTCAGTTCTGGGCAGCAAACAATAAACTTCGGTGATTTTGATGTGCTGGATGTTATTACG
AAAAGTAGTAAAGTGAATGGTAGCGCTACATTTAGATTTACTGAGTGTAGTGCTGTCAAT
AATATAAAAATAAAATTTAAGCAAGCCGGACAAAGCCCAGTTCCGGATATAGAAAACAAT
TATATCCCTAATGCTAAGGGGGATATAATGGCAAAGGGGGTGGCTGTAAAGCTTCTGGAT
GATCAAAAGAAAGAGGTCGAGTTGGACAAGATAATGAGCGTTAGTGTGGGGGAGAATCA
G
ATATCTAAAGATTTAACGTTAAATGCTCAGGTCATCTCTGTCAATAAAACTGGAGAAGGG
ATTTCGCCGGGAATGCTTCAAACGGCAATAGGGATGGAGATTTCATATGAGTGA
>CS20_csnG KJ922517 ~~~csnG~~~CS20 CsnG adhesin~~~
ATGTTGAAGTGTCTTTGTTTTGGCTATTTTACCAAAAGTCTAATGCAACGCATTATTAAA
TTAACGTATGGGGCTGGAGAGTATGCTACCATTTTTACCGTTTTATTTTTTTCTGCGCAT
AGATGTGCAATAGCAGTTGAGAATAACTATACGATATCAATCGACAAGACATTTGATATA
TCAAGTATTACCAGAGACTGGATAGAGTTAGGTGAGTTTCCGTATCCAAAAGCAGCAAGT
AATAATGAGACCCATTTCCGTTGCACTAAGGGAGCAGGAAAAGGTTTATGCGCACATACA
GAGTTTAGAGTGAATGGAGGCTTACAGAGTAACCCCGCAGCATACTGGAATGGATGGATG
AGGATGGAGCCTAAAGAGGTCAATATAGGAGATGGGGAAAAGTTAATTTTTTCAGCATAC
TGGAAAGGAAGCCCAAGCATAAGATGGGAGGAGACAAATAGAAAAAATATGAAGAGTAT
T
TTTCATCAATACACTATTGGTGTTGCTTCTATTGGCTCATCGGCCGGCTCAATAACTAAA
TGGTCTTCAGGCGAACAAGGACCATCATTGAGGTGTGGGGATTTGGGCGGATGTACAATA
GATACACATACGTACTTTGATAACAGAAGTGGAGGAGCATTAAAGCTAGCGGTCAAGCTT
CCGGCTGCTTTCAGGAAAGGCACGTATACATTTTCTAATGTTGAAGTGCTTGATTTGGGG
CATACAGCGCGAAATGCTTCTGGCACAACACAGCAGAGTGTCAGTGCTAAAGTTTATATT
AGTGGGAAAATTACTGTGCCTGAGAGATGTTATATTGATACAGGATCAGGTTCTGAAATA
AAATTTAATGATGTAAGCGCAGGGGCAAGTAATGGGGAGATTGATAAACGCAGTTTTACA
TTAAAAACAACATGCAAATATATTAATCTTAAACTCAATCAATATATAAAAGTAAATGGG
CGTAATGGTCTTTCCGAGTATGAGATTTTTTCCAGAGATAACTCAAATGAAAAAGCATTG
GCACTGGTGATGAGAATTGTTCGTGGTGACAATGGAGAGGACTTCTACGCAAATTGTGAA
CCTGACCATTCGGGTAGGGTATATTTTAGTAAAGAGTATCTCTTGAGAGAAATAAATGGC
AATGGATTGAACATACATACTTACAACGATATAATAAGGTTTTCATTATGCAAATATGGA
ATACCAAAAGATTATGGAGAAAAAATTATTCCACTTACTATTGTTTCGAGATGGAGCGAT
TCCTGA
>CS21_lngX1 LR883052 ~~~lngX1~~~CS21 LngX1 unknown function~~~
ATGGTGCGGGCTGAGGACTGGTTTGCCGTTTTTGGGGTAATGCCAGGTATGTATGGGGGC
GGTGCAGCAGAAGATGAAACACAGGAAAAAGCGTTGCAGGGTTCAGATAGTCTGCAGAA
A
ATGTACTCAGCATCATATCTCTGTCCGTATATATGGAGATGCGGAACAGAAATCAGAATG
TGCGGACGTACAGAGCCTCACAGAAAGACGCCTTCCTTTCACATATACAGAATACTGATT
ATTGTTTTTTACACTGGTGCAGCAGGCAGTTTTTTAAGGCATGATGTTAGGATACCTTGC
GTGAACAGGATAAATCGGCCTGAAGTGCTTTCTCTGTTATTCTCTTCACTTTCTTTTGCT
GTTGTTCATTTATTATCCTGA
>CS21_lngR LR883052 ~~~lngR~~~CS21 LngR regulator~~~
ATGTGTCAAAATAATGCAAATTCTGTTTCAGAATACGTACGTTTAAATATCATGGTGAAA
AAAGGCGAAGTATTACCCGGAACCATAGAAGGAGAGCATTTCTGGCTTCTTATGGAAATA
TTGCCAATTCACAGTAAAAAAATCATTAATGCCATGCGTGATCATCTCGTTTTCGGTATA
TCACGTAAAGAAGTCTGTGAAAAATACAGTGTCAATAATGGTTACCTGAGTATCAGTATT
GCAAAGCTAAATTACACCCATCAGATTGTCAGAAACATGATTCATTATTACAAGGACAGG
CAATGA
>CS21_lngS LR883052 ~~~lngS~~~CS21 LngS regulator~~~
ATGAGTGGTTATTCGTTAAAAACGATTAATGGAAATAGCATCATAAAAAAAACGGATGGA
CAGAACATCCGTATTTCATTTTTTAATGGCGAGGGTATTCCGGAAGATGTTAACGTTTCC

AACGTTTATGTTGCCGCTTTCACACTTGTCTGCCTGCATCACGGGCATGTAACCATCACT
GATGAGCGAGGGCGAACAATACATTGTGTTGCACCGGGGTTGATGGTGCTTGAACGAAAT
CAGTTGATAAATATCACGATGCAGGAAGTGGATGGTCATCTTTCCTTTGATGTACTGGAT
ATCCCTCATGAGTTATTAACCATCGCACATGAATTGCTTATTGAAAAACTCCAGACAGAG
CATCAGCCTGGAGAACATCCTTTCAGGATTGTTTACAGCAATGATTTCGCAGCAAGAAAA
GAAGTGTTTGATTTGCTTAGATTTACATTGTCGCAGGAAAAAAAAGGCATCTTCGAAAAT
GAGGAAAAAGAAAATATAGCGACATATGCTTTATTGCTGTTGCTCTCAGGCTTTATTCAT
GACACCGCTTTTGCAGGTATAATCTCCCGTTCAGCAAAACTGAGCGTGAAATACAAAGTC
TATAATCTGCTTTACACAGAGCCGGATAAACAGTGGAAACTGGATGATGTGTCCTCAGCT
ATTTTTATGAGTGCCTCCACTCTCAAAAGAAAACTGGCTACAGAAGGGACGACATTCAGT
GAGCTTTATATCACCGCGAGAATGAACCTTGCCAGTAAGTTGCTCAGAACCGGTAAATAC
AGCGTTACCAATGTGGCAGTGTTATGTGGCTATGACAGCGTTTCGTATTTCATTGCCTGT
TTTAAGAGGCAGTTTAACATTACCCCCTCCGCCTTTATGAAATCAGTGAATCACTGA
>CS21_lngT LR883052 ~~~lngT~~~CS21 LngT unknown function~~~
ATGCGTATAAAAATTATTATTCCTCTGTTTTTTTTATTTGTCTGCAGAACCGTTTCTGCA
GACTGTTTTGAGCAGGCCGGGTATGACAGTAATATAGACCCCGATTTGTTAAGAGCCATT
GCAAAAGTGGAGTCAAATTTTAATCATCTGGCAATCGGTAAAAATCCGGTAAGAGGATTT
GGTGTGGGACTTATGCAGATAGACTCGCAGAACTTTGCTCATTTGAGAAAGTTTAATATT
TCGCCAGAAATGCTGCTGGATGCCTGCATCAATGTTTATGCCGGTGCCTATTTTCTCCGA
CTGGCCGTAAACAGAATGGGCAATAACTGGGATGCTGTGGGAGCCTACAATGCCGGCTTT
TCCAGAAAGCCACAACAGATAAAACGACGCTATCAGTATGCGTCAAAAGTTCGTCTGCAT
TACCGTGATCTGAAGAAAAAGTAA
>CS21_lngX2 LR883052 ~~~lngX2~~~CS21 LngX2 unknown function
ATGTTTTCAATTCATAAAATACATTCATTAGTAAGTTTAAGTAAAAAACTGCTAATAAAA
TATCGTAAACAAAGGAAGGGAACCGGAAAGATTGGGTTAGGTTTTTTACTGGGAGTAATA
TATGTCATGGTGCTCTCCTGGTTCCTTCCTCTTGTTTATGATGAATATATATTATGGTTC
CATGATCTTTTCAGATTGGTTGAATCAGTTGTCAGTAGATAA
>CS21_lngA LR883052 ~~~lngA~~~CS21 LngA major subunit~~~
ATGCTATCCGTGTATAACCGGACACAGAAATTTAAAGAAGAGGCAAGAAAAAAAATCGCC
AAATACCATGAATTACGTAAACAACGTGGTATGAGCCTGCTGGAAGTTATCATTGTTCTT
GGCATTATCGGTACGATTGCAGCCGGTGTCGTGATTCTGGCTCAGCGTGCGTTTGATTCA
CGTGCTGTGACTGATTTAGTAACTAATACAAATACAGTCCGCGTAGCAATGAAAGATGCT
TATCAACGTGATGGTAAATATCCAGATTTTGTGGACCCATTAAGCCTTACTGCAAATACA
ATTAAAACTGATACAAACGGAATACCTGCAGCACAGTTAGTTCAGCTTGGGAAAATTACA
CCAGACGAAGTGCGTAATAACATTTCTGGCGACTTTATCGCTATTGGCGGTGCTTTAACT
TCGAATGGTGCTCAAGTTAAAAAAGGTTTTGCTATCGAACTTAATGGATTAAGCCAAGAG
CAGTGCCGTTCTATTCTTGGGCAAGTTGGGAATAACTGGGAATATGTTGCTATTGGTACT
TCTGCGTCTGGTTCATATGCCATGACAGCAACTGGTGTAGATATGTCTGTGGCCGCCTCT
ACAACTGTTTTACGCTCTTTAGGTAACAATGGACAAACAACCTTGACTGCAGACAAAATT
CTAAGTACCTGTACTGCTCAGGTAAACTCAATTACTTTAGGTAGCCGTTAA
>CS21_lngB LR883052 ~~~lngB~~~CS21 LngB minor subunit~~~
ATGAAAATGAGAGGCTTCACACTTCTGGAGATGATTATCACTCTCGCTATAATGGGAGTG
GGGATGATATCAGTTATTAAATATAAAGAAAAAGAAGCTGATGAGGCACGTCGACAAATT
ATATCAGATGCATTGATTTCAGAAGTGGCAGGAATTGTGGATTTTGTTGCTGAAGAACAG
ATATCAGTCGTTGAAAACAACAAAGAAAAAGAAATTACAAATCCACTTTATGATCAGACA
GCTGGAGTTCCTTACATAAATCGAACTAATAATAAAGATTTAAATGCAGCCATGTCTGCT
AATGCCACTGAATTAATCGACTGGAGCGCAGGAAACAATACAAGAATCCATTTTACAAGA
AAATATTGTATAAGTACGGGAACACAGGGAAAGTATGAATTCAGTAAGGATTATATTCCA
TGCAATGAACCCGCAGTTCTGGCAAACAGTGATTTAAAAATAGAACGTATAGATTTTGTG
GGTGCTGATGCAACAGTTGCCAGTGCGATAGAGCGCGTGGACTTTATTCTTGGATTTGAA
AAGGTGAGTCCCGAAGGAGCATTTCACTTTGCAAACTATTTAAACGCTTTAGAGAAGGCT
GCTGAGAAACATTCTATATCATTTAAAGATATTTATGTGGTTGAAAAAGGTCAGTCTGGT
GTTTCAAGTTGGGCACTTACTAAAGTCTCGGGACAGGCTTTAACTATATCTGATTTGTCT
AAAAATATAGGTTCATTAAGCAAGAATAAGAACTATGGTTTACGTATAAGTATCGATCCA
AATACTGGTAAATATTTACGTGCTGACGGGCGTGTCGGAGCCGATAAACTTTGCTGGAAC
ATCGATAGTAAGATGTCCGGACCATGTCTGACTGCTGCTAACGATGGTAATAATCTGGTT
TTAACCAAAGGAAAAGCATCATCAAATAATGAACCTGGTTTATGCTGGGATTTGAATACA

GGTACGAGTAAACTATGTTTGACACAAGTCGAAGGTAAAGATGAAAATGATAAGGATGCC
TCTCTTATTCAATTAAAAGATGATAATGGGAATCCAGCGACAATGCTCGCGAATGTTTTG
GTTGAAGAGAAGTCAATAACGGATCCAACGAAAAAAGTATACCGTACAATACCAAACACT
ACATATGCTTCTTTTGGAAACAGCACTGACAGTGATATTGTCATTAATAACCCTGATACG
TATAAGGGTAACGTAACAACTGAAAAGGGCCGAATTGAACTGAATGTGCAAGAGTGTCCA
GTATCTCCAAATGGACAGAAATTATATCCAAGACTCAGTGCTTCAATTGCTTCTGTTGTA
GCTGATACAAAAGATAATACAGGAAATTATCAGGCAGATTTTAGTCATCTTAGCACAAAC
AGAAATAGTGGAGGACAGCTGGGATATTGGTCTGGTTCGGCTATCCAGGTTAACCAGTCT
GGAGGAAAATGGTACATCACTGCAACAATGGGCGTATTCGACCCAGTAAATAACACAACA
TCGGTGTACCTGAACCCTAAATTTCTTTCAGTAAATATCACAACCTGGTGCAGTACAGAA
CCACAAACCTAA
>CS21_lngC LR883052 ~~~lngC~~~CS21 LngC outer membrane protein~~~
ATGAGAGCAAAATGGGGTGTTTTTTTCTTCCTTAGTATTCTTTCTTCGAGTGCACTGGCA
TCTCCTGTAGCAAGCACTGATAAACCCAGGAGTCCATTCAGTGTTGATCGTCAACCAGCA
GCCGATAGTGTGGAAGAACGAGCTAATATTAGTACCAATAGAATTTTATATTCGCTTACT
CTTAATGAAAGCTCGCTTCTCAGTCAGGAAGTAAAAAACTGGGCTCAGAAGCAGGGTTAT
AAGCTTCTGTGGAAAAGTGATAAAGATTATATTATATATAAAAATGTAAACTTAAGCGGA
CAGTCCAATGAAGATATTTTAAGGTCTTTGGGGGAGCTGTTTTCTTCAGAACAATATGGG
TTAGTTGTTAAATTATACGCCGGAAACAACGTACTGGTTATCGACTCTCAATGA
>CS21_lngD LR883052 ~~~lngD~~~CS21 LngD outer membrane protein~~~
ATGAAGTTAAAAATATCCATGATGTTAGTTTTATTGGCATTGCAGGGATGTTCATCATTT
GATGAAAAGTTTTCAATTGAGAAAAGTCTGTCGCCAGAAGTTGGTTTGATCGAGGCTGAG
GATGTAAAATCTTCTCCATTTATTGTCTACAAGACCCCATATCTTGGAGAAAAAGTAGCT
TACAGTGAAAAAAAGAAAAGACTACTTGAGCAGAATATCTCAATTACCAGTTACGATTCT
ATGACACTGGATGCTGTAATGGATACAGTGGCATCACAGGCGGGGCTCTCATACAGAATC
AAAGAGACATATCCAGGACAGAATAAAGATGATTTCGTCGAAGAATTTCATAATGTCAAT
TTTCGCGGAACAGTAGATGAATTCATTCGGTACATCTCAGCATTGTATGATGTTAGTGTA
AAAGTGGATGAAAACAACATTCTTAATATCTCATATTATGATAATTATGCAATAAAGCTT
GATTATTATGGCGAGAATAATAAATTCGAAACCAGTATTGATCTTTCAGGTAACGAAGCA
ACCACTTCTGGAGGGGTGAAGGGTAAATCTGAGCTGAAGTTTGAATCAACTTTCTGGTCA
GATGTTGAAAAACTTGCAGAACAGTATGTCAGCAGTAAAAATTATAATATCATCAAAGAT
GCATCAGTTCTGGCATTTATTGGAAGACCTTCTGAGTATCGAATGCTCGATAATGTGCTG
AAAAAGTATCGTGAAGATAACAGCCGACAGTTTGTTTTAACGTATAAGATTTTTACTATT
GACAAGAAAAAAGTCCGTAAACTGGGAGCTGCTGCAAATTTGAGTTATTTGAACGGCGGA
ACCAGCATTGGTATTGATACGAGCTTATTGTCTTCATTAAGTGGGGCAACCTTTGGTCGT
GATTTTATCGTTCCTGTAGAAAATACACAAAATTTCAACATTACCGGAAAGATTGAGGCA
ATTTATCAACTGACAGGTAAAAATGTGTTACAAAGTGGCACTTTTATTACCAGAAATAAT
GTTCCTGTGCCAGTGAACCTGACTCAGACTCAAAACTATATCTCAGGAAGGACGCAGACT
ACAAACTCATTAACATCTGTTTCTGACACGACAATTGAAACAGATAAAGTTATTACGGGG
TCCAGTTTTATTCTGACACCGCGTGTACTTTCAGATGGTTCAATTGAGGTCAGTAGCGCA
TTTACTAAGAAGACGCTGAATTCTATAGAGAATTTCGAATCGGTGCAGTTACCAAATGTC
AGCACAACAGAAATGTTTAATACATCTGTACTTAAACCCGGTACTCTGATGATGGTTGCG
AAATATGAAGGTGGCGAAGAAAATGACTCTCGCGGAGCCATGATCTTTGGTGGCGAATAT
AATCGCGAAAACAGTGATAATACCATCGCAATGGTTGTAGGTATTGACTATTATCGGGCA
CCTGTTATGGCACCATAA
>CS21_lngE LR883052 ~~~lngE~~~CS21 LngE integral membrane protein~~~
ATGAATCTTGAACAGATTTCAGATGAAAAAATTGCGCTGGAGGTTTTATGGATATCCTCA
GAGAACGTATCAAAAAAGATCACAGACACAAGGGAAAGAAGTTGGCGAAGATTCGTAGA
C
AGACATTACAGGAAAATTGAATATACAAATTCAGAAAAAAAACTATGTCTTGGATACAGT
CCTGATACATCTTCCCAGGCTCAACCTTTTGCTCTTTATATTCGCAATATTTTTGGAGAT
GGCATTTACTATACAAATGAAGAAAGTGATAAAAATTACTTATTAGTTATAAACAACGGT
GAAGTCATGGAAGGAACAGATGCCTATCTTAACAATGCGTTGTTTGAGAGGTATAGGAAA
CAATTACAATGTGAAGAATACGCATCTCTACAATGGAACTGTCTGACAATTACACATATA
GATGAGGTAATTGAAGCAAATAATTTGTATAAGAGAAAAAATAAAAAAAAGAAAATCACA
TATCTTTCAGTTATGTTAGGCGCAGGAGTGATTTTTCTTTTATTATTTTCCTTTACGCTT
AAGATGTTCTTGGTGAACTGA

>CS21_lngF LR883052 ~~~lngF~~~CS21 LngF integral membrane protein~~~
ATGAATTTATCTAATTTTGACTGGAAAACCTATCTTGCAAAAATACAGAGACAGTCATAC
CTTGCTATTGGGGGGGCAATATTTGTATTCTTTTGTATTTGTGCAGGAGGCGTTTGGTAC
TTTTATGAACAAAAGGAGTCTGCTAAGGAAGTTGAGAAGAAGCGGCAAGAGCAGGAACT
G
GCAAATAAAATAAAAAGTATTAATGATTATTATGCTGACATTTTAAGTGGTTCTTCAACA
ACTAAAGCAATAGATATTTTTTTGGCTATTAACCAGAGTAGTGTACCTCTCTCATTATCT
GGTTTTAATCTTGATTTATATTCTTGTGATGTGAACAGTTGTACTTTTAGCTACAGCACA
ACAAATGAGAAAATATTCAATATCCAGGAAGTTAATTTTTTAGGTGAATACTATAAAGCT
AATATTTCAGAGAAAGGGCTAGAATATCAGATAAGTCAATCATCACTGGCAGATAATGAT
TTACGAGAAAAATATAATTCCATGGAAGATATCCCTGTTGCTGATTGTAGTGAGTTGGTC
AACTATGTACATTCCTTTAACTCTCTTACATCCGATAGTAAAGGAAAAATTGTACTTTCC
GGTTATCCCGACTCAAGCATCAGTTCTGTAGAAGATGTGCTTCCAGAGATAAAAAAGAAA
TATGGGTTTAAAAATGTACAATGGAGTACAACATTACCTAATGATATTCTTAGTGTAACA
TCTTTCCTGAGTCGTCAGGCTTATAAAGAGTCTTTTCGTGTAAATAAAATTGAAAAAAAA
CAGTCTTCCGAAATTGAAATTTCAGGGAATTTATTATGTGCAATTTAA
>CS21_lngG LR883052 ~~~lngG~~~CS21 LngG outer membrane protein~~~
ATGTGCAATTTAAAGTTATGCTCAGTATTTTGTTTTGCAATTTCAGGTGTAATAATTTGC
AGTAATGCATCGGCCCAGGAGTCGCAGGATAAGTCCGAAAAGGTAACTCCGGCTCAGGAA
CAGATTGTGCGTATGCTTGAAGAAGATTTAAATTTTGAATCTCAGCGGAAGCAATTGAGC
AATGAACTGGCACTGGAAAAGTTACGTCTTGAATTAAATAAACTCAAGGCAGAAAATCAG
CCTGTAGTTCAGTTTACACCATCTCCCGCTGATGAAAAGAAGAGTGAGTTATCATCAGTA
AAGCTGGCCTCACCTCCGGCTATTGTACTTGTGTCCGAAGTGGCTGGTTTGTCCCGAATC
CTCGTAAAGGACGGGGAAAGTATAAAATTGCGTAAGCCCTCAGAGATTTTTACCGCGATT
AATGGAAACAAATACAGATTAGTTTCTCTGGGTAATCAGAAATACACGCTGAAGGATGTG
CAATAG
>CS21_lngH5 LR883052 ~~~lngH5~~~CS21 LngH ATPase~~~
GTGGGTAATATTTTACTCAGAGAACTGAGCAGCCATACTTACAGAAATGAACAGTGGCAT
TTCTTTATTTTAGAGGAGGACCTCCTTTTAGGAAAAAATAATGAAAATTATTATTTAATA
CAAAAAAATACAGATGAGATACTTCCCGGGAAAGTACAGGCTGACCTTACATACCTGAAT
CTTGTACATAAAGTTAAAATGACGGGCCTTGCAGTCGTTGATTCTGATTTATACTATCAG
CTTCTGGAAGATATCTCATTAGAGAATGTAAAGCGTCGTGACATTAATTCCAGTTCAATT
CAGCAGGAACTGGAAGCACTTTTGCTACATGCTGTTAATCGCGGAGCCAGTGACCTCCAT
ATTACTCGTGGTGATGTTATGGCCAAAATAGAACTACGTATAAATGGAGTCCTTGTTCTC
GATCATCAGTTATTATCATCAAAATGCGATGAACTAATATTTGTATTGTATAACGTTCAG
GCTTCAACGCGTGATACAACCTGGAACAGAGCAATTGCGCAGAGTGCAAATATATTATAT
ACATTAAACGAACGAAGTTATCGTTTCAGATATGAGCATTATCCTATCTTTGGTGAAGGT
GATGGATGTTATCATTGTGTTCTGCGTATTATCCCTTCAGGGCTATCTACAGTGATAAAT
CCTGATCTTGCTAAGTTAGGTGTATCTGATGCCGAAGTAAGAGATATACAAAGAATACTA
AGTAATCCTTACGGTGTTTATTTCATAGCAGGAACAACTGGTTCAGGAAAATCAACGACG
TTGAAAAACATGATGGAATGGATGCAGATTAATCGCTACGATGATAAAGGGTGTTTTTA
ACAGTTGAAGATCCTGTTGAATATCAAATTTATGGCGCTAAACAAAGCTCTGTTCTGGAT
GGAGAAAATGGAGGTTTTCACTCAGCAATAAAATCATCTTTGCGGCGTGATCCTGATGTT
CTGATGGTTGGTGAGATCCGTGATCCCGTATCATCAAATGCTCTTGCCGGAGCCGTGGAA
AGTGGACACTATTGTTTCACAACTGTGCATGCAGGAAATATTGTTACGTTATTGCAGCGT
CTTTCAGCTTTAGGTATCAGTAGTGATAAATTATCAACGCCTGGATTTATTGCTGGTTTG
CAATGTCAAAAATTAATTCCAGTTTTATGTAATAATTGCAAACTTGATTGTGAAACGGAA
ATTCTCGGACGAAAATTCATATTAAGTAAAGTGAATGAGCAGGGATGTGATGATTGTAAT
TTTACAGGAGTGAAATCACGACAACTGGTAATGGAATATTTGATTCCTACTCATCGTGAG
CTGGAAGCATTAGCAAATCAACAATGGCTTAATGTGTACTCTATATGGCGGGAGAAACGT
TTTACATCGGAAGGGGTTTCTGAAGGATTTGCTATAAAGGAAAAAGTGTTTATGCATGTG
TTGAAGAAGCGTGCATGTTATCACTGGTTCATGATGGAATTTGGGGCAATTCCGGATACA
GATATGGAGGTATTACTTGAAAAAATTCAATAA
>CS21_lngI LR883052 ~~~lngI~~~CS21 LngI integral membrane protein~~~
TTGAAAAAATTCAATAAAAAACAACGTATTTATCTCTATCAGTTTTGTGCAGATATGTTG
CATTCAGGATTACCGATCTATGACTCTATTGTAAAATTGCGATCTGAAGGTGAAGCATTA
CTGGGGAAATCGTTTGCAAAACGTCTTGATTATTTAATGGAACGTATGAAAAACAGTCCC

TCAGTATCTGCATCCTTTGACACCCTGATTCCGACAGAAGAGTTAAGTGCAATTACTGCT
GCGGAGAATAGTGGTAGCCTGGCTGAAGGTTTTAACAGTATGGTGTTGACCATAAATTAT
CAGGAAAAACTAAAAAGCCAGCTAATAAAATCTGTTACTTTCCCAGCAATAATGATGGTG
CTGGCTCTAATTGTTATAGCCGGTTATGCTGTAAAAGTATTTCCTGCTTTCGAAAAGGTT
GTTGCTGTTTCCCGATGGCCAGGAGTAACTCAAAGTCTTTACTATTTTGGCACGGCTTTA
TATAATGGGTTATGGATCACAATCCTTATCTCTTTTGTTGTATCTGTATTTACAATTCGT
TTCATCATGTTTAATTTTCATGGAGAGTTAAGGGATAAATTTCTTGACCGAATCGTGCCT
TTTTCTATCTATAAAAAACTTGTGGCATCAGTACTGATTAATGATTTGTCATTAATGATT
AAAAACAGGATTCCTCTGGCGAATTGCCTCATTATTATTGAGAAAAATGCGAATCGATGG
CTTAAATCTCATATCAGAAAAATGCAGGATAATATGGCCAAAGGTCTGGGATATGGTGAT
GCATTTAAAACAGGACTTCTTGGTGGTGATGAATTACTGAATATCAGTCTTTATGCCAGT
TTGCCATCTTTTGATCAGGTTCTTGGAACGGTATCTGATAAGGCAAAAATTAAAATTGAG
CAGAACATTAACGTCCTTGCAGGAATGTTAAAATCCCTGTCCACTTTAGTTCTGGGGGGG
TGTGTCGTATGGGTTTTTATCGCGCTGTTTGCGCTTTCTGACGAACTGTCAAAAATGACT
GGTTGA
>CS21_lngJ LR883052 ~~~lngJ~~~CS21 LngJ secreted protein~~~
ATGAAAACTAGATTCAAATATGGACTATTAGCTGCAGCTATATTCTATTCACTACCGGGG
ATGGCATCTACAACCTCCTCTGAAGGAGGTGCATTTACAGTTAAGATGGCAAAATCTTCT
ACTGTAGATGATATCAAGGGATGCCCGACACTGGAAACCCCGCTGAAGCTGACTTTTACC
GAAGATATGATTCCAATTAAAGCAGAGAATTCTAGCATCTATTCTTATTATGATGGGTGG
CTTGGTGTCGGTGTCGGTAATAATAGAGACACCCCGTATCCATGGCAGGATCTATTAGGT
AATACCCGATATACAGCTAAAAATCATGAAATCCATATTTATGTAGAGTTTTTTAAGGAG
CCTGTAAACCGTTTTTCTGATAAAGGGGGTATTTTTACATATACAGATCCAAACGGAATA
ATGTATTCTAATGGCGAATATCCTTGGCAGCAGGTACCTGAATTAGGAAAGAATGTTTAT
AAAGCTGTTATCACTCAATGGAATAAAGGTGAAACCAAGAATATTTATTTGCCTGGCCGT
GATTTTAAAAATGTAGAGGTTTTTTACTTCCAGGATAATGTTCCTTACTGGGATGACAGA
AACAATTATAAAAAAATGAAAGAAAGGCTTTCGAAGCTGAAATCAGAATATACTCAATCT
GAAATTCAAACAAAGTTGCCGTCCCAAGAAGAGTCTACCACTGGTGATTACGCACCTAAA
CCTGGTGACACTGTTCGCACGGCAGAGGCTCTATATCTGTATCAAAAACTAACTCGAGCC
AGTGTGAATGAGTCGAAAATTAACTTCGAACAGATAAGAGGACAGTTTAACAATTTAAGA
AATACCTGGTGGACACAAGAACTAATTTTATTTGGAGGTCAAGGCAAAGAAAGTTTACGA
AATAAAATAGAACTTAATCAGAATAATTACGTGATGAAAGATACAGGTGTTTTTGATAAG
GCTTTAAAAATTGAAAGTATTGATTTGAAACTGATGGAGAATAAAAGAGTTGGCTCGAGA
TTTAGTGGGCCTAATGGACTTAAAGGCACTTATATTGCGAGTTATGACAGAACGGATTTT
TCTATGACACCAGAAAATATCAAAGCTTGCGGTCTTGATTAA
>CS21_lngP LR883052 ~~~lngP~~~CS21 LngP prepilin peptidase~~~
ATGTATGTTGAAATCGGCGTTTTCTTTTTTTTATTCATTACAGGTTTGTGCATTGGTAGT
TTTTTAAATGTGGTCATCTATCGCATTCCTTTGTCGCTCATAAAGCCTCAGGACGATATT
TTTAATATAGCATGGCCACCTTCACATTGTACTAACTGTAAAAATAAAATTCTCAAAAGA
GACAATATACCTTTATTAAGCTGGTTATTATTGAAAGGTCAATGCCGATTTTGTGGTGGC
GTGATATCTTCACGCTACCCAATTATAGAATTAATCACAGGTCTGTGTTTTTCTGTAATT
GGATTATGGATGTTTGATTTCTTAAATCAGCAGCCATTGGTAGTAATTTCTGTTCTGTTT
CTGTTCTCAGTTTTACTTTGTCTGACAGTTATTGATCTTGACCATTTGTTATTGCCTGAT
AGCTTAGTTTTTACTTTATTATGGACAGGGCTACTGGCAGCAACATTTGAAATTTCACCT
GTTCATCTTAAAGATGCCGTTATTGGAGTTTGTGGAACGTGGTTATTTCTTACTGTAATT
ACATATCTTTTTACCGTCATCAGAAATAAAGAAGGCCTCGGTGGTGGTGATGTTAAATTA
ATCAGCGCGCTATCCGCATGGGTTGGCTGGTACTATATTCCAGCGTTGCTAGTCTTTTCA
TCTGTAATTGGTTTTATTTTATTCTTTATTGCAAAAAGAAAAATGGAATTCAGTTCCGAT
ACAGAGAGGACCCTATATTATGTAGTTCCATTCGGTCCAGCTATATCGTTAACTGCATTT
TTCATATATTTATCTAGTATCAACAAAGTGAATTTTATTGATGGTGTTTATGAATACTTT
CTCTTATTTCTATGA
>CS22_cseA AF145205.1 ~~~cseA~~~CS22 CseA major subunit~~~
ATGCGTAGTAAATTATCCATTCTTATTCTTGCTTTGATGACCTGTGGTGCCGCAAATGCC
GCAACTGTAGTAGGTGATGTTGCCACTGTTCAGGCTCCAGTAGTATTTAGTGCACAGAAT
ACCATCAACGCAACCTGGACTCAGGATCCCTCTGTATCTGGTTCTTCAGTTCAGGCAATG
CAAAAACTGGGTACTCTTAATATTCAATTAACCGGTTCTCATGCAGGGGTCTATGTTTCA
GGAGATGGTACGGGGGTAAGTGGCGGCCTCGTTACTATCCCATTTAAAAATGCTGCAGGT

CAGATTCCGTTCCGTGGACGTACTAATGCCGATATTGGACAAGCGTCCAACACGCTCATA
GCAGGCCACAGTGGACCGGGTTGGAATTTGCCAGACGCTGGGAATAATATTAGTCTTGAT
ATTAAAGCCTTCCAAAAAAATGATAATATCCCTGCTGGAACATATACAGCAACATTCTAC
ATTCAACAATATCAGAGCTAA
>CS23_aalR JQ434477 ~~~aalR~~~CS23 AalR regulator~~~
ATGGGAAAATTCGCGGGTGTCTTAATGGAAAATAAAAACAAATCCCTGAATAAAGGGGCA
AAGAACAAAATGCTAATTCCAGGGTTTGTATCTGAAGTTGCTTTTGATTTATTAATTGAA
CTCTCTTCTGTGCACAGTATTAAAATTATTAATGCTCTTCATGATTATCTCGTTTTAGGA
GATAGCAGGAAAATTGCTTGTGAACGATATGGTGCAAACAGTAGTTATTTTAGTATTGCG
CTGGGCCGTCTTTTTCGGACGAACCAGATAGTATCACAGCTCGCTTCATATTATGGGGAG
CAGGAAAGGTTTTTCTAA
>CS23_aalA JQ434477 ~~~aalA~~~CS23 AalA tip subunit~~~
ATGCGAACTTTCTATTTCTCTCTGTTCCTTCTGGTCGGGGGGGGGGTCAACGCTTCTGTT
CAGAAAACCGTATTCAGTGCTGACGTGGTGGCTTCGGCCTGTCATGTGGTGGTGGATGCG
GACAGTATCGGCAACAGTGGGCGGCTGACATTCGGGACTTACCGCAAATCCACGGGGGCG
TCTGTGCCTCCGCGTGACTTCACTGTGCGTCTGTATGAGTCCGGTGCCACAGTTCAGGGC
TGCTCAGCCTTTAAGGCAGGGCAGGTCGCCACCCTGGATTTTGGTAACCCGGGCCAGCTG
GATGCAGGGGGGGTGGTCACCCGCGGTGCCGGTGATGGTATTCGCGTGGATGTGCGGGCG
GTGGATACACAGGCTGATTATCGCGGACGTCTGACTCAGGACAGTCATTCGGTGAATTAC
CCGGTGGAGTTTGCTGCTAAGGGACAGTTCCGTTTTCGTGCGCAGCCGGTGTTTCCGTCG
AACGTGAAGGCGGGGGAATACACGGGTGCACTGACCTTTGTGGTCACGTATCAGTAA
>CS23_aalB JQ434477 ~~~aalB~~~CS23 AalB outer membrane usher~~~
ATGTCCTTTATCCAGGGTGGTGCCGGGGTTAATCCGGAAGTCTGGGCAGCCCTGAACGGC
AGCTATGCGCCGGGGCGTTATCTGGTTGACCTGTCCCTGAACGGGAAGGAGGCCGGGAAA
CAGATACTGGATGTGACACCGCAGGACAGTAATGAACTGTGTCTGACAGAGGCATGGCTG
ACGAAGGCCGGGATTTACGTCAGTGCAGATTACTTTCGTGAGGGATATGACGCCACACGA
CAGTGCTATGTGCTGACAAAAGCCCCGTCAGTGAAGGTGGATTTTGATGTTTCCACCCAG
AGTCTGGCGCTGTCCATTCCCCAGAAGGGGCTGGTGAAGATGCCGGAGAATGTGGACTGG
GATTACGGGACCAGTGCATTTCGCGTGAACTATAACGCGAACGCCAACACCGGTCGTAAT
AACACCTCAGCCTTTGGCTCAGCGGACCTGAAAGCCAATATCGGGCGCTGGGTGGTGAGC
TCTTCTGCCACGGCCAGCGGCGGTGACAGCGGGGATAACTCCACCACGATAAACATGTTT
ACGGCCACCCGGGCCATCCGTGCACTGAGTGCGGACCTGGCGGTCGGGAAAACATCCAC
C
GGGGACAGTCTGCTGGGCAGTACGGGAACGTACGGCGTGTCGCTGAGCCGGAACAACAG
C
ATGAAGCCGGGCAATCTGGGGTATACCCCGGTGTTCAGCGGCATTGCGAACGGGCCATCG
AGGGTGACGCTGACACAGAACGGGCGGTTGCTGCATTCGGAGATGGTGCCGGCGGGTCC
G
TTCTCCATCACGGATGTGCCGCTGTACACCAGTGGTGATGTGACCATGAAAATCACCGGT
GAAGACGGGCGCGAGCAGGTACAGAACTTCCCGTTGTCGGTGATGGCCGGGCAGTTAAG
T
CCGGGGCAGCACGAGTTCAGTGTGGCAGCCGGTTTGCCTGACGATGACAGTGACCTGAA
A
GGCGGTGTATTTGCGGTGTCATACGGTTACGGTCTGGACGGGCTGACGCTGCGCACCGGC
GGGGTGTTTAACCAGGACTGGCAGGGCGTCAGCGCCGGGGCTGTTCTGGGGCTGAGTTAC
CTGGGGGCGGTGTCTGCTGACGGGGCTTATGCCACGGCGAAATACCGTGATGGCAGCCGC
AGCGGAAATAAGGTGCAGTTATCCTGGGGTAAACAACTTGAGATGACGAACACCGGACTG
CGGGTGAGCTGGTCACGGCAGAGTGAGGAATATGAGGATATGTCCTCCTTTGATCCGACA
GAGTTGTGGTCGCAGTCAAATCATGGTCGCCGGACGAAGGATGAATGGAATGCCGGTGTC
AGTCAGTCGGTGGGTGGTCTGTTCAGTCTGTCGGTGTCCGGCTGGCAGCGGAGTTATTAC
CCCGCATCGATGACCGGGCGTTACCGGTACAGCGATGACAACGGTAAGGACACGGGGATT
ACCGGCACCCTGAGCACACAGATTAAGGGTGTCAGTCTTAACCTGGGCTGGTCCGGTTCA
CGGAACACCCGGGGGGGAAACAACTGGTCTGCATCGGCGTCAGTATCGGTACCGTTCACA
CTGTTTGACCGTCGCTACAGCAGCAGTACGTCGGTGAGTACGAGTAAAGACGGTGGCACA
GGCTTCAGCACGGGTGTGTCCGGCTCGCTGAATGACCGTTTCAGTTATGGTCTGGGCGGT
GGGCGTGACAGCGGTGGTGGTGTGAGCAGTTATCTGAATGCGTCGTACAGTGGTGACCGG
GCTTATCTGAATGGTGCCCTGAACCACTCGCAGTCCGGCGGAACCAGTGGTTCTGTTTCG

GTCAGCGGTTCGGTGCTGGCCGTTCCGGCGGCGAAAGACATCATGTTCAGCCGCACGACC
GGGGATACCGTGGCCGTGGTGAACGTGAAGGATACCCCCGGAGTGAAGGTGACGTCCGG
T
GACGGACAGACTGACAGCGACGGCAACCTGGTGGTACCGCTGAACAGCTATGACTGGAA
C
ACGGTGACGATTGATGCGGGCACGCTGCCGCTGAGCACAGAACTGACGAACACCAGTCA
G
AAGGTGGTGCCGACGGACAAAGCGGTGGTGTGGATGCCGTTTGATGCCCTGAAAGTTAA
G
CGTTACCTGCTGCAGGTGAAGCAGCGTGACGGTGAGTTTGTGCCGGGGGGAACCTGGGC
A
CGTGACAGCAAGAACACACCGCTGGGCTTTGTGGCCAACAATGGTGTGCTGATGATTAAC
ACGGTGGATGTGCCGGGGGATATCACCCTGGGCCAGTGCCGGATACCTGCGGCGAAACTG
CAGGATACTGAGAAACTACAGGAGATCACGTGTGAGTAA
>CS23_aalC JQ434477 ~~~aalC~~~CS23 AalC chaperone~~~
ATGTGCCGGGGGATATCACCCTGGGCCAGTGCCGGATACCTGCGGCGAAACTGCAGGATA
CTGAGAAACTACAGGAGATCACGTGTGAGTAAGCGTAACGCAGTAACGACGTTTTTCACT
AACCGGGTGACAAAAGCACTGGGAATGACTCTGGCACTGATGATGACCTGTCAGAGTGC
G
ATGGCTTCCCTGGCGGCAGACCAGACCCGCTATATCTTTCGCGGGGACAAGGATGCGCTG
ACCATCACGGTCACCAACAATGACAAGGAGCGTACCTTTGGTGGTCAGGCCTGGGTGGAC
AATATCGTGGAGAAGGACACCCGTCCGACTTTTGTGGTGACACCGTCCTTCTTCAAGGTG
AAACCGAATGGTCAGCAGACACTGCGTATCATCATGGCCTCGGACCATCTGCCGAAGGAT
AAAGAGTCGGTGTACTGGCTGAACCTGCAGGATATTCCGCCGGCTCTGGAGGGCAGCGGT
ATTGCGGTGGCGCTGCGCACGAAGCTGAAACTGTTCTACCGACCGAAGGCACTGCTTGAA
GGCCGCAAGGGGGCAGAGGAAGGGCTCAGCCTGCAGAGCCGCCCGGATGGCAGGACCAT
G
CTTGTGAACACCACGCCGTACATTTTTGCGATTGGTAGCCTGCTGGACGGAAACGGGAAG
AAAATTGCCACGGATAACGACACGGCACAGAAACTGCTGATGTTCATGCCGGGTGATGAA
GTGCAGGTGAAGGGAAATGTGGTGAAAGTGGATTCTCTGAATGATTACGGTGAACTGCAG
ACCTGGACGATTAACCAGAAAAAAACGCCAACGTCTTCCGGGCAGAAGGCATCTGATTCG
CCGGTTAATCCGTCTGATAAGGCAGATAAAAAATAA
>CS23_aalD JQ434477 ~~~aalD~~~CS23 AalD minor subunit~~
ATGAAGAAAACAATGATGGCAGCCACCCTGGTCCTGAGTGCACTCAGTATTCAGTCAGCA
CTGGCCGCTGAATACAGCGAGAAAACGCAGTATCTGGGTGTGGTGAATGGTCAGGTGTCA
GGTAACAGTGTGGTGAAGGTGACACGTACACCGACAGACCCGGTGCTGTACCGCTCAGA
C
AGTAACAGCCCGTTGCCTGCAGAGCTGATAATCCGGCATGCAGAAAGCCGCCCGGCTTCC
GGCGGACTGGCAAACATCACGGTGAAGCAGTCTCTGCCGGATAACGGGGAAGCCCGCAT
C
ACCCTGAAGACGGCCCTGATGGTTGACGGAAAGAGAGTGGCACTCAGTGCCAGACAGCA
G
GGTGAGGATGTGGTGATAACCGTTCCGGAGGCACAGAAGCTGGTTGAGTTAAGAACGGAT
GCACCGGCAGAGCTGGAAGTCCCGGTCAGTTACCGGGGAAATCTGCAGATAGCGCTGCA
G
GTGGAAGACTGA
>CS23_aalE JQ434477 ~~~aalE~~~CS23 AalE major subunit~~~
ATGAAATATCAAGGAATTTATTTTATGAAAAAGACTCTGATTGCACTGGCTGTGGCTGCC
TCTGCTGCAGTATCTGGTTCTGCTATGGCGTGGACTGTAAACGGCGATGGTGGTTCTGTT
AGCCTGAGCGGTGCCGTGAATGTGCTAGCAGCAGACACCCCATGGGAAGTTCAAACAGG
C
GATGCGGTAAGTGGCCTGGATGCTACTATCCACACAGGAGATAGGAAAGTCGAAATCCCT
GTGAAGAATACAATCCCTGTTCTGGCTATTCGTACTACGACCGATACCCCCTTCAAAGGA
CAGAATGGTATCAGTCCGCAGATTGATTTCAATGGTGCTATTGATATCAATTCATTTAAC
CAAGGTAAGACAAATCTAACTCTGGAGGTGAAAGATACTTCCGATAAGAAAATTGGCACT
CTAACCACTATCTTAAGTACTGGAGCTGAAGCGTCTGTGTCAAATGGAACGATCAGTTCA
AAGTATAATGTCTTTGCTGCCAATCCAGGTAATGCTTTTTATGGAGGGGTAGGTAAATCG

AATGATAAGATTTACGATAAGGGTTGGTTTTAGCTAATCTTATCAGTACGGATAATGTC
AAAAACTATAAAGACCAAAGTGGGAGTTATAAACCTGAAGGTAGTATTGTGAACTTCGAG
AACCCCCTATTCACCTATTCAGCTTTTTATGCTGCTGGTATTCTGAATAATGCAGTTATC
TCAATCGTACTTGATGATCCTGCTAAATCAAATATTACATGGAAAGCATCCCTGCCGGTT
AGTGTTACTTACCAGTAA
>CS23_aalF JQ434477 ~~~aalF~~~CS23 AalF minor subunit~~~
ATGAAAATAGCGCATCATTATAAATCCCTTCTTTCAGCCATTATTTCGGTGGCCCTGTTT
TATTCGGCAGCGCCACATGCAGATATTCTTGATGGTGGAGAAATTCAGTTTAATGGTTTT
GTCACTGACGAAGCCCCCAAATGGACCTGGCAGATAAGTTCACCGGACCAGACCTGGGC
A
GTGGATACTGCTGATGCGCGTACAGATAACGGACAACTGGTTTTTAATTTGCGTGATAAA
GGGGCTCTGCCGTTTCTGGAGGGGCATCTGCATGAAGTGGCTGAGCGAGGTGGTCCCGGA
TTCACCCCCTTTATTACCTTCAGCAGTAACGGACAGCCTTTTACTGTGACGGAAGGAAAT
GGTACCTCAGCACAGCATTTCCGCGCCTCTGTCCCGGTGCGTGACCCGGAAACGGGAAAT
GTGTCGGGGCAGCTTTCTTTCACCCTGAATCAGGGGATGGCAGTCAGTGCCGGTGTACAG
GAAGAGGGGGCGACAGTGCCGGCAGGGATGTCGCTGGTCGGCGGACAGAGCGTGACGG
AT
GTACAGTCAGGAACGTTGCCACAGTGGCTTAAAACCCGCCTTTCCACATTACTTCTGATG
AACCAGGGCTTTGGCAATGGCATGAGTGCCGCAGACAATGGTCAGGTCATCTCTCAGGGG
GTATTGGCTGACGGTCGGGTGATGAATCTGGCTGCGGCATATGCCTCAGCAGTGTCGGAT
TTTGAGCTGCGGTTACCGGCAGAGGGCACGCCTGCAGCATGGCAGGCAGGGCTGAATGT
G
ACGGTTACAGTACAGTGA
>CS23_aalG JQ434477 ~~~aalG~~~CS23 AalG minor subunit~~~
ATGACCGTTTTACTGGGAAAAACCACCCTGACCACACCGGCAGGACGTGAGGGGCTTTCT
CCCCGTGTCAGTTACGGAAAGGGTACTGAGGGATTTTCGCTTGAATGGACAGCACCAGGC
GTAGCGGAGGTGACGCTGCCTGTCACCGGAGATAATAACGTCCGGGCAGGCACATTTGTT
TTCAGAATGCAGGCTGCGGGGGTGTTGCGCCATGTGAAGGACGGACAGCCGGCGTATGCC
GGTGTGTATGACGACCTTAATGTGAATGGTCTGCCTGGTGAAAGCGCAGCCATGAAGACT
TCTGATATTCCGGGTGTTCTGCAGAAGATGTTCAGTGGCGAAGGCCCCGGCTGGCTGCAG
ACAATGACCATCAGTGGTTATTCGGGAGTGAGTCATTTCAGTGATGCCTCCCTGCGTCAG
GTTGAAGGTGCGTATGGTGCACAGACTGTGGCAGGTAGTGGTGAATTACGTCTGAACGGC
ACGATGCCGGAGCGCTGGCGGGTGTCACTGCCGGTAAGTATTGAGTACCAGTAA
>CS23_aalH JQ434477 ~~~aalH~~~CS23 AalH minor subunit~~~
ATGTTTCCGGCTCTGTTTTTCTGACTTCAGCCAGTGTGCTGGCGCAACCCCTGATTATT
CCGCCGGGCCACTGGCAGGAGGGAATGGCTGTGGGCGTGACGGAGTTCAGCGGCACGCT
G
TATGTCCGCGATACGTCCTGGCAGTGGCAGCCCCGCGCTGTGCGGATGAGTACACCAGAT
GCCGTGCAGGCCGGTCTGACAACGGGTAAAGGTGGTATGGTCAGTGAAAGCCGGAGAGG
G
CAGGATTTTTATATTCTTGGCGGACATACCACATCACTGACAACTGCCCGTCCGGGGCTG
CAGCCGTCGGTAACATTACTTCAGGTGGCGCCATCATCTCCCCGTATTGCGGCCCGGGGA
GAACTCGCCCGGGGACAGGTGCGTTACGGGGAAATCACGTTCACGATGCACCATCTTCTG
GCATGGCAGGACAATATTACTGGCGGTCAGGGCTGGAGTGTGGTCAGCGGAGAGGTGAC
A
CCGGAGGCCGAAAAGCAGGTGAAACGCCAGTTATGGCAGGTGAACGGCTATGAATGGAC
C
CCGGACTATGCCGGGTTAACCGCGCGTCCTGATGCGTTTATTTCGGGCGCTGAGTCGCTG
TTGTCACAGGAGAATGGCAGCCAGCATATTGCCGGTGCCTGGGTGACATCCCTGAGTGAT
GTGCGGGTGAATTTCCCCGGTGCTGAGGAGCAGGTGAAACGCTGGCAGGGTAATCTGAC
A
CCGGTGGTGATGTATTTCTGA
>CS26_crsS E528_7521_1#30 ~~~crsS~~~CS26 CrsS transcription regulator~~~
ATGAAACGTAAGTACCTTTCAGAGAAAGAGGTGCATTGTTTACTTAGAGCGGCTGCGAAA
AACCAATACTCAGTTCGTGACTATTGCATGATAAGCATGGCTTTTATTCATGGATTGAGA
GTGAGTGAATTAGTCAACCTTACAATTGATGATTATGATGCTCTCTCTGGCCAATTAAGC
ATAAAACGGCTAAAAAATGGATTCTGTACTATTCAGCCATTATTACCTGACGAGAATGAA

CTTCTTCAGCGTTGGCTGGATGAACGTGCAACATGGGATGGTCATGAAACCGATTGGTTA
TTTTTGTCTAAAAGTGGCGGACCACTTAGTCGTCAACGCTTTTGGCAAATATTACGTAAG
TATGGAGATGACGCTCATCTGACAGTAAGAGTTCATCCACATATGTTAAGACATGCTTGC
GGATTTAATCTCGCAGAACGAGGCAATGATACAAGGTTAATACAGGACTATTTAGGGCAC
AGGAATATACGGCACACTGTACATTATACAGCCAGCAACCCTGAGCGTTTCAGAAATGCA
TGGACAAAAAATAACTTTCACAGTTTCTCTTGCGCTCAGGATGTAACGTCTTAA
>CS26_crsT E528_7521_1#30 ~~~crsT~~~CS26 CrsT transcription regulator~~~
ATGACTAAGCGAAAATACCTAACCCAGAGTGAGGTGGAACTCATGCTTACAGAGGCAAAA
AAAGGGCAATTTTCAGAACGGAACTATTGCTTACTTTACCTGAGTTTCATTCATGGGTTT
CGCGTGAGCGAAGTATGTAATCTGAGGCTTGAAGATGTGTGTTTGCGCGATAAAAGCTTA
AACATCCGCCGCATAAAGAATGGATTTTGCACAATTCACCCCCTTCTTTCTGATGAAATT
AACGCGCTTAAGGCATGGCTTTGTGTGAGAGACCAGATGGGAGGCGCTGAAAGCGAATG
G
TTGTTTGTTTCACGCCAAGGTGGTCCTTTAAGCCGGCAACGTGTCTGGCAAATGATCCAA
AATCTGGGTAAGAAAGCCGGTATAAAAGTGAACTCCCATCCACACATGTTGAGACATGCA
TGTGGTTACGCCCTTGCCGATCGAGGTGTAGATACTCGTCTCATACAAGATTACCTCGGA
CACAGGAACATACGCCATACCGTACGTTATACCGCAAGCAATGCCGAGCGCTTTAAAAAT
GTCTGGTCAAAAAAAGGTCGAACACTTAACAATTAG
>CS26_crsH E528_7521_1#30 ~~~crsH~~~CS26 CrsH Major subunit~~~
ATGAAAAAGACAATTATGTCTCTGGCTGTGGTTTCAGCTTTAGTAAGCGGGGTTGCATTT
GCTGATCCGCCGAAGAACGATTCAAGTAAGGCAACTGTGAACTTTAATGGTAAAGTTACT
AGCAGCCTTTGCCAAGTGAGAACTGATAACATAAGCCAAGACATTTCTCTGGGTGAGGTT
ACCACCACAGTTCTGCAAGGGTCTGGCAAAGGTAAACCACAGAGTTTCGAGGTTGGTCT
G
ACGAACTGTGATACCGCTACTAACAAGATTTCTTATGTACTTTCTGATGCTAACTACACC
CCGATCGTTAATGGTGGTCAGGCGACCAACCTAAATTATTTGGTACCGAAATCAGGTGAT
AACTCTGCAGTAGGTGTAGGTGTATACGTTGAAACCAGCACTGGTACAGCGATTGCACCA
GGATCAACTACCGAGCTTAAAGTAGAGAAGGACGCAGAGAACAAGGCTCTTTCTAACCA
G
ACAATTTCCCTACGTGCTTACATTGGTACCAAGACTGGGACAGCTGATCCTGCTGCTGCC
TCCCAAGTTGAAGCAGGTACTGTTGAAGCAACTGGTGTACTGACTATTTACGCGATTTAA
>CS26_crsB E528_7521_1#30 ~~~crsB~~~CS26 crsB Chaperone~~~
ATGCTTCGTTTAGCCATTAAGAACTTATTTCTATTATCTTTAATATCAAGCACAGCCATC
GCAGGTTCATTTGGCCCAAGAGAGAATAGACTAATTTTTGAGGGCGGCAAAACTTATGTG
CAGTATCGTATTGACAATACGGATAAGAATACCCCCTGGTTAGTTCAGGCATGGGTGGAA
GATCGTAATGAAAAGAAAATAAAAGAATTCACGCCGACACCAATTGTCTTTAGGGTAGAG
CCCACATCAGTATTTTCCGTTCGAGTGATGAAGACGGGAATTCCTGATGAACAAAAGGAG
AGCTTCTACTGGGTTGTATCAAATTCATTACCTGGTGGGAAAAAGAAAGAGAAACAAAAA
CAGGATGATAAAATCACTGCGAGTATAAATCTCGCATATCGTTTTAAAGTACCTATGATT
TATCGCCCAGCTTCACTGAAGAATATTCCTCAGCAACCTGATAGTCTAGAGTGGTCTGTT
GATAGTAAAGGGGGCATTAAAGTAAAAAACCCTAGCAGGTATGTTGTTCAACTGCATAGT
ATAACCATTAACGGTGTGATAAACAAAGGAAAAGGTATCTCGAAATTCATTCTTCCTATG
AGCGAAGATTCATTAGAACTTAATGCCAAAAATGGTACCAAAATACATTATGGTGTCATA
AACGACTATGGCGCGGTAAAAGAGTATGAGGGTGTTATCAAGTAG
>CS26_crsC E528_7521_1#30 ~~~crsC~~~CS26 crsC Chaperone~~~
GTGTTATCAAGTAGGATCTTTAATTTACTGGTTTTTTTTGTGATCCTTCTATCATTTTGT
GCAGATGCTAAAGCGCCTGAATTAGGCGTTGCGATCGATCCGTTAAAAATAAAAGTAAAA
GAAAATCGGATGGTTTACTTCAGCGTAATCAATGATACAGAAAACGATTACATTGTAACA
ACAAAAGTAGTAAACGCTTTAACTAAAAAAGATTCTGATGCTGAACCTCGTTTTTTAGTC
AACCCACCAATACGGTTACTAAAGAAAAGAGACAAGGCACAAATGGGGGTGGTATATCTA
TCAGAACGACAACTGCCTCCACCTGACTCTAAAATTTATCTTTCCGTTTCATTTATTCCT
AAGGTTCCTGATAAGTCAGCTTTGGTTCATATGCCAGTTATTTTTGTACAGCAAGTACCA
TTAGTTTTTGAATAG
>CS26_crsD E528_7521_1#30 ~~~crsD~~~CS26 crsD outer membrane usher~~~
ATGAGTAAACATCATCTTATTCTTGCTCCTCTAATTTTAGGCATTGGCACTAATGTTGAT
GCCCGGGATTATTTTGACCCTTCTCTTTTAGCGTCAGATGTGGCGGGCGGGGGTGATATT
GATTTGTCTGCATTTGCAATCCCTGGTGGCGGTATGGAGGGTGAGCAAGAAGTAGGTATA

TACATCAATAATGATTTTTATTCAAGAGCAACGCTGAACTTCAAGAACACTGATGAGCGA
GGGTTGCTGCCGGATTTTCCTGCAGATTTTTTTGACGACATTCTATATGAAGAATACTTG
GTTTTTGACAAAACCCAGGTCATTCCCTCTTCAGACTTTCTGGCTAAAGTACCGTTTAGT
GAGGTTACCTTTGATCAGGCTTTGTCTCGAGTAAATGTTAGTATTCCACAAGCTTATATA
GATGAGGGAGCAAAACTTGTTTCTTCCCCTGATACATGGGACTACGGCGTCCCCGCACTA
CTGTTTGATTATAATATATCAGGGAACCGTAACAAATATAATGACTCTAATTCAGAGAGT
TTTTATGCATCTTCGTTGGTAGGCGTTAACCTGTGGGGATGGCGTTTGCGCACTTCGGCT
AACTACAATCAGTATACGACCGACTCAGTGTGGGGAAAAACACGCTCGGAGACGAACAG
C
TTCTTCAATACATACATTGAACGAGATATTAGCTCTTTGCGTGCCGGATTGCGCATAGGT
GAGGCTTCGACTGGGGGTATTATTCTTGATTCAATACCATTCCGGGGGATAAAGCTTTAC
AGCGACGATGATATGTTGGGATATCGCCTCAGAAACTACTCGCCAGCTGTGCGAGGTATT
GCCCGTAGCCAAGCTGTTGTAACGATTAGTCAAAATGGTAGACAAGTATATCAGACAAAC
GTTCCGCCAGGGCCTTTTCAACTTAATGATTTTTATATCTCTGGTTATTCTGGCGATATG
GTCGTGACCGTTCGTGAGGCTGATGGAAGTGAACATAGTTTTGTCCAGCCGTATTCTACA
TTACCCGAAATGAAACGTGAGGGCGTTTCTGGTTTTGAATTGTCCGTGGGCAAGTATGAT
AACTATGGTTCTGATGATTATTATGATCAACCTTCTTTTGTTTATGGCAACTGGTCACGG
GGTTTTTCGTATGGCATCACAACTTTCGGTGAGACACTTCAGGCTGAAAAATATCAAAGC
TTAGGGCTAGGAAGCACTGTTTCTTTGGGAGCGCTTGGTGCGGCATCAGCGGATGTGTCT
GTTTCTCGCGCAGAAAAATATGGTGATGTTAAGACTGGTCAATCTTATGGATTTAAGTAT
TCCAAGAGCCAACTTGAGACTGGTACTACTTTAACACTGGCGACTTATCGTTACTCGACA
AAGAATTTTTATACCTTCAGTGACTTTGCTTCTAACTCAGAAGAAGCTCGTTTTGTATGG
GAAAACAAACTCAAGAACAGAATGACTCTTAGCGTAAGTCAGGCTTTAGGGGCGTATGGT
CATCTGTCACTCAGTGCCAATCAGCAGAGTTACTGGACGAGTAGTGAGGTAACGCGAAAC
TATAGTTTGTCACATAGTTTTAGCTGGGAGGACATCTTCTTTAGTACAACGTTTTCTCTT
GATCAGTCACACAATCGTTATAACGATTACGCTGAGAATAAACAGATTGATTTTTACGTT
AGCGTGCCTTTGAGTAAGTTCCTTGGCGATAAGGATATTACCTCAAGTTCTCTAACATAC
AATGTGACAAACTCAGATCATCGAGTTCGCAACAGTGCGACCTTAAATGGGAACATTCCA
GAAACTGATTTCCGGTATCGTGTGGGCGGTAGCTGGGGTAATAATAATCTTAATAGTACT
AAAACAGCGTCTCTTAGCTGGACGGGAAGTTATACCAGTGCTTCTTTAGGGTATACCTAC
TCTCATAATAACAATACTTTAGATTACAGTTTATCAGGAGCGGCCGTTGCATATCCTTGG
GGGCTTGCATTGGGTAACAACAATGTAACTAACAGTGGCGCTATTGTTGTCGAAACGTCT
GGAGCTCCGGGGGTCAGAACCAGCGCTGGATATAGTACTTCTTTCTTAGGTACTGCACTA
ATAGGTTCGCCGCAAAAATATACAGAAAACAGAATCGACCTGTATCCAGATGGTCTTCCG
GATGATACGGTTTTGGCTGAAACATCGAAGGTAGCAGTACCTGCCAAGGGAGCCGTTGTT
GTTTTAGATTATACCGTGTTCAAAGGTAGCCAGGTTGTTTTCTCACTAAAACAGGAGAGT
GGTAAACCACTACCTTTCGGCACGATCGTTTCTCTCGATGGAATGCCAAAGGGAAAAGAG
AATACAGGAATAGTCGGCGAGAATGGGCGAGTTTATATGGCAGGCATTCCTAAAGAAGGT
ACATTAAAAGCTATATGGGGAAATAAAAATTGTAGTATTAAATTCCACCTGACTGAACAA
AAGTCAGTAGGCCCAATTAGAGAATCTAATGAGGTTTGTAAGCCATGA
>CS26_crsE E528_7521_1#30 ~~~crsE~~~CS26 crsE Chaperone~~~
ATGAGATACTTTTTATTTTTAATGCTTTATTTTTTTGTTGCTCATGGGGTATTTGCCAGT
ATTGACTACCCTTATCCGGAGGAAACTAACGTTTATCTTAAGTGTGATAAAAATGACTCG
CATTTAAGGGGCAGTTTGCGTATAGCAAATCCAGGGATGGGGGTTTGGTTGGTTCAGGTT
TGGACTGAGGATGAAAATCGAAATCGTTACGCTAATGCTTATCCTTCTTTAATGAGACTT
GATCCTAAAACCAGTAGAGTGGTTAGTATATATTCTGAGCAAGTATCCAAAGCAAATAAG
CTGAAATGGTTGCTAGTATCCTTTATACCATCACATCATAAAGATGAACGCAATAAGTTA
GTCATGCCAATATCTTACCGATTGAAAGTAACCACAGGTTCATAA
>CS26_crsF E528_7521_1#30 ~~~crsF~~~CS26 crsF minor subunit~~~
ATGTATTTTGATGATTTTGATGCTGTGGACATCGTCACGGGGAGCGACAAAACCATCGAG
AGTACAACCCTTAATTTTACTGGGTGCAGCAATGTTAAAAAACTTAAAATTACTTTTAAA
CAGACAGGGCAAAATCCATCAATTGATATGGCTAATAATTGGATACCCAACAAAACAGGA
GCAAATATGGCTGAGGGAATTGCAGTTGTCCTGTTAGATGATAAGAATAGTGAAATAAAC
CTAGATCAAATAATGGCCATTGACGTTGGACAATCAGAGACTTCAAAGCAGATTATTCTA
AAATCAAAAGTCGTTCCAACCGATAGAGTTGGTAGAGGGATTATACCCGGGAAACTTGAA
ACTGCAGTTGGGGTTGAGATTTCCTATGAATAA
>CS26_crsG E528_7521_1#30 ~~~crsG~~~CS26 crsG minor subunit~~~

ATGAAAAAAAATAACCTGATAAAATGCTTTTTTTTTGTTTTATTGTTTACTAATGTTTGC
AAAGCAGATGGTAACATAGGTACTGGTTCTTCTTATTCAAGAAACCAGTTAGTAGAAAAA
AATATTAACATCCCTGCTAACTATTATGCTAACACCTGGGTCGACTTAGGGGATTTAGGT
CTTGAAAAATATAACCAAGTGCAGTCAAATTATAACTGCTCAGGTGTGTCAATGTGTAGC
TATACAGCTGTCAGGGTAAATGGTGGTGGCCTGAACCCAGGTGCGTATTATAAAGTAAGA
ATCAGCGCAACTCCTCAGAAAAAATATATTAATGGAAAGGAGTTCATTTTTTCAGCATAT
TTTAAAGGTTCTCCCCAGATAATCTGGCAGGAAACAAACATTAATAATAGGAACACGTGG
TACCATCCTTACACAGTACCTGTATCTAATTTTGGTTCCAGTGCAGGAACTTCCACTGAT
TATTCTACATCAGGAAACCCCAGCGGAAAATGTGGTGTTATTGGTGGTTGTTCATATGCC
GCGACTACCTATTTTGGCCCAAGCTCAGGAAGCTTACACTTAAGCCTACAATTTCCTTCA
AATTTAGCATCAGGTACATATCAGTTTAATGATGTTGAGATATTAAGATTATGGCAACAA
TCTAGTAATGCTTCTGGAACGAATGTTTACTCTTCAGAGGCAGTCTTTAAAATTAGTGGC
GTGATTACGTTACCCAACAGGTGCTATATTTCTAGCTCACAAAATACATTCAATTTTAGA
GATGTGAATGTTAATGCGGAAAATGGAAAGTTAGAAACTCAGGAATATTATACAATAACA
ACCTGTCAAGGTATACTATCAAATGTAAAACAATATCTAACAGTTGACGGCGGGAATGAT
GATTACATAAAACCTTTTTCTTTTGATGCTAATGGAAGCAAAGCTTTAGGATTTGCAATG
ATGATAGTTCCGCAGGGGTTATCTGGGGAGCCTAATTGTAATGCAGCAACTGATGATAGA
AATAGATTTAACAAAGAATATCTTATAAGAACCATCACTCCTGCTAACTATCAGACTTTT
CAAGATACTTTAAAGTTTTCATTGTGTAAATATGGTATACCTTCTTCTGCGTACATTGGT
GAAAATAATGTATCAATAAAAATAATCTCCCGATGGGAGAACTAG
>CS27A_gene1 E604_7521_1#32 ~~~gene1~~~CS27A transcriptional regulator
ATGAAGCGCAAGTACCTTTCAGAAAAAGAGGTGCATTGTTTACTTAAAGCGGCTGCGGAA
AATCAATACTCAGTTCGTGATTACTGCATGATAAGTATGGCGTTTATTCATGGATTAAGA
GTGAGTGAATTAGTAAACCTCAAAGTAGATGACTACGACTCTCTATCTGCTCAATTAAAC
ATCAAAAGACTTAAAAATGGATTCTGTACGATTCAACCATTATTACCTGATGAAAATGAA
TTACTTCAATGTTGGCTAGATGAACGTAAAACATGGGATGGACATGAAAGCTGTTGGTTA
TTTCTGTCTAAAAATGGTGGACCTCTAAGTCGCCAACGCTTTTGGCAATTATTACGTAAG
TATGGAGATGAAGCACATCTTACCATAAAGGTTCACCCACATATGTTAAGACATGCTTGC
GGATTTAATCTTGCAGAACGCGGCAATGACACGAGATTAATACAGGATTACTTAGGACAT
AGAAACATAAGGCACACAGTGCATTACACTGCCAGTAATCCCGAACGTTTTAGAAACGCA
TGGACAAAGAATAACTTTCATAGTTTCTCATGCACTCATGCACTCATGCACTCATGCATT
TAA
>CS27A_gene2 E604_7521_1#32 ~~~gene2~~~CS27A transcriptional regulator~~~
ATGACTAAGCGAAAATACCTGACCCAAAGTGAGGTGGAGCTAATGCTTACTGAAGCAAAA
CATGGACAATTCTCAGAGAGGAATTATTGCTTGCTTTATCTTAGTTTCATTCATGGGTTT
CGCGTCAGTGAAGTATGTAATCTAAGGCTTGAAGATGTATGCCTGCGTGATAAAAGCTTA
AATATTCACCGAATGAAGAATGGGTTTAGCACAATTCATCCACTTCTATCCGATGAAGTA
AAAGTGCTTAAAGCATGGTTATGTGTGAGGGATAAAATGAATGGCTCTGAAAGCGAGTGG
TTATTTGTATCGCGGCATGGGGGGGCTTTAAGCCGGCAGCGTGTCTGGCAAATGATTCAA
AATCTTGGGAAGAAGGCCGGTGTAAAAGTGAACTCTCATCCACACATGCTAAGACATGCA
TGTGGCTATGCTTTGGCCGATAGGGGTGTTGATACTCGTCTAATACAAGATTATTTGGGG
CATAGAAACATACGACATACAGTTTGTTATACCGCAAGTAATGCTGAGCGTTTTAAGTAT
ATTTGGTTAAAAAAAGGTCTAAAGGTTAACAATTAG
>CS27A_gene3 E604_7521_1#32 ~~~gene3~~~CS27A major subunit
ATGAAAAAGACAATTATGTCTCTGGCTGTGGTTTCAGCTTTAGTAAGCGGTTCTGCATTT
GCAGCAGCTCCTCAGAACGATTCAAGTAAGGCAACCATCAATTTTAATGGTAAAGTGACT
TCCAGTCTTTGCCAGGTGAGAACTGATAATTTAAGCCAGGATATTTCTCTGGGTGAAGTT
ACAGCGACTGTACTAAAAGATTCTGGCAAAGGTAAAGCGCAGAGCTTCCAGGTTGGTCTG
ACGAACTGCGATACCACTACCAGCAAAATTTCTTATGTACTGACAGATGGTAACTACACT
CCGAGCCTAGATAGTAGCAATGCCGCAGTAAAACTGAGTTATCTACAACCTAAATCTGGT
GATAACTCTGCAGACGGTGTTGGTGTGTTTATTGAAACGAGCACTGGTACAAAGATTGCC
CCAGGGTCAACTACCAATCTTGATGTAGCAAAAGACGGTAACTCCCAGGCTCTTTCCGAC
CAGACCATTTCTCTGCGTGCCTATATCGGTACCAAGGATGGTCAACCAGACGCTAATCAG
AGTGTTAAAGCCGGTACTGTTGAGGCAACTGGTATACTGACCATTTACGCTGGTGTTTAA
>CS27A_gene4 E604_7521_1#32 ~~~gene4~~~CS27A chaperone~~~
ATGTCAAGCACGGCCATAGCTGGTTCATTTGGCCCAAGAGAGAATAGGCTCATTTTTGAG
GGCGACAAAACTTATGTGCAGTATCGTATTGATAACACGGATAAGGACACCCCTTGGTTA

GTTCAGGCATGGGTGGAGGATAGTGATGAAAAGAAAATAAAAGAATTCATGCCGACACCA
ATTGTCTTTAGGGTAGAGCCCACATCAGTATTTTCCGTTCGAGTGATGAAGACGGGGATT
CCTGATGAACAAAAGGAGAGCTTCTACTGGGTTGTATCAAATTCTTTACCTGGTGGGGAA
AAGAAAGAGAAACAAAAACAGGATAATAAAATAACTGCGAGCATAAATCTGGCATATCGT
TTTAAAGTACCAATGATTTATCGCCCAGCTTCATTGAAAAATATTCCACAGCAACCTGAG
CGTCTTGAGTGGTCTATTAATGATAAAGGGAGCATTAAAGTGAAAAATCCCAGTAGGTAT
GTTGTTCAGTTGCATAGTTTAACCATTAATGGTTCTGAATACAAAGGCAATGGAGTTTCA
AATTTTATTTTACCAATGAAAGATGTCTCATTGAGCGTTAATGCAAAACTTGGTTCAAAA
ATTAAGTATGGTGTCATAAACGACTATGGCGCGGTAAAAGAGTATGAGGGTATTATCAAG
TCGGTATTTTAA
>CS27A_gene5 E604_7521_1#32 ~~~gene5~~~CS27A chaperone~~~
ATGAGGGTATTATCAAGTCGGTATTTTAATTTAGTGGGAGTTTTTTTGTAATCTTTTTA
TCAGTTGCTGCATATGCTAAAGCGCCCGAATTAGGCGTTGCGATCGATCCGCTAAAAATA
AAAGTAAAACCAAATCGGATGGTTTACTTCAGCGTAATCAATGATACAGAAAACGATTAC
ATTGTAACAACAAAAGCAATAAACGCTTTAACTAAAAAAGATTTGATGTTGAACCAAGT
TTTTTAGTCAACCCGCCAATACGGTTACTAAAGAAAAGAGACAAGGCACAAATGGGAGTG
GTATACCTATCGGAACGACAACTGCCTTCACCTGGTTCTAAATTTTATCTTTCCGTTTCA
TTTATTCCTAAGGTTCCTGATAAGTCAGCTTTGGTTCATATGCCAGTTATTTTAGTACAG
CAAGTACCATTAATTTTTGAATAG
>CS27A_gene6 E604_7521_1#32 ~~~gene6~~~CS27A outer membrane usher~~~
ATGAGTAAACATCATCTTATTCTTGCTCCTCTAATTTTAGGCATTGGCACTAATGTTGAT
GCTCGGGATTATTTTGACCCTTCTCTTTTAGCGTCAGATGTGGCGGGCGGGGGTGATATT
GATTTGTCTGCATTTGCAATCCCTGGTGGCGGTATGGAGGGTGAGCAAGAAGTAGGTATA
TATATCAATAATGATTTTTATTCAAGAGCAACGCTGAACTTTAGAAATACTGATGAGCGG
GGGTTGCTGCCGGATTTTCCTGCGGATTTTTTTGACGACATTCTATATGAAGAATACTTG
GTTTTTGACAAAACCCAGGTCATTCCCTCTTCATACTTTCTGGCTAAAGTACCGTTTAGT
GAGGTTACCTTTGATCAGGCTTTGTCTCGAGTAAATGTTAGTATTCCACAAGCTTATATA
GATGAGGGTGCAAAACTTGTTTCCTCTCCTGATACATGGGACTACGGCGTCCCCGCACTG
CTGTTTGATTATAATATATCAGGGAACCGTAACAAATATAATGACTCTAACTCAGAGAGT
TTTTATGCATCTTCGTTGGCAGGCGTTAACCTGTGGGGATGGCGTTTACGCACTTCGGCT
AACTATAATCAGTATACGACCGACTCAGCGTGGGGAAAAACACGCTCGGAGACGAACAGC
TTCTTCAATACATACATTGAACGAGATATTAGCTCTTTGCGTGCCGGATTGCGCATAGGT
GAGGCTTCGATTGGGGGTATTATTCTTGATTCAATACCATTCCGGGGGATAAAGCTTTAC
AGCGACGATGATATGTTGGGATATCGCCTCAGAAACTACTCGCCAACTGTGCGAGGTATT
GCCCGTAGCCAAGCTGTTGTAACGATTAGTCAAAATGGTAGACAAGTATATCAGACAAAC
GTTCCGCCAGGGCCTTTTCAACTTAATGATTTTTATATCTCTGGTTATTCTGGCGATATG
GTCGTGACCGTTCGTGAGGCTGATGGAAGTGAACATAGTTTTGTCCAGCCGTATTCTACA
TTACCCGAAATGAAACGTGAGGGCGTTTCTGGTTTTGAATTGTCCGTGGGCAAGTATGAT
AACTATGGTTCTGATGATTATTATGATCAACCTTCTTTTATTTATGGCAACTGGTCACGG
GGTTTTTCGTATGGCATCACAACTTTCGGTGAGACACTTCAGGCTGAAAAATATCAAAGC
TTAGGGCTAGGAAGCACTGTTTCTTTGGGAGCGCTTGGTGCGGCATCAGCGGATGTGTCT
GTTTCTCGCGCAGAAAAATATGGTGATGTTAAGACTGGTCAATCTTATGGATTTAAGTAT
TCCAAGAGCCAACTTGAGACTGGTACTACTTTAACACTGGCGACTTATCGTTACTCGACA
AAGAATTTTTATACCTTCAGTGACTTTGCTTCTAACTCAGAAGAAGCTCGTTTTGTATGG
GAAAACAAACTCAAGAACAGAATGACTCTTAGCGTAAGTCAGGCTTTAGGGGCGTATGGT
CATCTGTCACTCAGTGCCAATCAGCAGAGTTACTGGACGAGTAGTGAGGTAACGCGAAAC
TATAGTTTGTCACATAGTTTTAGCTGGGAGGACATCTTCTTTAGTACAACGTTTTCTCTT
GATCAGTCACACAATCGTTATAACGATTACGCTGAGAATAAACAGATTGATTTTTACGTT
AGCGTGCCTTTGAGTAAGCTCCTTGGCGATAAGGATATTACCTCAAGTTCTCTAACATAC
AATGTGACAAACTCAGATCATCGAGTTCGCAATAGTGCGACCTTAAATGGGAACATTCCA
GAAACTGATTTCCGGTATCGTGTGGGCGGTAGCTGGGGTAATAATAATCTTAATAGTACT
AAAACAGCGTCTCTTAGCTGGACGGGAAGTTATACCAGTGCTTCTTTAGGGTATACCTAC
TCTCATAATAACAATACTTTAGATTACAGTTTATCAGGAGCGGCCGTTGCATATCCTTGG
GGGCTTGCATTGGGTAACAACAATGTAACTAACAGTGGCGCTATTGTTGTCGAAACGTCT
GGAGTTCCGGGGGTCAGAACCAGCGCTGGATATAGTACTTCTTTCTTAGGTACCGCACTA
ATAGGTTCGCCGCAAAAATATACAGAAAACAGAATCGACCTGTATCCAAATGGTCTTCCG
GATGATACGGTTTTGGCTGAAACATCGAAGGTAGCAGTACCTGCCAAGGGAGCCGTTGTT

GTTTTAGATTATACCGTGTTCAAAGGTAGCCAGGTTGTTTTCTCACTAAAACAGGAGAGT
GGTAAACCACTACCTTTCGGCACGATCGTTTCTCTCGATGGAATGCCAAAGGGAAAAGAG
AATACAGGAATAGTCGGCGAGAATGGGCGAGTTTATATGGCAGGCATTCCTAAAGAAGGT
ACATTAAAAGCTATATGGGGAAATAAAAATTGTAGTATCAAATTCCACCTGGCTGAACAA
AAGTCAGTAGGCCCAATTAGAGAATCTAATGAGGTTTGTAAGCCATGA
>CS27A_gene7 E604_7521_1#32 ~~~gene7~~~CS27A chaperone~~~
ATGAGATACTTTTTATTTTTAATGCTTTATTTTTTTGTTGCTCATGGGGTATTTGCCAGT
ATTGACTACCCTTATCCGGAGGAAACTAACATTTATCTTAAGTGTGATAAAAATGACTCG
CATTTAAGGGGCAGTTTGCGTATAGCAAATCCAGGGATGGGGGTTTGGTTGGTTCAGGTT
TGGACTGAGGATGAAAATCGAAATCGTTACGCTAATGCTTATCCTTCTTTAATGAGACTT
GAGCCTAAAACCAGTAGAGTAGTTAGTATATATTCTGAACAAGTATCCAAAGCAAATAAG
CTGAAATGGTTGCTAGTATCCTTTATACCATCACATCATAAAGATGAACGCAATAAGTTA
GTCATGCCAATATCTTACCGATTGAAAGTAACCACAGGTTCATAA
>CS27A_gene8 E604_7521_1#32 ~~~gene8~~~CS27A minor subunit~~~
ATGTATTTTGATGATTTTGATGCTGTGGACATCGTCACGGGGAGCGACAAAACCATCGAG
AGTACAACCCTTAATTTTACTGGGTGCAGCAATGTTAAAAAACTTAAAATTACTTTTAAA
CAGACAGGGCAAAATCCATCAATTGATATGGCTAATAATTGGATACCCAACAAAACAGGA
GCAAATATGGCTGAGGGAATTGCAGTTGTCCTGTTAGATGATAAGAATAGTGAAATAAAC
CTAGATCAAATAATGGTCATTGACGTTGGACAATCAGAGACTTCAAAGCAGATTATTCTA
AAATCAAAAGTCGTTCCAACCGATAGAGTTGGTAGAGGGATTATACCCGGGAAACTTGAA
ACTGCAGTTGGGGTTGAGATTTCCTATGAATAA
>CS27A_CS27A-gene9 E604_7521_1#32 ~~~CS27A-gene9~~~CS27A minor subunit~~~
ATGAAAAAAAATAACCTGATAAAATGCTTTTTTTTGTTTTATTGTTTACTAATGTTTGC
AAAGCAGATGGTAACATAGGTACTGGTTCTTCTTATTCAAGAAACCAGTTAGTAGAAAAA
AATATTGACATCCCTACTAACTATTATGCTAACACCTGGGTCGACTTAGGGGATTTAGGT
CTTGAAAAATATAACCAAGTGCAGTCAAATTATAACTGCTCAGGTGTGTCAATGTGTAGC
TATACAGCTGTCAGGGTAAATGGTGGTGGCCTGAACCCAGGTGCGTATTATAAAGTAAGA
ATCAGCGCAACTCCTCAGAAAAAATATATTAATGGAAAGGAGTTCATTTTTTCAGCATAT
TTTAAAGGTTCTCCCCAGATAATCTGGCAGGAAACAAACATTAATAATAGGAATACGTGG
TTCCATCCTTACACAATACCTGTATCTAATTTTGGTTCCAGTGCAGGAACTACCACTGAT
TATTCTACATCAGGAAACCCCAGCGGAAAATGTGGTGTTATTGGTGGTTGTTCATATGCC
GCGACTACCTATTTTGGCCCAAACTCAGGAAGCTTACACTTAAGCCTACAATTTCCTTCA
AATTTAGCATCAGGTACATATCAGTTTAATGATGTTGAGATATTAAGATTATGGCAACAA
TCTAGTAATGCTTCTGGAACGAATGTTTACTCTTCAGAGGCAGTCTTTAAAATTAGTGGC
GTGATTACGTTACCCAACAGGTGCTATATTTCTAGCTCACAAAATACATTCAATTTTAGA
GATGTGAATGTTAATGCAGAAAATGGAAAGTTAGAAACTCAGGAATATTATACAATAACA
ACCTGTCAAGGTATACTATCAAATGTAAAACAATATCTAACAGTTGACGGCGGGAATGAT
GATTACATAAAACCTTTTTCTTTTGATGCTAATGGAAGTAAAGCTTTAGGATTTGCAATG
ATGATAGTTCCGCAGGGGGTATCTGGGGAGCCTAATTGTAATGCAGCAACTGATGATAGA
AATAGATTTAACAAAGAATATCTTATAAGAATCATCACTCCTGCTAACTATCAGACTTTT
CAAGATACTTTAAAGTTTTCATTGTGTAAATATGGTATACCTTCTTCTGCGTACATTGGT
GAAAATAATGTATCAATAAAAATAATCTCCCGATGGGAGAACTAG
>CS27B_gene1 E451_7521_1#23 ~~~gene1~~~CS27B major subunit~~~
ATGAAAAAGACAATTATGTCTCTGGCTGTGGTTTCAGCTTTAGTAAGCGGTTCTGCATTT
GCAGCAGCTCCTCAGAACGATTCAAGTAAGGCAACCATCAATTTTAATGGTAAAGTGACT
TCCAGTCTTTGCCAGGTGAGAACTGATAATTTAAGCCAGGATATTTCTCTGGGTGAAGTT
ACAGCGACTGTACTAAAAGATTCTGGCAAAGGTAAAGCGCAGAGCTTCCAAGTTGGTCTG
ACGAACTGCGATACCACTACTAGCAAAATTTCTTATGTACTGACAGATGGTAACTACACT
CCGAGCCTAGATAGTAGCAATGCCGCAGTAAAACTGAGTTATCTACAACCTAAATCTGGT
GATAACTCTGCAGACGGTGTTGGTGTGTTTATTGAAACGAGCACTGGTACAGCGATTGCC
CCAGGGTCAACTACCAATCTTGATGTAGCAAAAGACGGTAAGTCCCAGGCTCTTTCCGAC
CAGACCATTTCTCTGCGTGCCTATATCGGTACCAAGGATGGTAATCCAGACGCTAATCAG
AGTGTTAAAGCCGGTACTGTTGAGGCAACTGGTATACTGACCATTTACGCTGGTGTTTAA
>CS27B_gene2 E451_7521_1#23 ~~~gene2~~~CS27B chaperone~~~
ATGTCAAGCACGGCCATAGCTGGTTCATTTGGCCCAAGAGAGAATAGGCTCATTTTTGAG
GGCGACAAAACTTATGTGCAGTATCGTATTGATAACACGGATAAGGACACCCCTTGGTTA
GTTCAGGCATGGGTGGAGGATAGTGATGAAAAGAAAATAAAAGAATTCATGCCGACACCA

ATTGTCTTTAGGGTAGAGCCCACATCAGTATTTTCCGTTCGAGTGATGAAGACGGGGATT
CCTGATGAACAAAAGGAGAGCTTCTACTGGGTTGTATCAAATTCTTTACCTGGTGGGGAA
AAGAAAGAGAAACAAAAACAGGATAATAAAATAACTGCGAGCATAAATCTGGCATATCGC
TTTAAAGTACCAATGATTTATCGCCCAGCTTCATTGAAAAATATTCCACAGCAACCTGAG
CGTCTTGAGTGGTCTATTAATGATAAAGGGAGCATTAAAGTGAAAAATCCCAGTAGGTAT
GTTGTTCAGTTGCATAGTTTAACCATTAATGGTTCTGAATACAAAGGCAATGGAGTTTCA
AATTTTATTTTACCAATGAAAGATGTCTCATTGAGCGTTAATGCAAAACTTGGTTCAAAA
ATTAAGTATGGTGTCATAAACGACTATGGCGCGGTAAAAGAGTATGAGGGTATTATCAAG
TCGGTATTTTAA
>CS27B_gene3 E451_7521_1#23 ~~~gene3~~~CS27B chaperone~~~
ATGAGGGTATTATCAAGTCGGTATTTTAATTTAGTGGGAGTTTTTTTTGTAATCTTTTTA
TCAGTTGCTGCATATGCTAAAGCGCCCGAATTAGGCGTTGCGATCGATCCGCTAAAAATA
AAAGTAAAACCAAATCGGATGGTTTACTTCAGCGTAATCAATGATACAGAAAACGATTAC
ATTGTAACAACAAAAGCAATAAACGCTTTAACTAAAAAAGATTTTGATGTTGAACCAAGT
TTTTTAGTCAACCCGCCAATACGGTTACTAAAGAAAAGAGACAAGGCACAAATGGGAGTG
GTATACCTATCGGAACGACAACTGCCTTCACCTGGTTCTAAATTTTATCTTTCCGTTTCA
TTTATTCCTAAGGTTCCTGATAAGTCAGCTTTGGTTCATATGCCAGTTATTTTAGTACAG
CAAGTACCATTAATTTTTGAATAG
>CS27B_gene4 E451_7521_1#23 ~~~gene4~~~CS27B outer membrane usher~~~
ATGAGTAAACATCATCTTATTCTTGCTCCTCTAATTTTAGGCATTGGCACTAATGTTGAT
GCTCGGGATTATTTTGACCCTTCTCTTTTAGCGTCAGATGTGGCGGGCGGGGGTGATATT
GATTTGTCTGCATTTGCAATCCCTGGTGGCGGTATGGAGGGTGAGCAAGAAGTAGGTATA
TATATCAATAATGATTTTTATTCAAGAGCAACGCTGAACTTTAGAAATACTGATGAGCGG
GGGTTGCTGCCGGATTTTCCTGCGGATTTTTTTGACGACATTCTATATGAAGAATACTTG
GTTTTTGACAAAACCCAGGTCATTCCCTCTTCATACTTTCTGGCTAAAGTACCGTTTAGT
GAGGTTACCTTTGATCAGGCTTTGTCTCGAGTAAATGTTAGTATTCCACAAGCTTATATA
GATGAGGGTGCAAAACTTGTTTCCTCTCCTGATACATGGGACTACGGCGTCCCCGCACTG
CTGTTTGATTATAATATATCAGGGAACCGTAACAAATATAATGACTCTAACTCAGAGAGT
TTTTATGCATCTTCGTTGGCAGGCGTTAACCTGTGGGGATGGCGTTTACGCACTTCGGCT
AACTATAATCAGTATACGACCGACTCAGCGTGGGGAAAAACACGCTCGGAGACGAACAGC
TTCTTCAATACATACATTGAACGAGATATTAGCTCTTTGCGTGCCGGATTGCGCATAGGT
GAGGCTTCGATTGGGGGTATTATTCTTGATTCAATACCATTCCGGGGGATAAAGCTTTAC
AGCGACGATGATATGTTGGGATATCGCCTCAGAAACTACTCGCCAACTGTGCGAGGTATT
GCCCGTAGCCAAGCTGTTGTAACGATTAGTCAAAATGGTAGACAAGTATATCAGACAAAC
GTTCCGCCAGGGCCTTTTCAACTTAATGATTTTTATATCTCTGGTTATTCTGGCGATATG
GTCGTGACCGTTCGTGAGGCTGATGGAAGTGAACATAGTTTTGTCCAGCCGTATTCTACA
TTACCCGAAATGAAACGTGAGGGCGTTTCTGGTTTTGAATTGTCCGTGGGCAAGTATGAT
AACTATGGTTCTGATGATTATTATGATCAACCTTCTTTTATTTATGGCAACTGGTCACGG
GGTTTTTCGTATGGCATCACAACTTTCGGTGAGACACTTCAGGCTGAAAAATATCAAAGC
TTAGGGCTAGGAAGCACTGTTTCTTTGGGAGCGCTTGGTGCGGCATCAGCGGATGTGTCT
GTTTCTCGCGCAGAAAAATATGGTGATGTTAAGACTGGTCAATCTTATGGATTTAAGTAT
TCCAAGAGCCAACTTGAGACTGGTACTACTTTAACACTGGCGACTTATCGTTACTCGACA
AAGAATTTTTATACCTTCAGTGACTTTGCTTCTAACTCAGAAGAAGCTCGTTTTGTATGG
GAAAACAAACTCAAGAACAGAATGACTCTTAGCGTAAGTCAGGCTTTAGGGGCGTATGGT
CATCTGTCACTCAGTGCCAATCAGCAGAGTTACTGGACGAGTAGTGAGGTAACGCGAAAC
TATAGTTTGTCACATAGTTTTAGCTGGGAGGACATCTTCTTTAGTACAACGTTTTCTCTT
GATCAGTCACACAATCGTTATAACGATTACGCTGAGAATAAACAGATTGATTTTTACGTT
AGCGTGCCTTTGAGTAAGCTCCTTGGCGATAAGGATATTACCTCAAGTTCTCTAACATAC
AATGTGACAAACTCAGATCATCGAGTTCGCAATAGTGCGACCTTAAATGGGAACATTCCA
GAAACTGATTTCCGGTATCGTGTGGGCGGTAGCTGGGGTAATAATAATCTTAATAGTACT
AAAACAGCGTCTCTTAGCTGGACGGGAAGTTATACCAGTGCTTCTTTAGGGTATACCTAC
TCTCATAATAACAATACTTTAGATTACAGTTTATCAGGAGCGGCCGTTGCATATCCTTGG
GGGCTTGCATTGGGTAACAACAATGTAACTAACAGTGGCGCTATTGTTGTCGAAACGTCT
GGAGTTCCGGGGGTCAGAACCAGCGCTGGATATAGTACTTCTTTCTTAGGTACCGCACTA
ATAGGTTCGCCGCAAAAATATACAGAAAACAGAATCGACCTGTATCCAAATGGTCTTCCG
GATGATACGGTTTTGGCTGAAACATCGAAGGTAGCAGTACCTGCCAAGGGAGCCGTTGTT
GTTTTAGATTATACCGTGTTCAAAGGTAGCCAGGTTGTTTTCTCACTAAAACAGGAGAGT

GGTAAACCACTACCTTTCGGCACGATCGTTTCTCTCGATGGAATGCCAAAGGGAAAAGAG
AATACAGGAATAGTCGGCGAGAATGGGCGAGTTTATATGGCAGGCATTCCTAAAGAAGGT
ACATTAAAAGCTATATGGGGAAATAAAAATTGTAGTATCAAATTCCACCTGGCTGAACAA
AAGTCAGTAGGCCCAATTAGAGAATCTAATGAGGTTTGTAAGCCATGA
>CS27B_gene5 E451_7521_1#23 ~~~gene5~~~CS27B chaperone~~~
ATGAGATACTTTTTATTTTTAATGCTTTATTTTTTTGTTGCTCATGGGGTATTTGCCAGT
ATTGACTACCCTTATCCGGAGGAAACTAACATTTATCTTAAGTGTGATAAAAATGACTCG
CATTTAAGGGGCAGTTTGCGTATAGCAAATCCAGGGATGGGGATTTGGTTGGTTCAGGTT
TGGACTGAGGATGAAAATCGAAATCGTTACGCTAATGCTTATCCTTCTTTAATGAGACTT
GAGCCTAAAACCAGTAGAGTAGTTAGTATATATTCTGAACAAGTATCCAAAGCAAATAAG
CTGAAATGGTTGCTAGTATCCTTTATACCATCACATCATAAAGATGAACGCAATAAGTTA
GTCATGCCAATATCTTACCGATTGAAAGTAACCACAGGTTCATAA
>CS27B_gene6 E451_7521_1#23 ~~~gene6~~~CS27B minor subunit~~~
ATGTATTTTGATGATTTTGATGCTGTGGACATCGTCACGGGGAGCGACAAAACCATCGAG
AGTACAACCCTTAATTTTACTGGGTGCAGCAATGTTAAAAAACTTAAAATTACTTTTAAA
CAGACAGGGCAAAATCCATCAATTGATATGGCTAATAATTGGATACCCAACAAAACAGGA
GCAAATATGGCTGAGGGAATTGCAGTTGTCCTGTTAGATGATAAGAATAGTGAAATAAAC
CTAGATCAAATAATGGTCATTGACGTTGGACAATCAGAGACTTCAAAGCAGATTATTCTA
AAATCAAAAGTCGTTCCAACCGATAGAGTTGGTAGAGGGATTATACCCGGGAAACTTGAA
ACTGCAGTTGGGGTTGAGATTTCCTATGAATAA
>CS27B_gene7 E451_7521_1#23 ~~~gene7~~~CS27B minor subunit~~~
ATGAAAAAAAATAACCTGATAAAATGCTTTTTTTTTGTTTTATTGTTTACTAATGTTTGC
AAAGCAGATGGTAACATAGGTACTGGTTCTTCTTATTCAAGAAACCAGTTAGTAGAAAAA
AATATTGACATCCCTACTAACTATTATGCTAACACCTGGGTCGACTTAGGGGATTTAGGT
CTTGAAAAATATAACCAAGTGCAGTCAAATTATAACTGCTCAGGTGTGTCAATGTGTAGC
TATACAGCTGTCAGGGTAAATGGTGGTGGCCAGAACCCAGGTGCGCATTATAAAGTAAGA
ATCAGCGCAACTCCTCAGAAAAAATATATTAATGGAAAGGAGTTCATTTTTTCAGCATAT
TTTAAAGGTTCTCCCCAGATAATCTGGCAGGAAACAAACATTAATAATAGGAAGACGTGG
TACCATCCTTACACAATACCTGTATCTAATTTTGGTTCCAGTGCAGGAACTACCACTGAT
TATTCTACATCAGGAAACCCCAGCGGAATATGTGGTGTTATTGGTGGTTGTTCATATGCC
GCGACTACCTATTTTGGCCCAAACTCAGGAAGCTTACACTTAAGCCTACAATTTCCTTCA
AATTTAGCATCAGGTACATATCAGTTTAATGATGTTGAGATATTAAGATTATGGCAACAA
TCTAGTAATGCTTCTGGAACGAATGTTTACTCTTCAGAGGCAGTCTTTAAAATTAGTGGC
GTGATTACGTTACCCAACAGGTGCTATATTTCTAGCTCACAAAATACATTCAATTTTAGA
GATGTGAATGTTAATGCGGAAAATGGAAAGTTAGAAACTCAGGAATATTATACAATAACA
ACCTGTCAAGGTATACTATCAAATGTAAAACAATATCTAACAGTTGACGGCGGGAATGAT
GATTACATAAAACCTTTTTCTTTTGATGCTAATGGAAGTAAAGCTTTAGGATTTGCAATG
ATGATAGTTCCGCAGGGGTTATCTGGGGAGCCTAATTGTAATGCAGCAACTGATGATAGA
AATAGATTTAACAAAGAATATCTTATAAGAATCATCACTCCTGCTAACTATCAGACTTTT
CAAGATACTTTAAAGTTTTCATTGTGTAAATATGGTATACCTTCTTCTGCGTACATTGGT
GAAAATAATGTATCAATAAAAATAATCTCCCGATGGGAGAACTAG
>CS28A_gene1 E833_7521_2#53_04435 ~~~gene1~~~CS28A cfaD-like regulator~~~
ATGAGTCTAGCTTATGGGTTTAGCAAAATTAATTCTCTTGACTTTGGGGCTGACTCTCCT
AAAATAAGGAAGATAATAAGCATCGAAGTAAATGACAGAATCAAAAACTCCTTTTACAAT
ATAATTTCTGAGGATAGCCATCATAATAGAATATTATCATTTATATTCTTTTGTAAAGAA
TCTAGTGTAATAGATGCAATACTCCCTCTCATATACTTCTCAGCTGCAACAACGTTTTGT
AATAGGGTGATAGAATTAATAGAATCAAACATAGCAAAGAAATGGACATTGAGATTATTG
TCTGAGGAATTTAGCATCTCAGAAATAACGATAAGAAAAAAATTAGAAGCAGAAGGGGTT
ATCTTCAGAGAACTCATTCTTGAAATAAGGATGAAAAAGGCAATGACTCTTTTAATAGCA
GGGGAGTTGTCTGTATCTAAGATAGCCGATAAGGTTGGCTACAATAATATATCATACTTC
ATATCCCATTTCAGACAATTTTTCGGAATGACGCCAAAACAACTTCATCTGTTATTAACA
AAATAA
>CS28A_gene2 E833_7521_2#53_04436 ~~~gene2~~~CS28A major subunit~~~
ATGAAAAAAATGATTATGCCTTTAACTATGGTGTCCGTTCTGATGAGCGGCTCTGCTCTG
GCTAAACCGGGGGCTAACGATTCCAGTCAGGCCACGCTGAATTTTAATGGTCGTGTGACA
TCCAGCCTTTGCCAGGTAAAAACAGATGATGTGGTAAAAGATATTTATTTGGGTGAGGTA
TCCAAATCCGCACTGGAAGCTAATGCTCAGGGGCCTAAACAGAGCTTCCAGGTTAACCTG

ATTAACTGTGATACCACCGTCAGCGACATTTCCTATGTATTGAGTGATGCAAATGGTAAT
GGTACCGCCGCTTATCTGATTCCAAAATCAGGTGATACTTCTGCAACCGGCGTAGGTGTT
TATGTGGAAAAGAGTGACGGTACTTCTGTAGGTGTCGGAGATACGCAGACTCTGACGGTT
ACGAAAAATGATGCTAATGCTCTTTCTGAACAAGTAATTCCTCTGCGTGCCTATATTCGT
GCTCAGGGTGGGGCAGGTGGCGTTACTGCTGGTACGGTTGATGCAACTGGTATTTTGACC
ATTCGCGCAACTGCAAATCCGTAA
>CS28A_gene3 E833_7521_2#53_04437 ~~~gene3~~~CS28A chaperone~~~
ATGCGTTATCAGTATTCTGTTTTATTCGTCTTTTTATTCTCTGGCGTTGTACAGGCGGCG
GAGTTTTTCGGTCCGCGGGAAAACCGGTTGCTTTTTGAAGAGAAGAAAGGAAACACCTTT
TACCGGATTGACAATTCAGACAAAAAACTTCCCTGGCTTGTACAGGCATGGATTGAAGAT
GCGTCTGAAAAGAAAACAACCGCACTGACAGCCACGCCCATGGTGTTTCGCGTTGAGCCT
TCTTCTGTATTTACTGTCAGGGTGGTTAAAACGGGAGCCCTGCCGGAAGACCGGGAAACA
CTTTTCTGGGCCGTTTCCAATTCATTACCGGGAGGTGCCTCAACAAAGCAGGATAATGAA
GAGGGAAAAATCAGCGCTAAAATCAGCCTGGCTTACCGGTTTAAGGTGCCACTTATTTAC
CGGCCTGCAGCGTTGGATAATTTCAGGCAGGAGCCGGAGAAACTGGAATGGACATATAAT
GGCAAGGAAGGGCTGAAATTATATAACCCTACCCGTTATGTGGTCCAGTTACATAACGTG
ACGGCGAACGGACGTGAATTTAAGGGCAAAGGGGTTTCGTTTATTCTTCTTCCGATGTCC
GGTAAAAATGTGAGTGCCGCTGTGAATAAAGGCACCAGAATAAAATATGGTGTCATAAAT
GACTATGGCGCGGTAAAAGAATATGATGGTGTTGTTAAGTAA
>CS28A_gene4 E833_7521_2#53_04438 ~~~gene4~~~CS28A chaperone~~~
ATGATGGTGTTGTTAAGTAATGCTCTAAAAAAAGCAGTGCTTTCCCTGTTGGCGTCCTGT
AGCATAGTTGTTTTTGCATCTGAATCAGAACTTGGCGTTGCTGTAGAACCGATGAGGTTA
TCAATTAAGCCTGGAGAGATGACATATTTTACGGTTATCAATGAGACAGAGAGAGAGTAC
ATTGTCACCACCAGGGTAGTAAGTGACTCAGGGATTGATGATAAAGATAAAGTATTTGTC
TTTAGTCCCCCCCTGAAGCATTTAAAAAAAAGGGAACAGTCTGTAATGGGAGTTGTATAT
CTGAAAAATGGGAGAGAGAATAAGATGAAATACTATTTATCTGTCTCATTTGTCCCCAAA
CTTTCTGAGGATAAGGTGAAAATATCTATTCCTGTAGTTCTTGTTCATCAAATACCGCTG
ATGTTTGAATAA
>CS28A_gene5 E833_7521_2#53_04439 ~~~gene5~~~CS28A outer membrane usher~~~
ATGTTTTTTATTGGTTCGCAGATGAATAAATATTATCTACTAATAATTCTTTTTTTTGTG
AGGAGCACAAAGGTTTTTGCCGAAGATTATTTTGACCCATCTCTTCTGGCAACCGATATT
ATTGGTGAAGGTAATATTGACCTTTCTGCATTCTCACGCCCTGGTGGAGGTATGGAGGGG
GAACAGGAGGTCGCTATTTATGTCAATGATGAGTTTTATTCCCGTAATACTCTGTTTTTT
AAAAATACTTTAGATAAAGGACTTTTACCTGAATTTACCCCGGGATTTTTTGACGAATTG
TTGTCGGGGGATTTTCTTGTGTCTGAAGAAGATAAAACAATATCATCATCAGACTTCCTG
AAAAAGGTTCCTTATAGTGATATAAACTTTAATCAAGGAATGTCTCGTGTCAATATCAGC
ATTCCTCAGGCATATCTTGGTGATGGAGCAAAGTTGATCTCCTCGCCTGATACATGGGAA
TATGGGGGGCCGGCATTTTTGCTAGATTATAATATTTCAGGCAACCGGAATGATTCAGGA
AATTATGATTCCCGTAGTCTGTATATCTCCTCACAGATGGGGGTAAATCTCATGAAGTGG
CGTTTGCGTACATCCTCCAGTTACAGCAATTATAAGACAAATTCTGTATGGGGAGGTGCT
CGTTCAGAGCAAAATAGCTTTTACAATACTTACGCAGAGAGGGATATTAGCTCGTTACGT
GCAATATTACGACTTGGTGAAGTTTCGACTGCAGGGTTAATACTGGATTCTGTTCCTTTC
CGAGGTGTGAAGCTGTCAAGTAGTGATGACATGCTGGGGATGCGTTTGCGTAATTACACA
CCCACAGTGCGAGGTATGGCGAACAGCCAAGCAGTTGTTACAATAACTCAGAATGGGCGA
CAGGTATACCAGACAAATGTACCAGCAGGGCCATTTGAACTGAATGATTTTTATCTTTCC
GGCTATTCGGGGGACATGCTGGTGACTGTCCGTGAGGCTGATGGTAGTGAGCACAGTTTC
TTGCAACCTTATTCGACCCTTCCGGAAATGAAACGCGAGGGAGTCTCCGGATTTGAGGTT
TCGGTTGGTCGCTATGATAATAACGGTGCTGAACACTATTACGATGCTGAGTCATTTGTT
TATGGTAACTGGTCTCGGGGTTTTGCTCGTGGAGTGACATTTTTTGCTGAGACTCTTCAG
GCAGAAAAATACCAGAGTCTGGGGGGAGGGAGTACGTTGTCGCTGGGAAGGCTGGGGGC
T
GCATCTGCAGATATATCCTTATCACGAGCAGATAAATACGGTGACATCCGGATTGGTCAG
TCTTATGGCTTTAAATACTCCAAGAGCCAGATTGAAACGGGCACAACGGTAACATTGGCA
ACATACCGATATTCCACTGAGAATTTTTATACTTTCCGTGATTTTGTTTCGAAAACGGAT
ACAGCTCGTTATATCTGGGAAAATAAACTGAAAAGCAGAATGACATTTAGCCTCAGTCAG
TCTCTGGGGGAGTATGGCTATTTGTCAGCCAATGCCAGTCAGCAGGATTATTGGAATAGC
CGAGAGGTGAGTCGAAATTACTCGCTGACTCATAGCTTTAGCTGGAATGATATTTATTTC

AGTACAACGTTGTCCATGGATGATCAACGAGGGCGGGAGACTGGACATTTATCCAATAAG
CAAGCAGGGATATATGCCAGTGTTCCTTTAAGTAAGCTTTTACCTAGAACTGATCCGACG
AGTAGTTCGCTAACTTGGAGTACTTCGCATGCTGATCATAAGGTACGCAATAGTGTGACT
CTGGACGGGAAAGTTCCAGAAAGTGACGTTCGTTATCGAGTGGGAGGCAGTTGGGGAAA
T
GGAACCACTGAAGGCTCACGTATGGCATCAGTGAGTTGGACTGGTGATCATGCAAGCACC
TCGCTGGGATATACCCGTGTAGGGAAATATCGAACCCTGGATTACAGTATGTCTGGCGCA
GCGGTGATGTATCCATGGGGGATTGCTGTGGGTAACAGTAGTGTTACTGGTGATGGTGCT
ATTGTAGTGGAAACACCAGGAGCTAAAGGAGTAAGAACTAGCACCGGATACAAAACTTCA
TGGTTGGGAACGGCTTTAATTAGTTCACCACAAAAATATACAGAGAATCGTATTAATTTA
TATCCCGACGGTCTTCCTAGTGACACCGTCCTTGGTGAAACATCTAAAACTGCCGTGCCT
GCTAAAGGTGCAGTCATTGTGCTGGATTATACTGTCTTTCGTGGTAGTCAGGTGGTGTTC
ACACTGAGACAGACTGATGGTAATCCATTACCGTTTGGTACAGTGATTACGCTTGATGGT
GTATCCAGAGGCAAGGAAAACAGTGGTATTGTAGGTGAGGAGGGCCGAGTGTATATGGCG
GGTATACCGGAAAAAGGAACTCTCACTGCATCATGGGGGCTGAATAAAACTTGTAGCATA
CCATTCCGTATAAACCAGCATAAAGCTGAGGCCGTTATCAGGGAGGTTCAGGGAGTATGC
CGTGTATGA
>CS28A_gene6 E833_7521_2#53_04440 ~~~gene6~~~CS28A chaperone~~~
ATGCCGTGTATGATGTTACAGATCTTCATTACAATTTTACTGTTACTGCCTTTATCAGTT
ACGAAAGCAGATATATTATATCCGTGGCCAAGTGATACTGAAGTCAGGCTGAAAATAAGT
GACGAAAAGGGTCAGAGCAGGGGAGATCTACGAGTTACTAATCCTGGCGATGCACTCTGG
TTGGTTCAGGCATGGGCTGAAGATGAAAAATATAGACGATATAGCGTTATATATCCATCC
GTATACAGATTGGAGCCATTTAGTGCTTATGCGCTGAATATTTACCCTGGTAATAATATT
ATGCCAGAACGGCTGAAATGGTTTTTGATTTCTTTTATACCTTCGATAGTGGAAAAAAAC
AAAAACCAACTAATTATACCAGTTACTTACAGACTCAAAATAATTGATGATGTGGTATGT
AAGGGTATTGAAAAAGTAGCTGGTGAGTGTTAA
>CS28A_gene7 E833_7521_2#53_04441 ~~~gene7~~~CS28A subunit~~~
ATGCTGAAGAAAATACCGTGCATAATATTAATGTCTTTACCTGGACTTATTTCTGCTGCG
GAAATAACAAAGCAGATAGAACTCACGCTAAAAGTTAATGTATTAAAGCCAGTATGTAAA
CTCAGTTCTGGGCAGCAAACAATAAACTTCGGTGATTTTGATGTGCTGGATGTTATTACG
AAAAGTAGTAAAGTGAATGGTAGCGCTACATTTAGATTTACTGAGTGTAGTGCTGTCAAT
AATATAAAAATAAAATTTAAGCAAGCCGGACAAAGCCCAGTTCCGGATATAGAAAACAAT
TATATCCCTAATGCTAAGGGGGATATAATGGCAAAGGGGGTGGCTGTAAAGCTTCTGGAT
GATCAAAAGAAAGAGGTCGAGTTGGACAAGATAATGAGCGTTAGTGTGGGGGAGAATCA
G
ATATCTAAAGATTTAACGTTAAATGCTCAGGTCATCTCTGTTAATAAAACTGGAGAAGGG
ATTTCGCCGGGAATGCTTCAAACGGCAATAGGGATGGAGATTTCATATGAGTGA
>CS28A_gene8 E833_7521_2#53_04442 ~~~gene8~~~CS28A minor subunit~~~
ATGTTGAAGTGTCTTTATTTTGGCTATTTTACCAAAAGTCTAATGCAACGCATTATTAAA
TTAACGTATGGGGCTGGAGAGTATGCTACCATTTTTACCGTTTTATTTTTTTCTGCGCAT
AGATGTGCAATAGCAGTTGAGAATAACTATACGATATCAATCGACAAGACATTTGATATA
TCAAGTATTACCAGAGACTGGATAGAGTTAGGTGAGTTTCCGTATCCAAAAGCAGCAAGT
AATAATGAGACCCATTTCCGTTGCACTAAGGGAGCAGGAAAAGGTTTATGCGCACATACA
GAGTTTAGAGTGAATGGAGGCTTACAGAGTAACCCCGCAGCATACTGGAATGGATGGATG
AGGATGGAGCCTAAAGAGGTCAATATAGGAGATGGGGAAAAGTTAATTTTTTCAGCATAC
TGGAAAGGAAGCCCAAGCATAAGATGGGAGGAGACAAATAGAAAAAATATGAAGAGTAT
T
TTTCATCAATACACTATTGGTGTTGCTTCTATTGGCTCATCGGCCGGCTCAATAACTAAA
TGGTCTTCAGGCGAACAAGGACCATCATTGAGGTGTGGGGATTTGGGCGGATGTACAATA
GATACACATACGTACTTTGATAACAGAAGTGGAGGAGCATTAAAGCTAGCGGTCAAGCTT
CCGGCTGCTTTCAGGAAAGGCACGTATACATTTTCTAATGTTGAAGTGCTTGATTTGGGG
CATACAGCGCGAAATGCTTCTGGCACAACACAGCAGAGTGTCAGTGCTAAAGTTTATATT
AGTGGGAAAATTACTGTGCCTGAGAGATGTTATATTGATACAGGATCAGGTTCTGAAATA
AAATTTAATGATGTAAGCGCAGGGGCAAGTAATGGGGAGGTTGATAAACGCAGTTTTACA
TTAAAAACAACATGCAAATATATTAATCTTAAACTCAATCAATATATAAAAGTAAATGGG
AGTAATGGTCTTTCCGAGTATGAGATTTTTTCCAGAGATAACTCAAATGAAAAAGCATTG
GCACTGGTGATGAGAATTGTTCGTGGTGACAATGGAGAGGACTTCTACGCAAATTGTGAA

CCTGACCATTCGGGTAGGGTATATTTAGTAAAGAGTATCTCTTGAGAGAAATAAATGGC
AATGGATTGAACATACATACTTACAACGATATAATAAGGTTTTCATTATGCAAATATGGA
ATACCAAAAGATTATGGAGAAAAAATCATTCCACTTACTATTGTTTCGAGATGGAGCGAT
TCCTGA
>CS28B_gene1 E1561_7521_3#30_04900 ~~~gene1~~~CS28B CfaD-like regulator~~~
ATGATAAGATTAAGAACTAACAAACTTGCGATATTATATACAGGTGAGCATGACATTTTG
GTCACAAACTTATCTGATAATACTCAGATACGGTGCAAAAGAAACTCTGTCGTAATTGTT
GGTAGAAATGTTAGAATTTCATTTATGGCAGATCAACTTCATGATCGCATATTACGAAAT
ACTGTGTTCTTTGACCATACTCAGATTATAACATTAAAAAAGATAATGAGTCTAGCTTAT
GGGTTTAGCAAAATTAATCCTCTTGACTTTGGGACTGACTCTCCTAAAATAAGGAAGATA
ATAAGCATCGAAGTAAATGACAGAATCAAAAACTCCTTTTACAATATAATTTCTGAGGAT
AGCCATCATAATAGAATATTATCATTTATATTCTTTTGTAAAGAATCTAGTGTAATGGAT
GCAATACTCCCTCTCATATACTTCTCAGCTGCAACAACGTTTTGTAATAGGGTGATAGAA
TTAATAGAATCAAACATAGCAAAGAAATGGACATTGAGATTATTGTCTGAGGAATTTAGC
ATCTCAGAAATAACGATAAGAAAAAAATTAGAAGCAGAAGGGGTTATCTTCAGAGAACTC
ATTCTTGAAATAAGGATGAAAAAGGCAATGACTCTTTTAATAGCAGGGGAGTTGTCTGTA
TCTAAGATAGCCGATAAGGTTGGCTACAATAATATATCATACTTCATATCCCATTTCAGA
CAATTTTTCGGAATGATGCCAAAACAACTTCATCTGTTATTAACAAAATAA
>CS28B_gene2 E1561_7521_3#30_04901 ~~~gene2~~~CS28B major subunit~~~
ATGAAAAAAATGATTATGCCTTTAACTATGGTGTCCGTTCTGATGAGCGGCTCTGCTCTG
GCTGCACCGGGGGCTAACGATTCCAGTCAGGCCACGCTGAATTTTAATGGTCGTGTGACA
TCCAGCCTTTGCCAGGTAAAAACAGATGATGTGGTAAAAGATATTTATTTGGGTGAGGTA
TCCAAATCCGCACTGGAAGCTAATGCTCAGGGGCCTAAACAGAGCTTCCAGGTTAACCTG
ATTAACTGTGATACCACCGTCAACGACATTTCCTATGTATTGAGTGATGCAAATGGTAAT
GGTACCGCCAATTATCTGATTCCAAAATCAGGTGATACTTCTGCAACCGGCGTAGGTGTT
TATGTGGAAAAGAGTGACGGTACTCCTGTAAATGTCGGAAATACGCAGACTCTGACGGTT
ACGAAAAAAGATGGTACTGATGCTCTTTCTGAACAAGTAATTCCTCTGCGTGCCTATATT
GGTGCTCAGGGTGGGGCAGGTGGCGTATCTGCTGTTACCGCAGGTACGGTTGATGCAACT
GGTATTTTGACCATTCGCGCAACTAAACCGTAA
>CS28B_gene3 E1561_7521_3#30_04902 ~~~gene3~~~CS28B chaperone~~~
ATGCGTTATCAGTATTCTGTTTTATTCGTCTTTTTATTCTCTGGCGTTGTACAGGCGGCG
GAGTTTTTCGGTCCGCGGGAAAACCGGTTGCTTTTTGAAGAGAAGAAAGGAAACACCTTT
TACCGGATTGACAATTCAGACAAAAAACTTCCCTGGCTTGTACAGGCATGGATTGAAGAT
GCGTCTGAAAAGAAAACAACCGCACTGACAGCCACGCCCATGGTGTTTCGCGTTGAGCCT
TCTTCTGTATTTACTGTCAGGGTGGTTAAAACGGGAGCCCTGCCGGAAGACCGGGAAACA
CTTTTCTGGGCCGTTTCCAATTCATTACCGGGAGGTGCCTCAACAAAGCAGGATAATGAA
GAGGGAAAAATCAGCGCTAAAATCAGCCTGGCTTACCGGTTTAAGGTGCCACTTATTTAC
CGGCCTGCAGCGTTGGATAATTTCAGGCAGGAGCCGGAGAAACTGGAATGGACATATAAT
GGCAAGGAAGGGCTGAAATTATATAACCCTACCCGTTATGTGGTCCAGTTACATAACGTG
ACGGCGAACGGACGTGAATTTAAGGGCAAAGGGGTTTCTTTTATTCTTCTTCCGATGTCC
GGTAAAAATGTGAGTGCCGCTGTGAATAAAGGCACCAGAATAAAATATGGTGTCATAAAT
GACTATGGCGCGGTAAAAGAATATGATGGTGTTGTTAAGTAA
>CS28B_gene4 E1561_7521_3#30_04903 ~~~gene4~~~CS28B chaperone~~~
ATGATGGTGTTGTTAAGTAATGCTCTAAAAAAAGCAGTGCTTTCCCTGTTGGCGTCCTGT
AGCATAGTTGTTTTTGCATCTGAATCAGAACTTGGCGTTGCTGTAGAACCGATGAGGTTA
TCAATTAAGCCTGGAGAGATGACATATTTTACGGTTATCAATGAGACAGAGAGAGAGTAC
ATTGTCACCACCAGGGTAGTAAGTGACTCAGGGATTGATGATAAAGATAAAGTATTTGTC
TTTAGTCCCCCCCTGAAGCATTTAAAAAAAAAGGGAACAGTCTGTAATGGGAGTTGTATAT
CTGAAAAATGGGAGAGAGAATAAGATGAAATACTATTTATCTGTCTCATTTGTCCCCAAA
CTTTCTGAGGATAAGGTGAAAATATCTATTCCTGTAGTTCTTGTTCATCAAATACCGCTG
ATGTTTGAATAA
>CS28B_gene5 E1561_7521_3#30_04904 ~~~gene5~~~CS28B outer membrane usher~~~
ATGAATAAATATTATCTACTAATAATTCTTTTTTTTGTGAGGAGCACAAAGGTTTTTGCC
GAAGATTATTTTGACCCATCTCTTCTGGCAACCGATATTATTGGTGAAGGTAATATTGAC
CTTTCTGCATTCTCACGCCCTGGTGGAGGTATGGAGGGGGAACAGGAGGTCGCTATTTAT
GTCAATGATGAGTTTTATTCCCGTAATACTCTGTTTTTTAAAAATACTTTAGATAAAGGA
CTTTTACCTGAATTTACCCCGGGATTTTTTGACGAATTGTTGTCGGGGGATTTTCTTGTG

TCTGAAGAAGATAAAACAATATCATCATCAGACTTCCTGAAAAAGGTTCCTTATAGTGAT
ATAAACTTTAATCAAGGAATGTCTCGTGTCAATATCAGCATTCCTCAGGCATATCTTGGT
GATGGAGCAAAGTTGATCTCCTCGCCTGATACATGGGAATATGGGGGGCCGGCATTTTTG
CTAGATTATAATATTTCAGGCAACCGGAATTATTCAGGAAATTATGATTCCCGTAGTCTG
TATATCTCCTCACAGATGGGGGTAAATCTCATGAAGTGGCGTTTGCGTACATCCTCCAGT
TACAGCAATTATAAGACAAATTCTGTATGGGGAGGTGCTCGTTCAGAGCAAAATAGCTTT
TACAATACTTACGCAGAGAGGGATATTAGCTCGTTACGTGCAATATTACGACTTGGTGAA
GTTTCGACTGCAGGGTTAATACTGGATTCTGTTCCTTTCCGAGGTGTGAAGCTGTCAAGT
AGTGATGACATGCTGGGGATGCGTTTGCGTAATTACACACCCACAGTGCGAGGTATGGCG
AGCAGTCAAGCAGTTGTTACAATAACTCAGAATGGGCGACAGGTATACCAGACAAATGTA
CCAGCAGGGCCATTTGAACTGAATGATTTTTATCTTTCCGGCTATTCGGGGGACATGCTG
GTGACTGTCCGTGAGGCTGATGGTAGTGAGCACAGTTTCTTGCAACCTTATTCGACCCTT
CCGGAAATGAAACGCGAGGGAGTCTCCGGATTTGAGGTTTCGGTTGGTCGCTATGATAAT
AACGGTGCTGAACACTATTACGATGCTGAGTCATTTGTTTATGGTAACTGGTCTCGGGGT
TTTGCTCGTGGAGTGACATTTTTTGCTGAGACTCTTCAGGCAGAAAAATACCAGAGTCTG
GGGGGAGGGAGTACGTTGTCGCTGGGGAGGCTGGGGGCTGCATCTGCAGATATATCCTTA
TCACGAGCAGATAAATACGGTGACATCCGGATTGGTCAGTCTTATGGCTTTAAATACTCC
AAGAGCCAGATTGAAACGGGCACAACGGTAACATTGGCAACATACCGATATTCCACTGAG
AATTTTTATACTTTCCGTGATTTTGTTTCGAAAACGGATACAGCTCGTTATATCTGGGAA
AATAAACTGAAAAGCAGAATGACATTTAGCCTCAGTCAGTCTCTGGGGGAGTATGGCTAT
TTGTCAGCCAATGCCAGTCAGCAGGATTATTGGAATAGCCGAGAGGTGAGTCGAAATTAC
TCGCTGACTCATAGCTTTAGCTGGAATGATATTTATTTCAGTACAACGTTGTCCATGGAT
GATCAACGAGGGCGGGAGACTGGACATTTATCCAATAAGCAAGCAGGGATATATGCCAGT
GTTCCTTTAAGTAAGCTTTTACCTAGAACTGATCCGACGAGTAGTTCGCTAACTTGGAGT
ACTTCGCATGCTGATCATAAGGTACGCAATAGTGTGACTCTGGACGGGAAAGTTCCAGAA
AGTGACGTTCGTTATCGAGTGGGAGGCAGTTGGGGAAATGGAACCACTGAAGGCTCACG
T
ATGGCATCAGTGAGTTGGACTGGTGATCATGCAAGCACCTCGCTGGGATATACCCGTGTA
GGGAAATATCGAACCCTGGATTACAGTATGTCTGGCGCAGCGGTGATGTATCCATGGGGG
ATTGCTGTGGGTAACAGTAGTGTTACTGGTGATGGTGCTATTGTAGTGGAAACACCAGGA
GCTAAAGGAGTAAGAACTAGCACCGGATACAAAACTTCATGGTTGGGAACGGCTTTAATT
AGTTCACCACAAAAATATACAGAGAATCGTATTAATTTATATCCCGACGGTCTTCCTAGT
GACACCGTCCTTGGTGAAACATCTAAAACTGCCGTGCCTGCTAAAGGTGCAGTCGTTGTG
CTGGATTATACTGTCTTTCGTGGTAGTCAGGTGGTGTTCACACTGAGACAGACTGATGGT
AATCCATTACCGTTTGGTACAGTGATTACGCTTGATGGTGTATCCAGAGGCAAGGAAAAC
AGTGGTATTGTAGGTGAGGAGGGCCGAGTGTATATGGCGGGTATACCGGAAAAAGGAACT
CTCACTGCATCATGGGGGCTGAATAAAACTTGTAGCATACCATTCCGTATAAACCAGCAT
AAAGCTGAGGCCGTTATCAGGGAGGTTCAGGGAGTATGCCGTGTATGA
>CS28B_gene6 E1561_7521_3#30_04905 ~~~gene6~~~CS28B chaperone~~~
ATGCCGTGTATGATGTTACAGATCTTCATTACAATTTTACTGTTACTGCCTTTATCAGTT
ACGAAAGCAGATATATTATATCCGTGGCCAAGTGATACTGAAGTCAGGCTGAAAATAAGT
GACGAAAAGGGTCAGAGCAGGGGAGATCTACGAGTTACTAATCCTGGCGATGCACTCTGG
TTGGTTCAGGCATGGGCTGAAGATGAAAAATATAGACGATATAGCGTTATATATCCATCC
GTATACAGATTGGAGCCATTTAGTGCTTATGCGCTGAATATTTACCCTGGTAATAATATT
ATGCCAGAACGGCTGAAATGGTTTTTGATTTCTTTTATACCTTCGATAGTGGAAAAAAAC
AAAAACCAACTAATTATACCAGTTACTTACAGACTCAAAATAATTGATGATGTGGTATGT
AAGGGTATTGAAAAAGTAGCTGGTGAGTGTTAA
>CS28B_gene7 E1561_7521_3#30_04906 ~~~gene7~~~CS28B subunit~~~
ATGCTGAAGAAAATACCGTGCATAATATTAATGTCTTTACCTGGACTTATTTCTGCTGCG
GAAATAACAAAGCAGATAGAACTCACGCTAAAAGTTAATGTATTAAAGCCAGTATGTAAA
CTTAGTTCTGGGCAGCAAACAATAAACTTCGGTGATTTTGATGTGCTGGATGTTATTACG
AAAAGTAGTAAAGTGAATGGTAGCGCTACATTTAGATTTACTGAGTGTAGTGCTGTCAAT
AATATAAAAATAAAATTTAAGCAAGCCGGACAAAGCCCAGTTCCGGATATAGAAAACAAT
TATATCCCTAATGCTAAGGGGGATATAATGGCAAAGGGGGTGGCTGTAAAGCTTCTGAAT
GATCAAAAGAAAGAGGTCGAGTTGGACAAGATAATGAGCGTTAGTGTGGGGGAGAATCA
G
ATATCTAAAGATTTAACGTTAAATGCTCAGGTCATCTCTGTTAATAAAACTGGAGAAGGG

ATTTCGCCGGGAATGCTTCAAACGGCAATAGGGATGGAGATTTCATATGAGTGA
>CS28B_gene8 E1561_7521_3#30_04907 ~~~gene8~~~CS28B minor subunit~~~
ATGTTGAAGTGTCTTTGTTTTGGCTATTTTACCAAAAGTCTAATGCAACGCATTATTAAA
TTAACGTATGGGGCTGGAGAGTATGCTACCATTTTTACCGTTTTATTTTTTTCTGCGCAT
AGATGTGCAATAGCAGTTGAGAATAACTATACGATATCAATCGACAAGACATTTGATATA
TCAAGTATTACCAGAGACTGGATAGAGTTAGGTGAGTTTCCGTATCCAAAAGCAGCAAGT
AATAATGAGACCCATTTCCGTTGCACTAAGGGAGCAGGAAAAGGTTTATGCGCACATACA
GAGTTTAGAGTGAATGGAGGCTTACAGAGTAACCCCGCAGCATACTGGAATGGATGGATG
AGGATGGAGCCTAAAGAGGTCAATATAGGAGATGGGGAAAAGTTAATTTTTTCAGCATAC
TGGAAAGGAAGCCCAAGCATAAGATGGGAGGAGACAAATAGAAAAAATATGAAGAGTTT
T
TTTCATCAATACACTATTGGTGTTGCTTCTATTGGCTCATCGGCCGGCTCAATAACTAAA
TGGTCTTCAGGCGAACAAGGACCATCATTGAGGTGTGGGGATTTGGGCGGATGTACAATA
GATACACATACGTACTTTGATAACAGAAGTGGAGGAGCATTAAAGCTAGCGGTCAAGCTT
CCGGCTGCTTTCAGGAAAGGCACGTATACATTTTCTAATGTTGAAGTGCTTGATTTGGGG
CATACAGCGCGAAATGCTTCTGGCACAACACAGCAGAGTGTCAGTGCTAAAGTTTATATT
AGTGGGAAAATTACTGTGCCTGAGAGATGTTATATTGATACAGGATCAGGTTCTGAAATA
AAATTTAATGATGTAAGCGCAGGGGCAAGTAATGGGGAGATTGATAAACGCAGTTTTACA
TTAAAAACAACATGCAAATATATTAATCTTAAACTCAATCAATATATAAAAGTAAATGGG
AGTAATGGTCTTTCCGAGTATGAGATTTTTTCCAGAGATAACTCAAATGAAAAAGCATTG
GCACTGGTGATGAGAATTGTTCGTGGTGACAATGGAGAGGACTTCTACGCAAATTGTGAA
CCTGACCATTCGGGTAGGGTATATTTAGTAAAGAGTATCTCTTGAGAGAAATAAATGGC
AATGGATTGAACATACATACTTACAACGATATAATAAGGTTTTCATTATGCAAATATGGA
ATACCAAAAGATTATGGAGAAAAAATCATTCCACTTACTATTGTTTCGAGATGGAGCGAT
TCCTGA
>CS30_csmS NZ_LT174529.1 ~~~csmS~~~CS30 CsmS transcriptional regulator~~~
ATGAAGCGCAAGTACCTTTCAGAAAAAGAGGTGCATTGTTTACTTAAAGCGGCTGCGGAA
AATCAATACTCAGTTCGTGATTACTGCATGATAAGTATGGCGTTTATTCATGGATTAAGA
GTGAGTGAATTAGTAAACCTCAAAGTAGATGACTACGACTCTCTATCTGCTCAATTAAAC
ATCAAAAGACTTAAAAATGGATTCTGTACGATTCAACCATTATTACCTGATGAAAATGAA
TTACTTCAATGTTGGCTAGATGAACGTAAAACATGGGATGGACATGAAAGCTGTTGGTTA
TTTCTGTCTAAAAATGGTGGACCTCTAAGTCGCCAACGCTTTTGGCAATTATTACGTAAG
TATGGAGATGAAGCACATCTTACCATAAAGGTTCACCCACATATGTTAAGACATGCTTGC
GGATTTAATCTTGCAGAACGCGGCAATGACACGAGATTAATACAGGATTACTTAGGACAT
AGAAACATAAGGCACACAGTGCATTACACTGCCAGTAATCCCGAACGTTTTAGAAACGCA
TGGACAAAGAATAACTTTCATAGTTTCTCATGCACTCATGCACTCATGCACTCATGCATT
TAA
>CS30_csmT NZ_LT174529.1 ~~~csmT~~~CS30 CsmT transcriptional regulator~~~
ATGACTAAGCGAAAATACCTGACCCAAAGTGAGGTGGAGCTAATGCTTACTGAAGCAAAA
CATGGACAATTCTCAGAGAGGAATTATTGCTTGCTTTATCTTAGTTTCATTCATGGGTTT
CGCGTCAGTGAAGTATGTAATCTAAGGCTTGAAGATGTATGCCTGCGTGATAAAAGCTTA
AATATTCACCGAATGAAGAATGGGTTTAGCACAATTCATCCACTTCTATCCGATGAAGTA
AAAGTGCTTAAAGCATGGTTATGTGTGAGGGATAAAATGAATGGCTCTGAAAGCGAGTGG
TTATTTGTATCGCGGCATGGGGGGGCTTTAAGCCGGCAGCGTGTCTGGCAAATGATTCAA
AATCTTGGGAAGAAGGCCGGTGTAAAAGTGAACTCTCATCCACACATGCTAAGACATGCA
TGTGGCTATGCTTTGGCCGATAGGGGTGTTGATACTCGTCTAATACAAGATTATTTGGGG
CATAGAAACATACGACATACAGTTTGTTATACCGCAAGTAATGCTGAGCGTTTTAAGTAT
ATTTGGTTAAAAAAAGGTCTAAAGGTTAACAATTAG
>CS30_csmA NZ_LT174529.1 ~~~csmA~~~CS30 CsmA major subunit~~~
ATGAAAAAGACAATTATGTCTCTGGCTGTAGTTTCAGCTTTAATGAATGGTGTTGCATTT
GCTGCTCCGGCCGATAACGATGCAAGTAAGGCTACTCTTAACTTTTCAGGGCGTGTGACT
TCTAGCCTTTGCCAGGTAAAAACTGATGATTTAACTAAGGACATCTCTCTTGGTGAGGTT
TCTAAGTCAGCTCTTGCAGCCAGTGGTAAAGGCCCAGCTCAGAGTTTCCAGGTTAATTTG
ATTAACTGTGACACCACCACAAACGATATTTCTTACGTACTTGCAGATGCGAATGGCAAT
GGCAATGGTGCCAATGGTGCCTCTACCTATCTGGTTCCTAAATCGGGTGATACCGCAGCA
GAAGGTGTGGGTGTGTTTGTTGAAACCAGCAATGGTACCAAGGTTAATATTGGTACAGCA
CAAACACTGAACGTTGTATCTAATGGCGCTACCGCACTTTCAGAGCAGGTCATTCCACTG

CGTGCCTACATTGGTACTCAAAATGGCACCGGCGGAACTATCGGTACTAACGGTCTTAAA
GCTGGTACTGTAGATGCAACTGGCGTACTGACAATTCGTGCAAATTACAAAGCAAACACT
CCGTAA
>CS30_csmB NZ_LT174529.1 ~~~csmB~~~CS30 CsmB chaperone~~~
ATGATTCGCTTAGCTATTAGTTTTTACTTATATTAATGATGGCATCAACTACCGCTGTA
GCTCAAGGAACATTTGGTCCAAGAGAAAATAAACTTATTTATGATGGCAGCAAAGGCTAT
ATACAGTATCGCATTGATAATACAGATAATAAAACGCCTTGGTTGGTGCAGGCTTGGGTG
GAAGATTATAAAGAAAATAAAATAAAAGACTTTACACCGACACCCTTTGTTTTCAGGGTA
GAGCCCTCATCCGTGTTCTCTGTTCGGGTGATGAAGACTGGAACTACTGATGAATTCAAG
GAGACTTTATTTTGGCTTGTTTCAAACACGATTCCCGGCGGTGCAAAGTCAAAGCAGCAA
AATGAAGATGATAAAATAACAGCGAAGTTAAGTTTAGCATATCGTTTTAAAGTACCTATG
ATTTACCGCCCGACTTCATTGAAGAATATTCAGCAAAAACCCGAAAGCCTTGAATGGTCA
CTGGATGAAAAGGGAAAACTCAAAGTAAAAAATCCGAGCAGATATGTTGTACAACTACAT
AGTGTAACAATCAATGGTACGATTCATCAGGGAAGTGGTGTTTCATATTTTATTTTGCCT
ATGAGCGGTGTTGAGTTTAACTTTAATGCTAAAAGTGGTTCTAGGGTAGATTATGCTGTA
ATTAACGACTATGGTGCACTGAACAAATATGAGGGTGTTATTAAGTAG
>CS30_csmC NZ_LT174529.1 ~~~csmC~~~CS30 CsmC chaperone~~~
ATGAGGGTGTTATTAAGTAGTTTAGTTGCTTTTTTAACATTAATTATTCCTACTAGTGTA
GATGCAAATGTGCAAGAGTTAGGTGTTGCTGTCGATCCACTAAAAATAAAAGTGGAGCCA
AACCATATGGTTTATTTTACTGTGAGTAATGATACAGAAAATGATTATATAGTGACTGCA
AAAACAACAAGCACCTTAACAATAAGAGATGCACCAGAAAAAGAATTTTTTTAGTTAAC
CCACCAATACGGTTACTAAAGAAAAGAAGCCGAGCGCAAATGGGGGTTGTATATTTACCG
GATCGACATCATACCTCACCTGGCTCTAAATACTACCTTTCCATTTCATTTATCCCTAAA
ATTTCTAAAGATACAAAACTGGCTCACATACCAATTATTTTAGTGCAACAAGTACCATTG
GTTATTAAATAA
>CS30_csmD NZ_LT174529.1 ~~~csmD~~~CS30 CsmD outer membrane usher~~~
ATGAGCAAACATCACTTTATTATTGCTCCTTTTATTTTAGGTATTAGTGCAAACATTTCT
GCCGAGGACTATTTCGATCCATCTCTTTTAGCTTCTGATGTTGCTGGGGGGAGTGATGTA
GATCTATCTGCATTCTCTCATCCGGGGGGAGGCATGGAAGGTGAGCAAGAAGTAAGTATT
TATATAAATAATGATTTTTACACAAGAGAAACTTTGACGTTCAGAAATACAAGTGATCGA
GGATTGTTACCAGATTTCCCCCCTAGGTTTTTTGGGCCACTTTTGGTGGAGGATTACTCT
TCGCTAGGGAAAAATAAAACTCTTTCATCATATGATTTTTTAGCTAGCATTCCATATAGT
GAGATAACCTTTGATCAAGGAGCATCCAGAGTGAATATTAGTATTCCTCAAGCATACTTG
GGAGGGGGTGCTAAATTAATTTCTTCTCCTGAGACATGGAACTACGGAGTTCCTGCTTTT
TTACTGGATTATAATATATCAGGTAATCGCAACAAAGCCAGCGATTATGATGCAGAAAGT
TTTTATGCATCATCGTTGATAGGTGTTAATTTTTCGAAGTGGCATTTACGTACATCAGCG
AACTACAGCCAATACAAGAACAACTCATCATGGGGAGGTGTAAGTACAGATAAAAGTAGT
TTTTATAACACATATGCTGAACGGGATATTAGTTCTTTGCGTGCTTCATTGCGCCTTGGT
GAGGCATCAACCGGTGGTATTATTCTTGACTCGATACCATTCCGTGGATTAAGACTTTAC
AGTGACGATGATATGTTAGGCTATCGTCTCAGGAATTACACGCCAACTGTACGAGGCATT
GCTCGTAGTCAATCTGTTGTAACTATAACACAAAATGGTAGGCAGGTATATCAAGCAAAT
ATACCACCAGGACCTTTTCAAATTGAAGATTTAATCTCTCCGGTTATACCGGTGATATG
GTCGTGACTATTCGAGAGGCTGACGGGAGTGAACATAGTTTTCTCCAGCCATTTTCGACT
CTTCCGGAAATGAAACGCGAAGGGATTTCAGATTTTGAGTTATCAATAGGGCAGTATGAT
AACAATGGATTTGACAGTCATTATGAAAAACCATCTTTTGTTTATGGAAGCTGGTCACGT
GGTTTTTCATATGGCATTACAACATTTGGCGAGACAGTGCAGTCAGATAAATATCAAAGT
ATTGGACTTGGAAGCACTGTCTCTTTAGGAACTTTTGGTGCTGCATCAGCCGATGTATCA
TTTTCTCGAGCTGATAAATTCCGTGATATAAAAACAGGTCAATCTTATGGTTTGAAATAT
TCTAAAAGTCAACTAGAGACTGGCACCACTCTTACATTAGCTACGTATCGTTATTCTACT
AAAGATTTTTATACATTTAGAGATTTTACATCAAAGATTGAGAGTTCACGCTATACATGG
GATAACAGATTGAAAAACAGGATGACGTTTAACCTAGCCCAATCTTTAGGTAAATACGGA
AATATATCACTTAGTGCTACGAGACAGAGTTATTGGACCAGTAGTGAACTGACACGCAAC
TATAACTTGTCACATAGCTTTAGTTGGCAAGATATTTATTTTAACACGACCTTATCGCTA
GATCAGATGCACAATCGCTACAAAGATCGATCTGAGAATAAACAGATCGATCTTTATGTA
AGTGTACCTTTTTCTAAATTTTTAGGCAATAATGACATTACTTCAAGTTCGTTAACATGG
AACATGACAAACTCGGACCATCGAGTTCGTAATAATGCAACTTTAAATGGAAGTATACCT
GATACGGATTTTCGATATCGTGTTGGTGGCAGTTGGGGGAACGATAACGTCGCGACTACC

AAGACAGCTTCTCTTAGCTGGACAGGAAACTATACCAGTGCATCTTTAGGCTATACTTAT
TCTAATGAATCTAATACTATAGATTATAGTTTGTCAGGTTCTGCAGTTGCTTATCCTTGG
GGGCTTGCATTAGGTAATAGTAGTGTAACTAACAGTGGTGCTATTGTTGTTGAGACGGCT
GGAACCTCGGGTGTCAAAACTAGCGCTGGATATAAGACCTCCTTGTTAGGTACGGCACTA
ATTGGTTCGCCTCAAAAATACACTGAAAATAGAGTTGACCTTTATCCTGATGGACTTCCG
GATGATACCGTGTTAGCTGAAACATCAAAAGTTGCTGTGCCTGCAAAAGGAGCTGTTGTT
GTATTAGATTACACAGTTTTTAAAGGAAGCCAAGTTGTTTTTACTTTGACGCAGCCAAGC
GGAAAACCGTTACCTTTTGGAGCGATTGTTTCTCTCGATGGAATGCCAAAGGGAAAAGAG
AATACAGGAATAGTCGGCGAGAATGGACGAGTTTATATGGCAGGTATTCCTAAAGAAGGC
TCATTAAAGGCTACCTGGGGGGGAAACATTTGCAGCATAAAATTTCGTATAGAAGATCAA
AAATCAGTGGGCCCAATCAGAGAAACTACTGAGGTTTGCAAAGCATGA
>CS30_csmE NZ_LT174529.1 ~~~csmE~~~CS30 CsmE chaperone~~~
ATGAAATGTTTATTAATACTGTTATTGATAATTTTGTCCTTTAACAACAAAGGGGTACTA
GCTAATGTAGACTATCCTTTCCCTGAGGATACTATCATTAATCTGAATCATGACAAAAAG
GATTTATATCTGAAAGGTAGTATTCGTATATCAAACCCAGGAAGTTTGGTTTGGTTGGTC
CAGACATGGACTGAGGATGAGAACAAAAGTCGATACGCTGATGTTTATCCTGCTCTAATG
AGGCTTGAACCATACTCTAGCAAAGTACTAAAAGTTTACCCGAAAAGCACTCCTGATAGA
AAAGAATTGAAATGGTTGCTAATATCTTTCATTCCATCTCAGGATAAAATTGGTCATAAT
CAATTGACTATACCAGTATCTTACAGATTGAAAATAATTAATAAACACTAA
>CS30_csmF NZ_LT174529.1 ~~~csmF~~~CS30 CsmF minor subunit~~~
ATGATTAATAAATTAATATTTCTTTTAACCTTAGTTTCCTTCGCAAACTGTTCGTTTGCC
GAACAAATCAAACAAGAAGCAACTTTAAGTTTAAATGTTAACATTCTCAAACCTGTTTGT
AAGTTAGCGAACGGGGATCAAACTTTATACTTTGATGATTTTGATGCTTTGGATGTAGTT
ACTAACAGTAATAAGCTAATTAAGAATACAGTTCTTAATTTTACTGAATGTAGTAGTGTG
AAAAAACTTAATATTTCATTTGTTCAGTCAGGACAAACCCCCCCTATTGATACAGTTAAT
AACTGGATACCTAACAAAAGCGGAACAGATATGGCAGCTGGTATCGCTATTATTTTGCTC
AATAACGATCACTCATTGATAAATCTAGGGCAAAAGATGGTCATTGACGTAGGACAGTCT
GAAAGCTCAAAACAAATTACTCTAAAAGCGCAAGTCGTCCCGACAGATAAAGCTGGTAGT
GTAATAAAACCCGGAAAACTGGAGACAGCAGTTGGCATTGAGATTTCATATGAGTAA
>CS30_csmG NZ_LT174529.1 ~~~csmG~~~CS30 CsmG adhesin~~~
ATGAAAAAAAAACTAAAATCCAAAAAAATTTGGATCTATTTCGCGTTGCTTGCAGCCCCT
AGTCATAGCTATGCTAATGACGGCACAGGAGAGGCTTTTACCAAAGATATGACATTCACT
AAGAATATTGAAGTTCCCACTTATAATTTCCCCGGCAGCTGGATTGAGCTTGGTAACTTG
GGAATAAGCAAGTATTCACAGGTTGATGGAAATCATCGTTGCTCAAATCTAACTTTGTGT
GGATATACTGCTGTTAGGGTAAATGGTGGTACAAGTGGTAATAAGGGGGCGTATTATCGC
ATGAAACTCGAAGCCTCCCCAGTTACAGTTTCCTCAAATGGTATAAATTTTGTTTTTTCC
GTTTATTTTAAAGGATCTCCATATTTAATATGGCAAGAAAGAAATCTCAATAATAACAGA
AATTGGTTTCATACTATTTATGTGCCTGTAAATAGTATTGGAAGTAGTGCAGGGAGTACT
ACCAACTATTCTAAAAATTTAGATCCGTCAGGTGTTTGTGGTTCTTTAAGTGGATGTACA
TATGGAGCCACGACATATTTTACTTCGGGAGGAGATGCTTATCTAGCATTAAAGGTACCT
GAAAATTTAGCTGCTGGCACTTATCAATTCAGTGATGTTGAAGTTTTAAGCCTGTGGCAA
CAGTCAAATAATGCTACATGGTTAAACCGTTATGAGGCCAAAGCTACGGTTAAAATTAGT
GGAACCATTAAGTTACCTAACAGGTGCTATTTCTCAAGCTCACAAAATAACATCAACTTT
AGTGATGTTAAAATAAATTCAAACAATGGTAGCTTAGAGACTAAAGATTTTAAATTGCGT
ACTATCTGTCAAGGAATTCAGGTGGATGTAAAACAATATTTAACTGTCAGTTCAGATGTG
AACGATTATATAAAAGTATTTTCCTATGATGACGAGGGGAATAAAGCTTTAGGTTTTGCC
ATGCAAATTGCTCAGCAGGGATTATCCTCAAAAGAACCTGATTGTGATGCAAGAAGTGAA
TCTTTGAACAAATTTAATAGCGAATATCTTATAAGAACAATACCGGCTTCATCTTATCAA
GCGTTTGAAGATACTGTTAAATTTTCATTATGTAAGTTTAGTGTTCCAGCATCAAAATAT
ATTGGAGCACATAATATACCGATAAAAATCATTTCCCGATGGGAAAGCTGA
>CS31A_clpE L05180.1 ~~~clpE~~~CS31A ClpE chaperone~~~
GTGAGTAAGCGTAACGCAGTAACGACGTTTTTCACTAACCGGGTGACAAAAGCACTGGGA
ATGACGCTGGCGCTGATGATGACCTGTCAGAGTGCTATGGCTTCCCTAGCGGCAGATCAG
ACCCGCTATATCTTTCGAGGGGACAAGGATGCGCTGACCATCACGGTCACCAACAATGAC
AAGGAGCGTACCTTTGGTGGTCAGGCCTGGGTGGACAATATCGTGGAGAAGGACACCCGT
CCGACTTTTGTGGTGACACCGTCCTTCTTCAAGGTGAAGCCGAATGGTCAGCAGACACTG
CGTATCATCATGGCCTCGGACCATCTGCCGAAGGATAAAGAGTCGGTGTACTGGCTGAAC

CTGCAGGATATTCCGCCGGCTCTGGAGGGCAGCGGTATTGCAGTGGCCGTGCGCACGAAG
CTGAAACTGTTCTATCGCCCGAAGGCACTGATTGAGGGGCGCAAAGGGGCAGAAGAAGG
C
ATCAGCCTGCAGAGCCGCCCGGATGGCAGGACCATGCTGGTGAACACCACGCCGTACATT
TTTGCGATTGGCAGCCTGCTGGACGGAAACGGGAAGAGAATTGCCACGGATAACGAGAC
A
GCACAGAAACTGCTGATGTTCATGCCGGGGGATGAAGTACAGGTGAAGGGAAATGTGGT
G
AAAGTGGATTCTCTGAATGATTACGGTGAACTGCAGACCTGGACGATTAATCAGAAAAAA
ACGCCAACGTCTTCCGGGCAGAAGGCATCTGATTCGCTGGTTAATCCGTCTGATAAGGCA
GATAAAAAATAA
>CS31A_clpF M96174.1 ~~~clpF~~~CS31A ClpF minor subunit~~~
ATGAAGAAAACAATGATGGCAGTCGCCCTGGCCCTGAGTGCACTCAGTATTCAGTCAGCA
CAGGCCGCTGAATACAGGGAGAAAACTCAGTATCTGGGGGTGGTGAACGGTCAGGTGGT
G
GGTAACAGTGTGATGAAGGTGACCCGTACACCAACAGACCCGGTGCTGTACCGGAGTGG
A
GACACGACGCCTCTTCCCGACAACCTGACTGTCCGCAACGCAGAGTCCAGGGAGGCATC
A
GGCGGTCTGGCGTATATCACGGTGAAGCAGGTGCTGCCGGATAACGGGGAAGCCCGTATC
ACCCTGAAGACAGCCCTGATGGTTGACGGAAAGAAAGTGGCACTCAGTGCCAGACAGCA
G
GGTGAGGATGTGGTGATAAGCGTGCCGGAGGCAAAGAAACAGGTTGAGTTAAGAACGGA
T
GCACCGGCAGAGCTGGAAGTCCCGGTCAGTTACCGGGGAAATCTGCAGATAGCACTGCA
G
GTGGAAGACTGA
>CS31A_clpG M55389.1 ~~~clpG~~~CS31A ClpG fimbrial subunit~~~
ATGAAAAAGACTCTGATTGCACTGGCTGTGGCTGTTTCGGCAGTATCAGGTGCGGCGCAT
GCGTGGACCACTGGTGATTTTAATGGTTCATTTGATATGAATGGCACAATTACTGCTGAT
GCGTATAAAGACAAATGGGAATGGATGGTTGGGGGCGCTCTCTCCTTCAACAACACTATC
AAGGAAATGACAGGTGACAGTAAGCTGCTGACCATCACTCAGTCTGAACCAGCTCCTATT
CTTTTAGGGCGCACAAAAGAGGCGTTTGCAGCATCGATTGTTGGTGTTGGTGCAATTCCT
TTAATTGCGTTCAGTGATTATGAAGGGAACGGAGTTGCCTTACAGAGTTCTGGGGATAAC
GGTAAGGGGTTCTTTGAATTGCCCATGAAAGATGATAGTGGAAATAATCTCGGTAGCGTA
AAAGTTAATGTTACTTCTGCTGGCCTGTTTTCCTATAGTGAAATATCAACAGGTTTAGTT
GGTATAACTTCTGTTGCCAGTGGCGATAATACAAGTATTTATTATGGTGGTCTGGTGTCG
CCAGCAATTAGGGCGGGTAAAGACGCAGCATCAGCTGTGTCGAAATTTGGCAACTATAAT
CATACACAATTGCTGGGCCAGCTTCAAGCAGTAAACCCTAACGCGGGCAATAGAGGACAA
GTAAATAAAAATAGTGCGGTCTCACAAAATATGGTGATGACTACTGGTGATGTAATTGCA
TCCTCTTACGCACTTGGTATTGACCAGGGACAGACTATTGAAGCAACCTTTACTAATCCT
GTGGTTAGCACCACCCAGTGGAGTGCTCCGCTGAACGTGGCAGTAACTTATAACTAA
>CS31A_clpL L05182.1 ~~~clpL~~~CS31A ClpL minor subunit~~~
GTGAAAAAGGTGACGTTGTTTCTGTTTGCTGCCAGCCTCCTGCCTTCCTGTGTACTGGCC
TGGAACACGCCGGGAGAGGATTTCAGCGGAGAGCTTAAGCTGGAAGGCCCGGTGACCAG
C
ACCCGTAACCCGTGGGTATGGAAAGTCGGACAGGGAAATGAAGGTCTGGAGGTTAAACA
G
AGCCGCGGTGTTCGTGACGGTGAGCAGGCAATTCCGGGCGCACTGCCGGCGATGACCGTT
TTACTGGGAAAAACCACTCTGACCACACCGGCAGGACGTGAGGGACTTTCTCCCCGGGTC
AGTTACGGAAAGGGGGCTGAGGATTTTTCACTTGTCTGGACTGCGCCTGGCGTGGCGGAA
GTGACGCTGCCTGTCACCGGAGATAATAACGTCCGGGCAGGCACATTTGTTTTCAGAATG
CAGGCTGCGGGGGTGTTGCGCCATGTGAAGGACGGACAGCCGGCGTATGCCGGTGTGTAT
GACGACCTGAATGTGAATGGTCTGCCTGGTGAAAGCGCCGCCATGAAGACTTCTGATATT
CCGAGTGTTCTGCAGAAGATGTTCAGTGGCGAAGGCCCCGGCTGGCTGCAGACAATGACC
ATCAGTGGTTATTCGGGAGTGAGTCATTTCAGTGATGCCTCCCTGCGTCAGGTTGAAGGT
GCGTATGGTGCACAGACTGTGGCAGGCAGTGGTGAATTACGTCTGAACGGCATGATGCCG

GAGCGCTGGCGGGTGTCACTGCCGGTAAGTATTGAGTACCAGTAA
>CS31A_clpH M96152.1 ~~~clpH~~~CS31A ClpH fimbrial subunit~~
ATGATGAAAATAACGCATCATTATAAATCCTTTCTTTCTGCCATTATTTCGGTGGTCCTG
TTTTATTCGGTAGCGCCACATGCAGATATTCTTGATGGCGGCGAAATTCAGTTTAATGGT
TTTGTCACTGACGAATCTCCCAAATGGACCTGGCAGATTAGTTCACCGGACCAGACCTGG
GTAGTGGATACTGCTGATGCGCGTACAGAGAATGGGCAACTGGTTTTTAATTTGCGTGAT
AAAGGGGCGTTGCCGTTTCTGGAGGGGCACTTACATGAGGTGGCTGAGCGCGGTGGTCCC
GGATTCACCCCCTTTATTACCTTCAGCAGTAACGGACAGCCTTTCGCCGTAAAGGAAGGC
AGTGGCACTACGGCGCAACGTTTTCGCGCCTCTGTTCCGGTGCGTGACCCGGAGACGGGA
AACGTGTCGGGACAGCTTTCTTTCACCCTGAATCAGGGGGTGGCAGTCAGTGCCGGTGCA
CAGGAGGAGGGGGCGACAGTGCCGGCAGGGATGTCGCTGGTCGGCGGACAGAGCGTGA
CG
GATGTACAGTCAGGAACGTTGCCACAGGGGCTTAAAACCCGCCTTTCCACATTACTTCTG
ATGAACCAGGGGGTTGGCAATGGTATGAATGCGGTGGATAACGGACAGGTTATCAGTCAG
GGGGTACTGGCTGACGGTCGGGTGATGAATCTGGCTGCGGCATATGCCTCGGTAGTGTCG
GATTTTGAACTGCGGTTGCCGGCGGAGGGTACACCTGCAGCATGGCAGGCAGGGCTGAAT
GTGACGGTCACGGTACAGTGA
>CS32_faeC 10290
ATGATATTTGTTGTGGGAGGGGGGGTGAAGATCTTTATATCGCTAATTGCTTTAATTAGTTCT
TCTGTTTATGCGGATGT
TGTTCAGCGGGTGGTTTTTAGTATGGATGTGGTTGCGTCATCCTGTCATGTGGTGGTGGATG
CGGACAGCATCGGCAACA
GTGGTCGGCTGACGTTCGGTACTTACCGCAAGTCCACGGGGGATTCTGTACCGCCGCGTG
ACTTCACTGTGCGTTTGTAT
GAGTCCGGTGCCACGGTTCAGGGGTGCTCAGCCTTTAAGGCAGGGCAGGTCGCCACCTTG
GATTTTGGTAACCCGGGACA
GCTGGATGCAGGGGGCGTGGTCACCCGCGGTGCTGGTGATGGTATTCGAGTGGATGTGCG
GGCAGTGGATACACAGGCAG
ATTATCGCGAACGTCTGACTCAGGACACTCATTCTGTGAGTTACCCGGTGGAGTTTGCTGC
TAAGGGGCAGTTCCGTTTT
CGTGCACAGCCGGTGTTTCCGGCGAACGTGAAGGCGGGGGAATACACGGGGGCACTGAC
CTTTGTGGTCACGTATCAGTA
A
>CS32_facD 10290
GTGTGTGCAGGAAAGAAGGCAGTCGCAGAGCCGGCTTCGTCCGTGTTTTTCCGTCGGGCC
GCTCTTGCGCTTGCCGTATC
AGCTGCTTTCGCCAGTGTCACAGGTATTGCAGGTGAAAAACTGGATATGTCCTTTATCCAG
GGTGGTGCCGGGGTTAATC
CGGAAATCTGGGCAGCCCTGAGCGGCAGCTATGCGCCGGGGCGTTATCTGGTTGACCTGT
CTCTGAATGGGAAGGAGGTC
GGGAAACAGATACTGGATGTGACACCACAGGACAGTAATGAACTGTGTCTGACAGAGGCA
TGGCTGACGAAGGCCGGGAT
TTACGTCAGTGCAGATTACTTTCGTGAGGGATATGACGCCACACGACAGTGCTATGTGCTG
ACAAAAGCCCTGTCAGTGA
AGGTGGATTTTGATGTTTCCACCCAGAGTCTGGCGCTGTCTATTCCCCAGAAGGGGCTGAT
GAAGATGCCGGAGAATGTG
GAGTGGGATTACGGGACCAGTGCATTTCGCGTGAATTATAACGCGAACGCCAACACCGGC
CGTAATAACACCTCGGCCTT
TGGCTCAGCGGACCTGAAAGCCAATATTGGGCGCTGGGTGGTGAGTTCTTCAGCCACGGC
CAGTGGCGGTGACAGCGGGG
ATAACTCCACCACGATAAACATGTTCACGGCCACCCGGGCCATCCGCGCACTGAGTGCGG
ACCTGGCGGTCGGGAAAACA
TCCACCGGGGACAGTCTGCTGGGCAGCACGGGAACGTACGGAGTGTCGCTGAGCCGGAA
CAACAGCATGAAGCCGGGCAA
TCTGGGGTATACCCCGGTGTTCAGCGGCATTGCGAACGGGCCGTCGAGGGTGACGCTGAC
ACAGAACGGGCGGTTGCTGC
ATTCGGAGATGGTGCCGGCAGGTCCGTTCTCCATCACGGATGTGCCGCTGTATACCAGTGG

TGATGTGACCATGAAAATC
ACCGGTGAAGACGGGCGCGAGCAGGTACAGAACTTCCCGTTGTCGGTGATGGCCGGGCA
GTTAAGCCCGGGGCAGCACGA
GTTCAGTGTGGCAGCCGGTTTGCCTGACGATGACAGTGACCTGAAAGGGGGGGTGTTTGC
TGCGTCATACGGTTACGGTC
TGGACGGGCTGACGCTGCGCACCGGGGGGGTGTTTAACCAAGACTGGCAGGGCGTCAGC
GCCGGGGCTGTTCTGGGGCTG
AGTTACCTGGGGGCGGTGTCTGCTGACGGGGCTTATGCCACGGTAAAATACCGTGACGGC
AGCCGCAGCGGAAATAAGGT
GCAGTTATCCTGGAGTAAACAACTGGATATGACGAACACCGGACTGCGGGTGAGCTGGTC
ACAGCAGAGTGAGGAGTATG
AGGATATGTCCTCCTTTGACCCGACAGATCTGTGGTCGCAGTCAAATCATGGTCGCCGGAC
GAAGGATGAATGGAATGCC
GGTGTCAGTCAACCGGTGGGTGGACTGTTCAGTCTGTCGGTGTCCGGCTGGCAGCGGAGC
TATTACCCCGCATCCACGAC
CGGGAGTTACCGGTACAGCGATGACAACGGTAAGGACACAGGTATTACCGGCACCCTGAG
CACACAGATTAAGGGCGTCA
GTCTTAACCTGGGCTGGTCCGGTTCGCGGAACACCCGGGGAGAAAACAACTGGTCTGCAT
CGGCGTCAGTGTCGGTACCG
TTCACACTGTTTGACCGTCGCTACAGCAGCAGTACGTCGGTGAGTACGAGTAAAGACGGT
GGCACAGGCTTCAGCACCGG
CGTGTCCGGCTCGCTGAATGACCGTTTCAGTTATGGTCTGGGCGGTGGGCGTGACAGTGG
TGGTGGTGTGAGCAGTTATC
TGAATGCGTCGTACAGTGGTGACCGGGCTTATCTGAATGGTGCCCTGAACCACTCGCAGTC
CGTCGGAACCAGTGGTTCT
GTATCGGTCAGCGGTTCGGTACTGGCCATTCCGGCGGCGAAAGACATTATGTTCAGCCGCA
CGACCGGGGACACCGTGGC
GGTAGTGAGCGTGAAGGATACACCCGGAGTGAAGGTGACGTCCGGTGACGGACAGACTG
ACAGTGACGGCAACTTGGTAG
TACCGCTGAACAGCTATGACTGGAACACGGTGACGATTGATGCGGGCACGCTGCCGCTGA
GTACCGAACTGATGAATACC
AGTCAGAAGGTTGTACCGACAGACAAAGCAGTGGTCTGGATGCCGTTTGATGCCCTGAAA
ATTAAGCGTTACCTGCTGCA
GGTGAAGCTGCGTGACGGTGAGTTTGTGCCGGGGGGCACCTGGGCACGTGACAGTAAGA
ACACTCCGCTGGGCTTCGTGT
CTAGTAATGGTGTGCTGATGATTAACACGGTGGATGCGCCGGGTGATATCACCCTGGGCCA
GTGCCGGATACCTGCGGCC
AGACTGCAGGATACTGAGAAACTACAGGAGATTACGTGTGAGTAA
>CS32_facE 10290
GTGAGTAAGCGTAACGCAGTAATGACGTTTTTCACTAACCGGGTGACAAAAGTACTGGGA
ATGACTCTGGCGCTGATGAT
GACCTGTCAGAGTGCGATGGCTTCCCTGGCGGCAGACCAGACCCGCTATATCTTTCGCGG
GGACAAGGATTCACTGACCA
TCACGGTCACCAACAATGACAGAGAACGTACCTTTGGTGGCCAGGCTTGGGTGGACAATA
TCGTGGAGAAGGACACCCGT
CCGACCTTTGTGGTGACGCCGTCCTTCTTCAAAGTGAAGCCGAATGGTCAGCAGACACTG
CGTATCATAATGGCCTCGGA
CCATCTGCCGAAGGATAAAGAGTCGGTGTACTGGCTGAACCTGCAGGATATTCCGCCGGC
TCTGGAGGGCAGCGGTATTG
CAGTGGCGCTGCGCACGAAGCTGAAACTATTCTATCGCCCGAAGGCACTGCTTGAAGGCC
GCAAGGGGGCAGAAGAGGGC
ATCAGCCTGCAGAGCCGCCCGGATGGCAGGATCATGCTGGTGAACACCACGCCGTATATTT
TTGCGATTGGTAGCCTGTT
GGACGGAAACGGAAAGAAAATTGCCACGGATAACGAGACGGCACAGAAACTGCTGATGT
TCATGCCGGGTGATGAAGTAC
AGGTGAAGGGAAATGTGGTGAAAGTTGACTCTCTGAATGACTGGGGAAGTCTGCAGACC
TGGACTATTAACCAGAAGAAA

CTGGCAGTACCGGAAGTAACAAAAGCTGATACTGCAGAGTAG
>CS32_facF 10290
ATGAAGAAAACAATGATGGCAGTCGCCTTGGCTCTGAGTGCTCTCAGTATTCCGTCAGCA
CTGGCCGCTGAATACAGCGA
GAAAACGCAGTATCTGGGTGTAGTGAATGGTCAGGTGTCAGGTAACAGCGTGGTGAAGGT
AAACCGCACACCGACAGACC
CGGTGTTGTACCGGAGTGGAGACACGACGCCTCTTCCCGGCAGCCTGACCATCCGCAACG
CAGAGTCTAGGGCAGCATCC
GGTGGCCTGGCGTATATCACGGTGAAGCAGGTGTTACCGGATAACGGGGAAGCCCGCATC
ACCCTGAAGACGGCACTGAT
GGTTGACGGAAAGAAAGTGGCACTCAGTGCCAGACAACAGGGTGAGGATATGGTGATCA
CTGTTCCGGAGGCACACACGC
AGGTTGAGCTAAGAACCGATGCGCCGATAGAACTGGAAGTGCCGGTCACTTACCGGGGA
AACCTGCAGATAGCCCTGCAG
GTGGAGAGCTGA
>CS32_facG 10290
ATGAAAAAGACCCTGATTTCACTGGCTGTGGCTGCTGGTGCTGTTATGTCCGTATCCAGTG
TAGCTAATGCATATACTCC
TTCATTTTCTAACGGAGATATTTCTTTAGGCGGTACTATTAGCACTCCTGTGCAAGCTGCTC
TGTATGAAGGTAAGGTTG
GTTCTCTTTCCGGGCTTGATGCGACTATCCCTGTCGGTGCAAGCACAGTGACTATTGCTGC
ACCTGCAAATGCGGGACTT
TTAGCATTGCGTTCTGTAGCAGGTGGGTTTAATAATACGGTCTCAGATAGAATCGCTAATAT
CACCTTTAATGGAGTGAG
CTTGCTGGACGCAGCTTCCGGTGATTCTTTCAGTAATGGCGCCATTCAGATGACCTTGGGT
GTCCAGGATGACACTGGTA
GTGCAATTGGTGATGTTGTTTTTCCGATGCAAGTAGCAGCTGTTATCACCACAGAAACCAA
TGGTGATGTGTGGGGGACT
TCTATGTTTGCTTCTGCAGACAATCATGCCTTTTATGGTGGACTGCCTACATCAGCTTCTCA
GGCTGTAACGCTGTATAG
TGATGCTGTCAATGTCATGAACGGACTGTTCTCTGATGTTACTGCCAATCTCCCTCAAATTA
CCCGTGAATCAGCTAAGG
CGGAAGTGAAAAATTTCGCTTTAGCTGATGCAGTATTTAATACTGCATACGCGGCTGGTATT
GTTATGGGGGAAAACATT
ACTATTAATCTAGACAATCCAGCGACAACAGGAAGCGTGGCTTGGACTGCATCTATGCCTA
TTGTTGTTACCTATAAATA
A
>CS32_facH 10290
TTGGATGGTGGCGAAATTCTGTTTAATGGCTTTGTCACTGACGAAGCTCCCAAATGGACTT
GGCAGATAAGTTCTCCCGA
CCAGATTTGGGCGGTGGATACTGCCGATGCGCGCACGGAGAACGGGCAACTGGTTTTTAA
TTTGCGTGATAAAGGAACGC
TGTCGTTTCTGGAGGGGCATTTGCATGAGGTGGCTGAGCGCGGTGGTCCCGGATTCACGC
CATTTATTAGCTTCAGCAGT
AACGGTCAGCCTTTCGCCGTAAAGGAAGGCAGTGGCACAACAGCGCAACGTTTTCGCGC
CTCTGTCCCGGTGCGTGACCC
GGAAACGGGGAACGTATTGGGACAGCTTTCTTTCACTCTGAATCAGGGGATGGCTGTCAG
TGTCGGTAGACAGGAAGACG
GGGGAGCTGTGCCGGCCGGGATGTCGCTGGTCAGTGGACAGAGCGTGACGGATGTACAA
TCGGGAAATCTGCCTCAAGGG
CTGAAAAACCGTCTATCTTCATTATTACTGATGAATCAGAATTTTGGAAATGGCATGAATGC
GGTGGATAACGGACAGGT
TATCAACCAGGGAATATTGGCTGACGGTCGAGTGATGGATCTGGCTGCGGCATATGCTTCT
GCTGTGTCGGATTTTGAAC
TGCGGTTACCGGCAGAGGGTACACCGGCCAGCTGGCAGGTGGGGCTGAATGTGACGGTT
ACAGTACAGTGA
>CS32_facI 10290

ATGAAAAAGGTGACGCTGTTTCTGTTTGCTGTCAGCCTCCTGCCCTCCACAGTGCTGGCCT
GGAATACACCGGGAGAAGA
TTTTAGCGGCGAGCTTAAGCTGGAAGGCCCGGTGACCAGTACCCGTAATCCGTGGGTATG
GAAAGTCGGACAGGGAAATG
AAAGCCTGGAGGTTAAACAGAGCCGTAGTGTTCGTGATGGTGAGCAGGGAATTCCGGTTG
CACTGCCGGCGATGACCGTT
TTACTGGGAAAAACTACCCTGACCACTCCGGCAGGACGTGAGGGGCTTTCACCCCGGGTC
AGTTACGGAACGGGCGGTGA
TTTTTTTTCACTTGTCTGGACAGCACCTGGCATGGCGGAAGTGACGCTGCCTGTTATCGGA
GATAATAATGTCCGGGAAG
GTTCATTTATTTTCAGAATGCAGGCGACCGGGGTATTGCGCTATGTGAAGGACGGACAGCC
GGTGTATGTCGGCGTGTAT
GACGATCTTAATGCGAATGGTCTGCCTGGTGAAAGCAATTCTATGAAAGTTTCTGATATTCC
GGGGGTTCTGCAGAAGAT
GTTTAGTGGTGAAGGCCCCGGCTGGATGCAGACAATGACGGTGACGGTTTCAGAGGGGCT
AAGTCGTTTCAATGATGTCT
CCCTGCGTCAGGTTGAAGGTGCGTATGGTGCTCAGACTGTAGCAGACAGTGGTGAATTAC
GTCTGAACGGCACGATGCCG
GAACGCTGGCGGGTGTCACTGCCGGTAAGTATTGAGTACCAGTAA
>CS32_facJ 10290
GTGCTGAATATTATTCATCGTCTGAAGTCCGGTATGTTTCCGGCTCTGTTTTTCTGACTTCA
GCCAGTGTGCTGGCACA
ACCTCTGATTATTCCGCCGGGCCATTGGCAGGAGGGAATGGCTGTGGGCGTGACGGAATT
CAGCGGCACGCTGTATGTTC
CTGAAGTTTCCTGGCAGTGGCAACCCCGCGCTGTACGGATGGCTACACCAGATGCCGTGC
ATGCCGGTCTGACAACGGGT
AAAAGTGGCATGGTCAGTGAAAGCCGGGCAGGGCAGGATTTTTATATTCTTGGCGGACAT
ACCACATCGCTGACAAGCTC
CCGTCCCGGGCTGCAGCCGTCAGTGACATTACTTCAGGTGATTCCATCATCTCCCCGGATT
GCGGCCCGGGGAGAACTTG
CCCGGGGGCAGATGCGTTACGGGGAAATCACGTTTACCGTTCGTCACCTGTTGGCATGGC
AGGACAATATCACAGCTGGT
CAGGGCTGGAGTGTGGTTAGCGGAGAGGTGACACCGGAGGCAGAACGGCAGGTGAAAC
GTCTGTTATGGCAGGTGAGGGG
CTATGAATGGACACCGAACTATGCAGGATTAACCGCACGTCCTGATGCGTTTATTTCAGGG
GCAGAGTCGCTGTTGTCAC
AGGAAAATGACAGAAAGTATATTGTCGGAGCCTGGGTGACATCACTGAGTGATGTGCGGG
TGAATTTCCCCGGTGCTGAG
GAGCCGGTAAAACGCTGGCAGGGGAGTTTGACGCCGGTAGTAGCCTATTTCTGA
>CS33_faeC HH-P024
ATGGATGTGGTTGCATCATCCTGTCATGTGGTGGTGGATGCGGACAGCGTCGGCAACAGTG
GCCGGCTGATGTTTGGGAC
TTACCGCAAGTCCACGGGAGTGTCTGTGCCCCCGCGTGACTTCACTGTGCGTCTGTATGAG
TCCGGCGCCACGGTTCAGG
GCTGTTCAGCCTTTAAAGCAGGACAGGTCGCCACCCTGGATTTTGGTAACCCGGGACAGC
TGGACGCAGGGGGCGTGGTC
ACCCGCGGTGCCGGTGATGGTATTCGCGTGGATGTGCGGGCGGTGGATGCGCAGGCAGAT
TATCGCGGACGTCTGACTCA
GGATAGTCATTCGGTGAATTACCCGGTGGAGTTTGCTGCTAAAGGACAGTTCCGTTTTCGT
GCGCAGCCGGTGTTTCCGG
CGAACGTGAAGGCGGGGGAATACACAGGGGCGCTGACCTTTGTGGTCACGTATCAGTAA
>CS33_facD HH-P024
ATGTCCTTTATCCAGGGTGGTGCCGGGGTTAATCCGGAAGTCTGGGCAGCCCTGAACGGT
AGCTATGTACCGGGGCGTTA
TCTGGTTGACCTGTCCCTGAACGGGAAGGAGGTCGGGAAACAGATACTGGATGTGACACC
ACAGGACAGTAATGAGCTGT
GTCTGACAGAGGCATGGCTGACGAAGGCCGGGATTTACGTCAGTGCAGATTACTTTCGTG

AGGGATATGATGCCACACGA
CGGTGCTATGTGCTGACAAAAGCCCCGTCAGTGAAGGTGGATTTTGATGTTTCCACCCAG
AGTCTGGCACTGTCCATTCC
CCAGAAAGGGCTGGTGAAGATGCCGGAGAATGTGGACTGGGATTACGGGACCAGTGCAT
TTCGCGTGAACTATAACGCGA
ACGCCAACACCGGCCGTAATAACACCTCGGCCTTTGGCTCAGCGGACCTGAAAGCCAATA
TCGGGCGCTGGGTGGTGAGT
TCTTCTGCCACGGCCAGTGGCGGTGACAGCGGGGATAACTCCACCACGATAAACATGTTT
ACGGCCACCCGGGCCATCCG
CGCACTGAGTGCGGACCTGGCGGTCGGGAAAACATCCACCGGGGACAGTCTGCTGGGCA
GTACGGGAACGTACGGCGTGT
CGCTGAGCCGGAACAACAGCATGAAGCCGGGCAATCTGGGGTATACCCCGGTGTTCAGCG
GCATTGCGAACGGGATGTCG
AGGGTGACGCTGACACAGAACGGGCGGTTGCTGCATTCGGAGATGGTGCCGGCGGGTCC
GTTCTCCATCACGGATGTGCC
GCTGTACACCAGTGGTGATGTGACCATGAAAATCACCGGAGAGGACGGGCGTGAACATGT
GCAGAACTTCCCGCTGTCGG
TGATGGCCGGGCAGTTAAGCCCGGGGCAGCACGAGTTCAGTGTGGCAGCCGGTTTGCCTG
ACGATGACAGTGACCTGAAA
GGCGGTGTGTTTGCGGCGTCATACGGTTACGGTCTGGACGGGCTGACGCTGCGCACCGGG
GGGGTGTTTAACCAGGACTG
GCAGGGCGTCAGCGCCGGGGCTGTTCTGGGGCTGAGTTACCTGGGGGCGGTGTCTGCTG
ACGGGGCTTATGCCACGGCAA
AATACCGTGACGGCAGCCGCAGCGGAAATAAGGTGCAGTTATCCTGGAGTAAACAACTGG
AGATGACGAACACCGGACTG
CGGGTGAGCTGGTCACGGCAGAGTGAGGAATATGAGGACATGTCCTCCTTTGACCCGACA
GAGTTGTGGTCGCAGTCAAA
TCATGGTCGCCGGACGAAGGATGAATGGAATGCCGGTGTCAGTCAGCCGGTGGGTGGACT
ATTCAGTCTGTCGGTGTCCG
GCTGGCAGCGGAGCTATTACCCCGCATCCATGACCGGGAGTTACCGGTACAGCGATGACA
ACGGTAAGGACACGGGGATT
ACCGGTACCCTGAGCACACAGATTAAGGGCGTCAGTCTTAACCTAGGCTGGTCCGGTTCA
CGGAACACCCGGGGAGAAAA
CAACTGGTCTGCATCGGCGTCAGTATCGGTACCGTTCACACTGTTTGACCGTCGCTATAGC
AGCAGTACGTCGGTGAGTA
CGAGTAAAGACGGAGGCACTGGCTTCAGCGCCGGCGTGTCAGGCTCGCTGAATGACCGT
TTCAGTTATGGTCTGGGCGGT
GGGCGTGACAGCGGTGGTGGTATGAGCAGTTATCTGAATGCGTCGTACAGTGGTGACCGG
GCTTATCTGAATGGTGCCCT
GAACCACTCGCAGTCCGGCGGAACCAGTGGTTCTGTCTCGGTCAGCGGTTCGGTACTGGC
CGTTCCGGCGGCGAAAGACA
TTATGTTCAGCCGCACGACCGGGGATACCGTGGCCGTGGTGAACGTGAAGGATACACCCG
GAGTGAAGGTGACGTCCGGT
GACGGACAGACTGACAGTGACGGCAACTTGGTGGTACCGCTGAACAGCTATGACTGGAA
CACGGTGACGATTGATGCGGG
CACACTGCCGCTGAGCACCGAACTGACGAACACCAGTCAGAGGGTGGTGCCGACGGACA
AGGCGGTGGTGTGGATGCCGT
TTGATGCCCTGAAAGTTAAGCGTTACTTTCTGCAGGTGAAGCAGCGTGACGGTGAGTTTG
TGCCAGGGGGAACCTGGGCA
CGTGACAGTAAGAACACACCGCTGGGCTTTGTAGCTAACAATGGTGTGCTGATGATTAAC
ACGGTGGATGCGCCGGGGGA
TATCACCCTGGGCCAGTGCCGGATACCTGCGGTCAGACTGCAGGATACTGAGAAACTACA
GGAGATTACGTGTGAGTAA
>CS33_facE HH-P024
GTGAGTAAGCGTAACGCAGTAACGACGTTTTTCACTAACCGGGTGACAAAAGCACTGGGA
ATGACTCTGGCGCTGATGAT
GACCTGTCAGAGTGCGGTGGCCTCCCTGGCGGCAGACCAGACCCGTTATATCTTTCGCGG

GGACAAGGATGCACTGACCA
TCACGGTCACTAACAATGACAAGGAGCGTACCTTTGGTGGTCAGGCCTGGGTGGACAATA
TCGTGGAGAAGGACACCCGT
CCGACTTTTGTGGTGACGCCGTCCTTCTTCAAGGTGAAGCCGAATGGTCAGCAGACACTG
CGTATCATCATGGCCTCGGA
CCATCTGCCGAAGGATAAAGAGTCGGTGTACTGGCTGAATCTGCAGGATATTCCGCCGGCC
CTGGAGGGAAGCGGTATTG
CAGTGGCGCTGCGCACGAAGCTGAAACTGTTCTACCGCCCGAAGGCACTGCTTGAAGGC
CGCAAGGGGGCAGAGGAAGGG
CTAAGCCTGCAGAGCCGCCCGGATGGCAGGACCATGCTGGTGAACACCACGCCGTACATT
TTTGCGATTGGCAGCCTGCT
GGATGGAAACGGGAAGAAAATTGCCACGGATAATGAGACGGCACAGAAAATGCTGATGT
TCATGCCGGGTGATGAAGTAC
GGGTTAAGGGGAATGTGGTTAAGGTTGCTTCACTGAACGATTACGGTGAACTGCAGACCT
GGACAATTAACCAGAAAAAA
ACGCCAGCGACTTCCGAGCAGAAGGTATCTGATTCGCCGGTTAATTTGTCTGATAAAGCAG
ATAAAAAATAA
>CS33_facF HH-P024
ATGAAGAAAACAATGATGGCAGTCGCCCTGGCCCTGAGTGCACTCAGTATTCAGTCAGCA
CTGGCCGCTGAATACAGCGA
GAAAACGCAGTATCTGGGGGTGGTGAATGGTCAGGTGTCAGGTAACAGTGTTGTGAAGGT
GACCCGCACACCGACAGACC
CGGTGTTGTACCGGAGTGGAGACACGACGCCTCTTCCCGGCAGCCTGACCATCCGCAGCG
CAGAGTCCAGGGCTGCATCA
GGTGGCCTGACATATATCACGGTGAAGCAGACTCTGCCGGATAACGGGGAAGCCCGCATC
ACCCTGAAGACGGCCCTGAT
GGTTGACGGAAAGAGAGTGGCACTCAGTGCCAGACAGCAGGGTGAGGATGTGGTGATAG
CCGTTCCGGAGGCACAGAAGC
TGGTTGAGTTAAGAACGGATGCACCGGCAGAGCTGGAAGTCCCGGTCAGTTACCGGGGA
AATCTGCAGATAGCGCTGCAG
GTGGAAGACTGA
>CS33_facG HH-P024
ATGAAAAAGACTCTGATTACACTAGCTGTGGCTGCAGCTGCGGCAGTATCTGGTTCAGTGA
TGGCTGCTGATTGGACGGA
AGGTCAACCAGGTGACATTATTATTGGTGGCGAAATTACGTCATCATCTGTTAAGTGGCTAT
GGAAGACTGGAGATGGAC
TTTCATCTCTTAGTAATACTACAAATGAAATTGTTCAACGGAAGTTGAATATTTCTGTTCCA
ACGGATGAATTATTTTTA
GCAGCGAAGATGAGTGATGGGGTTCAAGGTGTTTTCGCAGGGAATACACTCATTCCTAAG
ATCGAAATGGCATCTTATGA
TGGTAGTGTTATTATACCTAGTTTCACTTCAAATACAGCAATGGATATTGCTGTAAAAGTAA
AAAACTCAGGTGATAATA
CTGAACTAGGGACTCTTTCTATTCCTTTGTCATTTGGTGCGGCAGTTGCAACTATTTTTGAT
GGCGATACTACTGATAGC
GCTGTAGCGGATATTACCGGTGGTTCTGCTGGTACAGTATTTGAAGGGCTGGTTAATCCAG
GTAGTGTTACTGATCAGAA
TATAGCCTATAAATGGAATGGACTCTCAAAAGCTGAAATGGCTGGTTATGTAGAAAAGTTA
ATGCCAGGGCAAAGTGCTT
CAACCTCTTATACTGGTTACCACAATTGGAATGAGCTCACTCACGATAACTATACTTCTGCA
AATAAAGCATCTTATCTC
TCTTATGGATCTGGTGTTTCTGCAGGTAGTACTTTAGTTATGGATTTAAATAAGGATGTTGC
GGGTCGACTTGAATGGGT
GGCTCCAGTGACTATCACCGTTACTTATAGTTAA
>CS33_facH HH-P024
ATGATGAAAACAGTATGTCACTATAAATTTATTCTTTCAGCCATTATTTCGGTGGCTCTGTTT
TATTCAGCAGCGCCACA
TGCAGATATTCTTGATGGTGGCGAAATTCAGTTTAATGGTTTTGTCACTGACGAAGCTCCC

AAATGGACCTGGCAGATTA
GTTCACCGGACCAGACCTGGGCAGTGGATACTGCTGATGCGCGAGCAGAGAATGGACAA
CTGGTTTTTAATTTGCGTGAC
AAAGGGCCCTTGCCGTTTCTGGAAGGGCATTTGTATGAGGTGGCTGAGCGCGGAGGTCCC
GGATTCACGCCATTTATTAC
CTTCAGCAGTAACGGACAGCCTTTCACCGTAAAGGAAGGCAGTGGCACTACGGCACAAC
GTTTTCACGCCTCTGTCCCCG
TGCGTGATCCGGAGACGGGAAATGTGTCAGGACAGCTTTCTTTCACCCTGAATCAGGGGA
TGGCAGTCAGTGCCGGCAGA
CAGGAAGACGGAGCATCCGTACCAGTCGGGATGTCGCTGGTCAGTGGGCAGAGCGTGAC
GGATGTACAGTCAGGAACGCT
GCCACAGGGGCTGAAAGCCCGACTCTCTTCTCTGTTACTGATGAGTCAGAATTTTGGGAAT
GGCATGACTGCGGTGGATA
ACGGTCAGGTTATCAGTCAGGGGGTATTGGCTGACGGTCGCGTGATGAATCTTGCTGCGGC
ATATGCCTCTGTAGTATCG
GATTTTGAACTGCGGTTACCGGCAGAGGGTACACCTGCAGCGTGGCAGGCGGGCCTGAAT
GTGACTGTCACAGTGCAGTG
A
>CS33_facI HH-P024
ATGAAAAAGATGACGTTGATTCTATACACTGTCAGTCTCCTGCCTTCCTGTGTACTGGCTTG
GAGTACGCCGGGAGAGGA
TTTCAGTGGAGAGCTGAATCTGGAAGGTTCGGTGACCAGCACCCGTAATCCATGGATGTG
GAAAATCGGACAGGGGAATG
AACGTCAGGAGGTTAAACTGAACAGCGGTGTTCGTGACGGTGGTCAAGGGATTCCGGTT
GCACTACCAGAGATGACCGTT
TTACTGGGAAAAACCACCCTGACCACATCGGCAGGACGAGAAGGGCTTTCACCCCGGGT
CAGTTACGGAAAGGGCACTGA
GGATTTTTCACTCGTCTGGACAGCACCTGGCATGGCGGAAGTGACGCTGCCTGTCACCGG
AGATAATAATGTCCGGGAAG
GTTCATTTATTTTCAGAATACAGGCGACCGGGGTATTGCGCCATGTGAAGGACGGACAGCC
GGTGTATGTCGGCGTGTAT
GACGATCTGAATGCGAATGGTCTGCCTAGTGAAAGCAATTCTATGAAATTTTCTGATATTCC
TGGGATTCTGCAGAAGAT
GTTCAGTGGCGAAGGCCCTAGCTGGCTGCAGACAATGACAGTCAGTGGTTATTCGGGCGT
GAGTCATTTCAGTGATGCCT
CCCTGCGTCAGATTGAAGGTGCATATGGCGCACAGACTGTGGCAGGCAGTGGTGAATTAC
GTCTGAACGGTGCGATGCCG
GAACGCTGGCGGGTGTCACTGCCGGTAAGTATTGAGTACCAGTAA
>CS33_facJ HH-P024
GTGCTGAATATTATTCATCGTCTGAAGTCCGGTATGTCTCCGGCTCTGTTTTTTCTGACTTC
AGCCAGTGTGCTGGCACA
ACCTCTGATTATTCCACCGGGATACTGGCAGGAGGGAATGACTGTAGGCGTGACGGAATTC
AGCGGCACGTTGTATGTTC
CTGAGGTTTCCTGGCAGTGGCAGCCCTGCGCTGTGCGGATGAGTACATCAGACGCCGTGC
AGGTTGGTCTGACAACGGGT
AAAAGTGGAATGGTCAGTGAAAGCCGGACAGGGCAGGATTTTTATATTCTTGGCGGGCAT
ACAGCATCACTGACAACCGC
CCGTCCGGGGCTGCAGCCGTCAGTGACATTGCTTCAGGTGACACCGTCATCCCCCCGTATT
GCGGCCCGGGGAGAACTCG
CCCAAGGGCAGGTGCGTTACGGGGAAATCACGTTTACGATGCGCCATCTTCTGGCATGGC
AGGACAATATCACTGCCGGC
CAGGGCTGGAGCGTGGTCAGTGGAGAGGTGACGCCGGGGGCCGAAAAGCAGGTGAAAC
GCCAGTTATGGCAGGTGAACGG
CTATGAATGGACCCCGGACTATGGCGGGTTAACCGCACGTCCTGATGCGTTTATTTCAGAG
GCAGAGTCGCTGTTGTCAC
AGGAGAATGGCAGACAACATATTGCCGGTGCATGGGTGACATCCCTGAGTGATGTGCGGG
TGAATTTCCCCGGTGCTGAG

GAGCCGGTAAAACGCTGGCAGGGGAATCTAACTCCGGTGGTGGTGTACTTCTAA
